# Mechanism of Melatonin Metabolism by CYP1A1: What Determines the Bifurcation Pathways of Hydroxylation versus Deformylation?

**DOI:** 10.1021/acs.jpcb.2c07200

**Published:** 2022-11-16

**Authors:** Thirakorn Mokkawes, Ze Qing Lim, Sam P. de Visser

**Affiliations:** † Manchester Institute of Biotechnology, The University of Manchester, 131 Princess Street, Manchester M1 7DN, U.K.; ‡ Department of Chemical Engineering, The University of Manchester, Oxford Road, Manchester M13 9PL, U.K.

## Abstract

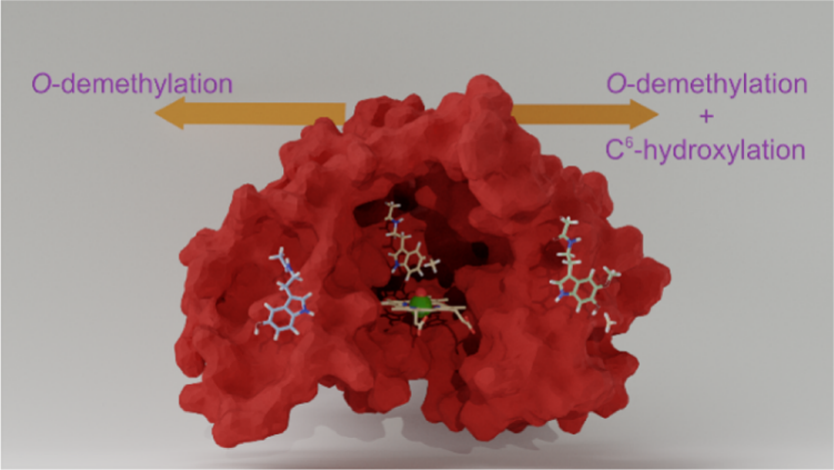

Melatonin, a widely applied cosmetic active ingredient, has a variety of uses as a skin protector through antioxidant and anti-inflammatory functions as well as giving the body UV-induced defenses and immune system support. In the body, melatonin is synthesized from a tryptophan amino acid in a cascade of reactions, but as melatonin is toxic at high concentrations, it is metabolized in the human skin by the cytochrome P450 enzymes. The P450s are diverse heme-based mono-oxygenases that catalyze oxygen atom-transfer processes that trigger metabolism and detoxification reactions in the body. In the catalytic cycle of the P450s, a short-lived high-valent iron(IV)–oxo heme cation radical is formed that has been proposed to be the active oxidant. How and why it activates melatonin in the human body and what the origin of the product distributions is, are unknown. This encouraged us to do a detailed computational study on a typical human P450 isozyme, namely CYP1A1. We initially did a series of molecular dynamics simulations with substrate docked into several orientations. These simulations reveal a number of stable substrate-bound positions in the active site, which may lead to differences in substrate activation channels. Using tunneling analysis on the full protein structures, we show that two of the four binding conformations lead to open substrate-binding pockets. As a result, in these open pockets, the substrate is not tightly bound and can escape back into the solution. In the closed conformations, in contrast, the substrate is mainly oriented with the methoxy group pointing toward the heme, although under a different angle. We then created large quantum cluster models of the enzyme and focused on the chemical reaction mechanisms for melatonin activation, leading to competitive O-demethylation and C^6^-aromatic hydroxylation pathways. The calculations show that active site positioning determines the product distributions, but the bond that is activated is not necessarily closest to the heme in the enzyme–substrate complex. As such, the docking and molecular dynamics positioning of the substrate versus oxidant can give misleading predictions on product distributions. In particular, in quantum mechanics cluster model I, we observe that through a tight hydrogen bonding network, a preferential 6-hydroxylation of melatonin is obtained. However, O-demethylation becomes possible in alternative substrate-binding orientations that have the C^6^-aromatic ring position shielded. Finally, we investigated enzymatic and non-enzymatic O-demethylation processes and show that the hydrogen bonding network in the substrate-binding pocket can assist and perform this step prior to product release from the enzyme.

## Introduction

**Scheme 1:**
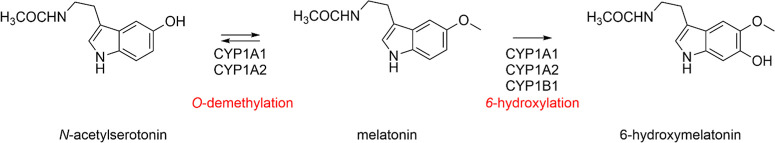
Metabolic Pathways of Melatonin by Various P450 Isozymes

Melatonin, known under the chemical name *N*-acetyl-5-methoxytryptamine, is a hormone secreted by the pineal gland in the brain.^1,2^ It is also expressed in skin cells and cutaneous tissue, where it is both synthesized and metabolized (Scheme 1).^3−6^ Research on melatonin has attracted considerable attention through its applications to the cosmetics industry that uses it as an antioxidant and skin immune strengthener. It is believed that to some extent, melatonin is more suitable than vitamin C, vitamin E, and phenolic acid for these purposes as it does not appear to generate highly toxic hydroxyl radicals.^7−12^ Melatonin also protects the body against UV-induced oxidative stress-mediated damaging through the melatoninergic anti-oxidative system.^3,9^ This process eliminates the reactive oxygen species in the skin by the biotransformation of melatonin to 2-hydroxymelatonin, 4-hydroxymelatonin, and consecutively to *N*^1^-acetyl-*N*^2^-formyl-5-methoxykynuramine. Recently, suggestions have been made that melatonin may have therapeutic properties against the Covid-19 virus.^13,14^ Melatonin and its metabolites are strongly lipophilic and are metabolized in the body by the cytochrome P450 CYP1A subfamily.^15,16^

The cytochrome P450s are a superfamily of heme proteins, mostly with mono-oxygenase activity, which are biological catalysts that metabolize endogenous compounds such as hormones, bile acids, cholesterol, and xenobiotics including environmental pollutants and drugs.^17−27^ The P450s have been classified into four families based on their exogenous metabolism. In particular, the P450s from family 1 are interesting from a biological and toxicological viewpoint because of their affinity to oxidize polycyclic compounds, aromatic amines, aromatic hydrocarbons, and endogenous compounds, thereby reducing the toxicity of these metabolites.^27,28^ The P450 isozymes CYP1A1 and CYP1A2 have been reported as the main enzymes that metabolize melatonin, see Scheme 1.^29^ CYP1A2 and CYP1A1 are the hepatic and extrahepatic enzymes, respectively; however, the level of each enzyme in the skin varies.^29^ Thus, the CYP1A subfamily generates 6-hydroxymelatonin and *N*-acetyl-5-hydroxytryptamine (*N*-acetylserotonin) via aromatic hydroxylation and O-demethylation of melatonin, respectively.

Human CYP1A1 and CYP1A2 are closely related, and their amino acid sequences show a 72% identity.^30,31^ However, the F helix of CYP1A1 is extended by five residues, which results in additional π-stacking interaction between a conserved phenylalanine in the F-helix. This interaction is believed to give the substrate greater flexibility in binding and orientation, as compared to other P450 isozymes.^32^ As CYP1A1 has fewer side chains pointing into the active site with respect to CYP1A2, this results in a larger substrate-binding pocket and active site with larger cavity volume. As a consequence of this, the product distributions of substrate activation are different between CYP1A1 and CYP1A2 isozymes. In particular, Ma et al.^33^ obtained 75% 6-hydroxylation and about 10% O-demethylation products for CYP1A1 activation of melatonin. Although substrate hydroxylation by the P450s is a common reaction pathway, for several substrates, P450 isozymes have been shown to react via an O-demethylation pathway, where the methoxy group is initially hydroxylated but in a subsequent step comes off as formaldehyde and results in the formation of a phenol product.^34−38^

For the principal metabolism pathway of melatonin, there are intriguing questions, for example, related to the products that are favored, namely *N*-acetyl-5-hydroxytryptamine or 6-hydroxymelatonin, and particularly how the P450 isozyme directs the reaction into a specific product channel. As recently, a new high-resolution structure of CYP1A1 was resolved,^39,40^ we decided to utilize it and perform a detailed study on melatonin metabolism using molecular dynamics (MD) and quantum mechanical methods. Extensive previous experimental and computational studies on a variety of P450 enzymes identified an iron(IV)–oxo heme cation radical species, also called compound I (CpdI), as the active species of the catalytic cycle.^17−26,41−45^ Therefore, the calculations focused on substrate activation by CpdI in the work presented here. In addition, we attempt to answer the question on how the chemoselectivity of melatonin activation is determined. Quantum chemistry methods have been widely adopted in the past to provide useful information about short-lived intermediates that are difficult to trap and characterize experimentally. The present work aims at clarifying the above questions to offer the missing insight and reveal mechanistic details at the theoretical level. Our work shows that even though substrate can potentially bind in different orientations, actually the stability of some substrate-binding poses gives major differences in energy. Furthermore, the aromatic hydroxylation pathway is inherently lower in energy than the primary C–H bond activation of the methoxy group. However, local interactions and a hydrogen bonding network stabilize the aliphatic C–H hydrogen atom abstraction and reduce it in energy so that it becomes competitive with the aromatic hydroxylation pathway.

## Methods

### Model Setup and MD Simulations

The computational studies were performed using well tested and benchmarked procedures, and all details are given in the Supporting Information while we give a brief summary here.^46−48^ In general, the work starts from a deposited crystal structure from the protein databank that is setup to study the problem of interest.^39^ In our particular case, the work started from the 6DWN protein databank (pdb) file, which is a resting state CYP1A1 structure with erlotinib bound.^40^ We selected chain A of the pdb file as it is the most complete chain (Supporting Information, Figure S1). Erlotinib was removed from the structure, and substrate melatonin was docked into the substrate-binding pocket of the pdb file using the AutoDock Vina software package,^49^ which resulted in four low-energy binding poses for the substrate in the substrate-binding pocket (protein models **I**, **II**, **III**, and **IV**), Figure S2. We decided to analyze all four of those and run MD simulations on each of these binding poses to determine the flexibility and mobility of the substrate in the binding pocket for each of the conformations.

Hydrogen atoms were added to the protein models at pH = 7 in Chimera,^50^ whereby all carboxylate groups were present in their deprotonated forms, while the Arg and Lys side chains were taken as protonated. The protonation states of histidine residues were manually assigned based on a visual inspection of the hydrogen-bonding surrounding, and all were in their singly protonated state. His_352_ and His_464_ were protonated on the N_δ_ atom, while all other His residues were protonated on the N_ε_ atom. The pdb was converted into a CpdI structure by adding an oxygen atom at a distance of 1.686 Å from the iron atom trans to the axial cysteinate group (Cys_457_). Parameters for the CpdI structure of each P450 model were generated with the MCPB.py routine as implemented in the Amber software package, while for the description of the protein atoms, the ff14SB4 force field parameters were used.^51,52^ Thereafter, the model was inserted in a rectangular water box of TIP3P molecules in Amber with a minimum distance of 10 Å from the box boundary.^53^ Counterions (Na^+^ and Cl^–^) were added to the surface of the protein to neutralize the system. The ff14SB force field and a general Amber force field were used for the equilibration, heating, and MD simulations,^54,55^ whereby the system was initially minimized by 2000 steps of steepest descent with all heavy atoms fixed. After that, the system was heated to 310 K for 100 ps under *NPT* conditions without any geometric constraints in Amber. Next, each of these four substrate-bound protein models were subjected to separate MD simulations for 100 ns in a production run in the Amber 16 software package.^55^ Snapshots of the MD simulation were stored every 100 ps and analyzed in detail (see the Supporting Information).

### Cluster Model Setup

**Scheme 2:**
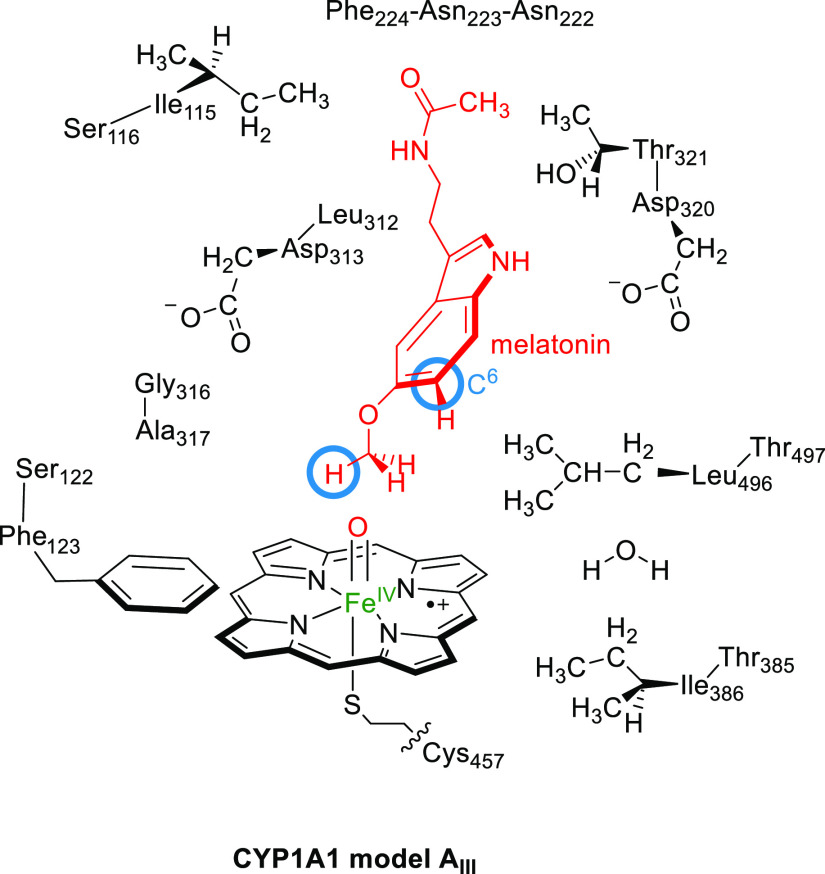
DFT Cluster Model of CYP1A1 Studied in This Work with the C^6^-Position and Methoxy C–H Groups that are Being Activated Highlighted with a Blue Circle

Based on the results of the MD simulations, we created several active site cluster models of CYP1A1 with the substrate in different orientations with respect to the heme. These cluster models have been used extensively in various groups before and generally give a good reflection for enzymatic reaction mechanisms.^46,56−59^ The 97 ns snapshot of the MD simulation for model **III** was used to create cluster model **A**_**III**_. The cluster model studied in this work is shown in Scheme 2 and contains the iron(IV)–oxo heme (with side chains truncated to hydrogen atoms) linked to ethylmercaptane for Cys_457_. In addition, a large part of the substrate-binding pocket was included that provides second-coordination sphere interactions such as hydrogen bonding and dipole moment interactions. In particular, the amino acid chains Ile_115_-Ser_116_, Ser_122_-Phe_123_, Asn_222_-Asn_223_-Phe_224_, Leu_312_-Asp_313_, Gly_316_-Ala_317_, Asp_320_-Thr_321_, Thr_385_-Ile_386_, and Leu_496_-Thr_497_ were included, whereby Asn_223_ was truncated to Gly. The overall system had 308 atoms and included one active site water molecule and had overall charge −2. All structures were calculated in the lowest energy doublet and quartet spin states, as identified with a superscript 2 or 4 before the label.

We also created a cluster model from the MD simulation of substrate-binding pose **II** using the 97 ns snapshot, namely model **A**_**II**_. For consistency, the model had the same atoms as those for model **A**_**III**_ but was expanded with the side chain of Asn_255_ that forms a hydrogen bonding interaction with the amide proton of the melatonin substrate and hence had 317 atoms in total.

### Computational Methods

The active site cluster models were investigated with density functional theory (DFT) methods, and a reaction mechanism between CpdI and substrate was explored using Gaussian-09.^60^ We used the unrestricted B3LYP density functional method in conjunction with the LanL2DZ basis set with core potential on iron and 6-31G* basis set on carbon, hydrogen, oxygen, nitrogen, and sulfur: basis set BS1.^61−65^ Mixed basis sets of this type have been found highly suitable for calculations on large cluster models.^66−68^ Local minima and transition states were minimized using the UB3LYP/BS1 protocol, and the connectivity between the stationary points was established using a geometry scan. Transition states were confirmed by harmonic frequency analysis and possess only one imaginary frequency for the correct mode while the stationary points were confirmed as the local minima with all real frequencies.

To improve the energetics, single-point calculations were performed with the continuum polarized conductor model with a dielectric constant mimicking chlorobenzene (ε = 4.7)^69^ and a 6-311+G* basis set on H, C, N, O, and S atoms and either LANL2DZ (with core potential) on iron (basis set BS2) or the cc-pVTZ basis set on iron (basis set BS3).

## Results and Discussion

### MD Simulations on Substrate Binding, Positioning, and Mobility

**Figure 1:**
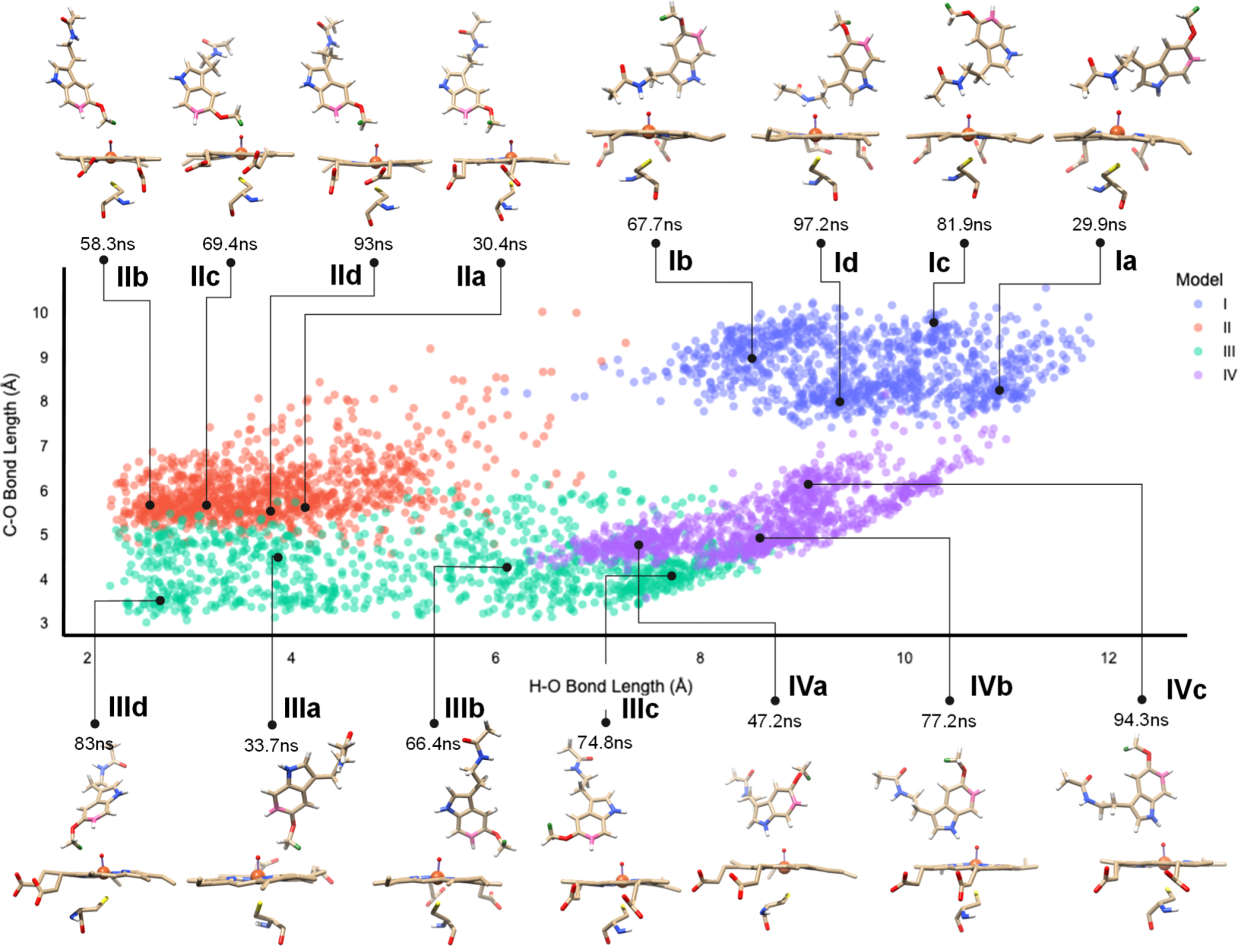
Scatter plot of substrate orientation with respect to CpdI during the MD simulation. Each dot represents an individual snapshot from the MD simulations for model **I**, **II**, **III**, and **IV** and displays the distance of the oxo group of CpdI vs the nearest hydrogen atom of the methoxy group on the *x*-axis, while its distance to the C^6^-atom of the aromatic ring of the substrate (atom highlighted in pink in the structures) is present on the *y*-axis. The data points represent structures from the MD simulations starting from binding position **I** (blue dots), **II** (orange dots), **III** (green dots), and **IV** (purple dots). Representative snapshot structures of the substrate and CpdI orientations at specific time points along the MD simulations are displayed at the top and bottom.

To find out what factors affect the regioselectivity of O-demethylation versus 6-hydroxylation of melatonin by CYP1A1 isozymes, we pursued a computational study. We started with docking of the substrate into the CYP1A1 pdb file and located four strong binding conformations (**I**, **II**, **III**, and **IV**). In conformation **I**, the *N*-acetyl bond of the substrate points toward CpdI, while the methoxy group is upward and forms hydrogen bonding interactions with the Thr_497_ residue. This interaction is also seen for substrate binding in conformation **IV**; however, it has the indole proton hydrogen bonding to CpdI. In conformation **II** and **III**, the *N*-acetyl bond of the substrate points up, and hydrogen bonds with the Asn_222_ and Asn_255_ residues, while the methoxy group is lower in the substrate-binding pocket and closer to CpdI than that in conformations **I** and **IV**. We then ran 100 ns MD simulations for each of these substrate-binding poses **I**, **II**, **III**, and **IV** and analyzed the structural changes and substrate orientations and summarize these in Figure 1. In each of the simulations, the structures equilibrated within a 30 ns timescale and gave a stable root-mean-square-deviation (rmsd) of the overall structure (Figures S3 and S4, Supporting Information). Indeed, in all cases, the substrate was relatively rigid and stayed in virtually the same position in the binding pocket throughout the full MD runs. The hydrogen bonding interactions found during the MD simulations are summarized in the Supporting Information, and as can be seen, most of those stay in place during the complete MD run. These rmsd values; therefore, imply that hydrogen bonding interactions of the substrate with protein residues lock it in a tight orientation, and despite the fact that CYP1A1 has a large and open binding pocket, the substrate mobility appears to be limited.

Figure 1 shows the distance distribution of the substrate with respect to the oxo group of CpdI for the potential activation of the C^6^-aromatic carbon atom and the methoxy group for the four MD simulations for conformations **I**, **II**, **III**, and **IV**, whereby each snapshot of the MD simulation is represented with a dot. For each MD simulation, three or four representative structures of heme versus substrate are shown, labeled as **a–d**. As can be seen from Figure 1, the individual snapshot structures for model **I**, **II**, **III**, and **IV** are very similar, and very little substrate movement is seen during each of the 100 ns MD runs. The C^6^–O distance for each substrate-binding pose stays within a narrow window of about 2 Å during each of the MD simulations, possibly due to hydrogen bonding interactions of the indole and amine proton donor interactions with protein residues. This will mean that the aromatic ring will be highly rigid and will be difficult to activate when it is located far away from CpdI. Indeed, in orientation **I**, it is located at the largest distances from the CpdI oxo group. Moreover, the methoxy group is also at a large distance from CpdI in substrate-binding model **I**, which implicates that this binding pose is unlikely to be catalytically active. Therefore, the model **I** structures from the MD simulations were not used for further studies on the reaction mechanisms and reaction pathways. As can be seen, the shortest C^6^–O distances are found for conformation **III** with a median distance of about 4 Å during the MD simulation (Figures 1 and S5, Supporting Information); hence, this binding pose is expected to be the ideal orientation for C^6^-activation of the substrate. In contrast, the median C^6^–O distances for the MD simulations of models **I**, **II**, and **IV** are 8, 6, and 5 Å, respectively. These distances are relatively large and will have to incur considerable structural changes in order for C^6^-hydroxylation reactions to occur. Consequently, the MD simulations indicate that only in substrate-binding position **III**, aromatic hydroxylation is a likely reaction channel.

Interestingly, the binding poses of the methoxy group of the substrate with respect to CpdI span a large distance in the set of MD simulations. As shown in Figure 1, in some MD snapshots, the C–H bonds of the methoxy group are in hydrogen bonding interaction with the oxo group of CpdI, that is, several model **II** and **III** structures, while the methoxy group is at a much further distance in models **I** and **IV** with a median of well over 9 Å from CpdI. In orientation **I**, the substrate is located with the *N*-acetyl group pointing toward the heme and held in position with a hydrogen bond with the alcohol group of Thr_497_. In contrast, in orientation **IV**, the melatonin indole N–H bond forms a hydrogen bond with the oxo group of CpdI, while the *N*-acetyl group is locked in an interaction with the carboxylate of Asp_313_. These MD simulation results therefore show that substrate-binding orientations **I** and **IV** are unlikely to lead to methoxy group activation and O-demethylation reactions. Furthermore, in both systems also the C^6^–O distance is relatively large and consequently both binding poses can be ruled out as catalytically active orientations and were not pursued further. Finally, in orientations **II** and **III**, the *N*-acetyl group points upward in the binding pocket due to hydrogen bonding interactions of the *N*-acetyl group with the side chains of Asn_222_ and Asn_255_. In both of these orientations, the C^6^-carbon atom and *O*-methyl groups of melatonin point toward the heme and hence are the most likely orientations for substrate activation. Based on the C^6^-oxo and methoxy-oxo distances from the snapshots in the MD simulations, we predict possible O-demethylation reactions in substrate-binding orientations **II** and **III** and possible aromatic hydroxylation at the C^6^-position in substrate-binding orientation **III**. If the nearest distance of substrate from CpdI determines catalysis and enzymatic turnover, then the different binding orientations, as shown in Figure 1, will lead to differences in product distributions by CYP1A1.

Apart from the structural analysis of the MD simulation snapshots, we also investigated the binding energies of the structures (Figure S7c, Supporting Information). The lowest energy conformations are found for the model **II** and **III** conformations with the indole and methoxy groups pointing into the substrate-binding pocket and the substrate peptide bond at the top. However, the median geometry changes in model **II** are relatively large, which implies less rigidity of the substrate and protein environment. Moreover, in model **II**, the L-helix moves considerably from its original position in the crystal structure coordinates and hence may not be an accurate representation of the real system (see Figure S9, Supporting Information). Nevertheless, to find out if these protein changes affect catalysis, we created DFT cluster models based on the last snapshot of the MD simulation for model **II** and **III**.

**Figure 2:**
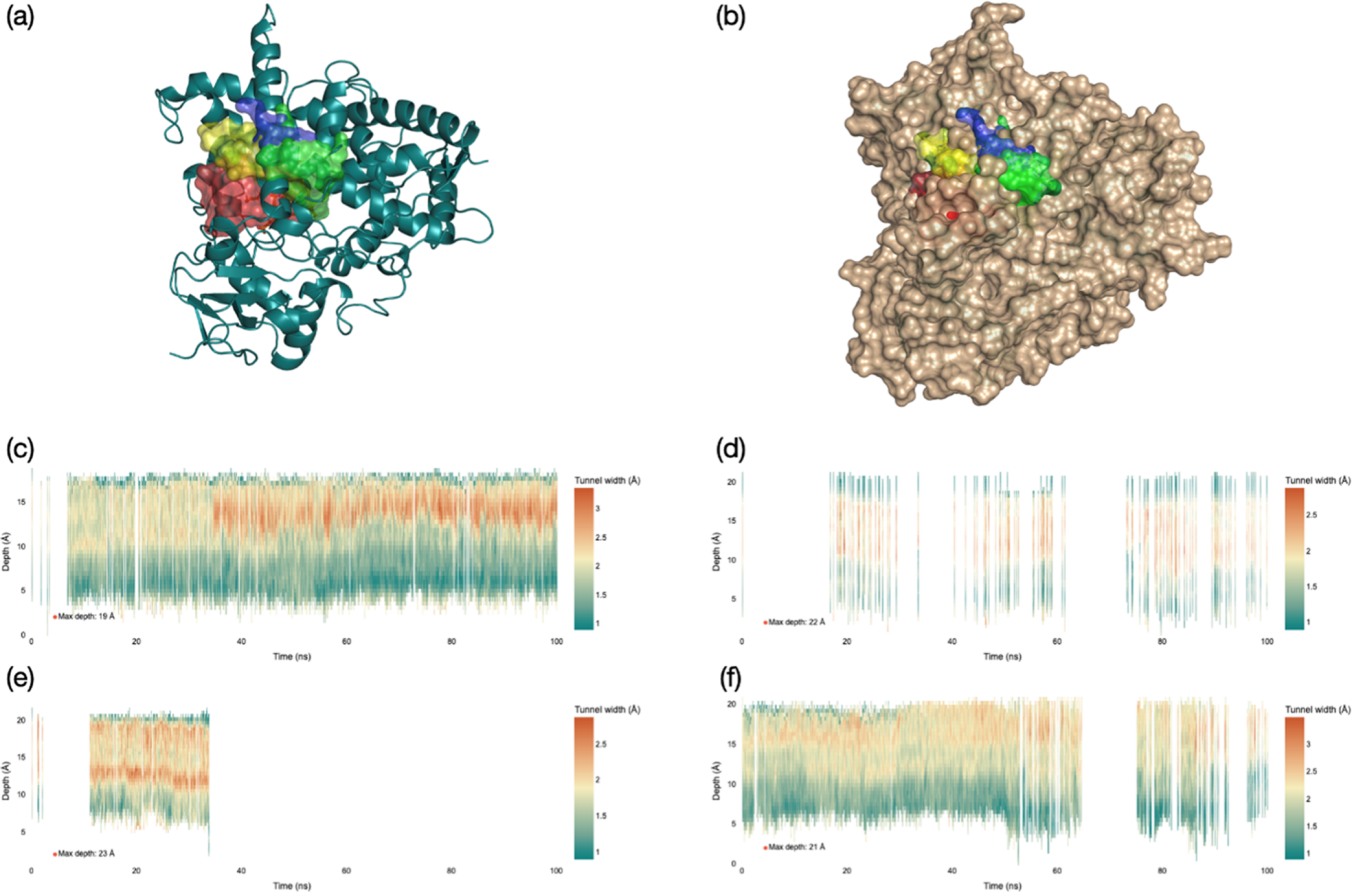
Tunneling analysis for the entrance and exit channels in CYP1A1, leading to CpdI as obtained from the individual snapshots from the MD simulations for 100 ns with substrate starting orientation **I**, **II**, **III**, and **IV**. (a) Protein structure with the major tunnels for model **I** (in red), model **II** (in green), model **III** (in blue), and model **IV** (in yellow). (b) Same as part (a) but with the protein surface highlighted. (c–f) Tunneling information per snapshot (time frame on the *x*-axis) for the four MD simulations for model **I** (part c), **II** (part d), **III** (part e), and **IV** (part f). The horizontal axis represents the time segment from the MD simulation, while on the *y*-axis, the depth of the tunnel from the surface is given in Å. For each binding orientation, the deepest tunnel is shown.

To further support our choices of model **II** and **III** for the quantum mechanics (QM) cluster calculations, we did a tunneling analysis on the MD snapshots using the CAVER software package.^70^ As can be seen from Figures 2 and S10, S11 (Supporting Information), there are distinct differences in the protein structure with substrate bound in positions **I**, **II**, **III**, and **IV**. The largest channels for each model are highlighted on the protein structure in Figure 2a,b and show that each channel reaches the surface of the protein. There are differences, however, in the size of these channels, whereby the entrance channel for model **II** and **IV** appears relatively wide, it is small for model **I**. Thus, in structures **I** and **IV**, the enzyme is relatively open with various channels permanently leading into the active site with deep and wide channels entering the protein, Figure 2c,f. Indeed, Figure 2c shows a relatively wide tunnel with width larger than 3 Å from the surface into the active site for most snapshots along the MD simulation for binding pose **I**. This implies that the substrate-binding orientation **I** keeps the substrate-binding pocket open and water accessible at all times. Moreover, it may lead to an equilibration between a binding and non-binding situation for the substrate, that is, release of the substrate back into solution. To a lesser extent, this is also the case in binding pose **IV**, where one representative tunnel shows a presence for most snapshots along the MD simulation.

This tunnel appears to be somewhat lesser wide than the one in model **I**; hence, substrate release may not be as easy as for model **I**. In contrast, in substrate-binding orientation **III**, the protein starts in an open conformation (Figure 2e) with a tunnel leading into the heme active site. After about 35 ns MD simulation, the tunnel disappears, as well as the other tunnels for model **III** (Supporting Information, Figure S11). Our work implies that in substrate-binding position **III**, the substrate will be locked in the substrate-binding pocket and will not be able to escape easily. In substrate-binding orientation **II**, in contrast, the protein seems to switch between open and closed forms continuously during the MD simulation, and no clear open or closed regions are seen. Nevertheless, in the majority of the MD snapshots for model **II**, the substrate-binding pocket is closed. These tunneling analysis results in combination with the MD simulations highlight clear differences in the protein structure as a result of substrate-binding orientation. In some poses, the substrate-binding pocket does not appear to close, and substrate can escape back into solution, while in other poses, the substrate-binding pocket is closed. As a result, the open binding pocket will retain the equilibrium of bound versus non-bound substrate into the heme and may lead to substrate release prior to its activation. Interestingly, the MD simulations with a closed substrate-binding pocket have the substrate bound in an orientation that is set up for O-demethylation or C^6^-aromatic hydroxylation, which are the dominant products observed for melatonin activation by CYP450 1A1.

Further evidence that structures **I** and **IV** have open active site conformations comes from an analysis of the rmsd values for specific group/loops of the protein. Thus, the F-helix of the protein has been implicated with closing the substrate-binding pocket.^71,72^ For substrate-binding orientations **I** and **IV**, the rmsd values for the F-helix are large (Figure S4, Supporting Information), while they are much smaller and close to the values of the rest of the protein for the MD simulations of orientations **II** and **III**. These data implicate that the F-helix is much less rigid in models **I** and **IV** than that in models **II** and **III** and lead to an open substrate-binding pocket with tunnels connecting the surface with the heme center.

### QM Cluster Calculations on CpdI Models

**Figure 3:**
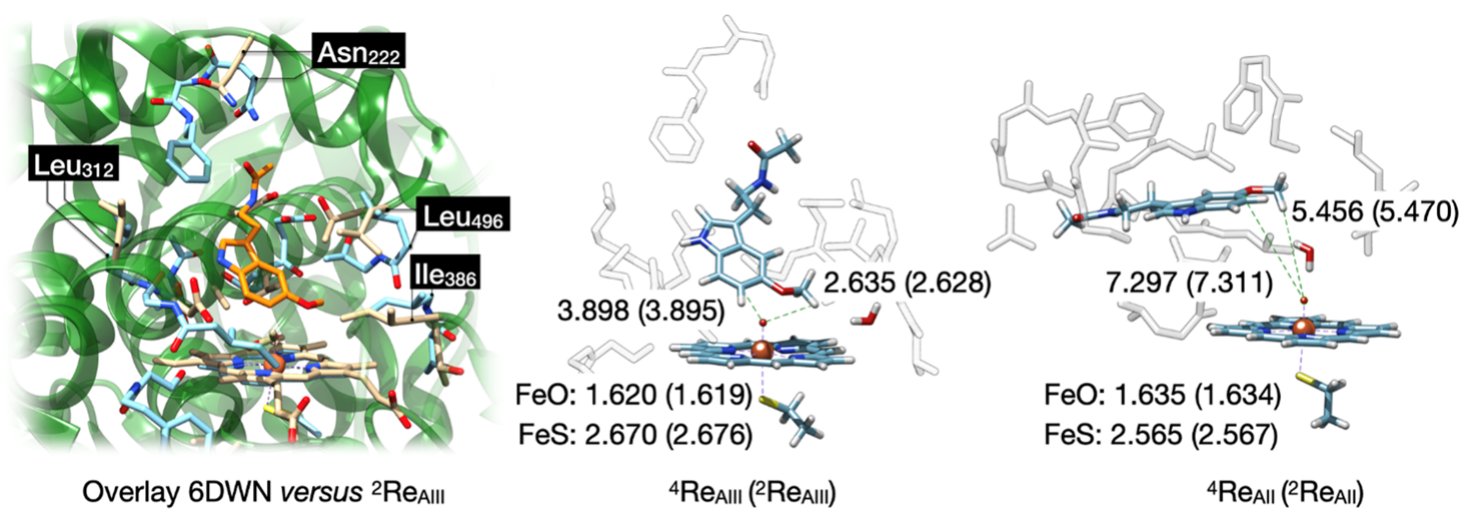
(Left) Overlay of the active site of chain A of the 6DWN pdb file (green ribbons and beige atoms) with ^2^**Re**_AIII_ (light blue). The substrate is shown in orange. (Right) QM cluster optimized geometries of the CYP1A1 reactant complexes with melatonin bound as obtained in the quartet and doublet (data in parenthesis) spin states. Bond lengths are in angstroms.

Next, we created quantum mechanical cluster models of the substrate- and oxidant-binding sites and studied the reaction mechanisms for O-demethylation and C^6^-hydroxylation of the substrate by CYP1A1 with DFT methods. In particular, we generated cluster models on low-energy snapshots of the MD simulations for substrate pose **II** (model **A**_**II**_) and substrate pose **III** (model **A**_**III**_) to obtain the reactant complexes for model **A**_II_, that is **Re**_AII_, and for model **A**_III_ as **Re**_AIII_. To validate the models, we started the quantum chemical work with a geometry optimization of the melatonin-bound reactant structures ^4,2^**Re**_AII_ and ^4,2^**Re**_AIII_ of the CYP1A1 model and compare the structures with crystal structure data and previous calculations. Optimized geometries of the reactant complexes are given in Figure 3.

An overlay of the original CYP1A1 pdb structure of the 6DWN file with the optimized geometry of ^2^**Re**_AIII_ is shown on the left-hand side of Figure 3. Most residues are in similar positions in ^2^**Re**_AIII_ as in the crystal structure, and particularly, the protein chains of the model match the positions of those in the crystal structure well. For instance, we highlight four residues on different sides of the model and in the protein pocket in the overlay, namely, Asn_222_, Leu_312_, Ile_386_, and Leu_496_. As can be seen, these residues are in approximately the same position in the crystal structure and the cluster model. Therefore, the model captures all features of the substrate-binding pocket and oxidant and reproduces the three-dimensional shape of the protein and heme-binding pocket. Moreover, the overlay implies that little geometric changes have occurred during the geometry optimizations and that the model is a good representation of the CYP1A1 substrate-binding pocket.

The iron(IV)–oxo bond is short, and we find values of 1.62 Å for model **A**_**III**_, while they are 1.63 Å for model **A**_**II**_. These values are typical for heme-based iron(IV)–oxo complexes and match earlier calculations on smaller CpdI cluster models and results obtained with QM/molecular mechanics (QM/MM).^25,73−85^ Moreover, they match the experimental extended X-ray absorption spectroscopy (EXAFS) that reported an Fe–O distance of 1.67 Å.^86^ The Fe–S bond lengths in our CpdI models are relatively long (2.67–2.68 Å in ^4,2^**Re**_AIII_ and 2.57 Å in ^4,2^**Re**_AII_); however, as this represents the interaction between two second row elements, it is not an unusual value and is in line with previous studies. In particular, previous calculations on P450 CpdI with alkylmercaptane as mimic for cysteinate typically gave long Fe–S distances as seen here as well, see a comparison of our calculated distances with those from previous work in Table S26, Supporting Information.^74,76^ All of these reported computational studies on P450 CpdI found somewhat longer Fe–S distances than those derived experimentally from EXAFS,^86^ although crystal structure coordinates of an NO-bound iron(III) complex of cytochrome P450 reported a much longer Fe–S bond length of 3.1 Å.^87^ Consequently, there is a large variation in Fe–S distances in the literature obtained from the experiment and theory. In all cases, CpdI can be considered as a triradical system with unpaired electrons in π*_xz_, π*_yz_, and a mixed a_2u_-axial ligand orbital.^41,44,88^ The latter is a mixed porphyrin–thiolate orbital, where the lone pair sulfur orbital mixes with the porphyrin a_2u_ orbital. In the quartet spin state, all of these orbitals have an up-spin electron, while in the doublet spin, the π* electrons are up-spin and the a_2u_ is down-spin. Nevertheless, the two spin states are close in energy; within 1 kcal mol^–1^, for both models. Consequently, a situation should be considered of close-lying spin states and a multistate reactivity patterns with barriers on the doublet and quartet spin state.^44,89^ An analysis of the group spin densities of our CpdI calculated structures indeed gives a spin of about 2 on the FeO group in the doublet and quartet spin structures for model **A**_II_ and **A**_III_ (ρ_FeO_ = 2.01–2.10) as evidence for single occupation of the π*_xz_ and π*_yz_ orbitals. Although the orbital analysis reveals single occupation of the a_2u_ orbital, the spin densities give dominant SCH_3_ (ρ_SMe_) radical with values of −0.59 for ^2^**Re**_AII_, 0.53 for ^4^**Re**_AII_, −0.71 for ^2^**Re**_AIII_, and 0.68 for ^4^**Re**_AIII_.

The latter values are a perfect match with those obtained by Thiel, Shaik, and their co-workers^74^ using QM/MM and a 6-31G* basis set on sulfur, who obtained spin densities of −0.73 and 0.66 on the SCH_3_ group for doublet and quartet CpdI. Similarly, DFT calculations on large QM cluster models of P450 CpdI that employ a 6-31G* basis set or better on the axial thiolate ligand give analogous spin distributions over the sulfur and porphyrin groups as, for instance, seen for the lignin-activating enzyme P450 GcoA.^90^ The substrate is oriented in such a way that in all cases, the nearest interaction with the oxo group is from one of the methoxy hydrogen atoms. In ^4,2^**Re**_AIII_, the methoxy hydrogen atom is at a distance of 2.63 Å for both structures. In the ^4,2^**Re**_AIII_ structure, the C^6^-position of melatonin is at a somewhat longer distance of 3.90 Å. Based on these substrate-binding positions, the methoxy activation appears to be the most likely reaction channel for model **A**_III_. Furthermore, these substrate-to-CpdI interactions are close to those seen in the model **III** MD simulation result and again show that little geometric changes have occurred during the structure minimization of the reactant complexes.

For the model **II** structures, the methoxy group points toward CpdI, and its nearest interaction of a hydrogen atom with the oxo group is at 5.5 Å for ^4,2^**Re**_AII_ with a water molecule in between the two groups. In contrast, the C^6^-position of melatonin is at large distances of 7.3 Å in the quartet and doublet spin states, respectively. As such, the substrate orientation in model **A**_II_ is further away from CpdI than that in model **A**_III_, which may affect reaction barriers and catalysis. Moreover, in both structures, two potential reaction channels are still possible, namely, C^6^-activation and methoxy hydroxylation. To find out whether these differences in substrate orientation would trigger changes in catalysis and product distributions, we expanded our work with catalytic reaction mechanism calculations. In particular, we considered multiple reaction pathways on the doublet and quartet spin states for the two substrate-binding conformations.

### QM Cluster Studies on O-Demethylation by CpdI

**Scheme 3:**
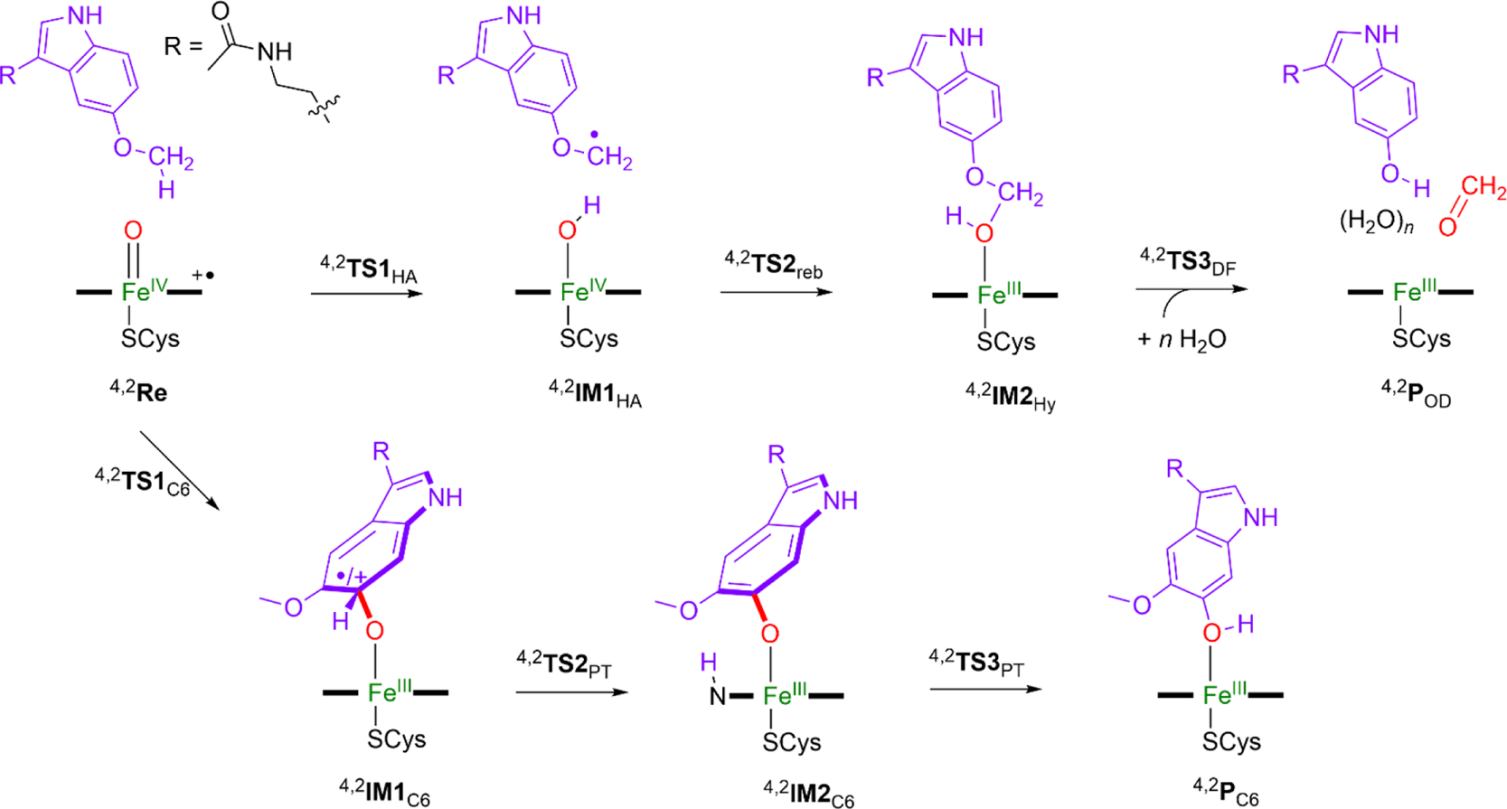
Reaction Mechanisms Investigated in This Work with Definition of the Labels of the Local Minima and Transition-State Structures

Experimental data showed that the overall reaction of P450-catalyzed melatonin metabolism proceeds mainly via either O-demethylation or aromatic hydroxylation.^33^ The two mechanisms were investigated with DFT methods for model ^4,2^**Re**_AII_ and ^4,2^**Re**_AIII_, according to the pathways displayed in Scheme 3. First, the mechanisms of O-demethylation start with a hydrogen atom abstraction from the methoxy side chain of melatonin via a transition-state **TS1**_HA_ to form an iron-hydroxo species (**IM1**_HA_), which is also called compound II (CpdII). Thereafter, an OH rebound barrier via transition-state **TS2**_reb_ gives hydroxylated products (**IM2**_Hy_). In the final stage, a proton transfer takes place that releases formaldehyde and gives the O-demethylation products (**P**_OD_) via a barrier **TS3**_DF_. It has been proposed that the proton relay for the final O-demethylation step is guided by several solvent molecules;^91^ hence, this was studied with additional water molecules present. Furthermore, the step can take place outside the enzyme with external protons, whereby the hydroxylated product releases formaldehyde to give *N*-acetyl-5-hydroxytryptamine. The deformylation pathway was tested for the hydroxylated product separately with nearby H_2_O molecules (up to three) that assists in the proton relay in a mechanism suggested by Shaik et al.^91^ The alternative reaction pathway (bottom reaction in Scheme 3) leads to aromatic hydroxylation of melatonin. The aromatic hydroxylation reaction is initiated with the formation of a σ-complex intermediate through an electrophilic transition-state **TS1**_C6_ to give the radical or cationic intermediate **IM1**_C6_. The latter transfers its *ipso*-proton to the heme via a proton-transfer transition-state **TS2**_PT_ to form intermediate **IM2**. A subsequent proton shuttle generates 6-hydroxymelatonin products (**P**_C6_) via transition-state **TS3**_PT_. We initially investigated the O-demethylation pathway of melatonin, as catalyzed by CYP1A1 using models **A**_II_ and **A**_III_, which is initiated by the methoxy group hydroxylation by CpdI.

**Figure 4:**
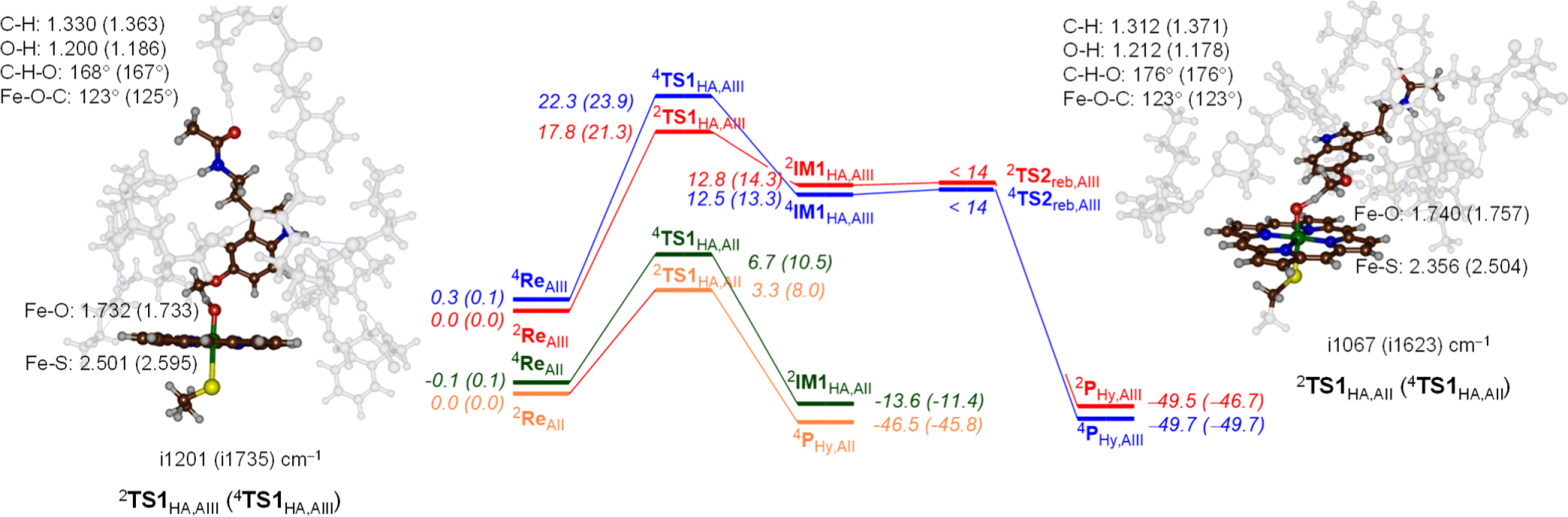
Reaction energy profile for methoxy-group hydroxylation of melatonin by CpdI model **A**_**II**_ and **A**_**III**_ of CYP1A1. Energies represent UB3LYP/BS3//UB3LYP/BS1+ZPE + *E*_solv_ data in kcal mol^–1^, while optimized geometries give bond lengths in angstroms, bond angles in degrees, and the imaginary frequency in cm^–1^.

The obtained energy landscapes in the doublet and quartet spin states for the model **A**_**II**_ and **A**_**III**_ cluster structures are given in Figure 4. In all models, the hydrogen atom abstraction barrier is rate-determining, and the OH rebound via **TS2**_reb_ is negligible. Geometry scans for the steps via **TS2**_reb,AIII_ are shown in the Supporting Information (Figure S27) and implicate that the intermediates will have short lifetime, and as soon as the hydrogen atom transfer has occurred, the system will relax to the alcohol product complex. Our potential energy landscape, therefore, is similar to previous DFT studies on O-demethylation and aliphatic hydroxylation of the substrate by the P450s.^91−100^ The hydrogen atom abstraction barriers for CYP1A1 have values of Δ*E* + ZPE = 17.8 (22.3) kcal mol^–1^ in the doublet (quartet) spin states for substrate bound orientation **III**, respectively, while they are Δ*E* + ZPE = 3.3 (doublet) and 6.7 (quartet) kcal mol^–1^ for model **A**_**II**_. The ^2^**TS1**_HA,AIII_ value is within 1 kcal mol^–1^ of what was obtained with a minimal cluster model for the terminal C–H abstraction for *n*-propane and hence is what would be expected for a terminal C–H bond.^101,102^ The addition of the protein to the model, therefore, appears to have little effect on the hydrogen atom abstraction barrier for the model **A**_**III**_ structure. In contrast, the model **A**_**II**_ transition state are well lower in energy, and consequently, these are stabilized by the protein environment and polar interactions in the pocket.

To understand the differences in barrier height for the model **A**_**II**_ and model **A**_**III**_ transition states, we analyzed the optimized geometries of ^4,2^**TS1**_HA,AIII_ (for binding orientation **III**) and ^4,2^**TS1**_HA,AII_ (for binding orientation **II**), as shown in Figure 4. In all four transition states, the structures are late on the potential energy surface with short O–H distances of 1.178–1.212 Å and much longer C–H distances in the range 1.312–1.371 Å. In the model **A**_**III**_ transition-state structures, the C–H–O angle is somewhat bent (at 167–168°), whereas it is closer to linearity for the model **A**_**II**_ structures (176°). Under ideal circumstances, these hydrogen atom abstraction barriers give linear C–H–O angles that lower their barriers.^44,89−115^ As such, the model **A**_**II**_ structures manage to position the substrate better than the model **A**_**III**_ structures for better electron transfer, and therefore, the barriers for model **A**_**II**_ are significantly lower in energy. Probably, this is caused by the tight substrate-binding pocket that latches the substrate in a specific orientation and prevents the ideal linear hydrogen atom transfer angle for model **A**_**III**_. Nevertheless, most distances for the structures for the model **A**_**II**_ and **A**_**III**_ transition states appear to be similar, and the energetic differences must result from substrate positioning that is stabilized in model **A**_**II**_ over model **A**_**III**_.

The imaginary frequencies for the hydrogen atom abstraction barriers are large: i1067 cm^–1^ for ^2^**TS1**_HA,AII_, i1623 cm^–1^ for ^4^**TS1**_HA,AII_, i1201 cm^–1^ for ^2^**TS1**_HA,AIII_, and i1735 cm^–1^ for ^4^**TS1**_HA,AIII_. The imaginary frequencies, therefore, are not dramatically different between the model **A**_**II**_ and model **A**_**III**_ structures and would implicate similar kinetic isotope effects for replacing hydrogen by deuterium due to a similar shape of the potential energy profile around the transition state. These values are typical for hydrogen atom abstraction transition state and seen before for P450 model reactions.^89−115^ The large imaginary frequency in the hydrogen atom abstraction transition states will result in a large kinetic isotope effect and a significant amount of quantum chemical tunneling for the reaction.^116,117^ After the radical intermediate, a small rebound barrier via **TS2** leads to the hydroxylated methoxy group products ^4,2^**P**_Hy,A_ with large exothermicity. From the hydroxylated methoxy group, a subsequent proton relay from the alcohol group to the methoxy oxygen atom leads to deformylation. However, this step can happen inside the substrate-binding pocket or outside the protein environment in water.

**Figure 5:**
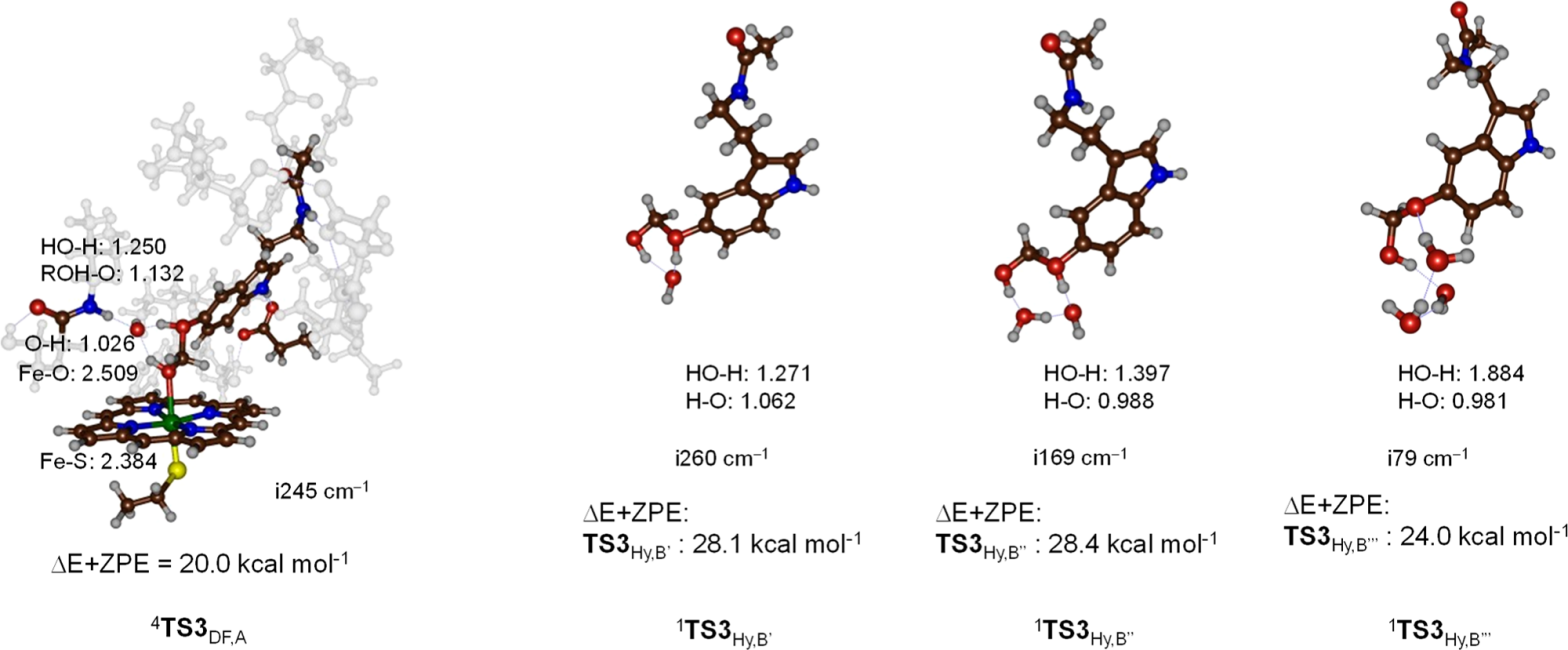
UB3LYP/BS1-optimized O-demethylation transition states in the heme pocket or in a water solution with bond lengths in angstroms, the imaginary frequency in cm^–1^, and UB3LYP/BS3 calculated relative energies with ZPE included with respect to the ^4^**P**_Hy,A_ intermediate in kcal mol^–1^. The reaction energies of the hydroxylated melatonin with 1, 2, and 3 water molecules are calculated relative to a complex of hydroxylated melatonin with 1, 2, or, 3 water molecules in kcal mol^–1^.

First, we explored the direct deformylation of the bound hydroxylated product (^4^**P**_Hy,AIII_) via transition-state ^4^**TS3**_DF,AIII_ to form the deformylated product ^4^**P**_OD,AIII_. With the assistance of an active site water molecule that bridges the methoxy oxygen atom and the alcohol proton atom, we find a proton-transfer transition states for the doublet and quartet spin states, see Figure 5. The imaginary frequency of ^4^**TS3**_DF,AIII_ is i245 cm^–1^ and represents simultaneous proton transfer from the alcohol group to the methoxy oxygen atom with a stretch vibration in the C–O bond. Consequently, the transition state implicates deformylation through proton transfer. Structurally, the geometry is reactant-like with a short alcohol O–H distance of 1.026 Å in the quartet spin state. The distance of the proton to the methoxy group is 1.382 Å. The barriers are Δ*E* + ZPE = 20.0 kcal mol^–1^ for ^4^**TS3**_DF,AIII_ with respect to the ^4^**P**_Hy,AIII_ complex. This large barrier would implicate that the hydroxylated products will have a long and finite lifetime that therefore could be detected experimentally. However, as no evidence of these products exists, we searched for lower energy pathways.

We then created models **B′**, **B″**, and **B‴**, which contain the hydroxylated melatonin with one, two, and three water molecules added to the system to calculate the water-assisted O-demethylation reaction outside the protein, see Figure 5. Generally, when three water molecules are added to the chemical system, the barrier drops significantly in energy; however, in all models **B′**, **B″** and **B‴**, the barrier is larger than that in the protein matrix. Thus, with one extra water molecule included, the proton transfer and deformylation step is Δ*E* + ZPE = 28.1 kcal mol^–1^ with respect to a complex of the hydroxylated melatonin with one water molecule. Adding an extra one or two water molecules to the model (as in **B″** and **B‴**) gives barriers of 28.4 and 24.0 kcal mol^–1^ for the two systems. The absolute barrier of the latter with respect to the reactant complex **IM2**, therefore, is of similar magnitude to the protein-assisted barrier ^4^**TS3**_DF,A_. These results match previous studies on analogous O-demethylation reactions.^91^ Nevertheless, a water environment may not have a sufficiently acidic pH to enable proton transfer to the methoxy group, leading to deformylation. Moreover, the comparison of enzymatic deformylation via ^4^**TS3**_DF,AIII_ and the model **B‴** transition state shows that the protein environment gives lower energy barriers for this process than the same reaction in water due to stabilizing hydrogen bonding interactions that assist the proton relay. Thus, the amide group of the peptide bond between Leu_496_ and Thr_497_ interacts with the hydrogen-bonding network involved in the O-demethylation reaction, while the carboxylate of Asp_313_ forms a hydrogen bond in the indole proton of the substrate. These calculations show that the hydrogen bonding network in the substrate-binding pocket and the polar interactions of the negatively charged Asp residue in the substrate-binding pocket assist the O-demethylation stage of the reaction. Furthermore, our calculations show that the reaction is not enhanced in the water solution/environment, and we do not see a lowering of the barriers with only water molecules surrounding the hydroxylated substrate.

As shown by previous studies on the O-demethylation mechanism, a hydroxylated methoxy group can split off formaldehyde through, for instance, a proton-assisted step with the involvement of a H_3_O^+^ ion. Although we did create models of **B′** and **B″** with a proton added, we were unable to characterize transition states for these processes. Nevertheless, the general geometry scans show that proton transfer will happen rapidly with small barriers and large exothermicity. This conclusion agrees with previous computational studies and experimental work on O- and N-demethylation reactions by heme proteins.^91−94,118,119^

### QM Cluster Studies on Aromatic Hydroxylation by CpdI

**Figure 6:**
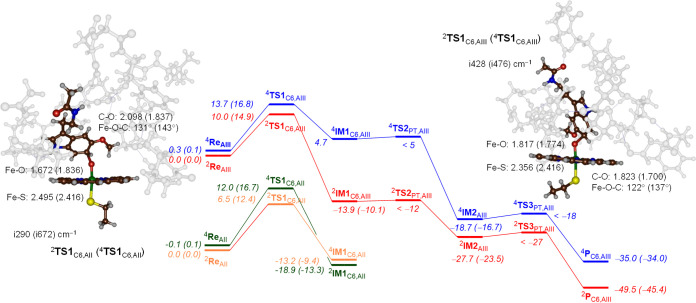
Reaction energy profile for aromatic hydroxylation of melatonin using a CpdI model of CYP1A1 with the substrate in binding orientation **II** and **III**. Energies represent UB3LYP/BS3//UB3LYP/BS1+ZPE + *E*_solv_ data in kcal mol^–1^, while free energies are shown in parenthesis. Optimized geometries of the rate-determining step give bond lengths in angstroms, bond angles in degrees, and the imaginary frequency in cm^–1^.

The alternative pathway to O-demethylation of melatonin by CYP1A enzymes is aromatic hydroxylation at the C^6^-position. This mechanism starts with an electrophilic attack of the oxo group on the C^6^ carbon atom of the substrate to form a σ-complex, ^4,2^**IM1**_C6_. The calculated potential energy landscape is given in Figure 6. The rate-determining step is the initial electrophilic attack via ^4,2^**TS1**_C6,AIII_ that leads to an intermediate **IM1**_C6,AIII_. The transition-state barriers have values of Δ*E* + ZPE = 10.0 (13.7) kcal mol^–1^ in the doublet (quartet) spin state. In contrast, the model **A**_**II**_ transition states give barriers of Δ*E* + ZPE = 6.5 (doublet) and 12.0 (quartet) kcal mol^–1^. This means that for model **A**_**II**_, the aromatic hydroxylation barriers are close in energy to the hydrogen atom abstraction barriers from the methoxy group, and as such, a mixture of products can be expected when the substrate binds in orientation **II** in the substrate-binding pocket. On the other hand, in substrate-bound orientation **III**, the aromatic hydroxylation barriers are considerably lower in energy than the methoxy hydrogen atom abstraction barriers by more than 7 kcal mol^–1^. Consequently, substrate-binding orientation **III** will give dominant aromatic hydroxylation products only and little O-demethylation. These energetic values of the transition states calculated with DFT, therefore, are opposite of what was concluded from the substrate-binding analysis from the MD simulations that predicted O-demethylation for models **II** and **III** and aromatic C^6^-hydroxylation only for model **III**. Clearly, the open substrate-binding pocket gives sufficient flexibility and mobility to the substrate to find the ideal orientation for aromatic hydroxylation. It should be noted that the aromatic hydroxylation transition state has lower imaginary frequencies (i290–i672 cm^–1^) than aliphatic hydrogen atom abstraction transition states (typically > i1000 cm^–1^), and consequently, the aromatic hydroxylation barrier is broad and wide, while the hydrogen atom abstraction barrier is narrow and tight. As such, hydrogen atom abstraction barriers often are highly sensitive to geometric constraints and changes, and the energies of the barriers vary between the two models by almost 20 kcal mol^–1^.

Consequently, the geometric orientation for hydrogen atom abstraction is vital for optimal hydrogen atom transfer, while the aromatic hydroxylation barrier is lesser sensitive to structural changes. From these radical intermediates, the *ipso*-proton is transferred to one of the nitrogen atoms of porphyrin. We were unable to locate the transition states for this process, but a geometry scan shows this step to be facile and leading to the proton-transfer intermediates **IM2** with large exothermicity. The conversion from **IM1** to **IM2** brings the aromaticity back into the aromatic ring and hence stabilizes the model strongly. In a final proton transfer, the proton is reshuttled back to the oxygen atom to form the phenol product. Also, the latter proton transfer (via **TS3**_PT,AIII_) has a negligible barrier. Therefore, the reaction past the C–O bond formation transition state will be fast and rapidly lead to the phenol product complexes. Previous aromatic hydroxylation studies confirm with the mechanism, as displayed in Figure 6, with a rate-determining C–O bond formation step.^120−127^

The two pairs of transition states (**TS1**_C6_) for model **A**_**II**_ and **A**_**III**_ structures are distinctly different. Thus, the N–H group of substrate melatonin forms a hydrogen bonding interaction with the carboxylate of Asp_313_ in both the model **A**_**II**_ and **A**_**III**_ structures. In the model **A**_**III**_ structure, the plane of the melatonin is perpendicular to the porphyrin plane with a dihedral angle of close to 180° for the Fe–O–C^6^–C^3^ dihedral, while it is only 130° for the model **A**_**II**_ structures. As such, the melatonin is in upright position in model **III** and twisted sideways for model **II**. Nevertheless, in all four transition-state structures for aromatic hydroxylation, the substrate forms a hydrogen bond with Asp_313_, and there is an interaction between the *ipso*-proton of the substrate with a nitrogen atom of the porphyrin ring. The transition states have an imaginary frequency of i290 (^2^**TS1**_C6,AII_), i672 (^4^**TS1**_C6,AII_), i428 (^2^**TS1**_C6,AIII_), and i476 (^4^**TS1**_C6,AIII_) cm^–1^ for the C–O stretch vibration. Geometrically, the transition states take place at a C–O distance of 1.700–2.098 Å, while the Fe–O distances span a range from 1.672 to 1.836 Å. The Fe–O–C angles in the transition states are very similar for the two doublet spin structures and for the two quartet spin structures, which means the overall orientation is not significantly different for the two complexes, and approach of the aromatic ring to CpdI leads to similar transition-state structures. After the C^6^–O activating transition-state **TS1**_C6,AIII_, the system rapidly leads to aromatic hydroxylation of the substrate with short-lived intermediates **IM1**_C6,AIII_ and **IM2**_C6,AIII_ that have negligible barriers for their subsequent steps. Indeed, we were unable to characterize transition-states **TS2**_PT,AIII_ and **TS3**_PT,AIII_ and estimate them within 1 kcal mol^–1^ of their precursor intermediate. Both steps lead to a large exothermicity and hence will be irreversible. Consequently, the first transition state via **TS1**_C6,AIII_ will be rate-determining for the aromatic hydroxylation pathway.

### Electronic Changes during the Reaction Mechanisms

**Figure 7:**
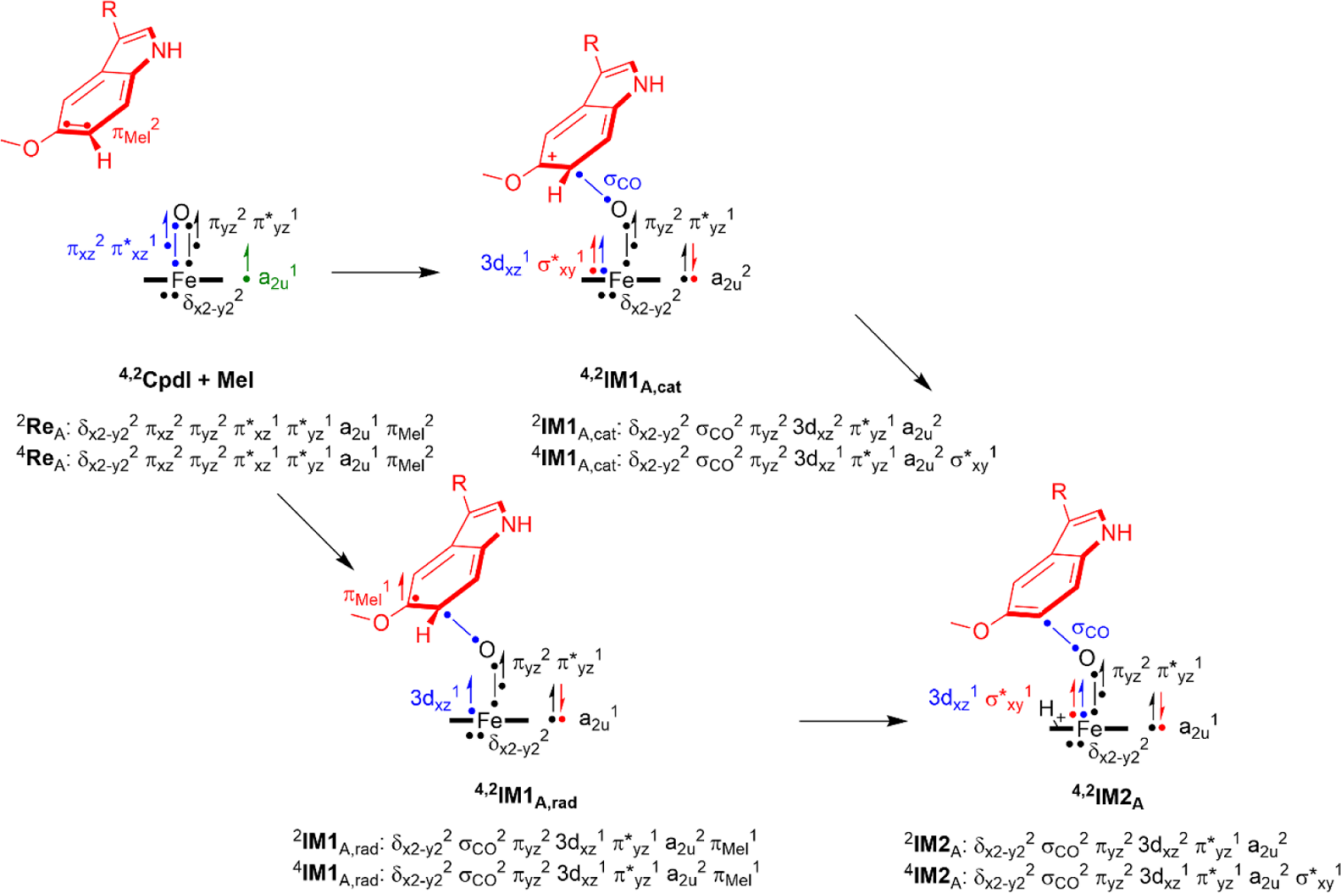
Electron distributions in the doublet and quartet spin states for reactants and intermediates along the aliphatic hydroxylation and aromatic hydroxylation pathways. Dots represent individual electrons with the arrows up-spin and down-spin assignments.

To understand the electron transfer processes and highlight how these are different for aromatic hydroxylation and aliphatic hydroxylation, we show an electron-transfer scheme in Figure 7. Thus, CpdI has the metal 3d-type orbitals occupied as δ_*x*^2^–*y*^2^_^2^ π_*xz*_^2^ π_*yz*_^2^ π*_xz_^1^ π*_yz_^1^, whereby the π and π* pairs of orbitals represent the antibonding interactions along the Fe–O bond. The δ_*x*^2^–*y*^2^_ orbital is a nonbonding orbital in the plane of the heme. In addition, CpdI has a radical on the heme in an orbital designated a_2u_ that mixes significantly with a lone pair orbital on the axial ligand. A comparison of the reactant and hydrogen atom abstraction structures shows that hydrogen atom abstraction leads to elongation of the Fe–O and shortening of the Fe–S distances. Thus, the hydrogen atom abstraction leads to an electron transfer into the metal FeO orbitals and fills π*_xz_ with a second electron, while the a_2u_ orbital remains singly occupied. As such, the transition-states ^4,2^**TS1**_HA,AIII_ and radical intermediates **IM1**_HA_ have a configuration π*_xz_^2^ π*_yz_^1^ a_2u_^1^ ϕ_Sub_^1^. The ϕ_Sub_ orbital represents the radical on the methoxy group, and its electron is up-spin in the quartet spin and doublet spin states. As a result, in ^2^**TS1**_HA,AIII_, the FeO group has a spin of 1.75, while it is 1.48 in the quartet spin state, and hence, these values are considerably less than those for the CpdI structures. After **IM1**_**HA**_, a second electron is transferred to form **P**_Hy_, resulting in a close-shell porphyrin group (doubly occupied a_2u_ orbital) and an iron(III) state with occupation π*_xz_^2^ π*_yz_^1^ in the doublet spin state and π*_xz_^1^ π*_yz_^1^ σ*_z^2^_^1^ in the quartet spin state. Overall, the optimized geometries match previous calculations of substrate hydroxylation reactions by P450 CpdI models well and find analogous distances, charge distributions, and structures.^89−115^

In principle, the step via ^4,2^**TS1**_C6_ can happen through O–C bond formation and the transfer of one electron from the substrate to CpdI, which retains a radical on the substrate moiety. Alternatively, two electrons are transferred from the substrate to oxidant during this step to create the [Fe^III^(Por)O–Sub] complex, where one electron has filled the heme a_2u_ orbital with a second electron and one electron has transferred into the metal 3d-system, namely, in π*_xz_ in the doublet spin state and in σ*_z^2^_ in the quartet spin state. Often these two pathways, that is, radical versus cationic, in aromatic hydroxylation mechanisms are close in energy with usually a preference for the cationic mechanism.^118−123^ Indeed, the group spin densities of ^4^**IM1**_C6,AIII_ and ^2^**IM1**_C6,AIII_ give dominant spin on the metal with values of 3.0 and 0.99, respectively, while little or no spin is found on the substrate moiety. Note that the σ*_z^2^_ orbital is built up from the 3d_*z*^2^_ orbital on iron that interacts with the 3p_*z*_ on sulfur and the 2p_*z*_ on oxygen, and hence give some spin on the sulfur atom in the quartet spin state structure as well (ρ_S_ = −0.21 in ^4^**IM1**_C6,AIII_).

### Selectivity Patterns

**Figure 8:**
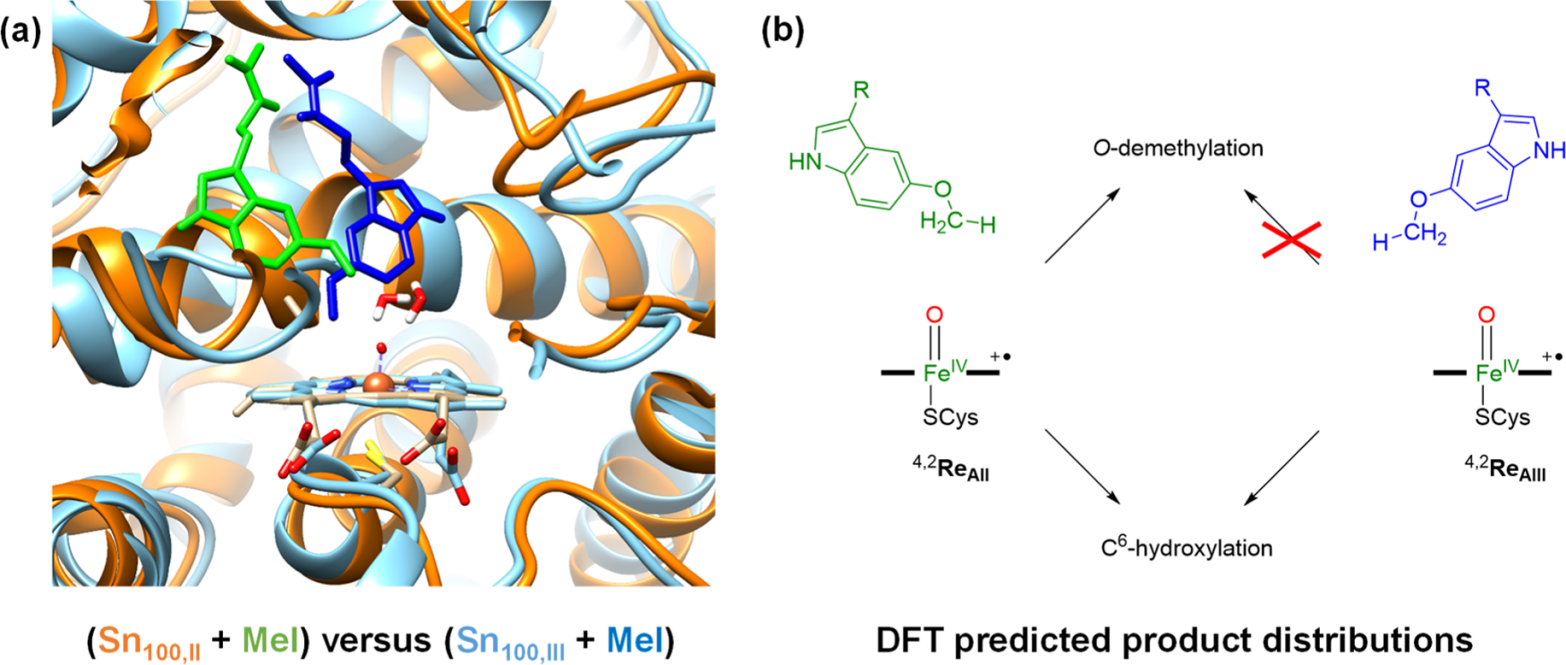
(a) Overlay of the 100 ns snapshots of the melatonin (Mel)-bound complexes as obtained from the MD simulation for models **II** (ribbons in amber and substrate in green) and **III** (ribbons in light blue and substrate in dark blue) from the MD snapshots (Sn) taken after 100 ns. (b) Product distributions as predicted from DFT cluster model calculations on ^4,2^**Re**_AII_ and ^4,2^**Re**_AIII_.

We then compared the overall reaction patterns and structures of the model **II** and **III** reaction pathways and show these in Figure 8. On the left-hand side, we show an overlay of the 100 ns snapshots obtained from the MD simulations for starting position **II** and **III** with the substrate and heme highlighted. As can be seen, the individual chains of the protein are in almost the same position in the structures, which shows that the protein is highly rigid in both cases. The substrate position and orientation are, of course, different for the two structures although both structures point the methoxy group into the heme-binding pocket. Cluster models were then created of these MD snapshots and transition states, and the local minima leading to products for methoxy and C^6^ hydroxylation were found. The aromatic hydroxylation initial transition states have low imaginary frequencies, and consequently, the peak is broad and wide, while the hydrogen atom abstraction transition state is narrow and tight due to the large imaginary frequency. As such, hydrogen atom abstraction barriers appear to be highly sensitive to geometric constraints and changes, and the energies of the barriers vary between the two models by almost 20 kcal mol^–1^. Consequently, the geometric orientation for hydrogen atom abstraction is vital for optimal hydrogen atom transfer, while the aromatic hydroxylation barrier is lesser sensitive to structural changes.

A comparison of the barrier heights for **TS1**_C6,AIII_ in Figure 6 with the methoxy group activation via **TS1**_HA,AIII_ in Figure 4 shows that aromatic hydroxylation has considerably lower barriers than methoxy group activation for model **A**_III_ by more than 7 kcal mol^–1^. Consequently, the dominant products for positioning the substrate in orientation **III** will be aromatic hydroxylation of melatonin at the C^6^-position, as shown in the summarizing scheme on the right-hand side of Figure 8. In contrast, the reaction pathways from ^4^**Re**_AII_ give much lower barriers for both hydrogen atom abstraction from the methoxy group and C–O activation of less than 7 kcal mol^–1^. These low-energy barriers implicate fast reaction processes for aromatic and aliphatic hydroxylation reactions for the substrate in binding orientation **II**. As such, a mixture of products is expected for substrate-binding orientation **II**. The two reactant conformations **Re**_AII_ and **Re**_AIII_, therefore, are expected to generate different product channels in a reaction between CpdI and substrate, whereby **Re**_AII_ gives dominant O-demethylation, while **Re**_AIII_ leads to dominant aromatic hydroxylation pathways. The relative population of these substrate-binding orientations will determine the product distributions. Experimental product distributions obtained by Ma et al.^33^ indeed predicted a mixture of products when melatonin is activated by CYP1A1 enzymes with 75% aromatic hydroxylation products and only 10% O-demethylation products.

Our binding energy studies implicate stronger binding of melatonin in position **III** than that in position **II**; hence, more products are expected from the reaction of **Re**_AIII_ than that of the **Re**_AII_ pathways, which is consistent with dominant aromatic hydroxylation products. Moreover, the two binding poses are predicted to give different reaction products; hence, the relative abundance of binding orientation **II** over **III** will determine the product distributions of C^6^-aromatic hydroxylation versus O-demethylation by CYP450 1A1 enzymes.

## Conclusions

In this work, a computational study is presented on metabolism of melatonin by CYP1A1 subfamily of enzymes. Our initial studies focused on substrate binding using MM and dynamics analysis. The MM and MD simulations highlight various low-energy substrate-binding modes and orientations in the substrate-binding pocket. However, these binding poses are highly rigid, and little movement of the protein and substrate is encountered in 100 ns MD simulations. Further tunneling analysis of the MD snapshots highlights a number of entrance and exit channels into the heme active site. However, several of those appear to be open during the main part of the MD simulation and may lead to substrate escape back into solution. Subsequently, two closed conformations of the substrate in the heme-binding pocket were selected, and large QM cluster models were created of 308 and 317 atoms that encapsulate the substrate environment and heme interactions. DFT studies were performed using the QM cluster models on two possible pathways including O-demethylation and aromatic hydroxylation. The work shows that substrate binding and positioning are important to guide the reaction. Thus, approach under “ideal” angles leads to low energy barriers, while a twisted approach of substrate to CpdI gives higher barriers. These studies highlight the effect of the second coordination sphere in proteins on kinetics and product selectivities and product distributions by CYP1A1 enzymes and explain experimental results.

## Supporting Information Available

Detailed analysis of the MD simulations, absolute and relative energies, group spin densities and charges, and a full set of Cartesian coordinates of all structures (PDF)

## Abbreviations

BDE: bond dissociation energy
CpdI: compound I
CpdII: compound II
CYP1A1: cytochrome P450 1A1
CYP1A2: cytochrome P450 1A2
DFT: density functional theory
MD: molecular dynamics
MM: molecular mechanics
P450: cytochrome P450
pdb: protein databank file
QM: quantum mechanics
QM/MM: quantum mechanics/molecular mechanics
rmsd: root-mean-square-deviation
ZPE: zero-point energy
