# Supplementary material for: Mechanism of Melatonin Metabolism by CYP1A1: What Determines the Bifurcation Pathways of Hydroxylation versus Deformylation?
Source: J Phys Chem B. 2022 Nov 16;126(46):9591–606. doi: 10.1021/acs.jpcb.2c07200 (PMC9706573; doi:10.1021/acs.jpcb.2c07200)
Supplement: Supplementary file 1 — jp2c07200_si_001.pdf [file jp2c07200_si_001.pdf]

Supporting Information

Mechanism of Melatonin Metabolism by CYP1A1. What Determines the Bifurcation Pathways of Hydroxylation Versus Deformylation?

Thirakorn Mokkawes,[§,‡] Ze Qing Lim,[§,‡] and Sam P. de Visser*[§,‡]

[§] Manchester Institute of Biotechnology, The University of Manchester, 131 Princess Street, Manchester M1 7DN, United Kingdom

[‡] Department of Chemical Engineering, The University of Manchester, Oxford Road, Manchester M13 9PL, United Kingdom

Email: sam.devisser@manchester.ac.uk

Methodology

Primary model preparation

CYP1A1 was selected as the main enzyme for this study. Currently, there were 3 PDB structures of CYP1A1 in the Protein Databank[1] which we analysed in detail. All three PDB structures are from human CYP1A1 origin and had different substrates bound. The chain A of the 6DWN[2] PDB structure was selected as it is the latest released structure and with good resolution. The pdb has erlotinib[3] in the active site and its structure is the closest to the metabolism of melatonin. Melatonin was taken from PDB ML1[4] and re-optimized using quantum mechanics methods in Gaussian-09[5] with neutral charge and singlet multiplicity. The optimised structure of melatonin was converted to a PDB file with the Antechamber[6] software package provided on AmberTools 2018[6]. The CYP1A1 structure was modified by removing erlotinib and the heme iron complex was changed into compound I by adding an oxygen atom over the central iron atom with bond length 1.686 Å and an water molecule was added nearby heme and I helix. All modifications were done on Chimera[7]. The optimised melatonin geometry was docked into the active site of the modified CYP1A1 structure by AutoDock Vina[8]. The docking resulted in 4 low-energy models whereby the orientation of the substrate was different. The grid boxes were adjusted manually which were generally placed into the centre of enzyme active site containing Compound I, I helix and G helix. The maximum energy difference between the best binding mode and the worst one was set to 2 kcal/mol. The maximum number of binding modes was set to 10. The four low-energy binding poses were selected and used for further MD studies.

Apart from structures of CYP1A1 with CpdI and substrate, we also created a product complex of methoxy-hydroxylated melatonin with an iron(III)-heme complex. Docking of methoxy-hydroxylated melatonin in the structure gave eight low-energy conformations that were subjected to MD simulations.

MD simulation

As the structure contains a metal atom in the Compound I state, the iron-ligand parameters had to be optimised for the forcefield that was used. This process made sure that the iron atom maintained 6 ligands, including 4 bonds to the heme, 1 ligand to the oxygen atom and 1 ligand to the axial cysteinate on the L helix. The MCPB.py[9] and Gaussian software packages were used to obtain the correct forcefield parameters for the ferryl core of the heme and nearby atoms in the porphyrin. Amber's Antechamber utility was applied to generate MD parameters for Compound I and melatonin substrate. Amber's tleap software package was applied to solvate the selected models in TIP3P[10] water and to generate the correct topology file to conduct MD simulations using the ff14SB[11] force field. For each complex, a rectangular box with 10 Å distance between the box edges and the protein was considered, resulting in around 14,600 water molecules for each system. Na^+ and Cl^- ions were added to neutralize the system. Minimization, heating, and MD productions were all performed with Amber's pmemd[12] utility. Minimization of each structure was carried out in only one step without restrains by setting steepest descent to 2000 cycles. Heating of each structure was heated up from 0 to 310 K by 100 ps. Finally, an MD production run was performed for 100 ns under constant temperature and pressure conditions at 310 K and 1 bar by running 20 times of 5 ns simulation.

QM model preparation

The calculated structures of model I to IV were analysed by python-based software packaged named Pytraj.py[13]. The model visualisation was presented by VMD[14]. The following values were extracted from the topologies and trajectories files:

Binding pose: binding poses were collected from the production of MD simulation in order to check the changes of the substrate orientation and residue interaction at 60 ns, 80 ns and 100 ns because after 50 ns all models had stable structures with the RMSDs.

RMSD: the Root Mean Square Deviation (RMSD) of the structural changes for each model from the MD runs were analysed and we made sure that the protein had equilibrated. RMSD values were also checked for specific residues within the protein as well as for the full system.

Bond length: bond lengths of the specific atoms were extracted in order to get the information for model selection for the QM calculations.

Hydrogen bonds: during the MD simulations from 0 ns to 100 ns, the substrate often was seen to interact with nearby residues. Hydrogen bonding interactions were extracted from the MD runs to establish if the melatonin was in a specific orientation and stayed rigid or not. Melatonin was treated as donor and acceptor. Based on these structures some hydrogen bonds were deemed important and were included in the QM active site cluster.

Overall structure visualisation: after the MD simulations a cluster model was created and studied in Gaussian-09 with density functional theory methods. The output files were visualized in Chemcraft[15].

QM calculations

The active site models of CYP1A1 were calculated in Gaussian09. The models with Compound I, which include Fe atom, were carried out by hybrid basis sets including:

- Basis set 1 (BS1): UB3LYP 6-31G* : C, H, O, N, and S // LANL2DZ+ECP: Fe
- Basis set 2 (BS2): UB3LYP 6-311+G* : C, H, O, N, and S // LANL2DZ+ECP: Fe
- Basis set 3 (BS3): UB3LYP 6-311+G* : C, H, O, N, and S // cc-pVTZ : Fe

The models with and without Fe atom were calculated in the doublet and quartet spin states with -2 charge, and singlet multiplicities with neutral charge respectively. We ran geometry optimizations for reactants, intermediates and products, as well as transition states. The optimised models were calculated with method BS1 and followed by a single point calculation at level BS2, solvation. The scanning calculation was performed by BS1 on quintet multiplicities. The frequency calculation was carried at temperature = 298.15 K (25°C).

Data analysis

The deep analysation was carried by Python[16] and R[17] based scripts on a Jupyter-notebook[18] and R studio[19] respectively. In addition, the presented data was extracted by data-mining python software. The tunnels occurring the MD simulation were

analysed by the CAVER[20]. The tunnels were reported with their lengths and widths in angstroms.

References

1. Berman, H. M.; Westbrook, J.; Feng, Z.; Gilliland, G.; Bhat, T. N.; Weissig, H.; Shindyalov, I. N.; Bourne, P. E. The Protein Data Bank. *Nucl. Acids Res.* **2000**, *28*, 235-242.
2. Bart, A. G.; Scott, E. E. Structures of human cytochrome P450 1A1 with bergamottin and erlotinib reveal active-site modifications for binding of diverse ligands. *J. Biol. Chem*. **2018**, *293*, 19201-19210.
3. Kim, Y.; Lee, S.-H.; Ahn, J. S.; Ahn, M.-J.; Park, K.; Sun, J.-M. Efficacy and Safety of Afatinib for EGFR-mutant Non-small Cell Lung Cancer, Compared with Gefitinib or Erlotinib, *Cancer Res. Treat.* **2019**, *51*, 502-509,.
4. Wang. L.; Erlandsen, H.; Haavik, J.; Knappskog, P. M.; Stevens, R. C. Three-dimensional structure of human tryptophan hydroxylase and its implications for the biosynthesis of the neurotransmitters serotonin and melatonin. Biochemistry. **2002**,*41*,12569-12574.
5. Wang, J.; Wang, W.; Kollman, P. A.; Case, D. A. ANTECHAMBER: an accessory software package for molecular mechanical calculations. *J. Chem. Inform. Comput. Sci.* **2000**, *222*, 11-30.
6. Case, D. A.; Ben-Shalom, I. Y.; Brozell, S. R.; Cerutti, D. S.; Cheatham, III, T. E.; Cruzeiro, V. W. D.; Darden, T. A.; Duke, R. E.; Ghoreishi, D.; Gilson, M. K.; Gohlke, H.; Goetz, A. W.; Greene, D.; Harris, R.; Homeyer, N.; Huang, Y.; Izadi, S.; Kovalenko, A.; Kurtzman, T.; Lee, T. S.; LeGrand, S.; Li, P.; Lin, C.; Liu, J.; Luchko, T.; Luo, R.; Mermelstein, D. J.; Merz, K. M.; Miao, Y.; Monard, G.; Nguyen, C.; Nguyen, H.; Omelyan, I.; Onufriev, A.; Pan, F.; Qi, R.; Roe, D. R.; Roitberg, A.; Sagui, C.; Schott-Verdugo, S.; Shen, J.; Simmerling, C. L.; Smith, J.; Salomon-Ferrer, R.; Swails, J.; Walker, R. C.; Wang, J.; Wei, H.; Wolf, R. M.; Wu, X.; Xiao, L.; York, D. M.; Kollman, P. A. AMBER-2018, University of California, San Francisco, 2018.
7. Pettersen, E. F.; Goddard, T. D.; Huang, C. C.; Couch, G. S.; Greenblatt, D. M.; Meng, E. C.; Ferrin, T. E. UCSF Chimera - A Visualization System for Exploratory Research and Analysis. *J. Comput. Chem*. **2004**, *25*, 1605-1612.
8. Trott, O.; Olson, A. J. AutoDock Vina: improving the speed and accuracy of docking with a new scoring function, efficient optimization and multithreading, *J. Comput. Chem*. **2010**, *31*, 455-461.
9. Li, P.; Merz, K. M. MCPB.py: A Python Based Metal Center Parameter Builder. *J. Chem. Inf. Model*. **2016**, *56*, 599–604.
10. Jorgensen, W. L.; Chandrasekhar, J.; Madura, J. D.; Impey, R. W.; Klein, M. L. Comparison of simple potential functions for simulating liquid water. *J. Chem. Phys.* **1983**, *79*, 926-935.
11. Maier, J. A.; Martinez, C.; Kasavajhala, K.; Wickstrom, L.; Hauser, K. E.; Simmerling, C. ff14SB: Improving the accuracy of protein side chain and backbone parameters from ff99SB. *J. Chem. Theory Comput*. **2015**, *11*, 3696-3713.
12. Andreas W. Götz, Mark J. Williamson, Dong Xu, Duncan Poole, Scott Le Grand, and Ross C. Walker
13. Journal of Chemical Theory and Computation. **2012**, *8*, 1542-1555.
14. *Pytraj*. **2015**, Available: https://github.com/Amber-MD/pytraj .
15. Humphrey, W.; Dalke, A.; Schulten, K. VMD: Visual molecular dynamics. *J. Molec. Graph.* **1996**, *14*, 33-38.
16. *ChemCraft 1.8*. **2021**, Available: http://www.chemcraftprog.com/index.html .
17. *Python reference manual. Centrum voor Wiskunde en Informatica Amsterdam.* **1995**, Available: https://www.python.org .
18. Team, R. C. A language and environment for statistical computing. Foundation for Statistical Computing, Vienna, Austria., **202**0. Available: https://www.R-project.org/ .
19. *Jupyter Notebooks – a publishing format for reproducible computational workflows.* In Positioning and Power in Academic Publishing: Players, Agents and Agendas. Loizides, F.; Schmidt, B. (Eds.); **2016**, pp. 87–90, Available: https://jupyter.org .
20. *RStudio: Integrated Development for R. RStudio, Inc., Boston, MA*. **2019**. Available: http://www.rstudio.com/ .
21. Chovancová, E.; Pavelka, A.; Beneš, P.; Strnad, O.; Brezovský, J.; Kozlíková, B.; Gora, A.; Šustr, V.; Klvaňa, M.; Medek, P.; Biedermannová, L.; Sochor, J.; Damborský, J. CAVER 3.0: A Tool for the Analysis of Transport Pathways in Dynamic Protein Structures, *PLoS Comput. Biol*. **2012**, *8*, e1002708.

Primary model preparation

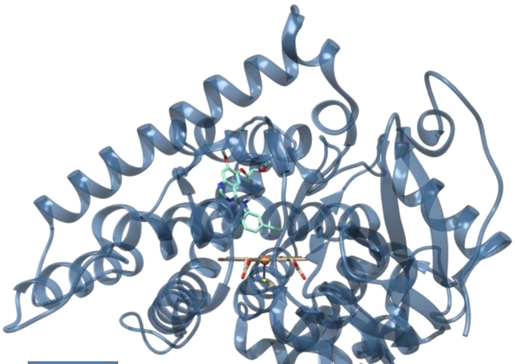

Chain A

MAKKTSSKGLKNPPGPWGWPLIGHMLTLGKNPHLA
LSRMSQQYGDVLQIRIGSTPVVVLSGLDTIRQALVR
QGDDFKGRPDLYTFTLISNGQSMSFSPDSGPVWAA
RRRLAQNGLKSFSIASDPASSTSCYLEEHVSKEAEV
LISTLQELMAGPGHFNPYRYVVVSVTNVICAICFGR
RYDHNHQELLSLVNLNNNFGEVVGSGNPADFIPILR
YLPNPSLNAFKDLNEKFYSFMQKMVKEHYKTFEKG
HIRDITDSLIEHCQEKQLDENANVQLSDEKIINIVLDL
FGAGFDTVTTAISWSLMYLVMNPRVQRKIQEELDTV
IGRSRRPRLSDRSHLPYMEAFILETFRHSSFVPFTIP
HSTTRDTSLKGFYIPKGRCVFVNQWQINHDQKLWV
NPSEFLPERFLTPDGAIDKVLSEKVIIFGMGKRKCIG
ETIARWEVFLFLAILLQRVEFSVPLGVKVDMTPIYGL
TMKHACCEHFQMQLRSHHHHHH

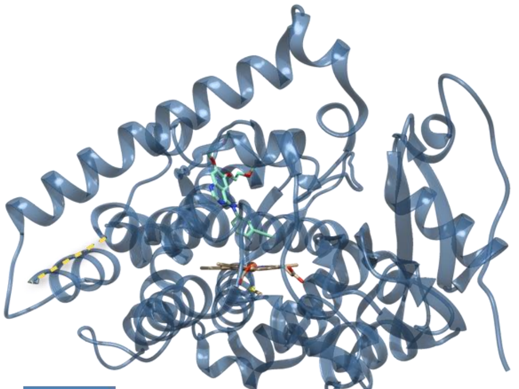

Chain B

MAKKTSSKGLKNPPGPWGWPLIGHMLTLGKNPHLA
LSRMSQQYGDVLQIRIGSTPVVVLSGLDTIRQALVR
QGDDFKGRPDLYTFTLISNGQSMSFSPDSGPVWAA
RRRLAQNGLKSFSIASDPASSTSCYLEEHVSKEAEV
LISTLQELMAGPGHFNPYRYVVVSVTNVICAICFGR
RYDHNHQELLSLVNLNNNFGEVVGSGNPADFIPILR
YLPNPSLNAFKDLNEKFYSFMQKMVKEHYKTFEKG
HIRDITDSLIEHCQEKQLDENANVQLSDEKIINIVLDL
FGAGFDTVTTAISWSLMYLVMNPRVQRKIQEELDTV
IGRSRRPRLSDRSHLPYMEAFILETFRHSSFVPFTIP
HSTTRDTSLKGFYIPKGRCVFVNQWQINHDQKLWV
NPSEFLPERFLTPDGAIDKVLSEKVIIFGMGKRKCIG
ETIARWEVFLFLAILLQRVEFSVPLGVKVDMTPIYGL
TMKHACCEHFQMQLRSHHHHHH

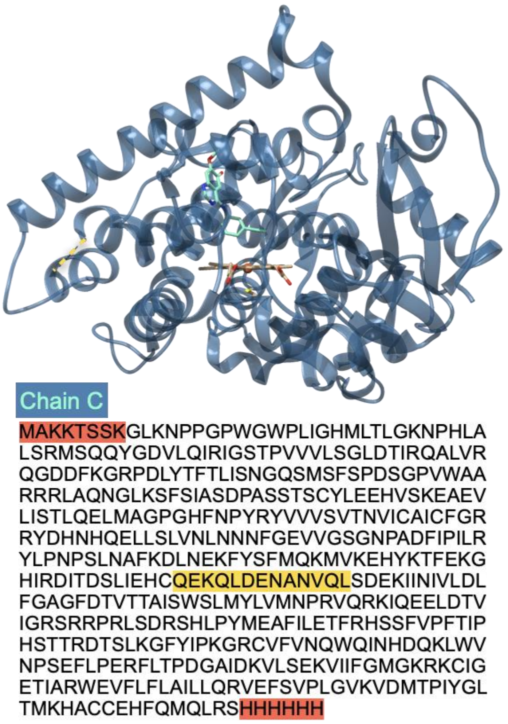

Chain C

MAKKTSSKGLKNPPGPWGWPLIGHMLTLGKNPHLA
LSRMSQQYGDVLQIRIGSTPVVVLSGLDTIRQALVR
QGDDFKGRPDLYTFTLISNGQSMSFSPDSGPVWAA
RRRLAQNGLKSFSIASDPASSTSCYLEEHVSKEAEV
LISTLQELMAGPGHFNPYRYVVVSVTNVICAICFGR
RYDHNHQELLSLVNLNNNFGEVVGSGNPADFIPILR
YLPNPSLNAFKDLNEKFYSFMQKMVKEHYKTFEKG
HIRDITDSLIEHCQEKQLDENANVQLSDEKIINIVLDL
FGAGFDTVTTAISWSLMYLVMNPRVQRKIQEELDTV
IGRSRRPRLSDRSHLPYMEAFILETFRHSSFVPFTIP
HSTTRDTSLKGFYIPKGRCVFVNQWQINHDQKLWV
NPSEFLPERFLTPDGAIDKVLSEKVIIFGMGKRKCIG
ETIARWEVFLFLAILLQRVEFSVPLGVKVDMTPIYGL
TMKHACCEHFQMQLRSHHHHHH

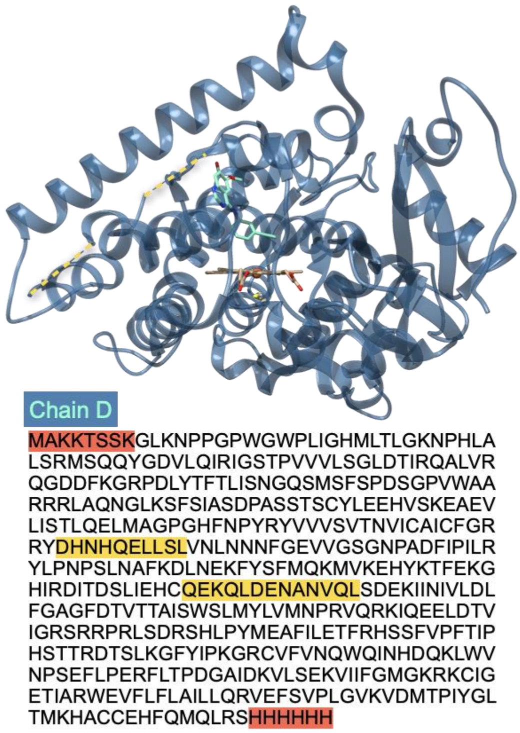

Chain D

MAKKTSSKGLKNPPGPWGWPLIGHMLTLGKNPHLA
LSRMSQQYGDVLQIRIGSTPVVVLSGLDTIRQALVR
QGDDFKGRPDLYTFTLISNGQSMSFSPDSGPVWAA
RRRLAQNGLKSFSIASDPASSTSCYLEEHVSKEAEV
LISTLQELMAGPGHFNPYRYVVVSVTNVICAICFGR
RYDHNHQELLSLVNLNNNFGEVVGSGNPADFIPILR
YLPNPSLNAFKDLNEKFYSFMQKMVKEHYKTFEKG
HIRDITDSLIEHCQEKQLDENANVQLSDEKIINIVLDL
FGAGFDTVTTAISWSLMYLVMNPRVQRKIQEELDTV
IGRSRRPRLSDRSHLPYMEAFILETFRHSSFVPFTIP
HSTTRDTSLKGFYIPKGRCVFVNQWQINHDQKLWV
NPSEFLPERFLTPDGAIDKVLSEKVIIFGMGKRKCIG
ETIARWEVFLFLAILLQRVEFSVPLGVKVDMTPIYGL
TMKHACCEHFQMQLRSHHHHHH

Figure S1. Extracts of the 6DWN PDB file. There are chain A to D. The chain A was used in this work by removing the existing substrate, Erlotinib. The residue sequences of chain A, B, C and D of 6DWN enzyme. Each chain has 512 residues. The missing residues in the middle and terminus are highlighted in yellow and red respectively.

MD simulation results.

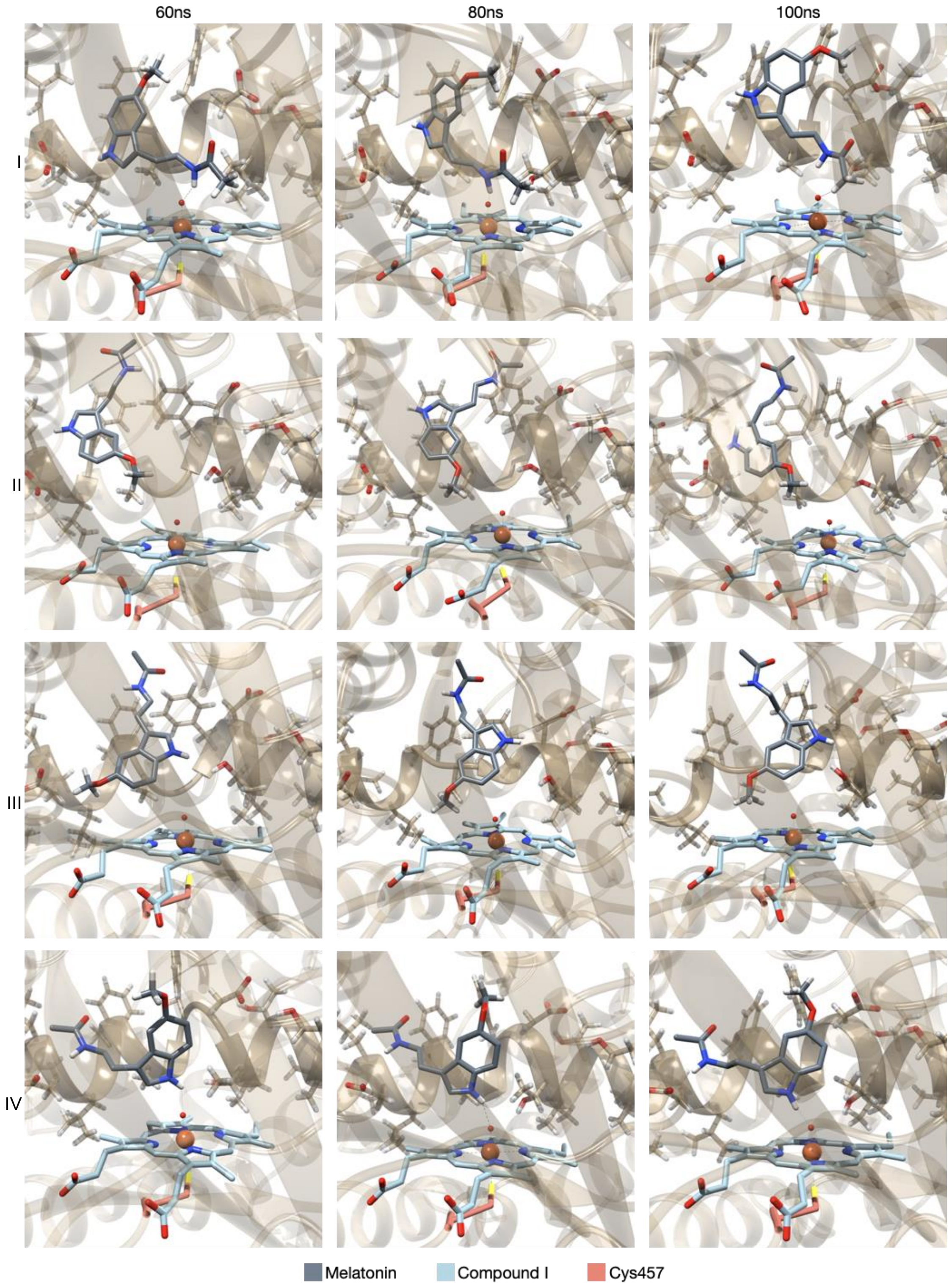

Figure S2. Selected binding poses of substrate versus CpdI as obtained from snapshots after 60, 80 or 100 ns from the MD simulations for CYP1A1 with melatonin with substrate starting orientation **I**, **II**, **III** and **IV**. Each MD was calculated from AutoDock Vina. The docking scores for each model were -9.4, -8.5, -6.5 and -7.0.

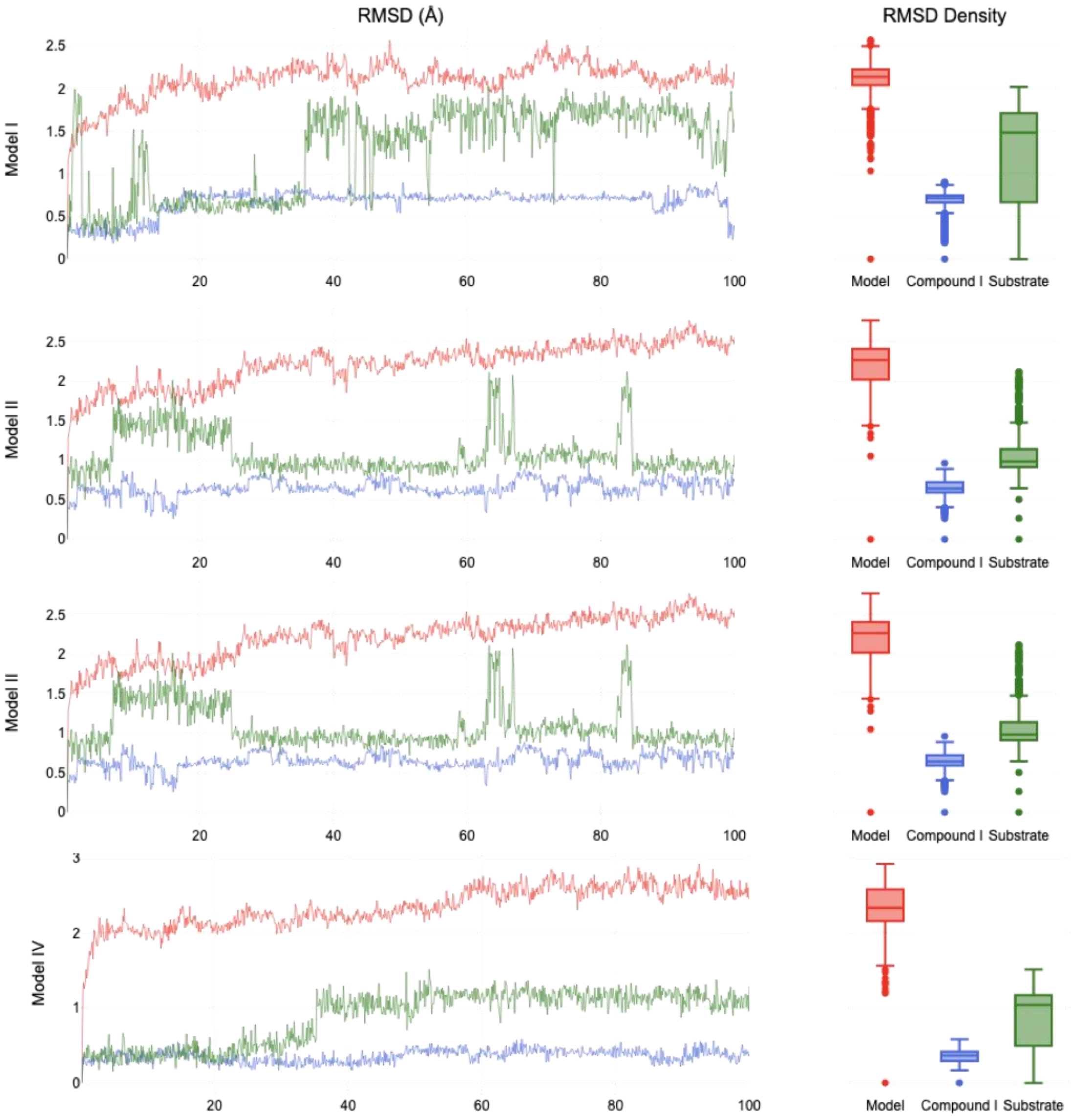

Figure S3. RMSD plots obtained from the MD simulations for CYP1A1 with melatonin with substrate starting orientation **I**, **II**, **III** and **IV** during a 100 ns MD calculation. The right-hand-side shows the individual RMSD values of the whole model (in red), Compound I only (in blue) and substrate only (in green).

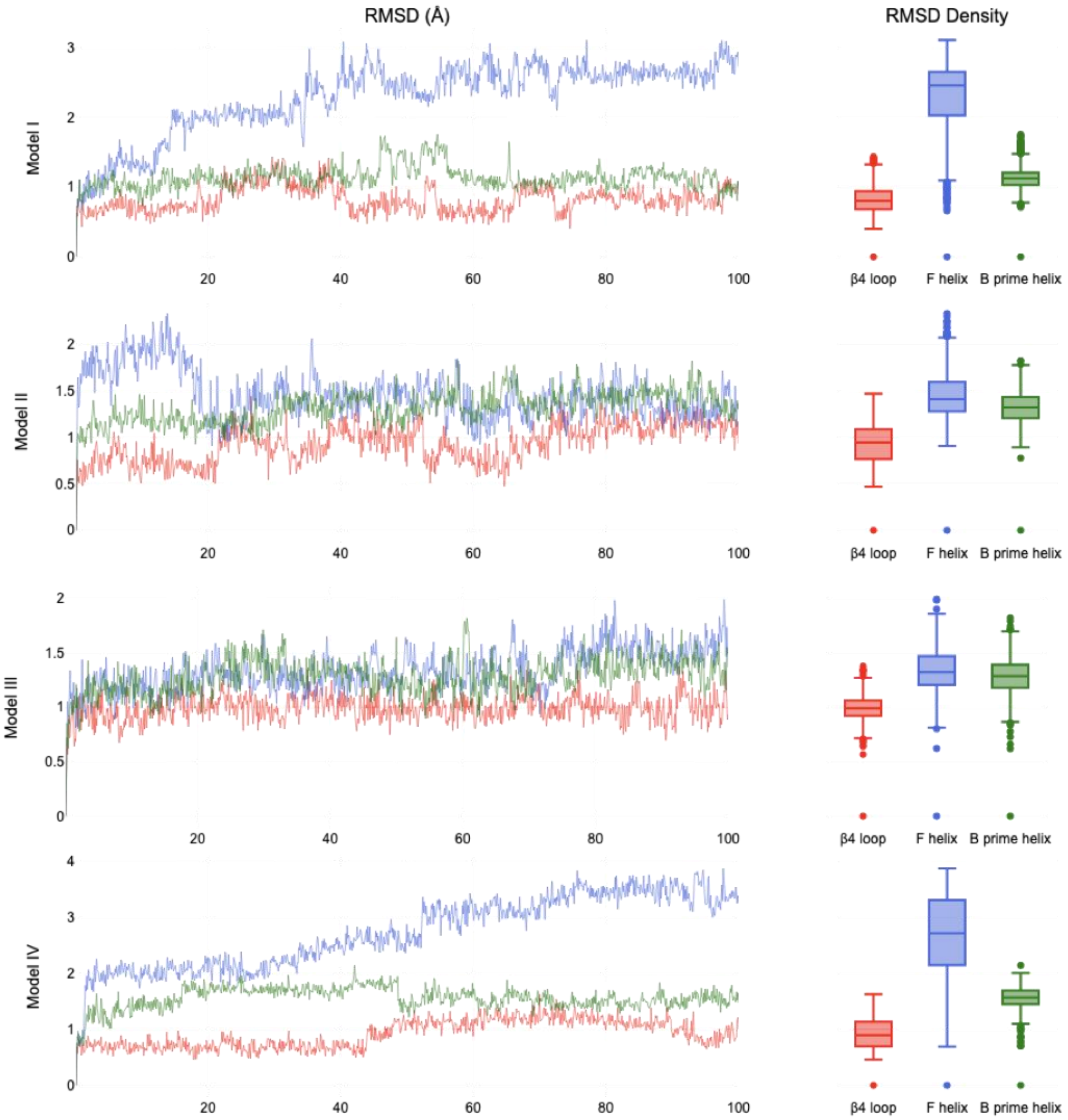

Figure S4. RMSD plots obtained from the MD simulations for CYP1A1 with melatonin with substrate starting orientation **I**, **II**, **III** and **IV** during a 100 ns MD calculation focusing on the change of the protein helixes. The right-hand-side shows the individual RMSD values of the β4 loop (in red), the F-helix (in blue) and the B' helix (in green).

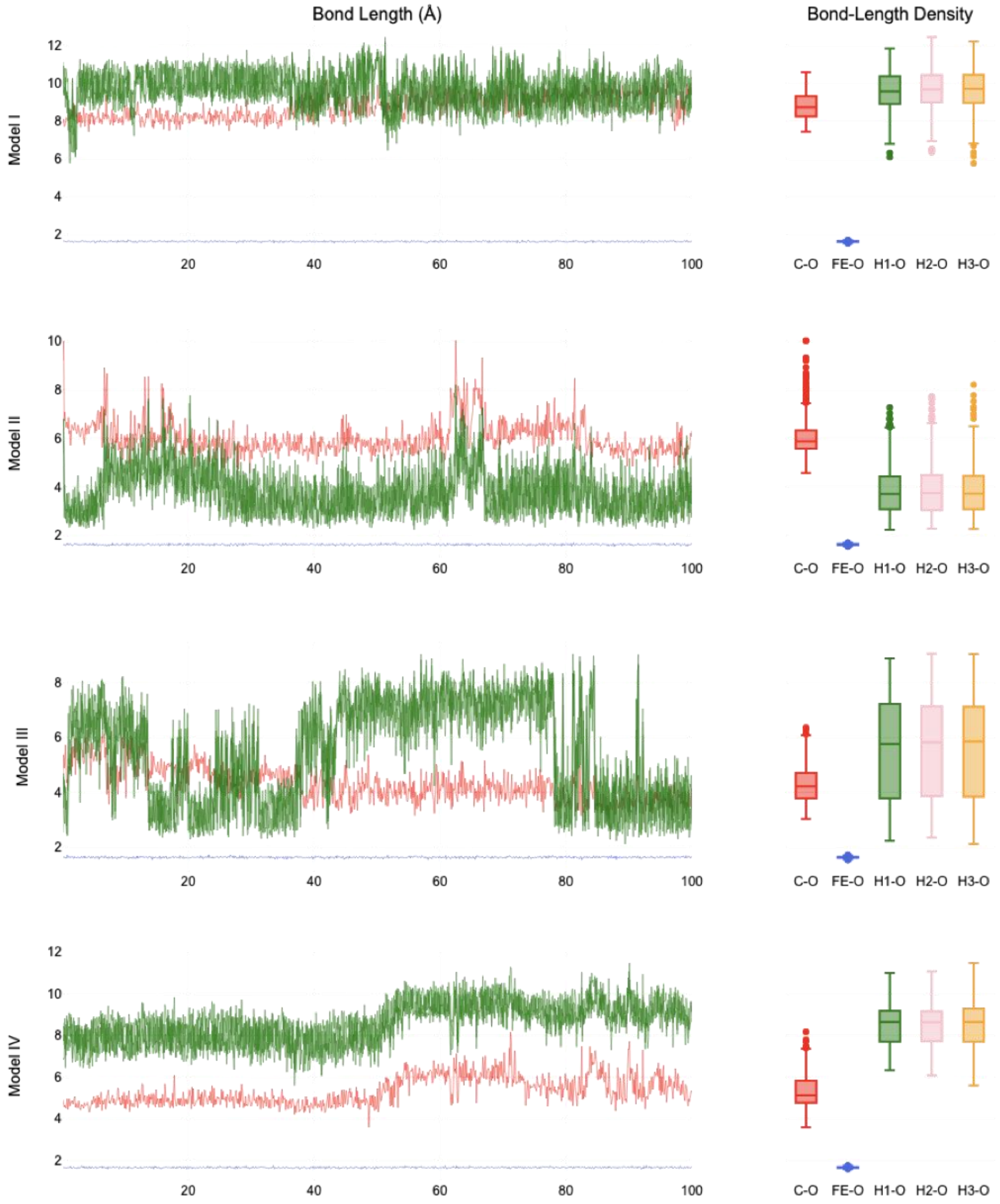

Figure S5. Bond length changes of key interactions obtained from the MD simulations for CYP1A1 with melatonin with substrate starting orientation **I**, **II**, **III** and **IV** during a 100 ns MD calculation. The right-hand-side shows the individual bond lengths for the C^6-O distance (in red), Fe-O distance (in blue), and the three methoxy H-O distances (in green, pink and yellow).

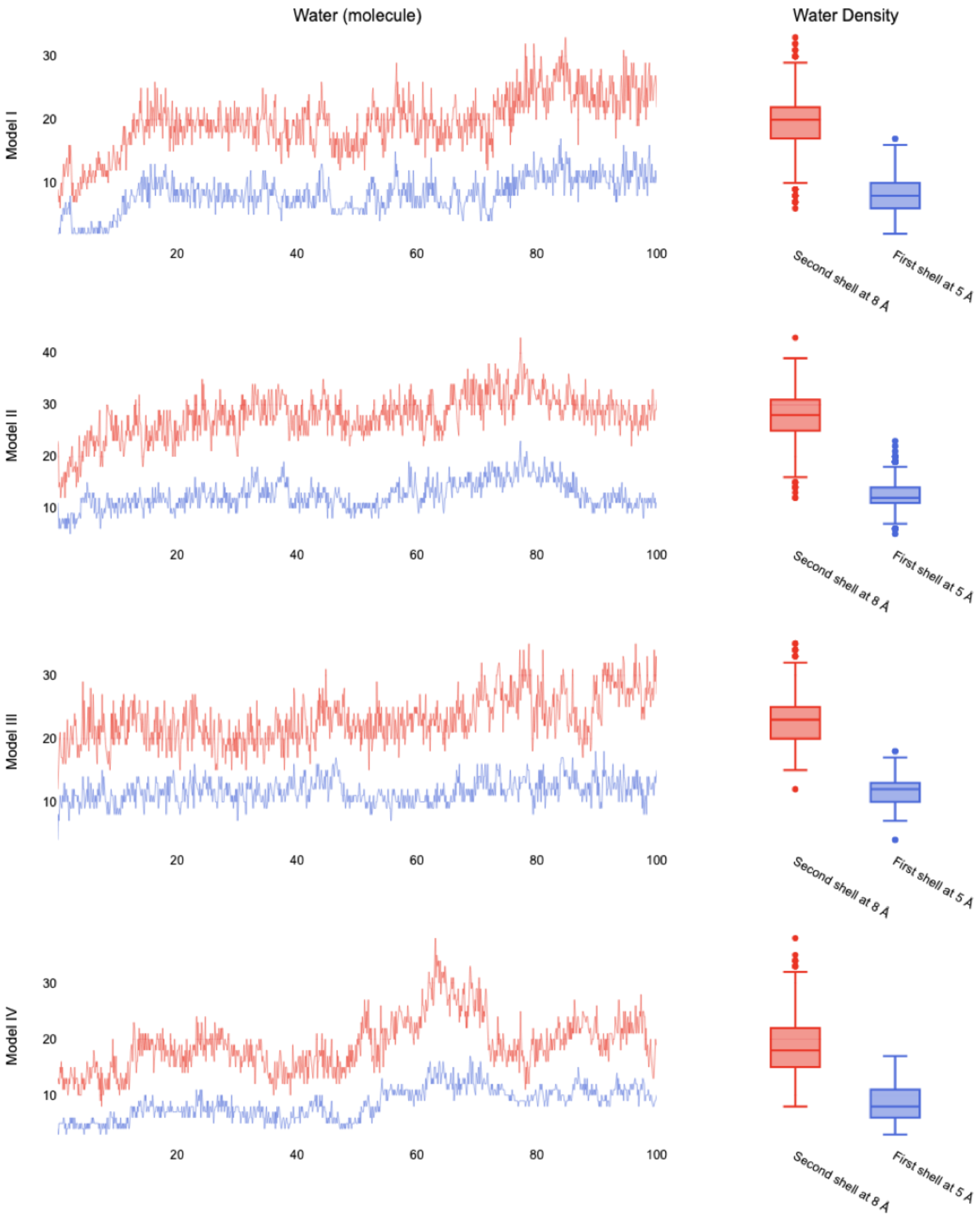

Figure S6. Protein solvation analysis obtained from the MD simulations for CYP1A1 with melatonin with substrate starting orientation **I**, **II**, **III** and **IV** during a 100 ns MD calculation focusing on the change of the protein helixes. The right-hand-side counts the number of water molecules within a radius of CpdI of 5 Å (in blue) and within 8 Å (in red).

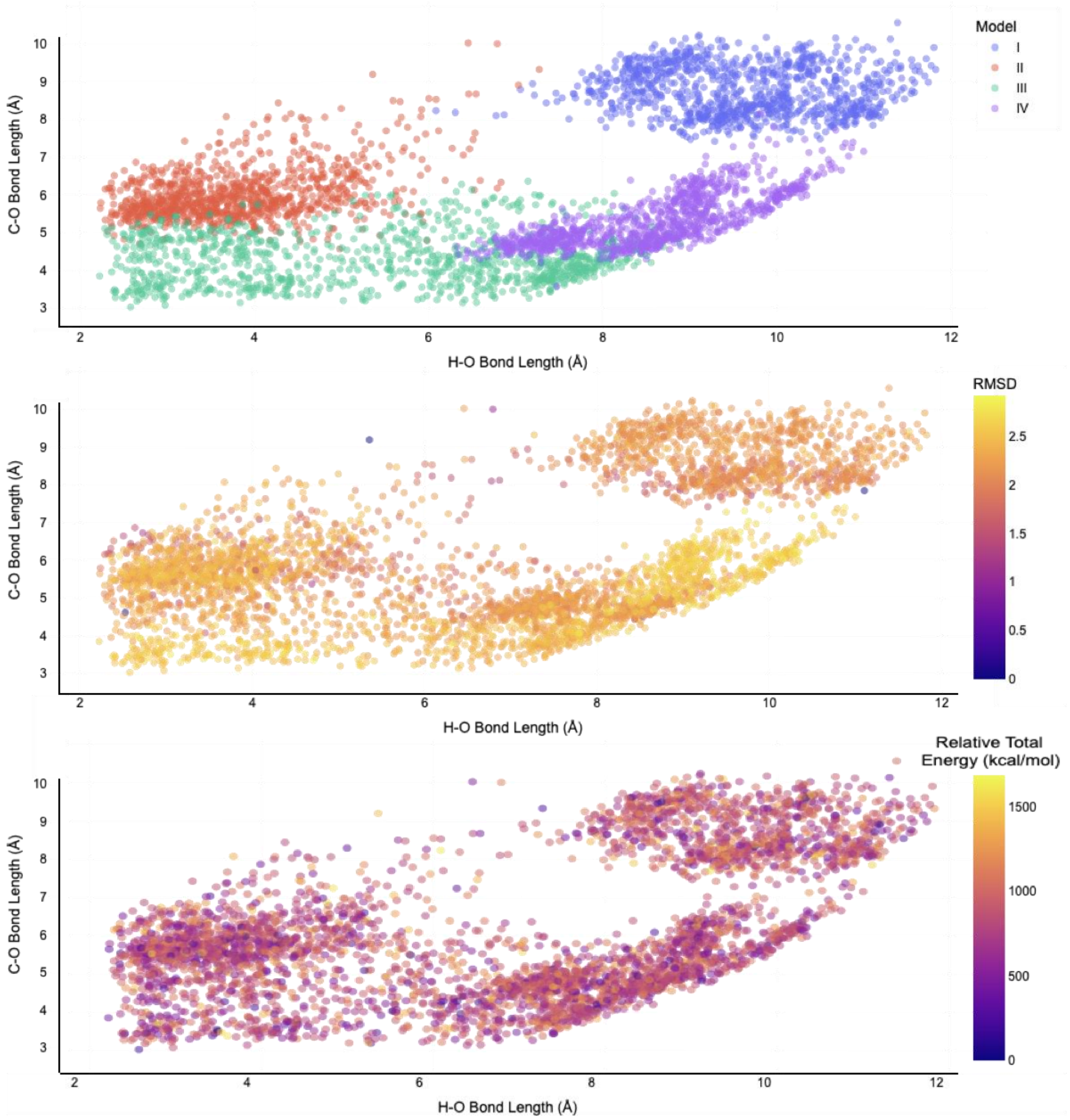

Figure S7. Analysis of position and orientation of the substrate with respect to CpdI from the MD simulations on CYP1A1 with melatonin with substrate starting orientation **I**, **II**, **III** and **IV** during a 100 ns. (a) Distance distribution from MD results of reactant models. The x-axis is the bond length between the hydrogen atom of the methoxy side chain of melatonin and the oxygen atom of Compound I, while the y-axis is the bond length between the carbon atom (C^6) of melatonin and the oxygen atom of Compound I. (b) RMSD value for individual snapshots. (c) Total energy of individual snapshots.

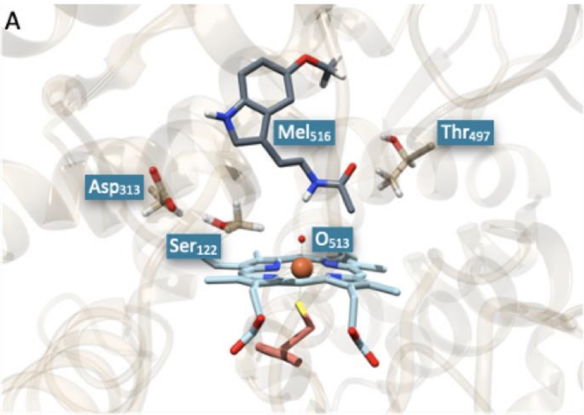

Donor residue	Donor atom	Acceptor residue	Acceptor atom	Lifetime
122	OG	516	H10	612
313	O	516	H10	64
313	OD2	516	H10	1
313	OD1	516	H10	19
513	O	516	H17	1
516	O15	497	HG1	1

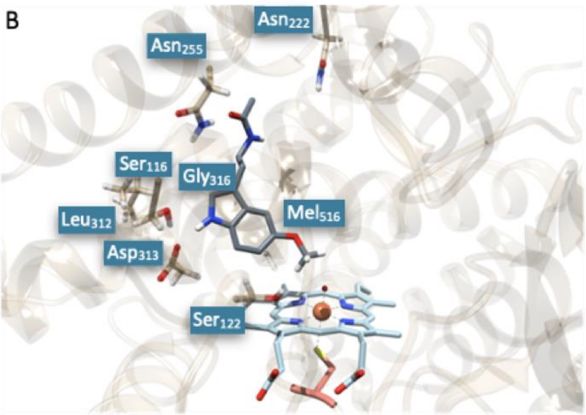

Donor residue	Donor atom	Acceptor residue	Acceptor atom	Lifetime
116	OG	516	H10	492
312	O	516	H10	8
313	OD1	516	H10	5
313	OD2	516	H10	7
316	O	516	H17	371
516	O15	222	HD22	1
516	O18	122	HG	3
516	O15	255	HD22	1

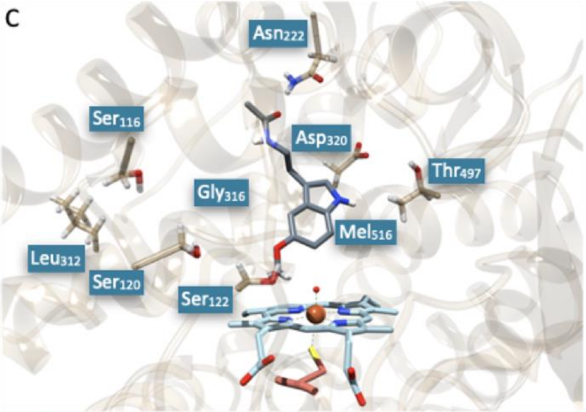

Donor residue	Donor atom	Acceptor residue	Acceptor atom	Lifetime
312	O	516	H17	34
316	O	516	H17	6
320	OD2	516	H10	7
497	OG1	516	H10	2
516	O15	116	H	1
516	O18	122	HG	1
516	O15	222	HD22	1
516	O18	120	HG	3

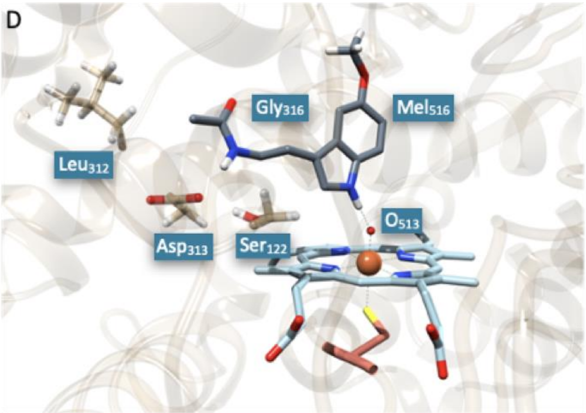

Donor residue	Donor atom	Acceptor residue	Acceptor atom	Lifetime
122	OG	516	H13	126
312	O	516	H13	10
313	OD1	516	H13	6
313	OD2	516	H13	69
316	O	516	H13	7
515	O1	516	H9	1

Melatonin Compound I Cys457

Figure S8. Key hydrogen bonding interactions of the substrate in the substrate-binding pocket of CYP1A1 as listed for model **I** (part A), **II** (part B), **III** (part C), and **IV** (part D). Data obtained from the MD simulations. Lifetime is the occurrence of each hydrogen bonds presented in frame from 1000 frames simulated by 100 ns.

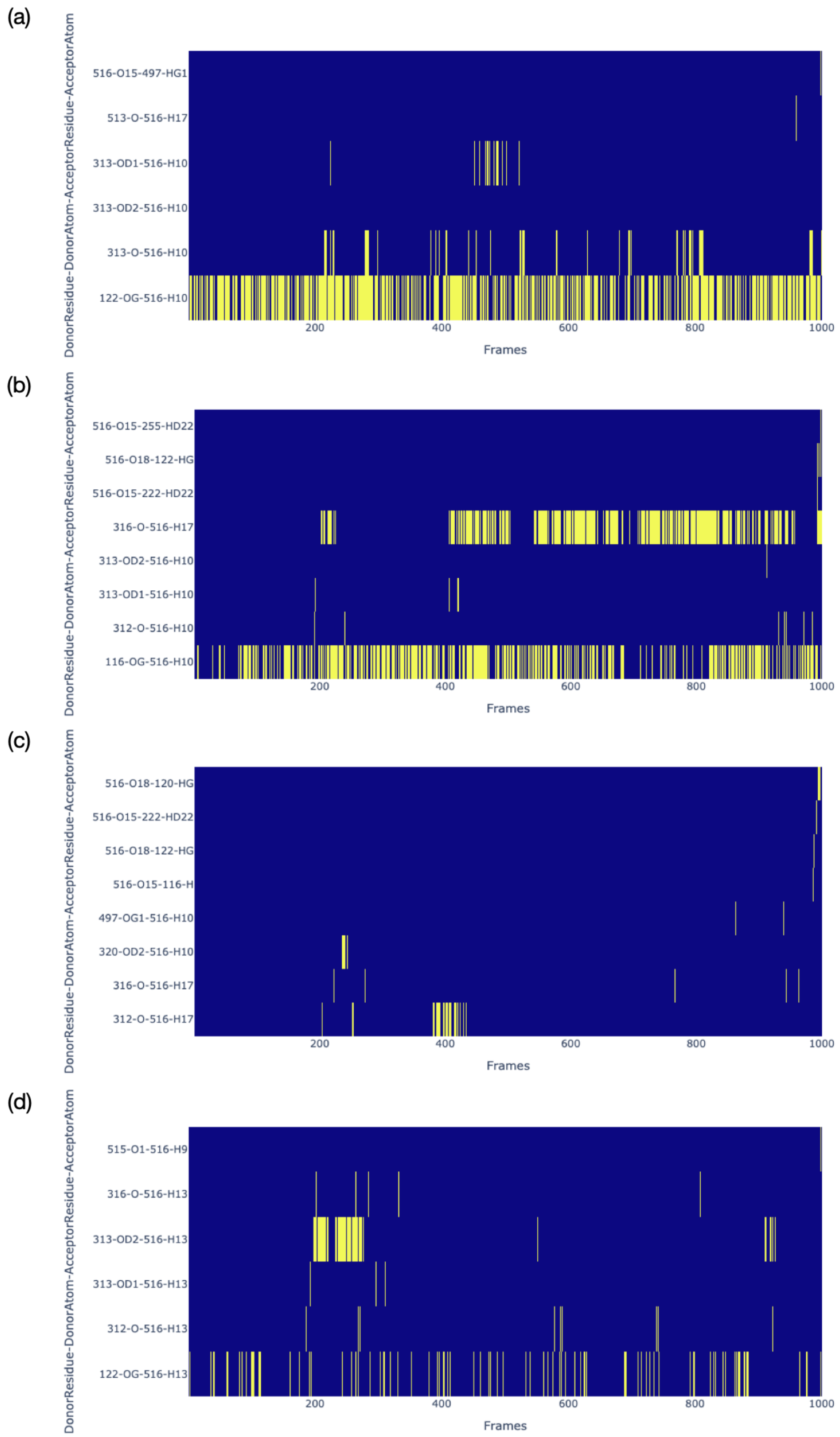

Figure S9. Selected hydrogen bonds and their lifetime during the MD simulations. Yellow and blue mean appear and disappear. The hydrogen bonds are also listed in Fig S8.

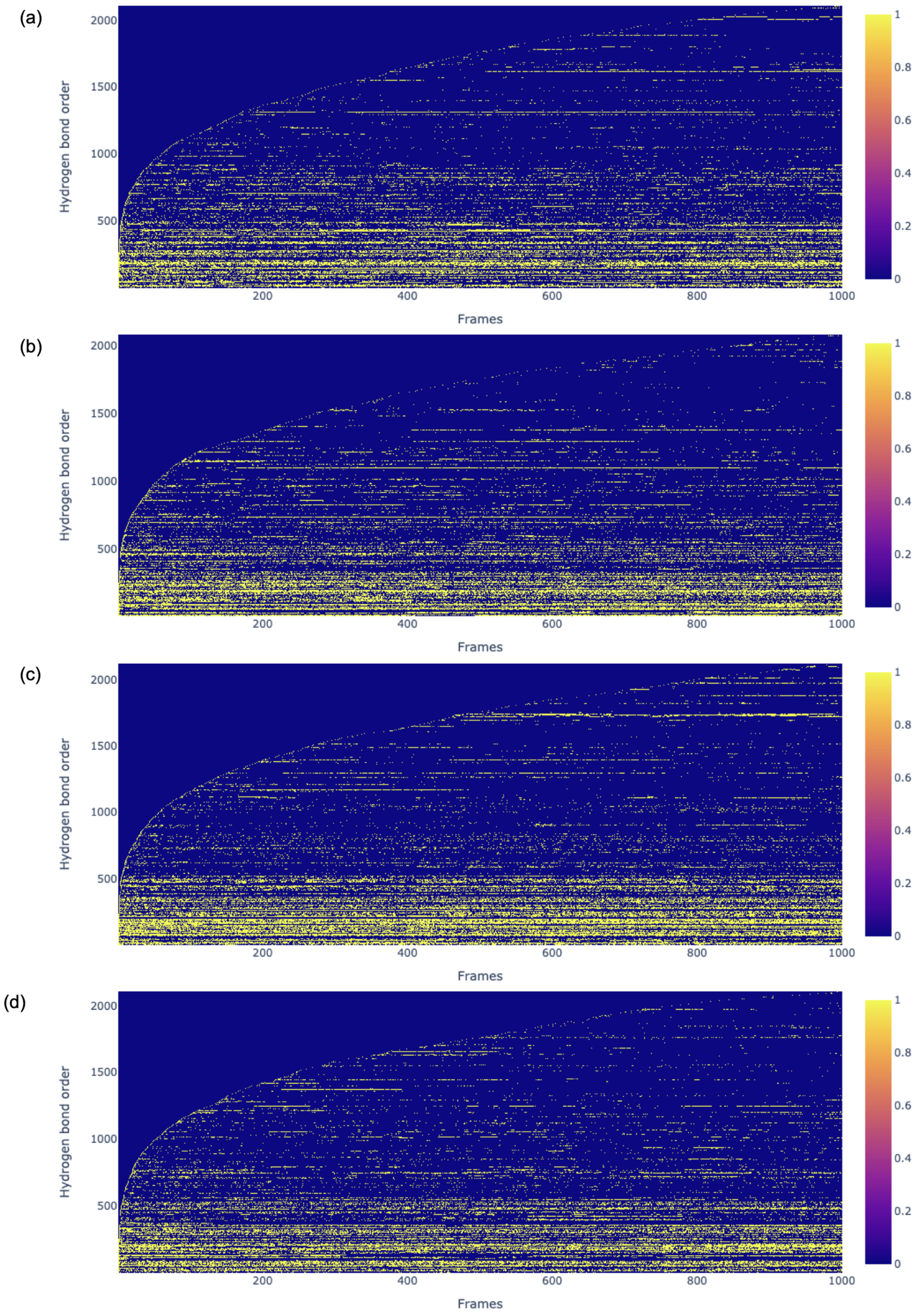

Figure S10. Hydrogen bonds and their lifetime during the MD simulations. Yellow and blue mean appear and disappear. The list of hydrogen bonds are presented at the end of this SI.

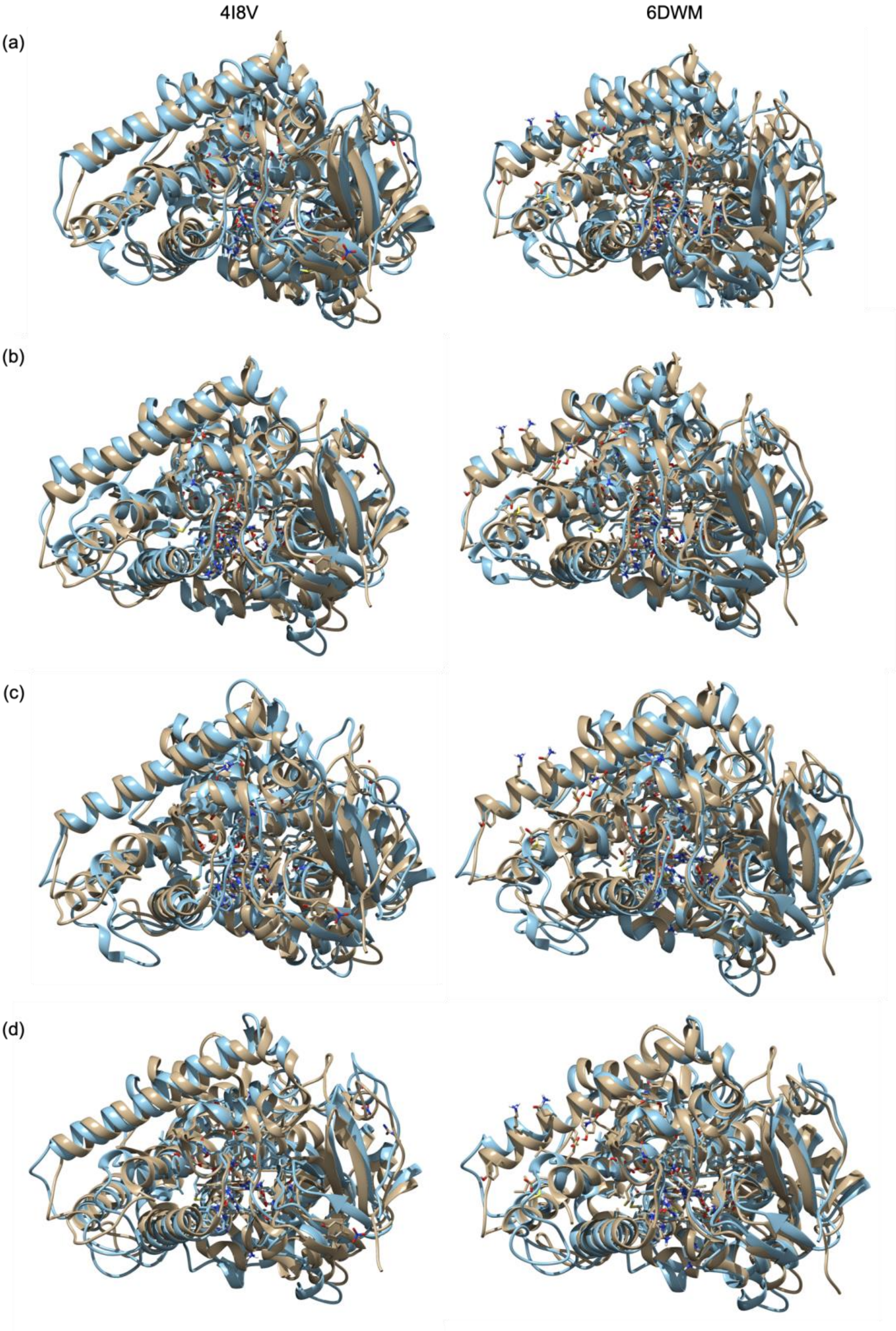

Figure S11. Overlay of the pdb from 4 model calculatd by MD and the CYP1A1 pdbs of previous studies.

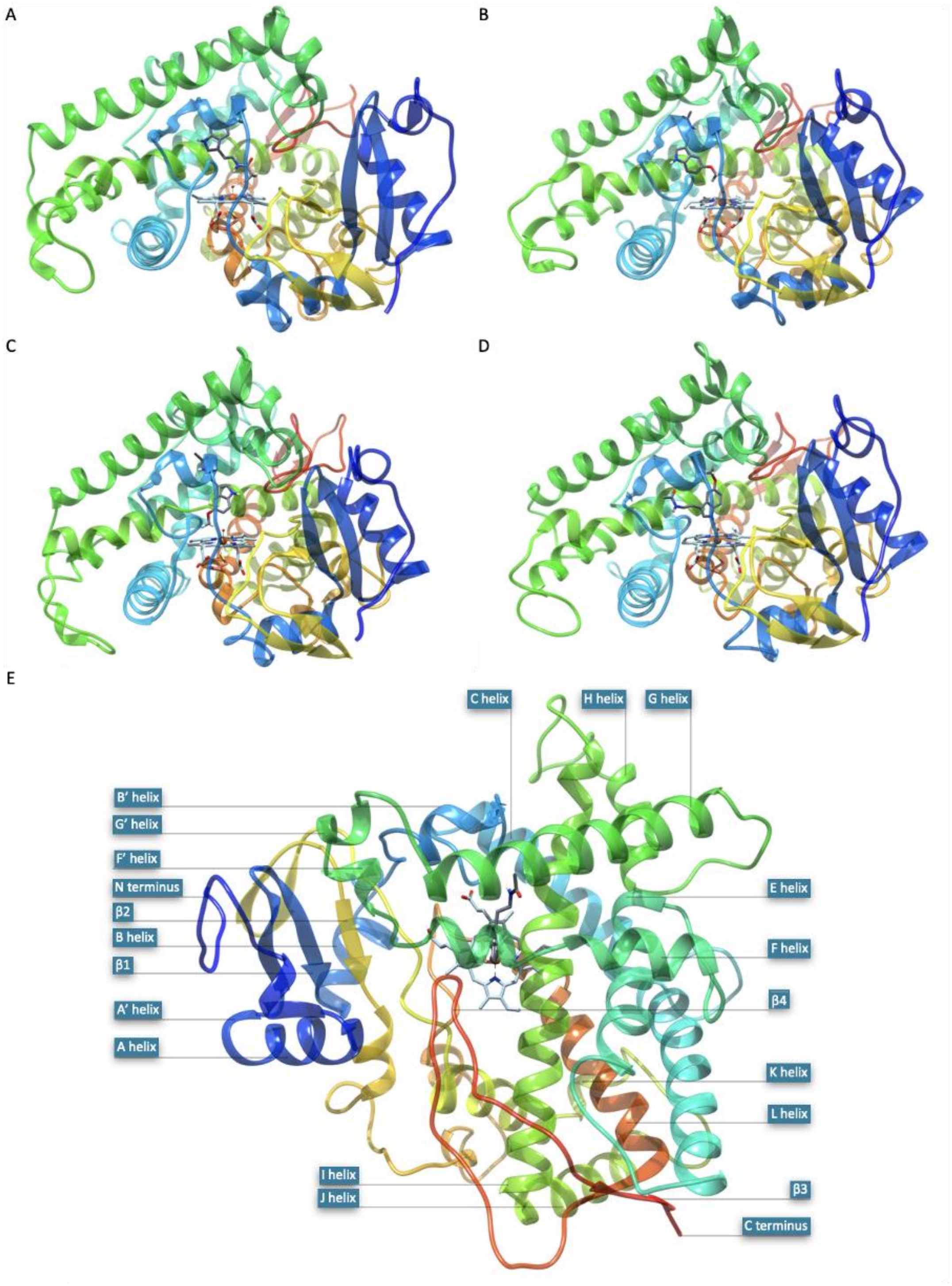

Figure S10. Overall structures of each model from the MD simulation: Model **I** (part A), Model **II** (part B), Model **III** (part C) and Model **IV** (part D). CYP1A1 coloured from blue at the N terminus to red at the V terminus with compound I as light blue and melatonin as grey sticks (A-D). Helix definitions (from Poulos, T. L. Heme Enzyme Structure and Function. *Chem. Rev*. **2014**, *114*, 3919-3962) of CYP1A1 are shown in part E.

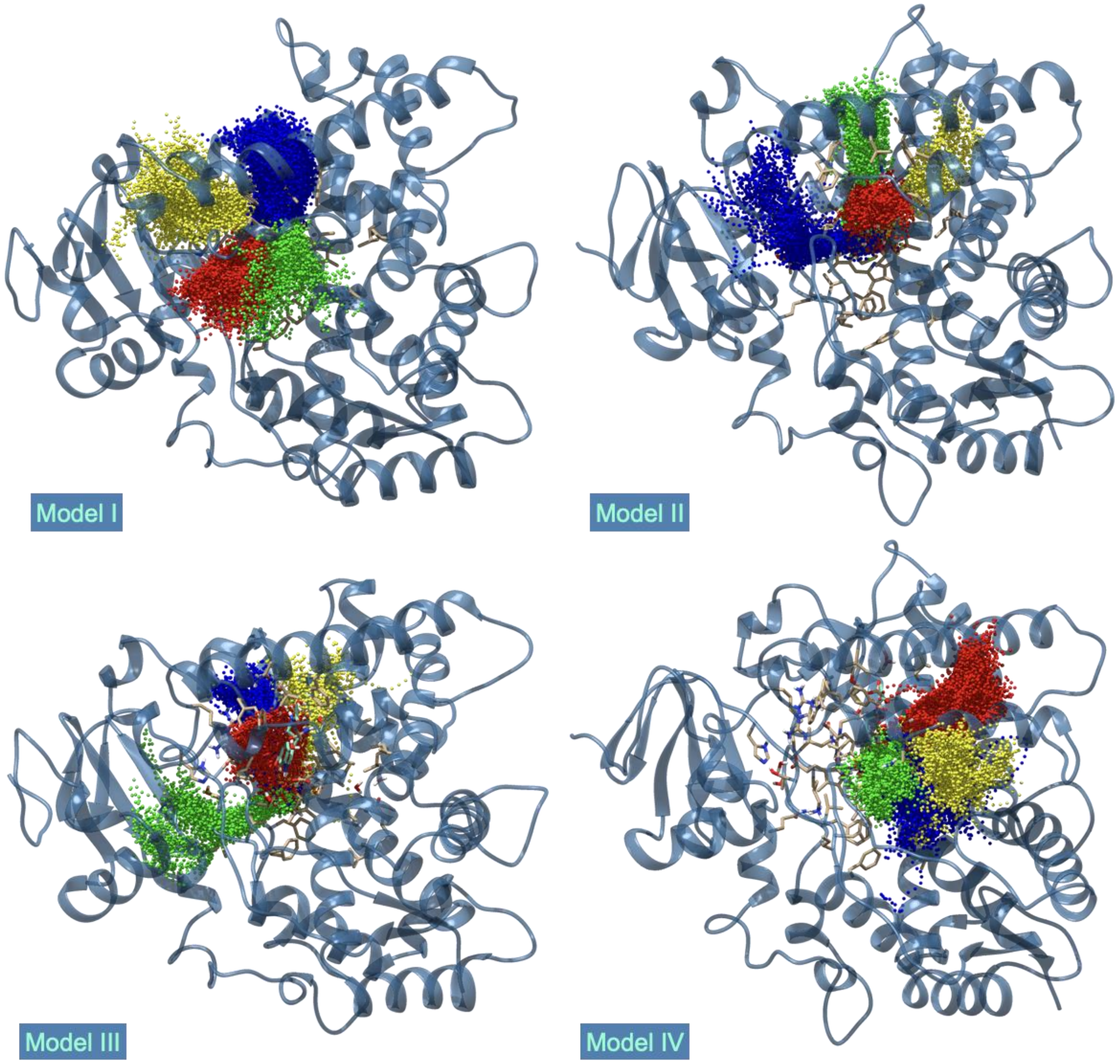

Figure S11. Tunneling analysis for the entrance and exit channels leading to CpdI as obtained from the individual snapshots from the MD simulations on CYP1A1 with melatonin with substrate starting orientation **I**, **II**, **III** and **IV** during a 100 ns. The top 4 channels are presented in red, blue, green and yellow for each of the models.

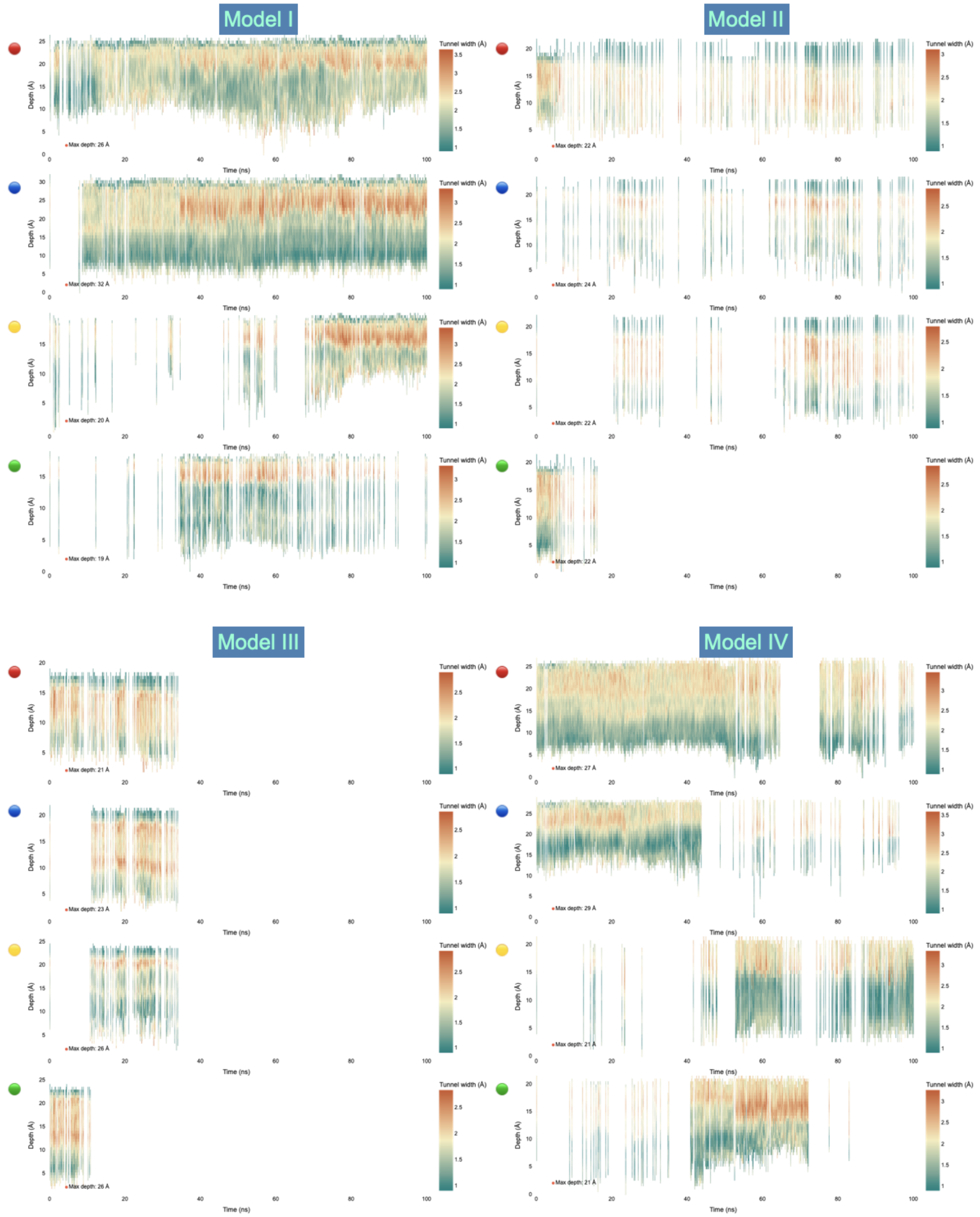

Figure S12. Tunneling analysis for the entrance and exit channels leading to CpdI as obtained from the individual snapshots from the MD simulations on CYP1A1 with melatonin with substrate starting orientation **I**, **II**, **III** and **IV** during a 100 ns. The details of top 4 channels from tunnel analysis for each model from the MD simulation are shown.

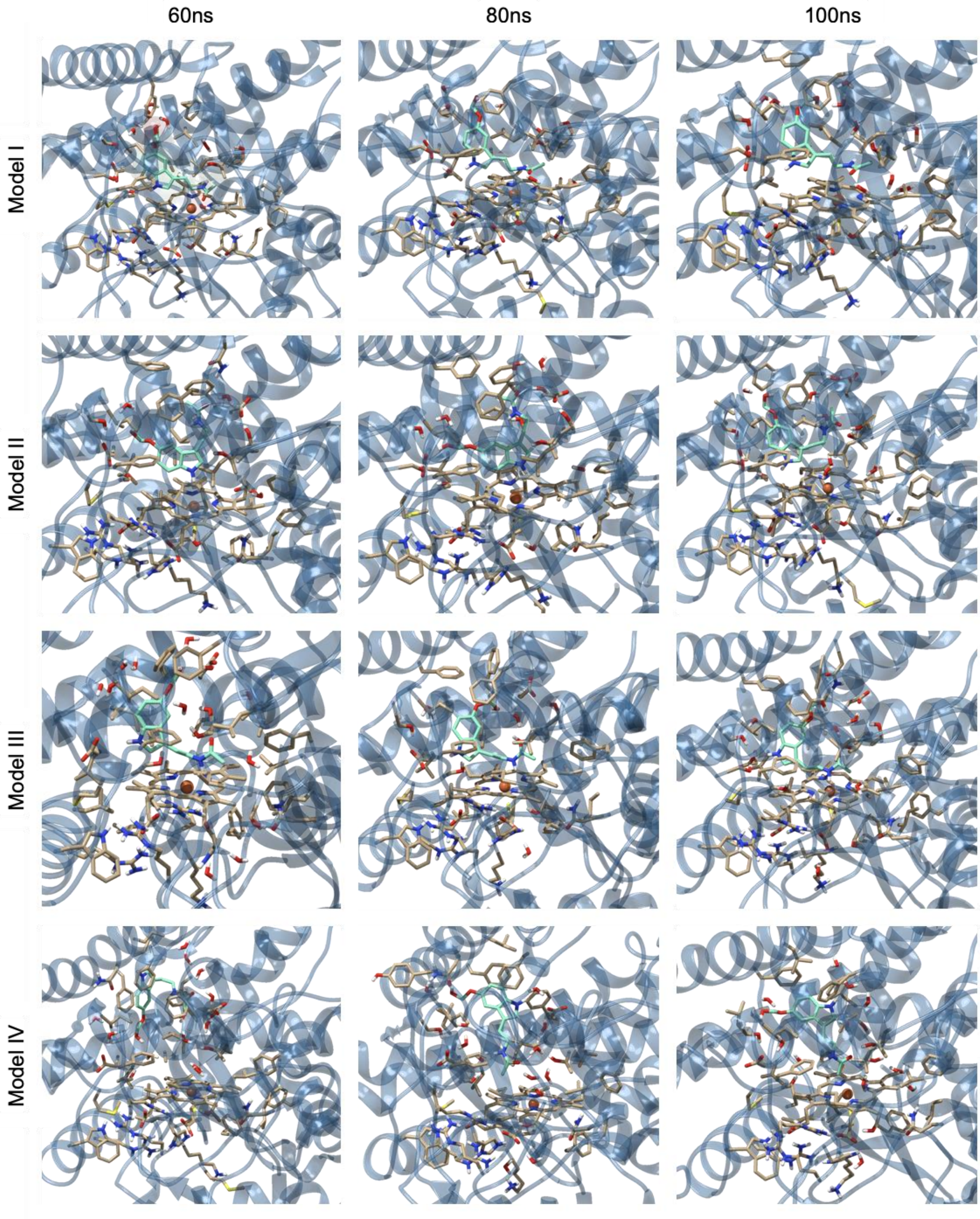

Figure S13. Selected binding poses of *O*-methoxy hydroxylated product versus heme as obtained from snapshots after 60, 80 or 100 ns from the MD simulations for CYP1A1 with melatonin with substrate starting orientation **I**, **II**, **III** and **IV**. Each MD was calculated from AutoDock Vina. The docking scores for each model were -10.1, -7.5, -9.0 and -5.2.

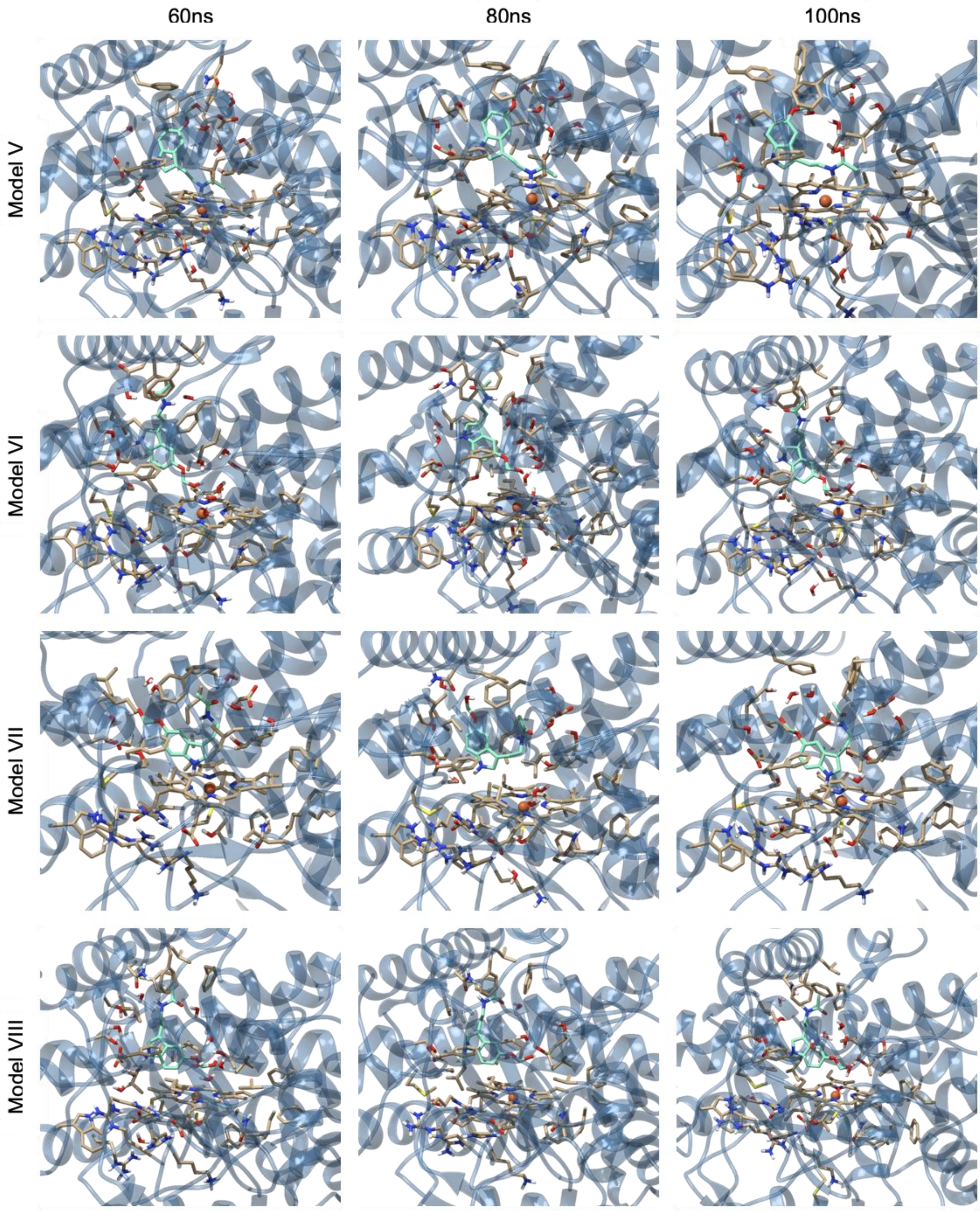

Figure S14. Selected binding poses of *O*-methoxy hydroxylated product versus heme as obtained from snapshots after 60, 80 or 100 ns from the MD simulations for CYP1A1 with melatonin with substrate starting orientation **V**, **VI**, **VII** and **VIII**. Each MD was calculated from AutoDock Vina. The docking scores for each model were -6.7, -8.8, -6.8 and -9.0.

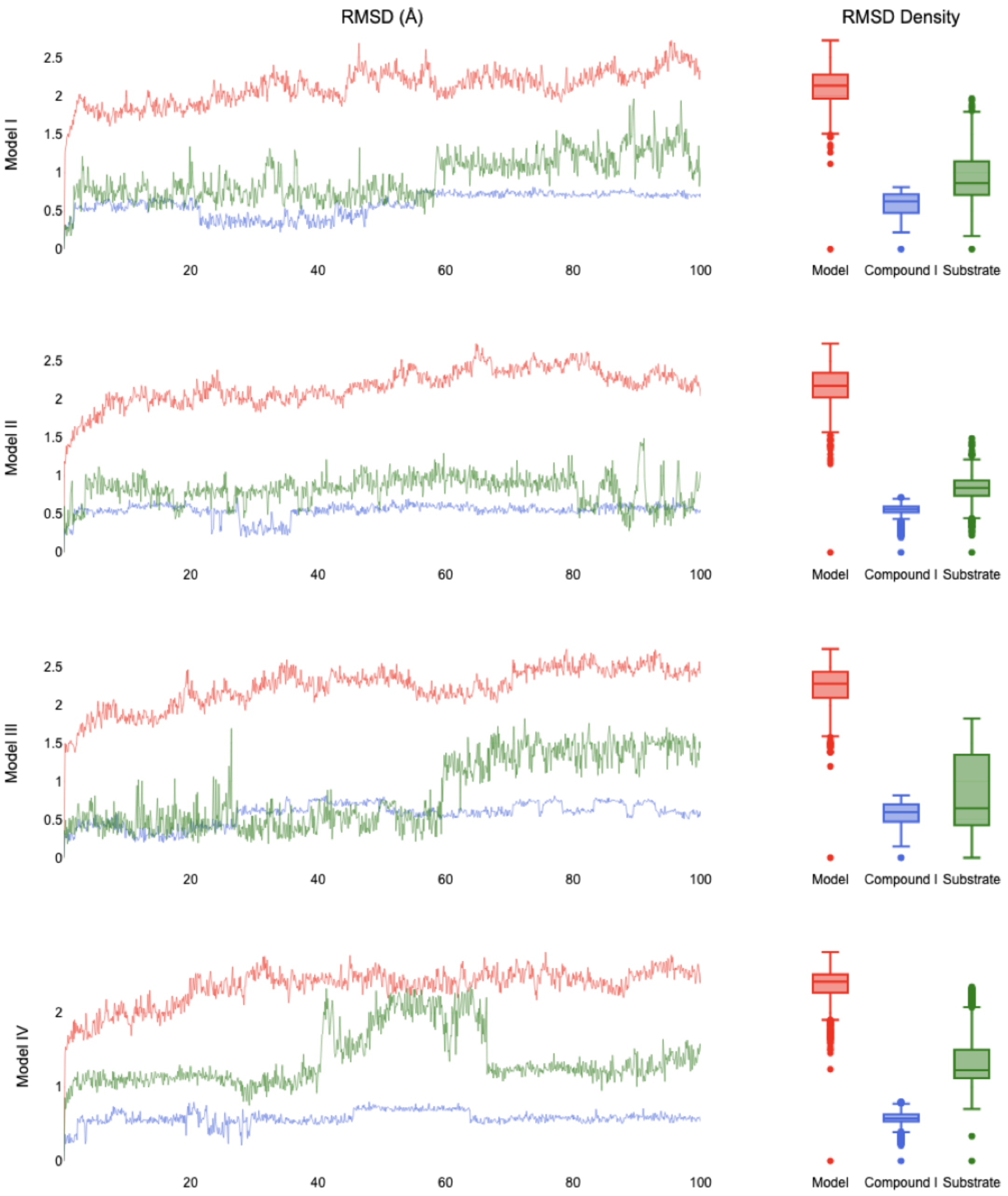

Figure S15. RMSD plots obtained from the MD simulations for *O*-methoxy hydroxylated product versus heme starting orientation **I**, **II**, **III** and **IV** during a 100 ns MD calculation. The right-hand-side shows the individual RMSD values of the whole model (in red), Compound I only (in blue) and substrate only (in green).

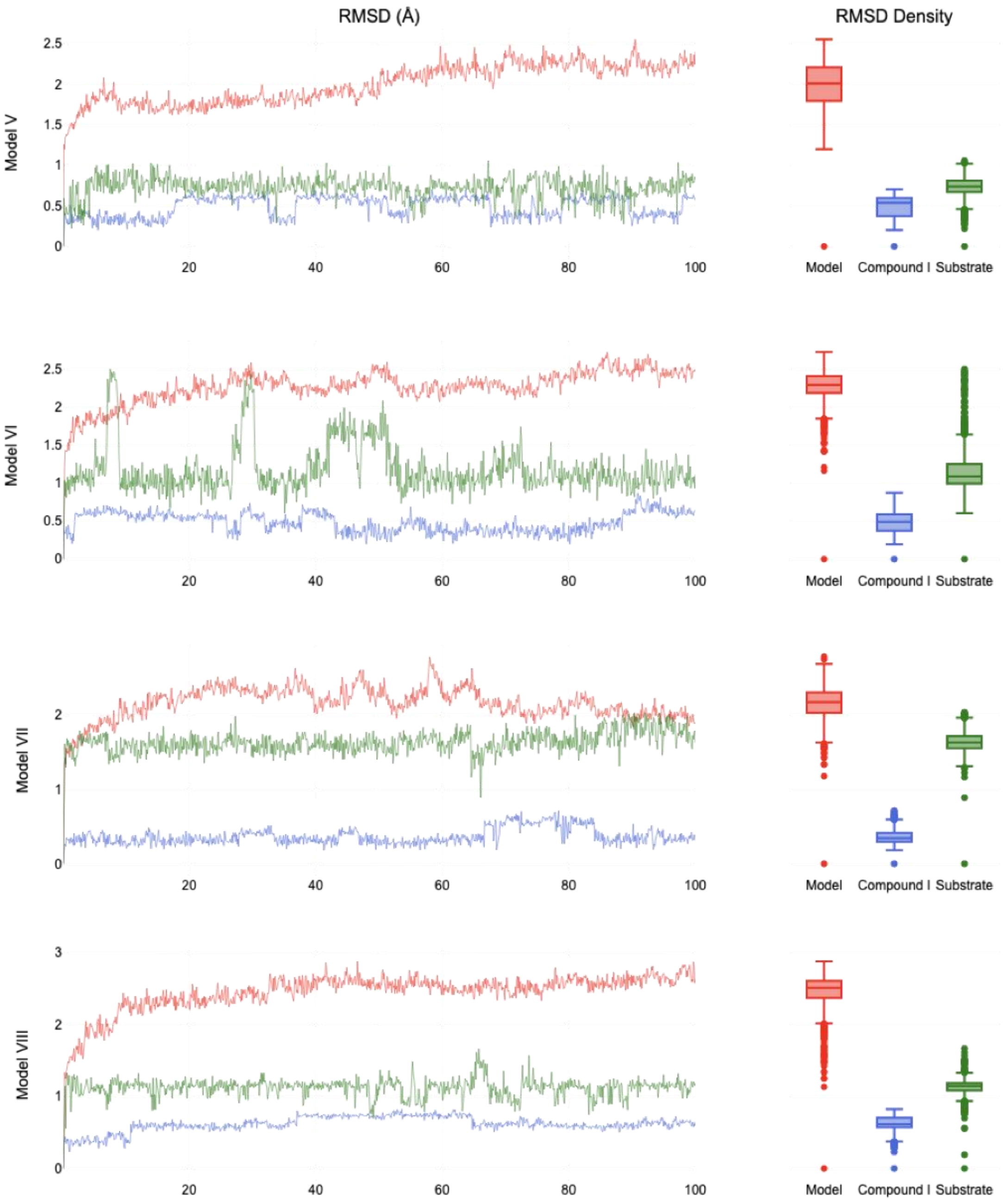

Figure S16. RMSD plots obtained from the MD simulations for *O*-methoxy hydroxylated product versus heme starting orientation **V**, **VI**, **VII** and **VIII** during a 100 ns MD calculation. The right-hand-side shows the individual RMSD values of the whole model (in red), Compound I only (in blue) and substrate only (in green).

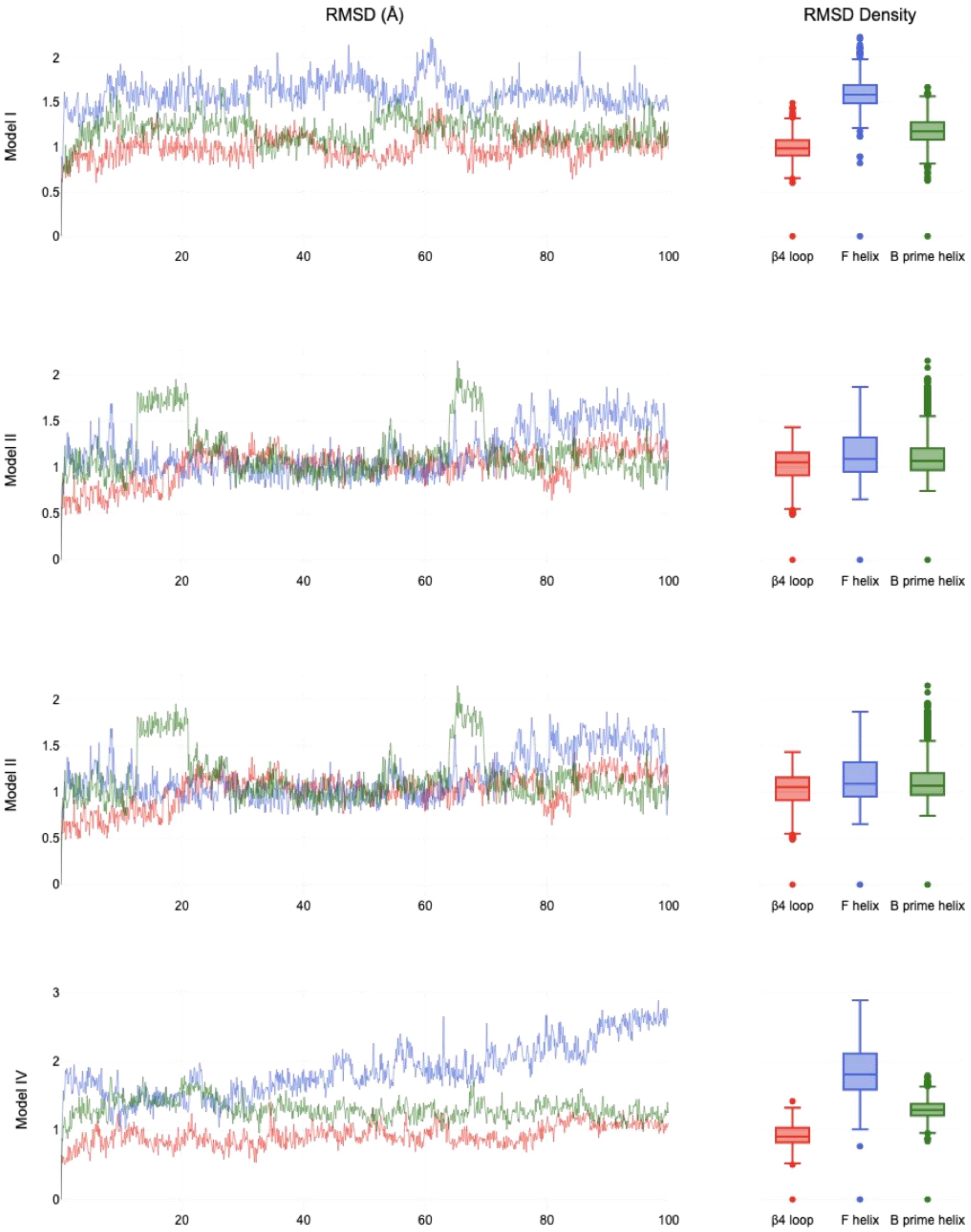

Figure S17. RMSD plots obtained from the MD simulations for *O*-methoxy hydroxylated product versus heme starting orientation **I**, **II**, **III** and **IV** for a 100 ns MD calculation focusing on the change of the protein helixes. The right-hand-side shows the individual RMSD values of the β4 loop (in red), the F-helix (in blue) and the B' helix (in green).

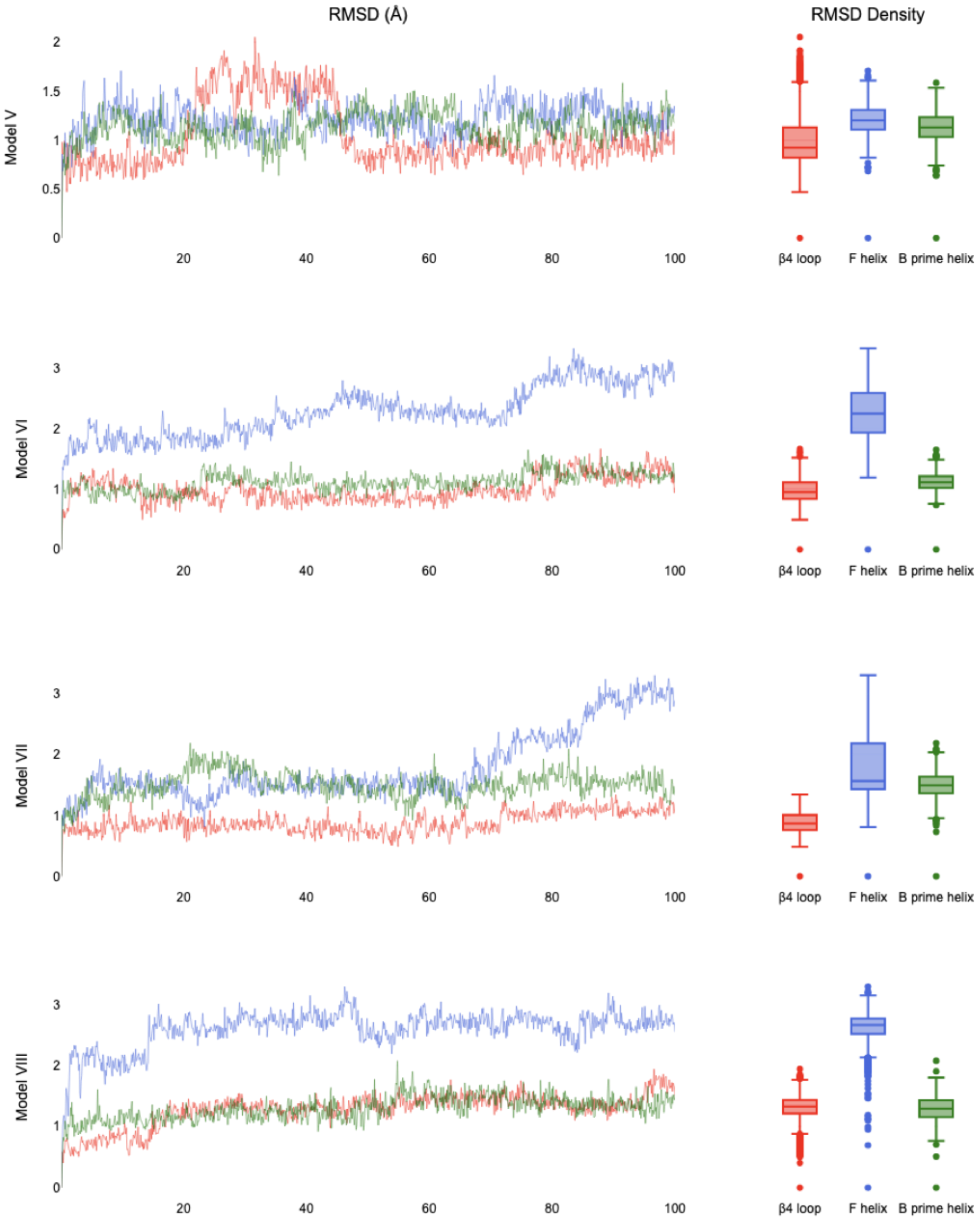

Figure S18. RMSD plots obtained from the MD simulations for *O*-methoxy hydroxylated product versus heme starting orientation **V**, **VI**, **VII** and **VIII** for a 100 ns MD calculation focusing on the change of the protein helixes. The right-hand-side shows the individual RMSD values of the β4 loop (in red), the F-helix (in blue) and the B' helix (in green).

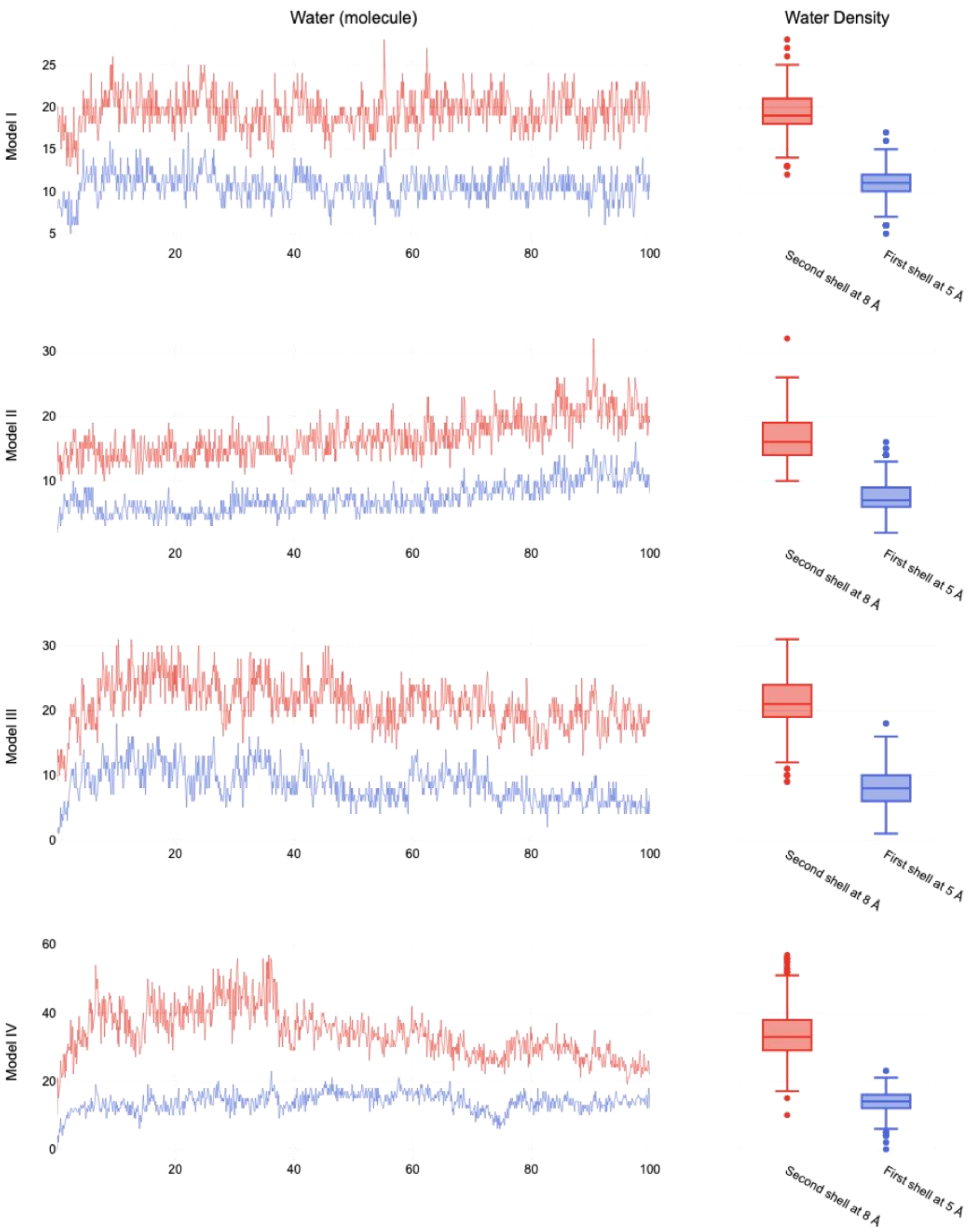

Figure S19. Protein solvation analysis obtained from the MD simulations for *O*-methoxy hydroxylated product versus heme starting orientation **I**, **II**, **III** and **IV** during a 100 ns MD calculation focusing on the change of the protein helixes. The right-hand-side counts the number of water molecules within a radius of CpdI of 5 Å (in blue) and within 8 Å (in red).

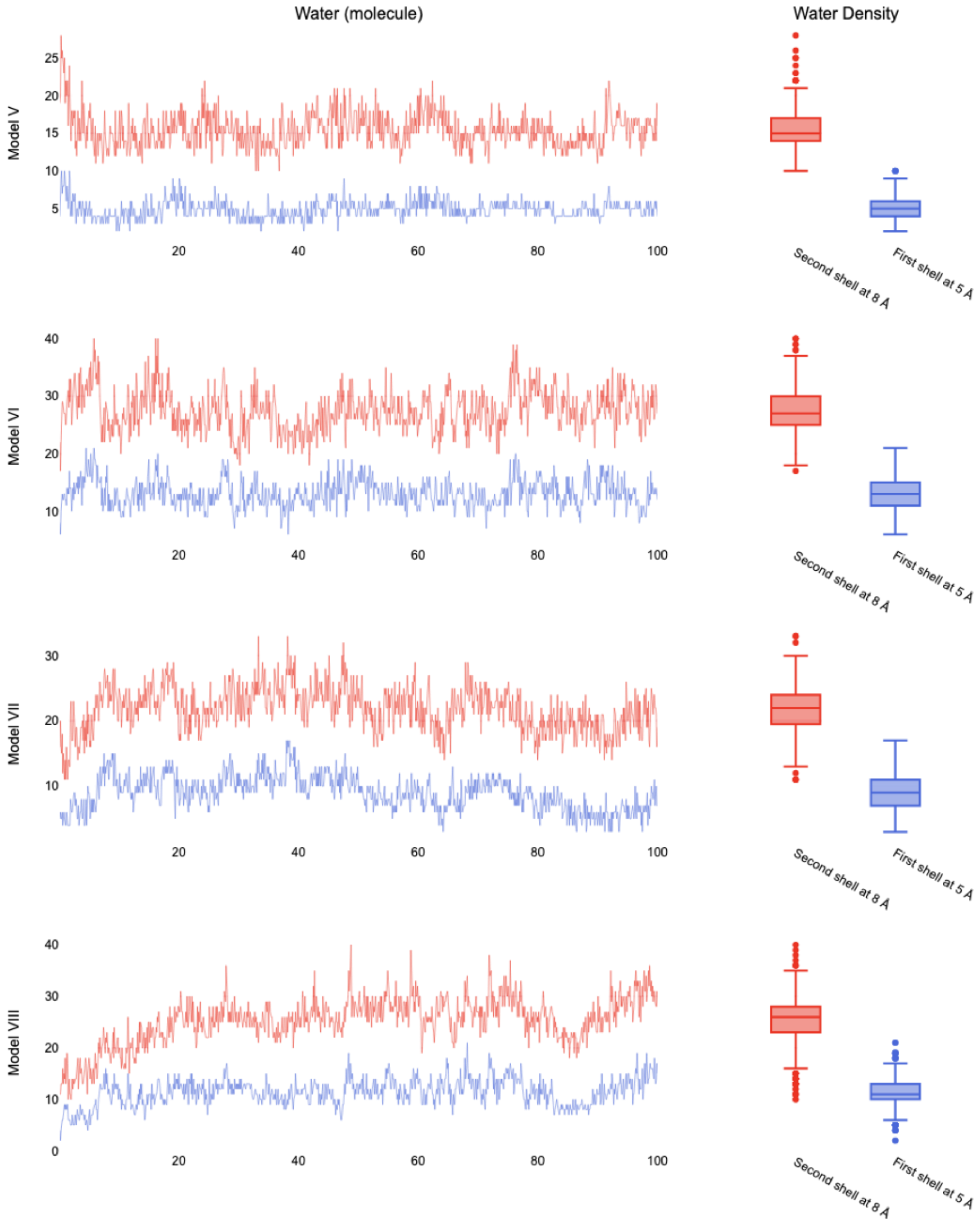

Figure S20. Protein solvation analysis obtained from the MD simulations for *O*-methoxy hydroxylated product versus heme starting orientation **V**, **VI**, **VII** and **VIII** during a 100 ns MD calculation focusing on the change of the protein helixes. The right-hand-side counts the number of water molecules within a radius of CpdI of 5 Å (in blue) and within 8 Å (in red).

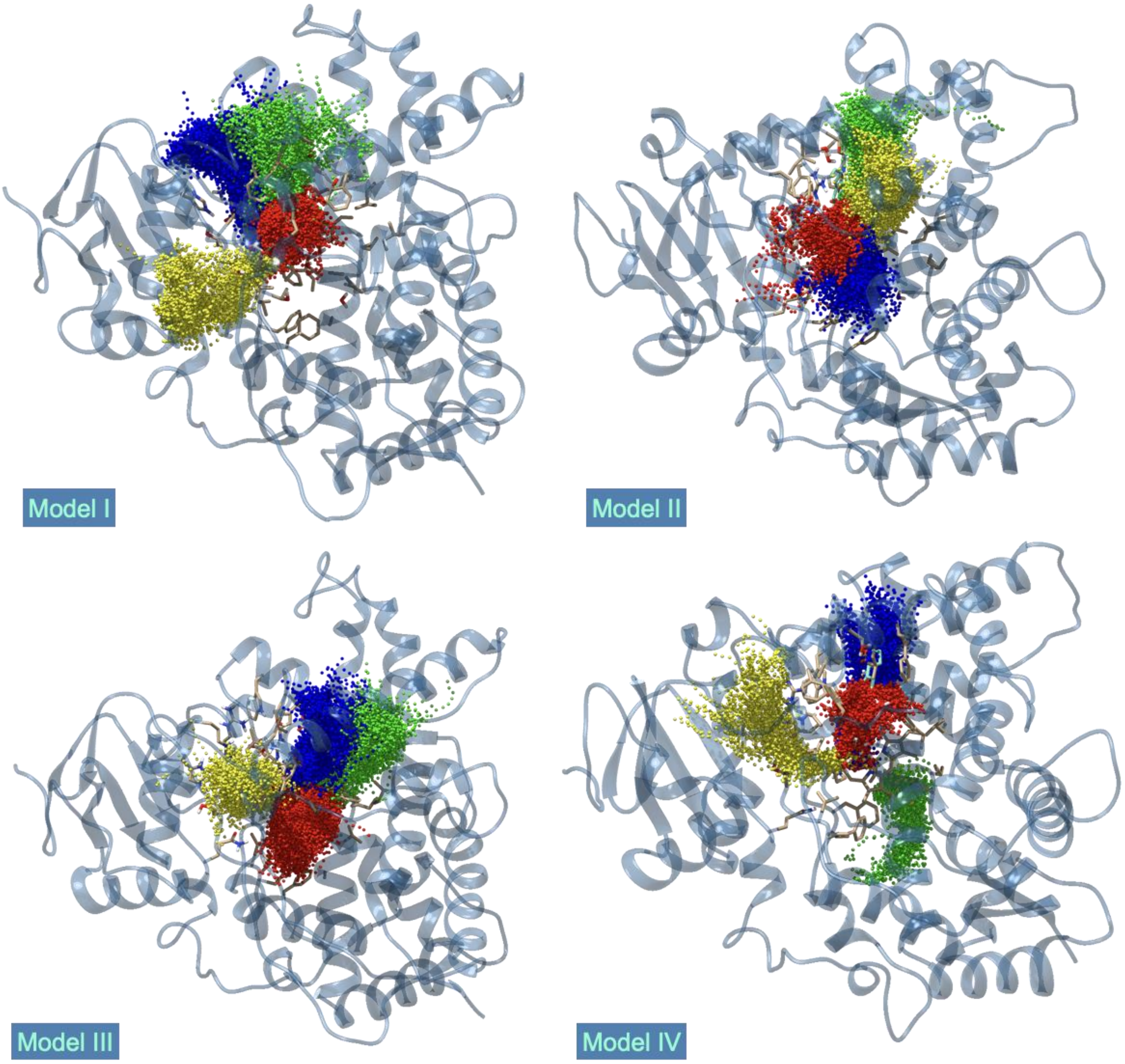

Figure S21. Tunneling analysis for the entrance and exit channels leading to heme as obtained from the individual snapshots from the MD simulations on CYP1A1 *O*-methoxy hydroxylated product versus heme starting orientation **I**, **II**, **III** and **IV** during a 100 ns. The top 4 channels are presented in red, blue, green and yellow for each of the models.

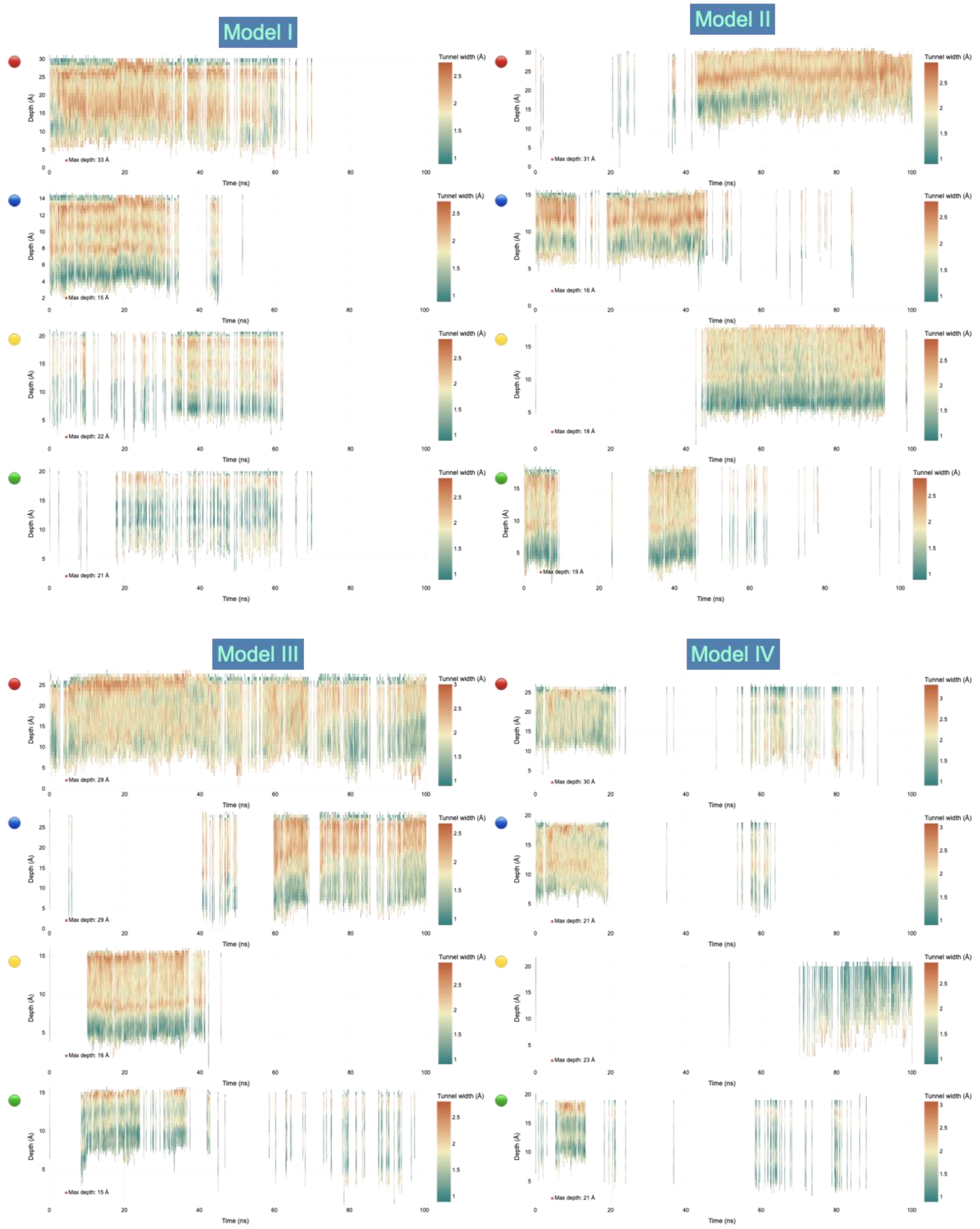

Figure S22. Tunneling analysis for the entrance and exit channels leading to heme as obtained from the individual snapshots from the MD simulations on CYP1A1 *O*-methoxy hydroxylated product versus heme starting orientation **I**, **II**, **III** and **IV** during a 100 ns. The details of top 4 channels from tunnel analysis for each model from the MD simulation are shown.

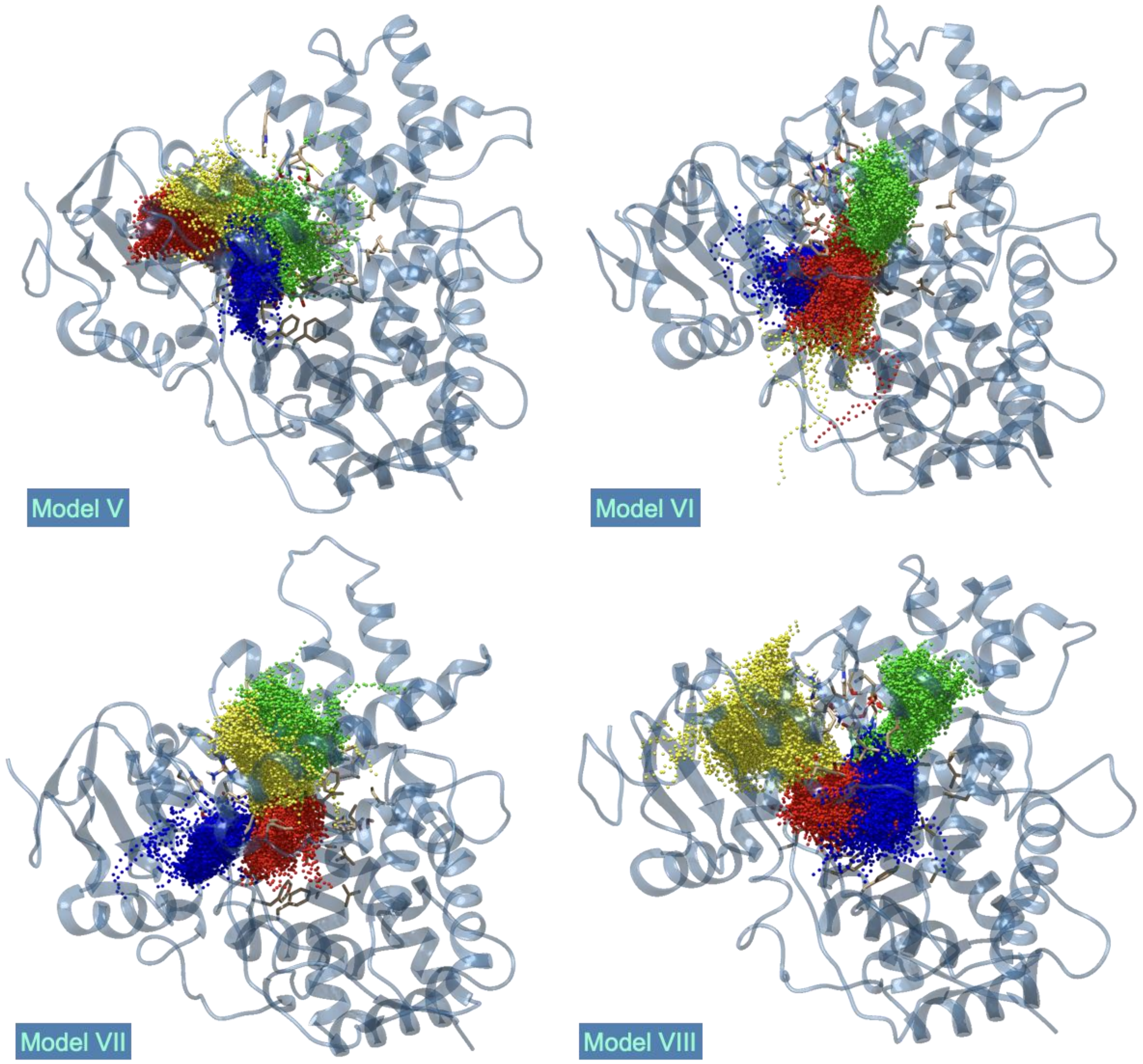

Figure S23. Tunneling analysis for the entrance and exit channels leading to heme as obtained from the individual snapshots from the MD simulations on CYP1A1 *O*-methoxy hydroxylated product versus heme starting orientation **V**, **VI**, **VII** and **VIII** during a 100 ns. The top 4 channels are presented in red, blue, green and yellow for each of the models.

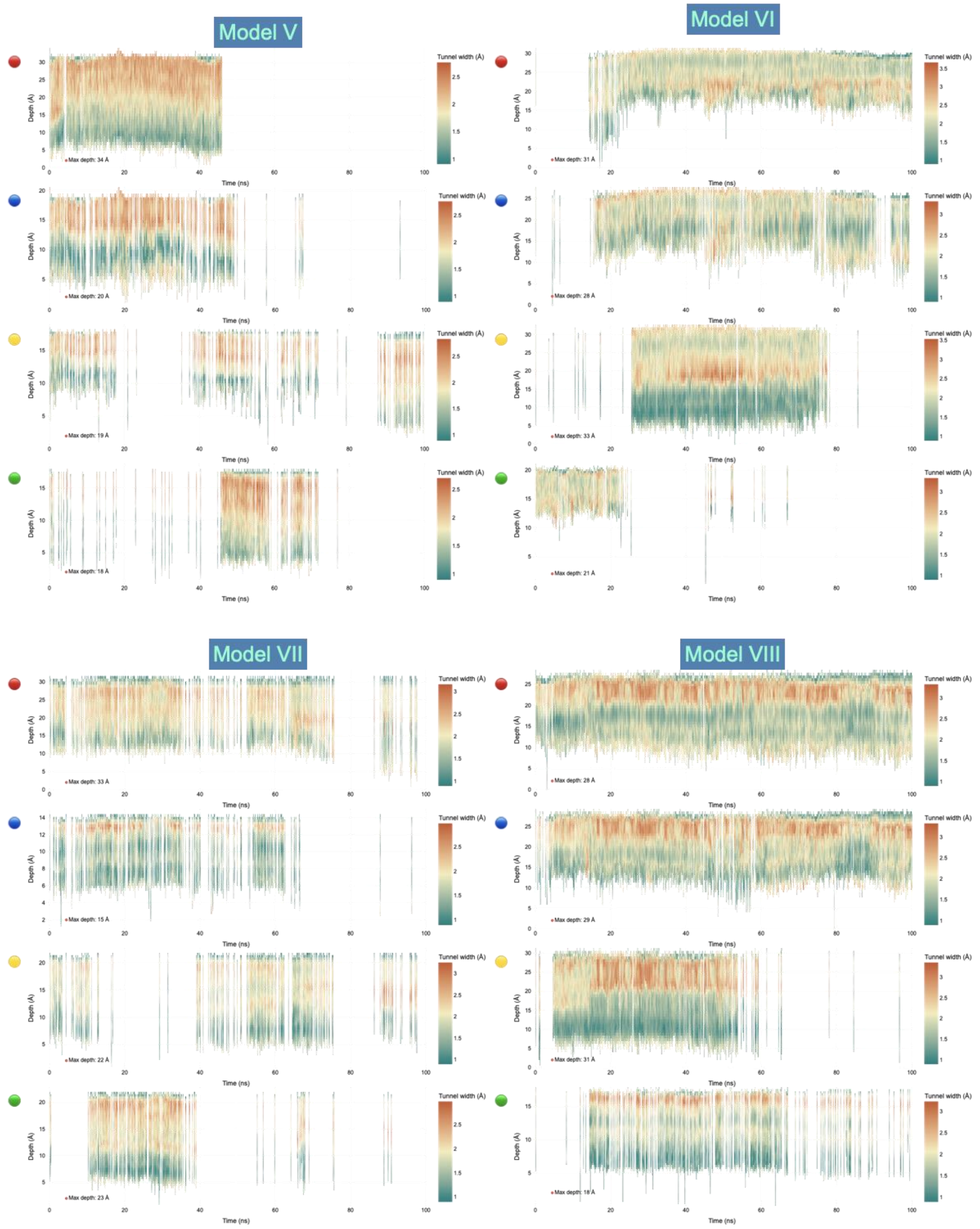

Figure S24. Tunneling analysis for the entrance and exit channels leading to heme as obtained from the individual snapshots from the MD simulations on CYP1A1 *O*-methoxy hydroxylated product versus heme starting orientation **V**, **VI**, **VII** and **VIII** during a 100 ns. The details of top 4 channels from tunnel analysis for each model from the MD simulation are shown.

QM model set-up

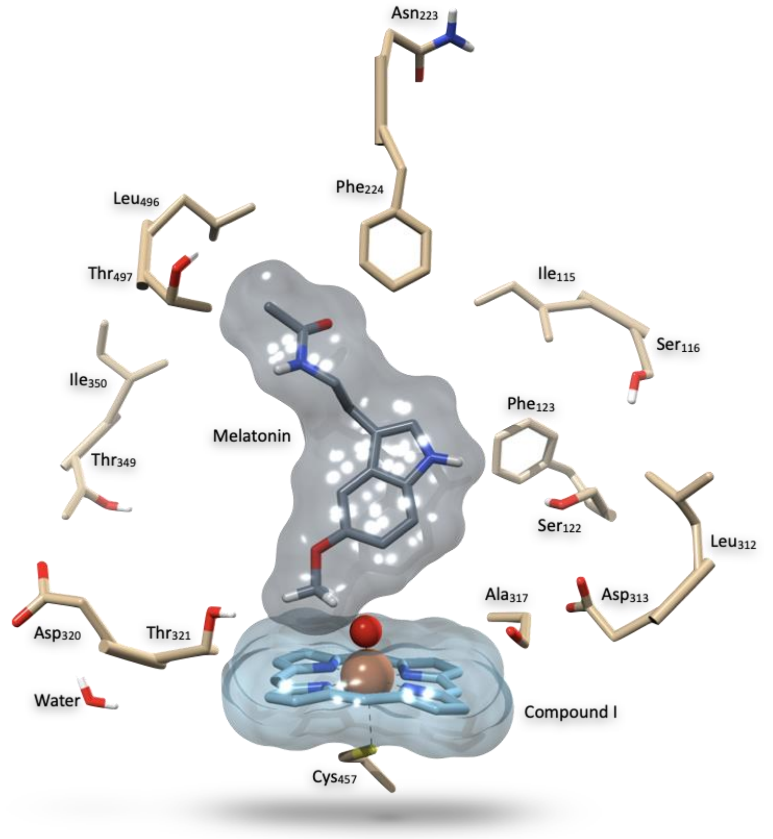

Figure S25. Cluster model ^{4}**Re**$_{AIII}$ of CYP1A1 studied in this work. The structure was taken from the MD simulation for model **III** based on the snapshot at 97 nm and modified by truncating sidechains as described in the methodology section. This model has 308 atoms.

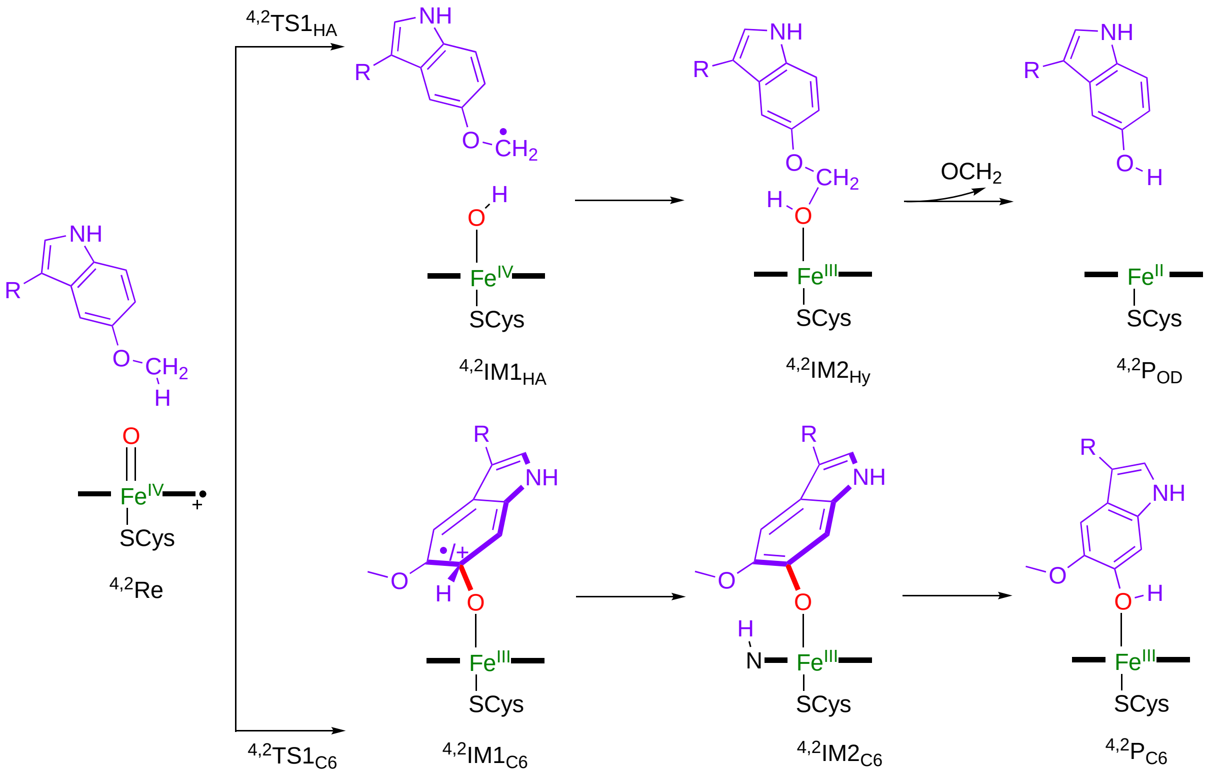

Figure S25. Calculated reaction mechanisms with definition of the labels of the structures.

QM simulation

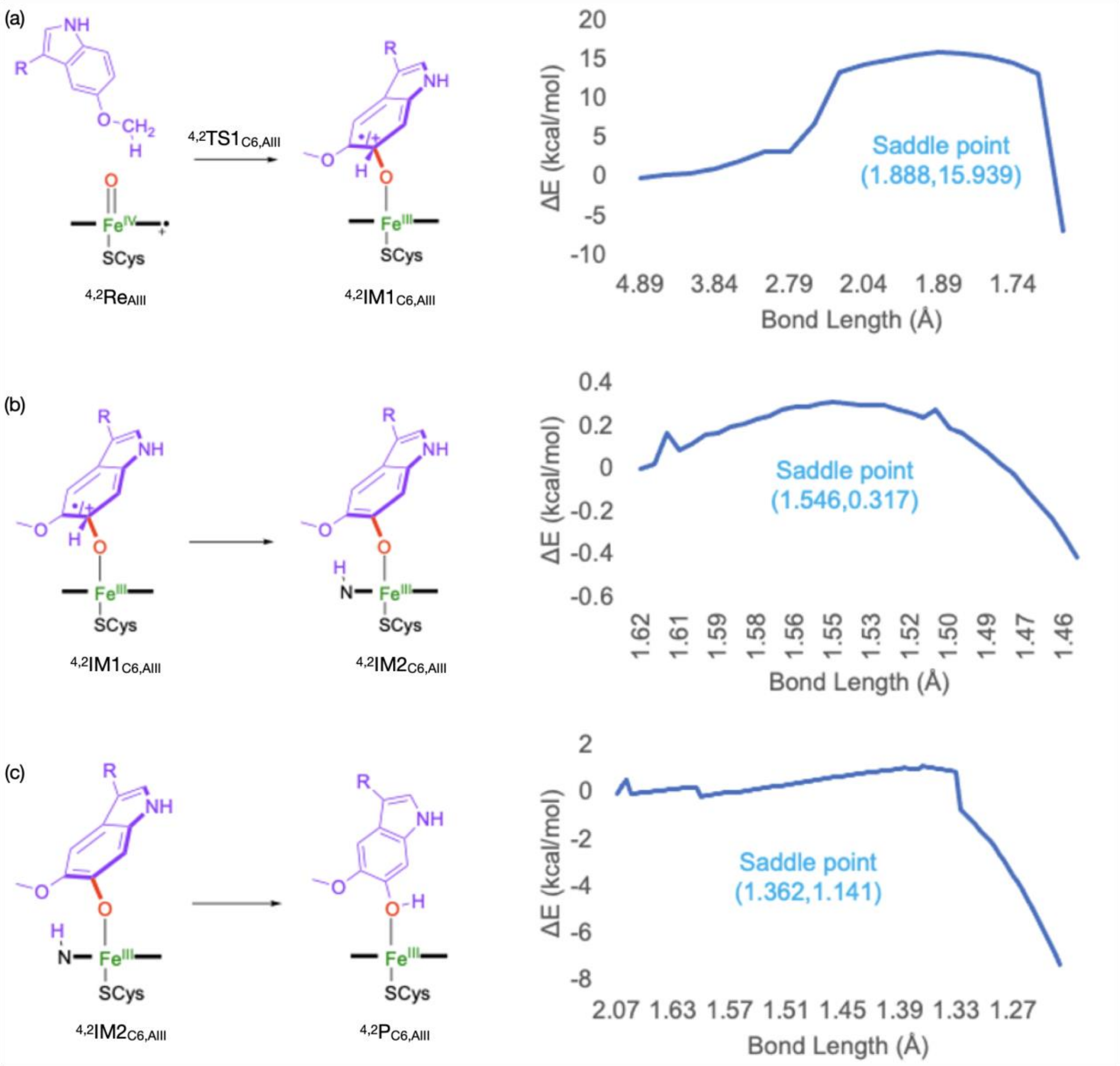

Figure S26. UB3LYP/BS1 calculated geometry scans for steps along the aromatic hydroxylation pathway. (a) The maximum for the scan from ^{4}**Re**$_{AIII}$ to ^{4}**IM1**$_{C6,AIII}$ was taken and geometry optimised to find ^{4}**TS1**$_{C6,AIII}$. (b) Geometry scan for proton transfer from the ipso position to porphyrin. This scan shows a small barrier of < 0.5 kcal mol^{-1} for proton transfer. (c) Geometry scan for proton transfer from porphyrin to phenol groups. This scan shows a small barrier of < 1.5 kcal mol^{-1} for proton transfer.

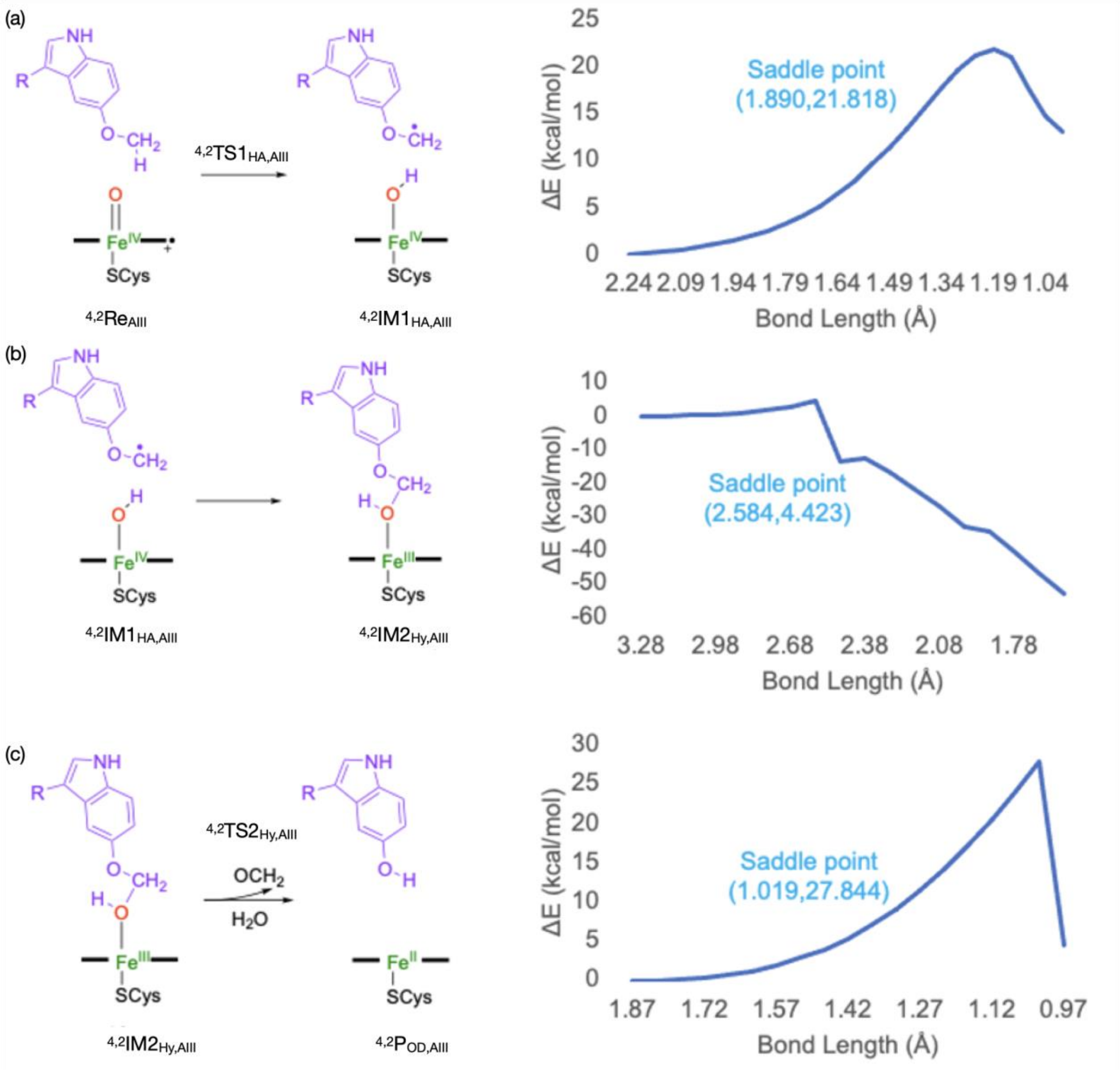

Figure S27. UB3LYP/BS1 calculated geometry scans for the *O*-demethylation pathway for model **A**. (a) Hydrogen atom abstraction scan from ^{4}**Re**$_{AIII}$. The structure at the maximum of the scan from ^{4}**Re**$_{AIII}$ to ^{4}**IM1**$_{HA,III}$ was used to search for the transition state. (b) Rebound scan between ^{4}**IM2**$_{Hy,AIII}$ to ^{4}**P**$_{OD,AIII}$. The maximum of the scan was used to search for ^{4}**TS**$_{reb,AIII}$. (c) Deformylation pathway from ^{4}**IM2**$_{Hy,AIII}$.

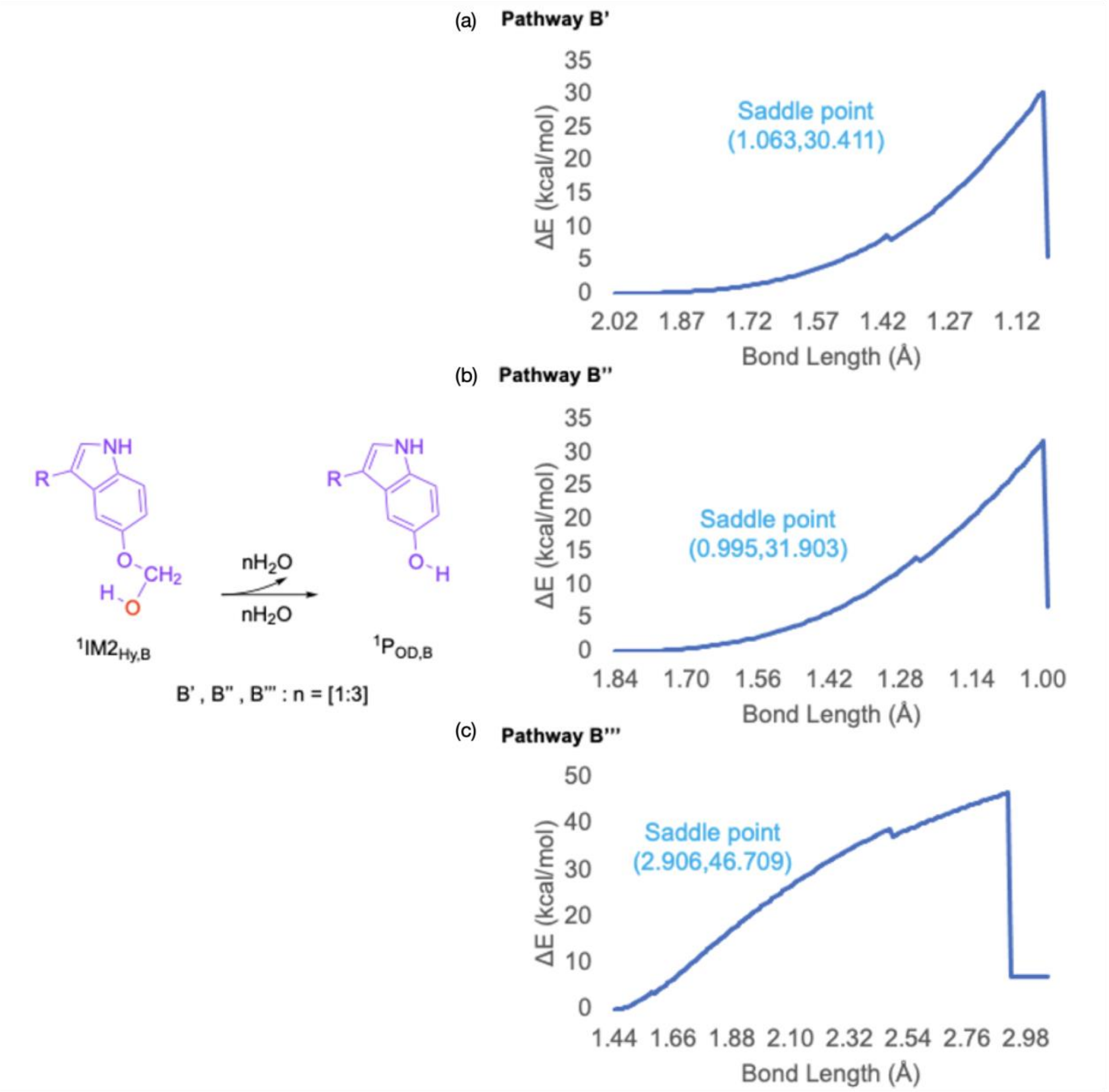

Figure S28. Geometry scans for the *O*-demethylation pathway outside the protein in a water environment, model **B**. The maxima of the scans for pathway **B'**, **B''** and **B'''** were used to find $^1\mathbf{TS}_{Hy,B'}$, $^1\mathbf{TS}_{Hy,B''}$ and $^1\mathbf{TS}_{Hy,B'}$. (a) Scan for n = 1 water. (b) Scan for n = 2 waters. (c) Scan for n = 3 waters.

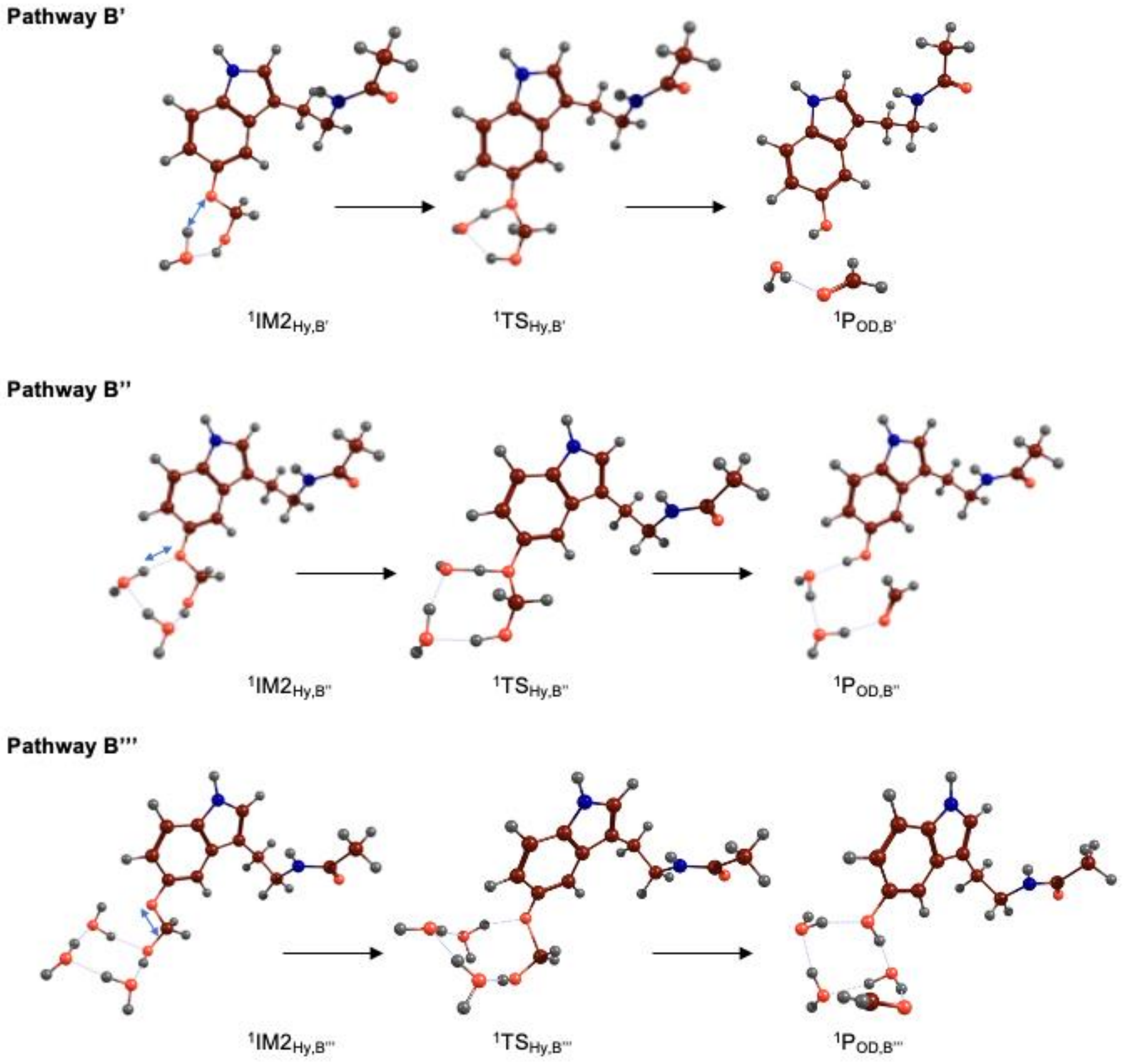

Figure S29. UB3LYP/BS1 optimized geometries for the local minima for pathways **B'**, **B''** and **B'''**.

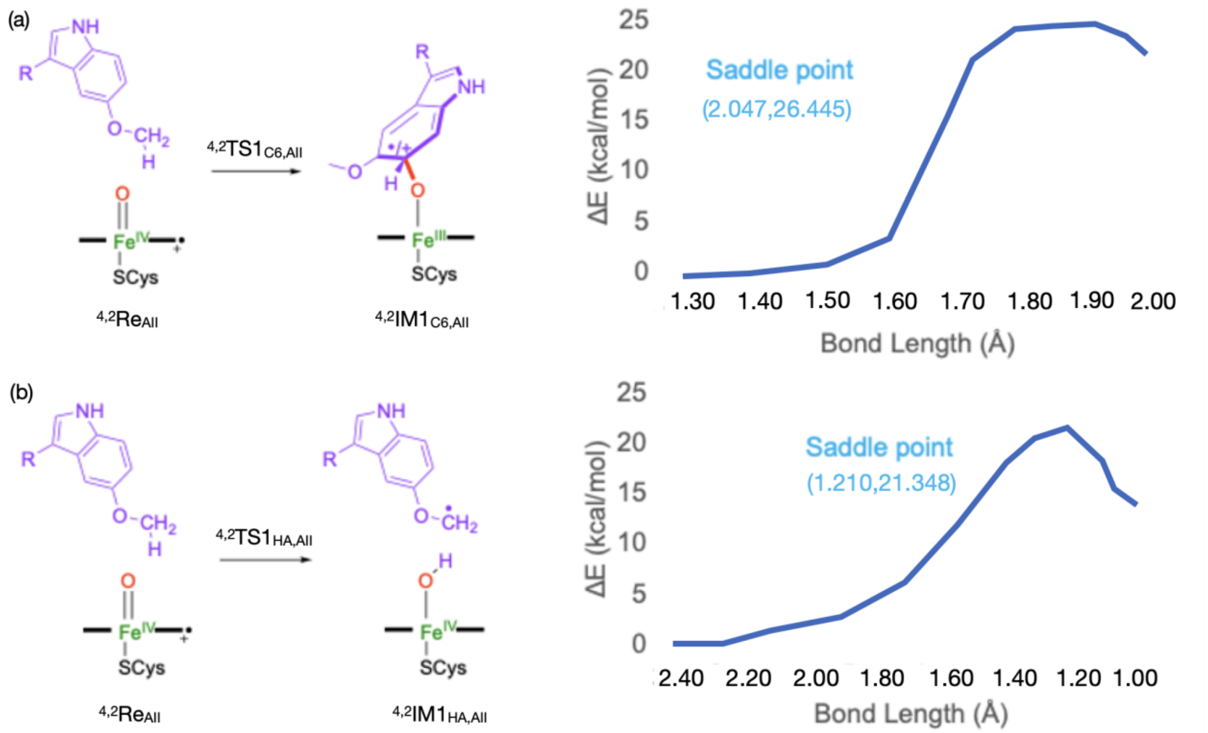

Figure S30. UB3LYP/BS1 calculated geometry scans for the *O*-demethylation pathway and the aromatic hydroxylation pathway for model **AII**. (a) The maximum for the scan from ^{4}**Re**$_{AII}$ to ^{4}**IM1**$_{C6,AII}$ was taken and geometry optimised to find ^{4}**TS1**$_{C6,AII}$. (a) Hydrogen atom abstraction scan from ^{4}**Re**$_{AII}$. The structure at the maximum of the scan from ^{4}**Re**$_{AII}$ to ^{4}**IM1**$_{HA,II}$ was used to search for the transition state.

Table S1. Absolute energies in kcal mol^{-1} of UB3LYP/BS1 optimised geometries for the metabolism of melatonin by CYP1A1 through aromatic hydroxylation in the doublet and quartet spin states (model $\mathbf{A}_{III}$).

CAL	Energy	State					MUL
		Re_{AIII}	$TS1_{C6,III}$	$IM1_{C6,III}$	$IM2_{C6,III}$	$P_{C6,III}$	
BS1	ZPE	1648.548	1648.630	1649.158	1648.825	1650.182	2
	E	-4558521.938	-4558513.369	-4558543.137	-4558560.432	-4558584.334	
	E+ZPE	-4556873.389	-4556864.739	-4556893.979	-4556911.607	-4556934.152	
	G	-4557034.488	-4557020.910	-4557051.293	-4557068.469	-4557091.216	
BS2	E	-4559698.982	-4559691.204	-4559719.535	-4559737.235	-4559762.225	
	E+ZPE	-4558050.433	-4558042.574	-4558070.376	-4558088.411	-4558112.043	
	G	-4558211.532	-4558198.745	-4558227.690	-4558245.273	-4558269.107	
BS2 / SOL	E	-4559806.597	-4559799.841	-4559825.253	-4559839.305	-4559862.704	
	E+ZPE	-4558158.049	-4558151.211	-4558176.094	-4558190.480	-4558212.522	
	G	-4558319.148	-4558307.382	-4558333.408	-4558347.342	-4558369.586	
BS3 / SOL	E	-5275393.324	-5275383.418	-5275407.803	-5275421.316	-5275444.434	
	E+ZPE	-5273744.776	-5273734.788	-5273758.645	-5273772.491	-5273794.252	
	G	-5273905.874	-5273890.959	-5273915.959	-5273929.353	-5273951.316	
BS1	ZPE	1648.596	1648.149	1648.632	1648.462	1648.779	4
	E	-4558521.869	-4558508.526	-4558515.167	-4558552.427	-4558572.936	
	E+ZPE	-4556873.273	-4556860.377	-4556866.535	-4556903.965	-4556924.157	
	G	-4557034.525	-4557018.397	-4557052.937	-4557063.108	-4557084.249	
BS2	E	-4559698.852	-4559686.731	-4559694.588	-4559731.648	-4559738.778	
	E+ZPE	-4558050.256	-4558038.581	-4558045.956	-4558083.185	-4558089.998	
	G	-4558211.508	-4558196.602	-4558232.357	-4558242.329	-4558250.091	
BS2 / SOL	E	-4559806.436	-4559792.946	-4559802.749	-4559835.200	-4559844.725	
	E+ZPE	-4558157.840	-4558144.796	-4558154.117	-4558186.737	-4558195.945	
	G	-4558319.092	-4558302.817	-4558340.519	-4558345.881	-4558356.038	
BS3 / SOL	E	-5275393.091	-5275379.247	-5275388.742	-5275411.905	-5275428.596	
	E+ZPE	-5273744.495	-5273731.098	-5273740.110	-5273763.443	-5273779.817	
	G	-5273905.747	-5273889.118	-5273926.512	-5273922.586	-5273939.909	

CAL : calculation method
MUL : multiplicity

BS1 : uB3LYP (6-31G* : (C, H, O, N, and S) // LANL2DZ : (Fe))
BS2 : uB3LYP (6-311+G* : (C, H, O, N, and S) // LANL2DZ : (Fe))
BS2 / SOL : uB3LYP (6-311+G* : (C, H, O, N, and S) // LANL2DZ : (Fe)) in chlorobenzene
BS3 / SOL : uB3LYP (6-311+G* : (C, H, O, N, and S) // cc-PVTZ : (Fe)) in chlorobenzene

Table S2. Relative energies in kcal mol^{-1} of UB3LYP/BS1 optimised geometries for the metabolism of melatonin by CYP1A1 through aromatic hydroxylation in the doublet and quartet spin state (model **A**$_{III}$).

CAL	Energy	State					MUL
		**Re**$_{AIII}$	**TS1**$_{C6,III}$	**IM1**$_{C6,III}$	**IM2**$_{C6,III}$	**P**$_{C6,III}$	
BS1	ΔE	0.000	8.569	-21.199	-38.494	-62.396	2
	Δ(E+ZPE)	0.000	8.65	-20.59	-38.218	-60.763	
	ΔG	0.000	13.578	-16.805	-33.981	-56.728	
BS2	ΔE	0.000	7.778	-20.553	-38.253	-63.243	
	Δ(E+ZPE)	0.000	7.859	-19.943	-37.978	-61.61	
	ΔG	0.000	12.787	-16.158	-33.741	-57.575	
BS2 / SOL	ΔE	0.000	6.756	-18.656	-32.708	-56.107	
	Δ(E+ZPE)	0.000	6.838	-18.045	-32.431	-54.473	
	ΔG	0.000	11.766	-14.26	-28.194	-50.438	
BS3 / SOL	ΔE	0.000	9.906	-14.479	-27.992	-51.111	
	Δ(E+ZPE)	0.000	9.988	-13.869	-27.715	-49.477	
	ΔG	0.000	14.915	-10.085	-23.479	-45.443	
BS1	ΔE	0.069	13.412	6.771	-30.489	-50.998	4
	Δ(E+ZPE)	0.116	13.012	6.854	-30.576	-50.768	
	ΔG	-0.037	16.091	-18.449	-28.620	-49.761	
BS2	ΔE	0.130	12.251	4.394	-32.666	-39.796	
	Δ(E+ZPE)	0.177	11.852	4.477	-32.752	-39.565	
	ΔG	0.024	14.930	-20.825	-30.797	-38.559	
BS2 / SOL	ΔE	0.161	13.651	3.848	-28.603	-38.128	
	Δ(E+ZPE)	0.209	13.253	3.932	-28.688	-37.896	
	ΔG	0.056	16.331	-21.371	-26.733	-36.890	
BS3 / SOL	ΔE	0.233	14.077	4.582	-18.581	-35.272	
	Δ(E+ZPE)	0.281	13.678	4.666	-18.667	-35.041	
	ΔG	0.127	16.756	-20.638	-16.712	-34.035	

CAL : calculation method
MUL : multiplicity

BS1 : uB3LYP (6-31G* : (C, H, O, N, and S) // LANL2DZ : (Fe))
BS2 : uB3LYP (6-311+G* : (C, H, O, N, and S) // LANL2DZ : (Fe))
BS2 / SOL : uB3LYP (6-311+G* : (C, H, O, N, and S) // LANL2DZ : (Fe)) in chlorobenzene
BS3 / SOL : uB3LYP (6-311+G* : (C, H, O, N, and S) // cc-PVTZ : (Fe)) in chlorobenzene

Table S3. Absolute energies in kcal mol^{-1} of UB3LYP/BS1 optimised geometries for the metabolism of melatonin by CYP1A1 through *O*-demethylation in the doublet and quartet spin state (model $\mathbf{A}_{III}$).

CAL	Energy	State				MUL
		$\mathbf{Re}_{AIII}$	$\mathbf{TS1}_{HA,III}$	$\mathbf{IM1}_{HA,III}$	$\mathbf{P}_{Hy,III}$	
BS1	ZPE	1648.548	1645.051	1646.653	1650.927	2
	E	-4558521.938	-4558498.765	-4558511.684	-4558578.499	
	E+ZPE	-4556873.389	-4556853.714	-4556865.031	-4556927.572	
	G	-4557034.488	-4557011.336	-4557024.711	-4557085.868	
BS2	E	-4559698.982	-4559676.004	-4559691.722	-4559758.53	
	E+ZPE	-4558050.433	-4558030.953	-4558045.068	-4558107.604	
	G	-4558211.532	-4558188.576	-4558204.749	-4558265.9	
BS2 / SOL	E	-4559806.597	-4559782.961	-4559796.685	-4559863.107	
	E+ZPE	-4558158.049	-4558137.91	-4558150.031	-4558212.18	
	G	-4559967.696	-4559940.583	-4559956.365	-4560021.403	
BS3 / SOL	E	-5275393.324	-5275372.05	-5275378.59	-5275445.236	
	E+ZPE	-5273744.776	-5273726.999	-5273731.937	-5273794.309	
	G	-5273905.874	-5273884.621	-5273891.617	-5273952.605	
BS1	ZPE	1648.596	1644.575	1646.655	1649.857	4
	E	-4558521.869	-4558499.518	-4558511.134	-4558573.868	
	E+ZPE	-4556873.273	-4556854.943	-4556864.479	-4556924.011	
	G	-4557034.525	-4557014.451	-4557024.709	-4557085.068	
BS2	E	-4559698.852	-4559676.665	-4559690.941	-4559755.08	
	E+ZPE	-4558050.256	-4558032.09	-4558044.286	-4558105.223	
	G	-4558211.508	-4558191.598	-4558204.516	-4558266.279	
BS2 / SOL	E	-4559806.436	-4559783.405	-4559796.034	-4559861.325	
	E+ZPE	-4558157.840	-4558138.830	-4558149.379	-4558211.468	
	G	-4559967.688	-4559942.913	-4559956.265	-4560022.382	
BS3 / SOL	E	-5275393.091	-5275367.028	-5275378.966	-5275444.362	
	E+ZPE	-5273744.495	-5273722.453	-5273732.311	-5273794.505	
	G	-5273905.747	-5273881.961	-5273892.541	-5273955.562	

CAL : calculation method

MUL : multiplicity

BS1 : uB3LYP (6-31G* : (C, H, O, N, and S) // LANL2DZ : (Fe))

BS2 : uB3LYP (6-311+G* : (C, H, O, N, and S) // LANL2DZ : (Fe))

BS2 / SOL : uB3LYP (6-311+G* : (C, H, O, N, and S) // LANL2DZ : (Fe)) in chlorobenzene

BS3 / SOL : uB3LYP (6-311+G* : (C, H, O, N, and S) // cc-PVTZ : (Fe)) in chlorobenzene

Table S4. Relative energies in kcal mol^{-1} of UB3LYP/BS1 optimised geometries for the metabolism of melatonin by CYP1A1 through *O*-demethylation in the doublet and quartet spin state (model $\mathbf{A}_{III}$).

CAL	Energy	State				MUL
		Re_{AIII}	$TS1_{HA,III}$	$IM1_{HA,III}$	$P_{Hy,III}$	
BS1	ΔE	0.000	23.173	10.254	-56.561	2
	Δ(E+ZPE)	0.000	19.675	8.358	-54.183	
	ΔG	0.000	23.152	9.777	-51.380	
BS2	ΔE	0.000	22.978	7.260	-59.548	
	Δ(E+ZPE)	0.000	19.480	5.365	-57.171	
	ΔG	0.000	22.956	6.783	-54.368	
BS2 / SOL	ΔE	0.000	23.636	9.912	-56.510	
	Δ(E+ZPE)	0.000	20.139	8.018	-54.131	
	ΔG	0.000	27.113	11.331	-53.707	
BS3 / SOL	ΔE	0.000	21.274	14.734	-51.912	
	Δ(E+ZPE)	0.000	17.777	12.839	-49.533	
	ΔG	0.000	21.253	14.257	-46.731	
BS1	ΔE	0.069	22.420	10.804	-51.930	4
	Δ(E+ZPE)	0.116	18.446	8.910	-50.622	
	ΔG	-0.037	20.037	9.779	-50.580	
BS2	ΔE	0.130	22.317	8.041	-56.098	
	Δ(E+ZPE)	0.177	18.343	6.147	-54.790	
	ΔG	0.024	19.934	7.016	-54.747	
BS2 / SOL	ΔE	0.161	23.192	10.563	-54.728	
	Δ(E+ZPE)	0.209	19.219	8.670	-53.419	
	ΔG	0.008	24.783	11.431	-54.686	
BS3 / SOL	ΔE	0.233	26.295	14.358	-51.038	
	Δ(E+ZPE)	0.281	22.322	12.465	-49.729	
	ΔG	0.127	23.912	13.333	-49.688	

CAL : calculation method
MUL : multiplicity

BS1 : uB3LYP (6-31G* : (C, H, O, N, and S) // LANL2DZ : (Fe))
BS2 : uB3LYP (6-311+G* : (C, H, O, N, and S) // LANL2DZ : (Fe))
BS2 / SOL : uB3LYP (6-311+G* : (C, H, O, N, and S) // LANL2DZ : (Fe)) in chlorobenzene
BS3 / SOL : uB3LYP (6-311+G* : (C, H, O, N, and S) // cc-PVTZ : (Fe)) in chlorobenzene

Table S5. Absolute energies in kcal mol^{-1} of UB3LYP/BS1 optimised geometries for the metabolism of melatonin by CYP1A1 through *O*-demethylation in quartet spin state (model $\mathbf{A}_{\mathrm{III}}$).

CAL	Energy	State			MUL
		$P_{Hy,AIII}$	$TS_{Hy,AIII}$	$P_{OD,AIII}$	
BS1	ZPE	1648.8471	1646.895545	1645.484276	4
	E	-4558568.434	-4558545.339	-4558535.89	
	E+ZPE	-4556919.587	-4556898.444	-4556890.405	
	G	-4557078.892	-4557053.782	-4557053.838	
BS2	E	-4559749.363	-4559725.773	-4559719.299	
	E+ZPE	-4558100.516	-4558078.877	-4558073.815	
	G	-4558259.82	-4558234.216	-4558237.247	
BS2 / SOL	E	-4559852.077	-4559830.132	-4559823.679	
	E+ZPE	-4558203.23	-4558183.236	-4558178.195	
	G	-4558362.535	-4558338.574	-4558341.627	

CAL : calculation method
MUL : multiplicity

BS1 : uB3LYP (6-31G* : (C, H, O, N, and S) // LANL2DZ : (Fe))
BS2 : uB3LYP (6-311+G* : (C, H, O, N, and S) // LANL2DZ : (Fe))
BS2 / SOL : uB3LYP (6-311+G* : (C, H, O, N, and S) // LANL2DZ : (Fe)) in chlorobenzene

Table S6. Relative energies in kcal mol^{-1} of UB3LYP/BS1 optimised geometries for the metabolism of melatonin by CYP1A1 through *O*-demethylation in quartet spin states (model $\mathbf{A}_{\text{III}}$).

CAL	Energy	State			MUL
		$P_{Hy,AIII}$	$TS_{Hy,AIII}$	$P_{OD,AIII}$	
BS1	ΔE	0.000	23.095	32.545	4
	Δ(E+ZPE)	0.000	21.143	29.182	
	ΔG	0.000	25.110	25.054	
BS2	ΔE	0.000	23.590	30.064	
	Δ(E+ZPE)	0.000	21.638	26.701	
	ΔG	0.000	25.605	22.573	
BS2 / SOL	ΔE	0.000	21.946	28.398	
	Δ(E+ZPE)	0.000	19.994	25.035	
	ΔG	0.000	23.960	20.908	

CAL : calculation method

MUL : multiplicity

BS1 : uB3LYP (6-31G* : (C, H, O, N, and S) // LANL2DZ : (Fe))

BS2 : uB3LYP (6-311+G* : (C, H, O, N, and S) // LANL2DZ : (Fe))

BS2 / SOL : uB3LYP (6-311+G* : (C, H, O, N, and S) // LANL2DZ : (Fe)) in chlorobenzene

Table S7. Absolute energies in kcal mol^{-1} of UB3LYP/BS1 optimised geometries for the non-protein *O*-demethylation via pathway **B'** in the singlet spin state.

CAL	Energy	State			MUL
		$IM2_{Hy,B'}$	$TS_{Hy,B'}$	$P_{OD,B'}$	
BS1	ZPE	191.578	0.30208	0.300963	1
	E	-575189.4	-575159	-575185.5	
	E+ZPE	-574997.8	-574969.5	-574996.7	
	G	-575030.9	-574998.7	-575032	
BS2	E	-575344.4	-575314.5	-575343	
	E+ZPE	-575152.9	-575125	-575154.1	
	G	-575185.9	-575154.2	-575189.5	
BS2 / SOL	E	-575357.7	-575327.6	-575355.6	
	E+ZPE	-575166.1	-575138	-575166.8	
	G	-575199.2	-575167.2	-575202.1	

CAL : calculation method
MUL : multiplicity

BS1 : uB3LYP (6-31G* : (C, H, O, N, and S)
BS2 : uB3LYP (6-311+G* : (C, H, O, N, and S)
BS2 / SOL : uB3LYP (6-311+G* : (C, H, O, N, and S)

Table S8. Relative energies in kcal mol^{-1} of UB3LYP/BS1 optimised geometries for the non-protein *O*-demethylation via pathway **B'** in the singlet spin state.

CAL	Energy	State			MUL
		$IM2_{Hy,B'}$	$TS_{Hy,B'}$	$P_{OD,B'}$	
BS1	ΔE	0.000	30.411	3.904	1
	Δ(E+ZPE)	0.000	28.391	1.183	
	ΔG	0.000	32.199	-1.118	
BS2	ΔE	0.000	29.896	1.451	
	Δ(E+ZPE)	0.000	27.877	-1.270	
	ΔG	0.000	31.684	-3.572	
BS2 / SOL	ΔE	0.000	30.164	2.078	
	Δ(E+ZPE)	0.000	28.144	-0.643	
	ΔG	0.000	31.952	-2.945	

CAL : calculation method
MUL : multiplicity

BS1 : uB3LYP (6-31G* : (C, H, O, N, and S)
BS2 : uB3LYP (6-311+G* : (C, H, O, N, and S)
BS2 / SOL : uB3LYP (6-311+G* : (C, H, O, N, and S)

Table S9. Absolute energies in kcal mol^{-1} of UB3LYP/BS1 optimised geometries for the non-protein *O*-demethylation via pathway **B''** in the singlet spin state.

CAL	Energy	State			MUL
		$IM2_{Hy,B''}$	$TS_{Hy,B''}$	$P_{OD,B''}$	
BS1	ZPE	207.524294	205.147916	204.770783	1
	E	-623151.96	-623119.27	-623145.2	
	E+ZPE	-622944.44	-622914.12	-622940.43	
	G	-622980.03	-622945.43	-622978.15	
BS2	E	-623326.71	-623295.59	-623323.3	
	E+ZPE	-623119.19	-623090.44	-623118.53	
	G	-623154.78	-623121.75	-623156.25	
BS2 / SOL	E	-623341.01	-623310.2	-623337.5	
	E+ZPE	-623133.48	-623105.05	-623132.73	
	G	-623169.07	-623136.36	-623170.45	

CAL : calculation method
MUL : multiplicity

BS1 : uB3LYP (6-31G* : (C, H, O, N, and S)
BS2 : uB3LYP (6-311+G* : (C, H, O, N, and S)
BS2 / SOL : uB3LYP (6-311+G* : (C, H, O, N, and S)

Table S10. Relative energies in kcal mol^{-1} of UB3LYP/BS1 optimised geometries for the non-protein *O*-demethylation via pathway **B''** in the singlet spin state.

CAL	Energy	State			MUL
		$IM2_{Hy,B''}$	$TS_{Hy,B''}$	$P_{OD,B''}$	
BS1	ΔE	0.000	32.689	6.760	1
	Δ(E+ZPE)	0.000	30.312	4.007	
	ΔG	0.000	34.596	1.876	
BS2	ΔE	0.000	31.118	3.411	
	Δ(E+ZPE)	0.000	28.742	0.657	
	ΔG	0.000	33.026	-1.473	
BS2 / SOL	ΔE	0.000	30.810	3.502	
	Δ(E+ZPE)	0.000	28.433	0.749	
	ΔG	0.000	32.717	-1.382	

CAL : calculation method
MUL : multiplicity

BS1 : uB3LYP (6-31G* : (C, H, O, N, and S)
BS2 : uB3LYP (6-311+G* : (C, H, O, N, and S)
BS2 / SOL : uB3LYP (6-311+G* : (C, H, O, N, and S)

Table S11. Absolute energies in kcal mol^{-1} of UB3LYP/BS1 optimised geometries for the non-protein *O*-demethylation via pathway **B'''** in the singlet spin state.

CAL	Energy	State			MUL
		$IM2_{Hy,B'''}$	$TS_{Hy,B'''}$	$P_{OD,B'''}$	
BS1	ZPE	223.620	221.329	221.060	1
	E	-671114.476	-671085.325	-671104.970	
	E+ZPE	-670890.856	-670863.996	-670883.909	
	G	-670928.569	-670896.081	-670923.350	
BS2	E	-671308.806	-671281.962	-671300.539	
	E+ZPE	-671085.186	-671060.633	-671079.479	
	G	-671122.898	-671092.718	-671118.920	
BS2 / SOL	E	-671324.202	-671297.939	-671315.960	
	E+ZPE	-671100.582	-671076.610	-671094.899	
	G	-671138.294	-671108.694	-671134.340	

CAL : calculation method
MUL : multiplicity

BS1 : uB3LYP (6-31G* : (C, H, O, N, and S)
BS2 : uB3LYP (6-311+G* : (C, H, O, N, and S)
BS2 / SOL : uB3LYP (6-311+G* : (C, H, O, N, and S)

Table S12. Relative energies in kcal mol^{-1} of UB3LYP/BS1 optimised geometries for the non-protein *O*-demethylation via pathway **B'''** in the singlet spin state.

CAL	Energy	State			MUL
		$IM2_{Hy,B'''}$	$TS_{Hy,B'''}$	$P_{OD,B'''}$	
BS1	ΔE	0.000	29.151	9.507	1
	Δ(E+ZPE)	0.000	26.860	6.947	
	ΔG	0.000	32.488	5.219	
BS2	ΔE	0.000	26.843	8.266	
	Δ(E+ZPE)	0.000	24.552	5.707	
	ΔG	0.000	30.181	3.978	
BS2 / SOL	ΔE	0.000	26.263	8.242	
	Δ(E+ZPE)	0.000	23.972	5.682	
	ΔG	0.000	29.600	3.954	

CAL : calculation method
MUL : multiplicity

BS1 : uB3LYP (6-31G* : (C, H, O, N, and S)
BS2 : uB3LYP (6-311+G* : (C, H, O, N, and S)
BS2 / SOL : uB3LYP (6-311+G* : (C, H, O, N, and S)

Table S13. Absolute energies in kcal mol^{-1} of UB3LYP/BS1 optimised geometries for the metabolism of melatonin by CYP1A1 through aromatic hydroxylation in the doublet and quartet spin states (model $\mathbf{A}_{II}$).

CAL	Energy	State			MUL
		Re_{AII}	$TS1_{HA,II}$	$IM1_{HA,II}$	
BS1	ZPE	1696.224695	1694.291965	1697.8763	2
	E	-4689820.631	-4689814.173	-4689867.041	
	E+ZPE	-4688124.406	-4688119.881	-4688169.165	
	G	-4688293.24	-4688283.953	-4688337.273	
BS2	E	-4691038.944	-4691029.509	-4691090.979	
	E+ZPE	-4689342.719	-4689335.218	-4689393.103	
	G	-4689511.553	-4689499.289	-4689561.21	
BS2 / SOL	E	-5406625.561	-5406620.369	-5406673.695	
	E+ZPE	-5404929.336	-5404926.077	-5404975.819	
	G	-5405098.17	-5405090.149	-5405143.927	
BS1	ZPE	1696.28117	1693.586017	1695.686291	4
	E	-4689820.551	-4689812.312	-4689834.788	
	E+ZPE	-4688124.27	-4688118.726	-4688139.101	
	G	-4688292.871	-4688283.757	-4688305.74	
BS2	E	-4691038.835	-4691028.566	-4691052.69	
	E+ZPE	-4689342.553	-4689334.98	-4689357.003	
	G	-4689511.155	-4689500.012	-4689523.642	
BS2 / SOL	E	-5406625.702	-5406616.215	-5406638.586	
	E+ZPE	-5404929.421	-5404922.629	-5404942.9	
	G	-5405098.022	-5405087.661	-5405109.538	

CAL : calculation method
MUL : multiplicity

BS1 : uB3LYP (6-31G* : (C, H, O, N, and S) // LANL2DZ : (Fe))
BS2 : uB3LYP (6-311+G* : (C, H, O, N, and S) // LANL2DZ : (Fe))
BS2 / SOL : uB3LYP (6-311+G* : (C, H, O, N, and S) // LANL2DZ : (Fe)) in chlorobenzene
BS3 / SOL : uB3LYP (6-311+G* : (C, H, O, N, and S) // cc-PVTZ : (Fe)) in chlorobenzene

Table S14. Relative energies in kcal mol^{-1} of UB3LYP/BS1 optimised geometries for the metabolism of melatonin by CYP1A1 through aromatic hydroxylation in the doublet and quartet spin state (model $\mathbf{A}_{II}$).

CAL	Energy	State			MUL
		Re_{AII}	$TS1_{HA,II}$	$IM1_{HA,II}$	
BS1	ΔE	0.000	6.458	-46.410	2
	Δ(E+ZPE)	0.000	4.526	-44.758	
	ΔG	0.000	9.288	-44.032	
BS2	ΔE	0.000	9.435	-52.035	
	Δ(E+ZPE)	0.000	7.502	-50.383	
	ΔG	0.000	12.264	-49.657	
BS3 / SOL	ΔE	0.000	5.192	-48.134	
	Δ(E+ZPE)	0.000	3.259	-46.483	
	ΔG	0.000	8.021	-45.757	
BS1	ΔE	0.000	8.239	-14.237	4
	Δ(E+ZPE)	0.137	5.681	-14.695	
	ΔG	0.370	9.483	-12.499	
BS2	ΔE	0.110	10.378	-13.746	
	Δ(E+ZPE)	0.000	7.573	-14.450	
	ΔG	0.399	11.541	-12.088	
BS3 / SOL	ΔE	-0.141	9.346	-13.025	
	Δ(E+ZPE)	-0.084	6.707	-13.564	
	ΔG	0.148	10.509	-11.368	

CAL : calculation method
MUL : multiplicity

BS1 : uB3LYP (6-31G* : (C, H, O, N, and S) // LANL2DZ : (Fe))
BS2 : uB3LYP (6-311+G* : (C, H, O, N, and S) // LANL2DZ : (Fe))
BS2 / SOL : uB3LYP (6-311+G* : (C, H, O, N, and S) // LANL2DZ : (Fe)) in chlorobenzene
BS3 / SOL : uB3LYP (6-311+G* : (C, H, O, N, and S) // cc-PVTZ : (Fe)) in chlorobenzene

Table S15. Absolute energies in kcal mol^{-1} of UB3LYP/BS1 optimised geometries for the metabolism of melatonin by CYP1A1 through *O*-demethylation in the doublet and quartet spin state (model $\mathbf{A}_{II}$).

CAL	Energy	State			MUL
		Re_{AII}	$TS1_{C6,II}$	$IM1_{C6,II}$	
BS1	ZPE	1696.224695	1697.123288	1698.151149	2
	E	-4689820.631	-4689818.164	-4689844.582	
	E+ZPE	-4688124.406	-4688121.04	-4688146.431	
	G	-4688293.24	-4688283.892	-4688309.683	
BS2	E	-4691038.944	-4691033.812	-4691064.032	
	E+ZPE	-4689342.719	-4689336.688	-4689365.881	
	G	-4689511.553	-4689499.54	-4689529.132	
BS2 / SOL	E	-5406625.561	-5406619.993	-5406646.375	
	E+ZPE	-5404929.336	-5404922.87	-5404948.224	
	G	-5405098.17	-5405085.721	-5405111.476	
BS1	ZPE	1696.28117	1696.527154	1696.939428	4
	E	-4689820.551	-4689811.416	-4689833.192	
	E+ZPE	-4688124.27	-4688114.889	-4688136.252	
	G	-4688292.871	-4688278.996	-4688301.34	
BS2	E	-4691038.835	-4691026.562	-4691053.545	
	E+ZPE	-4689342.553	-4689330.035	-4689356.605	
	G	-4689511.155	-4689494.142	-4689521.693	
BS2 / SOL	E	-5406625.702	-5406613.849	-5406639.457	
	E+ZPE	-5404929.421	-5404917.322	-5404942.517	
	G	-5405098.022	-5405081.429	-5405107.605	

CAL : calculation method
MUL : multiplicity

BS1 : uB3LYP (6-31G* : (C, H, O, N, and S) // LANL2DZ : (Fe))
BS2 : uB3LYP (6-311+G* : (C, H, O, N, and S) // LANL2DZ : (Fe))
BS2 / SOL : uB3LYP (6-311+G* : (C, H, O, N, and S) // LANL2DZ : (Fe)) in chlorobenzene
BS3 / SOL : uB3LYP (6-311+G* : (C, H, O, N, and S) // cc-PVTZ : (Fe)) in chlorobenzene

Table S16. Relative energies in kcal mol^{-1} of UB3LYP/BS1 optimised geometries for the metabolism of melatonin by CYP1A1 through *O*-demethylation in the doublet and quartet spin state (model **A**$_{II}$).

CAL	Energy	State			MUL
		Re$_{AII}$	TS1$_{C6,II}$	IM1$_{C6,II}$	
BS1	ΔE	0.000	2.467	-23.951	2
	Δ(E+ZPE)	0.000	3.366	-22.025	
	ΔG	0.000	9.349	-16.443	
BS2	ΔE	0.000	5.133	-25.088	
	Δ(E+ZPE)	0.000	6.031	-23.161	
	ΔG	0.000	12.014	-17.579	
BS3 / SOL	ΔE	0.000	5.568	-20.814	
	Δ(E+ZPE)	0.000	6.466	-18.888	
	ΔG	0.000	12.449	-13.306	
BS1	ΔE	0.000	9.135	-12.641	4
	Δ(E+ZPE)	0.137	9.517	-11.846	
	ΔG	0.370	14.244	-8.100	
BS2	ΔE	0.110	12.382	-14.601	
	Δ(E+ZPE)	0.166	12.685	-13.886	
	ΔG	0.399	17.412	-10.140	
BS3 / SOL	ΔE	-0.141	11.712	-13.896	
	Δ(E+ZPE)	-0.084	12.014	-13.181	
	ΔG	0.148	16.741	-9.435	

CAL : calculation method
MUL : multiplicity

BS1 : uB3LYP (6-31G* : (C, H, O, N, and S)
BS2 : uB3LYP (6-311+G* : (C, H, O, N, and S)
BS2 / SOL : uB3LYP (6-311+G* : (C, H, O, N, and S)

Table S17. Group charges (doublet/quartet) of UB3LYP/BS1 optimised geometries for the metabolism of melatonin by CYP1A1 through *aromatic hydroxylation* in the doublet and quartet spin state (model $\mathbf{A}_{\mathrm{III}}$).

Residue group	Composition	$\mathbf{Re}_{\mathbf{AIII}}$	$\mathbf{TS}_{\mathbf{C6,III}}$	$\mathbf{IM1}_{\mathbf{C6,III}}$	$\mathbf{IM2}_{\mathbf{C6,III}}$	$\mathbf{P}_{\mathbf{C6,III}}$
Compound I	Fe	0.479 / 0.475	0.266 / 0.406	0.209 / 0.372	0.206 / 0.367	0.184 / 0.412
	O	-0.378 / -0.375	-0.486 / -0.526	-0.551 / -0.548	-0.57 / -0.597	-0.638 / -0.637
	Fe-O*	0.101 / 0.1	-0.221 / -0.119	-0.342 / -0.177	-0.364 / -0.23	-0.454 / -0.225
	Por*	-0.187 / -0.186	-0.482 / -0.39	-0.57 / -0.765	-0.32 / -0.438	-0.486 / -0.459
	S	0.052 / 0.051	0.023 / 0.036	0.03 / 0.087	0.028 / -0.019	0.119 / -0.06
	S-Me*	0.013 / 0.012	-0.061 / -0.031	-0.056 / 0.018	-0.042 / -0.082	0.073 / -0.117
Melatonin	C_6*	-0.202 / -0.202	-0.087 / -0.02	0.102 / 0.083	0.321 / 0.333	0.325 / 0.325
	H_{C6}*	0.122 / 0.122	0.186 / 0.164	0.156 / 0.159	0.388 / 0.405	0.449 / 0.426
	H-Me*	0.196 / 0.196	0.177 / 0.172	0.189 / 0.179	0.178 / 0.186	0.177 / 0.181
	The rest atoms*	-0.148 / -0.147	0.357 / 0.112	0.348 / 0.37	-0.351 / -0.302	-0.282 / -0.305
Environment*		-1.895 / -1.894	-1.869 / -1.887	-1.825 / -1.867	-1.809 / -1.873	-1.802 / -1.827
**Total****		-2 / -2	-2 / -2	-2 / -2	-2 / -2	-2 / -2

**** is the summation of ***

Table S18. Group spin densities (doublet/quartet) of UB3LYP/BS1 optimised geometries for the metabolism of melatonin by CYP1A1 through *aromatic hydroxylation* in the doublet and quartet spin state (model $\mathbf{A}_{\mathrm{III}}$).

Residue group	Composition	$\mathbf{Re}_{\mathbf{AIII}}$	$\mathbf{TS}_{\mathbf{C6,III}}$	$\mathbf{IM1}_{\mathbf{C6,III}}$	$\mathbf{IM2}_{\mathbf{C6,III}}$	$\mathbf{P}_{\mathbf{C6,III}}$
Compound I	Fe	1.197 / 1.095	1.002 / 1.817	0.989 / 2.999	0.98 / 2.951	0.98 / 2.47
	O	0.888 / 0.917	0.13 / 0.436	0.039 / 0.003	0.05 / 0.052	0.05 / 0.006
	Fe-O*	2.085 / 2.013	1.132 / 2.254	1.028 / 3.003	1.03 / 3.003	1.03 / 2.476
	Por*	-0.361 / 0.294	-0.094 / -0.109	-0.08 / 0.182	-0.08 / 0.077	-0.08 / 0.021
	S	-0.709 / 0.675	0.019 / 0.157	0.046 / -0.207	0.055 / -0.051	0.055 / 0.488
	S-Me*	-0.722 / 0.687	0.017 / 0.163	0.045 / -0.214	0.054 / -0.047	0.054 / 0.503
Melatonin	C_6*	0 / 0	-0.031 / 0.021	-0.003 / -0.001	0.002 / 0.003	0.002 / -0.002
	H_{C6}*	0 / 0	0.003 / 0.013	0 / 0.001	0 / 0.001	0 / -0.002
	H-Me*	0 / -0.001	0 / 0.002	0 / 0	0 / 0	0 / 0
	The rest atoms*	-0.001 / 0.006	-0.026 / 0.651	0.011 / 0.03	-0.006 / -0.037	-0.006 / 0.004
Environment*		-0.001 / 0.001	0 / 0.004	0 / 0	0 / 0	0 / 0
**Total****		1 / 3	1 / 3	1 / 3	1 / 3	1 / 3

**** is the summation of ***

Table S19 Group charges (doublet/quartet) of UB3LYP/BS1 optimised geometries for the metabolism of melatonin by CYP1A1 through *O*-demethylation in the doublet and quartet spin state (model **A**$_{III}$).

Residue group	Composition	Re_{AIII}	$TS_{HA,III}$	$IM1_{HA,III}$	$P_{Hy,III}$
Compound I	Fe	0.479 / 0.475	0.358 / 0.401	0.325 / 0.334	0.175 / 0.423
	O	-0.378 / -0.375	-0.57 / -0.578	-0.688 / -0.687	-0.586 / -0.585
	Fe-O*	0.101 / 0.1	-0.212 / -0.177	-0.362 / -0.353	-0.411 / -0.162
	Por*	-0.187 / -0.186	-0.277 / -0.256	-0.194 / -0.19	-0.488 / -0.477
	S	0.052 / 0.051	0.024 / 0.067	0.092 / 0.084	0.132 / -0.056
	S-Me*	0.013 / 0.012	-0.048 / 0.024	0.054 / 0.046	0.085 / -0.116
Melatonin	C_6*	-0.202 / -0.202	-0.192 / -0.191	-0.22 / -0.221	-0.223 / -0.221
	H_{C6}*	0.122 / 0.122	0.15 / 0.14	0.139 / 0.137	0.177 / 0.171
	H-Me*	0.196 / 0.196	0.381 / 0.385	0.418 / 0.419	0.442 / 0.428
	The rest atoms*	-0.148 / -0.147	0.075 / -0.041	0.066 / 0.06	0.304 / 0.265
Environment*		-1.895 / -1.894	-1.878 / -1.883	-1.9 / -1.898	-1.886 / -1.886
**Total****		-2 / -2	-2 / -2	-2 / -2	-2 / -2

**** is the summation of ***

Table S20. Group spin densities (doublet/quartet) of UB3LYP/BS1 optimised geometries for the metabolism of melatonin by CYP1A1 through *O*-demethylation in the doublet and quartet spin state (model **A**$_{III}$).

Residue group	Composition	Re_{AIII}	$TS_{HA,III}$	$IM1_{HA,III}$	$P_{Hy,III}$
Compound I	Fe	1.197 / 1.095	1.457 / 0.858	0.98 / 0.856	1.047 / 2.505
	O	0.888 / 0.917	0.294 / 0.62	0.097 / 0.169	-0.003 / 0.011
	Fe-O*	2.085 / 2.013	1.751 / 1.478	1.077 / 1.025	1.044 / 2.516
	Por*	-0.361 / 0.294	-0.246 / 0.321	-0.479 / 0.406	-0.075 / -0.007
	S	-0.709 / 0.675	-0.316 / 0.647	-0.569 / 0.568	0.033 / 0.476
	S-Me*	-0.722 / 0.687	-0.319 / 0.659	-0.578 / 0.576	0.031 / 0.491
Melatonin	C_6*	0 / 0	-0.009 / 0.012	0.022 / 0.022	0 / 0
	H_{C6}*	0 / 0	0 / -0.001	-0.001 / -0.001	0 / 0
	H-Me*	0 / -0.001	-0.026 / -0.055	-0.005 / -0.005	0 / -0.001
	The rest atoms*	-0.001 / 0.006	-0.151 / 0.584	0.91 / 0.922	0 / 0.001
Environment*		-0.001 / 0.001	0 / 0.001	0.053 / 0.055	0 / 0
**Total****		1 / 3	1 / 3	1 / 3	1 / 3

**** is the summation of ***

Table S21. Group charges (doublet/quartet) of UB3LYP/BS1 optimised geometries for the metabolism of melatonin by CYP1A1 through *O*-demethylation in the doublet and quartet spin states (model **A**$_{III}$).

Residue group	Composition	IM2$_{Hy,III}$	TS$_{A,III}$	P$_{OD,III}$
Compound I	Fe	0 / 0.413	0 / 0.412	0 / 0.187
	O	0 / -0.604	0 / -0.598	0 / -0.32
	Fe-O*	0 / -0.191	0 / -0.186	0 / -0.132
	Por*	0 / -0.667	0 / -0.555	0 / -0.485
	S	0 / 0.09	0 / 0.025	0 / 0.139
	S-Me*	0 / 0.053	0 / -0.023	0 / 0.099
Melatonin	C_6*	0 / -0.233	0 / -0.21	0 / -0.26
	H_{C6}*	0 / 0.183	0 / 0.176	0 / 0.116
	H-Me*	0 / 0.447	0 / 0.463	0 / 0.475
	The rest atoms*	0 / 0.276	0 / 0.305	0 / 0.082
Environment*		0 / -1.869	0 / -1.971	0 / -1.895
**Total****		0 / -2	0 / -2	0 / -2

**** is the summation of ***

Table S22. Group spin densities (doublet/quartet) of UB3LYP/BS1 optimised geometries for the metabolism of melatonin by CYP1A1 through *O*-demethylation in the doublet and quartet spin state (model **A**$_{III}$).

Residue group	Composition	IM2$_{Hy,III}$	TS$_{A,III}$	P$_{OD,III}$
Compound I	Fe	0 / 3.396	0 / 3.165	0 / 1.062
	O	0 / 0.009	0 / 0.009	0 / 0.007
	Fe-O*	0 / 3.406	0 / 3.174	0 / 1.069
	Por*	0 / -0.017	0 / -0.063	0 / 1.911
	S	0 / -0.379	0 / -0.113	0 / 0.022
	S-Me*	0 / -0.39	0 / -0.112	0 / 0.02
Melatonin	C_6*	0 / 0	0 / 0	0 / 0.001
	H_{C6}*	0 / 0	0 / 0	0 / 0
	H-Me*	0 / 0	0 / 0	0 / 0
	The rest atoms*	0 / 0.001	0 / 0.001	0 / -0.001
Environment*		0 / 0	0 / 0.001	0 / 0
**Total****		0 / 3	0 / 3	0 / 3

**** is the summation of ***

Table S23. Group charges (doublet/quartet) of UB3LYP/BS1 optimised geometries for the non-protein *O*-demethylation via pathways **B'**, **B''** and **B'''** in the singlet spin state.

Residue group	Composition	IM2$_{Hy}$	TS$_{Hy}$	P$_{OD}$
Water	H$_1$*	0.152 / 0.445 / 0.409	0.166 / 0.438 / 0.435	0.143 / 0.449 / 0.451
	H$_2$*	0.4 / 0.397 / 0.427	0.365 / 0.355 / 0.399	0.403 / 0.396 / 0.408
	O*	-0.792 / -0.817 / -0.819	-0.897 / -0.896 / -0.815	-0.807 / -0.819 / -0.832
Melatonin	O-Me*	-0.628 / -0.644 / -0.659	-0.59 / -0.563 / -0.641	-0.359 / -0.39 / -0.375
	H-Me*	0.418 / 0.429 / 0.441	0.429 / 0.447 / 0.428	0.432 / 0.434 / 0.433
	The rest atoms*	0.451 / 0.18 / 0.193	0.527 / 0.242 / 0.184	0.189 / -0.092 / -0.11
Environment*		0 / 0.01 / 0.007	0 / -0.024 / 0.01	0 / 0.022 / 0.025
**Total****		0 / 0 / 0	0 / 0 / 0	0 / 0 / 0

**** is the summation of ***

Table S24. Group spin densities (doublet/quartet) of UB3LYP/BS1 optimised geometries for the non-protein *O*-demethylation via pathways **B'**, **B''** and **B'''** in the singlet spin state.

Residue group	Composition	IM2$_{Hy}$	TS$_{Hy}$	P$_{OD}$
Water	H$_1$*	0 / 0 / 0	0 / 0 / 0	0 / 0 / 0
	H$_2$*	0 / 0 / 0	0 / 0 / 0	0 / 0 / 0
	O*	0 / 0 / 0	0 / 0 / 0	0 / 0 / 0
Melatonin	O-Me*	0 / 0 / 0	0 / 0 / 0	0 / 0 / 0
	H-Me*	0 / 0 / 0	0 / 0 / 0	0 / 0 / 0
	The rest atoms*	0 / 0 / 0	0 / 0 / 0	0 / 0 / 0
Environment*		0 / 0 / 0	0 / 0 / 0	0 / 0 / 0
**Total****		0 / 0 / 0	0 / 0 / 0	0 / 0 / 0

Table S25. Imaginary frequencies (in cm^{-1}) of the UB3LYP/BS1 optimised transition states for the metabolism of melatonin by CYP1A1 through aromatic hydroxylation and *O*-demethylation.

State	Imaginary frequency
$^{2,4}TS1_{C6,III}$	i428.4 (doublet), i475.9 (quartet)
$^{2,4}TS1_{HA,III}$	i1735.2 (doublet), i1200.9 (quartet)
$^{4}TS_{Hy,AIII}$	i245.2 (quartet)
$^{1}TS_{B',B'',B'''}$	i260.7 (B'), i169.6 (B''), i132.3 (B''')

Table S26. The comparison of computed bond distances r (Å) around the iron centre of CpdI and the spin densities of relating atoms from different studies. (doublet/quartet).

Calculation method	S complex	System	rFe-O	rFe-S	ρFeO	ρScys	ρPor
QM/MM	S-CH3	A	1.626/1.626	2.585/2.565	2.172/2.042	-0.349/0.282	-0.82/0.671
		B	-	2.550/2.550	-	-0.390/0.370	-
	S-H	A	1.626/1.627	2.589/2.560	2.041/2.177	-0.270/0.201	-0.915/0.763
QM	S-CH3	A	1.619/1.620	2.625/2.622	2.141/2.040	-0.777/0.719	-0.350/0.229
		C	1.629/1.629	2.607/2.619	2.100/2.030	-0.750/0.720	-0.340/0.240
		D	1.647/1.649	2.645/2.644	2.095/2.016	-0.712/0.680	0.301/-0.382
	S-H	C	1.648/1.651	2.600/2.581	2.09/02.020	-0.590/0.540	-0.500/0.440
		CYP1A1-MEL^{AII}	1.640/1.642	2.525/2.512	2.109/2.049	-0.374/0.318	-0.375/0.267
		CYP1A1-MEL^{AIII}	1.626/1.629	2.579/2.558	2.095/2.018	-0.490/0.438	-0.604/0.533
	S-CH2-CH3	CYP1A1-MEL^{AII}	1.634/1.635	2.567/2.565	2.104/2.042	-0.587/0.534	-0.210/0.115
		CYP1A1-MEL^{AIII}	1.619/1.620	2.676/2.670	2.085/2.013	-0.709/0.675	-0.361/0.294

A Schöneboom JC, Lin H, Reuter N, et al. The elusive oxidant species of cytochrome P450 enzymes: characterization by combined quantum mechanical/molecular mechanical (QM/MM) calculations. J Am Chem Soc. 2002;124(27):8142-8151. doi:10.1021/ja026279w

B Bathelt CM, Zurek J, Mulholland AJ, Harvey JN. Electronic structure of compound I in human isoforms of cytochrome P450 from QM/MM modeling. J Am Chem Soc. 2005;127(37):12900-12908. doi:10.1021/ja0520924

C Shaik S, Kumar D, de Visser SP, Altun A, Thiel W. Theoretical perspective on the structure and mechanism of cytochrome P450 enzymes. Chem Rev. 2005;105(6):2279-2328. doi:10.1021/cr030722j

D Ali HS, Henchman RH, de Visser SP. Lignin Biodegradation by a Cytochrome P450 Enzyme: A Computational Study into Syringol Activation by GcoA. Chemistry. 2020;26(57):13093-13102. doi:10.1002/chem.202002203

Cartesian Coordinates

$^{2}Re_{AIII}$

6	-20.425064000	-43.506425000	29.090094000
6	-21.052974000	-42.293168000	33.733200000
6	-16.266066000	-41.814309000	34.249487000
6	-15.649993000	-42.917351000	29.577253000
6	-21.006583000	-43.282485000	30.328524000
6	-22.422778000	-43.338789000	30.587199000
6	-22.603109000	-42.997771000	31.894526000
6	-21.292228000	-42.730428000	32.437363000
6	-19.795872000	-42.032338000	34.271573000
6	-19.569085000	-41.596165000	35.627979000
6	-18.220938000	-41.482693000	35.780580000
6	-17.628821000	-41.837657000	34.513485000
6	-15.681747000	-42.085248000	33.018329000
6	-14.262634000	-42.025760000	32.762094000
6	-14.091255000	-42.329863000	31.445668000
6	-15.405585000	-42.577275000	30.904037000
6	-16.906308000	-43.165415000	29.045064000
6	-17.135517000	-43.501713000	27.659765000
6	-18.478317000	-43.671083000	27.521804000
6	-19.066393000	-43.435355000	28.820401000
7	-20.327456000	-42.938105000	31.476028000
7	-18.603571000	-42.178154000	33.610351000
7	-16.357987000	-42.428758000	31.877359000
7	-18.091275000	-43.134911000	29.738020000
26	-18.354429000	-42.515897000	31.639650000
1	-21.917540000	-42.112159000	34.364427000
1	-15.605173000	-41.535541000	35.064658000
1	-14.807149000	-42.987604000	28.894554000
1	-21.087028000	-43.749056000	28.264531000
6	-16.283500000	-35.651055000	28.505217000
6	-18.099185000	-36.937966000	28.853524000
6	-19.295122000	-37.565168000	29.259963000
6	-17.635665000	-38.779779000	27.283808000
6	-17.283349000	-37.556387000	27.871182000
6	-17.446091000	-35.713415000	29.246035000
6	-19.636065000	-38.782939000	28.682998000
6	-18.815495000	-39.379326000	27.696758000
7	-16.183299000	-36.747466000	27.668093000
1	-15.374988000	-37.016932000	27.108211000
6	-17.998640000	-34.719383000	30.233513000
6	-16.954069000	-33.834386000	30.937836000
7	-17.586061000	-32.925196000	31.889577000
6	-17.840758000	-31.636433000	31.631442000
8	-17.503459000	-31.060077000	30.568040000
6	-18.568060000	-30.875665000	32.724486000
1	-17.970752000	-33.321529000	32.758990000
8	-20.773952000	-39.493833000	29.003416000
6	-21.346114000	-39.249798000	30.278664000
1	-15.515885000	-34.888871000	28.490399000
1	-19.918474000	-37.109717000	30.021557000
1	-16.997302000	-39.258869000	26.547122000
1	-19.121758000	-40.339226000	27.293182000
1	-18.561797000	-35.257899000	31.007040000
1	-18.727102000	-34.054750000	29.744586000
1	-16.229930000	-34.451484000	31.481032000
1	-16.409004000	-33.222990000	30.214360000
1	-17.942704000	-30.035888000	33.046459000
1	-18.822976000	-31.515194000	33.570702000
1	-19.490490000	-30.455532000	32.307872000
1	-22.106247000	-40.023087000	30.419668000
1	-21.829969000	-38.264082000	30.332960000
1	-20.588274000	-39.332436000	31.066019000
6	-19.308362000	-45.618966000	33.516742000
16	-18.361472000	-45.164918000	32.019959000
1	-19.693058000	-44.702978000	33.973871000
1	-18.576309000	-46.034786000	34.223471000
6	-16.791838000	-40.826419000	21.535024000
6	-15.267308000	-40.809514000	21.547090000
8	-14.621326000	-41.854570000	21.370428000
6	-17.412589000	-41.666083000	22.674517000
6	-18.887997000	-41.975281000	22.347253000
6	-17.245285000	-40.962618000	24.028996000
6	-19.578368000	-42.920431000	23.337625000
1	-17.194863000	-39.805437000	21.563333000
1	-16.866650000	-42.619689000	22.704074000
1	-19.449343000	-41.030173000	22.297177000
1	-18.940446000	-42.417193000	21.341497000
1	-17.588339000	-41.593261000	24.855854000
1	-16.202923000	-40.704747000	24.242831000
1	-17.833754000	-40.034978000	24.058380000
1	-20.598498000	-43.153328000	23.009643000
1	-19.031918000	-43.868997000	23.422854000
1	-19.645673000	-42.482831000	24.338989000
7	-14.691104000	-39.595655000	21.697186000
6	-13.256519000	-39.362599000	21.788543000
6	-12.885952000	-38.863579000	23.185728000
8	-13.710017000	-37.756127000	23.505329000
1	-15.249829000	-38.831789000	22.058940000
1	-12.979337000	-38.609441000	21.038778000
1	-13.035220000	-39.671065000	23.910400000
1	-11.817917000	-38.584588000	23.188542000
1	-13.960840000	-37.864667000	24.461605000
6	-12.565445000	-42.956089000	24.593026000
6	-11.993147000	-43.880774000	23.524076000
8	-11.231221000	-44.803853000	23.812788000
6	-13.533778000	-43.697093000	25.531066000
8	-13.924609000	-42.911430000	26.643465000
1	-11.727153000	-42.586133000	25.194407000
1	-14.408915000	-44.034822000	24.949807000
1	-13.037906000	-44.591065000	25.922595000
1	-14.174289000	-42.004255000	26.329330000
7	-12.384778000	-43.608664000	22.243585000
6	-11.970647000	-44.429019000	21.121035000
6	-13.152711000	-44.885281000	20.237776000
6	-14.114620000	-45.826870000	20.929101000
6	-13.888153000	-47.210044000	20.934688000
6	-15.242079000	-45.331416000	21.599599000
6	-14.759140000	-48.078388000	21.594415000
6	-16.114585000	-46.197532000	22.261766000
6	-15.876800000	-47.573531000	22.262297000
1	-13.071166000	-42.877537000	22.066817000
1	-11.437958000	-45.286453000	21.540779000
1	-13.691172000	-43.995321000	19.890702000
1	-12.729476000	-45.372712000	19.348598000
1	-13.017559000	-47.610164000	20.417585000
1	-15.424322000	-44.259802000	21.597180000
1	-14.564824000	-49.148519000	21.587509000
1	-16.982171000	-45.795514000	22.779836000
1	-16.556021000	-48.246548000	22.779738000
6	-27.314162000	-41.382688000	32.847945000
6	-27.729614000	-40.984775000	31.436519000
8	-28.545244000	-41.656288000	30.779822000
6	-27.301194000	-42.910661000	33.039897000
8	-28.588341000	-43.468395000	32.820585000
6	-26.851309000	-43.288619000	34.446431000
1	-26.330601000	-40.969988000	33.092212000
1	-26.578114000	-43.332847000	32.317026000
1	-28.880039000	-43.063724000	31.975756000
1	-26.841628000	-44.378454000	34.558134000
1	-25.848490000	-42.897755000	34.650596000
1	-27.546838000	-42.877812000	35.188263000
7	-27.170405000	-39.852169000	30.951124000
6	-27.502299000	-39.347307000	29.626197000
6	-26.621207000	-39.910899000	28.490047000
6	-25.144853000	-39.502871000	28.656924000
6	-27.201253000	-39.475762000	27.135851000
6	-24.169189000	-40.224235000	27.718398000
1	-26.508907000	-39.329799000	31.530329000
1	-27.419124000	-38.252615000	29.651181000
1	-26.688463000	-41.006088000	28.558393000
1	-25.056547000	-38.414756000	28.509648000
1	-24.842292000	-39.693787000	29.694560000
1	-26.641187000	-39.904289000	26.298291000
1	-28.246303000	-39.793350000	27.032972000
1	-27.171051000	-38.382491000	27.028288000
1	-23.133049000	-39.936689000	27.926156000
1	-24.243897000	-41.312677000	27.842480000
1	-24.370102000	-39.997175000	26.664728000
6	-26.026093000	-36.184578000	31.902083000
6	-25.143198000	-37.098303000	32.757763000
8	-25.269572000	-38.340360000	32.643387000
6	-25.633947000	-34.702852000	31.787669000
6	-24.353913000	-34.433058000	30.959686000
6	-24.644475000	-34.483848000	29.451990000
6	-23.711383000	-33.098595000	31.364408000
1	-26.064068000	-36.646937000	30.909170000
1	-26.472374000	-34.159535000	31.328678000
1	-25.531836000	-34.268915000	32.791617000
1	-23.626567000	-35.223723000	31.187604000
1	-23.722296000	-34.368437000	28.871288000
1	-25.104107000	-35.433723000	29.151619000
1	-25.328428000	-33.674890000	29.159962000
1	-22.844103000	-32.872257000	30.733408000
1	-24.424944000	-32.268559000	31.263961000
1	-23.363945000	-33.138399000	32.402780000
7	-24.309789000	-36.511128000	33.614356000
6	-23.339386000	-37.133723000	34.515287000
6	-22.085778000	-36.235186000	34.530144000
8	-22.571345000	-34.901750000	34.609063000
6	-21.201224000	-36.435408000	33.295117000
1	-24.218697000	-35.499428000	33.633941000
1	-23.120585000	-38.151472000	34.186923000
1	-21.502443000	-36.479695000	35.429260000
1	-21.816268000	-34.332123000	34.958008000
1	-20.396501000	-35.693727000	33.307165000
1	-20.758626000	-37.439948000	33.281650000
1	-21.783461000	-36.300559000	32.373647000
6	-17.330208000	-24.968695000	31.756757000
6	-15.914121000	-24.827715000	31.189727000
8	-15.667368000	-24.062541000	30.262294000
6	-18.165119000	-25.994885000	30.972001000

6	-17.694771000	-27.439329000	31.185145000
8	-16.900151000	-27.729612000	32.096059000
7	-18.183542000	-28.359187000	30.336975000
1	-17.308025000	-25.255939000	32.812419000
1	-19.215381000	-25.930058000	31.288296000
1	-18.143880000	-25.753138000	29.902049000
1	-18.829020000	-28.091328000	29.607835000
1	-17.929647000	-29.365401000	30.440406000
7	-14.975043000	-25.620311000	31.794819000
6	-13.651392000	-25.802569000	31.227384000
6	-13.426999000	-27.039165000	30.330633000
8	-12.425645000	-27.081821000	29.619012000
1	-15.348256000	-26.337031000	32.408492000
1	-13.435514000	-24.928574000	30.611086000
7	-14.341773000	-28.037435000	30.414494000
6	-14.281118000	-29.216447000	29.556942000
6	-14.931259000	-28.960268000	28.179316000
6	-14.925034000	-30.174690000	27.275949000
6	-15.809998000	-31.241504000	27.504231000
6	-14.037188000	-30.261119000	26.195339000
6	-15.811066000	-32.356235000	26.663382000
6	-14.036175000	-31.376197000	25.353193000
6	-14.926135000	-32.427173000	25.583405000
1	-15.157523000	-27.939381000	31.010293000
1	-14.800946000	-30.031101000	30.069742000
1	-15.963363000	-28.628485000	28.350603000
1	-14.396597000	-28.131379000	27.701958000
1	-16.496173000	-31.202800000	28.348395000
1	-13.345211000	-29.442020000	26.009186000
1	-16.502276000	-33.173076000	26.853486000
1	-13.344950000	-31.420173000	24.514503000
1	-14.932069000	-33.294586000	24.928364000
6	-18.549170000	-34.572128000	37.617439000
6	-18.455406000	-36.059294000	37.263169000
8	-19.388371000	-36.844370000	37.431458000
6	-18.294539000	-33.616316000	36.434025000
6	-19.394723000	-33.731592000	35.351837000
8	-20.577021000	-33.680435000	35.789661000
8	-19.036757000	-33.863085000	34.147529000
1	-19.559192000	-34.400604000	37.992859000
1	-18.307914000	-32.586997000	36.819029000
1	-17.311510000	-33.776630000	35.977730000
7	-17.239875000	-36.452056000	36.784149000
6	-16.968993000	-37.766325000	36.232722000
6	-16.249201000	-37.634687000	34.882467000
8	-15.109256000	-36.808841000	35.135218000
6	-17.144714000	-37.075810000	33.776723000
1	-16.531035000	-35.761368000	36.572433000
1	-17.918605000	-38.297275000	36.131295000
1	-15.916960000	-38.645179000	34.586397000
1	-14.815165000	-36.395506000	34.293334000
1	-16.570860000	-36.956545000	32.851251000
1	-17.969516000	-37.767597000	33.570668000
1	-17.567720000	-36.102962000	34.049497000
6	-12.868043000	-34.904866000	30.909618000
6	-13.196939000	-35.882416000	32.025839000
8	-14.114738000	-35.630146000	32.821237000
1	-12.858456000	-33.892110000	31.321704000
1	-13.666716000	-34.946267000	30.160798000
7	-12.436307000	-36.997707000	32.063178000
6	-12.628314000	-38.087152000	33.012906000
6	-11.282776000	-38.647137000	33.478366000
1	-11.773148000	-37.154739000	31.299626000
1	-13.224726000	-38.887338000	32.549379000
1	-11.437409000	-39.492520000	34.159383000
1	-10.685392000	-38.998808000	32.628825000
1	-10.703334000	-37.881523000	34.006114000
6	-9.841000000	-36.812870000	27.640784000
6	-10.882090000	-37.395727000	28.602369000
8	-10.626418000	-37.435611000	29.818505000
6	-10.288416000	-36.368410000	26.237911000
6	-11.250908000	-35.162401000	26.169202000
6	-10.618644000	-33.878429000	26.728149000
6	-11.724634000	-34.951804000	24.722668000
1	-9.361626000	-35.990684000	28.181505000
1	-9.384146000	-36.117807000	25.663549000
1	-10.739569000	-37.218826000	25.707451000
1	-12.136996000	-35.391354000	26.777943000
1	-11.323461000	-33.041866000	26.661981000
1	-10.328661000	-33.980177000	27.780156000
1	-9.719664000	-33.605145000	26.157663000
1	-12.394185000	-34.086122000	24.658721000
1	-10.869614000	-34.759711000	24.058496000
1	-12.266522000	-35.824491000	24.339340000
7	-12.003071000	-37.898631000	28.055452000
6	-13.087287000	-38.545699000	28.790073000
6	-13.667545000	-39.713086000	27.985764000
6	-14.067591000	-39.382531000	26.534325000
8	-14.013814000	-38.157435000	26.168767000
8	-14.402778000	-40.341361000	25.796500000
1	-12.239400000	-37.714778000	27.085780000
1	-13.873987000	-37.815178000	29.012791000
1	-12.959346000	-40.549459000	27.941295000
1	-14.559390000	-40.093890000	28.499006000
8	-18.528235000	-40.954291000	31.247999000
1	-13.512986000	-41.778401000	33.503143000
1	-13.173188000	-42.387359000	30.875147000
1	-20.350356000	-41.396831000	36.349960000
1	-17.665790000	-41.169819000	36.655554000
1	-23.526712000	-42.909481000	32.452034000
1	-23.172539000	-43.596672000	29.849864000
1	-19.039612000	-43.930371000	26.632753000
1	-16.341563000	-43.569677000	26.927582000
6	-20.436914000	-46.616975000	33.255646000
1	-20.054529000	-47.537964000	32.802189000
1	-20.943385000	-46.879257000	34.194630000
1	-21.180501000	-46.189373000	32.575394000
1	-16.327981000	-38.338569000	36.921333000
1	-23.774124000	-37.182611000	35.521678000
1	-27.042011000	-36.276778000	32.311169000
1	-28.043104000	-40.953808000	33.550710000
1	-28.548588000	-39.601604000	29.429714000
1	-11.261182000	-43.873830000	20.487665000
1	-13.074886000	-42.091037000	24.156142000
1	-17.077023000	-41.260530000	20.567548000
1	-12.741208000	-40.295259000	21.549108000
1	-12.684697000	-38.899590000	29.741370000
1	-9.067166000	-37.587636000	27.541078000
1	-11.912991000	-35.119474000	30.423767000
1	-13.205054000	-37.707155000	33.858489000
1	-17.833291000	-34.364279000	38.426751000
1	-13.231264000	-29.492649000	29.427767000
1	-17.803005000	-23.986125000	31.671360000
1	-12.903743000	-25.839474000	32.029156000
8	-23.927976000	-40.573656000	33.659305000
1	-24.417194000	-39.746383000	33.454194000
1	-23.333321000	-40.670448000	32.899669000

^{4}Re$_{AIII}$

6	-20.427412000	-43.491486000	29.083451000
6	-21.054871000	-42.283687000	33.729180000
6	-16.266930000	-41.823801000	34.249642000
6	-15.651090000	-42.916217000	29.575234000
6	-21.007810000	-43.268807000	30.322382000
6	-22.423509000	-43.326686000	30.582012000
6	-22.603536000	-42.986792000	31.889665000
6	-21.292988000	-42.718963000	32.432589000
6	-19.797683000	-42.026342000	34.268950000
6	-19.569796000	-41.593718000	35.626347000
6	-18.221241000	-41.486723000	35.779721000
6	-17.629924000	-41.841673000	34.512103000
6	-15.682375000	-42.092431000	33.018017000
6	-14.263031000	-42.032256000	32.762063000
6	-14.091607000	-42.332690000	31.444824000
6	-15.406151000	-42.578794000	30.902710000
6	-16.907769000	-43.159231000	29.042088000
6	-17.136319000	-43.494496000	27.656540000
6	-18.479451000	-43.659911000	27.516571000
6	-19.068547000	-43.422777000	28.814320000
7	-20.328193000	-42.923369000	31.469829000
7	-18.606138000	-42.175579000	33.607702000
7	-16.358157000	-42.433181000	31.876304000
7	-18.094239000	-43.124576000	29.733317000
26	-18.358431000	-42.510760000	31.635195000
1	-21.919828000	-42.103754000	34.360226000
1	-15.605865000	-41.549185000	35.066088000
1	-14.808306000	-42.987951000	28.892577000
1	-21.089851000	-43.733314000	28.258058000
6	-16.285769000	-35.657694000	28.509029000
6	-18.101826000	-36.943447000	28.859016000
6	-19.297572000	-37.569769000	29.266951000
6	-17.643651000	-38.783156000	27.285290000
6	-17.288375000	-37.561369000	27.874247000
6	-17.447108000	-35.719878000	29.251763000
6	-19.641072000	-38.786231000	28.688807000
6	-18.823645000	-39.381709000	27.699421000
7	-16.187988000	-36.753165000	27.670308000
1	-15.380530000	-37.022712000	27.109152000
6	-17.997720000	-34.726799000	30.241240000
6	-16.952951000	-33.837399000	30.939573000
7	-17.583897000	-32.927552000	31.891316000
6	-17.841824000	-31.639718000	31.631367000
8	-17.507955000	-31.064636000	30.566240000
6	-18.568349000	-30.878405000	32.724554000
1	-17.966473000	-33.322974000	32.762022000
8	-20.779094000	-39.496383000	29.010888000
6	-21.346155000	-39.255678000	30.289046000
1	-15.517615000	-34.896060000	28.493627000
1	-19.918617000	-37.114907000	30.030778000
1	-17.008662000	-39.261588000	26.545260000
1	-19.132322000	-40.340174000	27.294304000
1	-18.555536000	-35.266332000	31.017931000
1	-18.730630000	-34.065083000	29.754990000
1	-16.225283000	-34.451279000	31.481721000
1	-16.412181000	-33.226323000	30.212583000
1	-17.943632000	-30.037310000	33.044326000

1	-18.821127000	-31.517057000	33.572064000
1	-19.492042000	-30.460200000	32.308797000
1	-22.108210000	-40.027172000	30.429475000
1	-21.826617000	-38.268660000	30.349131000
1	-20.585520000	-39.343518000	31.073298000
6	-19.316443000	-45.611714000	33.501630000
16	-18.357644000	-45.153754000	32.013430000
1	-19.703907000	-44.697041000	33.959027000
1	-18.589870000	-46.030143000	34.212452000
6	-16.779892000	-40.831597000	21.528399000
6	-15.255513000	-40.809985000	21.543306000
8	-14.606064000	-41.853250000	21.369079000
6	-17.400127000	-41.667186000	22.671244000
6	-18.874072000	-41.982316000	22.342930000
6	-17.237229000	-40.956279000	24.022374000
6	-19.563451000	-42.924804000	23.336553000
1	-17.186040000	-39.811705000	21.550756000
1	-16.851504000	-42.619082000	22.706754000
1	-19.438107000	-41.039114000	22.287288000
1	-18.923442000	-42.429223000	21.339241000
1	-17.582658000	-41.582574000	24.851557000
1	-16.195685000	-40.697036000	24.238445000
1	-17.826269000	-40.028761000	24.044562000
1	-20.582172000	-43.162504000	23.007645000
1	-19.014192000	-43.871221000	23.427506000
1	-19.634143000	-42.482583000	24.335656000
7	-14.683120000	-39.594170000	21.693148000
6	-13.249338000	-39.357706000	21.788624000
6	-12.883223000	-38.860577000	23.187531000
8	-13.710231000	-37.755135000	23.506696000
1	-15.244788000	-38.831920000	22.053775000
1	-12.972218000	-38.602294000	21.041133000
1	-13.032857000	-39.669487000	23.910563000
1	-11.815694000	-38.579648000	23.193432000
1	-13.961385000	-37.864398000	24.462730000
6	-12.561003000	-42.958217000	24.593666000
6	-11.986301000	-43.882907000	23.525994000
8	-11.227388000	-44.807809000	23.816743000
6	-13.530673000	-43.699403000	25.530121000
8	-13.922962000	-42.913955000	26.642130000
1	-11.723980000	-42.587721000	25.196555000
1	-14.405040000	-44.036915000	24.947566000
1	-13.035490000	-44.593491000	25.922159000
1	-14.172314000	-42.006795000	26.328130000
7	-12.372140000	-43.608426000	22.244275000
6	-11.954820000	-44.427806000	21.122235000
6	-13.134419000	-44.883459000	20.235278000
6	-14.098385000	-45.825464000	20.923164000
6	-13.872503000	-47.208733000	20.927818000
6	-15.227363000	-45.330282000	21.591306000
6	-14.745524000	-48.077416000	21.584411000
6	-16.101952000	-46.196744000	22.250268000
6	-15.864703000	-47.572841000	22.249949000
1	-13.056959000	-42.876259000	22.065869000
1	-11.423191000	-45.285480000	21.542821000
1	-13.671844000	-43.993150000	19.887470000
1	-12.708836000	-45.370192000	19.346836000
1	-13.000757000	-47.608678000	20.412524000
1	-15.409118000	-44.258585000	21.589565000
1	-14.551595000	-49.147613000	21.576879000
1	-16.970760000	-45.794939000	22.766456000
1	-16.545542000	-48.246151000	22.764874000
6	-27.312522000	-41.384498000	32.857525000
6	-27.729858000	-40.984393000	31.447279000
8	-28.544661000	-41.656165000	30.789788000
6	-27.295479000	-42.912905000	33.045681000
8	-28.581474000	-43.473181000	32.826175000
6	-26.843308000	-43.293320000	34.450812000
1	-26.329878000	-40.969803000	33.102032000
1	-26.572054000	-43.331447000	32.321038000
1	-28.874905000	-43.067170000	31.982583000
1	-26.831316000	-44.383403000	34.559787000
1	-25.841034000	-42.900995000	34.654849000
1	-27.538832000	-42.885745000	35.194431000
7	-27.173183000	-39.849737000	30.963805000
6	-27.506705000	-39.343055000	29.639959000
6	-26.626364000	-39.904217000	28.502012000
6	-25.149919000	-39.496184000	28.668311000
6	-27.207697000	-39.466614000	27.149172000
6	-24.175092000	-40.215149000	27.727042000
1	-26.512763000	-39.326881000	31.543845000
1	-27.424244000	-38.248358000	29.666640000
1	-26.693274000	-40.999539000	28.568287000
1	-25.062010000	-38.407701000	28.523518000
1	-24.846264000	-39.689566000	29.705163000
1	-26.648364000	-39.893569000	26.310321000
1	-28.252823000	-39.784074000	27.046632000
1	-27.177656000	-38.373140000	27.043588000
1	-23.138668000	-39.928385000	27.934552000
1	-24.249811000	-41.303886000	27.848432000
1	-24.377059000	-39.985432000	26.674141000
6	-26.030187000	-36.182446000	31.912656000
6	-25.147500000	-37.095119000	32.769685000
8	-25.275252000	-38.337289000	32.658141000
6	-25.638633000	-34.700660000	31.797052000
6	-24.358754000	-34.431054000	30.968715000
6	-24.649679000	-34.482573000	29.461094000
6	-23.716353000	-33.096321000	31.372656000
1	-26.067312000	-36.645691000	30.920117000
1	-26.477304000	-34.158040000	31.337687000
1	-25.536636000	-34.265874000	32.800644000
1	-23.631249000	-35.221498000	31.196866000
1	-23.727691000	-34.367094000	28.880100000
1	-25.109087000	-35.432724000	29.161242000
1	-25.333984000	-33.673969000	29.168915000
1	-22.849208000	-32.870219000	30.741385000
1	-24.430027000	-32.266415000	31.271914000
1	-23.368727000	-33.135559000	32.410982000
7	-24.312479000	-36.506806000	33.623887000
6	-23.342368000	-37.128017000	34.526057000
6	-22.087760000	-36.230845000	34.538495000
8	-22.571582000	-34.896728000	34.616511000
6	-21.204809000	-36.433609000	33.302678000
1	-24.221136000	-35.495109000	33.641599000
1	-23.124612000	-38.146764000	34.200084000
1	-21.503728000	-36.475027000	35.437243000
1	-21.815487000	-34.327664000	34.964216000
1	-20.399398000	-35.692638000	33.312712000
1	-20.763084000	-37.438561000	33.290230000
1	-21.788023000	-36.299614000	32.381683000
6	-17.337665000	-24.971779000	31.750822000
6	-15.922817000	-24.828891000	31.181173000
8	-15.679083000	-24.064355000	30.252421000
6	-18.172474000	-25.999413000	30.967797000
6	-17.700042000	-27.443216000	31.180922000
8	-16.903434000	-27.732076000	32.090550000
7	-18.189178000	-28.364107000	30.334076000
1	-17.313214000	-25.258686000	32.806521000
1	-19.222382000	-25.935772000	31.285492000
1	-18.153063000	-25.758176000	29.897691000
1	-18.836666000	-28.097450000	29.606272000
1	-17.934488000	-29.370077000	30.437927000
7	-14.981328000	-25.619498000	31.785132000
6	-13.658529000	-25.800210000	31.215202000
6	-13.434128000	-27.037312000	30.319225000
8	-12.433980000	-27.079370000	29.605864000
1	-15.352226000	-26.336290000	32.400103000
1	-13.445174000	-24.926456000	30.597690000
7	-14.347302000	-28.036813000	30.405955000
6	-14.286638000	-29.216575000	29.549432000
6	-14.940892000	-28.963105000	28.173252000
6	-14.934652000	-30.178565000	27.271295000
6	-15.817617000	-31.246389000	27.502563000
6	-14.048734000	-30.265008000	26.189098000
6	-15.818688000	-32.362123000	26.663049000
6	-14.047775000	-31.381056000	25.348258000
6	-14.935805000	-32.433016000	25.581391000
1	-15.162460000	-27.938938000	31.002609000
1	-14.803555000	-30.031742000	30.064378000
1	-15.973182000	-28.633189000	28.346903000
1	-14.409100000	-28.133696000	27.693588000
1	-16.502294000	-31.207577000	28.347943000
1	-13.358350000	-29.445100000	26.000598000
1	-16.508373000	-33.179725000	26.855467000
1	-13.358152000	-31.424997000	24.508246000
1	-14.941791000	-33.301144000	24.927312000
6	-18.546847000	-34.580043000	37.618341000
6	-18.455190000	-36.066418000	37.260114000
8	-19.389474000	-36.850553000	37.425528000
6	-18.290880000	-33.621619000	36.437312000
6	-19.391843000	-33.732077000	35.355403000
8	-20.573760000	-33.678082000	35.793919000
8	-19.034643000	-33.863054000	34.150785000
1	-19.556578000	-34.407959000	37.994297000
1	-18.302120000	-32.593250000	36.824893000
1	-17.308359000	-33.782755000	35.980218000
7	-17.240098000	-36.459536000	36.780341000
6	-16.970682000	-37.772587000	36.225263000
6	-16.250814000	-37.637939000	34.875266000
8	-15.110332000	-36.813357000	35.130021000
6	-17.145975000	-37.075659000	33.771013000
1	-16.530375000	-35.769193000	36.570373000
1	-17.920851000	-38.302259000	36.122365000
1	-15.919102000	-38.647806000	34.576582000
1	-14.815837000	-36.398543000	34.289052000
1	-16.571865000	-36.953822000	32.846043000
1	-17.970836000	-37.766691000	33.562677000
1	-17.568841000	-36.103544000	34.046649000
6	-12.863184000	-34.908241000	30.907629000
6	-13.193812000	-35.885604000	32.023502000
8	-14.111385000	-35.632251000	32.818815000
1	-12.852435000	-33.895567000	31.319893000
1	-13.661527000	-34.948457000	30.158391000
7	-12.434769000	-37.001957000	32.060715000
6	-12.628155000	-38.091286000	33.010356000
6	-11.283324000	-38.651821000	33.477136000

1	-11.770966000	-37.159268000	31.297805000
1	-13.224474000	-38.891216000	32.546287000
1	-11.438989000	-39.497107000	34.158046000
1	-10.685282000	-39.003808000	32.628190000
1	-10.704013000	-37.886475000	34.005419000
6	-9.842714000	-36.815288000	27.637857000
6	-10.882525000	-37.398600000	28.600570000
8	-10.624545000	-37.440242000	29.816177000
6	-10.292331000	-36.368420000	26.236480000
6	-11.253454000	-35.161120000	26.171200000
6	-10.618769000	-33.878772000	26.731148000
6	-11.729265000	-34.947683000	24.725777000
1	-9.361521000	-35.994332000	28.178842000
1	-9.388900000	-36.118162000	25.660657000
1	-10.745602000	-37.217562000	25.705786000
1	-12.138851000	-35.389893000	26.780997000
1	-11.322520000	-33.041156000	26.666814000
1	-10.327778000	-33.982323000	27.782697000
1	-9.720061000	-33.605941000	26.160021000
1	-12.397866000	-34.081112000	24.664408000
1	-10.875106000	-34.755568000	24.060504000
1	-12.272881000	-35.819121000	24.342041000
7	-12.005059000	-37.899524000	28.055031000
6	-13.088037000	-38.547968000	28.790244000
6	-13.668287000	-39.715143000	27.985623000
6	-14.068782000	-39.383970000	26.534413000
8	-14.016929000	-38.158440000	26.169994000
8	-14.402257000	-40.342656000	25.795670000
1	-12.242462000	-37.714816000	27.085813000
1	-13.875046000	-37.818229000	29.014324000
1	-12.960031000	-40.551386000	27.940686000
1	-14.560065000	-40.096037000	28.498977000
8	-18.497911000	-40.941284000	31.257233000
1	-13.513449000	-41.786658000	33.503779000
1	-13.173550000	-42.388514000	30.874117000
1	-20.350517000	-41.392839000	36.348505000
1	-17.665055000	-41.178498000	36.655691000
1	-23.526999000	-42.899238000	32.447455000
1	-23.173393000	-43.584302000	29.844727000
1	-19.040092000	-43.917743000	26.626687000
1	-16.341619000	-43.565220000	26.925458000
6	-20.443774000	-46.608153000	33.229663000
1	-20.058907000	-47.527655000	32.775269000
1	-20.956230000	-46.873693000	34.164497000
1	-21.183002000	-46.177854000	32.546390000
1	-16.330377000	-38.347488000	36.912303000
1	-23.776937000	-37.174157000	35.532646000
1	-27.046334000	-36.274765000	32.321121000
1	-28.042134000	-40.959247000	33.561795000
1	-28.553002000	-39.597704000	29.443971000
1	-11.243743000	-43.871898000	20.491324000
1	-13.069879000	-42.093381000	24.155658000
1	-17.061931000	-41.271627000	20.562716000
1	-12.731161000	-40.288595000	21.548505000
1	-12.684192000	-38.902329000	29.740822000
1	-9.069816000	-37.590666000	27.535684000
1	-11.908165000	-35.124011000	30.422221000
1	-13.205588000	-37.710950000	33.855306000
1	-17.830562000	-34.375355000	38.428086000
1	-13.236589000	-29.490858000	29.417875000
1	-17.812017000	-23.989918000	31.665962000
1	-12.909264000	-25.835285000	32.015548000
8	-23.924146000	-40.567107000	33.670309000
1	-24.418773000	-39.743164000	33.464965000
1	-23.324083000	-40.656508000	32.914078000

^{2}TS1$_{C6,III}$

6	-20.102778000	-43.695477000	28.633857000
6	-20.635194000	-42.255451000	33.227511000
6	-15.833821000	-42.035009000	33.778340000
6	-15.292369000	-43.556252000	29.211701000
6	-20.670253000	-43.319814000	29.846414000
6	-22.091070000	-43.282678000	30.112464000
6	-22.237693000	-42.882416000	31.405613000
6	-20.902823000	-42.673686000	31.927831000
6	-19.371285000	-42.051400000	33.773129000
6	-19.132815000	-41.570481000	35.117997000
6	-17.782429000	-41.500495000	35.272515000
6	-17.198273000	-41.939574000	34.023214000
6	-15.262834000	-42.468282000	32.584201000
6	-13.840223000	-42.599603000	32.355199000
6	-13.690814000	-43.045875000	31.076661000
6	-15.022221000	-43.177156000	30.523963000
6	-16.558606000	-43.676417000	28.650483000
6	-16.791765000	-44.112756000	27.292387000
6	-18.143086000	-44.168749000	27.127068000
6	-18.735229000	-43.755205000	28.380086000
7	-19.971029000	-42.941892000	30.961876000
7	-18.184125000	-42.272343000	33.133215000
7	-15.955452000	-42.836432000	31.463980000
7	-17.753622000	-43.442485000	29.285357000
26	-17.970116000	-42.852646000	31.198151000
1	-21.486039000	-42.084005000	33.881296000
1	-15.160143000	-41.760025000	34.584993000
1	-14.450528000	-43.772044000	28.559545000
1	-20.775807000	-43.978561000	27.829574000
6	-16.022711000	-36.455945000	29.320393000
6	-17.895474000	-37.581658000	29.815190000
6	-19.185545000	-38.040621000	30.190565000
6	-17.259141000	-39.778002000	28.910885000
6	-16.967317000	-38.464480000	29.150885000
6	-17.276069000	-36.316055000	29.927816000
6	-19.509170000	-39.350601000	29.931167000
6	-18.523638000	-40.297884000	29.394069000
7	-15.824811000	-37.717045000	28.867454000
1	-15.009346000	-38.027297000	28.314849000
6	-17.871327000	-35.089536000	30.563726000
6	-16.851951000	-34.118673000	31.192941000
7	-17.532027000	-33.052309000	31.914715000
6	-17.920029000	-31.909868000	31.329386000
8	-17.640414000	-31.616614000	30.142448000
6	-18.717705000	-30.960102000	32.202536000
1	-17.924521000	-33.273422000	32.843423000
8	-20.717153000	-39.904496000	30.125834000
6	-21.744636000	-39.110378000	30.708945000
1	-15.256398000	-35.705196000	29.177364000
1	-19.889627000	-37.355175000	30.645554000
1	-16.569819000	-40.459105000	28.423284000
1	-18.935208000	-41.162486000	28.889110000
1	-18.567757000	-35.401869000	31.350813000
1	-18.467595000	-34.524907000	29.832586000
1	-16.196359000	-34.648622000	31.892060000
1	-16.229782000	-33.657875000	30.420436000
1	-18.157663000	-30.025178000	32.313664000
1	-18.948356000	-31.394786000	33.176711000
1	-19.655821000	-30.717303000	31.691473000
1	-22.615696000	-39.759065000	30.785520000
1	-21.983290000	-38.247514000	30.074317000
1	-21.452630000	-38.755983000	31.702684000
6	-17.211670000	-45.478916000	33.372307000
16	-18.013890000	-45.142811000	31.751206000
1	-16.513460000	-44.669590000	33.598465000
1	-16.614447000	-46.390622000	33.241365000
6	-17.307974000	-39.570961000	22.669100000
6	-15.809972000	-39.599488000	22.390086000
8	-15.288599000	-40.560365000	21.804591000
6	-17.790139000	-40.716295000	23.586496000
6	-19.328615000	-40.811730000	23.528544000
6	-17.266329000	-40.537953000	25.019333000
6	-19.922253000	-42.023787000	24.255406000
1	-17.608060000	-38.603214000	23.092906000
1	-17.377845000	-41.649439000	23.177766000
1	-19.759762000	-39.889132000	23.945937000
1	-19.640006000	-40.844449000	22.474167000
1	-17.512464000	-41.401371000	25.645756000
1	-16.178090000	-40.421936000	25.057451000
1	-17.713637000	-39.648336000	25.485557000
1	-21.011693000	-42.055731000	24.134629000
1	-19.515600000	-42.960347000	23.852054000
1	-19.708143000	-42.006370000	25.328686000
7	-15.121146000	-38.497370000	22.767701000
6	-13.679167000	-38.339933000	22.648839000
6	-13.022461000	-38.331868000	24.028353000
8	-13.661724000	-37.356270000	24.837783000
1	-15.553750000	-37.856659000	23.421171000
1	-13.474761000	-37.391957000	22.133604000
1	-13.120703000	-39.322123000	24.486152000
1	-11.949239000	-38.106598000	23.908436000
1	-13.769029000	-37.766308000	25.736922000
6	-12.678681000	-42.656215000	24.014743000
6	-12.414671000	-43.192526000	22.612270000
8	-11.664514000	-44.149570000	22.419061000
6	-13.365967000	-43.699850000	24.912920000
8	-13.411240000	-43.303755000	26.271062000
1	-11.708657000	-42.413851000	24.464979000
1	-14.375517000	-43.905701000	24.516154000
1	-12.800432000	-44.635871000	24.867191000
1	-13.732010000	-42.372073000	26.318164000
7	-13.058611000	-42.534259000	21.602641000
6	-12.941247000	-42.944528000	20.216260000
6	-14.308880000	-43.086729000	19.512436000
6	-15.173629000	-44.201145000	20.060443000
6	-15.073094000	-45.504702000	19.555481000
6	-16.078383000	-43.956630000	21.103376000
6	-15.850756000	-46.539624000	20.077372000
6	-16.856068000	-44.990098000	21.629348000
6	-16.745718000	-46.285147000	21.118650000
1	-13.713209000	-41.785739000	21.820093000
1	-12.396772000	-43.892489000	20.215787000
1	-14.841357000	-42.131441000	19.592293000
1	-14.113335000	-43.258138000	18.444961000
1	-14.375971000	-45.710665000	18.744912000
1	-16.164001000	-42.947088000	21.496860000
1	-15.757287000	-47.544407000	19.671509000
1	-17.549182000	-44.782649000	22.441167000
1	-17.351030000	-47.089282000	21.529657000

6	-27.005352000	-40.872711000	32.666434000
6	-27.385436000	-40.529429000	31.231230000
8	-28.346139000	-41.069726000	30.656004000
6	-27.287357000	-42.349807000	33.001994000
8	-28.668594000	-42.651660000	32.862593000
6	-26.867917000	-42.684390000	34.428429000
1	-25.952433000	-40.641093000	32.855440000
1	-26.692352000	-42.971234000	32.307852000
1	-28.906161000	-42.277679000	31.988053000
1	-27.075737000	-43.738438000	34.642518000
1	-25.799376000	-42.493667000	34.574384000
1	-27.435024000	-42.074502000	35.142034000
7	-26.621075000	-39.592365000	30.620641000
6	-26.902539000	-39.147676000	29.262584000
6	-26.357382000	-40.077405000	28.156370000
6	-24.827574000	-40.223932000	28.244422000
6	-26.824455000	-39.559683000	26.787933000
6	-24.223138000	-41.264027000	27.293648000
1	-25.865766000	-39.143337000	31.141639000
1	-26.471245000	-38.144895000	29.148300000
1	-26.810009000	-41.063865000	28.326708000
1	-24.360454000	-39.243976000	28.059413000
1	-24.565933000	-40.500632000	29.272983000
1	-26.530666000	-40.234827000	25.977443000
1	-27.916507000	-39.459827000	26.755148000
1	-26.391284000	-38.573232000	26.571075000
1	-23.151569000	-41.386107000	27.486154000
1	-24.698872000	-42.243682000	27.430878000
1	-24.341460000	-40.982131000	26.240873000
6	-25.637671000	-36.049572000	31.589738000
6	-24.776213000	-36.908844000	32.517603000
8	-24.595171000	-38.112358000	32.217803000
6	-25.687818000	-34.525193000	31.793313000
6	-24.393617000	-33.725233000	31.513862000
6	-23.886763000	-33.920269000	30.076853000
6	-24.639587000	-32.236078000	31.801669000
1	-25.311278000	-36.291952000	30.572423000
1	-26.475879000	-34.138770000	31.129921000
1	-26.035496000	-34.303861000	32.812685000
1	-23.605659000	-34.056957000	32.202179000
1	-22.996284000	-33.307260000	29.896880000
1	-23.609430000	-34.960193000	29.870755000
1	-24.650339000	-33.624216000	29.343468000
1	-23.725498000	-31.651896000	31.646298000
1	-25.418047000	-31.827174000	31.141916000
1	-24.958377000	-32.079567000	32.838883000
7	-24.301961000	-36.334612000	33.624779000
6	-23.337086000	-36.886319000	34.578563000
6	-22.106331000	-35.958326000	34.602629000
8	-22.624595000	-34.634858000	34.627364000
6	-21.185469000	-36.170908000	33.396706000
1	-24.370806000	-35.327436000	33.724267000
1	-23.088891000	-37.911917000	34.302254000
1	-21.542429000	-36.164093000	35.523418000
1	-21.887366000	-34.037293000	34.959580000
1	-20.407180000	-35.401002000	33.400916000
1	-20.706071000	-37.157825000	33.436072000
1	-21.750488000	-36.090861000	32.457762000
6	-18.116439000	-25.443903000	29.668773000
6	-16.729470000	-25.314203000	29.031751000
8	-16.575542000	-24.799132000	27.928539000
6	-18.842447000	-26.719400000	29.208746000
6	-18.216543000	-28.001071000	29.771840000
8	-17.389911000	-27.962484000	30.698959000
7	-18.609152000	-29.156231000	29.207903000
1	-18.053495000	-25.440920000	30.761222000
1	-19.890580000	-26.681911000	29.536282000
1	-18.857419000	-26.765806000	28.112719000
1	-19.281912000	-29.156977000	28.454711000
1	-18.246122000	-30.065159000	29.561956000
7	-15.704473000	-25.822036000	29.785358000
6	-14.373656000	-26.005554000	29.236045000
6	-14.015555000	-27.404488000	28.691222000
8	-13.015081000	-27.528721000	27.988363000
1	-15.990055000	-26.382895000	30.580867000
1	-14.265090000	-25.310135000	28.402393000
7	-14.815291000	-28.436846000	29.061077000
6	-14.619844000	-29.787271000	28.544519000
6	-15.318821000	-29.996088000	27.182790000
6	-15.130458000	-31.391955000	26.628301000
6	-15.873065000	-32.472835000	27.130707000
6	-14.197692000	-31.638369000	25.611849000
6	-15.684678000	-33.760566000	26.622520000
6	-14.007066000	-32.926955000	25.107558000
6	-14.748822000	-33.998404000	25.610956000
1	-15.637929000	-28.272173000	29.631981000
1	-15.021120000	-30.490595000	29.280450000
1	-16.387273000	-29.782131000	27.312878000
1	-14.919677000	-29.254541000	26.481527000
1	-16.593113000	-32.304277000	27.929669000
1	-13.616687000	-30.809077000	25.212774000
1	-16.274711000	-34.584123000	27.017791000
1	-13.277640000	-33.096558000	24.319040000

1	-14.586392000	-35.002927000	25.227542000
6	-18.718835000	-34.211812000	37.700900000
6	-18.717760000	-35.719648000	37.431398000
8	-19.718802000	-36.421000000	37.576260000
6	-18.368026000	-33.344456000	36.475154000
6	-19.445125000	-33.440600000	35.369330000
8	-20.635529000	-33.324287000	35.765132000
8	-19.058963000	-33.624104000	34.178199000
1	-19.725086000	-33.953075000	38.033782000
1	-18.314069000	-32.296525000	36.802320000
1	-17.389018000	-33.601603000	36.056854000
7	-17.509707000	-36.230618000	37.055447000
6	-17.306871000	-37.600793000	36.619961000
6	-16.532050000	-37.632122000	35.295953000
8	-15.343119000	-36.871516000	35.529786000
6	-17.339755000	-37.100505000	34.112215000
1	-16.735521000	-35.610186000	36.855996000
1	-18.286579000	-38.073958000	36.522381000
1	-16.268348000	-38.683501000	35.091818000
1	-14.963084000	-36.597225000	34.667067000
1	-16.720716000	-37.097219000	33.208251000
1	-18.202675000	-37.749112000	33.923118000
1	-17.700668000	-36.081980000	34.292710000
6	-12.412526000	-35.540285000	31.544034000
6	-13.070768000	-36.455083000	32.565451000
8	-14.098263000	-36.086175000	33.154835000
1	-12.348224000	-34.534033000	31.967575000
1	-13.048737000	-35.478420000	30.653976000
7	-12.461924000	-37.643100000	32.761086000
6	-12.959625000	-38.672188000	33.670155000
6	-11.797743000	-39.443803000	34.296902000
1	-11.675604000	-37.895378000	32.158006000
1	-13.620655000	-39.364128000	33.128835000
1	-12.179897000	-40.235520000	34.951395000
1	-11.170085000	-39.909648000	33.528046000
1	-11.163855000	-38.779730000	34.895195000
6	-9.274528000	-37.550401000	28.885018000
6	-10.382750000	-38.235617000	29.690436000
8	-10.258718000	-38.365904000	30.919559000
6	-9.647999000	-36.873828000	27.555809000
6	-10.553395000	-35.628184000	27.661039000
6	-9.844468000	-34.460120000	28.364346000
6	-11.042592000	-35.207587000	26.267171000
1	-8.795933000	-36.843169000	29.568683000
1	-8.715355000	-36.581618000	27.051165000
1	-10.116542000	-37.607913000	26.885263000
1	-11.437959000	-35.896057000	28.257676000
1	-10.507911000	-33.590648000	28.434645000
1	-9.526597000	-34.714267000	29.382080000
1	-8.952324000	-34.152057000	27.801644000
1	-11.641015000	-34.291611000	26.321171000
1	-10.190889000	-35.011740000	25.600145000
1	-11.671737000	-35.979191000	25.808581000
7	-11.415894000	-38.749435000	28.992966000
6	-12.491376000	-39.544662000	29.581273000
6	-13.070470000	-40.512638000	28.548269000
6	-13.638570000	-39.852506000	27.280315000
8	-13.657762000	-38.566450000	27.260787000
8	-14.040063000	-40.592542000	26.356855000
1	-11.610781000	-38.421273000	28.054793000
1	-13.279080000	-38.890255000	29.974435000
1	-12.317278000	-41.244133000	28.229595000
1	-13.870804000	-41.096266000	29.016124000
8	-17.856682000	-41.062255000	30.908454000
1	-13.073314000	-42.383862000	33.089279000
1	-12.777506000	-43.269203000	30.539329000
1	-19.908656000	-41.320121000	35.830873000
1	-17.218460000	-41.185708000	36.141840000
1	-23.148657000	-42.722292000	31.969595000
1	-22.860064000	-43.538999000	29.394121000
1	-18.704464000	-44.466589000	26.250029000
1	-16.000404000	-44.333868000	26.588043000
6	-18.199853000	-45.669756000	34.525756000
1	-18.900516000	-46.484434000	34.308573000
1	-17.669682000	-45.914228000	35.459004000
1	-18.785779000	-44.760899000	34.691975000
1	-16.733045000	-38.154103000	37.378228000
1	-23.808335000	-36.900472000	35.568095000
1	-26.654610000	-36.457817000	31.671339000
1	-27.612171000	-40.247439000	33.337335000
1	-27.989167000	-39.063220000	29.150677000
1	-12.338180000	-42.214310000	19.654027000
1	-13.277864000	-41.739838000	23.995846000
1	-17.798733000	-39.660520000	21.690859000
1	-13.296269000	-39.157520000	22.034203000
1	-12.075361000	-40.096302000	30.427688000
1	-8.525250000	-38.331496000	28.690689000
1	-11.417936000	-35.880789000	31.246083000
1	-13.560567000	-38.184305000	34.440456000
1	-18.012525000	-34.005104000	38.518535000
1	-13.545373000	-29.959095000	28.440226000
1	-18.693332000	-24.571595000	29.348888000
1	-13.619272000	-25.745201000	29.988605000

8	-23.567135000	-40.394322000	33.513471000
1	-23.902306000	-39.557326000	33.128659000
1	-22.746453000	-40.570805000	33.029933000

^{4}TS1$_{C6,III}$

6	-20.033503000	-43.801842000	28.875420000
6	-20.542156000	-42.077878000	33.377750000
6	-15.724404000	-42.090006000	33.896044000
6	-15.219313000	-43.459690000	29.275029000
6	-20.587741000	-43.383768000	30.079731000
6	-22.006557000	-43.298657000	30.335142000
6	-22.154159000	-42.798164000	31.594063000
6	-20.821923000	-42.581195000	32.110572000
6	-19.265531000	-41.914057000	33.900412000
6	-18.989128000	-41.450760000	35.239701000
6	-17.636545000	-41.478952000	35.393238000
6	-17.082177000	-41.946383000	34.143956000
6	-15.169597000	-42.484261000	32.684473000
6	-13.750699000	-42.594533000	32.434146000
6	-13.610166000	-42.968761000	31.132112000
6	-14.942587000	-43.091508000	30.586384000
6	-16.496352000	-43.639142000	28.756562000
6	-16.763484000	-44.096517000	27.412112000
6	-18.115626000	-44.226720000	27.307288000
6	-18.675111000	-43.831144000	28.581382000
7	-19.890223000	-42.956472000	31.180678000
7	-18.091282000	-42.206346000	33.252888000
7	-15.869526000	-42.797579000	31.552435000
7	-17.670764000	-43.472391000	29.439384000
26	-17.881561000	-42.796806000	31.346448000
1	-21.381679000	-41.825535000	34.018754000
1	-15.041640000	-41.856847000	34.707445000
1	-14.386788000	-43.636390000	28.600281000
1	-20.720126000	-44.111335000	28.093094000
6	-15.989818000	-36.312921000	29.305023000
6	-17.808273000	-37.509718000	29.848329000
6	-19.080909000	-38.015555000	30.237437000
6	-17.166155000	-39.674095000	28.857253000
6	-16.890487000	-38.356296000	29.136595000
6	-17.219186000	-36.224554000	29.957995000
6	-19.383523000	-39.321597000	29.957719000
6	-18.386338000	-40.261875000	29.401448000
7	-15.786848000	-37.575030000	28.817312000
1	-14.980268000	-37.867403000	28.254233000
6	-17.834364000	-35.024636000	30.624732000
6	-16.828814000	-34.046580000	31.263062000
7	-17.520073000	-32.990717000	31.991519000
6	-17.918317000	-31.848122000	31.414750000
8	-17.640972000	-31.543054000	30.230016000
6	-18.726621000	-30.912653000	32.294129000
1	-17.905192000	-33.219286000	32.919801000
8	-20.591523000	-39.906771000	30.162742000
6	-21.593154000	-39.139815000	30.810629000
1	-15.244258000	-35.545499000	29.146742000
1	-19.799471000	-37.353090000	30.705223000
1	-16.482378000	-40.310140000	28.305179000
1	-18.825223000	-41.069863000	28.821718000
1	-18.517589000	-35.365211000	31.411943000
1	-18.448535000	-34.455729000	29.911530000
1	-16.169803000	-34.573626000	31.961003000
1	-16.209301000	-33.574298000	30.495570000
1	-18.181707000	-29.968512000	32.402688000
1	-18.943733000	-31.351599000	33.269592000
1	-19.672550000	-30.685809000	31.789975000
1	-22.461315000	-39.792468000	30.902064000
1	-21.865648000	-38.255366000	30.219119000
1	-21.260154000	-38.813734000	31.803142000
6	-19.132951000	-45.584939000	33.093129000
16	-17.747461000	-45.126796000	31.972598000
1	-19.581318000	-44.675450000	33.499087000
1	-18.679798000	-46.121628000	33.936648000
6	-17.308093000	-39.604387000	22.720019000
6	-15.815388000	-39.620287000	22.411318000
8	-15.295352000	-40.584699000	21.829425000
6	-17.763748000	-40.757356000	23.640954000
6	-19.300851000	-40.879052000	23.598198000
6	-17.229155000	-40.569767000	25.068310000
6	-19.865091000	-42.101261000	24.331743000
1	-17.607471000	-38.640717000	23.153195000
1	-17.340477000	-41.683473000	23.227401000
1	-19.744144000	-39.964490000	24.020048000
1	-19.622395000	-40.918322000	22.547059000
1	-17.453612000	-41.437025000	25.697646000
1	-16.142970000	-40.433276000	25.094892000
1	-17.686605000	-39.689877000	25.541888000
1	-20.954604000	-42.155295000	24.219374000
1	-19.442653000	-43.030501000	23.927148000
1	-19.643601000	-42.076451000	25.403422000
7	-15.132300000	-38.503869000	22.753821000
6	-13.696538000	-38.328071000	22.591745000
6	-13.003172000	-38.277358000	23.952604000
8	-13.631385000	-37.289069000	24.753974000
1	-15.555787000	-37.856879000	23.407330000
1	-13.520892000	-37.389169000	22.049879000
1	-13.078052000	-39.257057000	24.437300000
1	-11.936072000	-38.044049000	23.797652000
1	-13.720164000	-37.685201000	25.661822000
6	-12.685701000	-42.642674000	24.070782000
6	-12.423922000	-43.203982000	22.677801000
8	-11.680085000	-44.169301000	22.501244000
6	-13.421168000	-43.651282000	24.970459000
8	-13.478396000	-43.234552000	26.322355000
1	-11.713256000	-42.429399000	24.529897000
1	-14.429910000	-43.829924000	24.558336000
1	-12.885715000	-44.605907000	24.947486000
1	-13.751435000	-42.285534000	26.350102000
7	-13.064063000	-42.559370000	21.657090000
6	-12.944024000	-42.990690000	20.277423000
6	-14.309300000	-43.136863000	19.570021000
6	-15.180874000	-44.240576000	20.128676000
6	-15.078874000	-45.552497000	19.646098000
6	-16.094377000	-43.976847000	21.159182000
6	-15.863797000	-46.576964000	20.177647000
6	-16.879659000	-44.999769000	21.694430000
6	-16.767896000	-46.303288000	21.206114000
1	-13.714826000	-41.803672000	21.861616000
1	-12.403806000	-43.940981000	20.292870000
1	-14.838362000	-42.178635000	19.635271000
1	-14.109924000	-43.323012000	18.505709000
1	-14.374899000	-45.773313000	18.845429000
1	-16.180673000	-42.960739000	21.535222000
1	-15.769123000	-47.588464000	19.789086000
1	-17.580222000	-44.777109000	22.495846000
1	-17.379360000	-47.099168000	21.624155000
6	-27.042275000	-41.108432000	32.741897000
6	-27.409695000	-40.792385000	31.296723000
8	-28.251879000	-41.459270000	30.671002000
6	-27.130729000	-42.616275000	33.044046000
8	-28.452527000	-43.099639000	32.851700000
6	-26.715612000	-42.920570000	34.478555000
1	-26.038292000	-40.739558000	32.973926000
1	-26.434441000	-43.136968000	32.361368000
1	-28.706214000	-42.751671000	31.970885000
1	-26.787207000	-43.996965000	34.668989000
1	-25.686779000	-42.592790000	34.662402000
1	-27.381001000	-42.406159000	35.182499000
7	-26.771463000	-39.735217000	30.741079000
6	-27.026156000	-39.324196000	29.367822000
6	-26.181468000	-40.072483000	28.313098000
6	-24.674864000	-39.868656000	28.555099000
6	-26.618939000	-39.638329000	26.906940000
6	-23.752966000	-40.726229000	27.679850000
1	-26.090297000	-39.212528000	31.295333000
1	-26.831066000	-38.245728000	29.299267000
1	-26.408607000	-41.141336000	28.432481000
1	-24.428870000	-38.804153000	28.413154000
1	-24.464318000	-40.093155000	29.607410000
1	-26.090957000	-40.199095000	26.128592000
1	-27.694176000	-39.799192000	26.759913000
1	-26.414432000	-38.571184000	26.742800000
1	-22.708443000	-40.613740000	27.990697000
1	-24.009674000	-41.789786000	27.766722000
1	-23.817213000	-40.454101000	26.619860000
6	-25.668647000	-36.056520000	31.560407000
6	-24.847578000	-36.919382000	32.524081000
8	-24.850304000	-38.163948000	32.377175000
6	-25.281331000	-34.575169000	31.421594000
6	-23.916640000	-34.326415000	30.736141000
6	-24.025033000	-34.457453000	29.209015000
6	-23.339155000	-32.963836000	31.144958000
1	-25.616286000	-36.558685000	30.588280000
1	-26.066516000	-34.066205000	30.844525000
1	-25.296857000	-34.092775000	32.408195000
1	-23.213693000	-35.092839000	31.089804000
1	-23.040950000	-34.360618000	28.736222000
1	-24.440961000	-35.426325000	28.905953000
1	-24.673106000	-33.672604000	28.795273000
1	-22.404928000	-32.755183000	30.610374000
1	-24.043744000	-32.151198000	30.918703000
1	-23.117460000	-32.947509000	32.217777000
7	-24.207547000	-36.285047000	33.506480000
6	-23.322571000	-36.844999000	34.528480000
6	-22.085155000	-35.930605000	34.624398000
8	-22.587402000	-34.600220000	34.605906000
6	-21.091559000	-36.166886000	33.482314000
1	-24.171187000	-35.270204000	33.517931000
1	-23.065606000	-37.875365000	34.276752000
1	-21.582690000	-36.133991000	35.580316000
1	-21.859092000	-34.008052000	34.968403000
1	-20.307121000	-35.404774000	33.524082000
1	-20.626306000	-37.157899000	33.563288000
1	-21.593832000	-36.093758000	32.508031000
6	-18.207752000	-25.383342000	29.750140000
6	-16.834662000	-25.230370000	29.088583000
8	-16.707316000	-24.701733000	27.988278000
6	-18.921412000	-26.670967000	29.304327000

6	-18.266651000	-27.942529000	29.858105000
8	-17.424787000	-27.890097000	30.771007000
7	-18.650610000	-29.103907000	29.301962000
1	-18.124721000	-25.379141000	30.841292000
1	-19.964084000	-26.649105000	29.650231000
1	-18.955220000	-26.719505000	28.208794000
1	-19.338495000	-29.115852000	28.562610000
1	-18.271087000	-30.007786000	29.654237000
7	-15.790477000	-25.734351000	29.817804000
6	-14.468330000	-25.899096000	29.242365000
6	-14.101928000	-27.292268000	28.688208000
8	-13.113893000	-27.400909000	27.965247000
1	-16.056027000	-26.305750000	30.612980000
1	-14.385719000	-25.200999000	28.407944000
7	-14.879703000	-28.336417000	29.070913000
6	-14.674528000	-29.683305000	28.547908000
6	-15.386140000	-29.895975000	27.193360000
6	-15.181486000	-31.285306000	26.628064000
6	-15.892175000	-32.383245000	27.139403000
6	-14.264295000	-31.508337000	25.592162000
6	-15.689630000	-33.664701000	26.621003000
6	-14.058873000	-32.790465000	25.076996000
6	-14.769980000	-33.878317000	25.589232000
1	-15.691460000	-28.185331000	29.660741000
1	-15.059410000	-30.392893000	29.286544000
1	-16.456342000	-29.700061000	27.337489000
1	-15.007346000	-29.144089000	26.491823000
1	-16.599072000	-32.233250000	27.953822000
1	-13.707103000	-30.665910000	25.186609000
1	-16.251697000	-34.501959000	27.027123000
1	-13.341869000	-32.941768000	24.273413000
1	-14.597211000	-34.877810000	25.197004000
6	-18.773326000	-34.254854000	37.796325000
6	-18.748349000	-35.755313000	37.490235000
8	-19.744894000	-36.471027000	37.592051000
6	-18.393264000	-33.352068000	36.605321000
6	-19.437195000	-33.423944000	35.465902000
8	-20.639249000	-33.312179000	35.829618000
8	-19.015974000	-33.586925000	34.285091000
1	-19.791798000	-34.013580000	38.104459000
1	-18.356089000	-32.313481000	36.963015000
1	-17.401200000	-33.591825000	36.208021000
7	-17.525295000	-36.245215000	37.135573000
6	-17.296360000	-37.603049000	36.675692000
6	-16.471674000	-37.597876000	35.381614000
8	-15.294466000	-36.842342000	35.679033000
6	-17.237132000	-37.037518000	34.182957000
1	-16.752055000	-35.613191000	36.971749000
1	-18.268235000	-38.079733000	36.528734000
1	-16.197702000	-38.643895000	35.161670000
1	-14.888072000	-36.540437000	34.837322000
1	-16.586546000	-37.006277000	33.302395000
1	-18.090912000	-37.682048000	33.944259000
1	-17.608841000	-36.025502000	34.377333000
6	-12.479232000	-35.459283000	31.599659000
6	-13.042547000	-36.362916000	32.684654000
8	-14.020872000	-35.991947000	33.350460000
1	-12.414007000	-34.440600000	31.991732000
1	-13.178704000	-35.441077000	30.756380000
7	-12.413087000	-37.546641000	32.842952000
6	-12.831370000	-38.571055000	33.794274000
6	-11.616602000	-39.265822000	34.411810000
1	-11.662480000	-37.793272000	32.193432000
1	-13.469299000	-39.311284000	33.289717000
1	-11.940658000	-40.060354000	35.094088000
1	-10.983042000	-39.716141000	33.638483000
1	-11.005261000	-38.554377000	34.978124000
6	-9.297871000	-37.404184000	28.897463000
6	-10.403221000	-38.098410000	29.699430000
8	-10.273975000	-38.239181000	30.927369000
6	-9.667570000	-36.745757000	27.557998000
6	-10.588670000	-35.509664000	27.640013000
6	-9.903280000	-34.327963000	28.344099000
6	-11.061253000	-35.105792000	26.235348000
1	-8.835895000	-36.683680000	29.578863000
1	-8.733653000	-36.447372000	27.059175000
1	-10.121055000	-37.492557000	26.891367000
1	-11.479029000	-35.781923000	28.225622000
1	-10.575936000	-33.464154000	28.393410000
1	-9.602010000	-34.568874000	29.370136000
1	-9.003719000	-34.017299000	27.794671000
1	-11.673133000	-34.197879000	26.273441000
1	-10.201543000	-34.902569000	25.580769000
1	-11.671696000	-35.889157000	25.771493000
7	-11.438166000	-38.603803000	29.000617000
6	-12.521331000	-39.392489000	29.582793000
6	-13.078440000	-40.379438000	28.554375000
6	-13.593768000	-39.745409000	27.249389000
8	-13.575413000	-38.463616000	27.186206000
8	-13.991101000	-40.508770000	26.341121000
1	-11.635089000	-38.275586000	28.062004000
1	-13.319146000	-38.733700000	29.946877000
1	-12.323749000	-41.127367000	28.280142000
1	-13.904508000	-40.938107000	29.007744000
8	-17.868363000	-41.117649000	30.775529000
1	-12.979117000	-42.406152000	33.170453000
1	-12.701085000	-43.153922000	30.574075000
1	-19.744565000	-41.149125000	35.954330000
1	-17.051120000	-41.204476000	36.261739000
1	-23.064915000	-42.574973000	32.136365000
1	-22.776640000	-43.582146000	29.628557000
1	-18.698747000	-44.554793000	26.455760000
1	-15.990512000	-44.278531000	26.677175000
6	-20.197966000	-46.459447000	32.428449000
1	-19.755335000	-47.378284000	32.026838000
1	-20.976058000	-46.741269000	33.152942000
1	-20.677616000	-45.928725000	31.600534000
1	-16.748262000	-38.171533000	37.442039000
1	-23.856161000	-36.848945000	35.486945000
1	-26.715093000	-36.143235000	31.885626000
1	-27.752566000	-40.584427000	33.397512000
1	-28.088370000	-39.489007000	29.160463000
1	-12.335584000	-42.271534000	19.706761000
1	-13.252234000	-41.706502000	24.035100000
1	-17.816191000	-39.693264000	21.750327000
1	-13.318399000	-39.153846000	21.985101000
1	-12.119720000	-39.927588000	30.446689000
1	-8.534950000	-38.175798000	28.718763000
1	-11.498784000	-35.783043000	31.242027000
1	-13.438632000	-38.091853000	34.565022000
1	-18.094995000	-34.063245000	38.640975000
1	-13.599419000	-29.842803000	28.430894000
1	-18.804537000	-24.520647000	29.440916000
1	-13.702569000	-25.629609000	29.980177000
8	-23.591588000	-40.361690000	33.605161000
1	-24.038477000	-39.552653000	33.274903000
1	-22.806639000	-40.438077000	33.043337000

^{2}IM1$_{C6,III}$

6	-19.435993000	-44.645431000	27.366442000
6	-19.541158000	-44.693797000	32.206785000
6	-14.783255000	-43.828481000	32.375020000
6	-14.631050000	-44.022660000	27.539119000
6	-19.887065000	-44.718418000	28.679748000
6	-21.256685000	-44.973888000	29.064993000
6	-21.279050000	-45.005414000	30.426166000
6	-19.924078000	-44.761876000	30.871370000
6	-18.254604000	-44.445722000	32.673605000
6	-17.914366000	-44.273444000	34.071308000
6	-16.581892000	-44.001477000	34.120762000
6	-16.106219000	-44.025646000	32.753632000
6	-14.314935000	-43.855027000	31.063564000
6	-12.929709000	-43.683820000	30.677757000
6	-12.888783000	-43.757830000	29.318026000
6	-14.253735000	-43.962793000	28.877204000
6	-15.934182000	-44.174669000	27.073724000
6	-16.287911000	-44.268461000	25.675182000
6	-17.639485000	-44.439117000	25.621761000
6	-18.110415000	-44.450227000	26.987551000
7	-19.100107000	-44.586950000	29.794041000
7	-17.139150000	-44.296602000	31.897401000
7	-15.093947000	-44.037363000	29.954811000
7	-17.060385000	-44.275694000	27.850791000
26	-17.092790000	-44.315341000	29.881569000
1	-20.320252000	-44.826444000	32.953011000
1	-14.058339000	-43.649567000	33.164146000
1	-13.836583000	-43.925529000	26.803713000
1	-20.169466000	-44.775273000	26.575446000
6	-16.418170000	-37.261908000	28.912571000
6	-17.879033000	-38.740144000	29.742966000
6	-18.894886000	-39.455239000	30.435073000
6	-17.122232000	-40.716658000	28.491306000
6	-17.019546000	-39.397433000	28.775679000
6	-17.482986000	-37.392709000	29.816735000
6	-19.019293000	-40.795558000	30.178057000
6	-18.079538000	-41.583198000	29.267211000
7	-16.137386000	-38.421572000	28.289911000
1	-15.300859000	-38.553150000	27.696139000
6	-18.052640000	-36.345209000	30.728918000
6	-17.502451000	-34.934794000	30.473610000
7	-18.007496000	-33.988941000	31.451398000
6	-19.011336000	-33.140199000	31.220069000
8	-19.593049000	-33.033394000	30.108854000
6	-19.429614000	-32.283988000	32.401844000
1	-17.643973000	-34.116725000	32.425671000
8	-19.944861000	-41.593592000	30.707721000
6	-20.850922000	-41.059653000	31.670872000
1	-15.833207000	-36.379864000	28.686236000
1	-19.515815000	-38.935551000	31.157595000
1	-16.459499000	-41.233967000	27.804236000
1	-18.712173000	-42.153140000	28.564479000
1	-17.858417000	-36.612839000	31.775394000
1	-19.145390000	-36.315600000	30.635306000
1	-16.407996000	-34.938128000	30.554609000
1	-17.767650000	-34.581289000	29.473730000
1	-19.430284000	-31.231749000	32.098188000

1	-18.775577000	-32.429989000	33.264961000
1	-20.455738000	-32.544423000	32.688096000
1	-21.491616000	-41.893324000	31.958618000
1	-21.451080000	-40.251856000	31.235705000
1	-20.308125000	-40.681151000	32.542715000
6	-15.689632000	-47.233318000	31.024963000
16	-16.859354000	-46.621403000	29.741597000
1	-15.048238000	-46.408461000	31.343774000
1	-15.043298000	-47.972903000	30.534925000
6	-15.113283000	-39.638786000	21.301955000
6	-13.682643000	-39.214614000	21.609294000
8	-12.720124000	-39.905503000	21.244747000
6	-15.575294000	-40.911221000	22.049126000
6	-16.886764000	-41.413013000	21.411521000
6	-15.712395000	-40.658111000	23.558672000
6	-17.361419000	-42.786489000	21.901554000
1	-15.812720000	-38.817364000	21.508395000
1	-14.804140000	-41.679058000	21.893234000
1	-17.677081000	-40.668914000	21.593776000
1	-16.753514000	-41.455398000	20.320825000
1	-16.030631000	-41.561461000	24.089211000
1	-14.771012000	-40.338693000	24.018330000
1	-16.466152000	-39.881008000	23.750966000
1	-18.269069000	-43.097186000	21.369903000
1	-16.596510000	-43.553970000	21.726081000
1	-17.588611000	-42.784454000	22.972270000
7	-13.550654000	-38.026256000	22.244029000
6	-12.289728000	-37.466688000	22.707334000
6	-12.210240000	-37.521909000	24.232588000
8	-13.394271000	-36.957866000	24.778581000
1	-14.362936000	-37.637387000	22.706285000
1	-12.230752000	-36.424815000	22.365711000
1	-12.110758000	-38.564507000	24.552401000
1	-11.315079000	-36.967709000	24.562312000
1	-13.638866000	-37.539709000	25.545475000
6	-10.533248000	-41.719640000	24.048111000
6	-9.739334000	-42.029269000	22.783715000
8	-8.792261000	-42.815773000	22.789381000
6	-11.099593000	-42.989953000	24.704540000
8	-11.627897000	-42.746345000	25.995382000
1	-9.848188000	-41.247992000	24.764088000
1	-11.858750000	-43.431417000	24.035630000
1	-10.294797000	-43.723462000	24.811705000
1	-12.181386000	-41.930394000	25.955938000
7	-10.143581000	-41.357860000	21.663885000
6	-9.506404000	-41.549192000	20.375188000
6	-10.515996000	-41.852525000	19.245882000
6	-11.225001000	-43.181328000	19.394769000
6	-10.662710000	-44.359168000	18.883932000
6	-12.449494000	-43.264753000	20.072581000
6	-11.301093000	-45.589514000	19.047044000
6	-13.089569000	-44.494361000	20.239745000
6	-12.518470000	-45.661197000	19.727561000
1	-10.978172000	-40.777502000	21.697831000
1	-8.794681000	-42.369871000	20.497916000
1	-11.253817000	-41.041874000	19.213057000
1	-9.966789000	-41.827024000	18.294605000
1	-9.712467000	-44.311110000	18.354661000
1	-12.889370000	-42.354082000	20.470908000
1	-10.847387000	-46.492197000	18.644128000
1	-14.036144000	-44.540961000	20.773124000
1	-13.016668000	-46.618443000	19.859696000
6	-27.709933000	-32.912769000	35.177912000
6	-28.377575000	-33.662845000	34.033231000
8	-29.614342000	-33.755638000	33.942646000
6	-28.514918000	-33.024902000	36.486647000
8	-29.813263000	-32.468086000	36.342752000
6	-27.810184000	-32.310842000	37.633667000
1	-26.689709000	-33.274662000	35.342765000
1	-28.590818000	-34.098950000	36.740075000
1	-30.147622000	-32.859855000	35.508388000
1	-28.398267000	-32.406199000	38.553212000
1	-26.814018000	-32.735708000	37.796816000
1	-27.706011000	-31.242786000	37.407383000
7	-27.548744000	-34.210037000	33.111637000
6	-28.070148000	-34.939135000	31.964410000
6	-28.357386000	-36.431010000	32.241643000
6	-27.068274000	-37.199847000	32.591067000
6	-29.097166000	-37.043313000	31.042900000
6	-27.297657000	-38.599596000	33.173478000
1	-26.536820000	-34.153709000	33.236398000
1	-27.344648000	-34.845893000	31.145529000
1	-29.027432000	-36.460019000	33.112380000
1	-26.444640000	-37.280464000	31.686589000
1	-26.485233000	-36.610259000	33.309159000
1	-29.365327000	-38.089767000	31.223570000
1	-30.023695000	-36.496526000	30.829920000
1	-28.474583000	-37.013273000	30.138101000
1	-26.344574000	-39.076946000	33.428458000
1	-27.900390000	-38.550077000	34.089207000
1	-27.817047000	-39.260237000	32.469513000
6	-24.006699000	-34.287165000	31.144377000
6	-23.694290000	-34.587734000	32.610977000
8	-24.596954000	-34.388437000	33.455314000
6	-22.927244000	-34.459788000	30.061253000
6	-22.543296000	-35.900291000	29.645976000
6	-23.756107000	-36.728966000	29.196192000
6	-21.488581000	-35.835450000	28.529326000
1	-24.890756000	-34.888669000	30.899635000
1	-23.304066000	-33.953685000	29.159010000
1	-22.016213000	-33.914315000	30.342175000
1	-22.084577000	-36.418451000	30.499718000
1	-23.437443000	-37.716235000	28.842113000
1	-24.477808000	-36.891782000	30.005441000
1	-24.283443000	-36.234767000	28.368337000
1	-21.113072000	-36.835124000	28.282225000
1	-21.917534000	-35.404543000	27.613818000
1	-20.638389000	-35.211110000	28.825291000
7	-22.469135000	-35.021596000	32.925225000
6	-22.014174000	-35.513211000	34.227377000
6	-21.467661000	-36.947321000	34.059163000
8	-20.561759000	-36.956944000	32.953658000
6	-22.566003000	-37.973332000	33.798260000
1	-21.818193000	-35.250585000	32.182489000
1	-22.830624000	-35.451125000	34.947741000
1	-20.938868000	-37.200709000	34.985107000
1	-19.707586000	-36.594325000	33.311188000
1	-22.124301000	-38.967636000	33.677892000
1	-23.270952000	-38.009110000	34.636991000
1	-23.124797000	-37.736690000	32.884325000
6	-22.149735000	-27.312094000	29.815296000
6	-21.191850000	-26.848736000	28.714780000
8	-21.598177000	-26.517895000	27.605238000
6	-22.505724000	-28.801508000	29.680269000
6	-21.321409000	-29.731376000	29.969086000
8	-20.298978000	-29.316324000	30.541979000
7	-21.454590000	-31.006046000	29.566791000
1	-21.729317000	-27.131657000	30.809208000
1	-23.307696000	-29.047698000	30.389933000
1	-22.899762000	-29.003690000	28.676804000
1	-22.308885000	-31.315412000	29.126028000
1	-20.733426000	-31.718180000	29.794515000
7	-19.869452000	-26.846505000	29.072841000
6	-18.811573000	-26.658521000	28.098029000
6	-18.087425000	-27.919086000	27.584451000
8	-17.315260000	-27.812934000	26.633285000
1	-19.653013000	-27.311831000	29.946820000
1	-19.254076000	-26.169252000	27.228527000
7	-18.313009000	-29.081089000	28.245839000
6	-17.696644000	-30.330720000	27.814142000
6	-18.362086000	-30.928257000	26.554582000
6	-17.648281000	-32.175173000	26.080010000
6	-17.917276000	-33.427354000	26.652958000
6	-16.662768000	-32.094706000	25.085992000
6	-17.216923000	-34.564601000	26.241832000
6	-15.956106000	-33.228712000	24.680911000
6	-16.227366000	-34.471071000	25.258661000
1	-18.971777000	-29.120813000	29.017130000
1	-17.771734000	-31.039748000	28.643750000
1	-19.411562000	-31.148862000	26.787059000
1	-18.347085000	-30.162682000	25.772130000
1	-18.669158000	-33.504588000	27.436260000
1	-16.444410000	-31.129291000	24.633857000
1	-17.442648000	-35.527271000	26.693405000
1	-15.187658000	-33.142712000	23.916487000
1	-15.653033000	-35.345085000	24.961876000
6	-18.121901000	-35.783641000	37.152158000
6	-18.664221000	-37.211869000	37.085663000
8	-19.870029000	-37.440457000	37.209469000
6	-17.051700000	-35.362031000	36.134322000
6	-17.549028000	-35.288852000	34.678550000
8	-18.444082000	-36.108735000	34.331949000
8	-16.987503000	-34.415507000	33.953935000
1	-18.997445000	-35.133182000	37.072704000
1	-16.653279000	-34.377950000	36.404079000
1	-16.197627000	-36.052398000	36.149205000
7	-17.731509000	-38.205832000	37.019018000
6	-18.098716000	-39.614969000	37.002098000
6	-17.495388000	-40.403479000	35.827329000
8	-16.089014000	-40.565832000	35.992956000
6	-17.862626000	-39.814752000	34.463603000
1	-16.772024000	-37.964434000	36.801853000
1	-19.190889000	-39.645819000	36.970436000
1	-17.902027000	-41.421730000	35.894176000
1	-15.651139000	-39.711117000	35.786498000
1	-17.445954000	-40.434852000	33.663451000
1	-18.952199000	-39.769590000	34.343357000
1	-17.492936000	-38.789652000	34.355673000
6	-13.815845000	-36.332227000	34.173445000
6	-14.092608000	-37.799019000	34.455403000
8	-14.819801000	-38.131433000	35.413504000
1	-13.472557000	-35.857868000	35.098569000
1	-14.742178000	-35.826053000	33.876768000
7	-13.502952000	-38.671699000	33.615800000
6	-13.589734000	-40.125204000	33.724438000
6	-12.430761000	-40.717419000	34.533801000

1	-12.879942000	-38.310349000	32.892422000
1	-13.587959000	-40.530842000	32.706681000
1	-12.509237000	-41.810718000	34.567993000
1	-11.464033000	-40.455386000	34.087768000
1	-12.450809000	-40.343179000	35.562962000
6	-10.962013000	-36.064103000	30.036371000
6	-11.663600000	-37.383530000	30.372985000
8	-11.628018000	-37.799393000	31.542078000
6	-11.296635000	-35.343908000	28.721623000
6	-12.702322000	-34.713590000	28.627205000
6	-12.905950000	-33.592090000	29.658074000
6	-12.951520000	-34.195791000	27.203041000
1	-11.125830000	-35.408666000	30.897271000
1	-10.555415000	-34.543754000	28.578648000
1	-11.148038000	-36.028332000	27.874537000
1	-13.443261000	-35.500158000	28.836549000
1	-13.909434000	-33.160320000	29.568037000
1	-12.792843000	-33.943300000	30.689696000
1	-12.182255000	-32.781085000	29.497614000
1	-13.931527000	-33.712759000	27.123189000
1	-12.192618000	-33.449806000	26.927253000
1	-12.921283000	-35.005145000	26.464290000
7	-12.217150000	-38.077754000	29.358560000
6	-12.835942000	-39.392485000	29.514020000
6	-12.639586000	-40.255007000	28.264026000
6	-13.153735000	-39.661971000	26.940509000
8	-13.689637000	-38.491217000	26.989916000
8	-13.003259000	-40.330490000	25.896369000
1	-12.386016000	-37.645655000	28.457173000
1	-13.903828000	-39.282408000	29.738833000
1	-11.575794000	-40.484353000	28.120105000
1	-13.130162000	-41.222134000	28.417666000
8	-17.253936000	-42.392720000	30.042462000
1	-12.111580000	-43.528426000	31.370923000
1	-12.044486000	-43.663178000	28.645764000
1	-18.619507000	-44.336725000	34.891388000
1	-15.972003000	-43.772367000	34.985351000
1	-22.123231000	-45.177812000	31.083553000
1	-22.077331000	-45.114766000	28.371666000
1	-18.269993000	-44.565218000	24.749864000
1	-15.577830000	-44.224212000	24.858899000
6	-16.378659000	-47.869644000	32.234985000
1	-17.023780000	-48.700616000	31.925746000
1	-15.635627000	-48.260598000	32.947051000
1	-17.002387000	-47.138106000	32.757475000
1	-17.769699000	-40.096037000	37.934496000
1	-21.199272000	-34.872304000	34.581209000
1	-24.359691000	-33.247646000	31.143850000
1	-27.649789000	-31.850777000	34.899242000
1	-28.996312000	-34.446673000	31.652643000
1	-8.934304000	-40.650190000	20.097008000
1	-11.346135000	-41.011757000	23.854300000
1	-15.144578000	-39.826613000	20.221246000
1	-11.474094000	-38.028866000	22.247341000
1	-12.372226000	-39.872905000	30.378335000
1	-9.888648000	-36.301649000	30.052468000
1	-13.059496000	-36.193904000	33.395671000
1	-14.544413000	-40.396274000	34.175734000
1	-17.717426000	-35.651282000	38.167408000
1	-16.637675000	-30.144355000	27.610373000
1	-23.063360000	-26.719926000	29.710585000
1	-18.041864000	-25.988775000	28.500725000
8	-24.783405000	-34.383145000	36.263093000
1	-24.626613000	-34.329231000	35.296069000
1	-25.127672000	-35.280892000	36.384031000

^{4}IM1$_{C6,III}$

6	-20.051774000	-43.685221000	28.609122000
6	-20.612976000	-42.358956000	33.232731000
6	-15.814884000	-42.070675000	33.794458000
6	-15.246616000	-43.580824000	29.226070000
6	-20.626866000	-43.329713000	29.824514000
6	-22.050518000	-43.289556000	30.079060000
6	-22.205850000	-42.924653000	31.381629000
6	-20.873756000	-42.740410000	31.920410000
6	-19.351180000	-42.157891000	33.786000000
6	-19.122562000	-41.692376000	35.138026000
6	-17.773791000	-41.594729000	35.293243000
6	-17.181499000	-42.006107000	34.038516000
6	-15.235714000	-42.493872000	32.600469000
6	-13.811777000	-42.609824000	32.373784000
6	-13.656242000	-43.059117000	31.096534000
6	-14.985208000	-43.204846000	30.541884000
6	-16.508974000	-43.696003000	28.653874000
6	-16.733492000	-44.110195000	27.286655000
6	-18.083763000	-44.151380000	27.109675000
6	-18.682323000	-43.752314000	28.365205000
7	-19.935279000	-42.984877000	30.955359000
7	-18.159782000	-42.347317000	33.143365000
7	-15.923934000	-42.871581000	31.480530000
7	-17.706184000	-43.465600000	29.282975000
26	-17.931755000	-42.929552000	31.221336000
1	-21.467368000	-42.213053000	33.888418000
1	-15.147414000	-41.788104000	34.603746000
1	-14.399645000	-43.791906000	28.578908000
1	-20.720501000	-43.947416000	27.794137000
6	-16.013990000	-36.433526000	29.208557000
6	-17.864931000	-37.563058000	29.775677000
6	-19.147243000	-38.026782000	30.191549000
6	-17.136774000	-39.803796000	29.074195000
6	-16.903303000	-38.471970000	29.188584000
6	-17.283311000	-36.282765000	29.798000000
6	-19.420050000	-39.362214000	30.077755000
6	-18.380985000	-40.396203000	29.672317000
7	-15.772549000	-37.710178000	28.859754000
1	-14.945217000	-38.035655000	28.327697000
6	-17.898702000	-35.028032000	30.355472000
6	-16.911695000	-34.089359000	31.083078000
7	-17.621597000	-33.029315000	31.783839000
6	-17.996255000	-31.887893000	31.188116000
8	-17.686025000	-31.597187000	30.008052000
6	-18.818710000	-30.939518000	32.038983000
1	-18.023651000	-33.245312000	32.710199000
8	-20.599372000	-39.931466000	30.331113000
6	-21.685200000	-39.116737000	30.770834000
1	-15.272182000	-35.669180000	29.013196000
1	-19.871996000	-37.321052000	30.578880000
1	-16.409648000	-40.507783000	28.683335000
1	-18.853885000	-41.108789000	28.982512000
1	-18.680412000	-35.309226000	31.070029000
1	-18.395791000	-34.448750000	29.564930000
1	-16.317536000	-34.647846000	31.813574000
1	-16.226375000	-33.624756000	30.367927000
1	-18.260017000	-30.006539000	32.171432000
1	-19.080148000	-31.377900000	33.003885000
1	-19.739183000	-30.691868000	31.498945000
1	-22.529784000	-39.791417000	30.898050000
1	-21.927616000	-38.354829000	30.020160000
1	-21.448403000	-38.626904000	31.719792000
6	-17.114178000	-45.544584000	33.294109000
16	-18.005101000	-45.192290000	31.722578000
1	-16.427051000	-44.722289000	33.505117000
1	-16.502843000	-46.438880000	33.116886000
6	-17.370340000	-39.426757000	22.884389000
6	-15.888820000	-39.497338000	22.533472000
8	-15.423386000	-40.468424000	21.919035000
6	-17.849398000	-40.575142000	23.799073000
6	-19.391693000	-40.608114000	23.819883000
6	-17.245888000	-40.454375000	25.206455000
6	-19.997620000	-41.818972000	24.538568000
1	-17.617150000	-38.458978000	23.341224000
1	-17.496608000	-41.512749000	23.347561000
1	-19.761507000	-39.683443000	24.288563000
1	-19.758295000	-40.592596000	22.782983000
1	-17.494774000	-41.323529000	25.823742000
1	-16.153167000	-40.382163000	25.189214000
1	-17.631768000	-39.559702000	25.715892000
1	-21.092363000	-41.800833000	24.476629000
1	-19.654560000	-42.756858000	24.082836000
1	-19.726779000	-41.849040000	25.598649000
7	-15.152700000	-38.415845000	22.881266000
6	-13.715309000	-38.292906000	22.691253000
6	-12.990780000	-38.305780000	24.037042000
8	-13.568384000	-37.322664000	24.884698000
1	-15.536617000	-37.766343000	23.555906000
1	-13.513815000	-37.348464000	22.168224000
1	-13.085522000	-39.296282000	24.494313000
1	-11.921217000	-38.098628000	23.865872000
1	-13.679839000	-37.748857000	25.775401000
6	-12.712975000	-42.654963000	23.929310000
6	-12.553459000	-43.169731000	22.503492000
8	-11.837731000	-44.137505000	22.244183000
6	-13.397851000	-43.687578000	24.842798000
8	-13.360837000	-43.314901000	26.207659000
1	-11.709404000	-42.467102000	24.328668000
1	-14.432805000	-43.846035000	24.492762000
1	-12.873335000	-44.644232000	24.754153000
1	-13.653247000	-42.376567000	26.283549000
7	-13.251435000	-42.482380000	21.550481000
6	-13.242696000	-42.876060000	20.154360000
6	-14.660195000	-42.990973000	19.551736000
6	-15.497736000	-44.101404000	20.147906000
6	-15.451336000	-45.399458000	19.621275000
6	-16.320614000	-43.859664000	21.257262000
6	-16.202127000	-46.431602000	20.186142000
6	-17.071131000	-44.890418000	21.826324000
6	-17.015285000	-46.179902000	21.293133000
1	-13.874595000	-41.725216000	21.823155000
1	-12.713490000	-43.831239000	20.102788000
1	-15.172046000	-42.029931000	19.681760000
1	-14.545943000	-43.151065000	18.470730000
1	-14.817759000	-45.603101000	18.759562000
1	-16.364171000	-42.854706000	21.668888000
1	-16.151620000	-47.432101000	19.762464000
1	-17.699801000	-44.685314000	22.689520000
1	-17.599276000	-46.981899000	21.737740000

6	-26.917432000	-40.971959000	32.640029000
6	-27.295418000	-40.640787000	31.201640000
8	-28.243048000	-41.200220000	30.623138000
6	-27.164242000	-42.455011000	32.976613000
8	-28.536521000	-42.791955000	32.828277000
6	-26.746665000	-42.775960000	34.406751000
1	-25.872663000	-40.712636000	32.837776000
1	-26.548824000	-43.062659000	32.288080000
1	-28.778866000	-42.423794000	31.952622000
1	-26.928152000	-43.834756000	34.621343000
1	-25.684746000	-42.556538000	34.560210000
1	-27.335099000	-42.179977000	35.114846000
7	-26.545566000	-39.691249000	30.591982000
6	-26.834638000	-39.249553000	29.234565000
6	-26.272348000	-40.165471000	28.125638000
6	-24.739556000	-40.280602000	28.209039000
6	-26.753317000	-39.654048000	26.759538000
6	-24.117700000	-41.303001000	27.250163000
1	-25.802837000	-39.225642000	31.116376000
1	-26.422715000	-38.238415000	29.122442000
1	-26.704043000	-41.161450000	28.294650000
1	-24.293818000	-39.290038000	28.026820000
1	-24.468340000	-40.556712000	29.235327000
1	-26.447242000	-40.320529000	25.946466000
1	-27.847295000	-39.577099000	26.729440000
1	-26.341511000	-38.658043000	26.544649000
1	-23.043539000	-41.407018000	27.438752000
1	-24.574538000	-42.292276000	27.382099000
1	-24.244167000	-41.016643000	26.199487000
6	-25.656546000	-36.133755000	31.531665000
6	-24.788855000	-36.958658000	32.484858000
8	-24.569146000	-38.159855000	32.203606000
6	-25.741916000	-34.607667000	31.709154000
6	-24.460228000	-33.785671000	31.435533000
6	-23.929400000	-33.991789000	30.008779000
6	-24.739957000	-32.297592000	31.696984000
1	-25.311519000	-36.385449000	30.522891000
1	-26.527858000	-34.248762000	31.028044000
1	-26.109285000	-34.377030000	32.719566000
1	-23.675570000	-34.091009000	32.139791000
1	-23.047509000	-33.365345000	29.832807000
1	-23.630963000	-35.029400000	29.821463000
1	-24.687498000	-33.719678000	29.260569000
1	-23.835800000	-31.697440000	31.544350000
1	-25.517630000	-31.914614000	31.020920000
1	-25.075662000	-32.131816000	32.727414000
7	-24.352395000	-36.357721000	33.593984000
6	-23.386700000	-36.870054000	34.568460000
6	-22.168485000	-35.925992000	34.577994000
8	-22.703905000	-34.608565000	34.571675000
6	-21.240216000	-36.153326000	33.380698000
1	-24.446337000	-35.351106000	33.673751000
1	-23.123147000	-37.899063000	34.320407000
1	-21.606056000	-36.104779000	35.505020000
1	-21.970739000	-33.994716000	34.879292000
1	-20.471099000	-35.374426000	33.367328000
1	-20.747993000	-37.132384000	33.446381000
1	-21.802387000	-36.103178000	32.438012000
6	-18.056565000	-25.416484000	29.574937000
6	-16.636633000	-25.306599000	29.011191000
8	-16.419158000	-24.799120000	27.915229000
6	-18.779765000	-26.674745000	29.065843000
6	-18.203783000	-27.971310000	29.647513000
8	-17.423616000	-27.953584000	30.614346000
7	-18.587574000	-29.115386000	29.055108000
1	-18.050513000	-25.423351000	30.669094000
1	-19.842427000	-26.622363000	29.340142000
1	-18.739463000	-26.710658000	27.970071000
1	-19.221165000	-29.098833000	28.268913000
1	-18.254380000	-30.032530000	29.416154000
7	-15.657956000	-25.822072000	29.819366000
6	-14.302076000	-26.020725000	29.341048000
6	-13.934380000	-27.420970000	28.806119000
8	-12.897510000	-27.555608000	28.160322000
1	-15.989517000	-26.377575000	30.600553000
1	-14.139003000	-25.321796000	28.519289000
7	-14.770363000	-28.442796000	29.120944000
6	-14.569663000	-29.791509000	28.602625000
6	-15.197858000	-29.974915000	27.203130000
6	-15.005022000	-31.368216000	26.644025000
6	-15.805673000	-32.436371000	27.079628000
6	-14.012275000	-31.625061000	25.688736000
6	-15.615938000	-33.721293000	26.564635000
6	-13.819186000	-32.911375000	25.179770000
6	-14.619699000	-33.970261000	25.615251000
1	-15.621435000	-28.268160000	29.645448000
1	-15.021905000	-30.494721000	29.308501000
1	-16.267847000	-29.743108000	27.278102000
1	-14.748574000	-29.233949000	26.532308000
1	-16.571435000	-32.260257000	27.832962000
1	-13.386140000	-30.805547000	25.341050000
1	-16.254688000	-34.533734000	26.903516000
1	-13.042579000	-33.088986000	24.439643000

1	-14.456346000	-34.973519000	25.228837000
6	-18.788482000	-34.143853000	37.583914000
6	-18.807277000	-35.653981000	37.328055000
8	-19.818539000	-36.340423000	37.475188000
6	-18.433448000	-33.292281000	36.348198000
6	-19.518783000	-33.380826000	35.249627000
8	-20.705585000	-33.252675000	35.651399000
8	-19.141270000	-33.570273000	34.056236000
1	-19.789758000	-33.869615000	37.919374000
1	-18.361751000	-32.242454000	36.665852000
1	-17.461078000	-33.567306000	35.925894000
7	-17.606270000	-36.184505000	36.958686000
6	-17.419310000	-37.561187000	36.536336000
6	-16.623320000	-37.612886000	35.225738000
8	-15.426939000	-36.868329000	35.475053000
6	-17.401165000	-37.073857000	34.025340000
1	-16.819921000	-35.577682000	36.765224000
1	-18.404463000	-38.020058000	36.425977000
1	-16.372632000	-38.669221000	35.032072000
1	-15.030900000	-36.602872000	34.616793000
1	-16.762774000	-37.077723000	33.135018000
1	-18.267136000	-37.712392000	33.817362000
1	-17.754880000	-36.051350000	34.197634000
6	-12.423261000	-35.575467000	31.537805000
6	-13.105884000	-36.482734000	32.550189000
8	-14.149312000	-36.110439000	33.108659000
1	-12.365301000	-34.567002000	31.957088000
1	-13.039920000	-35.516710000	30.633833000
7	-12.499854000	-37.667389000	32.772360000
6	-13.016838000	-38.689361000	33.679324000
6	-11.868421000	-39.448499000	34.344758000
1	-11.696360000	-37.922543000	32.193832000
1	-13.660115000	-39.390183000	33.128398000
1	-12.264465000	-40.234170000	34.998186000
1	-11.219348000	-39.920746000	33.597905000
1	-11.252937000	-38.774907000	34.951573000
6	-9.209134000	-37.598011000	28.983383000
6	-10.338648000	-38.274467000	29.766354000
8	-10.242475000	-38.404868000	30.997642000
6	-9.552406000	-36.915654000	27.648930000
6	-10.448152000	-35.662118000	27.741430000
6	-9.739212000	-34.501112000	28.456354000
6	-10.912420000	-35.236226000	26.340703000
1	-8.736951000	-36.896411000	29.677205000
1	-8.608512000	-36.630940000	27.161331000
1	-10.015682000	-37.644110000	26.968569000
1	-11.343797000	-35.922611000	28.324592000
1	-10.395535000	-33.625566000	28.517599000
1	-9.438933000	-34.759015000	29.478466000
1	-8.835971000	-34.200937000	27.907302000
1	-11.501559000	-34.313784000	26.386650000
1	-10.049049000	-35.049400000	25.686236000
1	-11.543092000	-36.000854000	25.872602000
7	-11.360631000	-38.781761000	29.047201000
6	-12.450778000	-39.574287000	29.611871000
6	-13.015955000	-40.531227000	28.561260000
6	-13.561322000	-39.856486000	27.290797000
8	-13.604261000	-38.569775000	27.296335000
8	-13.920293000	-40.583591000	26.341315000
1	-11.531575000	-38.450940000	28.105645000
1	-13.240928000	-38.917934000	29.996616000
1	-12.258320000	-41.260151000	28.247754000
1	-13.823567000	-41.119507000	29.011196000
8	-17.844950000	-41.022491000	30.831918000
1	-13.047812000	-42.384307000	33.108071000
1	-12.739531000	-43.275000000	30.561731000
1	-19.903459000	-41.465604000	35.853419000
1	-17.216137000	-41.277710000	36.165964000
1	-23.120096000	-42.770642000	31.941934000
1	-22.814879000	-43.520087000	29.347040000
1	-18.640308000	-44.428606000	26.222763000
1	-15.938378000	-44.325150000	26.584535000
6	-18.045440000	-45.780262000	34.486053000
1	-18.737799000	-46.606135000	34.284737000
1	-17.468780000	-46.030367000	35.389733000
1	-18.642287000	-44.887487000	34.695558000
1	-16.867040000	-38.118809000	37.307193000
1	-23.865507000	-36.865211000	35.554360000
1	-26.665643000	-36.562287000	31.606896000
1	-27.545979000	-40.361445000	33.304527000
1	-27.922696000	-39.185655000	29.122543000
1	-12.672177000	-42.146753000	19.558048000
1	-13.270391000	-41.713066000	23.964236000
1	-17.909312000	-39.477680000	21.928881000
1	-13.383180000	-39.117189000	22.056210000
1	-12.052853000	-40.134480000	30.461308000
1	-8.463669000	-38.385626000	28.801137000
1	-11.423209000	-35.919693000	31.263421000
1	-13.639792000	-38.196174000	34.428347000
1	-18.075329000	-33.938034000	38.395786000
1	-13.494176000	-29.980848000	28.554603000
1	-18.600790000	-24.531383000	29.233481000
1	-13.586344000	-25.775850000	30.135240000

8	-23.512173000	-40.415780000	33.543482000
1	-23.838729000	-39.589318000	33.130921000
1	-22.650035000	-40.575178000	33.130901000

$^2P_{C6,III}$

6	-16.454119000	-43.220818000	27.782215000
6	-20.226048000	-41.826099000	30.483415000
6	-18.347904000	-44.082039000	34.328824000
6	-14.751960000	-45.750837000	31.544995000
6	-17.648837000	-42.645863000	28.195794000
6	-18.503415000	-41.859535000	27.339410000
6	-19.572348000	-41.481941000	28.092096000
6	-19.369053000	-42.033784000	29.411004000
6	-20.028471000	-42.311705000	31.769307000
6	-20.884471000	-41.991259000	32.888778000
6	-20.353695000	-42.622521000	33.971805000
6	-19.175804000	-43.321856000	33.515293000
6	-17.204476000	-44.744752000	33.899602000
6	-16.357488000	-45.547491000	34.746826000
6	-15.359728000	-46.032346000	33.955687000
6	-15.593002000	-45.518520000	32.627310000
6	-14.892380000	-45.193256000	30.281758000
6	-13.989795000	-45.413084000	29.179691000
6	-14.452845000	-44.680415000	28.126309000
6	-15.649633000	-44.019973000	28.583544000
7	-18.194034000	-42.742651000	29.454828000
7	-18.996913000	-43.124799000	32.167860000
7	-16.725665000	-44.741282000	32.612387000
7	-15.894937000	-44.321579000	29.908164000
26	-17.541923000	-43.842433000	31.007541000
1	-21.105322000	-41.209888000	30.321215000
1	-18.610830000	-44.161867000	35.379588000
1	-13.897974000	-46.401815000	31.709280000
1	-16.117273000	-43.051456000	26.763326000
6	-15.405955000	-36.665464000	29.240521000
6	-16.263864000	-37.924349000	30.898921000
6	-16.874363000	-38.482027000	32.041091000
6	-15.530950000	-40.156807000	30.181989000
6	-15.575560000	-38.768551000	29.992960000
6	-16.160809000	-36.579101000	30.387993000
6	-16.786351000	-39.851070000	32.266913000
6	-16.124282000	-40.681697000	31.319720000
7	-15.063836000	-37.979932000	28.981898000
1	-14.333382000	-38.268725000	28.331886000
6	-16.792527000	-35.355649000	30.984243000
6	-16.007520000	-34.770533000	32.178257000
7	-16.719499000	-33.712365000	32.889751000
6	-16.825212000	-32.450430000	32.416757000
8	-16.358116000	-32.087727000	31.323147000
6	-17.541214000	-31.473122000	33.342544000
1	-17.362793000	-33.989982000	33.634259000
8	-17.288507000	-40.497796000	33.360008000
6	-17.891719000	-39.699977000	34.366935000
1	-15.103463000	-35.881794000	28.559215000
1	-17.368167000	-37.839281000	32.760540000
1	-15.072623000	-40.818387000	29.452187000
1	-15.463110000	-42.491545000	30.918153000
1	-17.803350000	-35.604077000	31.332341000
1	-16.901252000	-34.571530000	30.227363000
1	-15.781728000	-35.549732000	32.912488000
1	-15.059574000	-34.358931000	31.817236000
1	-17.525288000	-30.480494000	32.889099000
1	-17.034638000	-31.433018000	34.313948000
1	-18.577522000	-31.776534000	33.527446000
1	-18.175049000	-40.393961000	35.160664000
1	-18.794983000	-39.199301000	33.991104000
1	-17.196963000	-38.947702000	34.757908000
6	-19.561511000	-46.420855000	31.653412000
16	-18.626420000	-45.652867000	30.267043000
1	-19.109211000	-46.111095000	32.597764000
1	-19.404198000	-47.502899000	31.558678000
6	-16.274689000	-37.252523000	23.394387000
6	-14.895956000	-37.727542000	22.956250000
8	-14.765343000	-38.652877000	22.140744000
6	-17.080560000	-38.312464000	24.178817000
6	-18.537085000	-37.826922000	24.332141000
6	-16.416415000	-38.627172000	25.527392000
6	-19.499422000	-38.858918000	24.930771000
1	-16.198749000	-36.333536000	23.989840000
1	-17.090715000	-39.228615000	23.570373000
1	-18.547385000	-36.918037000	24.951575000
1	-18.913184000	-37.527074000	23.343138000
1	-16.973070000	-39.387791000	26.084090000
1	-15.395740000	-39.007326000	25.409956000
1	-16.374418000	-37.731112000	26.160676000
1	-20.524838000	-38.470597000	24.948168000
1	-19.505994000	-39.783161000	24.337754000
1	-19.229119000	-39.122702000	25.958144000
7	-13.849756000	-37.039229000	23.472362000
6	-12.450667000	-37.360294000	23.231989000
6	-11.803561000	-37.977425000	24.472341000
8	-12.015356000	-37.122530000	25.585114000
1	-14.019174000	-36.444819000	24.274610000
1	-11.926719000	-36.433796000	22.962251000
1	-12.245531000	-38.961224000	24.663439000
1	-10.725767000	-38.112053000	24.277445000
1	-12.331657000	-37.703185000	26.324002000
6	-13.048441000	-42.168867000	23.206292000
6	-13.087524000	-42.245953000	21.684465000
8	-12.786960000	-43.278277000	21.083588000
6	-14.249372000	-42.881913000	23.855987000
8	-14.106682000	-43.001115000	25.260163000
1	-12.135431000	-42.675164000	23.536901000
1	-15.175651000	-42.347245000	23.588021000
1	-14.327794000	-43.896985000	23.452582000
1	-13.868014000	-42.112260000	25.623756000
7	-13.484108000	-41.102739000	21.050050000
6	-13.627517000	-41.034253000	19.608195000
6	-14.992496000	-40.467982000	19.159539000
6	-16.176049000	-41.335444000	19.529335000
6	-16.593961000	-42.379092000	18.692242000
6	-16.868348000	-41.124491000	20.730381000
6	-17.671766000	-43.193742000	19.042564000
6	-17.946108000	-41.938616000	21.084521000
6	-18.352276000	-42.975813000	20.242425000
1	-13.775743000	-40.291749000	21.591937000
1	-13.479338000	-42.050154000	19.232573000
1	-15.115412000	-39.470151000	19.596411000
1	-14.951506000	-40.340251000	18.068881000
1	-16.067862000	-42.555190000	17.755348000
1	-16.550371000	-40.315840000	21.383367000
1	-17.980424000	-43.998134000	18.378599000
1	-18.469428000	-41.761731000	22.021148000
1	-19.191351000	-43.609337000	20.519309000
6	-27.099148000	-38.564912000	32.471489000
6	-27.274541000	-38.894190000	30.991861000
8	-28.286621000	-39.487061000	30.578612000
6	-27.598919000	-39.713407000	33.372469000
8	-28.984753000	-39.941311000	33.172173000
6	-27.386613000	-39.407409000	34.850920000
1	-26.055282000	-38.315653000	32.690973000
1	-27.021353000	-40.620125000	33.115853000
1	-29.091351000	-39.949853000	32.196178000
1	-27.787184000	-40.225215000	35.459425000
1	-26.322266000	-39.287396000	35.082316000
1	-27.914663000	-38.487546000	35.129409000
7	-26.281284000	-38.493401000	30.168684000
6	-26.343083000	-38.718123000	28.729192000
6	-25.907384000	-40.129944000	28.283987000
6	-24.417051000	-40.392890000	28.576766000
6	-26.252468000	-40.316427000	26.798408000
6	-23.994881000	-41.857493000	28.403175000
1	-25.477678000	-38.001824000	30.564655000
1	-25.708137000	-37.962161000	28.250008000
1	-26.508749000	-40.839556000	28.868770000
1	-23.808820000	-39.754857000	27.916121000
1	-24.180580000	-40.080613000	29.601545000
1	-26.011467000	-41.326030000	26.450287000
1	-27.321494000	-40.150424000	26.616692000
1	-25.691769000	-39.608485000	26.172534000
1	-22.942672000	-42.000088000	28.672598000
1	-24.590221000	-42.515085000	29.049015000
1	-24.117957000	-42.204575000	27.370707000
6	-25.430531000	-34.993824000	31.248488000
6	-24.115003000	-35.726029000	31.510524000
8	-24.066656000	-36.961590000	31.324182000
6	-25.429280000	-33.471811000	31.034330000
6	-24.718133000	-32.934035000	29.773041000
6	-25.228518000	-33.589122000	28.480590000
6	-24.882529000	-31.407746000	29.702829000
1	-25.899396000	-35.502544000	30.401125000
1	-26.482025000	-33.156456000	30.991235000
1	-25.016916000	-32.969156000	31.921655000
1	-23.643844000	-33.152678000	29.858682000
1	-24.746032000	-33.136974000	27.606981000
1	-25.023695000	-34.665177000	28.449317000
1	-26.312995000	-33.449634000	28.371512000
1	-24.317400000	-30.974972000	28.870105000
1	-25.940171000	-31.139567000	29.570908000
1	-24.535718000	-30.931282000	30.628866000
7	-23.090275000	-35.018542000	32.002940000
6	-21.777595000	-35.539554000	32.378051000
6	-20.663441000	-34.745576000	31.669650000
8	-20.879713000	-33.348228000	31.883951000
6	-20.606008000	-35.006795000	30.169004000
1	-23.152819000	-34.008633000	32.045596000
1	-21.740110000	-36.604161000	32.141799000
1	-19.720625000	-35.044729000	32.143735000
1	-20.697663000	-33.169482000	32.868822000
1	-19.816503000	-34.405838000	29.707772000
1	-20.390767000	-36.061927000	29.965073000
1	-21.556381000	-34.748529000	29.687131000
6	-21.881055000	-28.912197000	29.649371000
6	-21.845553000	-28.906201000	28.121316000
8	-22.683697000	-29.507037000	27.451296000
6	-21.265643000	-30.189353000	30.241523000

6	-19.743977000	-30.256514000	30.081893000
8	-19.094510000	-29.295403000	29.627229000
7	-19.179269000	-31.414589000	30.454518000
1	-21.366513000	-28.036997000	30.058217000
1	-21.498296000	-30.252186000	31.312258000
1	-21.715072000	-31.078586000	29.785916000
1	-19.736313000	-32.138805000	30.924457000
1	-18.161176000	-31.473665000	30.516570000
7	-20.819280000	-28.183858000	27.573458000
6	-20.503687000	-28.238951000	26.158751000
6	-19.198575000	-28.948246000	25.750218000
8	-18.896462000	-28.978809000	24.558345000
1	-20.092688000	-27.910893000	28.225259000
1	-21.329255000	-28.761566000	25.669030000
7	-18.449496000	-29.481854000	26.747037000
6	-17.231327000	-30.235365000	26.491924000
6	-17.441666000	-31.756161000	26.659917000
6	-16.165558000	-32.543934000	26.459004000
6	-15.336651000	-32.863417000	27.545597000
6	-15.771884000	-32.950236000	25.176045000
6	-14.155790000	-33.584085000	27.350083000
6	-14.590833000	-33.670181000	24.980372000
6	-13.778406000	-33.999406000	26.069610000
1	-18.763213000	-29.438713000	27.714919000
1	-16.452616000	-29.893833000	27.184179000
1	-17.844251000	-31.947063000	27.662188000
1	-18.204702000	-32.076436000	25.940033000
1	-15.622512000	-32.562665000	28.552495000
1	-16.403943000	-32.706726000	24.324080000
1	-13.533596000	-33.833136000	28.206036000
1	-14.307836000	-33.977970000	23.975914000
1	-12.878330000	-34.591599000	25.926734000
6	-19.390638000	-34.647112000	37.538994000
6	-17.881076000	-34.712962000	37.798889000
8	-17.402436000	-34.424983000	38.898891000
6	-19.847427000	-33.468303000	36.652529000
6	-19.780946000	-33.676102000	35.124330000
8	-20.551337000	-32.923948000	34.453960000
8	-18.984597000	-34.534782000	34.647122000
1	-19.729299000	-35.588486000	37.089168000
1	-20.884304000	-33.206189000	36.889331000
1	-19.256494000	-32.571734000	36.888840000
7	-17.147228000	-35.108830000	36.726526000
6	-15.698155000	-35.146212000	36.727415000
6	-15.121190000	-36.537734000	36.441032000
8	-15.544539000	-36.913504000	35.136512000
6	-15.522996000	-37.583514000	37.483921000
1	-17.652598000	-35.196963000	35.843194000
1	-15.368125000	-34.796262000	37.709423000
1	-14.020626000	-36.428422000	36.461866000
1	-15.088232000	-37.758392000	34.908821000
1	-15.095281000	-38.556587000	37.219461000
1	-15.167594000	-37.302708000	38.483493000
1	-16.612998000	-37.678875000	37.519571000
6	-12.619722000	-37.976392000	33.334834000
6	-13.214315000	-39.269265000	33.862490000
8	-14.177460000	-39.268185000	34.648889000
1	-12.556362000	-37.250287000	34.149835000
1	-13.297078000	-37.563853000	32.578282000
7	-12.659912000	-40.411052000	33.406593000
6	-13.209836000	-41.721086000	33.745659000
6	-12.713691000	-42.257178000	35.092260000
1	-11.845766000	-40.359911000	32.792960000
1	-12.932519000	-42.407898000	32.938523000
1	-13.153605000	-43.242605000	35.289150000
1	-11.621496000	-42.355305000	35.104114000
1	-13.011442000	-41.579247000	35.898008000
6	-9.009840000	-38.468331000	30.408025000
6	-10.129236000	-39.495323000	30.601378000
8	-10.244875000	-40.076427000	31.693402000
6	-9.169429000	-37.398981000	29.314954000
6	-10.302123000	-36.370026000	29.517593000
6	-10.116590000	-35.545503000	30.800842000
6	-10.392871000	-35.451882000	28.288962000
1	-8.844957000	-38.011293000	31.388318000
1	-8.217644000	-36.851596000	29.242222000
1	-9.298697000	-37.886173000	28.338596000
1	-11.254314000	-36.913151000	29.597950000
1	-10.923120000	-34.811120000	30.908534000
1	-10.121649000	-36.168457000	31.702190000
1	-9.165819000	-34.994304000	30.777835000
1	-11.179907000	-34.699395000	28.420293000
1	-9.446937000	-34.912969000	28.135220000
1	-10.619843000	-36.016617000	27.377692000
7	-10.886124000	-39.790346000	29.525758000
6	-11.965302000	-40.772934000	29.568194000
6	-12.334524000	-41.237401000	28.156361000
6	-12.831217000	-40.133980000	27.197741000
8	-12.596343000	-38.923285000	27.541952000
8	-13.399745000	-40.488534000	26.139755000
1	-10.988034000	-39.130487000	28.758778000
1	-12.841144000	-40.349304000	30.074458000
1	-11.462607000	-41.708152000	27.680702000
1	-13.099504000	-42.019591000	28.214329000
8	-16.067508000	-42.037371000	31.605860000
1	-16.515721000	-45.702737000	35.806948000
1	-14.527711000	-46.668441000	34.232155000
1	-21.738907000	-41.328979000	32.824815000
1	-20.702602000	-42.611187000	34.997171000
1	-20.423214000	-40.878503000	27.801853000
1	-18.292177000	-41.637025000	26.301767000
1	-14.049444000	-44.562768000	27.127534000
1	-13.111920000	-46.046406000	29.223665000
6	-21.057171000	-46.100319000	31.635993000
1	-21.516724000	-46.415771000	30.692494000
1	-21.571529000	-46.621341000	32.456417000
1	-21.228625000	-45.026356000	31.750989000
1	-15.314903000	-34.456826000	35.962162000
1	-21.652687000	-35.426944000	33.460389000
1	-26.066509000	-35.227039000	32.114660000
1	-27.709303000	-37.677532000	32.693645000
1	-27.375878000	-38.545706000	28.408434000
1	-12.834785000	-40.401759000	19.178643000
1	-13.013612000	-41.133736000	23.562284000
1	-16.814978000	-36.998723000	22.472993000
1	-12.402385000	-38.044862000	22.381842000
1	-11.621709000	-41.620456000	30.168981000
1	-8.103616000	-39.055774000	30.199756000
1	-11.633442000	-38.122805000	32.887382000
1	-14.299891000	-41.641616000	33.753021000
1	-19.854015000	-34.557295000	38.525762000
1	-16.911504000	-30.005150000	25.473083000
1	-22.934271000	-28.859017000	29.939656000
1	-20.455356000	-27.230627000	25.729409000
8	-22.326007000	-39.200753000	31.569784000
1	-22.898202000	-38.410277000	31.491853000
1	-21.512571000	-38.965657000	31.099964000

$^4P_{C6,III}$

6	-20.685073000	-42.007734000	31.135654000
6	-18.886119000	-44.227643000	35.044063000
6	-14.973170000	-45.517996000	32.497021000
6	-16.740474000	-43.240309000	28.602131000
6	-20.533597000	-42.521542000	32.414448000
6	-21.478546000	-42.304195000	33.480921000
6	-20.968574000	-42.922621000	34.582520000
6	-19.717188000	-43.518625000	34.189588000
6	-17.664698000	-44.777799000	34.681759000
6	-16.796758000	-45.492288000	35.581435000
6	-15.701378000	-45.863134000	34.860713000
6	-15.896750000	-45.370820000	33.521793000
6	-15.110642000	-44.993204000	31.220968000
6	-14.152218000	-45.158962000	30.161887000
6	-14.646078000	-44.515233000	29.064220000
6	-15.912194000	-43.954167000	29.455571000
6	-17.973685000	-42.718095000	28.966899000
6	-18.812175000	-41.950601000	28.083010000
6	-19.917974000	-41.597771000	28.795689000
6	-19.759785000	-42.157231000	30.113532000
7	-19.469623000	-43.273865000	32.858997000
7	-17.105276000	-44.718992000	33.426588000
7	-16.179191000	-44.234132000	30.782955000
7	-18.567760000	-42.838754000	30.203192000
26	-17.943919000	-43.964241000	31.755208000
1	-19.205785000	-44.345027000	36.074887000
1	-14.068186000	-46.077357000	32.713834000
1	-16.380668000	-43.078685000	27.590100000
1	-21.577726000	-41.420418000	30.942977000
6	-16.281191000	-36.615169000	29.244538000
6	-16.931607000	-37.680067000	31.119381000
6	-17.447386000	-38.119334000	32.357297000
6	-15.935439000	-39.895892000	30.738022000
6	-16.155674000	-38.568083000	30.336842000
6	-17.019151000	-36.435996000	30.392522000
6	-17.193834000	-39.415973000	32.784294000
6	-16.451932000	-40.311728000	31.956564000
7	-15.769095000	-37.899779000	29.190730000
1	-15.006412000	-38.190027000	28.580180000
6	-17.819036000	-35.227888000	30.784417000
6	-17.170174000	-34.340342000	31.870344000
7	-18.096547000	-33.329457000	32.373365000
6	-18.406922000	-32.215004000	31.694792000
8	-17.900324000	-31.928505000	30.584961000
6	-19.386102000	-31.275410000	32.380860000
1	-18.629338000	-33.563384000	33.222811000
8	-17.613741000	-39.943695000	33.973929000
6	-18.201913000	-39.050709000	34.904705000
1	-16.102569000	-35.925303000	28.431034000
1	-18.014321000	-37.437476000	32.980481000
1	-15.401080000	-40.599878000	30.106168000
1	-15.854270000	-42.197421000	31.681622000
1	-18.802890000	-35.543660000	31.158379000
1	-18.002517000	-34.596084000	29.908346000
1	-16.846530000	-34.938949000	32.727456000
1	-16.290107000	-33.839100000	31.455119000
1	-20.008708000	-30.787800000	31.626183000

1	-18.820617000	-30.488052000	32.895002000
1	-20.029712000	-31.778889000	33.107443000
1	-18.358687000	-39.631844000	35.816424000
1	-19.172757000	-38.677182000	34.546914000
1	-17.547143000	-38.197082000	35.116504000
6	-19.865911000	-46.797999000	32.322154000
16	-18.941826000	-46.027351000	30.926654000
1	-19.445979000	-46.438348000	33.266098000
1	-19.670716000	-47.876410000	32.273048000
6	-16.967286000	-37.766431000	23.471482000
6	-15.555125000	-38.205644000	23.107358000
8	-15.357740000	-39.226720000	22.432436000
6	-17.694025000	-38.737357000	24.430310000
6	-19.182454000	-38.340071000	24.519808000
6	-17.007858000	-38.777926000	25.803474000
6	-20.065366000	-39.325736000	25.293866000
1	-16.960985000	-36.758231000	23.906490000
1	-17.637796000	-39.739218000	23.980530000
1	-19.259568000	-37.344308000	24.981292000
1	-19.580097000	-38.237192000	23.499690000
1	-17.516228000	-39.461603000	26.490448000
1	-15.967779000	-39.114984000	25.739205000
1	-17.018851000	-37.785850000	26.274364000
1	-21.118688000	-39.022801000	25.250503000
1	-19.995442000	-40.336081000	24.870295000
1	-19.783274000	-39.384896000	26.350072000
7	-14.561046000	-37.386450000	23.524879000
6	-13.141745000	-37.661076000	23.352564000
6	-12.500855000	-38.125576000	24.660312000
8	-12.775397000	-37.175768000	25.677521000
1	-14.779066000	-36.694858000	24.230861000
1	-12.655555000	-36.740074000	23.005793000
1	-12.906790000	-39.103901000	24.939254000
1	-11.414192000	-38.233432000	24.500234000
1	-13.030894000	-37.692359000	26.486013000
6	-13.459804000	-42.487999000	23.894823000
6	-13.530981000	-42.774857000	22.399309000
8	-13.205765000	-43.868738000	21.936889000
6	-14.545463000	-43.248820000	24.678255000
8	-14.368214000	-43.146709000	26.080341000
1	-12.482142000	-42.830343000	24.251702000
1	-15.538256000	-42.881196000	24.369875000
1	-14.495580000	-44.311804000	24.421323000
1	-14.230274000	-42.192480000	26.309505000
7	-13.983420000	-41.742031000	21.627529000
6	-14.179559000	-41.876486000	20.196691000
6	-15.593628000	-41.455196000	19.737830000
6	-16.702237000	-42.339692000	20.265068000
6	-17.096437000	-43.491484000	19.570598000
6	-17.342595000	-42.037753000	21.475578000
6	-18.099715000	-44.323669000	20.069546000
6	-18.345414000	-42.869126000	21.978514000
6	-18.727910000	-44.014974000	21.277822000
1	-14.294107000	-40.877770000	22.066004000
1	-13.982275000	-42.923667000	19.952881000
1	-15.764939000	-40.419245000	20.052449000
1	-15.599731000	-41.465071000	18.639128000
1	-16.609773000	-43.739481000	18.628730000
1	-17.042669000	-41.144407000	22.017130000
1	-18.390588000	-45.213158000	19.515242000
1	-18.828808000	-42.621394000	22.920522000
1	-19.508151000	-44.661918000	21.670949000
6	-26.880262000	-39.696805000	31.038544000
6	-27.070649000	-40.040175000	29.568255000
8	-28.030168000	-40.732752000	29.187195000
6	-27.050879000	-40.949253000	31.924205000
8	-28.377837000	-41.447972000	31.831918000
6	-26.749006000	-40.648229000	33.387082000
1	-25.908705000	-39.225793000	31.211068000
1	-26.332395000	-41.711751000	31.573238000
1	-28.583841000	-41.428486000	30.873523000
1	-26.923378000	-41.543708000	33.993571000
1	-25.705659000	-40.336646000	33.506091000
1	-27.406836000	-39.853149000	33.759091000
7	-26.139995000	-39.557208000	28.712001000
6	-26.191838000	-39.844008000	27.285057000
6	-25.597740000	-41.213096000	26.887502000
6	-24.100007000	-41.306218000	27.234707000
6	-25.874142000	-41.471780000	25.399117000
6	-23.502077000	-42.712147000	27.097981000
1	-25.351608000	-39.028859000	29.085897000
1	-25.655548000	-39.039726000	26.764462000
1	-26.136809000	-41.967958000	27.476392000
1	-23.539775000	-40.603514000	26.597601000
1	-23.958515000	-40.963698000	28.267843000
1	-25.523484000	-42.461119000	25.087546000
1	-26.948081000	-41.418052000	25.181994000
1	-25.367326000	-40.727653000	24.769047000
1	-22.455209000	-42.728090000	27.420288000
1	-24.048576000	-43.432027000	27.719751000
1	-23.531862000	-43.074665000	26.063880000
6	-23.475972000	-36.258723000	28.475603000
6	-23.672083000	-36.815854000	29.884752000
8	-23.992483000	-38.020016000	30.015032000
6	-22.857598000	-34.862051000	28.297020000
6	-21.363802000	-34.709013000	28.662882000
6	-20.459812000	-35.587516000	27.784930000
6	-20.950711000	-33.231546000	28.559769000
1	-22.902107000	-37.015548000	27.930516000
1	-22.980145000	-34.582008000	27.240706000
1	-23.449602000	-34.125939000	28.860707000
1	-21.220083000	-35.011401000	29.708775000
1	-19.408031000	-35.463048000	28.061629000
1	-20.692203000	-36.653292000	27.886115000
1	-20.565010000	-35.318425000	26.724573000
1	-19.910475000	-33.091305000	28.872960000
1	-21.049388000	-32.870379000	27.526092000
1	-21.582858000	-32.605313000	29.203171000
7	-23.540830000	-35.976735000	30.915338000
6	-23.739067000	-36.284487000	32.330325000
6	-22.596278000	-35.645202000	33.140320000
8	-22.426814000	-34.341903000	32.613039000
6	-21.300619000	-36.460998000	33.078638000
1	-23.194147000	-35.034830000	30.770729000
1	-23.780474000	-37.365987000	32.465896000
1	-22.928784000	-35.593224000	34.189325000
1	-21.936478000	-33.794863000	33.294513000
1	-20.507090000	-35.927585000	33.611426000
1	-21.433981000	-37.446981000	33.546032000
1	-20.981246000	-36.607492000	32.039479000
6	-20.172410000	-26.401258000	28.248374000
6	-19.148241000	-25.949378000	27.204281000
8	-19.458449000	-25.800320000	26.026238000
6	-20.320594000	-27.930785000	28.299497000
6	-19.153462000	-28.639669000	29.001047000
8	-18.179466000	-28.011294000	29.445311000
7	-19.256277000	-29.978083000	29.085831000
1	-19.917111000	-26.026557000	29.244726000
1	-21.247377000	-28.192219000	28.827438000
1	-20.423863000	-28.327318000	27.281008000
1	-20.089857000	-30.443902000	28.753682000
1	-18.605677000	-30.555979000	29.651075000
7	-17.886671000	-25.730498000	27.691001000
6	-16.757897000	-25.522338000	26.804090000
6	-15.862437000	-26.738854000	26.493826000
8	-14.998318000	-26.629966000	25.626915000
1	-17.718290000	-26.084757000	28.626650000
1	-17.155385000	-25.172596000	25.849538000
7	-16.074147000	-27.856254000	27.234886000
6	-15.345021000	-29.097307000	27.018291000
6	-16.244575000	-30.204817000	26.426847000
6	-15.509605000	-31.518004000	26.270121000
6	-15.423190000	-32.421867000	27.339864000
6	-14.868281000	-31.842755000	25.066550000
6	-14.708284000	-33.614113000	27.205599000
6	-14.162764000	-33.039884000	24.929034000
6	-14.076908000	-33.934346000	25.999103000
1	-16.800178000	-27.867102000	27.945553000
1	-14.923014000	-29.438893000	27.971296000
1	-17.112479000	-30.340876000	27.084395000
1	-16.623782000	-29.858715000	25.457444000
1	-15.928696000	-32.198923000	28.278406000
1	-14.928881000	-31.150484000	24.228596000
1	-14.644007000	-34.297494000	28.047972000
1	-13.677539000	-33.274438000	23.984052000
1	-13.528926000	-34.868213000	25.897085000
6	-19.685281000	-34.054509000	37.738752000
6	-18.160441000	-33.997019000	37.905103000
8	-17.644883000	-33.759196000	39.001716000
6	-20.310702000	-33.017135000	36.776998000
6	-20.384916000	-33.409209000	35.286011000
8	-21.385565000	-32.973285000	34.652298000
8	-19.442134000	-34.104301000	34.795746000
1	-19.969778000	-35.064355000	37.415336000
1	-21.333105000	-32.789625000	37.094788000
1	-19.754506000	-32.070663000	36.842110000
7	-17.460268000	-34.239646000	36.769675000
6	-16.012913000	-34.244186000	36.717105000
6	-15.410399000	-35.633401000	36.466522000
8	-15.860991000	-36.068092000	35.189554000
6	-15.757568000	-36.643813000	37.562565000
1	-18.004224000	-34.373175000	35.911570000
1	-15.650873000	-33.848639000	37.670275000
1	-14.312219000	-35.498746000	36.452371000
1	-15.436710000	-36.938973000	35.005766000
1	-15.306612000	-37.614834000	37.331297000
1	-15.392789000	-36.307370000	38.540898000
1	-16.843119000	-36.770352000	37.626615000
6	-13.239998000	-37.277932000	33.150138000
6	-13.592791000	-38.492744000	33.988439000
8	-14.506062000	-38.461706000	34.830840000
1	-13.396095000	-36.372465000	33.741025000
1	-13.924863000	-37.234045000	32.294877000
7	-12.887982000	-39.616144000	33.736780000
6	-13.215401000	-40.885493000	34.378657000
6	-12.617373000	-41.023453000	35.782819000

1	-12.107485000	-39.583623000	33.079948000
1	-12.842016000	-41.682142000	33.725814000
1	-12.883320000	-41.995547000	36.216556000
1	-11.523995000	-40.943897000	35.757458000
1	-13.009277000	-40.236927000	36.434443000
6	-9.406702000	-37.925147000	30.415458000
6	-10.490558000	-38.945221000	30.773449000
8	-10.535185000	-39.415454000	31.921767000
6	-9.652016000	-36.975697000	29.231489000
6	-10.791728000	-35.948106000	29.398254000
6	-10.539015000	-34.983429000	30.567203000
6	-10.987343000	-35.174923000	28.085547000
1	-9.200285000	-37.368014000	31.334151000
1	-8.717876000	-36.425178000	29.044602000
1	-9.829415000	-37.561954000	28.318999000
1	-11.722859000	-36.495000000	29.604978000
1	-11.353400000	-34.254624000	30.649304000
1	-10.467317000	-35.502040000	31.529908000
1	-9.605517000	-34.423109000	30.416368000
1	-11.785886000	-34.430261000	28.179673000
1	-10.068117000	-34.638741000	27.809176000
1	-11.255211000	-35.844900000	27.260674000
7	-11.299769000	-39.360951000	29.776782000
6	-12.347263000	-40.356801000	29.983364000
6	-12.772364000	-40.985552000	28.653852000
6	-13.365236000	-40.009812000	27.616018000
8	-13.200035000	-38.761421000	27.839004000
8	-13.942846000	-40.495530000	26.614864000
1	-11.459581000	-38.780892000	28.958443000
1	-13.209411000	-39.901761000	30.485142000
1	-11.911179000	-41.477788000	28.180657000
1	-13.502577000	-41.781029000	28.839664000
8	-16.274776000	-41.583988000	32.447601000
1	-13.221360000	-45.704093000	30.258894000
1	-14.225190000	-44.402540000	28.071785000
1	-17.008115000	-45.669193000	36.628699000
1	-14.826040000	-46.406727000	35.193878000
1	-21.384223000	-42.970916000	35.581385000
1	-22.392248000	-41.733913000	33.370881000
1	-20.766523000	-41.004762000	28.479441000
1	-18.563306000	-41.715406000	27.056742000
6	-21.371933000	-46.533195000	32.275203000
1	-21.805043000	-46.896258000	31.336591000
1	-21.880800000	-47.042048000	33.107167000
1	-21.582691000	-45.461949000	32.348816000
1	-15.678789000	-33.578996000	35.909620000
1	-24.699083000	-35.855065000	32.645100000
1	-24.477075000	-36.270854000	28.021225000
1	-27.661037000	-38.978094000	31.323354000
1	-27.241534000	-39.805663000	26.975893000
1	-13.443673000	-41.262165000	19.654893000
1	-13.545374000	-41.417790000	24.110368000
1	-17.522098000	-37.709215000	22.526276000
1	-13.038022000	-38.421331000	22.574789000
1	-11.948963000	-41.122550000	30.655373000
1	-8.500079000	-38.515251000	30.216832000
1	-12.213112000	-37.315807000	32.776067000
1	-14.304626000	-40.973995000	34.420317000
1	-20.088598000	-33.904402000	38.744213000
1	-14.518135000	-28.872096000	26.341289000
1	-21.129630000	-25.965172000	27.948532000
1	-16.104874000	-24.735706000	27.200957000
8	-23.171178000	-39.755602000	32.088372000
1	-23.447509000	-39.195791000	31.330764000
1	-22.295796000	-39.413488000	32.328199000

^{2}TS1$_{HA,III}$

6	-20.558681000	-43.227778000	29.262256000
6	-21.215018000	-41.986859000	33.899714000
6	-16.430708000	-41.502955000	34.445144000
6	-15.784017000	-42.633526000	29.789890000
6	-21.158660000	-42.927615000	30.479626000
6	-22.580506000	-42.948541000	30.718706000
6	-22.767499000	-42.588461000	32.020744000
6	-21.454295000	-42.352979000	32.576000000
6	-19.961124000	-41.790675000	34.466578000
6	-19.736654000	-41.370266000	35.829773000
6	-18.390498000	-41.241799000	35.982400000
6	-17.790096000	-41.580447000	34.713367000
6	-15.832971000	-41.758933000	33.216138000
6	-14.411854000	-41.664261000	32.960562000
6	-14.229478000	-41.983650000	31.650600000
6	-15.539958000	-42.269479000	31.108450000
6	-17.040067000	-42.898510000	29.251274000
6	-17.249025000	-43.267592000	27.870150000
6	-18.590768000	-43.449344000	27.719085000
6	-19.193888000	-43.182091000	29.005257000
7	-20.488433000	-42.558723000	31.622350000
7	-18.763877000	-41.934337000	33.809879000
7	-16.496047000	-42.129307000	32.079139000
7	-18.231948000	-42.854703000	29.923479000
26	-18.509954000	-42.301074000	31.834572000
1	-22.081901000	-41.813472000	34.530563000
1	-15.780896000	-41.201769000	35.261535000
1	-14.938961000	-42.714030000	29.110945000
1	-21.212801000	-43.499091000	28.438734000
6	-16.201639000	-35.312027000	27.983962000
6	-17.918195000	-36.647745000	28.558399000
6	-19.052279000	-37.293006000	29.082365000
6	-17.337488000	-38.671712000	27.261422000
6	-17.071310000	-37.353912000	27.658272000
6	-17.348844000	-35.336843000	28.749495000
6	-19.284904000	-38.609715000	28.710208000
6	-18.455518000	-39.292907000	27.798793000
7	-16.037104000	-36.512577000	27.315687000
1	-15.217262000	-36.813877000	26.787647000
6	-17.946156000	-34.249115000	29.604180000
6	-16.937580000	-33.440064000	30.444851000
7	-17.625029000	-32.502093000	31.326508000
6	-17.850493000	-31.220413000	31.013449000
8	-17.425872000	-30.676633000	29.963679000
6	-18.653570000	-30.424276000	32.024939000
1	-18.086464000	-32.879252000	32.170094000
8	-20.367727000	-39.333955000	29.205777000
6	-20.524068000	-39.395662000	30.573078000
1	-15.484248000	-34.514633000	27.844195000
1	-19.721975000	-36.781214000	29.766827000
1	-16.683074000	-39.203889000	26.578251000
1	-18.692033000	-40.323596000	27.557050000
1	-18.667901000	-34.699328000	30.296548000
1	-18.520450000	-33.538552000	28.991834000
1	-16.326815000	-34.107484000	31.061372000
1	-16.268948000	-32.863941000	29.799854000
1	-18.029471000	-29.610516000	32.410370000
1	-19.021025000	-31.048276000	32.840946000
1	-19.507551000	-29.965929000	31.514042000
1	-21.469605000	-39.884498000	30.806576000
1	-20.354883000	-38.455205000	31.105780000
1	-19.544880000	-40.142838000	31.074979000
6	-19.541666000	-45.335887000	33.455553000
16	-18.051095000	-44.613955000	32.667631000
1	-20.124995000	-44.518737000	33.893182000
1	-19.202005000	-45.970451000	34.282293000
6	-16.771017000	-40.804535000	21.516116000
6	-15.246916000	-40.852482000	21.520274000
8	-14.647150000	-41.930315000	21.384092000
6	-17.418564000	-41.586570000	22.681272000
6	-18.904435000	-41.855821000	22.366943000
6	-17.224689000	-40.847191000	24.012850000
6	-19.623612000	-42.743188000	23.389868000
1	-17.130448000	-39.767001000	21.518104000
1	-16.905159000	-42.556546000	22.740383000
1	-19.433283000	-40.894165000	22.285852000
1	-18.974622000	-42.329145000	21.376706000
1	-17.580187000	-41.443571000	24.859661000
1	-16.174557000	-40.610219000	24.212016000
1	-17.787733000	-39.903421000	24.017014000
1	-20.651695000	-42.952783000	23.071174000
1	-19.109169000	-43.706045000	23.506808000
1	-19.673951000	-42.270894000	24.376389000
7	-14.617990000	-39.659145000	21.616237000
6	-13.173827000	-39.485640000	21.685428000
6	-12.767424000	-38.935841000	23.053084000
8	-13.536663000	-37.776465000	23.323390000
1	-15.140356000	-38.856357000	21.946383000
1	-12.870279000	-38.783622000	20.897179000
1	-12.946750000	-39.698943000	23.817885000
1	-11.687883000	-38.706921000	23.034540000
1	-13.783927000	-37.826870000	24.284604000
6	-12.669204000	-42.972491000	24.685078000
6	-12.129771000	-43.971411000	23.667212000
8	-11.411122000	-44.911646000	24.006370000
6	-13.677622000	-43.621650000	25.648902000
8	-14.039385000	-42.764855000	26.718011000
1	-11.819696000	-42.611404000	25.276071000
1	-14.563243000	-43.947067000	25.076597000
1	-13.226863000	-44.516833000	26.088996000
1	-14.238553000	-41.864780000	26.356908000
7	-12.498111000	-43.743198000	22.371095000
6	-12.102985000	-44.626848000	21.290722000
6	-13.290862000	-45.068503000	20.408117000
6	-14.299913000	-45.942085000	21.121327000
6	-14.121885000	-47.330251000	21.197697000
6	-15.423187000	-45.377153000	21.741745000
6	-15.036382000	-48.135844000	21.877583000
6	-16.339546000	-46.180397000	22.423698000
6	-16.149941000	-47.561970000	22.494781000
1	-13.149231000	-42.991708000	22.152570000
1	-11.614295000	-45.487918000	21.754258000
1	-13.787357000	-44.171444000	20.019163000
1	-12.876706000	-45.607358000	19.544718000
1	-13.254784000	-47.784022000	20.720625000
1	-15.567119000	-44.301198000	21.685359000
1	-14.879199000	-49.210954000	21.926193000
1	-17.203306000	-45.724876000	22.902392000
1	-16.862943000	-48.185934000	23.027952000

6	-27.414032000	-41.065875000	32.921446000
6	-27.742408000	-40.726261000	31.472177000
8	-28.493412000	-41.441675000	30.785571000
6	-27.371617000	-42.585928000	33.165121000
8	-28.626293000	-43.187208000	32.881223000
6	-27.004549000	-42.905968000	34.609490000
1	-26.461654000	-40.615657000	33.218049000
1	-26.591596000	-43.009813000	32.505274000
1	-28.871016000	-42.819931000	32.005059000
1	-26.976501000	-43.990976000	34.758506000
1	-26.026004000	-42.483618000	34.862632000
1	-27.755583000	-42.489054000	35.291471000
7	-27.181231000	-39.593894000	30.988564000
6	-27.419095000	-39.145968000	29.624132000
6	-26.417790000	-39.702182000	28.588817000
6	-24.978106000	-39.233753000	28.878139000
6	-26.884533000	-39.321376000	27.175957000
6	-23.898636000	-39.943751000	28.051716000
1	-26.561540000	-39.043254000	31.587918000
1	-27.386003000	-38.048186000	29.617152000
1	-26.450398000	-40.797406000	28.680370000
1	-24.915039000	-38.147367000	28.706966000
1	-24.763836000	-39.385818000	29.943559000
1	-26.229052000	-39.741127000	26.405989000
1	-27.900553000	-39.687318000	26.982852000
1	-26.890728000	-38.230273000	27.045496000
1	-22.895428000	-39.604680000	28.330761000
1	-23.936396000	-41.028694000	28.213378000
1	-24.018772000	-39.764091000	26.976763000
6	-26.337162000	-35.920906000	31.936367000
6	-25.386451000	-36.740725000	32.810801000
8	-25.394993000	-37.988532000	32.693135000
6	-26.259187000	-34.384254000	31.956884000
6	-25.010130000	-33.722024000	31.328513000
6	-24.825262000	-34.108788000	29.853340000
6	-25.110478000	-32.196044000	31.478086000
1	-26.205043000	-36.297953000	30.916080000
1	-27.144245000	-34.010989000	31.420237000
1	-26.375706000	-34.027120000	32.990217000
1	-24.114976000	-34.037659000	31.879008000
1	-23.961815000	-33.587649000	29.424527000
1	-24.650334000	-35.182633000	29.722768000
1	-25.709099000	-33.836579000	29.258835000
1	-24.219087000	-31.704196000	31.072751000
1	-25.986261000	-31.801935000	30.943014000
1	-25.197406000	-31.905332000	32.531579000
7	-24.624992000	-36.079407000	33.681291000
6	-23.550816000	-36.603963000	34.525651000
6	-22.302102000	-35.724674000	34.296974000
8	-22.780690000	-34.388344000	34.220800000
6	-21.530911000	-36.108825000	33.029916000
1	-24.591781000	-35.064933000	33.654543000
1	-23.374056000	-37.655387000	34.293583000
1	-21.640258000	-35.842234000	35.165850000
1	-22.009112000	-33.784302000	34.455466000
1	-20.726338000	-35.384079000	32.869032000
1	-21.090990000	-37.110431000	33.126859000
1	-22.190464000	-36.099616000	32.151759000
6	-17.121323000	-24.596212000	31.131565000
6	-15.668545000	-24.524520000	30.651603000
8	-15.334777000	-23.794408000	29.723270000
6	-17.949414000	-25.591680000	30.301496000
6	-17.546798000	-27.051176000	30.546479000
8	-16.834459000	-27.367579000	31.514340000
7	-18.001933000	-27.954814000	29.662127000
1	-17.177865000	-24.875439000	32.188017000
1	-19.012795000	-25.485595000	30.556872000
1	-17.856607000	-25.355170000	29.234261000
1	-18.580515000	-27.665457000	28.886599000
1	-17.792731000	-28.968574000	29.785637000
7	-14.800216000	-25.336593000	31.332143000
6	-13.452520000	-25.582149000	30.851255000
6	-13.229414000	-26.831022000	29.971216000
8	-12.194982000	-26.916649000	29.312765000
1	-15.236371000	-26.023458000	31.938011000
1	-13.156258000	-24.722189000	30.248966000
7	-14.184559000	-27.793581000	30.010675000
6	-14.123920000	-28.977120000	29.159146000
6	-14.648138000	-28.686900000	27.735295000
6	-14.645054000	-29.899728000	26.829869000
6	-15.608281000	-30.910789000	26.982999000
6	-13.686176000	-30.037705000	25.817392000
6	-15.616997000	-32.018053000	26.132388000
6	-13.690938000	-31.147084000	24.967752000
6	-14.660524000	-32.140128000	25.120057000
1	-15.027074000	-27.661159000	30.560921000
1	-14.729102000	-29.757599000	29.629268000
1	-15.668895000	-28.294069000	27.826536000
1	-14.029606000	-27.892257000	27.303706000
1	-16.348869000	-30.835690000	27.776987000
1	-12.933221000	-29.262428000	25.689964000
1	-16.373164000	-32.788297000	26.260044000
1	-12.942339000	-31.231022000	24.183262000

1	-14.672724000	-33.001343000	24.456880000
6	-18.678212000	-33.867042000	36.969140000
6	-18.653594000	-35.382070000	36.745350000
8	-19.628637000	-36.104110000	36.954887000
6	-18.416673000	-33.037000000	35.695399000
6	-19.560627000	-33.184246000	34.663634000
8	-20.722765000	-33.060315000	35.138399000
8	-19.254886000	-33.404069000	33.456675000
1	-19.670202000	-33.618021000	37.348847000
1	-18.359085000	-31.977742000	35.983233000
1	-17.461152000	-33.295949000	35.227058000
7	-17.451172000	-35.870197000	36.327212000
6	-17.228972000	-37.233428000	35.881419000
6	-16.485935000	-37.228339000	34.536019000
8	-15.335619000	-36.401075000	34.739885000
6	-17.352301000	-36.749971000	33.370531000
1	-16.704466000	-35.233433000	36.078251000
1	-18.197101000	-37.734594000	35.807065000
1	-16.165794000	-38.263763000	34.330336000
1	-15.034628000	-36.040142000	33.876848000
1	-16.756099000	-36.703725000	32.452479000
1	-18.172980000	-37.455648000	33.198071000
1	-17.776852000	-35.757482000	33.557241000
6	-12.944485000	-34.655955000	30.539615000
6	-13.317423000	-35.591804000	31.677829000
8	-14.299948000	-35.338793000	32.391537000
1	-12.970080000	-33.626965000	30.909216000
1	-13.703846000	-34.740015000	29.754139000
7	-12.519416000	-36.670856000	31.828379000
6	-12.743832000	-37.723559000	32.813209000
6	-11.416553000	-38.223257000	33.386316000
1	-11.799158000	-36.838661000	31.121422000
1	-13.289103000	-38.559965000	32.350863000
1	-11.592473000	-39.044407000	34.091053000
1	-10.756033000	-38.591906000	32.592322000
1	-10.893128000	-37.420034000	33.916779000
6	-9.711086000	-36.636601000	27.520031000
6	-10.774327000	-37.183798000	28.478394000
8	-10.561915000	-37.151989000	29.702547000
6	-10.128747000	-36.253289000	26.090067000
6	-11.105313000	-35.064507000	25.953699000
6	-10.498979000	-33.749954000	26.468411000
6	-11.557867000	-34.920964000	24.492243000
1	-9.249622000	-35.789679000	28.037299000
1	-9.214245000	-36.012425000	25.527914000
1	-10.556247000	-37.129568000	25.582573000
1	-11.998183000	-35.280886000	26.557196000
1	-11.211960000	-32.925468000	26.355086000
1	-10.226189000	-33.803221000	27.528540000
1	-9.593513000	-33.489575000	25.902225000
1	-12.240306000	-34.069964000	24.381729000
1	-10.695304000	-34.741462000	23.834432000
1	-12.078955000	-35.817202000	24.135598000
7	-11.867096000	-37.738176000	27.922259000
6	-12.969517000	-38.347963000	28.660932000
6	-13.555933000	-39.530525000	27.885364000
6	-13.955940000	-39.235838000	26.426650000
8	-13.841327000	-38.030764000	26.008941000
8	-14.356586000	-40.202611000	25.733869000
1	-12.082292000	-37.596311000	26.940561000
1	-13.749468000	-37.601027000	28.853366000
1	-12.853317000	-40.372593000	27.862274000
1	-14.448879000	-39.895140000	28.408379000
8	-18.519093000	-40.602725000	31.495755000
1	-13.669484000	-41.390396000	33.699831000
1	-13.307432000	-42.029312000	31.085026000
1	-20.520226000	-41.178595000	36.551631000
1	-17.839522000	-40.924960000	36.858872000
1	-23.695092000	-42.485401000	32.569373000
1	-23.327520000	-43.208840000	29.979320000
1	-19.138971000	-43.730544000	26.828228000
1	-16.446240000	-43.348210000	27.148092000
6	-20.409315000	-46.144444000	32.485677000
1	-19.842488000	-46.974159000	32.048412000
1	-21.277236000	-46.565192000	33.012332000
1	-20.773094000	-45.515566000	31.668562000
1	-16.619978000	-37.775448000	36.620791000
1	-23.868664000	-36.531193000	35.572839000
1	-27.347873000	-36.235106000	32.230939000
1	-28.201992000	-40.638048000	33.558070000
1	-28.432285000	-39.454985000	29.349208000
1	-11.360762000	-44.129437000	20.646866000
1	-13.135051000	-42.107745000	24.201293000
1	-17.079976000	-41.252323000	20.562211000
1	-12.703169000	-40.451358000	21.488957000
1	-12.584935000	-38.679162000	29.627794000
1	-8.931434000	-37.410287000	27.470154000
1	-11.962138000	-34.873736000	30.113360000
1	-13.382998000	-37.324093000	33.602912000
1	-17.931903000	-33.616846000	37.737667000
1	-13.085111000	-29.315049000	29.111618000
1	-17.543872000	-23.594570000	31.011049000
1	-12.761970000	-25.650682000	31.700628000

8	-24.079480000	-40.155715000	33.881829000
1	-24.557978000	-39.346298000	33.598183000
1	-23.440811000	-40.295872000	33.166100000

$^{4}\mathbf{TS1}_{\mathbf{HA,III}}$

6	-20.521133000	-43.230500000	29.211832000
6	-21.175829000	-41.988201000	33.845025000
6	-16.389220000	-41.486115000	34.381660000
6	-15.737397000	-42.726822000	29.755538000
6	-21.124068000	-42.911274000	30.423249000
6	-22.548353000	-42.903275000	30.651747000
6	-22.735878000	-42.547650000	31.955047000
6	-21.419589000	-42.343068000	32.516384000
6	-19.922190000	-41.801923000	34.409508000
6	-19.694678000	-41.378375000	35.769123000
6	-18.349105000	-41.235102000	35.918239000
6	-17.745958000	-41.571142000	34.652212000
6	-15.789935000	-41.767959000	33.157300000
6	-14.367019000	-41.694648000	32.904958000
6	-14.184232000	-42.049873000	31.604338000
6	-15.495774000	-42.334834000	31.065044000
6	-16.995746000	-42.989394000	29.217186000
6	-17.207613000	-43.351667000	27.835174000
6	-18.552709000	-43.505119000	27.680512000
6	-19.153017000	-43.227590000	28.965646000
7	-20.454404000	-42.563793000	31.571228000
7	-18.719880000	-41.939074000	33.747189000
7	-16.455207000	-42.156987000	32.029334000
7	-18.185571000	-42.929951000	29.888943000
26	-18.459616000	-42.294403000	31.782670000
1	-22.040241000	-41.806898000	34.477038000
1	-15.740566000	-41.170928000	35.193592000
1	-14.891727000	-42.821300000	29.079074000
1	-21.175467000	-43.481508000	28.381969000
6	-16.199945000	-35.308317000	27.994760000
6	-17.941248000	-36.619644000	28.553462000
6	-19.091830000	-37.247883000	29.064292000
6	-17.368075000	-38.658019000	27.278014000
6	-17.092030000	-37.340779000	27.668661000
6	-17.357072000	-35.315653000	28.746254000
6	-19.335910000	-38.563536000	28.699094000
6	-18.499783000	-39.262824000	27.806484000
7	-16.042400000	-36.513773000	27.334232000
1	-15.222038000	-36.827638000	26.815349000
6	-17.950315000	-34.218599000	29.592089000
6	-16.941755000	-33.422683000	30.445299000
7	-17.626446000	-32.480178000	31.324811000
6	-17.849671000	-31.199290000	31.007161000
8	-17.426203000	-30.661118000	29.954016000
6	-18.648857000	-30.397085000	32.016960000
1	-18.085061000	-32.851651000	32.171981000
8	-20.438154000	-39.265684000	29.186421000
6	-20.561020000	-39.368207000	30.561057000
1	-15.471311000	-34.520233000	27.860198000
1	-19.765003000	-36.725994000	29.737907000
1	-16.711200000	-39.201995000	26.606448000
1	-18.743959000	-40.292893000	27.569250000
1	-18.686424000	-34.659163000	30.275504000
1	-18.507616000	-33.500341000	28.972977000
1	-16.343453000	-34.097920000	31.065363000
1	-16.261008000	-32.851695000	29.808342000
1	-18.024168000	-29.579608000	32.393449000
1	-19.011399000	-31.015976000	32.839058000
1	-19.506099000	-29.943688000	31.507020000
1	-21.507451000	-39.854037000	30.799041000
1	-20.377676000	-38.439728000	31.110662000
1	-19.541704000	-40.149869000	31.017312000
6	-19.664810000	-45.414081000	33.331532000
16	-18.084119000	-44.633598000	32.841082000
1	-20.322216000	-44.615783000	33.696394000
1	-19.461439000	-46.079227000	34.178463000
6	-16.792012000	-40.827950000	21.551595000
6	-15.268042000	-40.877702000	21.547979000
8	-14.670212000	-41.955779000	21.404207000
6	-17.434878000	-41.613424000	22.717013000
6	-18.923560000	-41.875748000	22.410135000
6	-17.230409000	-40.881359000	24.051108000
6	-19.639635000	-42.766667000	23.432133000
1	-17.150016000	-39.789969000	21.559395000
1	-16.924601000	-42.585564000	22.767940000
1	-19.449528000	-40.911827000	22.338284000
1	-19.001593000	-42.342871000	21.417520000
1	-17.583445000	-41.480256000	24.897157000
1	-16.178210000	-40.649732000	24.245777000
1	-17.789481000	-39.935386000	24.063300000
1	-20.670266000	-42.971139000	23.118328000
1	-19.127576000	-43.731879000	23.540398000
1	-19.682607000	-42.299561000	24.421454000
7	-14.637433000	-39.685636000	21.646027000
6	-13.192918000	-39.513210000	21.709819000
6	-12.781847000	-38.964891000	23.076845000
8	-13.552364000	-37.807763000	23.352680000
1	-15.157336000	-38.883545000	21.981902000
1	-12.891937000	-38.810479000	20.921196000
1	-12.956308000	-39.729516000	23.841255000
1	-11.702929000	-38.733469000	23.054050000
1	-13.800617000	-37.864006000	24.313617000
6	-12.645521000	-43.002241000	24.676349000
6	-12.114388000	-43.992522000	23.645805000
8	-11.386319000	-44.930790000	23.970354000
6	-13.642117000	-43.662137000	25.645103000
8	-13.994725000	-42.816269000	26.725666000
1	-11.791087000	-42.643367000	25.261476000
1	-14.532824000	-43.984916000	25.079105000
1	-13.184350000	-44.560046000	26.072367000
1	-14.206834000	-41.914757000	26.374529000
7	-12.502161000	-43.760138000	22.356231000
6	-12.118527000	-44.637184000	21.266349000
6	-13.316695000	-45.079045000	20.397838000
6	-14.312870000	-45.959942000	21.120162000
6	-14.128943000	-47.347860000	21.186623000
6	-15.429764000	-45.402361000	21.758575000
6	-15.031423000	-48.160429000	21.874227000
6	-16.333996000	-46.212599000	22.448410000
6	-16.138605000	-47.593861000	22.509410000
1	-13.159841000	-43.010684000	22.150191000
1	-11.620286000	-45.498578000	21.719005000
1	-13.821662000	-44.182011000	20.020031000
1	-12.912259000	-45.612252000	19.526367000
1	-13.266795000	-47.795892000	20.695335000
1	-15.578397000	-44.326674000	21.709740000
1	-14.869913000	-49.235247000	21.914709000
1	-17.192925000	-45.762732000	22.940952000
1	-16.842332000	-48.223288000	23.048488000
6	-27.357565000	-41.078227000	32.975871000
6	-27.712653000	-40.732297000	31.534390000
8	-28.468169000	-41.450905000	30.855976000
6	-27.287656000	-42.599590000	33.205117000
8	-28.537384000	-43.217726000	32.936468000
6	-26.891797000	-42.927146000	34.640174000
1	-26.408142000	-40.615542000	33.262150000
1	-26.512174000	-43.005363000	32.528650000
1	-28.803440000	-42.845131000	32.068700000
1	-26.847079000	-44.012983000	34.778862000
1	-25.914496000	-42.494448000	34.880370000
1	-27.636331000	-42.526262000	35.338675000
7	-27.169390000	-39.592389000	31.048614000
6	-27.433378000	-39.137972000	29.690974000
6	-26.444586000	-39.678434000	28.635492000
6	-25.003571000	-39.203639000	28.906594000
6	-26.936231000	-39.288113000	27.233708000
6	-23.932764000	-39.891426000	28.050610000
1	-26.547582000	-39.037985000	31.642308000
1	-27.409612000	-38.039963000	29.691045000
1	-26.468897000	-40.774668000	28.717320000
1	-24.951525000	-38.114172000	28.752028000
1	-24.769459000	-39.371080000	29.965535000
1	-26.292063000	-39.699423000	26.449808000
1	-27.954062000	-39.656226000	27.054458000
1	-26.948432000	-38.196052000	27.111950000
1	-22.927307000	-39.554511000	28.323877000
1	-23.964764000	-40.979874000	28.189262000
1	-24.068354000	-39.689368000	26.981478000
6	-26.340011000	-35.911763000	31.991839000
6	-25.378881000	-36.726788000	32.859325000
8	-25.383729000	-37.974788000	32.743307000
6	-26.268482000	-34.374741000	32.010794000
6	-25.027224000	-33.707476000	31.372323000
6	-24.851051000	-34.095737000	29.896486000
6	-25.133972000	-32.181788000	31.520399000
1	-26.213891000	-36.288791000	30.970797000
1	-27.159238000	-34.005750000	31.480664000
1	-26.378604000	-34.017356000	33.044749000
1	-24.126577000	-34.017938000	31.916774000
1	-23.993689000	-33.570560000	29.460485000
1	-24.670872000	-35.168756000	29.766470000
1	-25.740739000	-33.829541000	29.307997000
1	-24.247829000	-31.686216000	31.108173000
1	-26.015358000	-31.792789000	30.990806000
1	-25.215049000	-31.889932000	32.574053000
7	-24.612582000	-36.061382000	33.722327000
6	-23.530018000	-36.580821000	34.559012000
6	-22.288615000	-35.692541000	34.325291000
8	-22.776651000	-34.359112000	34.256698000
6	-21.523158000	-36.067376000	33.052194000
1	-24.584456000	-35.046736000	33.695214000
1	-23.347054000	-37.630221000	34.322477000
1	-21.621107000	-35.808359000	35.190096000
1	-22.006635000	-33.750714000	34.484397000
1	-20.722977000	-35.338259000	32.889180000
1	-21.077854000	-37.067294000	33.141092000
1	-22.187815000	-36.057771000	32.178018000
6	-17.142296000	-24.568698000	31.072132000
6	-15.690121000	-24.492474000	30.590886000
8	-15.360123000	-23.764701000	29.659354000
6	-17.965627000	-25.575368000	30.250827000

6	-17.556526000	-27.031036000	30.508111000
8	-16.840519000	-27.335794000	31.477112000
7	-18.010013000	-27.944329000	29.633046000
1	-17.196542000	-24.839769000	32.130843000
1	-19.029484000	-25.471944000	30.505327000
1	-17.874105000	-25.347718000	29.181542000
1	-18.591923000	-27.664209000	28.856609000
1	-17.796755000	-28.956369000	29.764908000
7	-14.818007000	-25.297926000	31.274414000
6	-13.469748000	-25.540548000	30.793661000
6	-13.241577000	-26.795042000	29.922952000
8	-12.207241000	-26.880763000	29.264334000
1	-15.251404000	-25.983859000	31.883421000
1	-13.178573000	-24.683707000	30.184466000
7	-14.192188000	-27.761690000	29.970570000
6	-14.126893000	-28.951772000	29.128493000
6	-14.657123000	-28.676661000	27.703890000
6	-14.650013000	-29.897431000	26.809229000
6	-15.607821000	-30.911838000	26.973649000
6	-13.692508000	-30.039775000	25.796073000
6	-15.612868000	-32.026857000	26.133235000
6	-13.693545000	-31.156842000	24.956569000
6	-14.657965000	-32.153165000	25.119924000
1	-15.034587000	-27.629236000	30.520972000
1	-14.725871000	-29.732083000	29.606805000
1	-15.679772000	-28.288747000	27.795005000
1	-14.044386000	-27.882325000	27.263497000
1	-16.347062000	-30.833303000	27.768571000
1	-12.943632000	-29.261984000	25.660068000
1	-16.364352000	-32.800072000	26.270012000
1	-12.946120000	-31.244271000	24.171318000
1	-14.667542000	-33.020332000	24.464518000
6	-18.673910000	-33.808075000	36.992855000
6	-18.650340000	-35.324288000	36.778081000
8	-19.624488000	-36.045114000	36.995964000
6	-18.412593000	-32.985393000	35.714297000
6	-19.556417000	-33.138384000	34.682775000
8	-20.718800000	-33.017842000	35.157930000
8	-19.249752000	-33.358593000	33.476328000
1	-19.665694000	-33.556587000	37.371427000
1	-18.355077000	-31.924497000	35.996109000
1	-17.456919000	-33.246455000	35.247434000
7	-17.449906000	-35.815920000	36.357861000
6	-17.233427000	-37.181351000	35.916223000
6	-16.495319000	-37.183753000	34.568311000
8	-15.337661000	-36.366354000	34.767709000
6	-17.361300000	-36.698884000	33.405251000
1	-16.703413000	-35.181446000	36.102558000
1	-18.203820000	-37.678934000	35.847056000
1	-16.184868000	-38.222392000	34.362575000
1	-15.034565000	-36.011227000	33.902776000
1	-16.769726000	-36.664312000	32.483796000
1	-18.193348000	-37.392904000	33.239920000
1	-17.772033000	-35.700284000	33.589778000
6	-12.953445000	-34.667748000	30.547185000
6	-13.324514000	-35.591433000	31.695752000
8	-14.300722000	-35.326119000	32.413590000
1	-12.973021000	-33.635296000	30.907339000
1	-13.717111000	-34.756160000	29.766386000
7	-12.532234000	-36.674253000	31.850139000
6	-12.756136000	-37.717273000	32.844980000
6	-11.428735000	-38.210958000	33.423284000
1	-11.816243000	-36.851713000	31.141110000
1	-13.300518000	-38.558341000	32.390065000
1	-11.604225000	-39.025332000	34.136063000
1	-10.768002000	-38.586780000	32.632861000
1	-10.905822000	-37.402373000	33.946036000
6	-9.732739000	-36.673599000	27.543620000
6	-10.796504000	-37.216651000	28.503855000
8	-10.581852000	-37.184549000	29.727744000
6	-10.150455000	-36.290672000	26.113579000
6	-11.122574000	-35.098355000	25.976394000
6	-10.511387000	-33.785548000	26.489812000
6	-11.574749000	-34.954450000	24.514863000
1	-9.268181000	-35.827484000	28.059459000
1	-9.235600000	-36.054112000	25.550146000
1	-10.581908000	-37.165954000	25.607715000
1	-12.016088000	-35.310929000	26.580313000
1	-11.220990000	-32.958394000	26.374724000
1	-10.239591000	-33.838428000	27.550223000
1	-9.604481000	-33.529467000	25.923947000
1	-12.254247000	-34.101195000	24.403720000
1	-10.711551000	-34.778340000	23.856937000
1	-12.098795000	-35.849278000	24.158997000
7	-11.891866000	-37.767387000	27.949813000
6	-12.994208000	-38.377616000	28.688605000
6	-13.573607000	-39.566062000	27.916458000
6	-13.965882000	-39.279499000	26.453946000
8	-13.860127000	-38.074462000	26.034266000
8	-14.349957000	-40.252672000	25.760586000
1	-12.106297000	-37.628664000	26.967466000
1	-13.777433000	-37.632580000	28.874557000
1	-12.867981000	-40.405830000	27.901050000
1	-14.468411000	-39.930328000	28.436535000
8	-18.500084000	-40.622184000	31.331443000
1	-13.623893000	-41.411735000	33.640025000
1	-13.261005000	-42.120966000	31.043411000
1	-20.477568000	-41.189742000	36.492507000
1	-17.800746000	-40.908614000	36.792783000
1	-23.663704000	-42.426092000	32.499413000
1	-23.295925000	-43.142911000	29.905976000
1	-19.104729000	-43.768746000	26.786676000
1	-16.405775000	-43.441616000	27.113539000
6	-20.346280000	-46.182290000	32.194705000
1	-19.710435000	-46.998808000	31.834315000
1	-21.292158000	-46.616991000	32.545280000
1	-20.559749000	-45.520418000	31.351626000
1	-16.623583000	-37.722989000	36.655233000
1	-23.842106000	-36.513287000	35.608324000
1	-27.347055000	-36.230165000	32.294365000
1	-28.142793000	-40.668687000	33.627657000
1	-28.448301000	-39.453761000	29.430416000
1	-11.387043000	-44.133962000	20.614770000
1	-13.119001000	-42.135252000	24.204177000
1	-17.106419000	-41.271705000	20.597569000
1	-12.723229000	-40.478832000	21.510570000
1	-12.611068000	-38.702612000	29.658150000
1	-8.955451000	-37.449688000	27.493915000
1	-11.974084000	-34.893585000	30.118266000
1	-13.395712000	-37.310510000	33.630594000
1	-17.927202000	-33.553938000	37.759747000
1	-13.086190000	-29.283728000	29.080709000
1	-17.570485000	-23.570422000	30.943919000
1	-12.777620000	-25.599178000	31.642518000
8	-24.007644000	-40.134942000	33.869635000
1	-24.507800000	-39.330166000	33.610654000
1	-23.373158000	-40.241507000	33.144467000

^{2}IM1$_{HA,III}$

6	-19.595266000	-43.588009000	28.443801000
6	-20.213934000	-41.398719000	32.717187000
6	-15.459797000	-41.788411000	33.556312000
6	-14.838393000	-43.929974000	29.267619000
6	-20.179319000	-42.942638000	29.527671000
6	-21.585739000	-42.633343000	29.638844000
6	-21.759984000	-42.014730000	30.839687000
6	-20.456669000	-41.949839000	31.458254000
6	-18.971156000	-41.346104000	33.337758000
6	-18.721804000	-40.738521000	34.622607000
6	-17.384590000	-40.847302000	34.854674000
6	-16.812542000	-41.522436000	33.714056000
6	-14.873043000	-42.424316000	32.465737000
6	-13.454605000	-42.671337000	32.329117000
6	-13.279108000	-43.279104000	31.123640000
6	-14.590411000	-43.402507000	30.529023000
6	-16.093186000	-44.028284000	28.673099000
6	-16.317095000	-44.559770000	27.349815000
6	-17.658693000	-44.488194000	27.126996000
6	-18.243367000	-43.891225000	28.306312000
7	-19.507431000	-42.517053000	30.647806000
7	-17.799624000	-41.834915000	32.806100000
7	-15.543430000	-42.885316000	31.366338000
7	-17.275412000	-43.633861000	29.238070000
26	-17.520469000	-42.668339000	30.980316000
1	-21.060554000	-40.954325000	33.234087000
1	-14.798502000	-41.469987000	34.357075000
1	-13.988547000	-44.230692000	28.663564000
1	-20.249429000	-43.862841000	27.620945000
6	-16.440794000	-35.827505000	28.944590000
6	-18.525567000	-36.636892000	29.136099000
6	-19.885188000	-36.911115000	29.366491000
6	-18.229952000	-38.846268000	28.077053000
6	-17.714199000	-37.607830000	28.482954000
6	-17.690599000	-35.502889000	29.429652000
6	-20.381812000	-38.143883000	28.965510000
6	-19.572691000	-39.106990000	28.327360000
7	-16.447036000	-37.084891000	28.375543000
1	-15.644698000	-37.552825000	27.936074000
6	-18.149980000	-34.253706000	30.130418000
6	-17.044057000	-33.454238000	30.841686000
7	-17.605763000	-32.326219000	31.577244000
6	-17.776056000	-31.114546000	31.031965000
8	-17.380568000	-30.815100000	29.879707000
6	-18.474226000	-30.088629000	31.905722000
1	-18.084139000	-32.521526000	32.469416000
8	-21.721542000	-38.492971000	29.136024000
6	-22.374032000	-38.105122000	30.259871000
1	-15.528770000	-35.246320000	28.952727000
1	-20.531533000	-36.171630000	29.827728000
1	-17.599270000	-39.593548000	27.606881000
1	-20.017596000	-40.057066000	28.050039000
1	-18.904421000	-34.523069000	30.881279000
1	-18.655081000	-33.574932000	29.427309000
1	-16.499364000	-34.088850000	31.548766000
1	-16.327653000	-33.057590000	30.117347000
1	-17.804503000	-29.234234000	32.051972000

1	-18.784629000	-30.508142000	32.864323000
1	-19.361401000	-29.720702000	31.378302000
1	-23.451533000	-38.084597000	30.156039000
1	-21.861346000	-37.459326000	30.963054000
1	-17.943922000	-40.526754000	30.244894000
6	-19.357468000	-44.994959000	33.005688000
16	-17.674471000	-44.747549000	32.328154000
1	-19.792186000	-44.008537000	33.199503000
1	-19.252670000	-45.502339000	33.971195000
6	-17.225044000	-40.112413000	22.347494000
6	-15.713216000	-40.017069000	22.176138000
8	-15.072610000	-40.953221000	21.671456000
6	-17.667450000	-41.271465000	23.268041000
6	-19.175660000	-41.536025000	23.083389000
6	-17.295694000	-40.985570000	24.730187000
6	-19.702585000	-42.767655000	23.829307000
1	-17.638258000	-39.164581000	22.716683000
1	-17.124260000	-42.167697000	22.936692000
1	-19.737924000	-40.647787000	23.408108000
1	-19.385354000	-41.656685000	22.010422000
1	-17.506260000	-41.844112000	25.376322000
1	-16.232915000	-40.754951000	24.856817000
1	-17.871310000	-40.131693000	25.114034000
1	-20.763429000	-42.935688000	23.607684000
1	-19.153471000	-43.671292000	23.532910000
1	-19.603613000	-42.658876000	24.914357000
7	-15.149153000	-38.847403000	22.549957000
6	-13.719826000	-38.569345000	22.531404000
6	-13.200216000	-38.374362000	23.956583000
8	-13.997699000	-37.400512000	24.610055000
1	-15.677762000	-38.214689000	23.138858000
1	-13.550380000	-37.655926000	21.945645000
1	-13.250560000	-39.325730000	24.498265000
1	-12.145629000	-38.054503000	23.907118000
1	-14.210520000	-37.778259000	25.507457000
6	-12.460438000	-42.576317000	24.233913000
6	-12.005484000	-43.147879000	22.896001000
8	-11.133910000	-44.015948000	22.829766000
6	-13.098670000	-43.652320000	25.129315000
8	-13.292840000	-43.216379000	26.460278000
1	-11.571231000	-42.196793000	24.750980000
1	-14.045244000	-43.986094000	24.667815000
1	-12.430865000	-44.519332000	25.167731000
1	-13.741941000	-42.335356000	26.447198000
7	-12.623786000	-42.628754000	21.793947000
6	-12.320328000	-43.090966000	20.453051000
6	-13.583560000	-43.425931000	19.630686000
6	-14.375756000	-44.600847000	20.162011000
6	-14.056919000	-45.912895000	19.785731000
6	-15.433567000	-44.401713000	21.060649000
6	-14.771218000	-46.999778000	20.291908000
6	-16.149741000	-45.487128000	21.569330000
6	-15.822142000	-46.789753000	21.187395000
1	-13.383235000	-41.958865000	21.903280000
1	-11.676412000	-43.967649000	20.562687000
1	-14.222411000	-42.535555000	19.595532000
1	-13.260274000	-43.631108000	18.600728000
1	-13.238132000	-46.084014000	19.088839000
1	-15.685714000	-43.386672000	21.356941000
1	-14.507542000	-48.010067000	19.987075000
1	-16.965314000	-45.313346000	22.267502000
1	-16.380118000	-47.634199000	21.584741000
6	-26.538971000	-41.145008000	33.192729000
6	-27.324796000	-40.867264000	31.914843000
8	-28.169522000	-41.665590000	31.472925000
6	-26.361463000	-42.653498000	33.447895000
8	-27.618878000	-43.300259000	33.584048000
6	-25.556661000	-42.915690000	34.715229000
1	-25.561372000	-40.653079000	33.149362000
1	-25.809286000	-43.076643000	32.589307000
1	-28.124058000	-42.996448000	32.800536000
1	-25.455494000	-43.994401000	34.876561000
1	-24.557299000	-42.473039000	34.642634000
1	-26.068927000	-42.488836000	35.585868000
7	-27.053059000	-39.694809000	31.296253000
6	-27.721497000	-39.311530000	30.061117000
6	-27.077366000	-39.899249000	28.785613000
6	-25.609035000	-39.458064000	28.648891000
6	-27.927178000	-39.520466000	27.564180000
6	-24.817787000	-40.147794000	27.531344000
1	-26.364351000	-39.057030000	31.700483000
1	-27.716199000	-38.215004000	30.006680000
1	-27.100765000	-40.992145000	28.899361000
1	-25.574595000	-38.367243000	28.498711000
1	-25.102610000	-39.653702000	29.601576000
1	-27.545358000	-39.980401000	26.646541000
1	-28.967248000	-39.846492000	27.689320000
1	-27.933534000	-38.432214000	27.411151000
1	-23.767086000	-39.840018000	27.563738000
1	-24.851550000	-41.239269000	27.644389000
1	-25.208046000	-39.904631000	26.536057000
6	-26.319484000	-35.789996000	31.887913000
6	-25.260491000	-36.551270000	32.687225000

8	-25.151355000	-37.787349000	32.513243000
6	-26.307184000	-34.251213000	31.882698000
6	-25.095530000	-33.548309000	31.226881000
6	-24.857938000	-34.014208000	29.782554000
6	-25.296643000	-32.025994000	31.281504000
1	-26.256089000	-36.178133000	30.865452000
1	-27.215229000	-33.927422000	31.352392000
1	-26.424436000	-33.878789000	32.910537000
1	-24.193213000	-33.771746000	31.809829000
1	-24.038531000	-33.447673000	29.325641000
1	-24.586125000	-35.074324000	29.727082000
1	-25.753962000	-33.861467000	29.164277000
1	-24.427951000	-31.501702000	30.867578000
1	-26.181052000	-31.721572000	30.703965000
1	-25.430693000	-31.680220000	32.313241000
7	-24.530406000	-35.857497000	33.559638000
6	-23.417989000	-36.333636000	34.381394000
6	-22.287978000	-35.284574000	34.299615000
8	-22.926001000	-34.016975000	34.348406000
6	-21.440808000	-35.423687000	33.030962000
1	-24.608074000	-34.845953000	33.605054000
1	-23.094283000	-37.316217000	34.032394000
1	-21.643524000	-35.416472000	35.180585000
1	-22.218396000	-33.346029000	34.597288000
1	-20.714846000	-34.604670000	32.992064000
1	-20.897293000	-36.377715000	33.016791000
1	-22.076718000	-35.368441000	32.137067000
6	-16.999332000	-24.649420000	29.405570000
6	-15.600966000	-24.728159000	28.785464000
8	-15.357599000	-24.238434000	27.686588000
6	-17.898455000	-25.809660000	28.945661000
6	-17.468832000	-27.164433000	29.522444000
8	-16.667711000	-27.236931000	30.469796000
7	-18.003948000	-28.255497000	28.949644000
1	-16.948461000	-24.647060000	30.498670000
1	-18.932636000	-25.619080000	29.264192000
1	-17.911507000	-25.860526000	27.849835000
1	-18.644344000	-28.166647000	28.173863000
1	-17.771156000	-29.206024000	29.307538000
7	-14.674037000	-25.386498000	29.548936000
6	-13.377662000	-25.765709000	29.018081000
6	-13.219087000	-27.204749000	28.482599000
8	-12.232935000	-27.475547000	27.800671000
1	-15.049139000	-25.896221000	30.342155000
1	-13.158054000	-25.097979000	28.183572000
7	-14.169964000	-28.106761000	28.833047000
6	-14.160144000	-29.475109000	28.323756000
6	-14.818107000	-29.583102000	26.930206000
6	-14.806651000	-30.993066000	26.378590000
6	-15.692773000	-31.965003000	26.870526000
6	-13.898840000	-31.364044000	25.377586000
6	-15.668154000	-33.269344000	26.370746000
6	-13.872290000	-32.668970000	24.878872000
6	-14.756005000	-33.630832000	25.374198000
1	-14.964335000	-27.823888000	29.397188000
1	-14.697934000	-30.103300000	29.040009000
1	-15.849521000	-29.216723000	27.012663000
1	-14.284355000	-28.908377000	26.251525000
1	-16.397149000	-31.700051000	27.657412000
1	-13.206124000	-30.620833000	24.987048000
1	-16.359925000	-34.007945000	26.766746000
1	-13.159119000	-32.935944000	24.102332000
1	-14.721744000	-34.651102000	24.998910000
6	-19.100722000	-32.880816000	37.357689000
6	-19.068218000	-34.411884000	37.339988000
8	-20.033647000	-35.100770000	37.668704000
6	-18.750856000	-32.213423000	36.013536000
6	-19.790067000	-32.530953000	34.910021000
8	-20.993677000	-32.499356000	35.283768000
8	-19.361346000	-32.781662000	33.747600000
1	-20.116906000	-32.597395000	37.635061000
1	-18.742741000	-31.124137000	36.163796000
1	-17.750701000	-32.488890000	35.660630000
7	-17.873663000	-34.956753000	36.968573000
6	-17.665645000	-36.377446000	36.760638000
6	-16.898955000	-36.616051000	35.453398000
8	-15.704018000	-35.843386000	35.565126000
6	-17.708625000	-36.263153000	34.204642000
1	-17.122147000	-34.367406000	36.634839000
1	-18.644684000	-36.863004000	36.748683000
1	-16.646817000	-37.691087000	35.412085000
1	-15.324039000	-35.707347000	34.668501000
1	-17.096454000	-36.400559000	33.306683000
1	-18.582224000	-36.920436000	34.120271000
1	-18.058449000	-35.225607000	34.227400000
6	-12.930511000	-35.279183000	31.297661000
6	-13.560600000	-35.980907000	32.489255000
8	-14.501982000	-35.448092000	33.097475000
1	-12.843487000	-34.213374000	31.523992000
1	-13.595204000	-35.380345000	30.432509000
7	-13.030107000	-37.180141000	32.806777000
6	-13.546071000	-38.032431000	33.872299000
6	-12.427451000	-38.896408000	34.455245000

1	-12.331077000	-37.588516000	32.179752000
1	-14.351484000	-38.673778000	33.484558000
1	-12.825199000	-39.558071000	35.233794000
1	-11.963953000	-39.516980000	33.679491000
1	-11.644370000	-38.273764000	34.902186000
6	-9.970302000	-37.802586000	28.915138000
6	-11.187036000	-38.238049000	29.739445000
8	-11.075881000	-38.351754000	30.973168000
6	-10.202857000	-37.163223000	27.536255000
6	-10.876352000	-35.774147000	27.537956000
6	-9.979095000	-34.701209000	28.174686000
6	-11.275091000	-35.373341000	26.109963000
1	-9.386745000	-37.142585000	29.564253000
1	-9.226995000	-37.073197000	27.036374000
1	-10.784191000	-37.848396000	26.903482000
1	-11.798027000	-35.839619000	28.134326000
1	-10.480702000	-33.726754000	28.174475000
1	-9.717909000	-34.936516000	29.212980000
1	-9.042342000	-34.593009000	27.610286000
1	-11.719045000	-34.371872000	26.093204000
1	-10.395588000	-35.360631000	25.450112000
1	-12.013805000	-36.064431000	25.687470000
7	-12.305502000	-38.547243000	29.059931000
6	-13.507731000	-39.158289000	29.628331000
6	-14.063774000	-40.229014000	28.682038000
6	-14.242255000	-39.755018000	27.230147000
8	-14.313152000	-38.484955000	27.049359000
8	-14.292044000	-40.613977000	26.319735000
1	-12.398814000	-38.293508000	28.083673000
1	-14.262952000	-38.387996000	29.820472000
1	-13.408953000	-41.106745000	28.669607000
1	-15.038904000	-40.562994000	29.063202000
8	-17.145094000	-41.082962000	30.223366000
1	-12.707364000	-42.399540000	33.064198000
1	-12.359222000	-43.609756000	30.658502000
1	-19.481278000	-40.279659000	35.242605000
1	-16.819994000	-40.496946000	35.709503000
1	-22.663399000	-41.622624000	31.288390000
1	-22.329199000	-42.862446000	28.885540000
1	-18.214168000	-44.783663000	26.245663000
1	-15.529982000	-44.905803000	26.694587000
6	-20.270809000	-45.799518000	32.074576000
1	-19.861575000	-46.798754000	31.887601000
1	-21.263892000	-45.916906000	32.528643000
1	-20.387955000	-45.294736000	31.112051000
1	-17.084863000	-36.802533000	37.593072000
1	-23.764729000	-36.428305000	35.417925000
1	-27.288339000	-36.139212000	32.271964000
1	-27.093347000	-40.712876000	34.038196000
1	-28.763340000	-39.642666000	30.123567000
1	-11.747699000	-42.324666000	19.907222000
1	-13.157847000	-41.741701000	24.110947000
1	-17.632882000	-40.272791000	21.340379000
1	-13.214143000	-39.398991000	22.032670000
1	-13.232947000	-39.603541000	30.586817000
1	-9.361722000	-38.709968000	28.791011000
1	-11.950714000	-35.686989000	31.037664000
1	-13.985486000	-37.392276000	34.640952000
1	-18.411096000	-32.530321000	38.140308000
1	-13.121250000	-29.811016000	28.268737000
1	-17.438841000	-23.704787000	29.073047000
1	-12.602827000	-25.613912000	29.779595000
8	-23.128950000	-39.711826000	32.879956000
1	-23.851249000	-39.052395000	32.942339000
1	-22.686342000	-39.452760000	32.047944000

^{4}IM1$_{HA,III}$

6	-19.590710000	-43.535098000	28.457267000
6	-20.227757000	-41.403016000	32.753346000
6	-15.479013000	-41.817184000	33.626465000
6	-14.838824000	-43.886874000	29.306752000
6	-20.178456000	-42.891284000	29.539688000
6	-21.585087000	-42.579905000	29.645097000
6	-21.765616000	-41.975859000	30.852542000
6	-20.465398000	-41.923058000	31.479352000
6	-18.991976000	-41.378839000	33.385907000
6	-18.752809000	-40.803596000	34.686450000
6	-17.418099000	-40.918600000	34.929362000
6	-16.833659000	-41.565714000	33.780316000
6	-14.883524000	-42.424444000	32.523549000
6	-13.464644000	-42.670718000	32.388494000
6	-13.286001000	-43.257899000	31.173600000
6	-14.595571000	-43.369546000	30.571908000
6	-16.092451000	-43.984738000	28.705967000
6	-16.306728000	-44.500308000	27.375160000
6	-17.647658000	-44.427441000	27.145192000
6	-18.238901000	-43.845185000	28.328078000
7	-19.512267000	-42.477156000	30.668044000
7	-17.813148000	-41.859520000	32.853851000
7	-15.551032000	-42.860390000	31.414077000
7	-17.277272000	-43.601243000	29.269206000
26	-17.522397000	-42.634036000	31.023874000
1	-21.074310000	-40.961953000	33.273092000
1	-14.825386000	-41.514629000	34.439506000
1	-13.986804000	-44.183052000	28.703256000
1	-20.240069000	-43.801186000	27.627736000
6	-16.445725000	-35.833849000	28.934648000
6	-18.533331000	-36.635219000	29.126018000
6	-19.893234000	-36.904937000	29.360435000
6	-18.254432000	-38.833478000	28.041287000
6	-17.729612000	-37.602917000	28.459078000
6	-17.692079000	-35.508313000	29.427795000
6	-20.398481000	-38.130877000	28.950378000
6	-19.597215000	-39.090607000	28.296566000
7	-16.460376000	-37.084775000	28.351536000
1	-15.659841000	-37.553949000	27.910440000
6	-18.144231000	-34.264079000	30.141864000
6	-17.032429000	-33.470354000	30.850180000
7	-17.587416000	-32.343398000	31.592599000
6	-17.762642000	-31.131228000	31.049889000
8	-17.376407000	-30.830724000	29.894755000
6	-18.454326000	-30.106276000	31.929851000
1	-18.060102000	-32.540259000	32.487458000
8	-21.738452000	-38.476291000	29.124492000
6	-22.387646000	-38.087142000	30.250932000
1	-15.530695000	-35.257441000	28.945646000
1	-20.532732000	-36.166642000	29.832899000
1	-17.630597000	-39.576502000	27.554864000
1	-20.049560000	-40.034191000	28.008974000
1	-18.893240000	-34.538298000	30.896410000
1	-18.653996000	-33.579633000	29.447668000
1	-16.485668000	-34.108560000	31.552416000
1	-16.319070000	-33.072926000	30.123222000
1	-17.784541000	-29.250937000	32.070167000
1	-18.755987000	-30.526271000	32.890987000
1	-19.346535000	-29.739458000	31.410207000
1	-23.465106000	-38.060669000	30.147394000
1	-21.872239000	-37.440648000	30.951501000
1	-17.979412000	-40.558006000	30.143430000
6	-19.412609000	-45.031891000	32.951295000
16	-17.708571000	-44.755369000	32.340464000
1	-19.862445000	-44.052752000	33.148821000
1	-19.338061000	-45.557176000	33.910021000
6	-17.192824000	-40.142545000	22.299609000
6	-15.679443000	-40.044819000	22.144801000
8	-15.031096000	-40.983784000	21.655409000
6	-17.642867000	-41.289421000	23.231855000
6	-19.147422000	-41.562931000	23.030613000
6	-17.291723000	-40.980013000	24.694294000
6	-19.680433000	-42.784538000	23.788592000
1	-17.613404000	-39.190758000	22.649833000
1	-17.091737000	-42.188390000	22.921520000
1	-19.717631000	-40.671836000	23.332873000
1	-19.341422000	-41.701712000	21.956914000
1	-17.510527000	-41.828515000	25.350887000
1	-16.230813000	-40.747179000	24.831995000
1	-17.873180000	-40.120591000	25.056567000
1	-20.737277000	-42.960450000	23.554394000
1	-19.123660000	-43.690634000	23.514925000
1	-19.597889000	-42.657880000	24.873088000
7	-15.122259000	-38.871035000	22.515944000
6	-13.693347000	-38.590269000	22.512407000
6	-13.190261000	-38.387991000	23.942507000
8	-13.997183000	-37.413116000	24.582678000
1	-15.658835000	-38.235411000	23.094416000
1	-13.519172000	-37.679214000	21.924401000
1	-13.244626000	-39.337292000	24.487379000
1	-12.135863000	-38.066057000	23.903484000
1	-14.217701000	-37.786861000	25.479821000
6	-12.440802000	-42.581424000	24.251428000
6	-11.967260000	-43.158249000	22.922199000
8	-11.090576000	-44.022143000	22.870734000
6	-13.070318000	-43.658769000	25.150991000
8	-13.287047000	-43.212440000	26.475248000
1	-11.561633000	-42.183610000	24.772084000
1	-14.006081000	-44.013097000	24.682674000
1	-12.388844000	-44.514118000	25.205523000
1	-13.742921000	-42.335052000	26.447811000
7	-12.574966000	-42.647638000	21.810381000
6	-12.254980000	-43.116046000	20.475500000
6	-13.508327000	-43.458530000	19.641160000
6	-14.303724000	-44.632314000	20.170218000
6	-13.977086000	-45.945660000	19.805327000
6	-15.372752000	-44.430763000	21.054983000
6	-14.694533000	-47.031509000	20.309242000
6	-16.092092000	-45.515180000	21.561347000
6	-15.756587000	-46.819113000	21.190915000
1	-13.338886000	-41.981019000	21.907696000
1	-11.610097000	-43.990461000	20.596928000
1	-14.148697000	-42.569803000	19.594001000
1	-13.173291000	-43.668609000	18.615960000
1	-13.149662000	-46.118597000	19.119168000
1	-15.631182000	-43.414735000	21.342428000
1	-14.424618000	-48.042829000	20.013434000
1	-16.916419000	-45.339638000	22.248711000
1	-16.317048000	-47.662722000	21.586526000

6	-26.546379000	-41.114774000	33.203525000
6	-27.338145000	-40.832592000	31.930258000
8	-28.186104000	-41.628752000	31.490518000
6	-26.366974000	-42.624197000	33.451975000
8	-27.623413000	-43.272134000	33.591380000
6	-25.556216000	-42.890933000	34.714567000
1	-25.569284000	-40.622147000	33.157680000
1	-25.818521000	-43.043716000	32.589211000
1	-28.132590000	-42.964971000	32.811746000
1	-25.453568000	-43.970206000	34.871137000
1	-24.557491000	-42.447294000	34.639184000
1	-26.064805000	-42.467922000	35.589221000
7	-27.068124000	-39.658888000	31.313367000
6	-27.742412000	-39.271933000	30.082574000
6	-27.107196000	-39.859898000	28.802732000
6	-25.638220000	-39.423245000	28.658825000
6	-27.962348000	-39.476592000	27.586453000
6	-24.854855000	-40.114506000	27.536654000
1	-26.376968000	-39.022800000	31.716243000
1	-27.734048000	-38.175362000	30.029636000
1	-27.133584000	-40.952853000	28.915112000
1	-25.601101000	-38.332420000	28.509358000
1	-25.127492000	-39.621275000	29.608702000
1	-27.586950000	-39.936387000	26.666087000
1	-29.002763000	-39.799499000	27.716724000
1	-27.966068000	-38.388101000	27.435030000
1	-23.803134000	-39.809655000	27.563777000
1	-24.891235000	-41.205966000	27.649080000
1	-25.249634000	-39.869513000	26.543590000
6	-26.315414000	-35.757388000	31.914889000
6	-25.258499000	-36.526840000	32.708995000
8	-25.157270000	-37.763144000	32.531758000
6	-26.294642000	-34.218677000	31.913873000
6	-25.081761000	-33.520345000	31.255396000
6	-24.850115000	-33.985666000	29.809921000
6	-25.276216000	-31.997247000	31.312235000
1	-26.257083000	-36.142901000	30.891124000
1	-27.202942000	-33.888608000	31.387873000
1	-26.406021000	-33.848468000	32.943173000
1	-24.178749000	-33.748301000	31.835510000
1	-24.030057000	-33.421579000	29.351139000
1	-24.582108000	-35.046652000	29.752683000
1	-25.747389000	-33.829201000	29.194387000
1	-24.406173000	-31.476171000	30.897089000
1	-26.160540000	-31.688395000	30.736921000
1	-25.406680000	-31.652067000	32.344643000
7	-24.521515000	-35.839572000	33.580803000
6	-23.409388000	-36.324633000	34.397816000
6	-22.273449000	-35.282258000	34.315331000
8	-22.903545000	-34.010996000	34.371638000
6	-21.432245000	-35.421674000	33.042745000
1	-24.593455000	-34.827783000	33.629493000
1	-23.092490000	-37.308077000	34.045182000
1	-21.626389000	-35.421608000	35.193232000
1	-22.191491000	-33.345672000	34.622662000
1	-20.701231000	-34.607148000	33.004362000
1	-20.895057000	-36.379139000	33.022472000
1	-22.071357000	-35.358631000	32.151624000
6	-17.048251000	-24.661033000	29.401721000
6	-15.654755000	-24.728488000	28.769403000
8	-15.425344000	-24.238854000	27.667503000
6	-17.940816000	-25.830882000	28.953503000
6	-17.494173000	-27.180103000	29.530463000
8	-16.683341000	-27.242826000	30.470288000
7	-18.024927000	-28.277443000	28.965758000
1	-16.987503000	-24.654805000	30.494313000
1	-18.973848000	-25.648472000	29.280438000
1	-17.963051000	-25.885394000	27.857989000
1	-18.673994000	-28.196412000	28.196352000
1	-17.780954000	-29.225013000	29.324165000
7	-14.715350000	-25.376731000	29.526263000
6	-13.420458000	-25.745769000	28.984739000
6	-13.255006000	-27.183971000	28.449047000
8	-12.273516000	-27.447356000	27.757559000
1	-15.079534000	-25.888661000	30.323207000
1	-13.213307000	-25.077015000	28.147870000
7	-14.194854000	-28.093354000	28.810045000
6	-14.179608000	-29.461981000	28.301687000
6	-14.850780000	-29.575975000	26.914935000
6	-14.835003000	-30.986201000	26.364123000
6	-15.710055000	-31.963636000	26.864878000
6	-13.934303000	-31.351737000	25.354724000
6	-15.681950000	-33.267953000	26.365284000
6	-13.904118000	-32.656640000	24.856189000
6	-14.777062000	-33.623812000	25.360172000
1	-14.985778000	-27.816056000	29.381754000
1	-14.705223000	-30.093781000	29.023805000
1	-15.883923000	-29.216941000	27.007495000
1	-14.328790000	-28.897965000	26.230419000
1	-16.408443000	-31.703108000	27.658544000
1	-13.250185000	-30.604232000	24.957293000
1	-16.365326000	-34.010723000	26.767965000
1	-13.196657000	-32.919350000	24.072999000
1	-14.740272000	-34.643869000	24.984587000
6	-19.066177000	-32.914965000	37.380344000
6	-19.044870000	-34.446181000	37.356976000
8	-20.013932000	-35.129225000	37.687333000
6	-18.713682000	-32.244712000	36.038309000
6	-19.757591000	-32.548924000	34.935415000
8	-20.960202000	-32.509471000	35.311650000
8	-19.333097000	-32.797110000	33.770924000
1	-20.079792000	-32.625139000	37.660519000
1	-18.696602000	-31.156162000	36.193119000
1	-17.716474000	-32.526521000	35.682110000
7	-17.855783000	-34.998340000	36.979109000
6	-17.658247000	-36.419782000	36.766135000
6	-16.895405000	-36.659286000	35.456725000
8	-15.695446000	-35.894220000	35.568443000
6	-17.705426000	-36.297514000	34.210743000
1	-17.101087000	-34.413318000	36.644941000
1	-18.640641000	-36.898518000	36.754062000
1	-16.650009000	-37.735709000	35.411815000
1	-15.319744000	-35.751875000	34.670975000
1	-17.096278000	-36.436416000	33.310992000
1	-18.583505000	-36.948818000	34.126611000
1	-18.048360000	-35.257735000	34.237192000
6	-12.937178000	-35.289721000	31.297045000
6	-13.562201000	-36.002531000	32.484814000
8	-14.503094000	-35.476724000	33.099898000
1	-12.851457000	-34.225672000	31.532015000
1	-13.604729000	-35.384856000	30.433397000
7	-13.028113000	-37.203038000	32.791080000
6	-13.538773000	-38.065671000	33.850930000
6	-12.415120000	-38.927692000	34.427117000
1	-12.329596000	-37.603987000	32.158743000
1	-14.340865000	-38.708809000	33.459358000
1	-12.808678000	-39.595582000	35.202464000
1	-11.950335000	-39.542074000	33.647236000
1	-11.634175000	-38.304008000	34.876372000
6	-9.980704000	-37.794014000	28.875979000
6	-11.193341000	-38.230568000	29.705636000
8	-11.075623000	-38.346848000	30.938440000
6	-10.219996000	-37.151919000	27.499578000
6	-10.895501000	-35.763857000	27.506812000
6	-9.998920000	-34.691406000	28.145281000
6	-11.297054000	-35.359615000	26.080566000
1	-9.393692000	-37.135467000	29.523445000
1	-9.246307000	-37.059454000	26.995902000
1	-10.803003000	-37.836557000	26.867736000
1	-11.816045000	-35.832544000	28.104577000
1	-10.501676000	-33.717544000	28.147749000
1	-9.736672000	-34.928836000	29.182815000
1	-9.062744000	-34.580784000	27.580380000
1	-11.744776000	-34.359773000	26.067538000
1	-10.418094000	-35.341314000	25.420114000
1	-12.033266000	-36.052310000	25.656277000
7	-12.315716000	-38.537511000	29.031422000
6	-13.514854000	-39.150093000	29.604633000
6	-14.070041000	-40.225587000	28.663295000
6	-14.253211000	-39.757162000	27.210227000
8	-14.328461000	-38.488290000	27.024493000
8	-14.302314000	-40.620108000	26.303292000
1	-12.413030000	-38.284108000	28.055459000
1	-14.271919000	-38.381460000	29.795880000
1	-13.412555000	-41.101341000	28.651999000
1	-15.043175000	-40.561084000	29.048088000
8	-17.152782000	-41.063151000	30.244922000
1	-12.720037000	-42.414689000	33.131820000
1	-12.365284000	-43.584289000	30.707100000
1	-19.517421000	-40.356720000	35.308879000
1	-16.861985000	-40.586927000	35.797098000
1	-22.671276000	-41.589498000	31.301487000
1	-22.324527000	-42.799189000	28.884960000
1	-18.197369000	-44.710957000	26.256361000
1	-15.514180000	-44.833952000	26.720161000
6	-20.285487000	-45.825396000	31.973229000
1	-19.860480000	-46.816952000	31.780563000
1	-21.292245000	-45.960994000	32.390570000
1	-20.374756000	-45.302326000	31.017540000
1	-17.079171000	-36.851737000	37.596254000
1	-23.753472000	-36.419940000	35.435168000
1	-27.285133000	-36.102214000	32.300694000
1	-27.097127000	-40.686266000	34.053179000
1	-28.784925000	-39.599981000	30.150287000
1	-11.678235000	-42.351144000	19.932060000
1	-13.149415000	-41.758427000	24.115150000
1	-17.587984000	-40.319159000	21.290220000
1	-13.180388000	-39.421164000	22.023284000
1	-13.236552000	-39.591339000	30.563918000
1	-9.373031000	-38.701324000	28.746992000
1	-11.957566000	-35.693556000	31.030275000
1	-13.980925000	-37.433304000	34.624427000
1	-18.372797000	-32.572611000	38.163266000
1	-13.138783000	-29.789997000	28.236430000
1	-17.499502000	-23.721532000	29.070390000
1	-12.640513000	-25.587045000	29.739596000

8	-23.131399000	-39.686755000	32.883034000
1	-23.854977000	-39.028924000	32.947754000
1	-22.690238000	-39.424931000	32.051061000

^{2}IM2$_{Hy,III}$

6	-21.208411000	-44.841171000	29.763317000
6	-21.125938000	-42.373694000	33.939025000
6	-16.358644000	-41.707745000	33.381946000
6	-16.620372000	-43.545214000	28.913668000
6	-21.589739000	-44.277402000	30.976799000
6	-22.927265000	-44.319316000	31.513551000
6	-22.914906000	-43.593821000	32.669628000
6	-21.563725000	-43.121352000	32.848720000
6	-19.807002000	-41.994942000	34.160084000
6	-19.344955000	-41.286827000	35.329235000
6	-17.997779000	-41.139782000	35.191493000
6	-17.637646000	-41.737185000	33.927382000
6	-16.020081000	-42.147142000	32.108748000
6	-14.708744000	-42.001403000	31.514879000
6	-14.792034000	-42.481874000	30.244564000
6	-16.153128000	-42.938687000	30.071140000
6	-17.895643000	-44.077304000	28.765742000
6	-18.345986000	-44.800896000	27.599942000
6	-19.622680000	-45.199695000	27.855479000
6	-19.959474000	-44.704799000	29.170567000
7	-20.770858000	-43.543747000	31.805517000
7	-18.751586000	-42.258899000	33.315977000
7	-16.884784000	-42.722424000	31.212014000
7	-18.892633000	-44.027393000	29.707931000
26	-18.790797000	-43.244522000	31.564487000
1	-21.868218000	-42.072090000	34.671404000
1	-15.573251000	-41.254279000	33.979287000
1	-15.921603000	-43.622903000	28.086160000
1	-21.965085000	-45.391820000	29.212066000
6	-16.005625000	-35.196418000	27.763414000
6	-17.523152000	-36.489783000	28.808185000
6	-18.550805000	-37.079213000	29.566659000
6	-16.815050000	-38.715648000	28.007271000
6	-16.660782000	-37.324094000	28.042713000
6	-17.091322000	-35.128388000	28.612738000
6	-18.688671000	-38.458610000	29.534265000
6	-17.841609000	-39.278255000	28.757283000
7	-15.745258000	-36.507171000	27.413267000
1	-14.963382000	-36.847279000	26.853002000
6	-17.717292000	-33.917299000	29.251158000
6	-16.918354000	-33.354610000	30.452408000
7	-17.712704000	-32.450793000	31.276408000
6	-17.860107000	-31.148019000	31.017288000
8	-17.303793000	-30.564856000	30.053130000
6	-18.741320000	-30.379063000	31.984655000
1	-18.255140000	-32.856958000	32.057495000
8	-19.727931000	-39.006185000	30.294370000
6	-19.389308000	-40.046553000	31.165076000
1	-15.397709000	-34.396181000	27.361481000
1	-19.221859000	-36.491512000	30.185865000
1	-16.166543000	-39.346148000	27.407110000
1	-17.997093000	-40.350670000	28.752041000
1	-18.718643000	-34.184184000	29.609450000
1	-17.857795000	-33.108081000	28.523503000
1	-16.569554000	-34.166868000	31.095172000
1	-16.037071000	-32.815832000	30.092855000
1	-18.126517000	-29.635438000	32.504237000
1	-19.229124000	-31.037825000	32.705327000
1	-19.504889000	-29.835855000	31.417373000
1	-20.131015000	-40.031871000	31.967799000
1	-18.377719000	-39.933272000	31.558725000
1	-20.307472000	-41.515560000	30.229385000
6	-19.093992000	-45.588213000	33.924875000
16	-18.132079000	-45.205791000	32.401568000
1	-19.521101000	-44.662942000	34.316235000
1	-18.363630000	-45.944593000	34.662082000
6	-16.922259000	-40.651638000	21.955193000
6	-15.411635000	-40.829694000	21.868746000
8	-14.910204000	-41.962348000	21.804225000
6	-17.522405000	-41.031173000	23.330518000
6	-19.053254000	-41.173742000	23.199137000
6	-17.114523000	-40.017569000	24.408270000
6	-19.765159000	-41.662216000	24.466622000
1	-17.206345000	-39.620388000	21.705643000
1	-17.115169000	-42.015530000	23.604045000
1	-19.477637000	-40.204410000	22.895329000
1	-19.269351000	-41.872411000	22.377560000
1	-17.534934000	-40.277940000	25.383916000
1	-16.029200000	-39.964488000	24.540991000
1	-17.482664000	-39.013315000	24.153777000
1	-20.831017000	-41.831591000	24.272083000
1	-19.339213000	-42.607298000	24.826835000
1	-19.691696000	-40.937391000	25.283504000
7	-14.682513000	-39.690101000	21.814844000
6	-13.227106000	-39.638359000	21.809379000
6	-12.698741000	-39.089777000	23.135540000
8	-13.351408000	-37.862326000	23.413460000
1	-15.120735000	-38.824386000	22.105444000
1	-12.909868000	-38.986406000	20.984582000
1	-12.896275000	-39.814111000	23.933046000
1	-11.606393000	-38.955247000	23.050452000
1	-13.541191000	-37.867410000	24.387713000
6	-12.593903000	-43.101739000	24.891940000
6	-12.297304000	-44.167423000	23.843530000
8	-11.605975000	-45.152597000	24.103698000
6	-13.556521000	-43.631430000	25.970761000
8	-13.713966000	-42.732047000	27.052446000
1	-11.648984000	-42.835861000	25.378265000
1	-14.525179000	-43.870625000	25.499498000
1	-13.153945000	-44.562687000	26.381898000
1	-13.933987000	-41.836870000	26.693331000
7	-12.866711000	-43.958025000	22.619503000
6	-12.767500000	-44.931901000	21.549361000
6	-14.137034000	-45.277923000	20.925390000
6	-15.101661000	-45.951890000	21.877870000
6	-15.049480000	-47.335613000	22.096054000
6	-16.058325000	-45.201584000	22.576210000
6	-15.925534000	-47.956336000	22.987499000
6	-16.938696000	-45.820169000	23.467053000
6	-16.875688000	-47.199387000	23.676756000
1	-13.470699000	-43.152480000	22.469152000
1	-12.292731000	-45.818745000	21.977582000
1	-14.586155000	-44.355326000	20.539715000
1	-13.949063000	-45.930628000	20.061445000
1	-14.312059000	-47.932742000	21.562221000
1	-16.100799000	-44.127282000	22.416262000
1	-15.866919000	-49.030980000	23.144011000
1	-17.678336000	-45.222396000	23.994960000
1	-17.559276000	-47.679942000	24.372124000
6	-27.305573000	-41.749914000	33.057855000
6	-27.356839000	-41.697111000	31.534081000
8	-27.945706000	-42.566629000	30.867891000
6	-27.374372000	-43.192768000	33.590359000
8	-28.580809000	-43.830430000	33.196897000
6	-27.291530000	-43.229890000	35.111709000
1	-26.396684000	-41.261611000	33.424724000
1	-26.506606000	-43.742565000	33.180839000
1	-28.630214000	-43.657900000	32.232441000
1	-27.347606000	-44.265346000	35.465106000
1	-26.353299000	-42.784073000	35.459750000
1	-28.130001000	-42.675536000	35.550615000
7	-26.734735000	-40.639375000	30.962245000
6	-26.690049000	-40.462395000	29.518182000
6	-25.380240000	-40.953080000	28.862969000
6	-24.173461000	-40.100727000	29.303286000
6	-25.551068000	-40.977249000	27.336524000
6	-22.804562000	-40.670649000	28.910608000
1	-26.262920000	-39.954422000	31.557328000
1	-26.837026000	-39.396478000	29.292513000
1	-25.221677000	-41.984053000	29.211507000
1	-24.280988000	-39.090193000	28.878397000
1	-24.201529000	-39.973447000	30.392484000
1	-24.658060000	-41.363021000	26.834193000
1	-26.396875000	-41.611532000	27.044089000
1	-25.739089000	-39.967287000	26.946565000
1	-21.997932000	-40.020758000	29.268916000
1	-22.664846000	-41.672912000	29.339141000
1	-22.688206000	-40.759775000	27.824480000
6	-26.332558000	-36.738789000	31.676662000
6	-25.318650000	-37.429408000	32.590331000
8	-25.287432000	-38.682830000	32.613040000
6	-26.239748000	-35.218182000	31.461982000
6	-25.012279000	-34.686180000	30.686404000
6	-24.919672000	-35.271598000	29.269399000
6	-25.059900000	-33.151295000	30.638021000
1	-26.284633000	-37.267449000	30.718334000
1	-27.144975000	-34.911715000	30.916770000
1	-26.300743000	-34.707017000	32.433473000
1	-24.099446000	-34.959841000	31.230177000
1	-24.063506000	-34.847352000	28.732710000
1	-24.788830000	-36.359589000	29.276302000
1	-25.824554000	-35.044454000	28.687857000
1	-24.186354000	-32.749379000	30.112429000
1	-25.960110000	-32.798677000	30.114650000
1	-25.063472000	-32.724312000	31.647531000
7	-24.544883000	-36.657512000	33.349534000
6	-23.448231000	-37.068730000	34.225719000
6	-22.273164000	-36.093923000	33.988541000
8	-22.861735000	-34.807205000	33.847659000
6	-21.440649000	-36.454776000	32.754400000
1	-24.561544000	-35.646834000	33.245826000
1	-23.182012000	-38.107653000	34.021210000
1	-21.627129000	-36.119507000	34.876439000
1	-22.166805000	-34.133538000	34.124928000
1	-20.690135000	-35.674132000	32.592245000
1	-20.927048000	-37.416324000	32.883058000
1	-22.071718000	-36.521855000	31.858859000
6	-16.339048000	-24.714534000	31.767747000
6	-14.874696000	-24.788266000	31.324156000
8	-14.414205000	-24.007303000	30.496768000
6	-17.262585000	-25.513793000	30.832355000

6	-17.062937000	-27.030189000	30.947880000
8	-16.433958000	-27.526600000	31.897377000
7	-17.601921000	-27.782089000	29.972627000
1	-16.462604000	-25.080497000	32.791581000
1	-18.310620000	-25.290601000	31.075662000
1	-17.106571000	-25.197165000	29.793557000
1	-18.100994000	-27.350350000	29.208276000
1	-17.512909000	-28.820178000	30.000608000
7	-14.149404000	-25.788746000	31.914370000
6	-12.832283000	-26.161449000	31.430918000
6	-12.754424000	-27.316496000	30.409599000
8	-11.733574000	-27.451074000	29.738689000
1	-14.695205000	-26.470781000	32.430715000
1	-12.400677000	-25.286983000	30.942205000
7	-13.819416000	-28.155248000	30.343388000
6	-13.893304000	-29.233622000	29.362902000
6	-14.367616000	-28.723844000	27.983906000
6	-14.509154000	-29.820162000	26.949769000
6	-15.554292000	-30.754978000	27.035243000
6	-13.604179000	-29.924668000	25.884949000
6	-15.692231000	-31.755405000	26.070983000
6	-13.738906000	-30.927256000	24.920755000
6	-14.786871000	-31.845608000	25.009336000
1	-14.644016000	-27.986992000	30.910275000
1	-14.591059000	-29.984031000	29.745403000
1	-15.332353000	-28.220102000	28.126676000
1	-13.653231000	-27.968882000	27.637811000
1	-16.256599000	-30.704160000	27.865135000
1	-12.788573000	-29.208398000	25.809248000
1	-16.509707000	-32.467851000	26.148541000
1	-13.028700000	-30.987008000	24.099369000
1	-14.899248000	-32.623693000	24.258513000
6	-18.931674000	-33.868701000	36.786535000
6	-18.748937000	-35.375835000	36.590609000
8	-19.658333000	-36.187697000	36.759604000
6	-18.713158000	-33.034726000	35.507392000
6	-19.810089000	-33.295066000	34.445856000
8	-20.989962000	-33.305280000	34.891153000
8	-19.453346000	-33.463995000	33.244979000
1	-19.955918000	-33.715288000	37.129345000
1	-18.759054000	-31.971049000	35.781054000
1	-17.724999000	-33.209607000	35.067986000
7	-17.483147000	-35.753210000	36.244913000
6	-17.119988000	-37.097895000	35.838664000
6	-16.337923000	-37.058831000	34.517389000
8	-15.247408000	-36.164955000	34.743593000
6	-17.198088000	-36.646972000	33.321817000
1	-16.787640000	-35.051916000	36.023499000
1	-18.037364000	-37.684672000	35.746470000
1	-15.951710000	-38.078065000	34.334778000
1	-14.934920000	-35.802004000	33.885018000
1	-16.586223000	-36.589252000	32.415279000
1	-17.988311000	-37.386709000	33.147890000
1	-17.675274000	-35.673407000	33.477394000
6	-13.009847000	-34.357554000	30.475963000
6	-13.247764000	-35.271904000	31.665653000
8	-14.229307000	-35.091304000	32.401802000
1	-13.114499000	-33.320002000	30.806187000
1	-13.789221000	-34.542069000	29.727974000
7	-12.334866000	-36.253908000	31.836562000
6	-12.403860000	-37.259691000	32.888702000
6	-11.037169000	-37.469226000	33.544040000
1	-11.610811000	-36.372466000	31.123226000
1	-12.764337000	-38.210523000	32.467669000
1	-11.095350000	-38.258524000	34.302694000
1	-10.284865000	-37.763362000	32.802506000
1	-10.694213000	-36.548549000	34.028590000
6	-9.541203000	-36.163785000	27.501609000
6	-10.538124000	-36.763177000	28.498642000
8	-10.341238000	-36.623274000	29.718015000
6	-10.013625000	-35.893117000	26.062631000
6	-11.106042000	-34.815049000	25.892140000
6	-10.624242000	-33.426337000	26.340285000
6	-11.590772000	-34.780899000	24.434183000
1	-9.158143000	-35.252316000	27.970270000
1	-9.135346000	-35.588779000	25.474050000
1	-10.357926000	-36.830191000	25.603398000
1	-11.964343000	-35.093107000	26.520280000
1	-11.411865000	-32.677643000	26.199831000
1	-10.338473000	-33.405707000	27.398038000
1	-9.751873000	-33.108910000	25.751628000
1	-12.369130000	-34.019371000	24.304049000
1	-10.763304000	-34.527829000	23.755750000
1	-12.007256000	-35.744615000	24.118164000
7	-11.550670000	-37.484471000	27.983279000
6	-12.574323000	-38.164791000	28.770428000
6	-13.072347000	-39.417649000	28.045782000
6	-13.602794000	-39.194374000	26.614925000
8	-13.510092000	-38.015030000	26.124437000
8	-14.076843000	-40.189314000	26.015243000
1	-11.794256000	-37.419104000	26.999431000
1	-13.413906000	-37.485834000	28.964673000
1	-12.275386000	-40.169604000	27.978533000
1	-13.872519000	-39.879627000	28.635795000
8	-19.397208000	-41.321047000	30.512956000
1	-13.853086000	-41.568292000	32.018365000
1	-14.037829000	-42.543448000	29.469591000
1	-19.982220000	-40.946747000	36.135527000
1	-17.302653000	-40.651403000	35.862809000
1	-23.733565000	-43.387136000	33.347449000
1	-23.763245000	-44.829068000	31.050666000
1	-20.293913000	-45.764543000	27.220220000
1	-17.741723000	-44.980152000	26.719218000
6	-20.183574000	-46.641065000	33.712184000
1	-19.761330000	-47.569598000	33.311021000
1	-20.680742000	-46.875791000	34.664201000
1	-20.942272000	-46.284766000	33.009403000
1	-16.488399000	-37.566427000	36.608500000
1	-23.786250000	-36.998662000	35.267006000
1	-27.319311000	-36.985361000	32.092701000
1	-28.170365000	-41.194755000	33.449420000
1	-27.537558000	-41.015020000	29.103833000
1	-12.109156000	-44.555960000	20.750559000
1	-13.016062000	-42.194988000	24.445726000
1	-17.345118000	-41.304149000	21.181378000
1	-12.850309000	-40.645668000	21.618485000
1	-12.136074000	-38.430873000	29.735262000
1	-8.691635000	-36.861408000	27.474166000
1	-12.028668000	-34.501975000	30.016854000
1	-13.142499000	-36.933426000	33.623284000
1	-18.239807000	-33.533749000	37.573448000
1	-12.901619000	-29.684843000	29.270981000
1	-16.626220000	-23.659745000	31.735470000
1	-12.187430000	-26.425570000	32.278012000
8	-24.058860000	-40.747208000	34.036594000
1	-24.477015000	-39.929064000	33.691023000
1	-23.564594000	-41.079754000	33.271831000

^{4}IM2$_{Hy,III}$

6	-21.200842000	-44.932051000	29.765016000
6	-21.104163000	-42.421386000	33.915064000
6	-16.344699000	-41.707850000	33.347128000
6	-16.600518000	-43.666098000	28.927033000
6	-21.575717000	-44.356317000	30.973060000
6	-22.914117000	-44.371402000	31.502840000
6	-22.894941000	-43.635682000	32.652858000
6	-21.538635000	-43.184895000	32.835868000
6	-19.789059000	-42.028277000	34.126280000
6	-19.340316000	-41.277771000	35.271732000
6	-17.995035000	-41.110995000	35.131554000
6	-17.623039000	-41.739041000	33.889063000
6	-16.006850000	-42.183042000	32.088155000
6	-14.706736000	-42.028357000	31.481416000
6	-14.789638000	-42.544706000	30.223780000
6	-16.138816000	-43.034809000	30.072284000
6	-17.874534000	-44.200508000	28.785350000
6	-18.326804000	-44.919763000	27.620784000
6	-19.609194000	-45.307380000	27.870727000
6	-19.947847000	-44.809225000	29.180592000
7	-20.746465000	-43.632937000	31.802433000
7	-18.727393000	-42.302178000	33.292484000
7	-16.866671000	-42.807028000	31.215478000
7	-18.874643000	-44.144546000	29.726047000
26	-18.749282000	-43.397013000	31.599155000
1	-21.848903000	-42.104805000	34.638236000
1	-15.565899000	-41.222742000	33.927256000
1	-15.904997000	-43.742846000	28.097104000
1	-21.961520000	-45.475383000	29.212740000
6	-15.972026000	-35.114286000	27.755193000
6	-17.546322000	-36.377465000	28.753373000
6	-18.606291000	-36.946357000	29.482856000
6	-16.882593000	-38.612270000	27.941771000
6	-16.690770000	-37.226215000	27.996987000
6	-17.073384000	-35.026304000	28.582470000
6	-18.784258000	-38.321175000	29.431443000
6	-17.940795000	-39.153519000	28.662280000
7	-15.740234000	-36.428082000	27.395572000
1	-14.956697000	-36.786174000	26.849612000
6	-17.681177000	-33.807118000	29.222883000
6	-16.899022000	-33.286929000	30.454149000
7	-17.693317000	-32.387039000	31.282789000
6	-17.828354000	-31.080251000	31.038187000
8	-17.259969000	-30.490434000	30.085240000
6	-18.711565000	-30.315733000	32.007370000
1	-18.244863000	-32.798506000	32.054562000
8	-19.852797000	-38.849177000	30.158897000
6	-19.553182000	-39.914926000	31.027296000
1	-15.335641000	-34.326295000	27.373692000
1	-19.273705000	-36.346373000	30.094272000
1	-16.238461000	-39.254255000	27.348794000
1	-18.127424000	-40.220466000	28.642091000
1	-18.698914000	-34.050850000	29.550268000
1	-17.779201000	-32.982887000	28.505411000
1	-16.580517000	-34.119992000	31.085619000
1	-15.999046000	-32.758590000	30.126536000
1	-18.096742000	-29.578777000	32.536327000

1	-19.206028000	-30.978413000	32.719888000
1	-19.470039000	-29.764695000	31.440748000
1	-20.330143000	-39.894851000	31.798428000
1	-18.562422000	-39.793812000	31.471768000
1	-20.424788000	-41.394901000	30.089495000
6	-19.085330000	-45.799729000	34.094564000
16	-18.064273000	-45.524834000	32.584999000
1	-19.505571000	-44.843442000	34.419289000
1	-18.403323000	-46.136113000	34.885003000
6	-16.996171000	-40.667163000	22.009552000
6	-15.486100000	-40.840025000	21.901319000
8	-14.984132000	-41.970422000	21.804304000
6	-17.587422000	-41.161305000	23.351057000
6	-19.117654000	-41.306636000	23.217046000
6	-17.181936000	-40.234236000	24.504778000
6	-19.816220000	-41.899650000	24.446781000
1	-17.281889000	-39.619271000	21.846788000
1	-17.169531000	-42.160396000	23.541534000
1	-19.552265000	-40.320330000	22.993528000
1	-19.333598000	-41.940616000	22.344495000
1	-17.569960000	-40.593053000	25.462691000
1	-16.095627000	-40.155745000	24.616300000
1	-17.584962000	-39.223543000	24.349690000
1	-20.882213000	-42.062428000	24.247488000
1	-19.379251000	-42.866585000	24.727050000
1	-19.741391000	-41.240925000	25.317688000
7	-14.759812000	-39.698750000	21.862654000
6	-13.305176000	-39.641928000	21.825801000
6	-12.753119000	-39.084959000	23.138812000
8	-13.406779000	-37.860205000	23.425793000
1	-15.194071000	-38.837557000	22.172148000
1	-13.006904000	-38.992169000	20.992094000
1	-12.930045000	-39.807183000	23.943049000
1	-11.663696000	-38.944104000	23.030954000
1	-13.589258000	-37.870044000	24.401644000
6	-12.620051000	-43.076888000	24.877876000
6	-12.306932000	-44.128262000	23.819590000
8	-11.591811000	-45.099207000	24.068438000
6	-13.568171000	-43.633671000	25.955449000
8	-13.736437000	-42.748882000	27.047995000
1	-11.678366000	-42.797620000	25.362900000
1	-14.534954000	-43.884323000	25.486279000
1	-13.147849000	-44.562230000	26.354889000
1	-13.963339000	-41.850797000	26.700289000
7	-12.888219000	-43.922967000	22.600357000
6	-12.771103000	-44.886306000	21.522320000
6	-14.135091000	-45.264201000	20.904749000
6	-15.076189000	-45.966586000	21.860087000
6	-14.983872000	-47.348448000	22.076856000
6	-16.050638000	-45.244151000	22.563066000
6	-15.837897000	-47.994310000	22.971773000
6	-16.909190000	-45.888042000	23.457250000
6	-16.806115000	-47.265015000	23.665747000
1	-13.513101000	-43.131544000	22.459361000
1	-12.270183000	-45.763230000	21.941007000
1	-14.609920000	-44.351703000	20.526025000
1	-13.935682000	-45.907948000	20.036648000
1	-14.232068000	-47.924060000	21.539513000
1	-16.124237000	-44.171478000	22.403548000
1	-15.748148000	-49.066917000	23.127293000
1	-17.663507000	-45.311701000	23.988379000
1	-17.472901000	-47.765476000	24.363421000
6	-27.266586000	-41.768066000	33.093655000
6	-27.359136000	-41.722201000	31.571593000
8	-27.964486000	-42.595564000	30.925631000
6	-27.328753000	-43.207673000	33.635757000
8	-28.547673000	-43.841561000	33.276926000
6	-27.207445000	-43.237061000	35.154724000
1	-26.345689000	-41.282641000	33.433269000
1	-26.474253000	-43.764065000	33.207315000
1	-28.620850000	-43.674865000	32.312996000
1	-27.257864000	-44.270379000	35.515021000
1	-26.259377000	-42.792534000	35.476890000
1	-28.032751000	-42.677571000	35.611617000
7	-26.754663000	-40.665967000	30.978066000
6	-26.755212000	-40.493462000	29.532717000
6	-25.460928000	-40.973248000	28.839880000
6	-24.252042000	-40.101259000	29.233744000
6	-25.679527000	-41.013260000	27.319794000
6	-22.888154000	-40.662108000	28.812078000
1	-26.268005000	-39.977291000	31.556981000
1	-26.920365000	-39.430093000	29.307693000
1	-25.278223000	-41.999173000	29.191711000
1	-24.382520000	-39.099291000	28.795470000
1	-24.251139000	-39.956197000	30.321115000
1	-24.797171000	-41.390098000	26.792270000
1	-26.524462000	-41.662508000	27.059645000
1	-25.894840000	-40.009654000	26.927582000
1	-22.080286000	-39.991115000	29.125780000
1	-22.720468000	-41.650805000	29.262401000
1	-22.805910000	-40.779929000	27.725625000
6	-26.289192000	-36.769890000	31.610241000
6	-25.289499000	-37.451437000	32.546050000
8	-25.263025000	-38.704937000	32.585956000
6	-26.193910000	-35.250873000	31.385421000
6	-24.958234000	-34.725743000	30.618086000
6	-24.862093000	-35.309196000	29.200507000
6	-24.994461000	-33.190502000	30.572639000
1	-26.224045000	-37.305949000	30.657025000
1	-27.093410000	-34.947527000	30.829166000
1	-26.264235000	-34.733086000	32.352737000
1	-24.050353000	-35.006954000	31.166318000
1	-23.997708000	-34.893195000	28.670668000
1	-24.742944000	-36.398554000	29.205827000
1	-25.760518000	-35.070990000	28.613403000
1	-24.116955000	-32.794080000	30.049525000
1	-25.891031000	-32.830202000	30.048247000
1	-24.996491000	-32.765831000	31.583081000
7	-24.522337000	-36.671545000	33.303242000
6	-23.438839000	-37.071286000	34.200757000
6	-22.264007000	-36.092908000	33.974849000
8	-22.856056000	-34.811078000	33.806495000
6	-21.404990000	-36.466307000	32.763002000
1	-24.536257000	-35.662095000	33.186414000
1	-23.164153000	-38.110484000	34.008769000
1	-21.635362000	-36.103917000	34.875354000
1	-22.168988000	-34.130226000	34.086346000
1	-20.659072000	-35.681538000	32.599848000
1	-20.883622000	-37.419223000	32.921065000
1	-22.016265000	-36.558029000	31.856143000
6	-16.305250000	-24.645031000	31.827639000
6	-14.837689000	-24.717148000	31.394389000
8	-14.371181000	-23.932919000	30.573480000
6	-17.222044000	-25.441932000	30.883635000
6	-17.023614000	-26.958703000	30.996577000
8	-16.401235000	-27.457927000	31.948961000
7	-17.556227000	-27.707728000	30.015661000
1	-16.436000000	-25.013683000	32.849627000
1	-18.271791000	-25.219187000	31.119879000
1	-17.058518000	-25.122703000	29.846781000
1	-18.049311000	-27.273639000	29.248761000
1	-17.467055000	-28.745899000	30.040812000
7	-14.116795000	-25.719950000	31.985979000
6	-12.796177000	-26.090882000	31.510799000
6	-12.711013000	-27.242986000	30.486749000
8	-11.685318000	-27.375888000	29.822911000
1	-14.666469000	-26.403846000	32.495823000
1	-12.361383000	-25.214915000	31.027636000
7	-13.775728000	-28.081179000	30.410191000
6	-13.842658000	-29.156593000	29.425929000
6	-14.304606000	-28.642182000	28.044440000
6	-14.437396000	-29.735279000	27.005737000
6	-15.484993000	-30.668401000	27.078068000
6	-13.521614000	-29.838741000	25.950100000
6	-15.614932000	-31.666233000	26.110043000
6	-13.648291000	-30.838624000	24.982051000
6	-14.698829000	-31.755259000	25.057527000
1	-14.604209000	-27.914520000	30.971818000
1	-14.544494000	-29.907273000	29.800364000
1	-15.270388000	-28.138525000	28.180314000
1	-13.586884000	-27.886483000	27.707022000
1	-16.195568000	-30.618254000	27.900928000
1	-12.703906000	-29.123841000	25.884711000
1	-16.433955000	-32.377860000	26.177706000
1	-12.929838000	-30.897547000	24.167787000
1	-14.805161000	-32.530994000	24.303382000
6	-18.960226000	-33.844517000	36.770619000
6	-18.762032000	-35.348616000	36.566836000
8	-19.662985000	-36.170878000	36.731607000
6	-18.738541000	-32.999589000	35.499271000
6	-19.823247000	-33.263455000	34.425909000
8	-21.006547000	-33.292082000	34.861702000
8	-19.454793000	-33.417201000	33.226827000
1	-19.988720000	-33.702974000	37.105665000
1	-18.797747000	-31.938390000	35.779903000
1	-17.744682000	-33.160952000	35.067462000
7	-17.492790000	-35.711502000	36.218886000
6	-17.116572000	-37.050426000	35.805949000
6	-16.326408000	-36.995700000	34.490063000
8	-15.245255000	-36.094606000	34.730208000
6	-17.182255000	-36.582683000	33.291692000
1	-16.803427000	-35.002413000	36.003259000
1	-18.029066000	-37.643153000	35.703344000
1	-15.930398000	-38.010603000	34.303022000
1	-14.923216000	-35.731051000	33.875221000
1	-16.564674000	-36.512277000	32.389954000
1	-17.964338000	-37.328133000	33.106016000
1	-17.669204000	-35.614704000	33.451648000
6	-12.985589000	-34.338800000	30.454477000
6	-13.227978000	-35.233495000	31.658116000
8	-14.203835000	-35.031314000	32.396124000
1	-13.074844000	-33.295519000	30.770930000
1	-13.771897000	-34.523480000	29.713767000
7	-12.325048000	-36.223188000	31.838377000
6	-12.396630000	-37.211033000	32.906987000
6	-11.031873000	-37.409990000	33.569846000

1	-11.606168000	-36.359597000	31.123057000
1	-12.755631000	-38.168790000	32.500593000
1	-11.092245000	-38.186820000	34.341126000
1	-10.277442000	-37.716155000	32.835374000
1	-10.690422000	-36.481509000	34.040312000
6	-9.554159000	-36.209994000	27.490298000
6	-10.555744000	-36.787616000	28.495640000
8	-10.351082000	-36.644356000	29.713281000
6	-10.033368000	-35.930677000	26.055138000
6	-11.108543000	-34.833951000	25.894728000
6	-10.598572000	-33.453228000	26.336140000
6	-11.608607000	-34.793170000	24.442127000
1	-9.148950000	-35.306118000	27.954935000
1	-9.154874000	-35.641539000	25.459290000
1	-10.397969000	-36.861348000	25.598484000
1	-11.964371000	-35.096689000	26.532800000
1	-11.375192000	-32.691580000	26.204229000
1	-10.300445000	-33.436742000	27.390570000
1	-9.727822000	-33.150527000	25.737391000
1	-12.375755000	-34.019066000	24.319511000
1	-10.784573000	-34.554276000	23.754475000
1	-12.043952000	-35.750338000	24.131651000
7	-11.582507000	-37.494306000	27.988247000
6	-12.614898000	-38.154874000	28.780872000
6	-13.124595000	-39.409849000	28.067799000
6	-13.643142000	-39.195992000	26.631112000
8	-13.546525000	-38.019796000	26.134195000
8	-14.110174000	-40.195836000	26.034388000
1	-11.825992000	-37.432812000	27.004154000
1	-13.447537000	-37.464725000	28.965118000
1	-12.336890000	-40.172515000	28.013915000
1	-13.935284000	-39.854811000	28.656723000
8	-19.524029000	-41.173335000	30.377664000
1	-13.857348000	-41.562083000	31.965070000
1	-14.041528000	-42.604976000	29.442662000
1	-19.984755000	-40.923596000	36.066123000
1	-17.310044000	-40.589144000	35.787660000
1	-23.713617000	-43.404682000	33.322656000
1	-23.756368000	-44.869436000	31.038926000
1	-20.282620000	-45.865636000	27.232284000
1	-17.721411000	-45.102809000	26.741695000
6	-20.201443000	-46.826742000	33.892738000
1	-19.792875000	-47.790801000	33.569038000
1	-20.756509000	-46.985441000	34.828935000
1	-20.908823000	-46.488911000	33.128772000
1	-16.486522000	-37.519799000	36.576611000
1	-23.794568000	-36.994770000	35.235717000
1	-27.282340000	-37.014686000	32.011819000
1	-28.117196000	-41.205734000	33.505463000
1	-27.608958000	-41.056546000	29.146329000
1	-12.128414000	-44.487370000	20.722006000
1	-13.060182000	-42.174377000	24.440757000
1	-17.423442000	-41.253378000	21.186083000
1	-12.928161000	-40.648437000	21.631289000
1	-12.181326000	-38.414770000	29.749475000
1	-8.719402000	-36.924970000	27.456162000
1	-12.009030000	-34.502193000	29.991851000
1	-13.137356000	-36.872641000	33.633875000
1	-18.278412000	-33.508890000	37.566012000
1	-12.850785000	-29.608811000	29.341051000
1	-16.592354000	-23.590210000	31.796095000
1	-12.157233000	-26.357349000	32.361639000
8	-23.992758000	-40.753814000	33.990672000
1	-24.430256000	-39.941448000	33.654684000
1	-23.496838000	-41.068673000	33.219604000

$^4P_{Hy,AIII}$

6	-21.684982000	-45.764485000	27.184739000
6	-21.751560000	-45.148853000	31.974672000
6	-16.935984000	-44.366729000	31.868941000
6	-17.095240000	-44.105468000	27.053836000
6	-22.093655000	-45.774329000	28.505089000
6	-23.437938000	-46.080863000	28.947616000
6	-23.472164000	-45.856845000	30.288838000
6	-22.143672000	-45.441619000	30.678556000
6	-20.440621000	-44.843147000	32.391134000
6	-20.030948000	-44.654501000	33.734178000
6	-18.658027000	-44.465538000	33.706140000
6	-18.269592000	-44.534253000	32.343530000
6	-16.555413000	-44.281730000	30.548269000
6	-15.205169000	-44.018315000	30.089410000
6	-15.256319000	-43.909209000	28.737041000
6	-16.638561000	-44.126416000	28.359191000
6	-18.400028000	-44.471859000	26.642816000
6	-18.843802000	-44.536136000	25.298241000
6	-20.128675000	-45.068904000	25.333015000
6	-20.434965000	-45.294046000	26.700486000
7	-21.315719000	-45.404785000	29.580115000
7	-19.353669000	-44.757890000	31.535704000
7	-17.415708000	-44.360536000	29.470147000
7	-19.385889000	-44.915155000	27.499960000
26	-19.342333000	-44.958792000	29.527827000
1	-22.512832000	-45.197397000	32.748288000
1	-16.162598000	-44.249847000	32.622119000
1	-16.374732000	-43.817249000	26.292364000
1	-22.411335000	-46.071722000	26.438104000
6	-15.934872000	-36.178535000	29.071287000
6	-17.838665000	-37.312738000	28.692908000
6	-19.143630000	-37.824215000	28.569720000
6	-16.922370000	-39.328139000	27.601681000
6	-16.740371000	-38.079629000	28.203775000
6	-17.299822000	-36.093542000	29.239956000
6	-19.321691000	-39.066732000	27.973077000
6	-18.222449000	-39.817506000	27.495084000
7	-15.589336000	-37.359792000	28.450082000
1	-14.641349000	-37.650678000	28.208399000
6	-18.088320000	-34.965046000	29.845485000
6	-17.219260000	-33.879661000	30.501140000
7	-18.006677000	-32.788881000	31.058643000
6	-18.524004000	-31.822181000	30.289275000
8	-18.353511000	-31.788737000	29.045865000
6	-19.361558000	-30.777091000	30.996408000
1	-18.147303000	-32.786107000	32.083572000
8	-20.607701000	-39.570540000	27.889801000
6	-19.675729000	-41.893906000	30.039521000
1	-15.163457000	-35.474641000	29.351650000
1	-20.009793000	-37.272911000	28.923679000
1	-16.082538000	-39.896403000	27.212626000
1	-18.400458000	-40.782830000	27.026724000
1	-18.783836000	-35.356346000	30.599519000
1	-18.705644000	-34.481181000	29.076583000
1	-16.637829000	-34.306120000	31.323899000
1	-16.523235000	-33.465909000	29.763300000
1	-19.221699000	-29.814865000	30.496154000
1	-19.107513000	-30.676812000	32.054200000
1	-20.421420000	-31.051730000	30.911494000
1	-20.052621000	-40.892409000	29.795434000
1	-19.017007000	-42.028636000	30.907871000
1	-20.778713000	-42.523332000	27.619980000
6	-19.604767000	-48.061847000	30.768947000
16	-18.670876000	-47.095814000	29.509589000
1	-20.047249000	-47.371788000	31.489915000
1	-18.853959000	-48.652750000	31.307839000
6	-14.569573000	-37.831589000	22.011654000
6	-13.157256000	-38.281400000	22.361069000
8	-12.700750000	-39.348789000	21.923498000
6	-15.673564000	-38.683108000	22.681347000
6	-17.040730000	-38.331670000	22.058220000
6	-15.660747000	-38.506533000	24.206164000
6	-18.196303000	-39.237368000	22.500767000
1	-14.715754000	-36.774561000	22.270242000
1	-15.457305000	-39.736357000	22.449352000
1	-17.285287000	-37.286231000	22.298332000
1	-16.951986000	-38.381738000	20.963158000
1	-16.452392000	-39.089139000	24.685606000
1	-14.717144000	-38.831489000	24.656466000
1	-15.824057000	-37.454452000	24.478029000
1	-19.119876000	-38.977643000	21.969253000
1	-17.975170000	-40.291340000	22.287000000
1	-18.398373000	-39.150710000	23.573226000
7	-12.441177000	-37.427295000	23.129209000
6	-11.108459000	-37.706139000	23.644841000
6	-11.151751000	-38.065763000	25.129057000
8	-11.839082000	-37.042404000	25.827581000
1	-12.941563000	-36.700536000	23.625308000
1	-10.492023000	-36.810164000	23.497951000
1	-11.665889000	-39.025351000	25.258853000
1	-10.115966000	-38.178870000	25.494191000
1	-12.356637000	-37.498272000	26.539837000
6	-12.152177000	-42.451972000	24.417081000
6	-11.488752000	-42.936229000	23.132096000
8	-11.102313000	-44.098821000	23.008590000
6	-13.521676000	-43.117423000	24.631553000
8	-14.059157000	-42.862303000	25.919615000
1	-11.502041000	-42.737617000	25.251846000
1	-14.213158000	-42.783914000	23.839570000
1	-13.410669000	-44.202205000	24.536097000
1	-13.937147000	-41.903407000	26.130414000
7	-11.362255000	-41.997850000	22.148507000
6	-10.793901000	-42.314149000	20.851812000
6	-11.682572000	-41.857525000	19.674277000
6	-13.011033000	-42.576929000	19.583886000
6	-13.126783000	-43.789036000	18.889685000
6	-14.151831000	-42.053480000	20.209327000
6	-14.346024000	-44.464775000	18.820886000
6	-15.373066000	-42.727505000	20.142682000
6	-15.475371000	-43.934929000	19.448651000
1	-11.753411000	-41.067506000	22.279256000
1	-10.641450000	-43.396464000	20.834482000
1	-11.852417000	-40.778304000	19.765340000
1	-11.110284000	-42.017019000	18.750061000
1	-12.250612000	-44.207689000	18.397348000
1	-14.069007000	-41.113101000	20.748190000
1	-14.414314000	-45.403971000	18.276644000
1	-16.247284000	-42.306358000	20.633851000
1	-16.426796000	-44.458699000	19.396999000

6	-24.476130000	-43.427689000	25.709527000
6	-24.836506000	-41.984524000	26.053257000
8	-26.005147000	-41.573242000	25.949757000
6	-25.258899000	-43.933154000	24.480446000
8	-26.653318000	-43.910863000	24.728932000
6	-24.878581000	-45.362639000	24.108834000
1	-23.395401000	-43.532074000	25.558304000
1	-25.011270000	-43.273611000	23.627710000
1	-26.813587000	-43.031270000	25.136977000
1	-25.482068000	-45.698439000	23.258815000
1	-23.819505000	-45.435182000	23.834096000
1	-25.074526000	-46.040008000	24.948891000
7	-23.823055000	-41.205941000	26.484332000
6	-24.021464000	-39.817814000	26.894591000
6	-24.044642000	-38.807293000	25.727210000
6	-22.713863000	-38.818741000	24.950280000
6	-24.392150000	-37.416798000	26.280130000
6	-22.703939000	-37.951859000	23.685650000
1	-22.884114000	-41.597549000	26.536354000
1	-23.211389000	-39.563307000	27.586441000
1	-24.849441000	-39.122157000	25.047599000
1	-21.904744000	-38.505294000	25.624395000
1	-22.489105000	-39.854775000	24.663480000
1	-24.501683000	-36.677773000	25.479484000
1	-25.336279000	-37.439556000	26.838171000
1	-23.609037000	-37.057600000	26.960534000
1	-21.757384000	-38.065232000	23.144059000
1	-23.515316000	-38.237339000	23.003193000
1	-22.821647000	-36.887038000	23.915586000
6	-21.281015000	-26.614752000	31.578364000
6	-21.057413000	-26.723013000	33.086778000
8	-21.729551000	-26.064440000	33.887422000
6	-22.563508000	-27.335238000	31.105449000
6	-23.894877000	-26.875754000	31.733893000
6	-24.146787000	-25.372696000	31.547089000
6	-25.053364000	-27.706326000	31.160545000
1	-21.343330000	-25.546906000	31.340616000
1	-22.629417000	-27.215649000	30.012687000
1	-22.442270000	-28.412388000	31.285975000
1	-23.836926000	-27.062136000	32.813011000
1	-25.141287000	-25.096060000	31.920111000
1	-23.409104000	-24.787378000	32.102982000
1	-24.099535000	-25.086595000	30.486307000
1	-26.005765000	-27.439515000	31.635612000
1	-25.164229000	-27.539133000	30.079856000
1	-24.891842000	-28.780502000	31.314267000
7	-20.104998000	-27.607280000	33.468701000
6	-19.755600000	-27.891951000	34.852666000
6	-19.281231000	-29.349851000	34.970488000
8	-18.330269000	-29.533479000	33.938642000
6	-20.434665000	-30.357682000	34.873883000
1	-19.532997000	-28.088595000	32.785415000
1	-20.631254000	-27.691285000	35.475960000
1	-18.799206000	-29.462806000	35.955451000
1	-17.795548000	-30.358975000	34.150958000
1	-20.036764000	-31.376131000	34.824380000
1	-21.103538000	-30.281774000	35.741634000
1	-21.025776000	-30.179053000	33.968039000
6	-20.426599000	-26.262869000	26.986721000
6	-19.040779000	-25.863490000	26.472902000
8	-18.837999000	-25.634909000	25.284053000
6	-20.684444000	-27.771031000	26.833649000
6	-19.820593000	-28.625284000	27.768993000
8	-19.211811000	-28.114562000	28.727298000
7	-19.761242000	-29.937719000	27.495569000
1	-20.559354000	-25.977748000	28.034650000
1	-21.738433000	-27.985162000	27.058273000
1	-20.515443000	-28.074537000	25.793061000
1	-20.268198000	-30.316348000	26.708304000
1	-19.228438000	-30.593745000	28.105358000
7	-18.071274000	-25.790913000	27.437228000
6	-16.668212000	-25.638820000	27.101249000
6	-15.803934000	-26.915410000	27.050141000
8	-14.678079000	-26.847572000	26.560480000
1	-18.321125000	-26.179562000	28.339531000
1	-16.618258000	-25.189055000	26.108224000
7	-16.329902000	-28.045300000	27.585143000
6	-15.622795000	-29.320538000	27.523820000
6	-15.813495000	-30.025982000	26.162813000
6	-15.037584000	-31.321126000	26.055231000
6	-15.550926000	-32.516454000	26.581576000
6	-13.776733000	-31.348834000	25.442450000
6	-14.826760000	-33.707075000	26.483012000
6	-13.047985000	-32.536647000	25.351037000
6	-13.570597000	-33.724938000	25.868008000
1	-17.268364000	-28.044255000	27.971144000
1	-16.001305000	-29.955416000	28.330284000
1	-16.885331000	-30.213578000	26.021661000
1	-15.494909000	-29.330530000	25.378393000
1	-16.518028000	-32.510413000	27.081484000
1	-13.366350000	-30.427503000	25.033357000
1	-15.246766000	-34.623795000	26.887722000
1	-12.070872000	-32.534854000	24.872796000
1	-13.004592000	-34.651744000	25.800268000
6	-17.689586000	-33.978518000	36.961464000
6	-18.837826000	-34.988357000	36.836522000
8	-19.302842000	-35.520603000	37.846589000
6	-16.750900000	-33.773575000	35.749100000
6	-17.312189000	-32.798927000	34.696541000
8	-16.939681000	-31.601422000	34.789005000
8	-18.131223000	-33.271223000	33.838738000
1	-18.123449000	-33.013273000	37.260586000
1	-15.815159000	-33.341929000	36.118757000
1	-16.532188000	-34.746451000	35.296689000
7	-19.294794000	-35.223292000	35.576089000
6	-20.380474000	-36.146709000	35.310793000
6	-19.999806000	-37.286797000	34.343930000
8	-19.055545000	-38.180119000	34.911854000
6	-19.542541000	-36.767802000	32.975607000
1	-18.930280000	-34.638165000	34.814185000
1	-21.244816000	-35.605246000	34.893207000
1	-20.910778000	-37.887523000	34.203518000
1	-18.195102000	-37.719436000	35.008967000
1	-19.323770000	-37.604848000	32.303590000
1	-20.316519000	-36.143621000	32.510524000
1	-18.635376000	-36.161501000	33.073006000
6	-14.034828000	-36.791023000	34.647010000
6	-15.291403000	-37.633280000	34.487591000
8	-16.366987000	-37.250013000	34.963972000
1	-13.940543000	-36.503134000	35.698225000
1	-14.153957000	-35.867351000	34.069367000
7	-15.132189000	-38.787549000	33.796966000
6	-16.216499000	-39.725766000	33.538950000
6	-16.123738000	-40.986650000	34.405474000
1	-14.205177000	-39.007865000	33.432575000
1	-16.190268000	-39.997170000	32.475215000
1	-16.940513000	-41.677058000	34.160506000
1	-15.171145000	-41.508904000	34.250171000
1	-16.203037000	-40.726814000	35.466270000
6	-10.717622000	-37.698393000	31.746737000
6	-11.910761000	-38.655847000	31.677872000
8	-12.381451000	-39.127194000	32.726593000
6	-10.390081000	-36.830670000	30.520090000
6	-11.410362000	-35.725951000	30.164504000
6	-11.520426000	-34.663002000	31.268722000
6	-11.038578000	-35.079679000	28.821004000
1	-10.877049000	-37.079108000	32.635008000
1	-9.416831000	-36.350662000	30.700571000
1	-10.235722000	-37.474785000	29.643164000
1	-12.398562000	-36.194426000	30.048192000
1	-12.238326000	-33.884249000	30.987382000
1	-11.853638000	-35.083771000	32.224090000
1	-10.550906000	-34.173974000	31.436565000
1	-11.750699000	-34.291169000	28.551460000
1	-10.041268000	-34.620467000	28.874948000
1	-11.034330000	-35.810072000	28.003905000
7	-12.347239000	-39.006345000	30.453152000
6	-13.462396000	-39.916315000	30.221411000
6	-13.318325000	-40.630010000	28.872704000
6	-13.268728000	-39.712461000	27.634425000
8	-12.950079000	-38.488218000	27.834981000
8	-13.523946000	-40.214387000	26.514594000
1	-12.085356000	-38.484418000	29.621287000
1	-14.413040000	-39.366508000	30.255703000
1	-12.396655000	-41.229280000	28.867095000
1	-14.144866000	-41.335957000	28.749199000
8	-19.997208000	-42.850508000	29.344856000
1	-14.347137000	-43.916051000	30.742015000
1	-14.469169000	-43.708276000	28.019959000
1	-20.687641000	-44.663474000	34.594801000
1	-17.988901000	-44.284784000	34.537284000
1	-24.309879000	-45.958350000	30.967428000
1	-24.242995000	-46.398520000	28.296887000
1	-20.786199000	-45.286276000	24.500486000
1	-18.253246000	-44.260272000	24.434030000
6	-20.668141000	-48.983978000	30.169340000
1	-20.226890000	-49.671960000	29.439530000
1	-21.141685000	-49.584021000	30.959268000
1	-21.448986000	-48.410154000	29.661849000
1	-20.675070000	-36.572544000	36.272701000
1	-18.946238000	-27.222423000	35.177145000
1	-20.432670000	-27.025209000	31.017590000
1	-24.748208000	-44.055303000	26.569722000
1	-24.971295000	-39.763345000	27.436127000
1	-9.807336000	-41.836836000	20.744758000
1	-12.267801000	-41.363393000	24.436183000
1	-14.655934000	-37.916476000	20.921291000
1	-10.682608000	-38.521368000	23.055533000
1	-13.476561000	-40.642668000	31.038477000
1	-9.849396000	-38.329992000	31.983913000
1	-13.131283000	-37.310640000	34.318222000
1	-17.161489000	-39.213645000	33.731242000
1	-17.110720000	-34.325815000	37.823726000
1	-14.559629000	-29.130923000	27.695173000
1	-21.156392000	-25.716335000	26.382482000
1	-16.187010000	-24.948814000	27.804903000

8	-20.988531000	-42.094781000	26.764279000
1	-20.611824000	-40.361876000	27.310041000
1	-20.510101000	-42.626288000	26.102361000

$^4P_{OD,AIII}$

6	-21.684982000	-45.764485000	27.184739000
6	-21.751560000	-45.148853000	31.974672000
6	-16.935984000	-44.366729000	31.868941000
6	-17.095240000	-44.105468000	27.053836000
6	-22.093655000	-45.774329000	28.505089000
6	-23.437938000	-46.080863000	28.947616000
6	-23.472164000	-45.856845000	30.288838000
6	-22.143672000	-45.441619000	30.678556000
6	-20.440621000	-44.843147000	32.391134000
6	-20.030948000	-44.654501000	33.734178000
6	-18.658027000	-44.465538000	33.706140000
6	-18.269592000	-44.534253000	32.343530000
6	-16.555413000	-44.281730000	30.548269000
6	-15.205169000	-44.018315000	30.089410000
6	-15.256319000	-43.909209000	28.737041000
6	-16.638561000	-44.126416000	28.359191000
6	-18.400028000	-44.471859000	26.642816000
6	-18.843802000	-44.536136000	25.298241000
6	-20.128675000	-45.068904000	25.333015000
6	-20.434965000	-45.294046000	26.700486000
7	-21.315719000	-45.404785000	29.580115000
7	-19.353669000	-44.757890000	31.535704000
7	-17.415708000	-44.360536000	29.470147000
7	-19.385889000	-44.915155000	27.499960000
26	-19.342333000	-44.958792000	29.527827000
1	-22.512832000	-45.197397000	32.748288000
1	-16.162598000	-44.249847000	32.622119000
1	-16.374732000	-43.817249000	26.292364000
1	-22.411335000	-46.071722000	26.438104000
6	-15.934872000	-36.178535000	29.071287000
6	-17.838665000	-37.312738000	28.692908000
6	-19.143630000	-37.824215000	28.569720000
6	-16.922370000	-39.328139000	27.601681000
6	-16.740371000	-38.079629000	28.203775000
6	-17.299822000	-36.093542000	29.239956000
6	-19.321691000	-39.066732000	27.973077000
6	-18.222449000	-39.817506000	27.495084000
7	-15.589336000	-37.359792000	28.450082000
1	-14.641349000	-37.650678000	28.208399000
6	-18.088320000	-34.965046000	29.845485000
6	-17.219260000	-33.879661000	30.501140000
7	-18.006677000	-32.788881000	31.058643000
6	-18.524004000	-31.822181000	30.289275000
8	-18.353511000	-31.788737000	29.045865000
6	-19.361558000	-30.777091000	30.996408000
1	-18.147303000	-32.786107000	32.083572000
8	-20.607701000	-39.570540000	27.889801000
6	-19.675729000	-41.893906000	30.039521000
1	-15.163457000	-35.474641000	29.351650000
1	-20.009793000	-37.272911000	28.923679000
1	-16.082538000	-39.896403000	27.212626000
1	-18.400458000	-40.782830000	27.026724000
1	-18.783836000	-35.356346000	30.599519000
1	-18.705644000	-34.481181000	29.076583000
1	-16.637829000	-34.306120000	31.323899000
1	-16.523235000	-33.465909000	29.763300000
1	-19.221699000	-29.814865000	30.496154000
1	-19.107513000	-30.676812000	32.054200000
1	-20.421420000	-31.051730000	30.911494000
1	-20.052621000	-40.892409000	29.795434000
1	-19.017007000	-42.028636000	30.907871000
1	-20.778713000	-42.523332000	27.619980000
6	-19.604767000	-48.061847000	30.768947000
16	-18.670876000	-47.095814000	29.509589000
1	-20.047249000	-47.371788000	31.489915000
1	-18.853959000	-48.652750000	31.307839000
6	-14.569573000	-37.831589000	22.011654000
6	-13.157256000	-38.281400000	22.361069000
8	-12.700750000	-39.348789000	21.923498000
6	-15.673564000	-38.683108000	22.681347000
6	-17.040730000	-38.331670000	22.058220000
6	-15.660747000	-38.506533000	24.206164000
6	-18.196303000	-39.237368000	22.500767000
1	-14.715754000	-36.774561000	22.270242000
1	-15.457305000	-39.736357000	22.449352000
1	-17.285287000	-37.286231000	22.298332000
1	-16.951986000	-38.381738000	20.963158000
1	-16.452392000	-39.089139000	24.685606000
1	-14.717144000	-38.831489000	24.656466000
1	-15.824057000	-37.454452000	24.478029000
1	-19.119876000	-38.977643000	21.969253000
1	-17.975170000	-40.291340000	22.287000000
1	-18.398373000	-39.150710000	23.573226000
7	-12.441177000	-37.427295000	23.129209000
6	-11.108459000	-37.706139000	23.644841000
6	-11.151751000	-38.065763000	25.129057000
8	-11.839082000	-37.042404000	25.827581000
1	-12.941563000	-36.700536000	23.625308000

1	-10.492023000	-36.810164000	23.497951000
1	-11.665889000	-39.025351000	25.258853000
1	-10.115966000	-38.178870000	25.494191000
1	-12.356637000	-37.498272000	26.539837000
6	-12.152177000	-42.451972000	24.417081000
6	-11.488752000	-42.936229000	23.132096000
8	-11.102313000	-44.098821000	23.008590000
6	-13.521676000	-43.117423000	24.631553000
8	-14.059157000	-42.862303000	25.919615000
1	-11.502041000	-42.737617000	25.251846000
1	-14.213158000	-42.783914000	23.839570000
1	-13.410669000	-44.202205000	24.536097000
1	-13.937147000	-41.903407000	26.130414000
7	-11.362255000	-41.997850000	22.148507000
6	-10.793901000	-42.314149000	20.851812000
6	-11.682572000	-41.857525000	19.674277000
6	-13.011033000	-42.576929000	19.583886000
6	-13.126783000	-43.789036000	18.889685000
6	-14.151831000	-42.053480000	20.209327000
6	-14.346024000	-44.464775000	18.820886000
6	-15.373066000	-42.727505000	20.142682000
6	-15.475371000	-43.934929000	19.448651000
1	-11.753411000	-41.067506000	22.279256000
1	-10.641450000	-43.396464000	20.834482000
1	-11.852417000	-40.778304000	19.765340000
1	-11.110284000	-42.017019000	18.750061000
1	-12.250612000	-44.207689000	18.397348000
1	-14.069007000	-41.113101000	20.748190000
1	-14.414314000	-45.403971000	18.276644000
1	-16.247284000	-42.306358000	20.633851000
1	-16.426796000	-44.458699000	19.396999000
6	-24.476130000	-43.427689000	25.709527000
6	-24.836506000	-41.984524000	26.053257000
8	-26.005147000	-41.573242000	25.949757000
6	-25.258899000	-43.933154000	24.480446000
8	-26.653318000	-43.910863000	24.728932000
6	-24.878581000	-45.362639000	24.108834000
1	-23.395401000	-43.532074000	25.558304000
1	-25.011270000	-43.273611000	23.627710000
1	-26.813587000	-43.031270000	25.136977000
1	-25.482068000	-45.698439000	23.258815000
1	-23.819505000	-45.435182000	23.834096000
1	-25.074526000	-46.040008000	24.948891000
7	-23.823055000	-41.205941000	26.484332000
6	-24.021464000	-39.817814000	26.894591000
6	-24.044642000	-38.807293000	25.727210000
6	-22.713863000	-38.818741000	24.950280000
6	-24.392150000	-37.416798000	26.280130000
6	-22.703939000	-37.951859000	23.685650000
1	-22.884114000	-41.597549000	26.536354000
1	-23.211389000	-39.563307000	27.586441000
1	-24.849441000	-39.122157000	25.047599000
1	-21.904744000	-38.505294000	25.624395000
1	-22.489105000	-39.854775000	24.663480000
1	-24.501683000	-36.677773000	25.479484000
1	-25.336279000	-37.439556000	26.838171000
1	-23.609037000	-37.057600000	26.960534000
1	-21.757384000	-38.065232000	23.144059000
1	-23.515316000	-38.237339000	23.003193000
1	-22.821647000	-36.887038000	23.915586000
6	-21.281015000	-26.614752000	31.578364000
6	-21.057413000	-26.723013000	33.086778000
8	-21.729551000	-26.064440000	33.887422000
6	-22.563508000	-27.335238000	31.105449000
6	-23.894877000	-26.875754000	31.733893000
6	-24.146787000	-25.372696000	31.547089000
6	-25.053364000	-27.706326000	31.160545000
1	-21.343330000	-25.546906000	31.340616000
1	-22.629417000	-27.215649000	30.012687000
1	-22.442270000	-28.412388000	31.285975000
1	-23.836926000	-27.062136000	32.813011000
1	-25.141287000	-25.096060000	31.920111000
1	-23.409104000	-24.787378000	32.102982000
1	-24.099535000	-25.086595000	30.486307000
1	-26.005765000	-27.439515000	31.635612000
1	-25.164229000	-27.539133000	30.079856000
1	-24.891842000	-28.780502000	31.314267000
7	-20.104998000	-27.607280000	33.468701000
6	-19.755600000	-27.891951000	34.852666000
6	-19.281231000	-29.349851000	34.970488000
8	-18.330269000	-29.533479000	33.938642000
6	-20.434665000	-30.357682000	34.873883000
1	-19.532997000	-28.088595000	32.785415000
1	-20.631254000	-27.691285000	35.475960000
1	-18.799206000	-29.462806000	35.955451000
1	-17.795548000	-30.358975000	34.150958000
1	-20.036764000	-31.376131000	34.824380000
1	-21.103538000	-30.281774000	35.741634000
1	-21.025776000	-30.179053000	33.968039000
6	-20.426599000	-26.262869000	26.986721000
6	-19.040779000	-25.863490000	26.472902000
8	-18.837999000	-25.634909000	25.284053000
6	-20.684444000	-27.771031000	26.833649000

6	-19.820593000	-28.625284000	27.768993000
8	-19.211811000	-28.114562000	28.727298000
7	-19.761242000	-29.937719000	27.495569000
1	-20.559354000	-25.977748000	28.034650000
1	-21.738433000	-27.985162000	27.058273000
1	-20.515443000	-28.074537000	25.793061000
1	-20.268198000	-30.316348000	26.708304000
1	-19.228438000	-30.593745000	28.105358000
7	-18.071274000	-25.790913000	27.437228000
6	-16.668212000	-25.638820000	27.101249000
6	-15.803934000	-26.915410000	27.050141000
8	-14.678079000	-26.847572000	26.560480000
1	-18.321125000	-26.179562000	28.339531000
1	-16.618258000	-25.189055000	26.108224000
7	-16.329902000	-28.045300000	27.585143000
6	-15.622795000	-29.320538000	27.523820000
6	-15.813495000	-30.025982000	26.162813000
6	-15.037584000	-31.321126000	26.055231000
6	-15.550926000	-32.516454000	26.581576000
6	-13.776733000	-31.348834000	25.442450000
6	-14.826760000	-33.707075000	26.483012000
6	-13.047985000	-32.536647000	25.351037000
6	-13.570597000	-33.724938000	25.868008000
1	-17.268364000	-28.044255000	27.971144000
1	-16.001305000	-29.955416000	28.330284000
1	-16.885331000	-30.213578000	26.021661000
1	-15.494909000	-29.330530000	25.378393000
1	-16.518028000	-32.510413000	27.081484000
1	-13.366350000	-30.427503000	25.033357000
1	-15.246766000	-34.623795000	26.887722000
1	-12.070872000	-32.534854000	24.872796000
1	-13.004592000	-34.651744000	25.800268000
6	-17.689586000	-33.978518000	36.961464000
6	-18.837826000	-34.988357000	36.836522000
8	-19.302842000	-35.520603000	37.846589000
6	-16.750900000	-33.773575000	35.749100000
6	-17.312189000	-32.798927000	34.696541000
8	-16.939681000	-31.601422000	34.789005000
8	-18.131223000	-33.271223000	33.838738000
1	-18.123449000	-33.013273000	37.260586000
1	-15.815159000	-33.341929000	36.118757000
1	-16.532188000	-34.746451000	35.296689000
7	-19.294794000	-35.223292000	35.576089000
6	-20.380474000	-36.146709000	35.310793000
6	-19.999806000	-37.286797000	34.343930000
8	-19.055545000	-38.180119000	34.911854000
6	-19.542541000	-36.767802000	32.975607000
1	-18.930280000	-34.638165000	34.814185000
1	-21.244816000	-35.605246000	34.893207000
1	-20.910778000	-37.887523000	34.203518000
1	-18.195102000	-37.719436000	35.008967000
1	-19.323770000	-37.604848000	32.303590000
1	-20.316519000	-36.143621000	32.510524000
1	-18.635376000	-36.161501000	33.073006000
6	-14.034828000	-36.791023000	34.647010000
6	-15.291403000	-37.633280000	34.487591000
8	-16.366987000	-37.250013000	34.963972000
1	-13.940543000	-36.503134000	35.698225000
1	-14.153957000	-35.867351000	34.069367000
7	-15.132189000	-38.787549000	33.796966000
6	-16.216499000	-39.725766000	33.538950000
6	-16.123738000	-40.986650000	34.405474000
1	-14.205177000	-39.007865000	33.432575000
1	-16.190268000	-39.997170000	32.475215000
1	-16.940513000	-41.677058000	34.160506000
1	-15.171145000	-41.508904000	34.250171000
1	-16.203037000	-40.726814000	35.466270000
6	-10.717622000	-37.698393000	31.746737000
6	-11.910761000	-38.655847000	31.677872000
8	-12.381451000	-39.127194000	32.726593000
6	-10.390081000	-36.830670000	30.520090000
6	-11.410362000	-35.725951000	30.164504000
6	-11.520426000	-34.663002000	31.268722000
6	-11.038578000	-35.079679000	28.821004000
1	-10.877049000	-37.079108000	32.635008000
1	-9.416831000	-36.350662000	30.700571000
1	-10.235722000	-37.474785000	29.643164000
1	-12.398562000	-36.194426000	30.048192000
1	-12.238326000	-33.884249000	30.987382000
1	-11.853638000	-35.083771000	32.224090000
1	-10.550906000	-34.173974000	31.436565000
1	-11.750699000	-34.291169000	28.551460000
1	-10.041268000	-34.620467000	28.874948000
1	-11.034330000	-35.810072000	28.003905000
7	-12.347239000	-39.006345000	30.453152000
6	-13.462396000	-39.916315000	30.221411000
6	-13.318325000	-40.630010000	28.872704000
6	-13.268728000	-39.712461000	27.634425000
8	-12.950079000	-38.488218000	27.834981000
8	-13.523946000	-40.214387000	26.514594000
1	-12.085356000	-38.484418000	29.621287000
1	-14.413040000	-39.366508000	30.255703000
1	-12.396655000	-41.229280000	28.867095000
1	-14.144866000	-41.335957000	28.749199000
8	-19.997208000	-42.850508000	29.344856000
1	-14.347137000	-43.916051000	30.742015000
1	-14.469169000	-43.708276000	28.019959000
1	-20.687641000	-44.663474000	34.594801000
1	-17.988901000	-44.284784000	34.537284000
1	-24.309879000	-45.958350000	30.967428000
1	-24.242995000	-46.398520000	28.296887000
1	-20.786199000	-45.286276000	24.500486000
1	-18.253246000	-44.260272000	24.434030000
6	-20.668141000	-48.983978000	30.169340000
1	-20.226890000	-49.671960000	29.439530000
1	-21.141685000	-49.584021000	30.959268000
1	-21.448986000	-48.410154000	29.661849000
1	-20.675070000	-36.572544000	36.272701000
1	-18.946238000	-27.222423000	35.177145000
1	-20.432670000	-27.025209000	31.017590000
1	-24.748208000	-44.055303000	26.569722000
1	-24.971295000	-39.763345000	27.436127000
1	-9.807336000	-41.836836000	20.744758000
1	-12.267801000	-41.363393000	24.436183000
1	-14.655934000	-37.916476000	20.921291000
1	-10.682608000	-38.521368000	23.055533000
1	-13.476561000	-40.642668000	31.038477000
1	-9.849396000	-38.329992000	31.983913000
1	-13.131283000	-37.310640000	34.318222000
1	-17.161489000	-39.213645000	33.731242000
1	-17.110720000	-34.325815000	37.823726000
1	-14.559629000	-29.130923000	27.695173000
1	-21.156392000	-25.716335000	26.382482000
1	-16.187010000	-24.948814000	27.804903000
8	-20.988531000	-42.094781000	26.764279000
1	-20.611824000	-40.361876000	27.310041000
1	-20.510101000	-42.626288000	26.102361000

$^{1}\mathbf{IM2}_{Hy,B'}$

6	1.025718000	-0.782467000	-1.575773000
6	0.941877000	-0.159255000	-0.334595000
6	-0.114085000	0.747035000	-0.106202000
6	0.074648000	-0.519525000	-2.590214000
6	-0.969130000	0.367508000	-2.383606000
6	-1.055679000	0.997350000	-1.137001000
6	-0.489057000	1.559760000	1.026502000
6	-1.616907000	2.249656000	0.644826000
7	-1.966381000	1.914593000	-0.648364000
8	2.010786000	-1.679788000	-1.926853000
6	0.190842000	1.620158000	2.365657000
6	-0.100816000	0.401208000	3.267191000
7	-1.514349000	0.256696000	3.567500000
6	-2.026209000	0.386014000	4.829831000
6	-3.539991000	0.250028000	4.933207000
8	-1.330422000	0.582638000	5.819180000
1	1.666826000	-0.352886000	0.447368000
1	0.189362000	-1.025264000	-3.543860000
1	-1.691056000	0.564461000	-3.171712000
1	-2.196160000	2.975316000	1.200261000
1	-0.117753000	2.526707000	2.899550000
1	1.279432000	1.689592000	2.231521000
1	0.265833000	-0.515097000	2.785933000
1	0.407047000	0.507765000	4.228157000
1	-2.147712000	0.157720000	2.786747000
1	-2.727790000	2.319271000	-1.169895000
1	-3.773172000	-0.608204000	5.571350000
1	-3.940673000	1.140207000	5.427469000
1	-4.041923000	0.119256000	3.968934000
6	3.030609000	-1.999486000	-0.950266000
1	3.550056000	-1.079475000	-0.660320000
1	2.526473000	-2.437830000	-0.073088000
8	3.948241000	-2.842391000	-1.529237000
1	3.467601000	-3.662241000	-1.783956000
8	1.992660000	-4.451913000	-2.641031000
1	1.643212000	-3.539153000	-2.632621000
1	2.311774000	-4.573164000	-3.548928000

$^{1}\mathbf{TS}_{B'}$

6	1.205524000	-0.899392000	-1.354223000
6	1.068771000	-0.151843000	-0.196900000
6	-0.095463000	0.622160000	-0.048328000
6	0.239814000	-0.919771000	-2.379157000
6	-0.910715000	-0.152512000	-2.252766000
6	-1.068128000	0.607152000	-1.088397000
6	-0.572082000	1.518250000	0.978676000
6	-1.782114000	1.994285000	0.533914000
7	-2.087769000	1.450034000	-0.700004000
8	2.371142000	-1.675056000	-1.519369000
6	0.100070000	1.843174000	2.282892000
6	-0.014029000	0.723423000	3.340162000
7	-1.388434000	0.432477000	3.708107000
6	-1.914083000	0.762162000	4.929164000
6	-3.389382000	0.431258000	5.112324000
8	-1.252508000	1.269575000	5.826585000
1	1.854412000	-0.159948000	0.552453000
1	0.433377000	-1.525005000	-3.259394000

1	-1.660291000	-0.146544000	-3.039279000
1	-2.453718000	2.701355000	1.002363000
1	-0.328837000	2.759576000	2.704580000
1	1.167279000	2.043790000	2.114810000
1	0.463274000	-0.192790000	2.969349000
1	0.491698000	1.023796000	4.260289000
1	-2.005334000	0.090326000	2.985307000
1	-2.900848000	1.676510000	-1.251122000
1	-3.487592000	-0.302760000	5.918456000
1	-3.916379000	1.335523000	5.431086000
1	-3.871423000	0.033401000	4.213319000
6	2.295581000	-3.143410000	-0.912008000
1	2.446741000	-3.003483000	0.160003000
1	1.262909000	-3.427135000	-1.158590000
8	3.268128000	-3.847423000	-1.486997000
1	3.124841000	-3.670742000	-2.508045000
8	2.613666000	-2.669752000	-3.577217000
1	2.532184000	-1.949778000	-2.533013000
1	3.323450000	-2.376085000	-4.165370000

$^{1}\mathrm{P}_{\mathrm{OD,B'}}$

6	1.219462000	-0.574574000	-1.350539000
6	1.017379000	0.185946000	-0.202803000
6	-0.187795000	0.895054000	-0.058610000
6	0.230423000	-0.642620000	-2.360750000
6	-0.966135000	0.053371000	-2.237543000
6	-1.166508000	0.817947000	-1.085694000
6	-0.710122000	1.770765000	0.965876000
6	-1.951331000	2.174518000	0.530487000
7	-2.237252000	1.603862000	-0.694057000
8	2.401497000	-1.257239000	-1.467868000
6	-0.046906000	2.143363000	2.262287000
6	-0.062452000	1.013533000	3.313011000
7	-1.414565000	0.612275000	3.661771000
6	-1.897422000	0.631949000	4.939867000
6	-3.358092000	0.223298000	5.087157000
8	-1.218349000	0.944243000	5.911703000
1	1.798151000	0.223511000	0.551088000
1	0.419111000	-1.251225000	-3.240688000
1	-1.718714000	-0.001839000	-3.020155000
1	-2.655932000	2.848211000	1.000463000
1	-0.537478000	3.025055000	2.692234000
1	1.000736000	2.421147000	2.081392000
1	0.495626000	0.147025000	2.933245000
1	0.412016000	1.340567000	4.240879000
1	-2.038544000	0.388981000	2.898720000
1	-3.059695000	1.789436000	-1.245519000
1	-3.413955000	-0.656373000	5.736102000
1	-3.900994000	1.030865000	5.587810000
1	-3.853737000	-0.005670000	4.138059000
6	2.882628000	-4.408142000	-0.658165000
1	3.004269000	-5.017151000	0.257644000
1	2.717075000	-3.324341000	-0.530672000
8	2.934109000	-4.928045000	-1.757418000
1	2.654736000	-3.712670000	-3.137784000
8	2.551451000	-2.885538000	-3.658978000
1	2.438420000	-1.721554000	-2.340239000
1	3.382624000	-2.793144000	-4.148650000

$^{1}\mathrm{IM2}_{\mathrm{Hy,B''}}$

6	1.087983000	-1.000452000	-1.475550000
6	1.000646000	-0.292694000	-0.281350000
6	-0.059560000	0.622850000	-0.119750000
6	0.141308000	-0.814685000	-2.510890000
6	-0.902061000	0.085719000	-2.371028000
6	-0.995235000	0.799849000	-1.171217000
6	-0.443351000	1.510807000	0.952040000
6	-1.570535000	2.169709000	0.517451000
7	-1.910438000	1.746719000	-0.752211000
8	2.085491000	-1.908721000	-1.756368000
6	0.227639000	1.666391000	2.288058000
6	-0.045418000	0.498938000	3.260663000
7	-1.458156000	0.342576000	3.558790000
6	-1.983020000	0.530176000	4.808146000
6	-3.494390000	0.366747000	4.907809000
8	-1.299875000	0.794738000	5.790583000
1	1.731685000	-0.422295000	0.508664000
1	0.249163000	-1.401308000	-3.417947000
1	-1.623553000	0.222799000	-3.172101000
1	-2.155620000	2.929108000	1.019071000
1	-0.102613000	2.597586000	2.763363000
1	1.315632000	1.749361000	2.155776000
1	0.344553000	-0.436739000	2.838642000
1	0.452076000	0.674837000	4.216831000
1	-2.082587000	0.187521000	2.779931000
1	-2.673695000	2.107359000	-1.302501000
1	-3.714468000	-0.476117000	5.570836000
1	-3.915469000	1.263530000	5.371971000
1	-3.987625000	0.195757000	3.945354000
6	2.940642000	-2.375632000	-0.678801000
1	3.571913000	-1.541473000	-0.352267000
1	2.284600000	-2.698705000	0.144141000
8	3.751054000	-3.368886000	-1.160499000
1	3.220130000	-4.203096000	-1.243659000
8	1.880274000	-3.775247000	-3.849517000
1	1.969008000	-3.057023000	-3.184143000
1	2.724284000	-3.749102000	-4.326456000
8	2.155078000	-5.495332000	-1.739626000
1	2.659234000	-6.287696000	-1.978133000
1	1.937308000	-5.057047000	-2.600555000

$^{1}\mathrm{TS}_{\mathrm{B''}}$

6	1.077578000	-1.196303000	-1.299188000
6	1.008325000	-0.372494000	-0.187888000
6	-0.092890000	0.494777000	-0.081863000
6	0.108624000	-1.203075000	-2.320520000
6	-0.977339000	-0.341461000	-2.236298000
6	-1.070845000	0.492711000	-1.117047000
6	-0.494670000	1.481821000	0.892675000
6	-1.669385000	2.019641000	0.424467000
7	-2.022573000	1.430197000	-0.775464000
8	2.177068000	-2.077058000	-1.401261000
6	0.208831000	1.833031000	2.173368000
6	0.044360000	0.775976000	3.286764000
7	-1.340761000	0.577124000	3.674880000
6	-1.834638000	0.970150000	4.890334000
6	-3.323832000	0.726179000	5.095814000
8	-1.137213000	1.466399000	5.766623000
1	1.797835000	-0.393932000	0.557216000
1	0.261769000	-1.865526000	-3.167465000
1	-1.727951000	-0.321892000	-3.021679000
1	-2.283823000	2.799851000	0.853621000
1	-0.164373000	2.792084000	2.550957000
1	1.283759000	1.964789000	1.986942000
1	0.471155000	-0.181482000	2.960758000
1	0.571821000	1.094798000	4.188280000
1	-1.982279000	0.244641000	2.969144000
1	-2.821704000	1.682532000	-1.335638000
1	-3.453704000	0.021500000	5.923377000
1	-3.797290000	1.666589000	5.393240000
1	-3.836104000	0.330372000	4.212773000
6	2.001261000	-3.489205000	-0.564809000
1	1.755677000	-3.086991000	0.418303000
1	1.144818000	-3.918934000	-1.095600000
8	3.127092000	-4.159613000	-0.602863000
1	3.168822000	-4.699562000	-1.505637000
8	2.272994000	-2.988577000	-3.589905000
1	2.298035000	-2.376472000	-2.334942000
1	2.880277000	-2.547041000	-4.201300000
8	3.037248000	-5.233867000	-2.877775000
1	3.926996000	-5.408572000	-3.220384000
1	2.719061000	-4.302279000	-3.328456000

$^{1}\mathrm{P}_{\mathrm{OD,B''}}$

6	1.213633000	-0.884437000	-1.395227000
6	1.059328000	-0.078435000	-0.270369000
6	-0.106629000	0.696559000	-0.141388000
6	0.215128000	-0.933543000	-2.398134000
6	-0.941479000	-0.171265000	-2.289790000
6	-1.094648000	0.637542000	-1.160767000
6	-0.575264000	1.631038000	0.856518000
6	-1.796555000	2.084718000	0.414153000
7	-2.119637000	1.490745000	-0.789910000
8	2.354404000	-1.637678000	-1.489638000
6	0.115354000	2.011765000	2.136138000
6	0.052557000	0.918862000	3.223505000
7	-1.314063000	0.593920000	3.593661000
6	-1.787894000	0.691724000	4.872088000
6	-3.266271000	0.364236000	5.042529000
8	-1.088219000	1.012159000	5.826270000
1	1.850059000	-0.050056000	0.473824000
1	0.371223000	-1.572720000	-3.261973000
1	-1.700334000	-0.209059000	-3.067327000
1	-2.462100000	2.808944000	0.865447000
1	-0.328307000	2.929847000	2.539951000
1	1.173889000	2.232108000	1.940059000
1	0.566270000	0.014501000	2.869934000
1	0.548485000	1.253127000	4.137441000
1	-1.953817000	0.372084000	2.843418000
1	-2.936045000	1.699561000	-1.342141000
1	-3.364048000	-0.457370000	5.758763000
1	-3.772436000	1.233229000	5.474535000
1	-3.770259000	0.084601000	4.111556000
6	2.254326000	-3.701998000	0.059860000
1	2.506454000	-2.975747000	0.849332000
1	1.223133000	-3.656185000	-0.327639000
8	3.055476000	-4.542630000	-0.307767000
1	2.705461000	-5.375314000	-1.863664000
8	2.411419000	-3.067540000	-3.788924000
1	2.378024000	-2.122270000	-2.358825000
1	3.224780000	-2.891751000	-4.284534000
8	2.576819000	-5.604894000	-2.813954000
1	3.373547000	-6.101552000	-3.053130000
1	2.460268000	-4.026000000	-3.530329000

$^{1}\mathrm{IM2}_{\mathrm{Hy,B'''}}$

6	0.878032000	-1.065328000	-1.551219000
6	0.861475000	-0.360849000	-0.351277000
6	-0.150514000	0.600044000	-0.147975000
6	-0.095661000	-0.827681000	-2.550467000
6	-1.092202000	0.117459000	-2.371311000
6	-1.112391000	0.827073000	-1.165171000
6	-0.456703000	1.496101000	0.941897000
6	-1.566863000	2.208879000	0.550442000
7	-1.968563000	1.811553000	-0.709386000
8	1.815003000	-2.019253000	-1.874778000
6	0.265590000	1.608215000	2.255312000
6	-0.031295000	0.446602000	3.227866000
7	-1.439617000	0.356392000	3.570631000
6	-1.912426000	0.535962000	4.841656000
6	-3.426212000	0.447505000	4.988782000
8	-1.184535000	0.738537000	5.806580000
1	1.610991000	-0.527784000	0.414048000
1	-0.046802000	-1.414170000	-3.462530000
1	-1.833354000	0.292442000	-3.146643000
1	-2.099254000	2.990069000	1.076694000
1	-0.002282000	2.550169000	2.748451000
1	1.351217000	1.639181000	2.086472000
1	0.299353000	-0.502689000	2.785761000
1	0.504839000	0.588931000	4.168646000
1	-2.096417000	0.250944000	2.810279000
1	-2.733432000	2.210165000	-1.230467000
1	-3.667908000	-0.399071000	5.639413000
1	-3.784807000	1.352921000	5.487423000
1	-3.958987000	0.325863000	4.039990000
6	2.705477000	-2.475727000	-0.845455000
1	3.390950000	-1.665627000	-0.568243000
1	2.105391000	-2.775468000	0.026085000
8	3.459264000	-3.522921000	-1.343136000
1	2.886437000	-4.345345000	-1.369426000
8	1.885745000	-5.434735000	-4.274715000
1	2.419595000	-6.073737000	-4.769565000
1	2.407043000	-4.586514000	-4.319701000
8	3.370941000	-3.170307000	-4.111431000
1	2.707550000	-2.470905000	-3.988066000
1	3.668228000	-3.300533000	-3.182261000
8	1.906980000	-5.674378000	-1.588147000
1	2.382089000	-6.491600000	-1.376008000
1	1.802543000	-5.683641000	-2.580354000

$^{1}TS_{B'''}$

6	1.156178000	-1.348139000	-1.213850000
6	1.202181000	-0.776910000	0.046548000
6	0.383637000	0.338636000	0.293664000
6	0.335028000	-0.868091000	-2.251955000
6	-0.474056000	0.237883000	-2.025578000
6	-0.444259000	0.826779000	-0.756663000
6	0.175454000	1.189721000	1.441678000
6	-0.748230000	2.130099000	1.053281000
7	-1.127775000	1.915868000	-0.259334000
8	1.981818000	-2.466298000	-1.466090000
6	0.815602000	1.061052000	2.795346000
6	0.233581000	-0.082367000	3.654278000
7	-1.181366000	0.089123000	3.933225000
6	-1.662241000	0.360577000	5.181913000
6	-3.166947000	0.569485000	5.270577000
8	-0.955856000	0.418704000	6.160287000
1	1.871188000	-1.180569000	0.800316000
1	0.359373000	-1.353732000	-3.224236000
1	-1.105010000	0.630910000	-2.817954000
1	-1.160424000	2.957808000	1.614898000
1	0.705278000	2.000416000	3.349488000
1	1.894633000	0.886237000	2.684047000
1	0.391114000	-1.045323000	3.151295000
1	0.734371000	-0.118063000	4.624106000
1	-1.812118000	0.128415000	3.145165000
1	-1.755884000	2.501708000	-0.786975000
1	-3.588295000	-0.189376000	5.937305000
1	-3.361380000	1.545283000	5.726013000
1	-3.681141000	0.516149000	4.305409000
6	1.308591000	-3.829574000	-1.254321000
1	1.237428000	-3.899667000	-0.166668000
1	0.322586000	-3.732491000	-1.722654000
8	2.086406000	-4.796760000	-1.768705000
1	1.766778000	-4.998298000	-2.701983000
8	1.137118000	-2.787079000	-4.997992000
1	1.316242000	-2.535159000	-5.915520000
1	2.006346000	-2.554299000	-4.450289000
8	3.075013000	-2.294286000	-3.483479000
1	2.516899000	-2.408309000	-2.385432000
1	3.745460000	-2.992159000	-3.525895000
8	0.992744000	-5.249875000	-4.123222000
1	1.511470000	-5.836327000	-4.693283000
1	0.986972000	-4.354962000	-4.588067000

$^{1}P_{OD,B'''}$

6	1.282454000	-0.863069000	-1.454465000
6	1.257795000	-0.218029000	-0.220873000
6	0.249539000	0.726717000	0.035269000
6	0.305959000	-0.580702000	-2.438917000
6	-0.696794000	0.353687000	-2.206737000
6	-0.717765000	1.001000000	-0.969374000
6	-0.056255000	1.571796000	1.167106000
6	-1.167810000	2.300464000	0.812580000
7	-1.576504000	1.961032000	-0.462368000
8	2.275457000	-1.779816000	-1.673509000
6	0.671302000	1.622833000	2.481342000
6	0.368552000	0.422860000	3.403705000
7	-1.041559000	0.329918000	3.740620000
6	-1.513224000	0.416716000	5.021054000
6	-3.029726000	0.358759000	5.158806000
8	-0.784636000	0.521731000	6.001059000
1	2.027829000	-0.447197000	0.509787000
1	0.347698000	-1.107045000	-3.388111000
1	-1.439181000	0.567648000	-2.971607000
1	-1.697707000	3.057466000	1.375800000
1	0.412955000	2.545071000	3.015563000
1	1.756031000	1.650983000	2.308496000
1	0.693955000	-0.507919000	2.920278000
1	0.905189000	0.519301000	4.350027000
1	-1.698454000	0.306550000	2.973164000
1	-2.331952000	2.396544000	-0.966993000
1	-3.294167000	-0.506672000	5.774506000
1	-3.369377000	1.252069000	5.691920000
1	-3.561236000	0.288745000	4.204023000
6	1.954849000	-4.240682000	-0.576208000
1	1.832047000	-3.671977000	0.361179000
1	1.118047000	-4.187102000	-1.293110000
8	2.943169000	-4.919321000	-0.781319000
1	3.022950000	-5.473185000	-2.555450000
8	0.141743000	-4.907899000	-3.698455000
1	-0.522401000	-5.168161000	-4.353494000
1	0.550923000	-4.088321000	-4.045343000
8	2.168495000	-3.026694000	-4.125328000
1	2.245235000	-2.110791000	-2.603300000
1	2.631178000	-2.653167000	-4.890495000
8	2.850160000	-5.492738000	-3.523713000
1	2.615691000	-3.911889000	-3.929151000
1	1.894054000	-5.702774000	-3.589267000

$^{2}Re_{AII}$

6	43.219075000	47.597348000	31.763620000
6	44.065572000	46.638525000	30.924275000
8	43.822277000	46.448796000	29.729483000
6	41.708486000	47.377667000	31.550340000
6	40.900914000	48.534105000	32.171748000
6	41.272397000	46.005415000	32.087169000
6	39.403619000	48.518925000	31.838922000
1	43.480741000	47.528038000	32.826299000
1	41.546698000	47.385938000	30.463607000
1	41.028497000	48.520357000	33.264201000
1	41.327965000	49.486253000	31.825643000
1	40.218268000	45.800138000	31.868899000
1	41.862806000	45.202152000	31.634228000
1	41.403210000	45.948252000	33.175987000
1	38.901489000	47.639084000	32.256229000
1	38.902300000	49.405988000	32.244994000
1	39.241592000	48.511800000	30.753074000
7	45.095370000	46.034383000	31.573186000
6	46.034428000	45.181054000	30.855952000
6	45.584569000	43.729378000	30.693417000
8	45.597181000	43.077957000	31.947943000
1	45.290007000	46.279461000	32.541761000
1	46.987065000	45.202781000	31.395965000
1	44.584925000	43.719947000	30.233198000
1	46.277636000	43.239501000	29.987906000
1	45.305916000	42.140272000	31.811279000
6	36.139429000	39.103105000	34.909639000
6	35.067969000	39.694004000	35.812706000
8	34.793853000	39.172913000	36.898813000
6	37.466156000	38.950139000	35.682961000
8	37.356075000	38.034259000	36.756610000
1	35.804747000	38.108239000	34.592353000
1	38.234763000	38.561129000	35.007890000
1	37.804069000	39.938014000	36.030069000
1	36.546821000	38.285800000	37.243124000
7	34.443842000	40.819846000	35.362560000
6	33.548118000	41.612460000	36.193722000
6	34.176539000	42.944001000	36.664192000
6	35.442424000	42.755636000	37.472153000
6	35.379736000	42.371118000	38.818660000
6	36.703045000	42.904048000	36.879097000
6	36.543602000	42.124092000	39.546753000
6	37.872502000	42.654522000	37.603040000
6	37.794958000	42.257879000	38.940015000
1	34.795798000	41.241226000	34.513918000
1	33.297536000	40.983809000	37.050664000
1	34.382581000	43.572959000	35.787186000
1	33.416528000	43.474395000	37.253702000
1	34.409631000	42.254645000	39.298183000
1	36.773255000	43.219091000	35.839544000
1	36.474315000	41.822133000	40.588477000

1	38.842157000	42.763666000	37.125582000
1	38.704820000	42.042200000	39.490809000
6	45.989337000	53.512411000	38.170169000
6	44.697457000	54.287112000	37.899818000
8	44.292717000	55.144079000	38.680143000
6	45.801203000	52.409525000	39.225220000
6	45.086771000	51.164711000	38.684755000
7	45.065722000	50.108064000	39.499936000
8	44.566304000	51.163026000	37.550468000
1	46.398170000	53.080114000	37.251538000
1	46.775886000	52.106766000	39.627735000
1	45.228154000	52.809654000	40.071851000
1	45.445775000	50.115518000	40.444966000
1	44.558075000	49.244411000	39.278238000
7	44.053097000	53.960094000	36.735977000
6	42.724720000	54.455847000	36.426483000
6	41.556376000	53.460950000	36.573218000
8	40.438124000	53.794757000	36.185733000
1	44.363809000	53.105645000	36.289821000
1	42.534700000	55.294927000	37.100889000
7	41.858441000	52.270319000	37.144703000
6	40.897166000	51.191453000	37.312513000
6	41.323343000	49.931131000	36.528720000
6	40.446296000	48.735541000	36.830016000
6	39.222125000	48.557109000	36.169583000
6	40.827177000	47.794688000	37.798685000
6	38.399743000	47.467939000	36.463645000
6	40.005950000	46.703217000	38.091985000
6	38.788983000	46.534820000	37.426890000
1	42.824968000	52.053323000	37.376799000
1	39.927902000	51.563442000	36.972247000
1	41.299619000	50.164652000	35.456643000
1	42.365066000	49.704521000	36.783333000
1	38.915957000	49.276724000	35.412557000
1	41.772961000	47.912103000	38.324179000
1	37.457646000	47.345064000	35.934044000
1	40.320886000	45.979335000	38.839641000
1	38.157002000	45.679723000	37.651237000
6	46.820611000	50.351680000	32.504177000
6	48.205843000	49.714979000	32.559406000
7	48.245929000	48.500947000	33.169499000
8	49.196091000	50.266818000	32.085459000
1	46.058788000	49.763785000	33.023090000
1	46.529830000	50.469215000	31.454630000
1	49.134840000	48.020881000	33.190563000
1	47.411777000	48.037152000	33.538381000
6	49.253129000	38.869761000	34.752481000
6	47.789695000	38.523559000	35.033420000
8	47.400015000	38.329030000	36.204479000
6	49.517221000	39.841190000	33.585637000
6	48.886979000	41.243256000	33.728113000
6	49.529141000	42.054248000	34.863849000
6	48.958039000	41.997834000	32.393205000
1	49.657004000	39.259596000	35.691626000
1	50.606679000	39.949701000	33.476160000
1	49.168953000	39.394437000	32.645930000
1	47.821063000	41.115293000	33.958925000
1	49.439008000	41.555675000	35.836540000
1	50.598666000	42.219301000	34.670823000
1	49.053002000	43.037291000	34.954529000
1	50.000703000	42.142819000	32.074430000
1	48.418160000	41.443267000	31.619595000
1	48.481795000	42.979988000	32.479620000
7	46.985627000	38.398445000	33.967202000
6	45.541371000	38.204877000	34.073237000
6	44.904162000	38.294581000	32.673605000
6	45.350363000	39.587863000	31.948215000
8	46.544960000	39.612936000	31.561082000
8	44.501274000	40.528367000	31.847474000
1	47.271333000	38.750795000	33.045440000
1	45.112658000	38.970547000	34.732726000
1	45.225508000	37.432958000	32.075679000
1	43.815350000	38.254136000	32.777992000
6	45.837106000	41.062531000	37.854892000
6	44.925404000	40.007349000	38.445212000
8	44.126338000	40.302086000	39.360438000
1	46.266523000	41.659206000	38.664245000
1	45.238370000	41.732741000	37.227841000
7	44.999304000	38.779996000	37.908250000
6	44.171828000	37.668308000	38.372875000
6	43.977063000	36.615059000	37.287665000
1	45.742524000	38.586514000	37.234220000
1	43.204551000	38.073911000	38.670102000
1	44.934038000	36.192201000	36.961641000
1	43.362965000	35.801786000	37.687515000
1	43.467981000	37.041597000	36.416236000
6	44.298029000	44.033591000	40.957460000
6	43.745365000	43.169567000	42.092549000
8	43.498161000	43.639212000	43.208657000
6	43.606097000	45.398400000	40.933047000
6	44.118761000	46.396838000	39.885342000
8	43.841282000	47.630409000	40.121996000
8	44.726595000	45.961492000	38.884923000

1	45.373077000	44.176563000	41.132200000
1	42.532126000	45.260110000	40.738915000
1	43.669134000	45.850988000	41.925741000
7	43.557327000	41.858678000	41.793840000
6	43.014262000	40.931423000	42.776877000
6	43.302092000	39.465374000	42.433472000
6	42.884025000	38.540488000	43.572138000
8	42.575070000	39.063365000	41.264283000
1	43.752864000	41.521860000	40.853509000
1	41.925138000	41.057382000	42.865579000
1	44.379960000	39.345197000	42.249575000
1	43.039935000	39.430929000	40.476675000
1	41.812463000	38.648180000	43.779287000
1	43.082631000	37.500135000	43.298829000
1	43.442460000	38.768511000	44.486336000
6	38.965161000	33.123525000	42.298322000
6	37.661727000	33.239125000	41.498396000
8	36.611674000	32.755405000	41.923728000
6	39.962483000	34.286274000	42.199678000
6	41.115248000	34.122059000	43.194665000
8	40.451364000	34.326830000	40.862177000
1	38.669051000	32.969191000	43.340257000
1	39.430033000	35.225358000	42.433072000
1	41.071050000	35.096514000	40.783583000
1	41.637411000	33.173020000	43.023946000
1	41.838929000	34.932746000	43.064578000
1	40.752521000	34.142254000	44.230270000
7	37.775715000	33.869009000	40.296407000
6	36.668582000	34.007055000	39.366748000
6	36.271924000	35.466849000	39.057537000
6	35.796279000	36.200065000	40.325891000
6	35.214681000	35.476030000	37.941865000
6	35.650087000	37.718808000	40.168435000
1	38.708810000	34.200218000	40.050929000
1	35.824162000	33.470052000	39.810173000
1	37.164852000	35.984443000	38.674789000
1	34.838222000	35.765410000	40.648877000
1	36.509482000	35.994326000	41.133418000
1	34.952301000	36.491886000	37.631914000
1	35.579854000	34.947639000	37.052209000
1	34.294660000	34.975264000	38.274537000
1	35.336492000	38.179976000	41.113178000
1	36.605015000	38.177767000	39.882142000
1	34.913755000	37.994815000	39.406044000
6	50.314192000	32.133239000	42.768921000
6	48.894016000	32.678302000	42.608148000
16	48.866090000	34.477839000	42.288895000
1	50.907841000	32.312594000	41.866198000
1	48.388575000	32.187907000	41.766651000
1	48.289638000	32.470891000	43.498734000
6	40.360070000	47.243862000	42.450564000
6	41.200321000	48.336524000	43.103627000
8	40.862771000	48.853681000	44.173049000
6	40.463103000	45.862525000	43.138084000
6	40.125487000	45.793625000	44.640681000
6	38.723206000	46.329337000	44.963418000
6	40.298269000	44.346230000	45.129985000
1	39.322876000	47.596229000	42.477868000
1	39.789400000	45.180473000	42.596565000
1	41.473831000	45.462047000	43.000108000
1	40.847069000	46.424649000	45.174964000
1	38.656719000	47.399784000	44.747739000
1	37.953068000	45.802626000	44.381292000
1	38.487380000	46.186713000	46.026350000
1	41.285246000	43.951119000	44.862847000
1	40.179862000	44.280215000	46.219526000
1	39.542453000	43.688887000	44.676828000
7	42.327441000	48.702367000	42.427850000
6	43.206057000	49.748264000	42.923794000
6	44.629355000	49.272304000	43.266686000
6	44.658519000	48.130411000	44.284632000
8	45.382291000	48.941368000	42.096258000
1	42.595178000	48.237240000	41.557042000
1	42.724807000	50.157869000	43.815799000
1	45.152357000	50.138846000	43.695150000
1	44.859402000	48.317903000	41.537791000
1	45.695221000	47.867973000	44.519810000
1	44.145435000	48.416923000	45.210437000
1	44.163088000	47.236754000	43.891151000
8	44.948448000	35.755861000	41.497159000
26	46.441657000	35.222579000	41.891253000
7	45.744974000	33.530132000	42.746940000
7	46.441380000	36.130099000	43.693841000
7	46.678562000	34.230605000	40.143084000
6	45.709536000	32.067583000	40.777092000
6	45.411249000	34.354108000	45.034280000
6	47.421102000	38.287088000	43.056002000
6	47.583875000	36.053294000	38.767419000
6	45.449872000	32.351710000	42.113595000
6	45.936364000	35.628698000	44.863794000
6	47.641228000	38.016145000	41.713586000
6	47.121496000	34.758443000	38.954573000
6	44.834105000	31.430319000	43.037053000

6	46.034461000	36.616483000	45.912241000
6	48.169760000	38.968285000	40.762911000
6	47.020760000	33.771375000	37.906126000
6	44.752185000	32.072979000	44.235678000
6	46.606638000	37.720293000	45.357018000
6	48.195112000	38.345167000	39.554324000
6	46.501747000	32.647364000	38.471104000
6	45.318028000	33.386100000	44.042686000
6	46.853441000	37.404774000	43.969702000
6	47.696389000	37.006432000	39.770650000
6	46.277143000	32.947519000	39.865468000
7	47.368873000	36.824909000	41.089296000
1	45.424416000	31.085584000	40.411448000
1	45.036504000	34.096135000	46.020326000
1	47.690853000	39.274781000	43.417152000
1	47.858629000	36.361806000	37.763766000
6	43.471421000	42.641622000	34.266162000
6	42.340282000	41.919722000	36.081696000
6	41.731016000	41.674678000	37.314830000
6	41.666713000	39.687551000	35.288986000
6	42.304812000	40.908722000	35.072330000
6	43.097233000	43.027474000	35.541663000
6	41.101378000	40.436474000	37.537633000
6	41.063021000	39.452666000	36.528718000
7	42.991127000	41.391140000	33.978292000
6	43.456012000	44.314529000	36.228203000
6	44.370121000	45.181074000	35.356779000
7	44.897996000	46.319781000	36.097128000
6	45.644585000	47.251377000	35.493813000
8	45.852187000	47.249938000	34.256157000
6	46.218263000	48.331663000	36.387290000
8	40.553458000	40.279453000	38.777142000
6	39.974922000	39.022248000	39.119732000
1	44.082095000	43.147168000	33.526497000
1	41.759662000	42.395385000	38.126110000
1	47.228026000	48.578950000	36.046717000
1	40.713227000	38.213607000	39.058816000
1	39.116326000	38.785687000	38.479474000
1	39.655528000	39.123282000	40.157703000
1	41.636914000	38.925187000	34.514597000
1	40.565069000	38.507206000	36.706534000
1	42.552660000	44.885829000	36.489845000
1	43.957772000	44.113293000	37.185072000
1	43.824503000	45.540587000	34.477580000
1	45.206021000	44.579799000	34.975708000
1	46.242704000	48.020716000	37.435053000
1	45.607540000	49.239290000	36.318356000
1	43.325292000	40.880119000	33.142529000
1	44.817264000	46.321415000	37.124557000
8	42.278586000	36.340423000	40.701931000
1	43.178797000	36.021099000	40.914714000
1	42.289553000	37.290768000	40.962880000
1	50.828996000	32.612217000	43.609214000
1	50.291078000	31.051170000	42.957261000
1	48.486420000	38.725303000	38.584156000
1	48.459103000	39.982641000	41.007191000
1	46.841975000	38.668415000	45.823882000
1	45.701870000	36.468078000	46.932012000
1	44.342933000	31.709975000	45.170204000
1	44.506524000	30.430005000	42.782790000
1	46.271816000	31.699047000	38.001546000
1	47.310724000	33.940300000	36.876870000
1	43.446925000	41.184859000	43.750780000
1	44.205094000	43.530139000	39.990298000
1	43.283500000	50.548134000	42.174386000
1	40.630774000	47.132228000	41.393558000
1	36.299192000	39.711544000	34.010540000
1	32.623934000	41.816441000	35.637026000
1	36.925828000	33.502420000	38.423502000
1	39.481709000	32.210092000	41.969044000
1	46.186753000	45.608309000	29.858605000
1	43.482525000	48.614406000	31.439614000
1	46.873435000	51.353753000	32.941995000
1	40.807654000	50.943555000	38.378392000
1	42.682255000	54.837615000	35.400204000
1	46.707357000	54.244021000	38.553887000
1	44.619412000	37.214919000	39.264947000
1	46.635462000	40.631072000	37.246355000
1	45.330229000	37.232845000	34.534406000
1	49.779659000	37.922928000	34.562510000

$^{4}Re_{AII}$

6	43.238751000	47.603469000	31.770121000
6	44.077896000	46.640807000	30.927934000
8	43.832481000	46.455577000	29.732892000
6	41.726389000	47.392216000	31.561094000
6	40.927526000	48.553427000	32.184747000
6	41.284193000	46.022591000	32.099664000
6	39.428554000	48.545834000	31.859355000
1	43.503010000	47.532220000	32.832000000
1	41.561299000	47.401238000	30.474853000
1	41.060290000	48.540315000	33.276586000
1	41.357929000	49.502897000	31.835465000
1	40.228415000	45.822945000	31.884186000
1	41.869216000	45.215934000	31.645714000
1	41.417407000	45.965415000	33.188209000
1	38.923302000	47.670559000	32.282377000
1	38.934950000	49.437496000	32.264793000
1	39.261055000	48.535964000	30.774352000
7	45.104265000	46.028388000	31.574697000
6	46.038196000	45.172114000	30.854331000
6	45.583663000	43.721695000	30.693929000
8	45.600114000	43.070487000	31.948545000
1	45.300315000	46.269237000	32.544037000
1	46.993075000	45.191154000	31.390520000
1	44.581857000	43.715006000	30.238247000
1	46.272032000	43.229653000	29.985386000
1	45.309109000	42.132655000	31.812788000
6	36.149580000	39.144049000	34.914470000
6	35.075870000	39.725741000	35.820831000
8	34.795266000	39.190740000	36.898473000
6	37.472216000	38.976313000	35.691866000
8	37.354539000	38.044713000	36.751061000
1	35.813541000	38.154629000	34.581935000
1	38.243329000	38.595913000	35.014823000
1	37.810489000	39.958130000	36.055432000
1	36.543110000	38.290786000	37.236695000
7	34.457325000	40.859672000	35.383346000
6	33.561318000	41.644475000	36.221570000
6	34.193966000	42.965505000	36.715439000
6	35.455191000	42.759377000	37.526259000
6	35.384775000	42.357994000	38.867410000
6	36.719163000	42.909004000	36.940536000
6	36.544322000	42.096401000	39.597396000
6	37.884228000	42.644724000	37.666223000
6	37.798887000	42.231880000	38.997772000
1	34.813796000	41.290872000	34.541539000
1	33.302269000	41.004312000	37.067414000
1	34.407058000	43.606666000	35.849010000
1	33.433703000	43.490489000	37.309430000
1	34.412073000	42.240100000	39.341279000
1	36.795476000	43.236818000	35.905384000
1	36.469113000	41.782145000	40.635055000
1	38.856344000	42.755129000	37.194090000
1	38.705253000	42.006546000	39.550399000
6	45.968122000	53.514542000	38.168401000
6	44.676197000	54.286261000	37.889983000
8	44.266399000	55.144830000	38.665898000
6	45.776045000	52.410448000	39.221539000
6	45.069564000	51.163185000	38.676016000
7	45.045558000	50.107267000	39.492081000
8	44.558476000	51.158757000	37.537433000
1	46.383928000	53.083743000	37.252185000
1	46.749014000	52.110523000	39.630271000
1	45.196315000	52.808191000	40.064732000
1	45.415818000	50.117955000	40.441059000
1	44.544231000	49.241093000	39.266707000
7	44.037608000	53.954442000	36.724349000
6	42.709338000	54.446067000	36.408099000
6	41.542379000	53.449934000	36.557258000
8	40.423775000	53.780711000	36.168283000
1	44.352591000	53.099607000	36.281835000
1	42.515613000	55.288528000	37.077189000
7	41.845976000	52.261646000	37.132973000
6	40.886526000	51.181558000	37.302668000
6	41.309380000	49.923442000	36.513509000
6	40.434846000	48.726640000	36.817378000
6	39.200668000	48.555902000	36.173593000
6	40.827469000	47.778064000	37.773710000
6	38.379573000	47.467197000	36.472775000
6	40.007855000	46.686597000	38.071501000
6	38.780505000	46.526466000	37.423769000
1	42.813174000	52.045964000	37.363275000
1	39.915286000	51.553622000	36.968216000
1	41.279550000	50.159560000	35.442130000
1	42.352680000	49.696992000	36.761785000
1	38.885119000	49.281669000	35.426370000
1	41.780278000	47.890479000	38.287432000
1	37.428078000	47.351150000	35.958585000
1	40.331603000	45.956969000	38.809727000
1	38.148390000	45.672909000	37.653478000
6	46.829321000	50.349786000	32.518655000
6	48.213888000	49.711565000	32.572624000
7	48.253298000	48.497261000	33.182308000
8	49.204473000	50.262995000	32.098945000
1	46.066243000	49.760277000	33.033868000
1	46.540072000	50.472675000	31.469320000
1	49.141294000	48.015332000	33.199990000
1	47.418221000	48.031370000	33.546425000
6	49.263824000	38.873543000	34.748853000
6	47.801289000	38.525061000	35.031528000
8	47.412764000	38.332501000	36.203255000
6	49.525418000	39.842648000	33.579521000
6	48.892813000	41.243946000	33.718854000
6	49.533766000	42.058753000	34.852510000
6	48.962364000	41.995359000	32.382062000

1	49.667461000	39.266397000	35.686834000
1	50.614617000	39.952813000	33.469148000
1	49.177419000	39.393000000	32.641098000
1	47.827145000	41.114802000	33.950115000
1	49.444461000	41.562424000	35.826420000
1	50.603019000	42.225039000	34.659041000
1	49.056050000	43.041248000	34.940778000
1	50.004740000	42.142025000	32.063123000
1	48.424036000	41.437389000	31.609792000
1	48.483627000	42.976489000	32.465956000
7	46.996645000	38.395793000	33.966176000
6	45.552852000	38.199631000	34.074070000
6	44.913934000	38.286266000	32.675027000
6	45.357004000	39.579577000	31.947855000
8	46.550554000	39.605819000	31.557883000
8	44.506641000	40.519275000	31.849139000
1	47.281029000	38.746694000	33.043548000
1	45.123537000	38.965320000	34.733142000
1	45.236322000	37.424526000	32.077861000
1	43.825307000	38.243847000	32.780651000
6	45.857610000	41.062461000	37.859391000
6	44.942189000	40.009797000	38.448525000
8	44.143127000	40.306582000	39.363068000
1	46.291129000	41.655065000	38.669553000
1	45.260891000	41.736984000	37.235129000
7	45.013195000	38.782272000	37.911471000
6	44.183043000	37.672641000	38.376087000
6	43.988784000	36.618166000	37.292087000
1	45.755880000	38.587203000	37.237334000
1	43.215806000	38.080014000	38.670922000
1	44.945555000	36.192268000	36.969474000
1	43.371883000	35.806873000	37.691647000
1	43.482898000	37.044378000	36.418656000
6	44.327598000	44.033794000	40.972369000
6	43.774024000	43.167411000	42.105130000
8	43.524414000	43.635072000	43.221477000
6	43.627616000	45.394418000	40.944062000
6	44.126101000	46.389162000	39.886101000
8	43.836950000	47.621763000	40.113274000
8	44.733623000	45.951389000	38.886473000
1	45.401018000	44.182898000	41.151763000
1	42.553401000	45.248196000	40.757760000
1	43.693877000	45.853737000	41.933489000
7	43.587092000	41.856913000	41.803863000
6	43.040011000	40.928295000	42.783527000
6	43.318570000	39.462086000	42.433558000
6	42.898305000	38.535586000	43.570174000
8	42.585978000	39.068499000	41.264998000
1	43.782225000	41.522235000	40.862685000
1	41.951900000	41.060777000	42.874533000
1	44.395232000	39.336324000	42.246339000
1	43.050389000	39.436788000	40.477562000
1	41.827663000	38.647750000	43.779750000
1	43.091691000	37.495233000	43.293366000
1	43.459648000	38.758144000	44.483920000
6	38.954757000	33.153466000	42.319521000
6	37.648983000	33.274097000	41.524216000
8	36.597983000	32.797227000	41.954853000
6	39.958785000	34.309800000	42.213469000
6	41.114423000	34.141150000	43.204375000
8	40.442640000	34.343450000	40.873964000
1	38.661746000	33.004156000	43.363058000
1	39.433115000	35.252844000	42.446272000
1	41.066921000	35.108957000	40.790707000
1	41.630038000	33.188460000	43.034135000
1	41.842616000	34.946980000	43.069416000
1	40.755922000	34.166378000	44.241332000
7	37.761714000	33.899966000	40.319951000
6	36.652138000	34.038737000	39.393316000
6	36.261881000	35.498669000	39.076645000
6	35.797595000	36.242979000	40.342649000
6	35.198449000	35.505476000	37.966800000
6	35.654656000	37.760998000	40.175289000
1	38.695852000	34.224341000	40.069399000
1	35.806410000	33.508986000	39.842928000
1	37.155510000	36.008693000	38.685330000
1	34.840577000	35.813767000	40.675901000
1	36.516004000	36.040785000	41.146459000
1	34.938691000	36.520309000	37.651330000
1	35.556156000	34.969377000	37.078721000
1	34.278042000	35.011198000	38.307952000
1	35.349947000	38.229445000	41.119371000
1	36.608182000	38.215732000	39.877960000
1	34.912784000	38.033619000	39.417076000
6	50.304299000	32.114085000	42.745948000
6	48.885632000	32.662731000	42.584154000
16	48.862023000	34.465412000	42.281889000
1	50.902164000	32.301077000	41.847550000
1	48.383496000	32.180675000	41.735888000
1	48.277302000	32.447684000	43.470210000
6	40.339396000	47.209568000	42.404067000
6	41.156378000	48.317511000	43.060789000
8	40.799864000	48.835139000	44.123743000

6	40.457482000	45.833657000	43.099759000
6	40.098198000	45.765091000	44.597287000
6	38.680170000	46.272975000	44.894930000
6	40.294098000	44.323954000	45.096079000
1	39.296611000	47.545678000	42.418476000
1	39.805091000	45.136419000	42.551568000
1	41.477558000	45.451757000	42.978775000
1	40.797771000	46.413798000	45.139604000
1	38.595098000	47.340879000	44.673032000
1	37.930403000	45.728089000	44.303073000
1	38.430198000	46.130428000	45.954617000
1	41.296944000	43.952776000	44.854887000
1	40.150136000	44.258084000	46.182573000
1	39.567338000	43.646207000	44.625768000
7	42.286214000	48.694473000	42.395694000
6	43.147775000	49.752161000	42.896379000
6	44.571863000	49.292113000	43.257377000
6	44.601292000	48.158217000	44.284118000
8	45.339311000	48.958680000	42.097064000
1	42.569342000	48.227019000	41.531086000
1	42.652189000	50.161167000	43.780796000
1	45.082998000	50.166444000	43.684313000
1	44.827468000	48.326856000	41.537595000
1	45.638277000	47.901559000	44.524259000
1	44.084726000	48.450471000	45.206206000
1	44.109242000	47.260063000	43.896824000
8	44.948370000	35.762396000	41.503245000
26	46.443458000	35.216629000	41.876610000
7	45.739405000	33.523981000	42.727831000
7	46.446055000	36.118862000	43.685218000
7	46.677252000	34.231457000	40.129051000
6	45.700631000	32.068407000	40.754201000
6	45.410159000	34.341278000	45.018661000
6	47.430608000	38.275038000	43.054027000
6	47.593349000	36.053628000	38.759194000
6	45.439269000	32.349019000	42.091105000
6	45.937890000	35.615408000	44.852600000
6	47.651662000	38.007258000	41.711285000
6	47.127872000	34.759648000	38.943066000
6	44.819602000	31.427120000	43.011463000
6	46.034975000	36.600662000	45.903803000
6	48.185606000	38.960524000	40.764723000
6	47.029975000	33.774205000	37.893124000
6	44.741570000	32.065926000	44.212387000
6	46.609596000	37.705021000	45.352314000
6	48.210789000	38.341550000	39.554021000
6	46.505772000	32.650600000	38.454067000
6	45.313068000	33.377325000	44.023614000
6	46.858987000	37.392019000	43.964696000
6	47.706719000	37.004024000	39.765049000
6	46.275044000	32.948969000	39.847622000
7	47.375331000	36.819235000	41.081951000
1	45.413514000	31.088556000	40.384411000
1	45.034472000	34.080888000	46.003728000
1	47.702722000	39.261013000	43.418120000
1	47.871392000	36.363239000	37.756822000
6	43.478735000	42.628797000	34.269680000
6	42.349584000	41.904447000	36.085563000
6	41.740781000	41.657956000	37.318501000
6	41.676859000	39.672448000	35.291003000
6	42.314218000	40.894285000	35.075313000
6	43.105263000	43.013387000	35.545724000
6	41.111264000	40.419168000	37.540165000
6	41.073422000	39.436109000	36.530506000
7	42.999377000	41.377999000	33.981348000
6	43.462895000	44.300798000	36.232129000
6	44.374510000	45.169402000	35.359941000
7	44.900367000	46.309065000	36.100261000
6	45.648961000	47.239723000	35.497844000
8	45.859828000	47.236846000	34.260766000
6	46.220365000	48.320962000	36.391665000
8	40.563217000	40.261145000	38.779229000
6	39.982473000	39.004413000	39.120315000
1	44.088222000	43.135548000	33.529866000
1	41.769428000	42.377613000	38.130690000
1	47.230681000	48.568520000	36.052997000
1	40.719699000	38.194694000	39.060501000
1	39.124453000	38.769526000	38.478687000
1	39.661380000	39.105687000	40.157736000
1	41.647449000	38.910793000	34.515917000
1	40.576438000	38.490058000	36.707827000
1	42.558741000	44.870452000	36.494707000
1	43.965908000	44.100345000	37.188539000
1	43.827364000	45.527780000	34.481181000
1	45.211429000	44.570030000	34.978126000
1	46.242749000	48.010757000	37.439698000
1	45.609225000	49.228196000	36.320603000
1	43.333273000	40.867947000	33.144589000
1	44.818732000	46.311032000	37.127724000
8	42.280282000	36.346707000	40.699947000
1	43.178753000	36.024408000	40.915772000
1	42.294111000	37.297063000	40.960886000
1	50.816068000	32.584515000	43.592905000

1	50.278917000	31.030297000	42.924031000
1	48.505543000	38.723781000	38.585734000
1	48.478695000	39.972804000	41.013135000
1	46.844900000	38.651914000	45.821692000
1	45.699669000	36.450409000	46.922416000
1	44.331631000	31.701384000	45.146020000
1	44.487477000	30.429150000	42.753903000
1	46.275921000	31.703446000	37.982132000
1	47.325158000	33.943993000	36.865497000
1	43.475572000	41.174622000	43.758002000
1	44.241255000	43.529986000	40.004810000
1	43.225400000	50.549111000	42.143892000
1	40.623109000	47.097228000	41.350554000
1	36.315188000	39.764455000	34.024677000
1	32.641469000	41.861907000	35.662809000
1	36.903144000	33.527133000	38.452129000
1	39.464587000	32.235936000	41.991173000
1	46.187532000	45.598888000	29.856385000
1	43.506847000	48.619211000	31.445797000
1	46.882426000	51.349699000	32.961396000
1	40.802851000	50.930937000	38.368336000
1	42.669386000	54.821978000	35.379562000
1	46.681784000	54.247677000	38.557221000
1	44.628123000	37.219782000	39.269819000
1	46.653083000	40.628998000	37.248533000
1	45.344055000	37.227775000	34.536697000
1	49.792107000	37.927291000	34.560862000

^{2}TS1$_{C6,II}$

6	42.168407000	41.265239000	32.135818000
6	42.989561000	41.091186000	30.862794000
8	42.670562000	41.623475000	29.794504000
6	40.663499000	41.005061000	31.914568000
6	39.861969000	41.480355000	33.142600000
6	40.405560000	39.530872000	31.568373000
6	38.339010000	41.434738000	32.967303000
1	42.549592000	40.618007000	32.937542000
1	40.359957000	41.616749000	31.055203000
1	40.147371000	40.877995000	34.019227000
1	40.161526000	42.511875000	33.367385000
1	39.349990000	39.345085000	31.341881000
1	40.987408000	39.224106000	30.691898000
1	40.687441000	38.876081000	32.405618000
1	37.967534000	40.412021000	32.831557000
1	37.833739000	41.853105000	33.845781000
1	38.027314000	42.020837000	32.093610000
7	44.095697000	40.307921000	30.970493000
6	45.076105000	40.188015000	29.898891000
6	46.202890000	39.263313000	30.347855000
8	46.778505000	39.808457000	31.513822000
1	44.431715000	40.058966000	31.892412000
1	45.484590000	41.175772000	29.645818000
1	45.798204000	38.254760000	30.534845000
1	46.936466000	39.176602000	29.528859000
1	47.233864000	39.094229000	32.040197000
6	41.630442000	42.707896000	40.413924000
6	41.521732000	43.378737000	41.779017000
8	40.538488000	43.147897000	42.518839000
6	41.023269000	41.299433000	40.440961000
8	39.640890000	41.331561000	40.831273000
1	41.092850000	43.329734000	39.685895000
1	41.074173000	40.842027000	39.449756000
1	41.594917000	40.662318000	41.130712000
1	39.632674000	42.011313000	41.552606000
7	42.510960000	44.217579000	42.130232000
6	42.476183000	44.990117000	43.368324000
6	43.668539000	44.696882000	44.302305000
6	43.661148000	43.308870000	44.909736000
6	42.768045000	42.991839000	45.944446000
6	44.538316000	42.312826000	44.457347000
6	42.745186000	41.718483000	46.513614000
6	44.520041000	41.037198000	45.030340000
6	43.625220000	40.733674000	46.057520000
1	43.189335000	44.505229000	41.422048000
1	41.533695000	44.748786000	43.864251000
1	44.602527000	44.870778000	43.756855000
1	43.631366000	45.447305000	45.105038000
1	42.082083000	43.754714000	46.308597000
1	45.243217000	42.528513000	43.655957000
1	42.043664000	41.496204000	47.314527000
1	45.213542000	40.280630000	44.670386000
1	43.612996000	39.740532000	46.500543000
6	43.056812000	51.984135000	36.159658000
6	41.779812000	52.782097000	36.441986000
8	41.822052000	53.892565000	36.963462000
6	43.482108000	51.075601000	37.331481000
6	42.738291000	49.738071000	37.385580000
7	43.357213000	48.759103000	38.050900000
8	41.619969000	49.585584000	36.850941000
1	42.952297000	51.386003000	35.247646000
1	44.559145000	50.878340000	37.280726000
1	43.317738000	51.601820000	38.281660000
1	44.288798000	48.857489000	38.467265000
1	42.915814000	47.845012000	38.153595000
7	40.617011000	52.166244000	36.062713000
6	39.317542000	52.678959000	36.452666000
6	38.579597000	51.930615000	37.580086000
8	37.444500000	52.287705000	37.888909000
1	40.705245000	51.180223000	35.837064000
1	39.465997000	53.709946000	36.783707000
7	39.259146000	50.914939000	38.169878000
6	38.684101000	50.068487000	39.203867000
6	38.582894000	48.599034000	38.742089000
6	38.096284000	47.677241000	39.839400000
6	36.734594000	47.624235000	40.170692000
6	38.992383000	46.871038000	40.559129000
6	36.275622000	46.789887000	41.191047000
6	38.535381000	46.025798000	41.576078000
6	37.175310000	45.985713000	41.895225000
1	40.150613000	50.621070000	37.779768000
1	37.698509000	50.476362000	39.440230000
1	37.903966000	48.555904000	37.880655000
1	39.567514000	48.274793000	38.385316000
1	36.027470000	48.238432000	39.616269000
1	40.053645000	46.896997000	40.317371000
1	35.214971000	46.761093000	41.430131000
1	39.231889000	45.377075000	42.100359000
1	36.819590000	45.324447000	42.681294000
6	40.159407000	46.457977000	30.431102000
6	40.628037000	45.227353000	29.652532000
7	41.401075000	44.370304000	30.363087000
8	40.314942000	45.064290000	28.474837000
1	40.470033000	46.449803000	31.479781000
1	39.067135000	46.514340000	30.377542000
1	41.743764000	43.512748000	29.933471000
1	41.617870000	44.540932000	31.341073000
6	52.303087000	41.489947000	34.018976000
6	51.433788000	40.844115000	35.102684000
8	51.248782000	41.417372000	36.188623000
6	51.869388000	41.256592000	32.559149000
6	50.464471000	41.780675000	32.188612000
6	50.401832000	43.315620000	32.192199000
6	50.018457000	41.205475000	30.836794000
1	52.337806000	42.557169000	34.257166000
1	52.612053000	41.734785000	31.902674000
1	51.917943000	40.185360000	32.325784000
1	49.755196000	41.409258000	32.940499000
1	50.663253000	43.741283000	33.168452000
1	51.091740000	43.737253000	31.447466000
1	49.392579000	43.663564000	31.942961000
1	50.702462000	41.511758000	30.031770000
1	49.987472000	40.112613000	30.885997000
1	49.010007000	41.548979000	30.582047000
7	50.941447000	39.622281000	34.824619000
6	49.960179000	38.952579000	35.673879000
6	49.439370000	37.688783000	34.962262000
6	48.958969000	38.015705000	33.528316000
8	49.852539000	38.251654000	32.682522000
8	47.698079000	38.065331000	33.332090000
1	50.958957000	39.260429000	33.867158000
1	49.130789000	39.638048000	35.890305000
1	50.252854000	36.955955000	34.893913000
1	48.630603000	37.255900000	35.558361000
6	49.747890000	44.398772000	36.818449000
6	49.467250000	43.892274000	38.223294000
8	48.953692000	44.626191000	39.087059000
1	50.219939000	45.383930000	36.884860000
1	48.796566000	44.521686000	36.287370000
7	49.790920000	42.609829000	38.462260000
6	49.552951000	41.967465000	39.753289000
6	49.409893000	40.456184000	39.587211000
1	50.246142000	42.074800000	37.722329000
1	48.651876000	42.398266000	40.199856000
1	50.301368000	40.017801000	39.121250000
1	49.271342000	39.985106000	40.566473000
1	48.543604000	40.209805000	38.963989000
6	46.280337000	46.718903000	37.765406000
6	46.590275000	47.542536000	39.010195000
8	45.939675000	48.553193000	39.322948000
6	45.593768000	45.381661000	38.148113000
6	44.275183000	45.626906000	38.895255000
8	44.349022000	45.692769000	40.175113000
8	43.229783000	45.805832000	38.225547000
1	45.631353000	47.303262000	37.108450000
1	45.387088000	44.832167000	37.228099000
1	46.276180000	44.784109000	38.758754000
7	47.588760000	47.031983000	39.763233000
6	47.804006000	47.355253000	41.163653000
6	47.661209000	46.093194000	42.031314000
6	48.083218000	46.351216000	43.474545000
8	46.317461000	45.602251000	42.068573000
1	48.086240000	46.209617000	39.406026000
1	47.075713000	48.117781000	41.452548000
1	48.309772000	45.317616000	41.596820000
1	45.822595000	45.735300000	41.220644000
1	47.480221000	47.153178000	43.916084000

1	47.936244000	45.446386000	44.072534000
1	49.141317000	46.633323000	43.528467000
6	37.093802000	39.004157000	44.096871000
6	35.793543000	39.002195000	43.281503000
8	34.689875000	39.090889000	43.817539000
6	38.218555000	39.885510000	43.528994000
6	39.348135000	40.116482000	44.533332000
8	38.719481000	39.250249000	42.342496000
1	36.823514000	39.336409000	45.102891000
1	37.789170000	40.862997000	43.258164000
1	39.121874000	39.952248000	41.767288000
1	39.809674000	39.163706000	44.819117000
1	40.119003000	40.762374000	44.100582000
1	38.971768000	40.606196000	45.439824000
7	35.980151000	38.885844000	41.936996000
6	34.918116000	39.023712000	40.956749000
6	35.263688000	40.062104000	39.869104000
6	35.367962000	41.489950000	40.444358000
6	34.247615000	39.976454000	38.720835000
6	36.198846000	42.452651000	39.586435000
1	36.951256000	38.881980000	41.629212000
1	34.013054000	39.308846000	41.503151000
1	36.252743000	39.790712000	39.464273000
1	34.351698000	41.887084000	40.587316000
1	35.817732000	41.437976000	41.443410000
1	34.478696000	40.694173000	37.926556000
1	34.233276000	38.975803000	38.271204000
1	33.233942000	40.195257000	39.082551000
1	36.186958000	43.464801000	40.006499000
1	37.245460000	42.127876000	39.545393000
1	35.821752000	42.519234000	38.558333000
6	40.724171000	32.583938000	39.265052000
6	41.346615000	33.968149000	39.469466000
16	42.645958000	34.294745000	38.218097000
1	40.262644000	32.503806000	38.274802000
1	40.574590000	34.739819000	39.378206000
1	41.773839000	34.051226000	40.474348000
6	39.422268000	49.323751000	44.330695000
6	40.589336000	50.032246000	43.645590000
8	41.080368000	51.065981000	44.103144000
6	39.869807000	48.430159000	45.509305000
6	40.660324000	49.122830000	46.638068000
6	39.893431000	50.300963000	47.256631000
6	41.048337000	48.092216000	47.709403000
1	38.739280000	50.102045000	44.685021000
1	38.969584000	47.965934000	45.939482000
1	40.475975000	47.604132000	45.111665000
1	41.582806000	49.528593000	46.203427000
1	39.762669000	51.104457000	46.526284000
1	38.903723000	49.987022000	47.618257000
1	40.442537000	50.714494000	48.112136000
1	41.619352000	47.260117000	47.278829000
1	41.663792000	48.548903000	48.494506000
1	40.156693000	47.667863000	48.191366000
7	41.047549000	49.418526000	42.519694000
6	42.211395000	49.854874000	41.763837000
6	43.260163000	48.739889000	41.647045000
6	43.998638000	48.476527000	42.962589000
8	42.554297000	47.587214000	41.189892000
1	40.634896000	48.541867000	42.227661000
1	42.615501000	50.734690000	42.269547000
1	43.996548000	49.052951000	40.895038000
1	43.189080000	46.926967000	40.820330000
1	44.684210000	47.629805000	42.849467000
1	44.583086000	49.356561000	43.261011000
1	43.292882000	48.248619000	43.769631000
8	43.846527000	38.256232000	38.541299000
26	43.510063000	36.619207000	38.495555000
7	42.876682000	36.664697000	40.417536000
7	45.295865000	35.901051000	39.095894000
7	41.653899000	37.169152000	37.891509000
6	40.589324000	37.497234000	40.074324000
6	44.947098000	35.966392000	41.527700000
6	46.312815000	35.428257000	36.912257000
6	42.049427000	37.208472000	35.474615000
6	41.661876000	37.109539000	40.870437000
6	45.705285000	35.707969000	40.392565000
6	45.270492000	35.881621000	36.114310000
6	41.265900000	37.413662000	36.601666000
6	41.641899000	37.133396000	42.313719000
6	47.035360000	35.146853000	40.416357000
6	45.313451000	35.956339000	34.671997000
6	39.914500000	37.928406000	36.577055000
6	42.865535000	36.699649000	42.726232000
6	47.412922000	34.976839000	39.119283000
6	44.118974000	36.472543000	34.276121000
6	39.498619000	37.998575000	37.869937000
6	43.631477000	36.415425000	41.534474000
6	46.317869000	35.444088000	38.303306000
6	43.344192000	36.707357000	35.472059000
6	40.598584000	37.532819000	38.686100000
7	44.069457000	36.353726000	36.580920000
1	39.691344000	37.824049000	40.588462000
1	45.416144000	35.785537000	42.490703000
1	47.199302000	35.051757000	36.410844000
1	41.608966000	37.448918000	34.511357000
6	45.793325000	41.047295000	34.307273000
6	45.307182000	41.211915000	36.498700000
6	44.954086000	41.532453000	37.829080000
6	46.125349000	39.007159000	37.232471000
6	45.916825000	39.939964000	36.235456000
6	45.218728000	41.896972000	35.248582000
6	45.152644000	40.600491000	38.829179000
6	45.669638000	39.293646000	38.538379000
7	46.206617000	39.882789000	34.883715000
6	44.641006000	43.257007000	34.974478000
6	43.193883000	43.424296000	35.492628000
7	42.727042000	44.803136000	35.461254000
6	42.149563000	45.344229000	34.368236000
8	41.992039000	44.711613000	33.310564000
6	41.688047000	46.785746000	34.497011000
8	44.820779000	40.998772000	40.102475000
6	44.819620000	40.024743000	41.150095000
1	45.961175000	41.200783000	33.248854000
1	44.543195000	42.500583000	38.093684000
1	42.219869000	47.394673000	33.757667000
1	45.842344000	39.710381000	41.393983000
1	44.215874000	39.159196000	40.868891000
1	44.389461000	40.526888000	42.017398000
1	46.575152000	38.044979000	37.024907000
1	45.973311000	38.641934000	39.344656000
1	45.262499000	44.036732000	35.435346000
1	44.651522000	43.459492000	33.897446000
1	43.118680000	43.087619000	36.528669000
1	42.517289000	42.808802000	34.894969000
1	41.836765000	47.220016000	35.488710000
1	40.622705000	46.834524000	34.248630000
1	46.693941000	39.118729000	34.388235000
1	42.843641000	45.342680000	36.323058000
8	46.629854000	42.746360000	41.736712000
1	45.995903000	42.456142000	41.057794000
1	46.468483000	43.707022000	41.850954000
1	41.479200000	31.793640000	39.348325000
1	39.951068000	32.395768000	40.022693000
1	43.781353000	36.693286000	33.271369000
1	46.171887000	35.707097000	34.063087000
1	48.340298000	34.573359000	38.731992000
1	47.586798000	34.908039000	41.317414000
1	43.236756000	36.589124000	43.737691000
1	40.794881000	37.469183000	42.897362000
1	38.549220000	38.345536000	38.257963000
1	39.376737000	38.202119000	35.677964000
1	48.810708000	47.773038000	41.305767000
1	47.207790000	46.495426000	37.224739000
1	41.895695000	50.146752000	40.753684000
1	38.869658000	48.704464000	43.613795000
1	42.674557000	42.645378000	40.091554000
1	42.469098000	46.050003000	43.097682000
1	34.718107000	38.052110000	40.479247000
1	37.473396000	37.975638000	44.178093000
1	44.587340000	39.797471000	28.999285000
1	42.304686000	42.302159000	32.470359000
1	40.554131000	47.357208000	29.945286000
1	39.297542000	50.126253000	40.112586000
1	38.635861000	52.701603000	35.594456000
1	43.840755000	52.727894000	35.992763000
1	50.385583000	42.193950000	40.436379000
1	50.383971000	43.716589000	36.249462000
1	50.416052000	38.691386000	36.636845000
1	53.327151000	41.112872000	34.156883000

^{4}TS1$_{C6,II}$

6	42.107216000	41.297412000	31.868508000
6	43.020041000	41.228352000	30.649138000
8	42.791428000	41.863135000	29.614335000
6	40.615503000	41.126514000	31.511697000
6	39.745568000	41.463916000	32.738759000
6	40.338445000	39.723500000	30.950763000
6	38.237044000	41.495675000	32.463803000
1	42.402236000	40.550026000	32.618171000
1	40.391456000	41.859593000	30.726292000
1	39.956746000	40.741264000	33.542252000
1	40.057515000	42.445528000	33.117493000
1	39.294884000	39.611620000	30.636582000
1	40.966550000	39.519039000	30.076512000
1	40.544690000	38.950438000	31.704459000
1	37.847430000	40.512806000	32.173385000
1	37.685686000	41.816320000	33.355748000
1	38.000426000	42.199797000	31.656237000
7	44.105653000	40.417649000	30.766352000
6	45.167166000	40.382512000	29.768496000
6	46.244118000	39.400937000	30.220285000
8	46.687958000	39.811918000	31.493303000
1	44.372760000	40.085429000	31.684702000
1	45.603753000	41.382755000	29.643313000
1	45.823710000	38.381478000	30.250155000

1	47.059797000	39.400474000	29.478843000
1	47.179115000	39.080456000	31.960308000
6	41.535906000	42.642832000	40.575431000
6	41.559761000	43.303407000	41.950059000
8	40.617574000	43.122647000	42.754154000
6	40.816836000	41.288103000	40.626080000
8	39.466253000	41.426196000	41.091817000
1	41.014662000	43.316679000	39.882612000
1	40.776231000	40.841069000	39.629464000
1	41.373578000	40.597169000	41.275128000
1	39.553531000	42.089764000	41.821614000
7	42.618688000	44.078347000	42.240439000
6	42.709669000	44.835749000	43.485621000
6	43.949692000	44.473297000	44.326785000
6	43.931059000	43.070378000	44.899056000
6	43.061604000	42.742004000	45.950331000
6	44.778513000	42.071915000	44.398563000
6	43.034405000	41.455937000	46.489638000
6	44.756376000	40.783123000	44.941655000
6	43.886123000	40.469042000	45.986536000
1	43.258224000	44.342839000	41.488469000
1	41.793678000	44.632613000	44.043978000
1	44.850375000	44.627646000	43.723003000
1	43.997181000	45.202644000	45.148339000
1	42.397119000	43.505424000	46.350915000
1	45.460606000	42.297511000	43.580267000
1	42.350805000	41.224944000	47.303415000
1	45.427041000	40.024521000	44.544456000
1	43.870423000	39.465943000	46.406391000
6	42.837813000	51.722732000	36.182132000
6	41.570614000	52.533315000	36.474854000
8	41.630604000	53.673522000	36.925728000
6	43.289738000	50.837870000	37.363866000
6	42.561837000	49.493910000	37.454108000
7	43.223732000	48.518955000	38.083134000
8	41.417611000	49.331630000	36.980260000
1	42.707805000	51.104591000	35.286668000
1	44.367982000	50.652102000	37.300992000
1	43.132896000	51.378121000	38.307558000
1	44.172368000	48.628570000	38.456709000
1	42.794915000	47.602300000	38.209638000
7	40.395543000	51.890476000	36.190738000
6	39.113418000	52.417143000	36.617522000
6	38.434478000	51.717052000	37.810966000
8	37.315897000	52.086150000	38.162532000
1	40.481207000	50.893672000	36.013212000
1	39.271981000	53.462497000	36.894489000
7	39.145340000	50.726545000	38.407587000
6	38.621793000	49.918272000	39.497549000
6	38.471764000	48.438098000	39.085123000
6	38.048130000	47.554318000	40.238101000
6	36.710750000	47.528754000	40.659343000
6	38.980101000	46.757355000	40.921233000
6	36.310511000	46.730173000	41.731779000
6	38.582060000	45.947151000	41.990169000
6	37.245542000	45.934471000	42.398668000
1	40.012985000	50.413820000	37.980988000
1	37.657966000	50.347846000	39.780381000
1	37.736647000	48.380875000	38.272075000
1	39.426510000	48.090374000	38.673248000
1	35.975699000	48.136292000	40.134741000
1	40.023440000	46.762854000	40.609946000
1	35.267781000	46.722500000	42.040914000
1	39.304837000	45.303729000	42.484729000
1	36.934683000	45.300069000	43.224892000
6	40.273588000	46.655263000	30.477272000
6	40.799781000	45.494460000	29.631299000
7	41.513110000	44.570516000	30.320302000
8	40.580465000	45.440040000	28.422849000
1	40.490716000	46.546413000	31.543721000
1	39.190686000	46.732464000	30.334729000
1	41.881819000	43.749091000	29.844098000
1	41.650900000	44.647744000	31.324370000
6	52.282234000	41.780216000	33.646883000
6	51.525101000	41.068236000	34.772482000
8	51.419891000	41.590655000	35.894901000
6	51.714992000	41.606092000	32.224655000
6	50.275129000	42.122274000	32.014145000
6	50.197602000	43.655033000	32.076877000
6	49.705363000	41.586416000	30.692535000
1	52.326400000	42.835748000	33.931648000
1	52.385174000	42.125964000	31.523513000
1	51.754309000	40.547666000	31.938108000
1	49.647740000	41.714834000	32.818623000
1	50.556863000	44.051461000	33.034161000
1	50.801081000	44.109599000	31.278612000
1	49.164537000	43.998040000	31.946253000
1	50.314863000	41.915283000	29.838210000
1	49.675347000	40.492438000	30.711979000
1	48.680517000	41.941242000	30.537376000
7	51.041760000	39.846230000	34.483690000
6	50.173654000	39.101590000	35.390098000
6	49.672234000	37.823817000	34.691568000
6	49.038644000	38.146111000	33.317941000
8	49.823549000	38.502140000	32.408525000
8	47.766078000	38.064716000	33.225258000
1	50.980678000	39.522031000	33.514199000
1	49.325451000	39.730398000	35.691830000
1	50.523720000	37.151722000	34.526480000
1	48.954552000	37.320312000	35.344592000
6	49.860186000	44.477802000	36.660203000
6	49.545215000	43.898647000	38.028980000
8	48.985485000	44.579062000	38.907266000
1	50.313019000	45.465609000	36.790559000
1	48.923591000	44.613929000	36.106523000
7	49.892588000	42.613951000	38.221485000
6	49.632793000	41.913565000	39.477918000
6	49.500681000	40.409971000	39.246502000
1	50.384598000	42.123980000	37.473518000
1	48.721336000	42.321873000	39.924249000
1	50.406028000	39.993173000	38.787571000
1	49.338862000	39.899422000	40.202176000
1	48.652366000	40.185610000	38.590657000
6	46.199551000	46.547737000	37.697897000
6	46.531245000	47.401291000	38.916404000
8	45.859790000	48.394698000	39.240330000
6	45.562586000	45.201136000	38.131020000
6	44.259347000	45.426727000	38.911404000
8	44.371401000	45.528831000	40.186536000
8	43.188805000	45.554043000	38.270529000
1	45.512380000	47.102221000	37.053865000
1	45.346179000	44.623822000	37.230562000
1	46.279093000	44.636916000	38.734520000
7	47.573156000	46.933656000	39.637559000
6	47.824741000	47.270321000	41.028573000
6	47.749359000	46.006947000	41.903832000
6	48.214425000	46.282769000	43.330649000
8	46.423822000	45.474526000	41.987990000
1	48.087517000	46.127372000	39.267911000
1	47.084300000	48.011988000	41.339863000
1	48.406436000	45.250405000	41.448683000
1	45.887443000	45.606744000	41.165240000
1	47.604838000	47.068945000	43.791007000
1	48.115220000	45.376718000	43.936610000
1	49.265182000	46.595598000	43.345820000
6	36.845603000	39.354800000	44.503283000
6	35.518480000	39.402732000	43.733704000
8	34.444382000	39.599416000	44.300595000
6	38.001308000	40.141472000	43.861988000
6	39.189124000	40.309619000	44.810923000
8	38.400867000	39.441771000	42.676260000
1	36.634143000	39.741344000	45.503946000
1	37.628783000	41.140998000	43.586066000
1	38.847549000	40.085355000	42.069390000
1	39.596509000	39.329771000	45.087495000
1	39.981297000	40.895555000	44.333347000
1	38.889296000	40.833930000	45.726763000
7	35.647205000	39.198777000	42.392822000
6	34.564494000	39.367134000	41.440620000
6	34.935131000	40.347287000	40.307737000
6	35.133805000	41.784973000	40.830800000
6	33.883081000	40.280490000	39.190959000
6	35.987797000	42.673869000	39.917764000
1	36.605897000	39.120546000	42.055170000
1	33.695114000	39.723642000	42.002767000
1	35.895256000	40.007394000	39.886439000
1	34.145413000	42.241577000	40.990785000
1	35.611239000	41.740734000	41.817238000
1	34.126648000	40.962376000	38.369238000
1	33.805664000	39.269242000	38.772395000
1	32.892286000	40.560614000	39.573192000
1	36.038254000	43.698263000	40.303417000
1	37.015453000	42.295221000	39.860624000
1	35.585724000	42.725139000	38.898165000
6	41.451973000	32.171448000	39.377166000
6	41.881442000	33.635190000	39.510755000
16	42.749457000	34.197311000	37.992477000
1	40.777659000	32.033995000	38.524393000
1	41.007337000	34.271051000	39.685960000
1	42.550561000	33.751169000	40.369687000
6	39.624217000	49.305716000	44.530731000
6	40.757431000	49.978094000	43.758468000
8	41.242194000	51.051631000	44.122301000
6	40.096117000	48.656999000	45.851367000
6	40.757945000	49.590643000	46.885643000
6	39.849892000	50.762208000	47.286994000
6	41.184763000	48.778606000	48.118210000
1	38.876086000	50.077854000	44.738769000
1	39.223116000	48.175523000	46.317430000
1	40.797171000	47.845377000	45.610223000
1	41.656227000	50.019027000	46.424896000
1	39.680182000	51.431202000	46.438646000
1	38.877106000	50.406427000	47.655725000
1	40.311547000	51.353141000	48.088273000
1	41.857204000	47.955909000	47.844984000
1	41.707879000	49.410105000	48.847251000

1	40.313128000	48.340151000	48.623966000
7	41.192602000	49.292635000	42.665688000
6	42.310545000	49.705041000	41.831365000
6	43.351688000	48.585784000	41.693706000
6	44.153283000	48.358540000	42.978906000
8	42.624815000	47.420018000	41.305868000
1	40.777872000	48.398500000	42.437099000
1	42.740307000	50.601235000	42.284578000
1	44.049574000	48.875830000	40.897731000
1	43.236737000	46.762223000	40.896372000
1	44.830522000	47.506357000	42.856872000
1	44.753323000	49.244969000	43.222758000
1	43.486727000	48.156774000	43.825309000
8	44.137627000	38.180885000	38.487536000
26	43.464508000	36.478486000	38.343662000
7	41.841550000	36.954298000	39.392823000
7	44.391827000	35.884189000	40.047787000
7	42.525054000	36.973840000	36.626317000
6	40.361498000	37.642787000	37.553842000
6	42.669789000	36.526084000	41.668332000
6	46.503943000	35.067796000	39.090256000
6	44.330966000	36.538493000	35.021196000
6	40.654803000	37.433755000	38.893598000
6	43.917287000	36.011831000	41.324917000
6	46.242574000	35.337185000	37.751608000
6	43.055679000	36.961668000	35.362460000
6	39.751572000	37.736995000	39.976999000
6	44.896010000	35.523165000	42.267269000
6	47.183941000	35.127123000	36.675230000
6	42.075633000	37.435706000	34.410524000
6	40.405896000	37.448916000	41.135358000
6	45.965262000	35.097902000	41.537210000
6	46.581718000	35.564201000	35.532367000
6	40.950316000	37.725495000	35.117242000
6	41.709970000	36.954589000	40.763496000
6	45.644848000	35.333105000	40.149218000
6	45.267248000	36.026182000	35.912654000
6	41.246560000	37.439352000	36.503602000
7	45.086910000	35.882922000	37.260364000
1	39.375469000	38.027816000	37.312797000
1	42.420443000	36.580565000	42.723643000
1	47.470585000	34.634807000	39.330968000
1	44.622249000	36.601765000	33.977608000
6	45.823643000	40.964589000	34.285225000
6	45.288747000	41.094103000	36.465787000
6	44.895716000	41.398580000	37.792193000
6	46.260318000	38.952110000	37.206202000
6	45.991004000	39.859913000	36.213418000
6	45.180451000	41.774357000	35.221955000
6	45.121176000	40.482955000	38.792641000
6	45.687671000	39.167204000	38.509276000
7	46.311475000	39.833265000	34.864998000
6	44.545280000	43.108161000	34.943754000
6	43.089701000	43.225727000	35.453217000
7	42.606496000	44.598911000	35.484011000
6	42.043594000	45.190023000	34.409092000
8	41.899341000	44.605725000	33.322001000
6	41.580161000	46.623878000	34.597267000
8	44.765901000	40.857718000	40.058988000
6	44.790651000	39.881139000	41.108242000
1	45.981477000	41.123422000	33.226019000
1	44.436114000	42.346850000	38.047493000
1	42.134525000	47.268979000	33.906664000
1	45.823180000	39.644317000	41.393170000
1	44.262015000	38.977730000	40.797821000
1	44.292180000	40.352087000	41.956251000
1	46.804363000	38.035383000	37.015568000
1	46.149711000	38.635930000	39.332547000
1	45.133082000	43.910742000	35.409005000
1	44.557228000	43.312932000	33.867230000
1	43.007790000	42.836648000	36.470078000
1	42.427067000	42.632778000	34.818324000
1	41.699359000	47.006514000	35.613617000
1	40.522875000	46.687882000	34.320081000
1	46.810979000	39.083782000	34.358960000
1	42.738093000	45.108152000	36.361958000
8	46.708957000	42.620628000	41.559109000
1	45.995746000	42.355099000	40.953546000
1	46.572146000	43.578787000	41.717589000
1	42.319362000	31.518945000	39.226977000
1	40.926012000	31.841357000	40.283990000
1	46.981136000	35.622854000	34.528161000
1	48.176857000	34.711971000	36.797536000
1	46.895298000	34.669049000	41.889785000
1	44.764959000	35.513621000	43.342352000
1	40.045917000	37.590311000	42.145652000
1	38.755029000	38.141398000	39.855365000
1	40.002965000	38.104664000	34.755036000
1	42.245659000	37.521713000	33.344482000
1	48.822482000	47.718630000	41.135928000
1	47.112819000	46.336942000	37.128884000
1	41.939931000	49.963858000	40.830613000
1	39.135293000	48.538987000	43.918081000
1	42.552880000	42.501219000	40.194545000
1	42.731717000	45.898264000	43.227527000
1	34.294062000	38.393731000	41.003126000
1	37.165311000	38.308510000	44.611228000
1	44.749393000	40.085435000	28.800236000
1	42.249912000	42.285230000	32.326939000
1	40.715484000	47.589546000	30.113551000
1	39.289966000	49.991424000	40.365513000
1	38.391444000	52.399305000	35.792703000
1	43.619896000	52.458869000	35.978372000
1	50.450204000	42.113811000	40.186994000
1	50.525159000	43.834695000	36.079060000
1	50.722469000	38.849070000	36.305666000
1	53.318965000	41.413654000	33.669580000

^{2}IM1$_{C6,II}$

6	41.464714000	42.393825000	31.361866000
6	42.507762000	42.265228000	30.256897000
8	42.528733000	43.005010000	29.268137000
6	40.041033000	42.662874000	30.837251000
6	39.122588000	43.063496000	32.009063000
6	39.508309000	41.460385000	30.043473000
6	37.722969000	43.531169000	31.592066000
1	41.467773000	41.495418000	31.995727000
1	40.110139000	43.519409000	30.155266000
1	39.031908000	42.216409000	32.706502000
1	39.614907000	43.870336000	32.567260000
1	38.523890000	41.666149000	29.608589000
1	40.183779000	41.208093000	29.218522000
1	39.411488000	40.574461000	30.686847000
1	37.143796000	42.730242000	31.117735000
1	37.152345000	43.877621000	32.461956000
1	37.784362000	44.364320000	30.880767000
7	43.436599000	41.288670000	30.437549000
6	44.641875000	41.202042000	29.624127000
6	45.497572000	40.046664000	30.131762000
8	45.714244000	40.252047000	31.511727000
1	43.512186000	40.847009000	31.345436000
1	45.207283000	42.142281000	29.684054000
1	44.975459000	39.092423000	29.948919000
1	46.442338000	40.023215000	29.567373000
1	46.131558000	39.448812000	31.931711000
6	40.665775000	41.514049000	40.576441000
6	40.950334000	42.443883000	41.749016000
8	40.050583000	42.706583000	42.573703000
6	39.853859000	40.296030000	41.027388000
8	38.595202000	40.682888000	41.598108000
1	40.084638000	42.085505000	39.840511000
1	39.639927000	39.648531000	40.172554000
1	40.435585000	39.709557000	41.750419000
1	38.816915000	41.476150000	42.145865000
7	42.189492000	42.966151000	41.824283000
6	42.595416000	43.978236000	42.803327000
6	43.819331000	43.566679000	43.629671000
6	43.678886000	42.358021000	44.539779000
6	42.463689000	41.981791000	45.130788000
6	44.826019000	41.607264000	44.845591000
6	42.403451000	40.902591000	46.016515000
6	44.768560000	40.532407000	45.733777000
6	43.555119000	40.177671000	46.328844000
1	42.867292000	42.679442000	41.126535000
1	41.721888000	44.165316000	43.430414000
1	44.675361000	43.408319000	42.962032000
1	44.088410000	44.442594000	44.238826000
1	41.550007000	42.515225000	44.885329000
1	45.765673000	41.867056000	44.363146000
1	41.450020000	40.628825000	46.462311000
1	45.672057000	39.969479000	45.957202000
1	43.506588000	39.342045000	47.023145000
6	43.819505000	52.966486000	36.549105000
6	42.426405000	53.557809000	36.792059000
8	42.263986000	54.526703000	37.527627000
6	44.217341000	51.897516000	37.586530000
6	43.620103000	50.514398000	37.314340000
7	44.189702000	49.490532000	37.960360000
8	42.644929000	50.361191000	36.550652000
1	43.898952000	52.547993000	35.539847000
1	45.309538000	51.805775000	37.627996000
1	43.904783000	52.221341000	38.588299000
1	45.049781000	49.564859000	38.509611000
1	43.849675000	48.538892000	37.794740000
7	41.406955000	52.931373000	36.125015000
6	40.013698000	53.210747000	36.419316000
6	39.253247000	52.170672000	37.267323000
8	38.062126000	52.354284000	37.508837000
1	41.648011000	52.032515000	35.719819000
1	39.983780000	54.159232000	36.961120000
7	39.977494000	51.107999000	37.696908000
6	39.426447000	50.021628000	38.491026000
6	39.460713000	48.676029000	37.730081000
6	39.178956000	47.502024000	38.642281000
6	37.867711000	47.165296000	39.009329000
6	40.236681000	46.760842000	39.189785000

6	37.617018000	46.121987000	39.903742000
6	39.988105000	45.721504000	40.090062000
6	38.678096000	45.397170000	40.453790000
1	40.937581000	50.997707000	37.382900000
1	38.403784000	50.299433000	38.756201000
1	38.735151000	48.719030000	36.907722000
1	40.453406000	48.562625000	37.278467000
1	37.035302000	47.726592000	38.588583000
1	41.265133000	46.985727000	38.916270000
1	36.592145000	45.875028000	40.171500000
1	40.827777000	45.173134000	40.505125000
1	38.496692000	44.587395000	41.155578000
6	40.995166000	48.118382000	30.405242000
6	41.336683000	46.943950000	29.486981000
7	41.824032000	45.848312000	30.118815000
8	41.185253000	47.028863000	28.269798000
1	40.997379000	47.854905000	31.466842000
1	40.009792000	48.505213000	30.128578000
1	42.050997000	45.012651000	29.582848000
1	41.912728000	45.799123000	31.131503000
6	51.771560000	41.357077000	33.338817000
6	50.956452000	40.750414000	34.485219000
8	50.998730000	41.244721000	35.624370000
6	51.112118000	41.334160000	31.947152000
6	49.783488000	42.113937000	31.833757000
6	50.001859000	43.633238000	31.894984000
6	49.032416000	41.705143000	30.558510000
1	52.015337000	42.378529000	33.645659000
1	51.828932000	41.745082000	31.220639000
1	50.941861000	40.295897000	31.635703000
1	49.149173000	41.825785000	32.683603000
1	50.503976000	43.944362000	32.818976000
1	50.615605000	43.973859000	31.049517000
1	49.045526000	44.167033000	31.844954000
1	49.641261000	41.895925000	29.663205000
1	48.778888000	40.640862000	30.597704000
1	48.098223000	42.269689000	30.456914000
7	50.252725000	39.641166000	34.193033000
6	49.332062000	39.013486000	35.134714000
6	48.608108000	37.841506000	34.447541000
6	47.932732000	38.285671000	33.132893000
8	48.687763000	38.592093000	32.181759000
8	46.654809000	38.338439000	33.117024000
1	50.080015000	39.367523000	33.221536000
1	48.605831000	39.754790000	35.495256000
1	49.344319000	37.064689000	34.205308000
1	47.878302000	37.414437000	35.139733000
6	50.109976000	44.407134000	36.403524000
6	49.654658000	43.896619000	37.760314000
8	49.230400000	44.675631000	38.631805000
1	50.779581000	45.259529000	36.555399000
1	49.236958000	44.770620000	35.848727000
7	49.722467000	42.565309000	37.945102000
6	49.307716000	41.919487000	39.189506000
6	48.884145000	40.474012000	38.940446000
1	50.118021000	41.989605000	37.201049000
1	48.486101000	42.491386000	39.629100000
1	49.697081000	39.887329000	38.495214000
1	48.598576000	39.998155000	39.884282000
1	48.024686000	40.428460000	38.262240000
6	47.043489000	47.545344000	37.417980000
6	47.289778000	47.972420000	38.863180000
8	46.684813000	48.925273000	39.379741000
6	46.280281000	46.198651000	37.321440000
6	44.982334000	46.218947000	38.147876000
8	45.081553000	45.878273000	39.365698000
8	43.919236000	46.612241000	37.589586000
1	46.471718000	48.336970000	36.928128000
1	46.043381000	46.022026000	36.267397000
1	46.926443000	45.392327000	37.680303000
7	48.194467000	47.214637000	39.523918000
6	48.384691000	47.254658000	40.966155000
6	48.109534000	45.880499000	41.598504000
6	48.481527000	45.860877000	43.077799000
8	46.729588000	45.523191000	41.509772000
1	48.612394000	46.418280000	39.036597000
1	47.708246000	48.010981000	41.372392000
1	48.714199000	45.135930000	41.060329000
1	46.358625000	45.706755000	40.610372000
1	47.914217000	46.620305000	43.628325000
1	48.247601000	44.881905000	43.508136000
1	49.552432000	46.051544000	43.214899000
6	36.103500000	38.242018000	44.910647000
6	34.787570000	38.322699000	44.125563000
8	33.700275000	38.438317000	44.689049000
6	37.238278000	39.134368000	44.379735000
6	38.411902000	39.215415000	45.358264000
8	37.674640000	38.602159000	43.123427000
1	35.863267000	38.510171000	45.943110000
1	36.838926000	40.149070000	44.223563000
1	38.027152000	39.352773000	42.581183000
1	38.814531000	38.214530000	45.553452000
1	39.215097000	39.829469000	44.935798000
1	38.103570000	39.661028000	46.312131000
7	34.941132000	38.237663000	42.774393000
6	33.859691000	38.426017000	41.824683000
6	34.191834000	39.498256000	40.765890000
6	34.321012000	40.903171000	41.389662000
6	33.148958000	39.460979000	39.639257000
6	35.130699000	41.892112000	40.541445000
1	35.905383000	38.219303000	42.444986000
1	32.971432000	38.702035000	42.402351000
1	35.168562000	39.232471000	40.330369000
1	33.312486000	41.300489000	41.578405000
1	34.801474000	40.812933000	42.371452000
1	33.368134000	40.202165000	38.863253000
1	33.115512000	38.475950000	39.157412000
1	32.145548000	39.675753000	40.030709000
1	35.130561000	42.889663000	40.996780000
1	36.174728000	41.567068000	40.459662000
1	34.725302000	41.996414000	39.527555000
6	42.728898000	32.050800000	41.311005000
6	42.649396000	33.529929000	40.924437000
16	44.315311000	34.172269000	40.487995000
1	43.120972000	31.448720000	40.483249000
1	41.977158000	33.656896000	40.069700000
1	42.238166000	34.111538000	41.756023000
6	39.415784000	48.588031000	43.427797000
6	40.736667000	49.264796000	43.065785000
8	41.140903000	50.259243000	43.673816000
6	39.498132000	47.750351000	44.723595000
6	39.927754000	48.493610000	46.005227000
6	39.029130000	49.699400000	46.316322000
6	39.963070000	47.512009000	47.186776000
1	38.671164000	49.382766000	43.542605000
1	38.509140000	47.297928000	44.891955000
1	40.188988000	46.912299000	44.554719000
1	40.942371000	48.878574000	45.845805000
1	39.138016000	50.470155000	45.548242000
1	37.971190000	49.405692000	46.375633000
1	39.302458000	50.147863000	47.280079000
1	40.624558000	46.660656000	46.983469000
1	40.321440000	48.003980000	48.099869000
1	38.962105000	47.110314000	47.397264000
7	41.422077000	48.678866000	42.047860000
6	42.732856000	49.108570000	41.587404000
6	43.753758000	47.959400000	41.643842000
6	44.202349000	47.624605000	43.067977000
8	43.152351000	46.818196000	41.029867000
1	41.058323000	47.841034000	41.610685000
1	43.036635000	49.951198000	42.213327000
1	44.631886000	48.271922000	41.062562000
1	43.790839000	46.437286000	40.374153000
1	44.937355000	46.814110000	43.035566000
1	44.661953000	48.496000000	43.552155000
1	43.346940000	47.308793000	43.676639000
8	43.913472000	38.267182000	39.214532000
26	44.139291000	36.373287000	39.809331000
7	42.856013000	36.797231000	41.308067000
7	45.720437000	36.814414000	41.001066000
7	42.571557000	35.957302000	38.618312000
6	40.727643000	36.534642000	40.125051000
6	44.490281000	37.248436000	43.084133000
6	47.571053000	36.377366000	39.451545000
6	43.807509000	35.192615000	36.639682000
6	41.487731000	36.819202000	41.253894000
6	45.656158000	37.123645000	42.334453000
6	46.811684000	35.927229000	38.375283000
6	42.639989000	35.492407000	37.333690000
6	40.937938000	37.157311000	42.551348000
6	46.987630000	37.321481000	42.863314000
6	47.357733000	35.416918000	37.138919000
6	41.308210000	35.363577000	36.780366000
6	42.000122000	37.329933000	43.385957000
6	47.854938000	37.113510000	41.834458000
6	46.297289000	35.063188000	36.357955000
6	40.441505000	35.762681000	37.750536000
6	43.194172000	37.106232000	42.598596000
6	47.053773000	36.773469000	40.679943000
6	45.103606000	35.363586000	37.115530000
6	41.243828000	36.125378000	38.899223000
7	45.442963000	35.891456000	38.335746000
1	39.647588000	36.600701000	40.219730000
1	44.598946000	37.516118000	44.131003000
1	48.651637000	36.368035000	39.341822000
1	43.698972000	34.804806000	35.630758000
6	45.161153000	41.055706000	34.481423000
6	44.748510000	41.228334000	36.680032000
6	44.470769000	41.573342000	38.027388000
6	45.169235000	38.875567000	37.269942000
6	45.109245000	39.857814000	36.349237000
6	44.782172000	41.968567000	35.487827000
6	44.528454000	40.609962000	39.002240000
6	44.878390000	39.133473000	38.732440000
7	45.339541000	39.818304000	34.966371000
6	44.532219000	43.436247000	35.290195000

6	43.044485000	43.856767000	35.334198000
7	42.898605000	45.299428000	35.239984000
6	42.478177000	45.936076000	34.124724000
8	42.139221000	45.346397000	33.085043000
6	42.425737000	47.451178000	34.221976000
8	44.282803000	41.034570000	40.256032000
6	44.486949000	40.146083000	41.387085000
1	45.331131000	41.240174000	33.424696000
1	44.229033000	42.596572000	38.301981000
1	42.998360000	47.878282000	33.392159000
1	45.557478000	40.083931000	41.599765000
1	44.065302000	39.174093000	41.155994000
1	43.978461000	40.623524000	42.222736000
1	45.388319000	37.848599000	37.010811000
1	45.866302000	38.995049000	39.241399000
1	45.066697000	43.998613000	36.064408000
1	44.949771000	43.755235000	34.327895000
1	42.589599000	43.516333000	36.271968000
1	42.493169000	43.404923000	34.507384000
1	42.805436000	47.848655000	35.166061000
1	41.386688000	47.776153000	34.100482000
1	45.769430000	39.053820000	34.397707000
1	43.209021000	45.852101000	36.047761000
8	46.579383000	42.719346000	41.523354000
1	45.878836000	42.517343000	40.883359000
1	46.614866000	43.704284000	41.542793000
1	43.390158000	31.903670000	42.172790000
1	41.733934000	31.664787000	41.574280000
1	46.306234000	34.646888000	35.358281000
1	48.414443000	35.337415000	36.915392000
1	48.936995000	37.161383000	41.843000000
1	47.210071000	37.584622000	43.890149000
1	41.997592000	37.611035000	44.431752000
1	39.881951000	37.266930000	42.771025000
1	39.359969000	35.809025000	37.713454000
1	41.087684000	35.018725000	35.777492000
1	49.416291000	47.555926000	41.198768000
1	48.001951000	47.445209000	36.892876000
1	42.655666000	49.456462000	40.549035000
1	39.073301000	47.938973000	42.613059000
1	41.587363000	41.179444000	40.089644000
1	42.826854000	44.908386000	42.270668000
1	33.636530000	37.474830000	41.317328000
1	36.462272000	37.202974000	44.910840000
1	44.368574000	41.052492000	28.573947000
1	41.777594000	43.235746000	31.995282000
1	41.719393000	48.924684000	30.240460000
1	40.001301000	49.925738000	39.421271000
1	39.441852000	53.340446000	35.493017000
1	44.516711000	53.805229000	36.628682000
1	50.134253000	41.948834000	39.914832000
1	50.613254000	43.636701000	35.814778000
1	49.884663000	38.661128000	36.013768000
1	52.726699000	40.813646000	33.296487000

^{4}IM1$_{C6,II}$

6	41.616057000	42.496067000	31.295558000
6	42.666618000	42.349761000	30.200082000
8	42.705424000	43.085426000	29.208860000
6	40.198416000	42.768973000	30.756141000
6	39.271931000	43.183307000	31.916737000
6	39.666198000	41.563476000	29.966570000
6	37.878439000	43.655086000	31.484128000
1	41.607580000	41.603793000	31.937985000
1	40.278365000	43.620063000	30.068468000
1	39.170573000	42.341900000	32.619591000
1	39.763655000	43.991734000	32.473026000
1	38.686430000	41.770975000	29.522158000
1	40.347015000	41.301612000	29.149006000
1	39.559556000	40.682774000	30.615543000
1	37.299444000	42.854010000	31.009831000
1	37.301903000	44.009919000	32.346702000
1	37.950124000	44.483430000	30.768164000
7	43.580273000	41.360802000	30.391113000
6	44.787511000	41.249612000	29.583522000
6	45.627164000	40.089540000	30.106661000
8	45.855883000	40.314310000	31.481668000
1	43.641138000	40.920866000	31.300787000
1	45.364502000	42.183194000	29.634742000
1	45.089147000	39.140885000	29.942187000
1	46.568116000	40.041791000	29.537073000
1	46.235585000	39.501613000	31.917621000
6	40.648688000	41.497364000	40.596689000
6	40.930544000	42.451804000	41.749918000
8	40.036089000	42.707643000	42.582779000
6	39.892825000	40.258468000	41.087330000
8	38.638396000	40.608802000	41.690411000
1	40.027407000	42.036534000	39.869379000
1	39.678148000	39.591097000	40.248395000
1	40.515785000	39.704041000	41.801584000
1	38.843772000	41.426579000	42.208572000
7	42.160157000	42.998360000	41.802741000
6	42.561635000	44.020671000	42.772904000
6	43.799321000	43.629702000	43.589162000
6	43.685771000	42.424393000	44.507011000
6	42.489116000	42.054476000	45.138385000
6	44.840998000	41.673882000	44.781735000
6	42.455388000	40.983675000	46.035422000
6	44.810066000	40.606972000	45.681015000
6	43.615848000	40.260388000	46.318313000
1	42.834165000	42.718120000	41.098275000
1	41.691662000	44.198654000	43.407546000
1	44.650684000	43.479801000	42.914220000
1	44.061965000	44.512355000	44.191276000
1	41.569369000	42.588177000	44.917209000
1	45.764832000	41.928808000	44.267237000
1	41.516431000	40.715351000	46.513937000
1	45.719566000	40.045004000	45.881619000
1	43.588669000	39.432848000	47.023501000
6	43.771646000	53.045359000	36.568002000
6	42.371256000	53.620395000	36.808187000
8	42.195466000	54.583887000	37.547772000
6	44.176731000	51.974611000	37.600733000
6	43.594362000	50.587064000	37.318957000
7	44.171161000	49.565230000	37.961683000
8	42.623953000	50.428179000	36.550257000
1	43.859913000	52.633730000	35.556657000
1	45.269675000	51.893540000	37.644954000
1	43.857956000	52.289677000	38.603331000
1	45.027548000	49.644190000	38.515695000
1	43.840250000	48.611297000	37.789599000
7	41.361329000	52.986304000	36.133984000
6	39.964016000	53.250157000	36.423199000
6	39.209968000	52.198051000	37.261879000
8	38.016019000	52.368778000	37.498907000
1	41.613527000	52.091958000	35.725572000
1	39.922171000	54.195221000	36.970164000
7	39.942302000	51.139876000	37.688993000
6	39.397503000	50.044933000	38.475657000
6	39.442464000	48.703763000	37.707567000
6	39.164313000	47.523434000	38.612802000
6	37.854456000	47.185271000	38.983735000
6	40.224024000	46.777974000	39.150450000
6	37.607132000	46.136841000	39.873052000
6	39.978739000	45.732815000	40.044953000
6	38.670298000	45.407629000	40.413351000
1	40.904830000	51.040197000	37.378909000
1	38.372342000	50.314008000	38.740169000
1	38.719446000	48.747014000	36.882965000
1	40.437276000	48.598201000	37.258749000
1	37.020490000	47.749859000	38.570547000
1	41.251345000	47.005119000	38.874562000
1	36.583386000	45.889625000	40.144747000
1	40.819545000	45.180268000	40.452069000
1	38.491352000	44.594894000	41.112514000
6	41.224751000	48.230034000	30.275139000
6	41.566314000	47.040263000	29.376805000
7	42.012948000	45.940551000	30.031136000
8	41.448821000	47.117019000	28.155351000
1	41.203236000	47.979175000	31.339556000
1	40.250343000	48.627672000	29.975664000
1	42.239741000	45.096374000	29.508468000
1	42.076725000	45.900414000	31.046056000
6	51.863702000	41.245521000	33.464406000
6	50.987636000	40.707318000	34.599649000
8	50.997619000	41.246845000	35.718608000
6	51.252373000	41.196462000	32.051440000
6	49.953655000	42.011562000	31.865066000
6	50.214730000	43.524972000	31.900090000
6	49.243197000	41.594152000	30.569402000
1	52.131453000	42.268166000	33.745848000
1	52.006712000	41.561338000	31.338454000
1	51.060127000	40.154218000	31.767446000
1	49.276255000	41.761961000	32.693473000
1	50.683791000	43.844533000	32.838430000
1	50.876398000	43.825692000	31.075824000
1	49.278324000	44.085092000	31.793527000
1	49.892482000	41.746879000	29.695415000
1	48.957697000	40.538545000	30.621441000
1	48.330300000	42.181786000	30.417738000
7	50.265828000	39.605626000	34.322427000
6	49.283553000	39.046807000	35.245134000
6	48.549821000	37.873251000	34.570415000
6	47.950595000	38.287616000	33.208740000
8	48.759716000	38.532689000	32.284917000
8	46.677796000	38.381361000	33.130367000
1	50.124747000	39.299725000	33.355290000
1	48.568152000	39.825346000	35.543178000
1	49.267558000	37.062327000	34.393119000
1	47.773237000	37.499548000	35.243000000
6	50.095946000	44.436091000	36.417855000
6	49.637849000	43.949543000	37.782635000
8	49.214437000	44.744172000	38.640378000
1	50.765613000	45.290759000	36.555892000
1	49.224201000	44.790054000	35.855018000
7	49.702266000	42.621426000	37.989439000

6	49.284909000	41.997130000	39.243764000
6	48.876892000	40.542991000	39.020554000
1	50.100161000	42.032390000	37.257191000
1	48.454171000	42.568238000	39.666621000
1	49.698467000	39.955730000	38.592195000
1	48.589606000	40.083672000	39.972027000
1	48.021986000	40.476127000	38.338353000
6	47.034594000	47.629582000	37.423585000
6	47.270756000	48.045700000	38.873510000
8	46.661587000	48.994080000	39.393191000
6	46.275664000	46.281784000	37.312083000
6	44.973366000	46.292280000	38.131853000
8	45.065834000	45.938122000	39.346270000
8	43.913152000	46.691793000	37.572175000
1	46.463600000	48.423583000	36.936709000
1	46.044756000	46.113431000	36.255382000
1	46.921954000	45.474047000	37.667506000
7	48.171360000	47.283217000	39.534702000
6	48.352217000	47.314199000	40.978355000
6	48.079005000	45.934895000	41.600046000
6	48.439088000	45.908382000	43.082191000
8	46.701624000	45.570856000	41.497963000
1	48.592132000	46.490166000	39.044741000
1	47.669973000	48.065099000	41.384997000
1	48.691837000	45.196892000	41.062232000
1	46.336477000	45.758459000	40.597023000
1	47.863201000	46.661368000	43.632641000
1	48.207078000	44.925585000	43.504799000
1	49.507782000	46.104060000	43.229080000
6	36.253730000	38.171519000	45.070587000
6	34.968367000	38.103954000	44.234829000
8	33.853697000	38.183933000	44.748366000
6	37.335137000	39.120327000	44.526266000
6	38.454865000	39.359688000	45.541211000
8	37.868389000	38.550242000	43.324507000
1	35.951983000	38.479106000	46.075511000
1	36.862757000	40.086448000	44.288203000
1	38.170113000	39.295104000	42.744516000
1	38.923487000	38.409154000	45.822109000
1	39.226614000	40.005477000	45.107551000
1	38.071902000	39.842668000	46.448587000
7	35.183289000	37.935330000	42.899510000
6	34.135253000	38.002938000	41.896854000
6	34.419190000	39.076007000	40.824673000
6	34.380405000	40.500731000	41.413780000
6	33.446099000	38.911355000	39.647708000
6	35.125586000	41.545637000	40.573725000
1	36.158969000	37.970372000	42.608065000
1	33.200203000	38.211856000	42.426561000
1	35.438760000	38.896005000	40.447230000
1	33.329925000	40.799030000	41.548529000
1	34.822651000	40.481243000	42.417214000
1	33.634541000	39.652296000	38.863449000
1	33.532872000	37.916941000	39.192475000
1	32.407105000	39.036662000	39.981030000
1	35.009879000	42.547847000	41.003284000
1	36.198411000	41.321485000	40.541166000
1	34.754549000	41.588159000	39.542375000
6	42.377424000	31.914708000	41.611528000
6	42.421490000	33.388880000	41.201729000
16	44.029940000	33.837242000	40.430511000
1	42.524895000	31.263417000	40.742390000
1	41.612721000	33.598655000	40.491452000
1	42.249010000	34.021819000	42.080252000
6	39.363390000	48.580988000	43.397225000
6	40.679551000	49.271929000	43.044686000
8	41.072463000	50.266116000	43.660515000
6	39.447701000	47.736044000	44.688135000
6	39.865590000	48.474858000	45.976183000
6	38.955618000	49.671162000	46.290745000
6	39.904019000	47.486215000	47.151765000
1	38.611186000	49.368320000	43.513756000
1	38.461961000	47.274102000	44.849591000
1	40.146397000	46.904965000	44.516950000
1	40.877626000	48.869277000	45.823621000
1	39.061375000	50.447257000	45.527633000
1	37.899934000	49.368265000	46.343835000
1	39.221124000	50.116153000	47.258289000
1	40.573376000	46.641609000	46.946045000
1	40.254412000	47.975380000	48.069441000
1	38.905513000	47.074972000	47.355418000
7	41.373880000	48.698936000	42.025450000
6	42.681985000	49.144018000	41.571938000
6	43.713691000	48.004365000	41.625467000
6	44.162589000	47.667867000	43.049109000
8	43.123981000	46.860442000	41.005528000
1	41.019688000	47.860381000	41.581852000
1	42.975371000	49.986064000	42.203579000
1	44.590126000	48.327416000	41.047380000
1	43.768237000	46.486140000	40.351603000
1	44.903833000	46.863154000	43.014768000
1	44.614470000	48.540995000	43.537410000
1	43.308669000	47.343014000	43.655114000
8	43.832502000	38.387836000	39.121305000
26	43.933547000	36.306490000	39.771956000
7	42.893596000	36.792364000	41.427791000
7	45.676930000	36.639483000	40.766370000
7	42.201460000	36.024267000	38.766728000
6	40.610142000	36.584741000	40.545188000
6	44.761002000	37.233913000	42.965232000
6	47.271309000	36.032147000	39.001712000
6	43.117486000	35.314794000	36.603091000
6	41.528592000	36.847891000	41.554867000
6	45.809592000	37.003385000	42.081184000
6	46.353078000	35.683399000	38.014560000
6	42.071696000	35.631325000	37.460846000
6	41.168595000	37.234413000	42.900128000
6	47.208451000	37.104309000	42.427579000
6	46.705895000	35.207381000	36.698728000
6	40.673223000	35.569742000	37.100272000
6	42.336861000	37.411701000	43.579971000
6	47.915071000	36.779315000	41.308953000
6	45.537224000	35.010757000	36.022541000
6	39.966595000	35.925535000	38.208320000
6	43.409262000	37.132059000	42.653830000
6	46.949088000	36.476019000	40.278594000
6	44.469087000	35.359737000	36.927111000
6	40.933373000	36.205495000	39.246227000
7	44.989873000	35.766938000	38.130036000
1	39.557712000	36.676441000	40.796806000
1	45.015506000	37.529380000	43.978451000
1	48.325052000	35.929372000	38.759871000
1	42.859115000	34.998231000	35.596941000
6	45.171323000	41.148216000	34.430478000
6	44.742097000	41.328254000	36.626648000
6	44.458140000	41.683661000	37.971617000
6	45.129164000	38.970730000	37.221458000
6	45.088000000	39.952357000	36.298252000
6	44.793616000	42.065720000	35.432347000
6	44.502095000	40.718595000	38.944882000
6	44.816945000	39.235714000	38.676905000
7	45.327358000	39.908325000	34.917239000
6	44.559332000	43.535371000	35.230344000
6	43.076222000	43.970801000	35.284245000
7	42.940383000	45.414429000	35.186707000
6	42.556918000	46.051994000	34.059168000
8	42.248629000	45.462401000	33.009705000
6	42.508011000	47.567520000	34.152232000
8	44.262855000	41.132815000	40.205332000
6	44.447582000	40.214560000	41.318005000
1	45.360429000	41.330552000	33.376794000
1	44.226472000	42.710883000	38.240536000
1	43.135160000	47.990386000	33.360266000
1	45.514872000	40.145639000	41.545512000
1	44.028226000	39.248220000	41.052898000
1	43.927656000	40.671733000	42.158235000
1	45.336133000	37.937919000	36.969988000
1	45.798892000	39.078773000	39.202679000
1	45.105497000	44.095368000	35.998077000
1	44.972435000	43.845611000	34.263287000
1	42.625559000	43.639842000	36.227073000
1	42.514864000	43.519419000	34.463877000
1	42.829394000	47.964369000	35.118086000
1	41.481020000	47.898404000	33.963687000
1	45.761511000	39.137802000	34.359831000
1	43.232522000	45.964375000	36.003169000
8	46.559758000	42.767563000	41.509230000
1	45.858520000	42.563385000	40.870231000
1	46.593227000	43.752723000	41.527968000
1	43.168666000	31.686778000	42.335001000
1	41.409651000	31.662281000	42.070236000
1	45.398816000	34.667935000	35.004988000
1	47.718473000	35.049366000	36.349257000
1	48.989480000	36.732587000	41.180261000
1	47.583226000	37.383172000	43.404585000
1	42.478932000	37.730083000	44.604893000
1	40.150949000	37.376683000	43.245489000
1	38.892719000	35.995819000	38.330363000
1	40.300143000	35.287899000	36.123408000
1	49.380975000	47.618311000	41.219812000
1	47.996483000	47.536267000	36.903510000
1	42.604816000	49.496912000	40.535260000
1	39.030075000	47.933985000	42.577069000
1	41.567093000	41.186583000	40.088664000
1	42.776844000	44.951357000	42.234522000
1	34.023560000	37.023484000	41.407497000
1	36.691820000	37.166240000	45.146181000
1	44.516166000	41.091341000	28.534026000
1	41.928333000	43.342091000	31.923704000
1	41.963459000	49.023846000	30.114371000
1	39.971012000	49.948072000	39.406592000
1	39.395197000	53.379462000	35.494972000
1	44.459445000	53.891052000	36.655479000
1	50.106022000	42.048933000	39.974051000
1	50.600214000	43.655605000	35.843300000
1	49.784051000	38.711333000	36.161182000

1	52.800090000	40.668983000	33.474013000

^{2}TS1$_{HA,II}$

6	40.212668000	45.092868000	33.340810000
6	40.476167000	44.582313000	31.928480000
8	40.076785000	45.174469000	30.919378000
6	38.813577000	45.708233000	33.529424000
6	38.763090000	46.468209000	34.869986000
6	37.717247000	44.639990000	33.403185000
6	37.474815000	47.262855000	35.113674000
1	40.374669000	44.288926000	34.072686000
1	38.674991000	46.436845000	32.720246000
1	38.913501000	45.758989000	35.698180000
1	39.616120000	47.157804000	34.902836000
1	36.714254000	45.076496000	33.469419000
1	37.787457000	44.123621000	32.439348000
1	37.807355000	43.888060000	34.200053000
1	36.595332000	46.612587000	35.191635000
1	37.546376000	47.833314000	36.047310000
1	37.291121000	47.975625000	34.299594000
7	41.203672000	43.437886000	31.848415000
6	41.723240000	42.918758000	30.592294000
6	42.313005000	41.533576000	30.832418000
8	43.303555000	41.650218000	31.831715000
1	41.661797000	43.076897000	32.676628000
1	42.498990000	43.587263000	30.191797000
1	41.511380000	40.843366000	31.143027000
1	42.729264000	41.153581000	29.884860000
1	43.483633000	40.754893000	32.227688000
6	40.137795000	37.556804000	40.611053000
6	39.280582000	37.923683000	39.412213000
8	38.133661000	37.449709000	39.292671000
6	39.309369000	37.633578000	41.900841000
8	38.220431000	36.701744000	41.889750000
1	40.483584000	36.523031000	40.480024000
1	39.939129000	37.389880000	42.758943000
1	38.919253000	38.652397000	42.040448000
1	37.842252000	36.789820000	40.982440000
7	39.815137000	38.774911000	38.509485000
6	39.085409000	39.282796000	37.353638000
6	39.134002000	40.820637000	37.259352000
6	38.589556000	41.529008000	38.483410000
6	37.228708000	41.454879000	38.813293000
6	39.439022000	42.262807000	39.322667000
6	36.730673000	42.090146000	39.951285000
6	38.944280000	42.900840000	40.464296000
6	37.587770000	42.814952000	40.782703000
1	40.741076000	39.149868000	38.683282000
1	38.059443000	38.923751000	37.457743000
1	40.168019000	41.137492000	37.078919000
1	38.563228000	41.108871000	36.366219000
1	36.551254000	40.893533000	38.173146000
1	40.495124000	42.343144000	39.075181000
1	35.671673000	42.020976000	40.187530000
1	39.619284000	43.467925000	41.100399000
1	37.201506000	43.311774000	41.668816000
6	46.601517000	54.289436000	38.876246000
6	45.412096000	54.787903000	39.703453000
8	45.566108000	55.240413000	40.833978000
6	47.014528000	52.847794000	39.225496000
6	46.102431000	51.781442000	38.611974000
7	46.558457000	50.526219000	38.662398000
8	44.995635000	52.077677000	38.118352000
1	46.397235000	54.365846000	37.802967000
1	48.044746000	52.660396000	38.898654000
1	47.015860000	52.721219000	40.316041000
1	47.510801000	50.283315000	38.951261000
1	46.003169000	49.764877000	38.264387000
7	44.193846000	54.701120000	39.081903000
6	42.948068000	54.926826000	39.792447000
6	42.069747000	53.689341000	40.066148000
8	40.933370000	53.850138000	40.507395000
1	44.175346000	54.118140000	38.252714000
1	43.208759000	55.375821000	40.754863000
7	42.639549000	52.487754000	39.805518000
6	41.948760000	51.216777000	39.951444000
6	41.785723000	50.502449000	38.589775000
6	41.254124000	49.093360000	38.735156000
6	39.885648000	48.860685000	38.935646000
6	42.121970000	47.989529000	38.706816000
6	39.392947000	47.564829000	39.105350000
6	41.631619000	46.691261000	38.884582000
6	40.265199000	46.473939000	39.083353000
1	43.558681000	52.451195000	39.371327000
1	40.979243000	51.423610000	40.410632000
1	41.115554000	51.103231000	37.961604000
1	42.763107000	50.486598000	38.094347000
1	39.199032000	49.704916000	38.951425000
1	43.186464000	48.137277000	38.532981000
1	38.327310000	47.406216000	39.252155000
1	42.322425000	45.852081000	38.867493000
1	39.881760000	45.465193000	39.209827000
6	41.085050000	50.534470000	31.514854000
6	40.480677000	49.330390000	30.789441000
7	40.784766000	48.131422000	31.340930000
8	39.785718000	49.477398000	29.785436000
1	41.629008000	50.261762000	32.423761000
1	40.280691000	51.232273000	31.770126000
1	40.412551000	47.271609000	30.943746000
1	41.352967000	48.062442000	32.180900000
6	49.258550000	40.796443000	31.604524000
6	48.712991000	40.693096000	33.032628000
8	49.205782000	41.370132000	33.949700000
6	48.236751000	40.642005000	30.462532000
6	47.110833000	41.698241000	30.428132000
6	47.639743000	43.094674000	30.067079000
6	45.997779000	41.255410000	29.467999000
1	49.772230000	41.760577000	31.545501000
1	48.784860000	40.677487000	29.508682000
1	47.782348000	39.644178000	30.503052000
1	46.658900000	41.752108000	31.427367000
1	48.402038000	43.446418000	30.772476000
1	48.086125000	43.096929000	29.062418000
1	46.826648000	43.829925000	30.070421000
1	46.386355000	41.111251000	28.449162000
1	45.553613000	40.317755000	29.817488000
1	45.202263000	42.007670000	29.425027000
7	47.728244000	39.798257000	33.229737000
6	46.998279000	39.679158000	34.488272000
6	45.855655000	38.657275000	34.327583000
6	44.976436000	38.998536000	33.100730000
8	45.495181000	38.813793000	31.974623000
8	43.817324000	39.485472000	33.321681000
1	47.226310000	39.386120000	32.437412000
1	46.592839000	40.657491000	34.776983000
1	46.285891000	37.659529000	34.176043000
1	45.265059000	38.644212000	35.248361000
6	50.898482000	44.279530000	34.219701000
6	50.331187000	44.425090000	35.623138000
8	50.645390000	45.394185000	36.336296000
1	51.990011000	44.349558000	34.273980000
1	50.549961000	45.119870000	33.608537000
7	49.485798000	43.456608000	36.019114000
6	48.875801000	43.443386000	37.348360000
6	47.803494000	42.366234000	37.459877000
1	49.294380000	42.681035000	35.384843000
1	48.440706000	44.424872000	37.559768000
1	48.219111000	41.370455000	37.259753000
1	47.391925000	42.386029000	38.473430000
1	46.986278000	42.541128000	36.751665000
6	48.734307000	48.581625000	36.782375000
6	49.406281000	48.516349000	38.153059000
8	49.181506000	49.361712000	39.033271000
6	47.721949000	47.435244000	36.535932000
6	46.698275000	47.280675000	37.672954000
8	47.005023000	46.486952000	38.612712000
8	45.627677000	47.953706000	37.610855000
1	48.234867000	49.550708000	36.712392000
1	47.188402000	47.645743000	35.602910000
1	48.269887000	46.498053000	36.415504000
7	50.250828000	47.474216000	38.331042000
6	50.835837000	47.140917000	39.619784000
6	50.394179000	45.747726000	40.094790000
6	51.088842000	45.355936000	41.395353000
8	48.993746000	45.701331000	40.337313000
1	50.359047000	46.787837000	37.581126000
1	50.514717000	47.904149000	40.332869000
1	50.664355000	45.025091000	39.307878000
1	48.481871000	46.046625000	39.566693000
1	50.851058000	46.077241000	42.185760000
1	50.746979000	44.368951000	41.723338000
1	52.176754000	45.320288000	41.265150000
6	37.787345000	31.998285000	42.459897000
6	37.685660000	31.667564000	40.964259000
8	37.043223000	30.696727000	40.566753000
6	37.720365000	33.486933000	42.829543000
6	37.553815000	33.699958000	44.335784000
8	38.923518000	34.114291000	42.373438000
1	36.976447000	31.450053000	42.947926000
1	36.858333000	33.938286000	42.313419000
1	38.723784000	35.074626000	42.228283000
1	38.390685000	33.245046000	44.879158000
1	37.539268000	34.771848000	44.562243000
1	36.618701000	33.257896000	44.701462000
7	38.377349000	32.510872000	40.148375000
6	38.412041000	32.384947000	38.701794000
6	37.855391000	33.616476000	37.954930000
6	36.357016000	33.825098000	38.245819000
6	38.147990000	33.473027000	36.453675000
6	35.786703000	35.146688000	37.714759000
1	38.860827000	33.279656000	40.608512000
1	37.833721000	31.489683000	38.453209000
1	38.391203000	34.505864000	38.322515000
1	35.791441000	32.976973000	37.831249000
1	36.208682000	33.786027000	39.332267000
1	37.829606000	34.357799000	35.893570000

1	39.220528000	33.335137000	36.269342000
1	37.623188000	32.603861000	36.034070000
1	34.743401000	35.273138000	38.029283000
1	36.359732000	36.001434000	38.092378000
1	35.801908000	35.188974000	36.619194000
6	46.711639000	34.634013000	45.301191000
6	45.578368000	35.527559000	44.790180000
16	46.199068000	36.647835000	43.472122000
1	47.120005000	34.014940000	44.494880000
1	44.768221000	34.913923000	44.384306000
1	45.166038000	36.123664000	45.609854000
6	41.469623000	47.989108000	42.813990000
6	42.991207000	48.036891000	42.934758000
8	43.545223000	48.309187000	44.002224000
6	40.864212000	46.666225000	43.335656000
6	41.178200000	46.289458000	44.797720000
6	40.750589000	47.379716000	45.791052000
6	40.522692000	44.942507000	45.139518000
1	41.079643000	48.833489000	43.390929000
1	39.772345000	46.727661000	43.212287000
1	41.197884000	45.848375000	42.681262000
1	42.265383000	46.173975000	44.889678000
1	41.340055000	48.289011000	45.644396000
1	39.685110000	47.626724000	45.678562000
1	40.905421000	47.045036000	46.824774000
1	40.846682000	44.151715000	44.451111000
1	40.778130000	44.623767000	46.157788000
1	39.427390000	45.008577000	45.077917000
7	43.675715000	47.745155000	41.792266000
6	45.126355000	47.639839000	41.738172000
6	45.653085000	46.192786000	41.710829000
6	45.133411000	45.349927000	42.868627000
8	45.289476000	45.529963000	40.492008000
1	43.155741000	47.465207000	40.970814000
1	45.510534000	48.154815000	42.622680000
1	46.750202000	46.258190000	41.771906000
1	45.807370000	45.958741000	39.759866000
1	45.602246000	44.363130000	42.851204000
1	45.342137000	45.835081000	43.827945000
1	44.050117000	45.214880000	42.780382000
8	43.065213000	38.624119000	41.750124000
26	44.411212000	37.890971000	42.573907000
7	43.343151000	37.687961000	44.273665000
7	45.184567000	39.595241000	43.245939000
7	43.635800000	36.127410000	41.900993000
6	42.010234000	35.701235000	43.686017000
6	44.139400000	39.678705000	45.466703000
6	46.595112000	40.203491000	41.323025000
6	44.931580000	35.903372000	39.834060000
6	42.369728000	36.750172000	44.524641000
6	44.969875000	40.175628000	44.475093000
6	46.394422000	39.045484000	40.582881000
6	43.964405000	35.484511000	40.739532000
6	41.769843000	37.003469000	45.810419000
6	45.706957000	41.414454000	44.568679000
6	47.015545000	38.771459000	39.309885000
6	43.124375000	34.321477000	40.566115000
6	42.391603000	38.100957000	46.329432000
6	46.361327000	41.580566000	43.386787000
6	46.574804000	37.546682000	38.912417000
6	42.276962000	34.282422000	41.632596000
6	43.359377000	38.531926000	45.352409000
6	46.046912000	40.429229000	42.575739000
6	45.666469000	37.076062000	39.930879000
6	42.615532000	35.412894000	42.468101000
7	45.589547000	37.997952000	40.946543000
1	41.197256000	35.053439000	43.999927000
1	44.054497000	40.253400000	46.383927000
1	47.218279000	40.991207000	40.911501000
1	45.093290000	35.287236000	38.954769000
6	43.890394000	42.608746000	35.023676000
6	43.740035000	42.526718000	37.267480000
6	43.755246000	42.690194000	38.664930000
6	43.152123000	40.134854000	37.538989000
6	43.430892000	41.238353000	36.722601000
6	44.028335000	43.392419000	36.152280000
6	43.457291000	41.586258000	39.457095000
6	43.162026000	40.311944000	38.919482000
7	43.517783000	41.326500000	35.357214000
6	44.412739000	44.848321000	36.185828000
6	43.343207000	45.755919000	35.555767000
7	43.772677000	47.148778000	35.540024000
6	43.230904000	48.061222000	34.708932000
8	42.353792000	47.783625000	33.874758000
6	43.772869000	49.475276000	34.845225000
8	43.552322000	41.776537000	40.833335000
6	42.697060000	41.118270000	41.661538000
1	44.033097000	42.856712000	33.979220000
1	44.022825000	43.628315000	39.145327000
1	44.174497000	49.795804000	33.877808000
1	42.925642000	39.827973000	41.728574000
1	41.656675000	41.098544000	41.313525000
1	42.841261000	41.461665000	42.684943000
1	42.976567000	39.152355000	37.107865000
1	43.049533000	39.463246000	39.587036000
1	44.599718000	45.160070000	37.219848000
1	45.357959000	45.003976000	35.647924000
1	42.395416000	45.653360000	36.102067000
1	43.149556000	45.450221000	34.523713000
1	44.548606000	49.563568000	35.610266000
1	42.947131000	50.149576000	35.095288000
1	43.522819000	40.540643000	34.679445000
1	44.433171000	47.462613000	36.258101000
8	47.324927000	43.438056000	40.817904000
1	46.498506000	43.934907000	40.644046000
1	48.016387000	44.121907000	40.693829000
1	47.532127000	35.232102000	45.713565000
1	46.344604000	33.967168000	46.093422000
1	46.811326000	37.006681000	38.004172000
1	47.689948000	39.448624000	38.802726000
1	46.987224000	42.391922000	43.037792000
1	45.695605000	42.069400000	45.431055000
1	42.209348000	38.596572000	47.274928000
1	40.976350000	36.408014000	46.244808000
1	41.481758000	33.582192000	41.852127000
1	43.180851000	33.642923000	39.723829000
1	51.932840000	47.167290000	39.549493000
1	49.501992000	48.537822000	35.999491000
1	45.486952000	48.165177000	40.846112000
1	41.153034000	48.129039000	41.774174000
1	41.035813000	38.173772000	40.712772000
1	39.498943000	38.851756000	36.432315000
1	39.448943000	32.209282000	38.379030000
1	38.734196000	31.593772000	42.845855000
1	40.912968000	42.880003000	29.857290000
1	40.969602000	45.862549000	33.544705000
1	41.763533000	51.057652000	30.831745000
1	42.517309000	50.570921000	40.632987000
1	42.314648000	55.641952000	39.254933000
1	47.434029000	54.957995000	39.115186000
1	49.655778000	43.277090000	38.104342000
1	50.607144000	43.338410000	33.747515000
1	47.683664000	39.370821000	35.286864000
1	50.038037000	40.027576000	31.499728000

^{4}TS1$_{HA,II}$

6	40.290528000	45.164631000	33.305191000
6	40.552178000	44.592289000	31.916501000
8	40.164578000	45.149263000	30.882706000
6	38.889048000	45.782530000	33.468773000
6	38.833768000	46.587876000	34.782475000
6	37.795215000	44.708201000	33.376883000
6	37.542463000	47.386105000	34.996644000
1	40.456963000	44.395635000	34.072427000
1	38.750799000	46.482467000	32.634518000
1	38.984893000	45.908215000	35.634827000
1	39.684652000	47.280924000	34.793362000
1	36.791419000	45.144977000	33.427889000
1	37.866905000	44.160447000	32.430625000
1	37.885731000	43.982985000	34.198011000
1	36.664633000	46.735805000	35.091503000
1	37.608732000	47.984952000	35.912714000
1	37.359631000	48.072883000	34.160254000
7	41.260001000	43.433435000	31.885378000
6	41.756103000	42.843916000	30.651064000
6	42.295568000	41.448521000	30.944441000
8	43.301798000	41.569747000	31.926540000
1	41.707516000	43.095012000	32.728840000
1	42.554755000	43.465861000	30.221492000
1	41.471707000	40.803921000	31.292798000
1	42.684820000	41.012912000	30.009208000
1	43.456451000	40.686812000	32.359357000
6	40.075288000	37.581331000	40.673319000
6	39.195169000	37.969262000	39.498136000
8	38.063963000	37.461268000	39.364839000
6	39.261759000	37.547136000	41.973214000
8	38.202834000	36.581948000	41.917248000
1	40.464671000	36.572600000	40.481195000
1	39.912017000	37.271353000	42.806391000
1	38.840697000	38.541019000	42.183607000
1	37.817466000	36.705547000	41.016873000
7	39.696484000	38.868894000	38.624012000
6	38.961820000	39.361500000	37.464353000
6	38.999887000	40.897812000	37.353389000
6	38.409769000	41.617384000	38.549146000
6	37.033849000	41.564464000	38.814214000
6	39.229577000	42.341196000	39.425178000
6	36.492200000	42.211739000	39.925153000
6	38.691669000	42.990325000	40.540408000
6	37.320453000	42.926282000	40.794029000
1	40.611854000	39.264941000	38.805458000
1	37.938153000	38.996403000	37.571163000
1	40.036427000	41.221330000	37.200759000
1	38.454634000	41.168903000	36.439300000
1	36.379326000	41.011667000	38.143224000
1	40.297422000	42.402758000	39.228739000

1	35.422117000	42.160039000	40.111006000
1	39.346426000	43.545228000	41.207398000
1	36.900181000	43.431973000	41.659560000
6	46.695427000	54.244497000	38.940482000
6	45.505746000	54.749842000	39.763541000
8	45.659453000	55.212815000	40.890065000
6	47.087620000	52.793284000	39.274546000
6	46.170362000	51.744199000	38.638919000
7	46.613479000	50.484036000	38.677517000
8	45.071091000	52.056782000	38.138434000
1	46.499917000	54.336168000	37.866677000
1	48.118738000	52.598418000	38.954953000
1	47.077553000	52.651894000	40.363266000
1	47.558821000	50.226627000	38.976380000
1	46.052642000	49.732923000	38.267314000
7	44.288343000	54.654868000	39.142536000
6	43.041558000	54.886334000	39.849029000
6	42.150281000	53.655400000	40.109230000
8	41.013329000	53.825532000	40.545621000
1	44.272941000	54.066208000	38.316885000
1	43.301538000	55.326627000	40.815704000
7	42.707618000	52.448841000	39.843444000
6	41.996998000	51.186427000	39.973257000
6	41.826259000	50.490157000	38.603702000
6	41.245990000	49.098093000	38.727793000
6	39.871452000	48.914019000	38.938084000
6	42.069839000	47.962497000	38.663703000
6	39.329718000	47.634925000	39.081891000
6	41.529526000	46.680348000	38.811674000
6	40.157754000	46.511578000	39.020503000
1	43.627043000	52.405014000	39.411041000
1	41.029309000	51.404592000	40.430966000
1	41.182092000	51.118356000	37.974946000
1	42.806914000	50.447478000	38.116254000
1	39.218766000	49.783885000	38.981442000
1	43.138119000	48.074768000	38.485329000
1	38.260295000	47.514702000	39.237733000
1	42.181055000	45.812089000	38.758024000
1	39.737375000	45.515216000	39.126898000
6	41.209652000	50.519619000	31.318766000
6	40.606546000	49.297387000	30.623171000
7	40.896797000	48.114738000	31.215949000
8	39.924666000	49.416676000	29.606553000
1	41.742618000	50.270500000	32.240841000
1	40.406058000	51.228325000	31.544982000
1	40.522037000	47.244488000	30.844522000
1	41.452254000	48.072313000	32.065785000
6	49.219882000	40.642911000	31.688714000
6	48.685161000	40.601475000	33.124148000
8	49.197779000	41.304328000	34.010825000
6	48.187361000	40.454184000	30.561591000
6	47.075449000	41.523123000	30.490289000
6	47.620675000	42.895883000	30.067902000
6	45.950320000	41.055310000	29.556424000
1	49.743507000	41.598215000	31.587672000
1	48.728510000	40.441851000	29.603120000
1	47.720304000	39.465092000	30.647541000
1	46.630587000	41.624730000	31.488891000
1	48.393644000	43.265338000	30.752357000
1	48.058894000	42.850705000	29.060620000
1	46.817902000	43.642075000	30.047443000
1	46.331658000	40.856188000	28.544074000
1	45.491495000	40.143349000	29.951943000
1	45.167802000	41.818506000	29.480335000
7	47.688531000	39.730517000	33.361813000
6	46.970051000	39.664123000	34.630568000
6	45.818514000	38.645813000	34.518371000
6	44.930269000	38.949841000	33.287359000
8	45.439598000	38.720317000	32.164302000
8	43.778537000	39.453452000	33.501466000
1	47.173238000	39.296177000	32.589752000
1	46.573823000	40.655069000	34.886784000
1	46.239942000	37.639680000	34.398994000
1	45.235426000	38.672199000	35.443749000
6	50.887755000	44.219541000	34.206765000
6	50.333279000	44.392927000	35.612061000
8	50.661099000	45.372040000	36.305282000
1	51.979693000	44.290562000	34.249629000
1	50.533408000	45.047657000	33.582390000
7	49.482147000	43.438358000	36.029984000
6	48.884437000	43.452223000	37.364271000
6	47.773358000	42.417035000	37.492262000
1	49.284213000	42.650205000	35.413149000
1	48.487196000	44.449450000	37.573476000
1	48.151071000	41.406763000	37.289178000
1	47.373936000	42.453310000	38.510733000
1	46.954121000	42.620684000	36.794333000
6	48.803915000	48.526187000	36.813085000
6	49.445039000	48.436124000	38.196903000
8	49.217009000	49.278656000	39.079051000
6	47.759740000	47.414252000	36.540040000
6	46.727649000	47.266647000	37.670115000
8	47.030127000	46.477261000	38.615023000
8	45.658196000	47.940376000	37.600031000
1	48.337365000	49.511245000	36.737997000
1	47.238505000	47.655588000	35.607672000
1	48.282657000	46.464762000	36.405920000
7	50.267146000	47.377565000	38.384764000
6	50.823110000	47.031594000	39.682919000
6	50.371344000	45.635641000	40.139681000
6	51.039014000	45.237167000	41.452570000
8	48.966776000	45.581639000	40.350868000
1	50.381197000	46.697819000	37.629129000
1	50.488304000	47.790362000	40.394544000
1	50.661850000	44.917422000	39.355529000
1	48.470889000	45.955588000	39.583224000
1	50.783908000	45.954026000	42.241594000
1	50.690630000	44.248274000	41.767832000
1	52.129504000	45.202608000	41.345386000
6	37.740785000	31.881394000	42.304570000
6	37.644463000	31.615516000	40.795416000
8	37.004285000	30.661755000	40.355150000
6	37.690913000	33.353463000	42.738159000
6	37.520216000	33.503595000	44.251489000
8	38.904103000	33.983535000	42.314231000
1	36.920679000	31.322210000	42.763961000
1	36.837196000	33.838224000	42.239015000
1	38.714908000	34.950973000	42.202546000
1	38.347794000	33.013557000	44.778500000
1	37.519950000	34.564885000	44.523530000
1	36.577211000	33.059552000	44.593789000
7	38.338193000	32.493075000	40.018378000
6	38.378533000	32.428737000	38.567779000
6	37.824680000	33.690946000	37.871949000
6	36.323092000	33.881313000	38.158899000
6	38.130775000	33.616005000	36.368427000
6	35.751399000	35.222069000	37.679976000
1	38.822378000	33.240263000	40.512265000
1	37.801914000	31.544588000	38.278704000
1	38.354222000	34.564417000	38.284057000
1	35.764631000	33.049173000	37.704123000
1	36.166074000	33.795672000	39.241487000
1	37.815206000	34.524561000	35.846187000
1	39.205251000	33.488953000	36.187489000
1	37.611758000	32.765681000	35.905233000
1	34.705136000	35.330828000	37.991319000
1	36.317999000	36.062094000	38.098011000
1	35.774758000	35.310839000	36.587327000
6	47.660023000	35.943945000	45.559276000
6	46.350138000	36.546067000	45.047359000
16	46.314125000	36.643488000	43.222725000
1	47.803879000	34.924970000	45.183103000
1	45.484664000	35.967904000	45.388137000
1	46.221524000	37.565881000	45.431064000
6	41.426562000	47.789292000	42.765942000
6	42.935988000	47.972074000	42.909999000
8	43.449545000	48.296497000	43.982893000
6	40.947582000	46.384701000	43.197259000
6	41.291938000	45.951726000	44.636553000
6	40.763389000	46.936620000	45.689730000
6	40.765695000	44.531153000	44.892340000
1	40.954089000	48.556389000	43.387148000
1	39.855105000	46.350035000	43.067736000
1	41.360727000	45.643801000	42.498584000
1	42.385372000	45.931382000	44.729442000
1	41.264240000	47.904716000	45.600091000
1	39.679476000	47.088435000	45.584787000
1	40.947862000	46.557489000	46.703093000
1	41.158014000	43.819349000	44.154868000
1	41.053966000	44.173762000	45.888887000
1	39.668738000	44.498652000	44.833581000
7	43.657915000	47.734618000	41.777626000
6	45.113116000	47.733525000	41.740602000
6	45.734371000	46.324816000	41.741114000
6	45.322102000	45.492976000	42.949935000
8	45.358861000	45.600451000	40.563492000
1	43.174522000	47.395003000	40.956212000
1	45.452596000	48.291243000	42.617567000
1	46.826867000	46.459811000	41.749620000
1	45.858714000	45.997355000	39.801574000
1	45.873631000	44.549430000	42.951832000
1	45.517786000	46.033312000	43.882022000
1	44.252529000	45.263203000	42.902113000
8	42.903033000	38.598199000	41.897741000
26	44.257665000	37.679550000	42.484808000
7	43.332951000	37.313929000	44.235940000
7	45.060248000	39.384940000	43.271878000
7	43.482709000	36.001422000	41.712134000
6	42.032554000	35.335786000	43.571694000
6	44.079843000	39.283705000	45.511186000
6	46.323492000	40.175345000	41.320637000
6	44.664201000	35.972160000	39.568073000
6	42.416539000	36.320480000	44.478482000
6	44.856880000	39.891168000	44.531522000
6	46.112597000	39.068925000	40.506521000
6	43.751988000	35.460678000	40.484772000

6	41.873251000	36.464174000	45.805309000
6	45.539381000	41.159463000	44.674965000
6	46.665785000	38.922693000	39.182916000
6	42.942635000	34.281703000	40.267985000
6	42.460464000	37.566637000	46.354619000
6	46.141740000	41.421285000	43.482568000
6	46.230255000	37.726038000	38.701707000
6	42.178050000	34.119792000	41.382748000
6	43.352736000	38.103290000	45.358462000
6	45.845137000	40.301938000	42.618536000
6	45.389357000	37.147893000	39.721947000
6	42.539656000	35.190114000	42.286595000
7	45.350611000	37.966954000	40.822196000
1	41.263986000	34.633765000	43.880667000
1	43.991149000	39.797465000	46.464277000
1	46.895934000	41.011794000	40.928132000
1	44.787452000	35.433058000	38.633320000
6	43.914785000	42.630835000	35.059161000
6	43.688857000	42.725854000	37.296503000
6	43.650190000	43.007850000	38.674625000
6	43.045788000	40.380557000	37.742523000
6	43.370127000	41.405997000	36.845683000
6	44.037601000	43.494251000	36.129647000
6	43.309019000	41.983029000	39.548521000
6	43.014775000	40.673706000	39.104911000
7	43.501409000	41.386645000	35.477637000
6	44.459112000	44.938305000	36.081152000
6	43.386983000	45.857351000	35.473460000
7	43.831612000	47.246119000	35.460065000
6	43.304154000	48.167861000	34.630618000
8	42.431372000	47.905197000	33.787123000
6	43.856661000	49.576902000	34.781053000
8	43.347723000	42.300984000	40.902504000
6	42.514267000	41.671349000	41.764834000
1	44.091286000	42.797018000	34.003725000
1	43.918401000	43.977471000	39.086093000
1	44.285693000	49.894434000	33.824378000
1	43.035411000	39.557669000	42.072530000
1	41.536857000	41.367624000	41.392561000
1	42.625827000	42.038672000	42.779201000
1	42.868907000	39.366468000	37.391776000
1	42.856576000	39.884216000	39.833395000
1	44.702964000	45.281688000	37.092797000
1	45.380345000	45.047788000	35.492722000
1	42.448018000	45.763162000	36.036136000
1	43.172772000	45.559075000	34.443612000
1	44.613938000	49.659194000	35.565284000
1	43.030995000	50.258420000	35.010564000
1	43.493402000	40.561011000	34.853967000
1	44.487881000	47.547376000	36.187600000
8	47.206547000	43.375643000	40.913156000
1	46.411085000	43.923415000	40.741134000
1	47.933590000	44.016460000	40.768778000
1	48.519314000	36.544381000	45.242764000
1	47.654293000	35.901734000	46.656451000
1	46.425666000	37.276567000	37.736238000
1	47.291729000	39.660711000	38.699818000
1	46.724770000	42.275467000	43.162565000
1	45.529698000	41.765613000	45.572449000
1	42.297018000	38.000389000	47.333448000
1	41.131972000	35.806214000	46.241451000
1	41.427136000	33.371028000	41.596225000
1	42.964678000	33.676479000	39.370149000
1	51.921648000	47.056350000	39.637750000
1	49.585395000	48.460092000	36.045720000
1	45.442102000	48.265986000	40.840508000
1	41.108823000	47.961927000	41.731409000
1	40.946074000	38.230666000	40.805622000
1	39.378598000	38.924322000	36.547022000
1	39.416894000	32.267357000	38.242312000
1	38.680448000	31.448915000	42.677732000
1	40.941563000	42.806402000	29.920770000
1	41.043378000	45.946347000	33.474282000
1	41.897402000	51.019260000	30.627306000
1	42.551933000	50.524273000	40.650066000
1	42.416232000	55.610761000	39.314265000
1	47.534405000	54.899484000	39.193468000
1	49.664777000	43.260445000	38.114144000
1	50.591890000	43.269418000	33.755871000
1	47.662090000	39.380418000	35.432888000
1	49.990489000	39.862162000	31.608662000

^{2}IM1$_{HA,II}$

6	40.002997000	45.098991000	31.392018000
6	40.185432000	44.074193000	30.268873000
8	39.370712000	43.952407000	29.353026000
6	38.528516000	45.384999000	31.721832000
6	38.420430000	46.619514000	32.638913000
6	37.851056000	44.143306000	32.322606000
6	36.988990000	47.114453000	32.879638000
1	40.541594000	44.778472000	32.295009000
1	38.031694000	45.612415000	30.769093000
1	38.897950000	46.398552000	33.604611000
1	39.006706000	47.436529000	32.196519000
1	36.785389000	44.314012000	32.513106000
1	37.931627000	43.293286000	31.637469000
1	38.320332000	43.863711000	33.275767000
1	36.381812000	46.374612000	33.413636000
1	36.988614000	48.031999000	33.480052000
1	36.483584000	47.336271000	31.930482000
7	41.326093000	43.333400000	30.341376000
6	41.728154000	42.384664000	29.315441000
6	42.421165000	41.175173000	29.929734000
8	43.562276000	41.619486000	30.630471000
1	41.978925000	43.471917000	31.101252000
1	42.414132000	42.862704000	28.600336000
1	41.715006000	40.650425000	30.595432000
1	42.688869000	40.477117000	29.117647000
1	43.969363000	40.825749000	31.073265000
6	41.397681000	36.183367000	38.754383000
6	40.073696000	36.952410000	38.700412000
8	39.027276000	36.424063000	39.069905000
6	42.675669000	36.985791000	38.982683000
8	42.709289000	37.633742000	40.258218000
1	41.284273000	35.431891000	39.540838000
1	43.532625000	36.310607000	38.976880000
1	42.839218000	37.717413000	38.179670000
1	41.981998000	38.277160000	40.305710000
7	40.115682000	38.228188000	38.209779000
6	38.883318000	38.948761000	37.888070000
6	39.086215000	40.468467000	37.806033000
6	39.330408000	41.168722000	39.129795000
6	38.350657000	41.157411000	40.134471000
6	40.513050000	41.882163000	39.358523000
6	38.551417000	41.840139000	41.334710000
6	40.720649000	42.574892000	40.556395000
6	39.736794000	42.551378000	41.547359000
1	40.966411000	38.563801000	37.773882000
1	38.152238000	38.685894000	38.655460000
1	39.906928000	40.692213000	37.111768000
1	38.181031000	40.879889000	37.338235000
1	37.417811000	40.620090000	39.972814000
1	41.276661000	41.918707000	38.586374000
1	37.778139000	41.825260000	42.099486000
1	41.639189000	43.138459000	40.702354000
1	39.891578000	43.091398000	42.478005000
6	46.907845000	52.772816000	39.298209000
6	45.743842000	53.738807000	39.541694000
8	45.694990000	54.440805000	40.547111000
6	46.690782000	51.388366000	39.940849000
6	45.774134000	50.463750000	39.133708000
7	45.781889000	49.176024000	39.483404000
8	45.045518000	50.906099000	38.218382000
1	47.104224000	52.654647000	38.227118000
1	47.656198000	50.887033000	40.081462000
1	46.266502000	51.509981000	40.946301000
1	46.446162000	48.764826000	40.144990000
1	45.226461000	48.499452000	38.948854000
7	44.789476000	53.757263000	38.557349000
6	43.509426000	54.412698000	38.755485000
6	42.324794000	53.523483000	39.190660000
8	41.235290000	54.045897000	39.417435000
1	44.839782000	52.982408000	37.902911000
1	43.647482000	55.166183000	39.534014000
7	42.586828000	52.197586000	39.288619000
6	41.609005000	51.201969000	39.694988000
6	41.406810000	50.119579000	38.611803000
6	40.627617000	48.928583000	39.128255000
6	39.254582000	49.031561000	39.398011000
6	41.270128000	47.706807000	39.384462000
6	38.541393000	47.947458000	39.913558000
6	40.559376000	46.620625000	39.905993000
6	39.193003000	46.739131000	40.172813000
1	43.500372000	51.846804000	39.013925000
1	40.677851000	51.730531000	39.911892000
1	40.900663000	50.576076000	37.750901000
1	42.392459000	49.790122000	38.264106000
1	38.738860000	49.968839000	39.196206000
1	42.328426000	47.583925000	39.157372000
1	37.476057000	48.047113000	40.109890000
1	41.086323000	45.694018000	40.111596000
1	38.642305000	45.890000000	40.569880000
6	40.938788000	50.375677000	31.038803000
6	41.708013000	49.526963000	30.030054000
7	42.398760000	48.489833000	30.579863000
8	41.681506000	49.761822000	28.825858000
1	41.170000000	50.120746000	32.077220000
1	39.864887000	50.235977000	30.871806000
1	42.930552000	47.903204000	29.951498000
1	42.492852000	48.335132000	31.584792000
6	49.898827000	41.522341000	31.891008000
6	49.178919000	40.878191000	33.080826000
8	49.484971000	41.189720000	34.244223000
6	49.087771000	41.691274000	30.592839000
6	47.849644000	42.610402000	30.694209000
6	48.242217000	44.085276000	30.870430000

6	46.936539000	42.414262000	29.475149000
1	50.269488000	42.486525000	32.252241000
1	49.762041000	42.089890000	29.819666000
1	48.769542000	40.707879000	30.224532000
1	47.272550000	42.305623000	31.577884000
1	48.861059000	44.249011000	31.760915000
1	48.806302000	44.446400000	29.998781000
1	47.350785000	44.715293000	30.971732000
1	47.463223000	42.667231000	28.543080000
1	46.592925000	41.376573000	29.425062000
1	46.043937000	43.045713000	29.545951000
7	48.259877000	39.940278000	32.791529000
6	47.374924000	39.358659000	33.796565000
6	46.354243000	38.427645000	33.113212000
6	45.654799000	39.136607000	31.926995000
8	46.358430000	39.324474000	30.905000000
8	44.449029000	39.508594000	32.097162000
1	47.918424000	39.810865000	31.833853000
1	46.853960000	40.159714000	34.335945000
1	46.876936000	37.544645000	32.725040000
1	45.622900000	38.098985000	33.858110000
6	50.669664000	43.875470000	35.897162000
6	49.911918000	43.397218000	37.124613000
8	49.960255000	44.034156000	38.191939000
1	50.259205000	44.841885000	35.583135000
1	50.608453000	43.167876000	35.066889000
7	49.203946000	42.262912000	36.976367000
6	48.425330000	41.689022000	38.070686000
6	47.579281000	40.516733000	37.590446000
1	49.199727000	41.795718000	36.068976000
1	47.781173000	42.461934000	38.503806000
1	48.206257000	39.719214000	37.173084000
1	47.005359000	40.110385000	38.427546000
1	46.867959000	40.834719000	36.822550000
6	48.007422000	46.778798000	38.546456000
6	48.031041000	46.515122000	40.046045000
8	47.550559000	47.312198000	40.867064000
6	46.899801000	45.949883000	37.840548000
6	45.552179000	45.972551000	38.589357000
8	45.425817000	45.138181000	39.537803000
8	44.679695000	46.816086000	38.232995000
1	47.832989000	47.846458000	38.397074000
1	46.765319000	46.348889000	36.830262000
1	47.234951000	44.912160000	37.757883000
7	48.566079000	45.322558000	40.397453000
6	48.419305000	44.749212000	41.721349000
6	47.970347000	43.283349000	41.650393000
6	47.968259000	42.642007000	43.036337000
8	46.659937000	43.120439000	41.109358000
1	49.002975000	44.756565000	39.666056000
1	47.690400000	45.354645000	42.267224000
1	48.691506000	42.746056000	41.010995000
1	46.414289000	43.827492000	40.459826000
1	47.301241000	43.191228000	43.710580000
1	47.613974000	41.607995000	42.976033000
1	48.976916000	42.636913000	43.467156000
6	40.248990000	37.451515000	48.174594000
6	39.165782000	36.805495000	47.296330000
8	37.974119000	36.931523000	47.569421000
6	41.435539000	38.085053000	47.438289000
6	42.297753000	38.948645000	48.358677000
8	42.213685000	37.001334000	46.897643000
1	39.736015000	38.207848000	48.774546000
1	41.054172000	38.704933000	46.612903000
1	42.850832000	37.352210000	46.252813000
1	42.668656000	38.354447000	49.201782000
1	43.161766000	39.348975000	47.814799000
1	41.725875000	39.796964000	48.751680000
7	39.632531000	36.084913000	46.238995000
6	38.770410000	35.483631000	45.233007000
6	39.132494000	35.914338000	43.796181000
6	38.958079000	37.432130000	43.601965000
6	38.312242000	35.096358000	42.786561000
6	39.540549000	37.974399000	42.292332000
1	40.639555000	36.070300000	46.105851000
1	37.746329000	35.776246000	45.483877000
1	40.197379000	35.673822000	43.640038000
1	37.886928000	37.676809000	43.661349000
1	39.433422000	37.949830000	44.444742000
1	38.583553000	35.332941000	41.752745000
1	38.463695000	34.019179000	42.934828000
1	37.239063000	35.299011000	42.903739000
1	39.449943000	39.065072000	42.246232000
1	40.605828000	37.719128000	42.207960000
1	39.033108000	37.562127000	41.414994000
6	47.951885000	32.441013000	41.604193000
6	47.340844000	33.099582000	42.842995000
16	47.922135000	34.823486000	43.119791000
1	47.664903000	32.974863000	40.693547000
1	46.250804000	33.095904000	42.785557000
1	47.619043000	32.538084000	43.743540000
6	41.203038000	48.814341000	43.480532000
6	42.523676000	48.092434000	43.742117000

8	43.083186000	48.154253000	44.840977000
6	39.979231000	48.082713000	44.076592000
6	40.008909000	47.800981000	45.592489000
6	40.198983000	49.077350000	46.424803000
6	38.730020000	47.057118000	46.007378000
1	41.294393000	49.812817000	43.920834000
1	39.087575000	48.684668000	43.845202000
1	39.847290000	47.132541000	43.540966000
1	40.867569000	47.149386000	45.797268000
1	41.186391000	49.511069000	46.244472000
1	39.432769000	49.828321000	46.184335000
1	40.121883000	48.857413000	47.497569000
1	38.598632000	46.132103000	45.432079000
1	38.753809000	46.789797000	47.071408000
1	37.839505000	47.679363000	45.841548000
7	43.013963000	47.381828000	42.691743000
6	44.195606000	46.535832000	42.767954000
6	43.860749000	45.062763000	42.461329000
6	42.930949000	44.442749000	43.501301000
8	43.230731000	44.954491000	41.190106000
1	42.469984000	47.312475000	41.840223000
1	44.606327000	46.650947000	43.774572000
1	44.809707000	44.505754000	42.452344000
1	43.936384000	45.048509000	40.498777000
1	42.745816000	43.389662000	43.262705000
1	43.365270000	44.505417000	44.505828000
1	41.967672000	44.964972000	43.511732000
8	44.954979000	37.841025000	42.063005000
26	46.349645000	36.314707000	42.502306000
7	44.840806000	35.346240000	43.428400000
7	46.759063000	37.365121000	44.180809000
7	45.857595000	35.361605000	40.792380000
6	43.970914000	33.999812000	41.572606000
6	45.138657000	36.288881000	45.679085000
6	48.576263000	38.794900000	43.374614000
6	47.571396000	36.341761000	39.325722000
6	43.967393000	34.431583000	42.893438000
6	46.169451000	37.179579000	45.401816000
6	48.599885000	38.340278000	42.061634000
6	46.507956000	35.484755000	39.589467000
6	43.065059000	33.948516000	43.912533000
6	46.762888000	38.063768000	46.381825000
6	49.533524000	38.793699000	41.056222000
6	45.918329000	34.599036000	38.613686000
6	43.411198000	34.575412000	45.071294000
6	47.718992000	38.781463000	45.733210000
6	49.255693000	38.095852000	39.922237000
6	44.901483000	33.941500000	39.241479000
6	44.518873000	35.450811000	44.755755000
6	47.714173000	38.330405000	44.359605000
6	48.148625000	37.219487000	40.233034000
6	44.870662000	34.425707000	40.600761000
7	47.762772000	37.388726000	41.539010000
1	43.231942000	33.258322000	41.283654000
1	44.780044000	36.245009000	46.703398000
1	49.296572000	39.557066000	43.654613000
1	47.980480000	36.328641000	38.319989000
6	44.128788000	42.534124000	33.857330000
6	44.076968000	42.512645000	36.104961000
6	44.081005000	42.745468000	37.488858000
6	43.897090000	40.088624000	36.504234000
6	43.960731000	41.172654000	35.622393000
6	44.197005000	43.369051000	34.951935000
6	44.000666000	41.663790000	38.359910000
6	43.926233000	40.339149000	37.878666000
7	43.973610000	41.222597000	34.245475000
6	44.394217000	44.856606000	34.938137000
6	43.144462000	45.674887000	35.330887000
7	43.459611000	47.085086000	35.523703000
6	43.272064000	48.026176000	34.573623000
8	42.757533000	47.784030000	33.469338000
6	43.718537000	49.434326000	34.939018000
8	43.961095000	42.006025000	39.701872000
6	44.125380000	41.039748000	40.703601000
1	44.182010000	42.766102000	32.801166000
1	44.156632000	43.739938000	37.913105000
1	44.442996000	49.777149000	34.192038000
1	44.228388000	37.614699000	41.443451000
1	43.480249000	40.168839000	40.548202000
1	43.831848000	41.561181000	41.620802000
1	43.865538000	39.066818000	36.131400000
1	43.950609000	39.509389000	38.574131000
1	45.199484000	45.126228000	35.633612000
1	44.720754000	45.182486000	33.942790000
1	42.725409000	45.294866000	36.267666000
1	42.379860000	45.601751000	34.554227000
1	44.162434000	49.522986000	35.935008000
1	42.852944000	50.103161000	34.879355000
1	44.037306000	40.436823000	33.582812000
1	43.933289000	47.307281000	36.400469000
8	45.444027000	40.548259000	40.789141000
1	45.359945000	38.661154000	41.703364000
1	46.009362000	41.348687000	40.913303000

1	49.046330000	32.436964000	41.660468000
1	47.611761000	31.399205000	41.514405000
1	49.737497000	38.160742000	38.955138000
1	50.293131000	39.548623000	41.215076000
1	48.374382000	39.547475000	46.128260000
1	46.469350000	38.115472000	47.423111000
1	42.965024000	34.482999000	46.052845000
1	42.279807000	33.221385000	43.746751000
1	44.227765000	33.197006000	38.835346000
1	46.248295000	34.506967000	37.586355000
1	49.370478000	44.793338000	42.273554000
1	48.974286000	46.513325000	38.104560000
1	44.947205000	46.883174000	42.048253000
1	41.034758000	48.945036000	42.405271000
1	41.495144000	35.639428000	37.803208000
1	38.485379000	38.582772000	36.929951000
1	38.825028000	34.386940000	45.307036000
1	40.643614000	36.695269000	48.868124000
1	40.827239000	42.089297000	28.771260000
1	40.491785000	46.027134000	31.061250000
1	41.163094000	51.431723000	30.859728000
1	41.946538000	50.722068000	40.623515000
1	43.201556000	54.931879000	37.840396000
1	47.783987000	53.237632000	39.759234000
1	49.106849000	41.365785000	38.870319000
1	51.716146000	44.042288000	36.173356000
1	47.966704000	38.807690000	34.537884000
1	50.790936000	40.912048000	31.686633000

^{4}IM1$_{\mathrm{HA,II}}$

6	40.965197000	45.187746000	29.942632000
6	40.990369000	43.960703000	29.032507000
8	40.092293000	43.742730000	28.218637000
6	39.662905000	45.281469000	30.765525000
6	39.577524000	46.647818000	31.474805000
6	39.548252000	44.101298000	31.743506000
6	38.228622000	46.927673000	32.149271000
1	41.834902000	45.207664000	30.613460000
1	38.836493000	45.209993000	30.044306000
1	40.379768000	46.733654000	32.220504000
1	39.768879000	47.438012000	30.734876000
1	38.603598000	44.126416000	32.297398000
1	39.591615000	43.142905000	31.214118000
1	40.365149000	44.115770000	32.477526000
1	38.029087000	46.232349000	32.972329000
1	38.208247000	47.940661000	32.567296000
1	37.400214000	46.843420000	31.433139000
7	42.069982000	43.145504000	29.185420000
6	42.247044000	41.886799000	28.481403000
6	42.601479000	40.775685000	29.465654000
8	43.720003000	41.200836000	30.215074000
1	42.747541000	43.331120000	29.912028000
1	43.054114000	41.982745000	27.740902000
1	41.732144000	40.573986000	30.114709000
1	42.815849000	39.852787000	28.902098000
1	43.986422000	40.447062000	30.810129000
6	41.500571000	36.824151000	38.542090000
6	40.419529000	37.884969000	38.299625000
8	39.240690000	37.638045000	38.547820000
6	42.905146000	37.312071000	38.887439000
8	42.945728000	38.056223000	40.111618000
1	41.122810000	36.181056000	39.341913000
1	43.562806000	36.455641000	39.046508000
1	43.335653000	37.901995000	38.067824000
1	42.359716000	38.828145000	40.027509000
7	40.834696000	39.078297000	37.782701000
6	39.893246000	40.115426000	37.367978000
6	40.140061000	41.471756000	38.043991000
6	40.005067000	41.501864000	39.556249000
6	38.927040000	40.896165000	40.220911000
6	40.941383000	42.212907000	40.320862000
6	38.776141000	41.025018000	41.602936000
6	40.794865000	42.347248000	41.704289000
6	39.702562000	41.761154000	42.347862000
1	41.800730000	39.205930000	37.501327000
1	38.897935000	39.728052000	37.589912000
1	41.133323000	41.840163000	37.762170000
1	39.424085000	42.181347000	37.602686000
1	38.198682000	40.315915000	39.661131000
1	41.792532000	42.681059000	39.834244000
1	37.928900000	40.556454000	42.097900000
1	41.534112000	42.922401000	42.254984000
1	39.568487000	41.882292000	43.419941000
6	46.341195000	52.714918000	39.388806000
6	45.289834000	53.539711000	40.138307000
8	45.553557000	54.103394000	41.196160000
6	46.410952000	51.251164000	39.866527000
6	45.310749000	50.353532000	39.291740000
7	45.478816000	49.040928000	39.472873000
8	44.311388000	50.831210000	38.712927000
1	46.166987000	52.742361000	38.307759000
1	47.383993000	50.816948000	39.606372000
1	46.351801000	51.217091000	40.962320000
1	46.346912000	48.628363000	39.833100000
1	44.801844000	48.381795000	39.081371000
7	44.059176000	53.595132000	39.536768000
6	42.892689000	54.106392000	40.233156000
6	41.905749000	53.064499000	40.800308000
8	40.891826000	53.451442000	41.377995000
1	43.920974000	52.929542000	38.783036000
1	43.256500000	54.702732000	41.073295000
7	42.247594000	51.766478000	40.614716000
6	41.443501000	50.640170000	41.057908000
6	40.962067000	49.778991000	39.867981000
6	40.374110000	48.460246000	40.320152000
6	39.088949000	48.401072000	40.879778000
6	41.121213000	47.274478000	40.233262000
6	38.562906000	47.193932000	41.345740000
6	40.599705000	46.066820000	40.707382000
6	39.319369000	46.022145000	41.265682000
1	43.064766000	51.539788000	40.054375000
1	40.601361000	51.042766000	41.625634000
1	40.229002000	50.357643000	39.291031000
1	41.818363000	49.596875000	39.208570000
1	38.494050000	49.310307000	40.946339000
1	42.111110000	47.276736000	39.778865000
1	37.561798000	47.169279000	41.770361000
1	41.204655000	45.167809000	40.649359000
1	38.917501000	45.078990000	41.626419000
6	39.959080000	50.847956000	33.185593000
6	40.971753000	51.014940000	32.056428000
7	41.953963000	50.077062000	32.035939000
8	40.894528000	51.934614000	31.244663000
1	40.131573000	49.948728000	33.783183000
1	38.953674000	50.811315000	32.753659000
1	42.633499000	50.135834000	31.290426000
1	41.996520000	49.298116000	32.700028000
6	49.916713000	40.176789000	31.402767000
6	49.127948000	39.919383000	32.690840000
8	49.495382000	40.426470000	33.764394000
6	49.107967000	40.232697000	30.092918000
6	48.042484000	41.348174000	30.013083000
6	48.675970000	42.745300000	29.929477000
6	47.086996000	41.093981000	28.838186000
1	50.467104000	41.107746000	31.569022000
1	49.817240000	40.357631000	29.260615000
1	48.618957000	39.266059000	29.918672000
1	47.436988000	41.303400000	30.928164000
1	49.323110000	42.962405000	30.787951000
1	49.282811000	42.845659000	29.018337000
1	47.901564000	43.520765000	29.898273000
1	47.628715000	41.092126000	27.880744000
1	46.579938000	40.133172000	28.968679000
1	46.309485000	41.864452000	28.792867000
7	48.075620000	39.087541000	32.593135000
6	47.131204000	38.870470000	33.685255000
6	45.952454000	38.015700000	33.182222000
6	45.345478000	38.623758000	31.895575000
8	46.030619000	38.502369000	30.851543000
8	44.235557000	39.237180000	32.006535000
1	47.692768000	38.831247000	31.677506000
1	46.765214000	39.837382000	34.055066000
1	46.312284000	37.005340000	32.952676000
1	45.201803000	37.944008000	33.975269000
6	49.740362000	43.664079000	34.668478000
6	49.348471000	43.245944000	36.075522000
8	49.324314000	44.070545000	37.003986000
1	48.899846000	44.194310000	34.205053000
1	49.999575000	42.808148000	34.040492000
7	49.020079000	41.949902000	36.235028000
6	48.583185000	41.416379000	37.520849000
6	48.009809000	40.011951000	37.367307000
1	49.085542000	41.321191000	35.433174000
1	47.831153000	42.086812000	37.950419000
1	48.753506000	39.323646000	36.946450000
1	47.695405000	39.633223000	38.344087000
1	47.133416000	40.018868000	36.712346000
6	47.509831000	46.797694000	37.792664000
6	48.018168000	46.633986000	39.219305000
8	47.799041000	47.471480000	40.108365000
6	46.317394000	45.851150000	37.490118000
6	45.219139000	45.904597000	38.568564000
8	45.435374000	45.243944000	39.627618000
8	44.198531000	46.624324000	38.349942000
1	47.211630000	47.840733000	37.661038000
1	45.895122000	46.132101000	36.520129000
1	46.692252000	44.826588000	37.414816000
7	48.688720000	45.480845000	39.449599000
6	48.959561000	44.987822000	40.786020000
6	48.513357000	43.528882000	40.942894000
6	48.894224000	42.984515000	42.317070000
8	47.104731000	43.359697000	40.789915000
1	48.884602000	44.868946000	38.655377000
1	48.428755000	45.634876000	41.489938000
1	49.030774000	42.936162000	40.169881000
1	46.682498000	44.054884000	40.218633000

1	48.437882000	43.590629000	43.107919000
1	48.536652000	41.956561000	42.428077000
1	49.982827000	42.989225000	42.452447000
6	39.590998000	39.876051000	46.575726000
6	38.359036000	39.029931000	46.225944000
8	37.223161000	39.427614000	46.475863000
6	40.798882000	39.790904000	45.636655000
6	41.864140000	40.832618000	45.975316000
8	41.343249000	38.459880000	45.764828000
1	39.240967000	40.910514000	46.633883000
1	40.461814000	39.939937000	44.600819000
1	42.012611000	38.335382000	45.071081000
1	42.192392000	40.720722000	47.015203000
1	42.740676000	40.719143000	45.326803000
1	41.478473000	41.849573000	45.840693000
7	38.621076000	37.818686000	45.661107000
6	37.577330000	36.854535000	45.355491000
6	37.942286000	35.947586000	44.166386000
6	38.043023000	36.738466000	42.846496000
6	36.932977000	34.792335000	44.078680000
6	38.687460000	35.964149000	41.690517000
1	39.598877000	37.559238000	45.565906000
1	36.661659000	37.419019000	45.150593000
1	38.934141000	35.511011000	44.374074000
1	37.034252000	37.068253000	42.556206000
1	38.620526000	37.653561000	43.027161000
1	37.182278000	34.100624000	43.267744000
1	36.903880000	34.216088000	45.011843000
1	35.920662000	35.174106000	43.890877000
1	38.755448000	36.578694000	40.786597000
1	39.704604000	35.646660000	41.958031000
1	38.115625000	35.064777000	41.430592000
6	47.912331000	33.130731000	42.313368000
6	47.104994000	33.832243000	43.407776000
16	47.637433000	35.562147000	43.729534000
1	47.780855000	33.627729000	41.348146000
1	46.038805000	33.820073000	43.169072000
1	47.225410000	33.304723000	44.362383000
6	41.317072000	47.869790000	44.506097000
6	42.793501000	47.494622000	44.395691000
8	43.562548000	47.620323000	45.352522000
6	40.460229000	46.769609000	45.172711000
6	40.899547000	46.305673000	46.576216000
6	40.982890000	47.463856000	47.581097000
6	39.952628000	45.204923000	47.079254000
1	41.268673000	48.791607000	45.094491000
1	39.426118000	47.141670000	45.229258000
1	40.429600000	45.898245000	44.504109000
1	41.906816000	45.878418000	46.489635000
1	41.775464000	48.161972000	47.298121000
1	40.031091000	48.011373000	47.638520000
1	41.210363000	47.089223000	48.587446000
1	39.904829000	44.363067000	46.376456000
1	40.278841000	44.812830000	48.050528000
1	38.930449000	45.588971000	47.202881000
7	43.181605000	47.007070000	43.184796000
6	44.514654000	46.483497000	42.924738000
6	44.540765000	44.950513000	42.771164000
6	44.065495000	44.221469000	44.024109000
8	43.716101000	44.537957000	41.683466000
1	42.480880000	46.845329000	42.471655000
1	45.147471000	46.799943000	43.757977000
1	45.584037000	44.666543000	42.567029000
1	44.186574000	44.802683000	40.853044000
1	44.166061000	43.138106000	43.894594000
1	44.646479000	44.527116000	44.901101000
1	43.011313000	44.447978000	44.219673000
8	45.147292000	38.539410000	41.816921000
26	46.310958000	37.063147000	42.641742000
7	44.607176000	36.242607000	43.373871000
7	46.499697000	38.253916000	44.276314000
7	46.087311000	35.922172000	40.972232000
6	44.059872000	34.660980000	41.589183000
6	44.477403000	37.508787000	45.475751000
6	48.603939000	39.399862000	43.726889000
6	48.047723000	36.760708000	39.721176000
6	43.825219000	35.261527000	42.817088000
6	45.635718000	38.297454000	45.348712000
6	48.803165000	38.850071000	42.471091000
6	46.906955000	35.936089000	39.864098000
6	42.715883000	34.945566000	43.691952000
6	46.095838000	39.225282000	46.323412000
6	49.923926000	39.156154000	41.604641000
6	46.464622000	34.994237000	38.902195000
6	42.831052000	35.753748000	44.779746000
6	47.276257000	39.751497000	45.838844000
6	49.754056000	38.428092000	40.471430000
6	45.340814000	34.381409000	39.437669000
6	44.012751000	36.564665000	44.578558000
6	47.501334000	39.136068000	44.575294000
6	48.536652000	37.662585000	40.642015000
6	45.127552000	34.975538000	40.709095000
7	47.966621000	37.946913000	41.866195000
1	43.360266000	33.896689000	41.263402000
1	43.889756000	37.654340000	46.378144000
1	49.345300000	40.106422000	44.087238000
1	48.605036000	36.655000000	38.794543000
6	43.477969000	42.365898000	33.527045000
6	43.730814000	42.521321000	35.753003000
6	43.894436000	42.873399000	37.099730000
6	44.081295000	40.153783000	36.319676000
6	43.808614000	41.147488000	35.373011000
6	43.522355000	43.286845000	34.551501000
6	44.158133000	41.881114000	38.039964000
6	44.268276000	40.526290000	37.657932000
7	43.625839000	41.083506000	34.008486000
6	43.519266000	44.787637000	34.473960000
6	42.407503000	45.474959000	35.309921000
7	42.786123000	46.804771000	35.774925000
6	42.570917000	47.917616000	35.050879000
8	42.008611000	47.898624000	33.938335000
6	43.034285000	49.222589000	35.673990000
8	44.287182000	42.345172000	39.331357000
6	44.481141000	41.469309000	40.410703000
1	43.380047000	42.510514000	32.458500000
1	43.832043000	43.900199000	37.442553000
1	43.668106000	49.754758000	34.957038000
1	44.450766000	38.247123000	41.186356000
1	43.790823000	40.620633000	40.363736000
1	44.271960000	42.091611000	41.283736000
1	44.197299000	39.114110000	36.020233000
1	44.556845000	39.780671000	38.388855000
1	44.483914000	45.160204000	34.848021000
1	43.446102000	45.125007000	33.433542000
1	42.180985000	44.879397000	36.197447000
1	41.490273000	45.562744000	34.722399000
1	43.576172000	49.096952000	36.613900000
1	42.160413000	49.856204000	35.861237000
1	43.778650000	40.269132000	33.393806000
1	43.295051000	46.860027000	36.665498000
8	45.783565000	40.925271000	40.452982000
1	45.602554000	39.313408000	41.405878000
1	46.387705000	41.696172000	40.604661000
1	48.981727000	33.133468000	42.553063000
1	47.588974000	32.085017000	42.208505000
1	50.382514000	38.393504000	39.590488000
1	50.718869000	39.848694000	41.850200000
1	47.918641000	40.494770000	46.293430000
1	45.592918000	39.449627000	47.255606000
1	42.188633000	35.814897000	45.648971000
1	41.957462000	34.202312000	43.480115000
1	44.726446000	33.604634000	38.999676000
1	46.935994000	34.811881000	37.944651000
1	50.033623000	45.049827000	41.017802000
1	48.314905000	46.568302000	37.085458000
1	44.904612000	46.930519000	42.002862000
1	40.892555000	48.087151000	43.519277000
1	41.559341000	36.205936000	37.634460000
1	39.964030000	40.246091000	36.280486000
1	37.369791000	36.229726000	46.239385000
1	39.921983000	39.593753000	47.585679000
1	41.315797000	41.673041000	27.950765000
1	41.041196000	46.069642000	29.292905000
1	40.001183000	51.729482000	33.834795000
1	42.040724000	50.015432000	41.735128000
1	42.313271000	54.770158000	39.580907000
1	47.302140000	53.195083000	39.594508000
1	49.430719000	41.405171000	38.222005000
1	50.579918000	44.362296000	34.729265000
1	47.638422000	38.378635000	34.523952000
1	50.675881000	39.384477000	31.326227000

$^{2}RE_{II,SH}$

6	43.273741000	47.689290000	31.720244000
6	44.076487000	46.685430000	30.891075000
8	43.816825000	46.485286000	29.701573000
6	41.753827000	47.522205000	31.528131000
6	41.001196000	48.728539000	32.122739000
6	41.270862000	46.185151000	32.111514000
6	39.495273000	48.752234000	31.832876000
1	43.545007000	47.633110000	32.781170000
1	41.579827000	47.504488000	30.443446000
1	41.160004000	48.753772000	33.210964000
1	41.450596000	49.650700000	31.727279000
1	40.209531000	46.009791000	31.902090000
1	41.831228000	45.346520000	31.684793000
1	41.402468000	46.160981000	33.201593000
1	38.975298000	47.912423000	32.306868000
1	39.038368000	49.675695000	32.209101000
1	39.300160000	48.699499000	30.753698000
7	45.091398000	46.056881000	31.541061000
6	46.003254000	45.173316000	30.824951000
6	45.523112000	43.729039000	30.686015000
8	45.554277000	43.087045000	31.945487000
1	45.296251000	46.305463000	32.506624000
1	46.963136000	45.180752000	31.352419000

1	44.512603000	43.734030000	30.249776000
1	46.190169000	43.219923000	29.969588000
1	45.275038000	42.144482000	31.817874000
6	36.161207000	38.936398000	34.888074000
6	35.063075000	39.543278000	35.746725000
8	34.784285000	39.065858000	36.851623000
6	37.479786000	38.859144000	35.686327000
8	37.379671000	37.993780000	36.801711000
1	35.857686000	37.918048000	34.617694000
1	38.269085000	38.460328000	35.041374000
1	37.783590000	39.872506000	35.989205000
1	36.552997000	38.239742000	37.261051000
7	34.422246000	40.634387000	35.238341000
6	33.500273000	41.442764000	36.024675000
6	34.100001000	42.799630000	36.458544000
6	35.361426000	42.662227000	37.284034000
6	35.296363000	42.289210000	38.633702000
6	36.622873000	42.851990000	36.705138000
6	36.459098000	42.096744000	39.379644000
6	37.791666000	42.655429000	37.446440000
6	37.712348000	42.272103000	38.787237000
1	34.776379000	41.025517000	34.376229000
1	33.243586000	40.840615000	36.898602000
1	34.304140000	43.404251000	35.564293000
1	33.324234000	43.334625000	37.023002000
1	34.325652000	42.139436000	39.102347000
1	36.694286000	43.157686000	35.662908000
1	36.387650000	41.803578000	40.423668000
1	38.762307000	42.796395000	36.979248000
1	38.621774000	42.097729000	39.353271000
6	45.948056000	53.532894000	38.172835000
6	44.639060000	54.295038000	37.956978000
8	44.233390000	55.109910000	38.780615000
6	45.802317000	52.408996000	39.211483000
6	45.071220000	51.174340000	38.670543000
7	45.053962000	50.110889000	39.477403000
8	44.538350000	51.186052000	37.542344000
1	46.335498000	53.123006000	37.234998000
1	46.792102000	52.100039000	39.570289000
1	45.261083000	52.790531000	40.086999000
1	45.440059000	50.111175000	40.420037000
1	44.539245000	49.252006000	39.254834000
7	43.979719000	54.009097000	36.790402000
6	42.638902000	54.496592000	36.524883000
6	41.488295000	53.478314000	36.649601000
8	40.359200000	53.809716000	36.292654000
1	44.296288000	53.182991000	36.297870000
1	42.448846000	55.303446000	37.237546000
7	41.817453000	52.270304000	37.166723000
6	40.870958000	51.176256000	37.319959000
6	41.326354000	49.927018000	36.535499000
6	40.460915000	48.718319000	36.816870000
6	39.255770000	48.520860000	36.127613000
6	40.831967000	47.785425000	37.797106000
6	38.441412000	47.421550000	36.405619000
6	40.018090000	46.684402000	38.075209000
6	38.819621000	46.497603000	37.382025000
1	42.788971000	52.061531000	37.385248000
1	39.899794000	51.534840000	36.970808000
1	41.315717000	50.166638000	35.464537000
1	42.367135000	49.713445000	36.804712000
1	38.957473000	49.233955000	35.361348000
1	41.763980000	47.916483000	38.343726000
1	37.513022000	47.284250000	35.855759000
1	40.324329000	45.968131000	38.833822000
1	38.191952000	45.636824000	37.596529000
6	46.767560000	50.492946000	32.641576000
6	48.151197000	49.850616000	32.628961000
7	48.201640000	48.604360000	33.169526000
8	49.132463000	50.427187000	32.166176000
1	46.008798000	49.866118000	33.117561000
1	46.467373000	50.700858000	31.608962000
1	49.089807000	48.123029000	33.143882000
1	47.373983000	48.117681000	33.523003000
6	49.255639000	38.847668000	34.753009000
6	47.793531000	38.487477000	35.022693000
8	47.400837000	38.267550000	36.187910000
6	49.515587000	39.848683000	33.610833000
6	48.877282000	41.243432000	33.788754000
6	49.529091000	42.035832000	34.932145000
6	48.925727000	42.024399000	32.468026000
1	49.656072000	39.217132000	35.701697000
1	50.604413000	39.965922000	33.504824000
1	49.171489000	39.422614000	32.660034000
1	47.815436000	41.102869000	34.031927000
1	49.458159000	41.516065000	35.895396000
1	50.593991000	42.214445000	34.726652000
1	49.046182000	43.012598000	35.051048000
1	49.963330000	42.179774000	32.138071000
1	48.377661000	41.482459000	31.691218000
1	48.447084000	43.002970000	32.579241000
7	46.992626000	38.379813000	33.951757000
6	45.550258000	38.171425000	34.052248000
6	44.914227000	38.278896000	32.653702000
6	45.347054000	39.589762000	31.952866000
8	46.539807000	39.635239000	31.564459000
8	44.487843000	40.524156000	31.871429000
1	47.278473000	38.752752000	33.038759000
1	45.114075000	38.921678000	34.724649000
1	45.246589000	37.431901000	32.041279000
1	43.825604000	38.224164000	32.754657000
6	45.866906000	41.033291000	37.838676000
6	44.931655000	39.990274000	38.413763000
8	44.118952000	40.298414000	39.312483000
1	46.311995000	41.605033000	38.657760000
1	45.283365000	41.729921000	37.226426000
7	45.001957000	38.759479000	37.884298000
6	44.158230000	37.658162000	38.345383000
6	43.970407000	36.596931000	37.266881000
1	45.753570000	38.554219000	37.223095000
1	43.189975000	38.074737000	38.623769000
1	44.927853000	36.163880000	36.956535000
1	43.345836000	35.791502000	37.666112000
1	43.475947000	37.018462000	36.384489000
6	44.299124000	44.030676000	40.913546000
6	43.777982000	43.163251000	42.061283000
8	43.555847000	43.630289000	43.183526000
6	43.628585000	45.405527000	40.929172000
6	44.104750000	46.402595000	39.863475000
8	43.820212000	47.634333000	40.099904000
8	44.693673000	45.967837000	38.851199000
1	45.382699000	44.154895000	41.044277000
1	42.544339000	45.285885000	40.788402000
1	43.746547000	45.850943000	41.920353000
7	43.589128000	41.851942000	41.765569000
6	43.078577000	40.919247000	42.761148000
6	43.352654000	39.455831000	42.396834000
6	42.966666000	38.520609000	43.538012000
8	42.591429000	39.068044000	41.244075000
1	43.766032000	41.516824000	40.821157000
1	41.993626000	41.047377000	42.888356000
1	44.424243000	39.335079000	42.180237000
1	43.045527000	39.429126000	40.447754000
1	41.897837000	38.614575000	43.765155000
1	43.175077000	37.484732000	43.256062000
1	43.538584000	38.751739000	44.443096000
6	38.963931000	33.215160000	42.430503000
6	37.661446000	33.314305000	41.627063000
8	36.605340000	32.862474000	42.072089000
6	39.978216000	34.358295000	42.283872000
6	41.134296000	34.210704000	43.277743000
8	40.459478000	34.343828000	40.943170000
1	38.667110000	33.107928000	43.478136000
1	39.461947000	35.313379000	42.486771000
1	41.081678000	35.108054000	40.830348000
1	41.638098000	33.246631000	43.139973000
1	41.872023000	35.002266000	43.113528000
1	40.778340000	34.276287000	44.313815000
7	37.782905000	33.890234000	40.398937000
6	36.679409000	33.995899000	39.461255000
6	36.269774000	35.443954000	39.115207000
6	35.771798000	36.198520000	40.362293000
6	35.225164000	35.416016000	37.988013000
6	35.608949000	37.711625000	40.170419000
1	38.719902000	34.200281000	40.140874000
1	35.837259000	33.462038000	39.912805000
1	37.161112000	35.963414000	38.731308000
1	34.815877000	35.759132000	40.684789000
1	36.478742000	36.018931000	41.181347000
1	34.954875000	36.421458000	37.651927000
1	35.606428000	34.871367000	37.115014000
1	34.307100000	34.912163000	38.321402000
1	35.281592000	38.189315000	41.102210000
1	36.560898000	38.175684000	39.882169000
1	34.876084000	37.961776000	39.395852000
16	48.742564000	34.318213000	42.277284000
6	40.353004000	47.253628000	42.467979000
6	41.194150000	48.357316000	43.101080000
8	40.864169000	48.883929000	44.168105000
6	40.494136000	45.874921000	43.154763000
6	40.193737000	45.802921000	44.665210000
6	38.790701000	46.315233000	45.020139000
6	40.403420000	44.359530000	45.151539000
1	39.311955000	47.590936000	42.520493000
1	39.818884000	45.180967000	42.630557000
1	41.507582000	45.490587000	42.993014000
1	40.916910000	46.447333000	45.181299000
1	38.701830000	47.384520000	44.806884000
1	38.016134000	45.776132000	44.455616000
1	38.581818000	46.168611000	46.088040000
1	41.394523000	43.984849000	44.870868000
1	40.301333000	44.291104000	46.242537000
1	39.656055000	43.686195000	44.707986000
7	42.315153000	48.718376000	42.412715000
6	43.199064000	49.766760000	42.894338000
6	44.620682000	49.288023000	43.240398000

6	44.644615000	48.154726000	44.267731000
8	45.370052000	48.942250000	42.071770000
1	42.576838000	48.244622000	41.545029000
1	42.721560000	50.188637000	43.782618000
1	45.148637000	50.155608000	43.660446000
1	44.839820000	48.321797000	41.517082000
1	45.679985000	47.885740000	44.501006000
1	44.137169000	48.453452000	45.192799000
1	44.139353000	47.262531000	43.883591000
8	44.918212000	35.792449000	41.549451000
26	46.402495000	35.191912000	41.906248000
7	45.653796000	33.534506000	42.783157000
7	46.477810000	36.112954000	43.702809000
7	46.555631000	34.189423000	40.157559000
6	45.550784000	32.057894000	40.830819000
6	45.449219000	34.364662000	45.079877000
6	47.460978000	38.254025000	43.020036000
6	47.519807000	35.963447000	38.761959000
6	45.330827000	32.355737000	42.171835000
6	45.999789000	35.625523000	44.891025000
6	47.663694000	37.962370000	41.679805000
6	47.009410000	34.686485000	38.964532000
6	44.738063000	31.444619000	43.122030000
6	46.141493000	36.617585000	45.929749000
6	48.211352000	38.886185000	40.711539000
6	46.869458000	33.693225000	37.925356000
6	44.711034000	32.093837000	44.318764000
6	46.704219000	37.713674000	45.349473000
6	48.215837000	38.243906000	39.513268000
6	46.320789000	32.592177000	38.506667000
6	45.287167000	33.398452000	44.093688000
6	46.906065000	37.386635000	43.957816000
6	47.681486000	36.922087000	39.751636000
6	46.119765000	32.914856000	39.899595000
7	47.358742000	36.767157000	41.075553000
1	45.243906000	31.076297000	40.482187000
1	45.110164000	34.115099000	46.081019000
1	47.748607000	39.242707000	43.364778000
1	47.796498000	36.250509000	37.752297000
6	43.459911000	42.643062000	34.263964000
6	42.322168000	41.942575000	36.085677000
6	41.707690000	41.713460000	37.317605000
6	41.676748000	39.690179000	35.326724000
6	42.302618000	40.914503000	35.093764000
6	43.074672000	43.047938000	35.531587000
6	41.087104000	40.470680000	37.557438000
6	41.065920000	39.469812000	36.565842000
7	42.993288000	41.384007000	33.997319000
6	43.421817000	44.346564000	36.199465000
6	44.350583000	45.201100000	35.330343000
7	44.870883000	46.343876000	36.069047000
6	45.619308000	47.276480000	35.468937000
8	45.833202000	47.275959000	34.232423000
6	46.186005000	48.357261000	36.366264000
8	40.533348000	40.331663000	38.793142000
6	39.954601000	39.077395000	39.152087000
1	44.069918000	43.141776000	33.518864000
1	41.721160000	42.448404000	38.116324000
1	47.195682000	48.609398000	36.029347000
1	40.696215000	38.270996000	39.110947000
1	39.103185000	38.829611000	38.506918000
1	39.625578000	39.195403000	40.185045000
1	41.662019000	38.914327000	34.565550000
1	40.578558000	38.521568000	36.757404000
1	42.513459000	44.920413000	36.437426000
1	43.907143000	44.160708000	37.167850000
1	43.819177000	45.554555000	34.440126000
1	45.189721000	44.593297000	34.966614000
1	46.208976000	48.044279000	37.413425000
1	45.571590000	49.262536000	36.298118000
1	43.329502000	40.864949000	33.164958000
1	44.788246000	46.344480000	37.096527000
8	42.285328000	36.343370000	40.677803000
1	43.181376000	36.023798000	40.913383000
1	42.294201000	37.291601000	40.945181000
1	48.510052000	38.602469000	38.535799000
1	48.527993000	39.896414000	40.938660000
1	46.961553000	38.662633000	45.802877000
1	45.839492000	36.478686000	46.960273000
1	44.339267000	31.740035000	45.272195000
1	44.393338000	30.445708000	42.885974000
1	46.062265000	31.644496000	38.051122000
1	47.159643000	33.841385000	36.893008000
1	43.544618000	41.164747000	43.721711000
1	44.159408000	43.537178000	39.946706000
1	43.278114000	50.556830000	42.135022000
1	40.600676000	47.144397000	41.404942000
1	36.318242000	39.502657000	33.961428000
1	32.583387000	41.612360000	35.445103000
1	36.947250000	33.471482000	38.531949000
1	39.466993000	32.281533000	42.139127000
1	46.151182000	45.584840000	29.820449000
1	43.570441000	48.688953000	31.371635000
1	46.827941000	51.453993000	33.162782000
1	40.772278000	50.921251000	38.383588000
1	42.572616000	54.920716000	35.516680000
1	46.668491000	54.265680000	38.550088000
1	44.587302000	37.209520000	39.248679000
1	46.654740000	40.594174000	37.222164000
1	45.347778000	37.189982000	34.497034000
1	49.787555000	37.908575000	34.541311000
1	49.204462000	35.362303000	43.002220000

$^{4}RE_{II,SH}$

6	43.271140000	47.687145000	31.725283000
6	44.073598000	46.684278000	30.894613000
8	43.813413000	46.485492000	29.704981000
6	41.751105000	47.519570000	31.534339000
6	40.998653000	48.725446000	32.130080000
6	41.268988000	46.182169000	32.117553000
6	39.492524000	48.749028000	31.841258000
1	43.543306000	47.630345000	32.785939000
1	41.576252000	47.502252000	30.449773000
1	41.158177000	48.750035000	33.218210000
1	41.447594000	49.647889000	31.734772000
1	40.207457000	46.006764000	31.909196000
1	41.829032000	45.343837000	31.689841000
1	41.401689000	46.157527000	33.207491000
1	38.973041000	47.908925000	32.315264000
1	39.035771000	49.672245000	32.218277000
1	39.296592000	48.696839000	30.762197000
7	45.088620000	46.054900000	31.543522000
6	45.999662000	45.171426000	30.826336000
6	45.519172000	43.727385000	30.686839000
8	45.551052000	43.084708000	31.945998000
1	45.293619000	46.302030000	32.509535000
1	46.960020000	45.178517000	31.352982000
1	44.508307000	43.732770000	30.251404000
1	46.185681000	43.218754000	29.969574000
1	45.271429000	42.142233000	31.818291000
6	36.169150000	38.957426000	34.880900000
6	35.070141000	39.560445000	35.741229000
8	34.788963000	39.076964000	36.842849000
6	37.486025000	38.872797000	35.681236000
8	37.382327000	37.999933000	36.790391000
1	35.864539000	37.941299000	34.603515000
1	38.276023000	38.477193000	35.035186000
1	37.790806000	39.883630000	35.991597000
1	36.555230000	38.244395000	37.249731000
7	34.431075000	40.654961000	35.238048000
6	33.508184000	41.459517000	36.027145000
6	34.108204000	42.813256000	36.469874000
6	35.367816000	42.669973000	37.297071000
6	35.299535000	42.291266000	38.644989000
6	36.630698000	42.859860000	36.721323000
6	36.460547000	42.093555000	39.392299000
6	37.797711000	42.658076000	37.463997000
6	37.715197000	42.269153000	38.802976000
1	34.787336000	41.050873000	34.378993000
1	33.249383000	40.852595000	36.897144000
1	34.314618000	43.422747000	35.579469000
1	33.331682000	43.345520000	37.035847000
1	34.327679000	42.141364000	39.111219000
1	36.704646000	43.169870000	35.680543000
1	36.386605000	41.796224000	40.434974000
1	38.769425000	42.799288000	36.999130000
1	38.623288000	42.091099000	39.369981000
6	45.955053000	53.527691000	38.179623000
6	44.646424000	54.291132000	37.965935000
8	44.241647000	55.104629000	38.791358000
6	45.809519000	52.402675000	39.217101000
6	45.075996000	51.169644000	38.675747000
7	45.056573000	50.105967000	39.482231000
8	44.543239000	51.182757000	37.547492000
1	46.341150000	53.118465000	37.240952000
1	46.799499000	52.091867000	39.573794000
1	45.270310000	52.783769000	40.094047000
1	45.442939000	50.104964000	40.424738000
1	44.541145000	49.247697000	39.258869000
7	43.986302000	54.007973000	36.799112000
6	42.645846000	54.497209000	36.534956000
6	41.494220000	53.479900000	36.658506000
8	40.365082000	53.813301000	36.303561000
1	44.302117000	53.182696000	36.304749000
1	42.456793000	55.302994000	37.249088000
7	41.822654000	52.270310000	37.172326000
6	40.875025000	51.177174000	37.324871000
6	41.329303000	49.928090000	36.539597000
6	40.462959000	48.719906000	36.820428000
6	39.257956000	48.523329000	36.130671000
6	40.832880000	47.786734000	37.800837000
6	38.442805000	47.424480000	36.408189000
6	40.018138000	46.686232000	38.078530000
6	38.819931000	46.500213000	37.384704000
1	42.794006000	52.060312000	37.390447000
1	39.904097000	51.536806000	36.976063000

1	41.318753000	50.168410000	35.468786000
1	42.369944000	49.713537000	36.808556000
1	38.960483000	49.236659000	35.364314000
1	41.764622000	47.917246000	38.348057000
1	37.514594000	47.287848000	35.857845000
1	40.323391000	45.969865000	38.837444000
1	38.191560000	45.639882000	37.598964000
6	46.772539000	50.485506000	32.644187000
6	48.154808000	49.840287000	32.630205000
7	48.202908000	48.593395000	33.169542000
8	49.136993000	50.415124000	32.167237000
1	46.013059000	49.860484000	33.121411000
1	46.471506000	50.693342000	31.611803000
1	49.090053000	48.110234000	33.143113000
1	47.374635000	48.108658000	33.524143000
6	49.257737000	38.854532000	34.749404000
6	47.796234000	38.494231000	35.022165000
8	47.405129000	38.277923000	36.188448000
6	49.515527000	39.852768000	33.604302000
6	48.876324000	41.247469000	33.779170000
6	49.528262000	42.043127000	34.920231000
6	48.923429000	42.025165000	32.456503000
1	49.659359000	39.226642000	35.696546000
1	50.604162000	39.970558000	33.496774000
1	49.170660000	39.423872000	32.655057000
1	47.814715000	41.106859000	34.023324000
1	49.458069000	41.525685000	35.884782000
1	50.592966000	42.221875000	34.713809000
1	49.044823000	43.019891000	35.036974000
1	49.960714000	42.180566000	32.125581000
1	48.375386000	41.480822000	31.681349000
1	48.443947000	43.003544000	32.565650000
7	46.994180000	38.382377000	33.952410000
6	45.552068000	38.173771000	34.055511000
6	44.913708000	38.277868000	32.657763000
6	45.344512000	39.587416000	31.953290000
8	46.536180000	39.632626000	31.561738000
8	44.484881000	40.521626000	31.872651000
1	47.278724000	38.753055000	33.038131000
1	45.116758000	38.925317000	34.727001000
1	45.245375000	37.429732000	32.046586000
1	43.825280000	38.222707000	32.760787000
6	45.867989000	41.030475000	37.846804000
6	44.932953000	39.986644000	38.420826000
8	44.118144000	40.294509000	39.317823000
1	46.313312000	41.601049000	38.666599000
1	45.284176000	41.727968000	37.235846000
7	45.005773000	38.755527000	37.892509000
6	44.162628000	37.653508000	38.352773000
6	43.974699000	36.593330000	37.273296000
1	45.757350000	38.551784000	37.230683000
1	43.194462000	38.069564000	38.632182000
1	44.932110000	36.160454000	36.962555000
1	43.350186000	35.787492000	37.671809000
1	43.480141000	37.015761000	36.391371000
6	44.295709000	44.025884000	40.916115000
6	43.773133000	43.159256000	42.063816000
8	43.548837000	43.627161000	43.185255000
6	43.625398000	45.400843000	40.930114000
6	44.104438000	46.398121000	39.865901000
8	43.820714000	47.629965000	40.102916000
8	44.694581000	45.963603000	38.854226000
1	45.379161000	44.149961000	41.047990000
1	42.541486000	45.281517000	40.786603000
1	43.741013000	45.845989000	41.921670000
7	43.585540000	41.847539000	41.769029000
6	43.073734000	40.915575000	42.764653000
6	43.347247000	39.451870000	42.401055000
6	42.959000000	38.517342000	43.542070000
8	42.587665000	39.064346000	41.247114000
1	43.764085000	41.511545000	40.825218000
1	41.988761000	41.044415000	42.890821000
1	44.419079000	39.330343000	42.186023000
1	43.043204000	39.425567000	40.451615000
1	41.890094000	38.612680000	43.768262000
1	43.166250000	37.481087000	43.260619000
1	43.530434000	38.747851000	44.447603000
6	38.947330000	33.217091000	42.421623000
6	37.646316000	33.320446000	41.616349000
8	36.588372000	32.870905000	42.059322000
6	39.964112000	34.358535000	42.278853000
6	41.118161000	34.206539000	43.274429000
8	40.447637000	34.345976000	40.938979000
1	38.648569000	33.108482000	43.468559000
1	39.449293000	35.314165000	42.483071000
1	41.073911000	35.107357000	40.829532000
1	41.620642000	33.241979000	43.135117000
1	41.857493000	34.997283000	43.113565000
1	40.760417000	34.270097000	44.309989000
7	37.771070000	33.896856000	40.388781000
6	36.669145000	34.005582000	39.449626000
6	36.262099000	35.454675000	39.104997000
6	35.763635000	36.208613000	40.352255000
6	35.218850000	35.429626000	37.976489000
6	35.603993000	37.722253000	40.161762000
1	38.708981000	34.205558000	40.132536000
1	35.825561000	33.472328000	39.899202000
1	37.154820000	35.973153000	38.722905000
1	34.806359000	35.770704000	40.672715000
1	36.468912000	36.026710000	41.172229000
1	34.949859000	36.435896000	37.641852000
1	35.600567000	34.886151000	37.102970000
1	34.299953000	34.926097000	38.308043000
1	35.275937000	38.199563000	41.093505000
1	36.557298000	38.184861000	39.875635000
1	34.873030000	37.974690000	39.386146000
16	48.731123000	34.316497000	42.285369000
6	40.351451000	47.252914000	42.466786000
6	41.193768000	48.354354000	43.102274000
8	40.863163000	48.880439000	44.169364000
6	40.489385000	45.872987000	43.151838000
6	40.187111000	45.799419000	44.661844000
6	38.784548000	46.313665000	45.015917000
6	40.393714000	44.354984000	45.146337000
1	39.310932000	47.591931000	42.518546000
1	39.813418000	45.181016000	42.625951000
1	41.502281000	45.486981000	42.990667000
1	40.910848000	46.441795000	45.179697000
1	38.697814000	47.383413000	44.804149000
1	38.009697000	45.776767000	44.449678000
1	38.574137000	46.165821000	46.083349000
1	41.384360000	43.978869000	44.865991000
1	40.290511000	44.285234000	46.237147000
1	39.645516000	43.683645000	44.701170000
7	42.316726000	48.713627000	42.416174000
6	43.201850000	49.759954000	42.900032000
6	44.622935000	49.278860000	43.245033000
6	44.645788000	48.143369000	44.269966000
8	45.371887000	48.934939000	42.075606000
1	42.578401000	48.240623000	41.548044000
1	42.724777000	50.180580000	43.789137000
1	45.151850000	50.144964000	43.666916000
1	44.841583000	48.315112000	41.520284000
1	45.680904000	47.872749000	44.502478000
1	44.138947000	48.440770000	45.195800000
1	44.139409000	47.252529000	43.884167000
8	44.918715000	35.786150000	41.544290000
26	46.410358000	35.195114000	41.892911000
7	45.652459000	33.535193000	42.764380000
7	46.473005000	36.114092000	43.692874000
7	46.567968000	34.195672000	40.145349000
6	45.558650000	32.062511000	40.809563000
6	45.437374000	34.362518000	45.061185000
6	47.460655000	38.254948000	43.019336000
6	47.540931000	35.970622000	38.757697000
6	45.329140000	32.358716000	42.149350000
6	45.989544000	35.623440000	44.877528000
6	47.670295000	37.964573000	41.679916000
6	47.030673000	34.693026000	38.955845000
6	44.726720000	31.448289000	43.094413000
6	46.126266000	36.613173000	45.919439000
6	48.226038000	38.888696000	40.716671000
6	46.899351000	33.699948000	37.915422000
6	44.694895000	32.095674000	44.291910000
6	46.691388000	37.710809000	45.344498000
6	48.236078000	38.248707000	39.517227000
6	46.347519000	32.598306000	38.492484000
6	45.277550000	33.398645000	44.072449000
6	46.899888000	37.386765000	43.952991000
6	47.697429000	36.927673000	39.749893000
6	46.135552000	32.920259000	39.883984000
7	47.365241000	36.771383000	41.071238000
1	45.253274000	31.081856000	40.456992000
1	45.092961000	34.111628000	46.060182000
1	47.748043000	39.242748000	43.366805000
1	47.823627000	36.258835000	37.750071000
6	43.460439000	42.639030000	34.267291000
6	42.324325000	41.937029000	36.089372000
6	41.710509000	41.707024000	37.321340000
6	41.679051000	39.684820000	35.329305000
6	42.304696000	40.909436000	35.096839000
6	43.076057000	43.043049000	35.535378000
6	41.089817000	40.463987000	37.560522000
6	41.068382000	39.463722000	36.568349000
7	42.994431000	41.379830000	34.000311000
6	43.423188000	44.341558000	36.203519000
6	44.350166000	45.197039000	35.333498000
7	44.871126000	46.339464000	36.072144000
6	45.619122000	47.272103000	35.471475000
8	45.831456000	47.272031000	34.234681000
6	46.187414000	48.352233000	36.368523000
8	40.535761000	40.324558000	38.795835000
6	39.956371000	39.070315000	39.154038000
1	44.069578000	43.138350000	33.521893000
1	41.724134000	42.441328000	38.120649000
1	47.197083000	48.603579000	36.030972000

1	40.697554000	38.263588000	39.111957000
1	39.104779000	38.823759000	38.508642000
1	39.627798000	39.187812000	40.187166000
1	41.663782000	38.909586000	34.567525000
1	40.580311000	38.515681000	36.759162000
1	42.514753000	44.914703000	36.442883000
1	43.909819000	44.155725000	37.171263000
1	43.817105000	45.550796000	34.444361000
1	45.188961000	44.589812000	34.968032000
1	46.210842000	48.039023000	37.415599000
1	45.573733000	49.258032000	36.300999000
1	43.330148000	40.861691000	33.166884000
1	44.789428000	46.339755000	37.099666000
8	42.282902000	36.338287000	40.683467000
1	43.178302000	36.018869000	40.921710000
1	42.290540000	37.287117000	40.948610000
1	48.536926000	38.608861000	38.542407000
1	48.543888000	39.897675000	40.947579000
1	46.946695000	38.658584000	45.801512000
1	45.819667000	36.471738000	46.948260000
1	44.315512000	31.741919000	45.242355000
1	44.379122000	30.451263000	42.854582000
1	46.092967000	31.650625000	38.034701000
1	47.196717000	33.848682000	36.885193000
1	43.539014000	41.161086000	43.725601000
1	44.157091000	43.531787000	39.949421000
1	43.281945000	50.551584000	42.142432000
1	40.600202000	47.144739000	41.403896000
1	36.328879000	39.529261000	33.958117000
1	32.592551000	41.633319000	35.446824000
1	36.937527000	33.482195000	38.519900000
1	39.449026000	32.283051000	42.129213000
1	46.146833000	45.583316000	29.821879000
1	43.566903000	48.687217000	31.377060000
1	46.835553000	51.446753000	33.164701000
1	40.776185000	50.921520000	38.388328000
1	42.579667000	54.923178000	35.527525000
1	46.676556000	54.259444000	38.556867000
1	44.592328000	37.204022000	39.255483000
1	46.655614000	40.592255000	37.229485000
1	45.350531000	37.193187000	34.502677000
1	49.789794000	37.915193000	34.539151000
1	49.196387000	35.362523000	43.005243000

^{2}RE$_{III,SH}$

6	-20.474266000	-43.437873000	29.121338000
6	-21.102254000	-42.304956000	33.781811000
6	-16.317675000	-41.847868000	34.296347000
6	-15.704351000	-42.822686000	29.596855000
6	-21.060178000	-43.208521000	30.361674000
6	-22.478095000	-43.276312000	30.615570000
6	-22.658830000	-42.958793000	31.928160000
6	-21.346464000	-42.695651000	32.471597000
6	-19.844299000	-42.064544000	34.327329000
6	-19.613517000	-41.687078000	35.701033000
6	-18.265157000	-41.576912000	35.851728000
6	-17.681552000	-41.876099000	34.565613000
6	-15.730079000	-42.081363000	33.058973000
6	-14.311803000	-42.006183000	32.802549000
6	-14.141402000	-42.267182000	31.476536000
6	-15.455600000	-42.506847000	30.930774000
6	-16.957643000	-43.089803000	29.066232000
6	-17.189662000	-43.427930000	27.681446000
6	-18.530713000	-43.610882000	27.548284000
6	-19.118135000	-43.379832000	28.848129000
7	-20.386242000	-42.867824000	31.504321000
7	-18.658183000	-42.172450000	33.654336000
7	-16.406835000	-42.395035000	31.909510000
7	-18.140355000	-43.076536000	29.766435000
26	-18.409203000	-42.492580000	31.681878000
1	-21.965107000	-42.152976000	34.423048000
1	-15.655505000	-41.598721000	35.120260000
1	-14.863476000	-42.873074000	28.909415000
1	-21.138323000	-43.679335000	28.296887000
6	-16.317215000	-35.633962000	28.457653000
6	-18.120223000	-36.933002000	28.825077000
6	-19.305793000	-37.568626000	29.246489000
6	-17.670518000	-38.763525000	27.237796000
6	-17.315832000	-37.541291000	27.826819000
6	-17.468376000	-35.706780000	29.215096000
6	-19.648905000	-38.786083000	28.668589000
6	-18.840054000	-39.372279000	27.666053000
7	-16.224196000	-36.724647000	27.611967000
1	-15.419179000	-36.988149000	27.044288000
6	-18.013161000	-34.720644000	30.214845000
6	-16.963105000	-33.844113000	30.921738000
7	-17.589734000	-32.937846000	31.879289000
6	-17.844716000	-31.648229000	31.625700000
8	-17.508293000	-31.068794000	30.563609000
6	-18.570003000	-30.890496000	32.722137000
1	-17.972379000	-33.337450000	32.748407000
8	-20.776105000	-39.505056000	29.002582000
6	-21.357603000	-39.237988000	30.269202000
1	-15.553665000	-34.868057000	28.435445000
1	-19.919255000	-37.119911000	30.019886000
1	-17.041051000	-39.234171000	26.488017000
1	-19.147429000	-40.331416000	27.261463000
1	-18.573931000	-35.264942000	30.985969000
1	-18.742346000	-34.049836000	29.735474000
1	-16.240707000	-34.467363000	31.460187000
1	-16.417241000	-33.231643000	30.199765000
1	-17.938213000	-30.059790000	33.055006000
1	-18.835389000	-31.535384000	33.561183000
1	-19.485401000	-30.456867000	32.304028000
1	-22.113046000	-40.013289000	30.421847000
1	-21.847746000	-38.254333000	30.297669000
1	-20.603932000	-39.298023000	31.062436000
16	-18.292690000	-45.043893000	32.041310000
6	-16.722876000	-40.870177000	21.457497000
6	-15.199318000	-40.842036000	21.505400000
8	-14.542974000	-41.885624000	21.361484000
6	-17.363696000	-41.695916000	22.596266000
6	-18.826260000	-42.029934000	22.237430000
6	-17.241057000	-40.964757000	23.940918000
6	-19.529441000	-42.965701000	23.227798000
1	-17.134354000	-39.852164000	21.458921000
1	-16.807499000	-42.642045000	22.658518000
1	-19.397968000	-41.093246000	22.154945000
1	-18.847559000	-42.491178000	21.239244000
1	-17.600051000	-41.583242000	24.770264000
1	-16.207999000	-40.690824000	24.178245000
1	-17.840230000	-40.043445000	23.936038000
1	-20.536675000	-43.220659000	22.877244000
1	-18.971570000	-43.903804000	23.347161000
1	-19.630699000	-42.509746000	24.218126000
7	-14.633621000	-39.622355000	21.649236000
6	-13.202675000	-39.381263000	21.772567000
6	-12.867877000	-38.866475000	23.172571000
8	-13.698106000	-37.754023000	23.456485000
1	-15.205092000	-38.856226000	21.985297000
1	-12.911182000	-38.634243000	21.022208000
1	-13.037839000	-39.665273000	23.902432000
1	-11.799630000	-38.589555000	23.200523000
1	-13.965740000	-37.844762000	24.409839000
6	-12.598457000	-42.914241000	24.653778000
6	-11.974574000	-43.853540000	23.627212000
8	-11.209902000	-44.758037000	23.963279000
6	-13.557571000	-43.656573000	25.598969000
8	-13.993532000	-42.848848000	26.679343000
1	-11.784778000	-42.494608000	25.256743000
1	-14.411757000	-44.040475000	25.015240000
1	-13.040060000	-44.521169000	26.026617000
1	-14.240634000	-41.950738000	26.337424000
7	-12.323450000	-43.613720000	22.328438000
6	-11.847640000	-44.442593000	21.237315000
6	-12.981807000	-44.919510000	20.303906000
6	-13.971220000	-45.857895000	20.960220000
6	-13.733943000	-47.238644000	21.001656000
6	-15.136859000	-45.361531000	21.561028000
6	-14.632244000	-48.103798000	21.628044000
6	-16.037034000	-46.224551000	22.189397000
6	-15.788780000	-47.598233000	22.225600000
1	-13.010500000	-42.893781000	22.112427000
1	-11.328134000	-45.290241000	21.692028000
1	-13.508199000	-44.038498000	19.917514000
1	-12.512308000	-45.416215000	19.443602000
1	-12.833521000	-47.639466000	20.539136000
1	-15.327079000	-44.291662000	21.531776000
1	-14.429070000	-49.172056000	21.649873000
1	-16.934622000	-45.821830000	22.652983000
1	-16.489666000	-48.268885000	22.716513000
6	-27.359469000	-41.377546000	32.915880000
6	-27.762386000	-40.974742000	31.502102000
8	-28.576200000	-41.640788000	30.837515000
6	-27.365210000	-42.905677000	33.108293000
8	-28.656653000	-43.448460000	32.877201000
6	-26.933267000	-43.289244000	34.518827000
1	-26.373076000	-40.974938000	33.166342000
1	-26.640440000	-43.336410000	32.392217000
1	-28.934654000	-43.042034000	32.028644000
1	-26.940161000	-44.379036000	34.630505000
1	-25.926605000	-42.913597000	34.732490000
1	-27.629344000	-42.868378000	35.254477000
7	-27.194834000	-39.843975000	31.022104000
6	-27.524204000	-39.332676000	29.698981000
6	-26.660191000	-39.911070000	28.557289000
6	-25.175727000	-39.530001000	28.716493000
6	-27.239560000	-39.462563000	27.207232000
6	-24.219875000	-40.262462000	27.766117000
1	-26.538224000	-39.322312000	31.607555000
1	-27.420172000	-38.239830000	29.724470000
1	-26.747255000	-41.004835000	28.624237000
1	-25.069341000	-38.442730000	28.575266000
1	-24.869941000	-39.733154000	29.750862000
1	-26.690060000	-39.897318000	26.365954000
1	-28.289921000	-39.763380000	27.108526000

1	-27.192694000	-38.369598000	27.102841000
1	-23.177954000	-39.986338000	27.960424000
1	-24.305815000	-41.349994000	27.890994000
1	-24.431315000	-40.033124000	26.714939000
6	-26.038529000	-36.169792000	31.969785000
6	-25.162778000	-37.088985000	32.827090000
8	-25.307416000	-38.330160000	32.726465000
6	-25.609532000	-34.701221000	31.820843000
6	-24.332373000	-34.481310000	30.974277000
6	-24.639580000	-34.553637000	29.470649000
6	-23.650541000	-33.156720000	31.346189000
1	-26.106682000	-36.649456000	30.986863000
1	-26.438825000	-34.145257000	31.360436000
1	-25.483442000	-34.250161000	32.814426000
1	-23.623420000	-35.286410000	31.210030000
1	-23.720445000	-34.474233000	28.879142000
1	-25.127219000	-35.496611000	29.193451000
1	-25.304467000	-33.732553000	29.168630000
1	-22.783907000	-32.966264000	30.702572000
1	-24.342853000	-32.310158000	31.235663000
1	-23.294238000	-33.185279000	32.381939000
7	-24.314909000	-36.505057000	33.671599000
6	-23.345966000	-37.131403000	34.571105000
6	-22.081612000	-36.247129000	34.569802000
8	-22.551204000	-34.906788000	34.631563000
6	-21.203356000	-36.474558000	33.335045000
1	-24.209662000	-35.494526000	33.680599000
1	-23.141037000	-38.154266000	34.249464000
1	-21.497727000	-36.485630000	35.469982000
1	-21.790010000	-34.341955000	34.975112000
1	-20.389237000	-35.743138000	33.335386000
1	-20.773981000	-37.484945000	33.333488000
1	-21.785681000	-36.343113000	32.413152000
6	-17.294254000	-24.988436000	31.785076000
6	-15.875489000	-24.860719000	31.221933000
8	-15.620155000	-24.101364000	30.292035000
6	-18.137768000	-26.002497000	30.993737000
6	-17.680396000	-27.452024000	31.200365000
8	-16.892006000	-27.753964000	32.112619000
7	-18.174486000	-28.363107000	30.345595000
1	-17.278146000	-25.280207000	32.839566000
1	-19.187777000	-25.929753000	31.309104000
1	-18.112759000	-25.755374000	29.925106000
1	-18.814340000	-28.085460000	29.615164000
1	-17.928245000	-29.371640000	30.443734000
7	-14.943752000	-25.657866000	31.832363000
6	-13.619483000	-25.849656000	31.269403000
6	-13.404307000	-27.082168000	30.364866000
8	-12.403613000	-27.128403000	29.652558000
1	-15.322114000	-26.369488000	32.448501000
1	-13.392056000	-24.974153000	30.659515000
7	-14.326711000	-28.073915000	30.443145000
6	-14.279912000	-29.244279000	29.572726000
6	-14.919769000	-28.962700000	28.195308000
6	-14.929082000	-30.167246000	27.278665000
6	-15.826361000	-31.226027000	27.496330000
6	-14.043488000	-30.252373000	26.196176000
6	-15.841029000	-32.331542000	26.643472000
6	-14.056016000	-31.358305000	25.342096000
6	-14.957835000	-32.401325000	25.561979000
1	-15.143334000	-27.971303000	31.037134000
1	-14.814827000	-30.055932000	30.074517000
1	-15.947120000	-28.616187000	28.366143000
1	-14.369840000	-28.137361000	27.729434000
1	-16.510824000	-31.188557000	28.341849000
1	-13.342236000	-29.439379000	26.018341000
1	-16.541947000	-33.141964000	26.825407000
1	-13.366151000	-31.401094000	24.502265000
1	-14.974362000	-33.261602000	24.897755000
6	-18.506562000	-34.627523000	37.598406000
6	-18.429569000	-36.113760000	37.236881000
8	-19.371061000	-36.889222000	37.401846000
6	-18.250680000	-33.669573000	36.417034000
6	-19.365632000	-33.761172000	35.347952000
8	-20.541942000	-33.700529000	35.800595000
8	-19.024450000	-33.884834000	34.137956000
1	-19.512719000	-34.447832000	37.980187000
1	-18.242322000	-32.642069000	36.806984000
1	-17.276436000	-33.844224000	35.947826000
7	-17.219056000	-36.517612000	36.754118000
6	-16.962750000	-37.831563000	36.195085000
6	-16.242344000	-37.699139000	34.844853000
8	-15.095551000	-36.883460000	35.100854000
6	-17.132697000	-37.129165000	33.740703000
1	-16.502045000	-35.834026000	36.546577000
1	-17.917856000	-38.352408000	36.092148000
1	-15.917110000	-38.710640000	34.544232000
1	-14.807491000	-36.455761000	34.264170000
1	-16.556208000	-37.007870000	32.817125000
1	-17.961208000	-37.814807000	33.528885000
1	-17.550322000	-36.155220000	34.017774000
6	-12.906074000	-34.921959000	30.872528000
6	-13.201811000	-35.903771000	31.994154000
8	-14.115265000	-35.668506000	32.799436000
1	-12.906138000	-33.908914000	31.284081000
1	-13.716135000	-34.976434000	30.136818000
7	-12.417382000	-37.003243000	32.024642000
6	-12.572852000	-38.093899000	32.979406000
6	-11.211117000	-38.585660000	33.474477000
1	-11.760077000	-37.146719000	31.253523000
1	-13.121441000	-38.924834000	32.510906000
1	-11.337687000	-39.433371000	34.158447000
1	-10.581159000	-38.913239000	32.638824000
1	-10.679845000	-37.789522000	34.007614000
6	-9.853370000	-36.806126000	27.560629000
6	-10.895548000	-37.369554000	28.532549000
8	-10.634651000	-37.401702000	29.747519000
6	-10.308072000	-36.356398000	26.161636000
6	-11.259615000	-35.141257000	26.102274000
6	-10.610117000	-33.863775000	26.656630000
6	-11.745244000	-34.923977000	24.660692000
1	-9.356217000	-35.990879000	28.095457000
1	-9.406074000	-36.114635000	25.579883000
1	-10.772194000	-37.201838000	25.634492000
1	-12.141988000	-35.362750000	26.719102000
1	-11.307236000	-33.020249000	26.597131000
1	-10.312033000	-33.968835000	27.706034000
1	-9.713614000	-33.599029000	26.078339000
1	-12.416787000	-34.059063000	24.607169000
1	-10.896246000	-34.726374000	23.990465000
1	-12.288396000	-35.795566000	24.276659000
7	-12.024694000	-37.865212000	27.994911000
6	-13.116040000	-38.484622000	28.742344000
6	-13.720918000	-39.652175000	27.956635000
6	-14.115526000	-39.335857000	26.500588000
8	-14.046639000	-38.116222000	26.119972000
8	-14.462646000	-40.299692000	25.774611000
1	-12.264168000	-37.685354000	27.025226000
1	-13.888858000	-37.737952000	28.959857000
1	-13.031039000	-40.504137000	27.925406000
1	-14.620995000	-40.004561000	28.476113000
8	-18.557772000	-40.918132000	31.302031000
1	-13.561826000	-41.777648000	33.549170000
1	-13.223952000	-42.302030000	30.903270000
1	-20.392010000	-41.524131000	36.434867000
1	-17.704916000	-41.303209000	36.736412000
1	-23.580579000	-42.894552000	32.491750000
1	-23.224945000	-43.533786000	29.875325000
1	-19.092110000	-43.872865000	26.660129000
1	-16.397945000	-43.486281000	26.946042000
1	-16.327025000	-38.414335000	36.879687000
1	-23.774478000	-37.167435000	35.580737000
1	-27.048974000	-36.226740000	32.398532000
1	-28.088006000	-40.940283000	33.613931000
1	-28.576095000	-39.566997000	29.507103000
1	-11.113041000	-43.888336000	20.632294000
1	-13.127974000	-42.082640000	24.177877000
1	-16.981595000	-41.322922000	20.491181000
1	-12.677726000	-40.313870000	21.554922000
1	-12.715569000	-38.833235000	29.696389000
1	-9.093294000	-37.593463000	27.454301000
1	-11.955700000	-35.121584000	30.371502000
1	-13.183236000	-37.735994000	33.810685000
1	-17.783561000	-34.429893000	38.403883000
1	-13.234013000	-29.535769000	29.444894000
1	-17.756295000	-24.000527000	31.702286000
1	-12.876276000	-25.899242000	32.074570000
8	-23.968305000	-40.570626000	33.728166000
1	-24.457047000	-39.740012000	33.536231000
1	-23.398620000	-40.672164000	32.950290000
1	-17.068423000	-45.236723000	31.500252000

^{4}RE$_{III,SH}$

6	-20.472952000	-43.438341000	29.123842000
6	-21.101219000	-42.295808000	33.782251000
6	-16.316469000	-41.852578000	34.298406000
6	-15.703331000	-42.813597000	29.596330000
6	-21.059262000	-43.207687000	30.363707000
6	-22.476511000	-43.279318000	30.619185000
6	-22.656833000	-42.958441000	31.931130000
6	-21.344825000	-42.689775000	32.472585000
6	-19.843769000	-42.054888000	34.328339000
6	-19.612425000	-41.674411000	35.701463000
6	-18.263823000	-41.570027000	35.852803000
6	-17.680530000	-41.875036000	34.567486000
6	-15.728782000	-42.083623000	33.060441000
6	-14.310289000	-42.007133000	32.804549000
6	-14.139466000	-42.261979000	31.477416000
6	-15.453553000	-42.500047000	30.930498000
6	-16.957101000	-43.081687000	29.066702000
6	-17.188462000	-43.426928000	27.683589000
6	-18.529117000	-43.613788000	27.551424000
6	-19.116853000	-43.377606000	28.850147000
7	-20.385134000	-42.860762000	31.504481000
7	-18.657801000	-42.168987000	33.656542000
7	-16.405015000	-42.392382000	31.909385000

7	-18.140049000	-43.065531000	29.766331000
26	-18.408528000	-42.493587000	31.680998000
1	-21.964712000	-42.142823000	34.422419000
1	-15.653614000	-41.606979000	35.122884000
1	-14.862828000	-42.864676000	28.908468000
1	-21.136315000	-43.684979000	28.300339000
6	-16.317429000	-35.641202000	28.464555000
6	-18.117865000	-36.943011000	28.834177000
6	-19.302559000	-37.579660000	29.256062000
6	-17.665650000	-38.774516000	27.248457000
6	-17.312661000	-37.551153000	27.836292000
6	-17.467958000	-35.715254000	29.222688000
6	-19.644446000	-38.797879000	28.678731000
6	-18.834538000	-39.384235000	27.676981000
7	-16.222588000	-36.732930000	27.620348000
1	-15.417888000	-36.994498000	27.051245000
6	-18.013871000	-34.728737000	30.221445000
6	-16.965070000	-33.847071000	30.923802000
7	-17.592563000	-32.939968000	31.879931000
6	-17.848456000	-31.650875000	31.624666000
8	-17.512405000	-31.072485000	30.561895000
6	-18.574422000	-30.892284000	32.720090000
1	-17.975008000	-33.338741000	32.749558000
8	-20.771698000	-39.516988000	29.011770000
6	-21.356826000	-39.247340000	30.276174000
1	-15.555353000	-34.873891000	28.440940000
1	-19.916674000	-37.130910000	30.028895000
1	-17.035610000	-39.244988000	26.499053000
1	-19.141061000	-40.343784000	27.272750000
1	-18.571152000	-35.273103000	30.995054000
1	-18.746355000	-34.061562000	29.742050000
1	-16.239784000	-34.466736000	31.462531000
1	-16.422482000	-33.234972000	30.199054000
1	-17.942431000	-30.061765000	33.053077000
1	-18.840752000	-31.536579000	33.559293000
1	-19.489210000	-30.458341000	32.301001000
1	-22.113331000	-40.021793000	30.427702000
1	-21.846165000	-38.263235000	30.301564000
1	-20.605439000	-39.306515000	31.071690000
16	-18.290626000	-45.020623000	32.062290000
6	-16.722305000	-40.865942000	21.459134000
6	-15.198741000	-40.837162000	21.505987000
8	-14.542012000	-41.880075000	21.358803000
6	-17.361784000	-41.693468000	22.597423000
6	-18.824352000	-42.028146000	22.239268000
6	-17.238784000	-40.963779000	23.942842000
6	-19.526194000	-42.965223000	23.229348000
1	-17.134308000	-39.848146000	21.462167000
1	-16.804858000	-42.639258000	22.658256000
1	-19.396749000	-41.091774000	22.157990000
1	-18.845991000	-42.488523000	21.240690000
1	-17.596972000	-41.583454000	24.771652000
1	-16.205719000	-40.689600000	24.179871000
1	-17.838449000	-40.042795000	23.939361000
1	-20.533540000	-43.220470000	22.879339000
1	-18.967639000	-43.903083000	23.347413000
1	-19.626923000	-42.510291000	24.220199000
7	-14.633475000	-39.617666000	21.652543000
6	-13.202554000	-39.376147000	21.775330000
6	-12.867290000	-38.863122000	23.175880000
8	-13.698080000	-37.751544000	23.461629000
1	-15.205072000	-38.852390000	21.990323000
1	-12.911802000	-38.628009000	21.025803000
1	-13.036388000	-39.663058000	23.904696000
1	-11.799209000	-38.585572000	23.203743000
1	-13.964551000	-37.843106000	24.415218000
6	-12.600440000	-42.914163000	24.650404000
6	-11.976340000	-43.852574000	23.623143000
8	-11.212391000	-44.757895000	23.958677000
6	-13.558638000	-43.657535000	25.595625000
8	-13.994062000	-42.850927000	26.677013000
1	-11.786746000	-42.494254000	25.253193000
1	-14.413122000	-44.041165000	25.012115000
1	-13.040588000	-44.522401000	26.022077000
1	-14.239365000	-41.951818000	26.336518000
7	-12.324003000	-43.610912000	22.324448000
6	-11.847150000	-44.438171000	21.232518000
6	-12.980360000	-44.912838000	20.296824000
6	-13.971050000	-45.852035000	20.950072000
6	-13.734144000	-47.232902000	20.989724000
6	-15.137515000	-45.356345000	21.549810000
6	-14.633678000	-48.098835000	21.613249000
6	-16.038927000	-46.220153000	22.175322000
6	-15.791075000	-47.593946000	22.209715000
1	-13.010667000	-42.890486000	22.108839000
1	-11.328672000	-45.286874000	21.686434000
1	-13.505870000	-44.030852000	19.911457000
1	-12.510049000	-45.408116000	19.436140000
1	-12.833055000	-47.633191000	20.528046000
1	-15.327338000	-44.286368000	21.522075000
1	-14.430786000	-49.167172000	21.633746000
1	-16.937158000	-45.817962000	22.638126000
1	-16.492908000	-48.265212000	22.698432000
6	-27.358800000	-41.375215000	32.911135000
6	-27.760771000	-40.971688000	31.497254000
8	-28.573821000	-41.637555000	30.831552000
6	-27.366550000	-42.903351000	33.103327000
8	-28.658585000	-43.444425000	32.871461000
6	-26.935902000	-43.287755000	34.514010000
1	-26.371916000	-40.973907000	33.161789000
1	-26.641971000	-43.334898000	32.387569000
1	-28.935370000	-43.037894000	32.022562000
1	-26.944260000	-44.377552000	34.625449000
1	-25.928854000	-42.913517000	34.728363000
1	-27.631847000	-42.866136000	35.249357000
7	-27.193033000	-39.840566000	31.018313000
6	-27.520908000	-39.328973000	29.694960000
6	-26.656350000	-39.908211000	28.554121000
6	-25.171566000	-39.529318000	28.715395000
6	-27.233351000	-39.458440000	27.203473000
6	-24.215486000	-40.263327000	27.766426000
1	-26.537306000	-39.318828000	31.604685000
1	-27.415818000	-38.236247000	29.720596000
1	-26.745082000	-41.001860000	28.620652000
1	-25.063344000	-38.442210000	28.574284000
1	-24.867568000	-39.732900000	29.750201000
1	-26.683394000	-39.893729000	26.362765000
1	-28.284015000	-39.757708000	27.103297000
1	-27.184778000	-38.365513000	27.099461000
1	-23.173382000	-39.989185000	27.962558000
1	-24.303621000	-41.350732000	27.890879000
1	-24.424736000	-40.033326000	26.714960000
6	-26.039651000	-36.165346000	31.969828000
6	-25.164047000	-37.085162000	32.826652000
8	-25.308173000	-38.326280000	32.724667000
6	-25.610056000	-34.696919000	31.821263000
6	-24.332801000	-34.477266000	30.974798000
6	-24.640004000	-34.548843000	29.471127000
6	-23.650405000	-33.153136000	31.347267000
1	-26.108266000	-36.644678000	30.986795000
1	-26.439119000	-34.140505000	31.360977000
1	-25.483797000	-34.246148000	32.814950000
1	-23.624194000	-35.282746000	31.210281000
1	-23.720823000	-34.469449000	28.879685000
1	-25.127916000	-35.491547000	29.193494000
1	-25.304624000	-33.727431000	29.169409000
1	-22.783725000	-32.962762000	30.703690000
1	-24.342368000	-32.306231000	31.237158000
1	-23.294059000	-33.182347000	32.382981000
7	-24.317014000	-36.501787000	33.672399000
6	-23.347967000	-37.128647000	34.571456000
6	-22.083285000	-36.244869000	34.569718000
8	-22.552435000	-34.904380000	34.631245000
6	-21.205310000	-36.472825000	33.334846000
1	-24.211792000	-35.491258000	33.682077000
1	-23.143584000	-38.151572000	34.249680000
1	-21.499283000	-36.483380000	35.469813000
1	-21.791424000	-34.339826000	34.975672000
1	-20.391122000	-35.741482000	33.334898000
1	-20.776048000	-37.483260000	33.333565000
1	-21.787795000	-36.341550000	32.413023000
6	-17.291927000	-24.991026000	31.777296000
6	-15.873288000	-24.865405000	31.213353000
8	-15.617666000	-24.107653000	30.282233000
6	-18.136869000	-26.005028000	30.987380000
6	-17.681153000	-27.454864000	31.195511000
8	-16.893035000	-27.756787000	32.108005000
7	-18.176502000	-28.366301000	30.341843000
1	-17.275661000	-25.281686000	32.832086000
1	-19.186703000	-25.930702000	31.302959000
1	-18.111891000	-25.759173000	29.918457000
1	-18.816012000	-28.088715000	29.611092000
1	-17.931251000	-29.374957000	30.440967000
7	-14.942050000	-25.662698000	31.824362000
6	-13.618335000	-25.856642000	31.260808000
6	-13.405266000	-27.090080000	30.357006000
8	-12.405206000	-27.138049000	29.643925000
1	-15.320841000	-26.373179000	32.441560000
1	-13.390067000	-24.981902000	30.650152000
7	-14.328710000	-28.080733000	30.436765000
6	-14.283621000	-29.252064000	29.567564000
6	-14.923679000	-28.971228000	28.190098000
6	-14.934966000	-30.176871000	27.274907000
6	-15.832612000	-31.234844000	27.495021000
6	-14.050809000	-30.263889000	26.191398000
6	-15.848889000	-32.341501000	26.643666000
6	-14.065043000	-31.370910000	25.338752000
6	-14.967070000	-32.413213000	25.561170000
1	-15.144589000	-27.976932000	31.031552000
1	-14.819321000	-30.062510000	30.070455000
1	-15.950494000	-28.623122000	28.360959000
1	-14.372831000	-28.147190000	27.723020000
1	-16.516141000	-31.195764000	28.341227000
1	-13.349332000	-29.451521000	26.011612000
1	-16.549977000	-33.151331000	26.827592000
1	-13.376318000	-31.415105000	24.498066000

1	-14.984708000	-33.274445000	24.898209000
6	-18.508047000	-34.626866000	37.599093000
6	-18.431708000	-36.113063000	37.237207000
8	-19.373401000	-36.888217000	37.402567000
6	-18.252384000	-33.668698000	36.417873000
6	-19.367672000	-33.760062000	35.349149000
8	-20.543833000	-33.698513000	35.802063000
8	-19.026927000	-33.884494000	34.139094000
1	-19.513966000	-34.446907000	37.981386000
1	-18.243829000	-32.641280000	36.808031000
1	-17.278312000	-33.843416000	35.948339000
7	-17.221534000	-36.517255000	36.753969000
6	-16.965566000	-37.831483000	36.195430000
6	-16.245123000	-37.699931000	34.845134000
8	-15.098473000	-36.883767000	35.100531000
6	-17.135519000	-37.131004000	33.740478000
1	-16.504278000	-35.833939000	36.546420000
1	-17.920768000	-38.352170000	36.092747000
1	-15.919645000	-38.711588000	34.545327000
1	-14.810395000	-36.456790000	34.263512000
1	-16.558796000	-37.009677000	32.817048000
1	-17.963399000	-37.817372000	33.528580000
1	-17.553854000	-36.157240000	34.017152000
6	-12.903034000	-34.921409000	30.875521000
6	-13.201851000	-35.904322000	31.995390000
8	-14.117339000	-35.669733000	32.798559000
1	-12.902476000	-33.908899000	31.288418000
1	-13.711972000	-34.973845000	30.138452000
7	-12.417669000	-37.003899000	32.026913000
6	-12.576020000	-38.095451000	32.980248000
6	-11.215503000	-38.590984000	33.474844000
1	-11.758741000	-37.147078000	31.257158000
1	-13.126486000	-38.924504000	32.510641000
1	-11.344170000	-39.439305000	34.157644000
1	-10.586186000	-38.918918000	32.638844000
1	-10.682431000	-37.796817000	34.009131000
6	-9.851094000	-36.802217000	27.566030000
6	-10.891995000	-37.369145000	28.537263000
8	-10.631026000	-37.402333000	29.752180000
6	-10.306701000	-36.352247000	26.167428000
6	-11.260738000	-35.139016000	26.109096000
6	-10.613909000	-33.860690000	26.664638000
6	-11.746756000	-34.921405000	24.667687000
1	-9.355936000	-35.986309000	28.101713000
1	-9.405198000	-36.108097000	25.585914000
1	-10.769056000	-37.198208000	25.639548000
1	-12.142693000	-35.362870000	26.725675000
1	-11.312744000	-33.018542000	26.605760000
1	-10.315697000	-33.966047000	27.713980000
1	-9.717893000	-33.593611000	26.086668000
1	-12.419928000	-34.057731000	24.615009000
1	-10.898137000	-34.721653000	23.997618000
1	-12.288287000	-35.793673000	24.282912000
7	-12.020119000	-37.866589000	27.999040000
6	-13.110152000	-38.488722000	28.746081000
6	-13.714113000	-39.655565000	27.958642000
6	-14.111173000	-39.336782000	26.503827000
8	-14.043265000	-38.116434000	26.125315000
8	-14.459205000	-40.299444000	25.776737000
1	-12.260367000	-37.685358000	27.029800000
1	-13.883789000	-37.743465000	28.965536000
1	-13.022873000	-40.506339000	27.924705000
1	-14.612921000	-40.010542000	28.478516000
8	-18.546840000	-40.908606000	31.331418000
1	-13.560593000	-41.781566000	33.552358000
1	-13.221865000	-42.294150000	30.904234000
1	-20.390712000	-41.505888000	36.434259000
1	-17.702981000	-41.296138000	36.737052000
1	-23.578170000	-42.896515000	32.495665000
1	-23.223329000	-43.541613000	29.880609000
1	-19.089994000	-43.882093000	26.664836000
1	-16.396384000	-43.487830000	26.948764000
1	-16.329961000	-38.414084000	36.880290000
1	-23.776133000	-37.164497000	35.581241000
1	-27.050017000	-36.221940000	32.398827000
1	-28.086878000	-40.937064000	33.609118000
1	-28.572856000	-39.562273000	29.502079000
1	-11.111460000	-43.883269000	20.629416000
1	-13.130557000	-42.082606000	24.175077000
1	-16.981546000	-41.317625000	20.492473000
1	-12.677387000	-40.308265000	21.556111000
1	-12.708550000	-38.838566000	29.699213000
1	-9.089143000	-37.587651000	27.459066000
1	-11.952071000	-35.121485000	30.375783000
1	-13.185704000	-37.737158000	33.811882000
1	-17.784638000	-34.429682000	38.404315000
1	-13.238126000	-29.544967000	29.439605000
1	-17.752961000	-24.002721000	31.693641000
1	-12.874796000	-25.906614000	32.065645000
8	-23.968066000	-40.567345000	33.722672000
1	-24.456825000	-39.736477000	33.531795000
1	-23.397821000	-40.667587000	32.945001000
1	-17.072580000	-45.222973000	31.510850000

Central iron atom parameter generated by MCPB.py

Fe atom of CpdI

```
d@<TRIPOS>MOLECULE
FE1
    1     0     1     0     0
SMALL
RESP Charge


@<TRIPOS>ATOM
      1 FE          4.9160   24.9970   23.9080 M1        1 FE1      0.042478
@<TRIPOS>BOND
@<TRIPOS>SUBSTRUCTURE
     1 FE1        1 TEMP              0 ****  ****    0 ROOT
```

Por complex of CpdI

```
@<TRIPOS>MOLECULE
HM1
   72    76     1     0     0
SMALL
RESP Charge


@<TRIPOS>ATOM
      1 CHA         5.2990   25.5880   20.4970 ce        1 HM1     -0.149972
      2 CHB         8.1810   24.4350   24.1630 ce        1 HM1     -0.171065
      3 CHC         4.5320   24.0690   27.1820 ce        1 HM1     -0.214791
      4 CHD         1.7430   24.8320   23.4530 ce        1 HM1     -0.088846
      5 C1A         6.4790   25.3360   21.2110 cc        1 HM1     -0.006007
```

6	C2A	7.8190	25.3410	20.6940	cc	1 HM1	-0.063546
7	C3A	8.6560	25.0820	21.7840	cd	1 HM1	0.024111
8	C4A	7.7860	24.9180	22.9170	cd	1 HM1	-0.031981
9	CMA	10.2610	24.9950	21.7700	c3	1 HM1	-0.298638
10	CAA	8.2700	25.5850	19.2740	c3	1 HM1	-0.055406
11	CBA	8.5860	27.0180	18.8670	c3	1 HM1	-0.102302
12	CGA	9.1290	27.0440	17.4180	c	1 HM1	0.700663
13	O1A	9.9800	27.8390	17.0880	o	1 HM1	-0.563996
14	O2A	8.7710	26.2450	16.5850	o	1 HM1	-0.525312
15	C1B	7.5010	24.1910	25.3530	cc	1 HM1	-0.004532
16	C2B	8.1050	23.8060	26.5880	cc	1 HM1	0.065865
17	C3B	7.0200	23.6170	27.4710	cd	1 HM1	-0.021290
18	C4B	5.8360	23.9190	26.6850	cd	1 HM1	-0.005691
19	CMB	9.6190	23.6600	26.8390	c3	1 HM1	-0.218500
20	CAB	7.0260	23.2290	28.8230	cf	1 HM1	-0.075056
21	CBB	8.0620	23.1600	29.7660	c2	1 HM1	-0.469514
22	C1C	3.3770	24.3330	26.4810	cc	1 HM1	0.057021
23	C2C	2.1030	24.2600	26.9930	cc	1 HM1	0.060742
24	C3C	1.2340	24.3100	25.8660	cd	1 HM1	-0.025203
25	C4C	2.1010	24.4050	24.7500	cd	1 HM1	-0.031937
26	CMC	1.7630	24.1530	28.5150	c3	1 HM1	-0.170304
27	CAC	-0.1430	24.2840	25.7030	cf	1 HM1	-0.074837
28	CBC	-1.1220	24.4650	26.6570	c2	1 HM1	-0.446459
29	C1D	2.4180	24.9280	22.2820	cc	1 HM1	-0.011905
30	C2D	1.8100	25.1480	21.0120	cc	1 HM1	0.106347
31	C3D	2.8200	25.4120	20.1390	cd	1 HM1	-0.085844
32	C4D	3.9980	25.3270	20.9310	cd	1 HM1	0.005751
33	CMD	0.2980	25.0880	20.6880	c3	1 HM1	-0.323133
34	CAD	2.7920	25.7390	18.6180	c3	1 HM1	-0.066217

35	CBD	2.7120	27.3010	18.3350	c3	1 HM1	-0.032525
36	CGD	2.7590	27.7460	16.8150	c	1 HM1	0.551914
37	O1D	2.3810	28.9210	16.4360	o	1 HM1	-0.540575
38	O2D	3.2250	26.8720	15.9620	o	1 HM1	-0.505851
39	NA	6.5090	25.0700	22.5760	Y3	1 HM1	0.074371
40	NB	6.1560	24.2480	25.4440	Y4	1 HM1	0.012513
41	NC	3.3630	24.4140	25.1650	Y5	1 HM1	0.061010
42	ND	3.6970	25.0430	22.1850	Y6	1 HM1	0.066520
43	HHA	5.4070	26.0270	19.5160	ha	1 HM1	0.214672
44	HHB	9.2340	24.2010	24.2170	ha	1 HM1	0.159095
45	HHC	4.4200	23.9640	28.2510	ha	1 HM1	0.153782
46	HHD	0.7130	25.1490	23.3780	ha	1 HM1	0.095662
47	HAA2	9.1720	24.9950	19.1130	hc	1 HM1	0.035218
48	HAA3	7.4970	25.2070	18.6050	hc	1 HM1	0.035218
49	HBA2	9.3370	27.4290	19.5420	hc	1 HM1	0.025979
50	HBA3	7.6790	27.6200	18.9250	hc	1 HM1	0.025979
51	HAB	6.0600	22.9310	29.2030	ha	1 HM1	0.106817
52	HAC	-0.4940	24.0980	24.6990	ha	1 HM1	0.100737
53	HAD2	3.6990	25.3460	18.1590	hc	1 HM1	0.032992
54	HAD3	1.9270	25.2520	18.1670	hc	1 HM1	0.032992
55	HBD2	3.5520	27.7730	18.8450	hc	1 HM1	0.022084
56	HBD3	1.7890	27.6810	18.7740	hc	1 HM1	0.022084
57	HBC1	-0.8470	24.6560	27.6840	ha	1 HM1	0.147977
58	HBC2	-2.1640	24.4160	26.3770	ha	1 HM1	0.147977
59	HMA1	10.6230	24.7820	22.7760	hc	1 HM1	0.087046
60	HMA2	10.5760	24.1990	21.0950	hc	1 HM1	0.087046
61	HMA3	10.6730	25.9450	21.4290	hc	1 HM1	0.087046
62	HMB1	10.1590	23.8130	25.9050	hc	1 HM1	0.061747
63	HMB2	9.9390	24.4030	27.5690	hc	1 HM1	0.061747

64	HMB3	9.8290	22.6610	27.2210	hc	1 HM1	0.061747
65	HMC1	2.6740	23.9490	29.0780	hc	1 HM1	0.045527
66	HMC2	1.3280	25.0920	28.8570	hc	1 HM1	0.045527
67	HMC3	1.0500	23.3440	28.6720	hc	1 HM1	0.045527
68	HMD1	0.1500	25.2150	19.6160	hc	1 HM1	0.085077
69	HMD2	-0.2200	25.8840	21.2230	hc	1 HM1	0.085077
70	HMD3	-0.1020	24.1230	20.9980	hc	1 HM1	0.085077
71	HBB1	9.0670	23.4370	29.4840	ha	1 HM1	0.155797
72	HBB2	7.8550	22.8290	30.7730	ha	1 HM1	0.155797

@<TRIPOS>BOND

1	1	5	1
2	1	32	1
3	1	43	1
4	2	8	1
5	2	15	1
6	2	44	1
7	3	18	1
8	3	22	1
9	3	45	1
10	4	25	1
11	4	29	1
12	4	46	1
13	5	6	1
14	5	39	1
15	6	7	1
16	6	10	1
17	7	8	1
18	7	9	1
19	8	39	1

20	9	59	1
21	9	60	1
22	9	61	1
23	10	11	1
24	10	47	1
25	10	48	1
26	11	12	1
27	11	49	1
28	11	50	1
29	12	13	1
30	12	14	1
31	15	16	1
32	15	40	1
33	16	17	1
34	16	19	1
35	17	18	1
36	17	20	1
37	18	40	1
38	19	62	1
39	19	63	1
40	19	64	1
41	20	21	1
42	20	51	1
43	21	71	1
44	21	72	1
45	22	23	1
46	22	41	1
47	23	24	1
48	23	26	1

```
49   24   25 1
50   24   27 1
51   25   41 1
52   26   65 1
53   26   66 1
54   26   67 1
55   27   28 1
56   27   52 1
57   28   57 1
58   28   58 1
59   29   30 1
60   29   42 1
61   30   31 1
62   30   33 1
63   31   32 1
64   31   34 1
65   32   42 1
66   33   68 1
67   33   69 1
68   33   70 1
69   34   35 1
70   34   53 1
71   34   54 1
72   35   36 1
73   35   55 1
74   35   56 1
75   36   37 1
76   36   38 1
@<TRIPOS>SUBSTRUCTURE
```

1 HM1 1 TEMP 0 **** **** 0 ROOT

Oxygen atom of CpdI

@<TRIPOS>MOLECULE

OO1

1 0 1 0 0

SMALL

RESP Charge

@<TRIPOS>ATOM

1 O1 4.7240 23.5100 23.4100 Y2 1 OO1 -0.412137

@<TRIPOS>BOND

@<TRIPOS>SUBSTRUCTURE

1 OO1 1 TEMP 0 **** **** 0 ROOT

Cysteine ligand of CpdI

@<TRIPOS>MOLECULE

CS1

10 9 1 0 0

SMALL

RESP Charge

@<TRIPOS>ATOM

1 N 6.3070 29.4380 21.9000 N 1 CS1 -0.415700

2 CA 5.3050 28.7430 22.6880 CX 1 CS1 0.021300

3 C 4.1120 29.5970 23.2160 C 1 CS1 0.597300

4 O 4.2890 30.6070 23.8710 O 1 CS1 -0.567900

5 CB 5.9850 28.0610 23.8670 2C 1 CS1 -0.009069

6 SG 4.7990 27.1450 24.8480 Y1 1 CS1 -0.515874

```
  7 H          7.2770   29.1830   22.0200 H        1 CS1      0.233040
  8 HA         4.8860   27.9580   22.0580 H1       1 CS1     -0.012348
  9 HB2        6.7440   27.3740   23.4920 H1       1 CS1      0.062133
 10 HB3        6.4610   28.8160   24.4920 H1       1 CS1      0.062133
@<TRIPOS>BOND
 1    1    2 1
 2    1    7 1
 3    2    3 1
 4    2    5 1
 5    2    8 1
 6    3    4 1
 7    5    6 1
 8    5    9 1
 9    5   10 1
@<TRIPOS>SUBSTRUCTURE
 1 CS1        1 TEMP           0 ****  ****    0 ROOT
```

Hydrogen bonds list of model I, II, III and IV of CYP1A1 with melatonin

Model I

RES_DONOR,ATOM_DONOR,RES_ACCEPTOR,ATOM_ACCEPTOR, TAG

1,37,O,39,H,37-O-39-H

2,37,O,39,HD22,37-O-39-HD22

3,38,O,400,H,38-O-400-H

4,39,O,38,HZ2,39-O-38-HZ2

5,39,OD1,39,H,39-OD1-39-H

6,39,ND2,39,H,39-ND2-39-H

7,39,ND2,75,HE22,39-ND2-75-HE22

8,39,OD1,405,HH21,39-OD1-405-HH21

9,39,O,39,HD22,39-O-39-HD22

10,39,OD1,75,HE22,39-OD1-75-HE22

11,40,O,75,HE21,40-O-75-HE21

12,40,O,39,HD22,40-O-39-HD22

13,41,O,75,H,41-O-75-H

14,43,O,51,HE2,43-O-51-HE2

15,44,O,51,HE2,44-O-51-HE2

16,45,O,77,HE,45-O-77-HE

17,45,O,77,HH22,45-O-77-HH22

18,45,O,77,HH21,45-O-77-HH21

19,45,O,77,HH11,45-O-77-HH11

20,45,O,77,HH12,45-O-77-HH12

21,46,O,49,H,46-O-49-H

22,46,O,50,H,46-O-50-H

23,46,O,77,HH21,46-O-77-HH21

24,46,O,48,H,46-O-48-H

25,46,O,77,HH12,46-O-77-HH12

26,47,O,77,HE,47-O-77-HE

27,47,O,77,HH22,47-O-77-HH22

28,47,O,77,HH11,47-O-77-HH11

29,47,O,80,HG,47-O-80-HG

30,47,O,77,HH21,47-O-77-HH21

31,49,O,79,H,49-O-79-H

32,50,O,52,H,50-O-52-H

33,50,O,53,H,50-O-53-H

34,50,O,46,H,50-O-46-H

35,51,O,55,H,51-O-55-H

36,51,ND1,51,H,51-ND1-51-H

37,51,O,54,H,51-O-54-H

38,51,ND1,77,H,51-ND1-77-H

39,51,O,54,HG1,51-O-54-HG1

40,52,O,54,H,52-O-54-H

41,52,O,55,H,52-O-55-H

42,52,O,56,H,52-O-56-H

43,53,O,56,H,53-O-56-H

44,54,O,65,HH22,54-O-65-HH22

45,55,O,58,HD22,55-O-58-HD22

46,55,O,58,H,55-O-58-H

47,57,O,58,HD22,57-O-58-HD22

48,57,O,57,HZ1,57-O-57-HZ1

49,57,O,57,HZ2,57-O-57-HZ2

50,58,OD1,494,H,58-OD1-494-H

51,58,O,58,HD22,58-O-58-HD22

52,58,O,61,H,58-O-61-H

53,58,O,62,H,58-O-62-H

54,59,O,63,H,59-O-63-H

55,59,O,62,H,59-O-62-H

56,60,O,64,H,60-O-64-H

57,60,O,64,HG,60-O-64-HG

58,60,ND1,61,H,60-ND1-61-H

59,60,O,410,HD21,60-O-410-HD21

60,60,ND1,60,H,60-ND1-60-H

61,61,O,64,H,61-O-64-H

62,61,O,65,H,61-O-65-H

63,62,O,66,H,62-O-66-H

64,62,O,65,H,62-O-65-H

65,63,O,67,H,63-O-67-H

66,63,O,67,HG,63-O-67-HG

67,63,O,66,H,63-O-66-H

68,63,O,410,HD22,63-O-410-HD22

69,64,O,68,HE22,64-O-68-HE22

70,64,O,67,H,64-O-67-H

71,64,OG,410,HD21,64-OG-410-HD21

72,64,O,68,H,64-O-68-H

73,64,O,67,HG,64-O-67-HG

74,64,O,68,HE21,64-O-68-HE21

75,64,O,413,HE22,64-O-413-HE22

76,65,O,65,HH11,65-O-65-HH11

77,65,O,68,H,65-O-68-H

78,65,O,69,HE22,65-O-69-HE22

79,65,O,69,HE21,65-O-69-HE21

80,65,O,69,H,65-O-69-H

81,66,O,69,H,66-O-69-H

82,66,O,70,H,66-O-70-H

83,67,OG,413,HE22,67-OG-413-HE22

84,67,OG,410,HD22,67-OG-410-HD22

85,67,O,70,H,67-O-70-H

86,67,O,69,H,67-O-69-H

87,67,O,71,H,67-O-71-H

88,67,OG,68,HE21,67-OG-68-HE21

89,67,OG,413,HE21,67-OG-413-HE21

90,67,OG,87,HG,67-OG-87-HG

91,68,NE2,418,HE22,68-NE2-418-HE22

92,68,OE1,418,HE22,68-OE1-418-HE22

93,68,OE1,419,HZ1,68-OE1-419-HZ1

94,68,OE1,65,HH11,68-OE1-65-HH11

95,68,OE1,419,HZ3,68-OE1-419-HZ3

96,68,OE1,419,HZ2,68-OE1-419-HZ2

97,68,NE2,413,HE22,68-NE2-413-HE22

98,68,OE1,68,H,68-OE1-68-H

99,68,OE1,413,HE22,68-OE1-413-HE22

100,68,OE1,65,HE,68-OE1-65-HE

101,68,O,68,HE21,68-O-68-HE21

102,69,OE1,65,HH22,69-OE1-65-HH22

103,69,OE1,68,HE21,69-OE1-68-HE21

104,69,OE1,70,HH,69-OE1-70-HH

105,69,O,69,HE21,69-O-69-HE21

106,69,OE1,65,HH12,69-OE1-65-HH12

107,70,OH,69,HE21,70-OH-69-HE21

108,71,O,67,HG,71-O-67-HG

109,71,O,87,HG,71-O-87-HG

110,72,OD2,91,HG1,72-OD2-91-HG1

111,72,OD2,397,HZ1,72-OD2-397-HZ1

112,72,OD1,72,H,72-OD1-72-H

113,72,O,88,H,72-O-88-H

114,72,OD1,91,HG1,72-OD1-91-HG1

115,72,OD1,397,HZ3,72-OD1-397-HZ3

116,72,OD1,397,HZ2,72-OD1-397-HZ2

117,72,OD2,397,HZ2,72-OD2-397-HZ2

118,72,OD2,72,H,72-OD2-72-H

119,72,OD1,397,HZ1,72-OD1-397-HZ1

120,72,OD2,397,HZ3,72-OD2-397-HZ3

121,72,O,87,H,72-O-87-H

122,74,O,85,H,74-O-85-H

123,75,OE1,39,HD22,75-OE1-39-HD22

124,75,NE2,39,HD21,75-NE2-39-HD21

125,75,OE1,77,HH21,75-OE1-77-HH21

126,75,OE1,405,HH21,75-OE1-405-HH21

127,75,OE1,39,HD21,75-OE1-39-HD21

128,75,OE1,405,HE,75-OE1-405-HE

129,75,NE2,39,HD22,75-NE2-39-HD22

130,75,OE1,77,HE,75-OE1-77-HE

131,76,O,75,HE21,76-O-75-HE21

132,76,O,83,H,76-O-83-H

133,77,O,51,H,77-O-51-H

134,78,O,81,H,78-O-81-H

135,78,O,80,H,78-O-80-H

136,79,O,77,HH11,79-O-77-HH11

137,79,O,81,H,79-O-81-H

138,79,O,48,H,79-O-48-H

139,79,O,77,HE,79-O-77-HE

140,80,O,80,HG,80-O-80-HG

141,80,O,77,HH11,80-O-77-HH11

142,80,O,77,HE,80-O-77-HE

143,80,O,77,HH21,80-O-77-HH21

144,80,O,81,HG1,80-O-81-HG1

145,80,OG,77,HH21,80-OG-77-HH21

146,80,OG,81,HG1,80-OG-81-HG1

147,81,O,81,HG1,81-O-81-HG1

148,81,O,78,H,81-O-78-H

149,82,O,405,HH21,82-O-405-HH21

150,82,O,405,HH11,82-O-405-HH11

151,83,O,76,H,83-O-76-H

152,83,O,75,HE21,83-O-75-HE21

153,84,O,408,H,84-O-408-H

154,85,O,87,HG,85-O-87-HG

155,85,O,74,H,85-O-74-H

156,86,O,410,H,86-O-410-H

157,86,O,87,HG,86-O-87-HG

158,87,O,87,HG,87-O-87-HG

159,87,O,413,HE21,87-O-413-HE21

160,87,O,91,HG1,87-O-91-HG1

161,87,O,413,HE22,87-O-413-HE22

162,87,OG,410,HD22,87-OG-410-HD22

163,87,OG,67,HG,87-OG-67-HG

164,88,O,92,H,88-O-92-H

165,88,O,91,H,88-O-91-H

166,89,O,93,H,89-O-93-H

167,89,O,92,H,89-O-92-H

168,90,O,98,HH12,90-O-98-HH12

169,90,OD2,93,HH12,90-OD2-93-HH12

170,90,OD2,93,HH22,90-OD2-93-HH22

171,90,OD2,419,HZ2,90-OD2-419-HZ2

172,90,OD1,93,HH21,90-OD1-93-HH21

173,90,OD2,98,HH12,90-OD2-98-HH12

174,90,OD2,93,HE,90-OD2-93-HE

175,90,OD2,91,H,90-OD2-91-H

176,90,OD1,397,HZ3,90-OD1-397-HZ3

177,90,OD1,397,HZ1,90-OD1-397-HZ1

178,90,O,98,HH22,90-O-98-HH22

179,90,O,94,H,90-O-94-H

180,90,OD1,98,HH22,90-OD1-98-HH22

181,90,OD2,397,HZ3,90-OD2-397-HZ3

182,90,OD1,93,HH11,90-OD1-93-HH11

183,90,OD2,93,HH21,90-OD2-93-HH21

184,90,OD1,93,HE,90-OD1-93-HE

185,90,OD2,90,H,90-OD2-90-H

186,90,OD1,91,H,90-OD1-91-H

187,90,OD1,90,H,90-OD1-90-H

188,90,OD2,397,HZ1,90-OD2-397-HZ1

189,90,OD2,91,HG1,90-OD2-91-HG1

190,90,OD2,93,HH11,90-OD2-93-HH11

191,90,OD2,397,HZ2,90-OD2-397-HZ2

192,90,OD1,91,HG1,90-OD1-91-HG1

193,90,OD2,98,HH22,90-OD2-98-HH22

194,90,OD1,397,HZ2,90-OD1-397-HZ2

195,90,OD1,98,HH12,90-OD1-98-HH12

196,91,OG1,397,HZ1,91-OG1-397-HZ1

197,91,O,95,H,91-O-95-H

198,91,OG1,397,HZ3,91-OG1-397-HZ3

199,91,OG1,397,HZ2,91-OG1-397-HZ2

200,92,O,96,H,92-O-96-H

201,92,O,95,H,92-O-95-H

202,93,O,98,H,93-O-98-H

203,93,O,97,H,93-O-97-H

204,94,OE1,397,H,94-OE1-397-H

205,94,O,98,H,94-O-98-H

206,94,OE1,397,HZ2,94-OE1-397-HZ2

207,94,OE1,397,HZ3,94-OE1-397-HZ3

208,94,O,99,H,94-O-99-H

209,94,OE1,397,HZ1,94-OE1-397-HZ1

210,94,O,99,HE21,94-O-99-HE21

211,94,OE1,98,HE,94-OE1-98-HE

212,94,OE1,98,HH21,94-OE1-98-HH21

213,94,OE1,98,HH11,94-OE1-98-HH11

214,95,O,100,H,95-O-100-H

215,95,O,99,H,95-O-99-H

216,96,O,454,H,96-O-454-H

217,97,O,100,H,97-O-100-H

218,97,O,454,HZ3,97-O-454-HZ3

219,97,O,454,HZ2,97-O-454-HZ2

220,98,O,99,HE21,98-O-99-HE21

221,98,O,98,HH11,98-O-98-HH11

222,99,O,103,H,99-O-103-H

223,99,O,102,H,99-O-102-H

224,99,O,101,H,99-O-101-H

225,99,OE1,94,HE21,99-OE1-94-HE21

226,99,OE1,394,HG1,99-OE1-394-HG1

227,100,O,104,H,100-O-104-H

228,100,O,103,H,100-O-103-H

229,101,OD2,392,HH12,101-OD2-392-HH12

230,101,OD1,392,HH12,101-OD1-392-HH12

231,101,O,104,HZ1,101-O-104-HZ1

232,101,O,104,HZ2,101-O-104-HZ2

233,101,OD1,392,HH22,101-OD1-392-HH22

234,101,OD2,454,HZ1,101-OD2-454-HZ1

235,101,O,104,HZ3,101-O-104-HZ3

236,101,OD2,392,HH22,101-OD2-392-HH22

237,101,OD2,104,HZ1,101-OD2-104-HZ1

238,101,OD2,454,HZ3,101-OD2-454-HZ3

239,101,OD1,454,HZ3,101-OD1-454-HZ3

240,101,OD2,104,HZ3,101-OD2-104-HZ3

241,101,OD2,104,HZ2,101-OD2-104-HZ2

242,101,OD1,454,HZ2,101-OD1-454-HZ2

243,101,OD2,454,HZ2,101-OD2-454-HZ2

244,101,OD1,104,HZ3,101-OD1-104-HZ3

245,101,OD1,104,HZ1,101-OD1-104-HZ1

246,101,OD1,104,HZ2,101-OD1-104-HZ2

247,101,OD1,454,HZ1,101-OD1-454-HZ1

248,101,O,392,HH12,101-O-392-HH12

249,101,O,391,HG1,101-O-391-HG1

250,102,OD2,392,HE,102-OD2-392-HE

251,102,OD1,392,HH21,102-OD1-392-HH21

252,102,OD2,394,HG1,102-OD2-394-HG1

253,102,OD1,392,HH11,102-OD1-392-HH11

254,102,OD1,392,HE,102-OD1-392-HE

255,102,OD2,99,HE21,102-OD2-99-HE21

256,102,OD2,392,HH21,102-OD2-392-HH21

257,102,O,104,H,102-O-104-H

258,102,O,391,HG1,102-O-391-HG1

259,102,OD1,394,HG1,102-OD1-394-HG1

260,102,O,391,H,102-O-391-H

261,102,OD1,99,HE21,102-OD1-99-HE21

262,103,O,388,HD1,103-O-388-HD1

263,103,O,105,H,103-O-105-H

264,104,O,455,HE,104-O-455-HE

265,104,O,455,HH21,104-O-455-HH21

266,104,O,127,HG,104-O-127-HG

267,105,O,389,H,105-O-389-H

268,107,O,109,H,107-O-109-H

269,108,OD2,108,H,108-OD2-108-H

270,108,OD1,241,HH22,108-OD1-241-HH22

271,108,OD1,241,HH12,108-OD1-241-HH12

272,108,OD2,241,HH22,108-OD2-241-HH22

273,108,OD2,241,HH12,108-OD2-241-HH12

274,108,OD2,109,H,108-OD2-109-H

275,108,O,241,HH22,108-O-241-HH22

276,108,OD1,108,H,108-OD1-108-H

277,108,O,241,HH12,108-O-241-HH12

278,109,O,113,HG1,109-O-113-HG1

279,109,O,112,H,109-O-112-H

280,109,O,113,H,109-O-113-H

281,110,O,113,H,110-O-113-H

282,110,OH,252,HZ2,110-OH-252-HZ2

283,110,O,114,H,110-O-114-H

284,110,OH,241,HH21,110-OH-241-HH21

285,110,OH,252,HZ1,110-OH-252-HZ1

286,110,OH,242,HH,110-OH-242-HH

287,110,O,113,HG1,110-O-113-HG1

288,110,OH,252,HZ3,110-OH-252-HZ3

289,111,O,114,H,111-O-114-H

290,111,OG1,112,H,111-OG1-112-H

291,111,O,115,H,111-O-115-H

292,112,O,115,H,112-O-115-H

293,113,O,118,H,113-O-118-H

294,113,O,113,HG1,113-O-113-HG1

295,114,O,117,H,114-O-117-H

296,114,O,118,H,114-O-118-H

297,115,O,120,H,115-O-120-H

298,115,O,118,H,115-O-118-H

299,116,O,309,HD22,116-O-309-HD22

300,116,O,119,HE21,116-O-119-HE21

301,116,O,309,HD21,116-O-309-HD21

302,116,O,117,HD22,116-O-117-HD22

303,116,O,119,H,116-O-119-H

304,117,OD1,119,HE21,117-OD1-119-HE21

305,117,ND2,259,HH,117-ND2-259-HH

306,117,O,119,HE21,117-O-119-HE21

307,117,OD1,309,HD21,117-OD1-309-HD21

308,117,OD1,119,HE22,117-OD1-119-HE22

309,117,OD1,309,HD22,117-OD1-309-HD22

310,117,O,117,HD22,117-O-117-HD22

311,118,O,124,HG,118-O-124-HG

312,118,O,119,HE21,118-O-119-HE21

313,119,OE1,134,HH22,119-OE1-134-HH22

314,119,OE1,309,HD22,119-OE1-309-HD22

315,119,OE1,306,HZ2,119-OE1-306-HZ2

316,119,OE1,309,HD21,119-OE1-309-HD21

317,119,OE1,117,HD22,119-OE1-117-HD22

318,119,NE2,309,HD22,119-NE2-309-HD22

319,119,OE1,306,HZ1,119-OE1-306-HZ1

320,119,OE1,306,HZ3,119-OE1-306-HZ3

321,119,O,309,HD21,119-O-309-HD21

322,119,O,134,HH21,119-O-134-HH21

323,119,O,134,HH12,119-O-134-HH12

324,119,O,134,HH22,119-O-134-HH22

325,120,O,134,HH12,120-O-134-HH12

326,120,OG,122,H,120-OG-122-H

327,120,O,134,HH22,120-O-134-HH22

328,120,O,124,HG,120-O-124-HG

329,120,OG,123,H,120-OG-123-H

330,120,OG,122,HG,120-OG-122-HG

331,120,O,123,H,120-O-123-H

332,120,O,124,H,120-O-124-H

333,121,O,455,HH22,121-O-455-HH22

334,121,O,455,HH12,121-O-455-HH12

335,121,O,124,H,121-O-124-H

336,121,O,131,HE1,121-O-131-HE1

337,122,O,106,HH21,122-O-106-HH21

338,122,OG,516,H10,122-OG-516-H10

339,122,O,455,HH12,122-O-455-HH12

340,122,O,106,HE,122-O-106-HE

341,124,OG,306,HZ3,124-OG-306-HZ3

342,124,O,455,HH12,124-O-455-HH12

343,124,O,455,HH22,124-O-455-HH22

344,124,OG,126,H,124-OG-126-H

345,124,OG,134,HH12,124-OG-134-HH12

346,124,O,126,H,124-O-126-H

347,124,OG,455,HH22,124-OG-455-HH22

348,124,OG,455,HH12,124-OG-455-HH12

349,125,O,127,H,125-O-127-H

350,125,O,127,HG,125-O-127-HG

351,125,O,106,H,125-O-106-H

352,126,OD2,134,HH12,126-OD2-134-HH12

353,126,OD1,126,H,126-OD1-126-H

354,126,OD2,134,HH22,126-OD2-134-HH22

355,126,OD2,126,H,126-OD2-126-H

356,126,OD1,127,H,126-OD1-127-H

357,126,OD1,131,H,126-OD1-131-H

358,126,OD2,306,HZ3,126-OD2-306-HZ3

359,126,OD1,124,HG,126-OD1-124-HG

360,126,OD1,134,HH12,126-OD1-134-HH12

361,126,O,455,HH21,126-O-455-HH21

362,126,O,124,HG,126-O-124-HG

363,126,OD1,128,H,126-OD1-128-H

364,126,OD2,128,H,126-OD2-128-H

365,126,OD2,124,HG,126-OD2-124-HG

366,126,OD1,134,HH22,126-OD1-134-HH22

367,126,OD2,127,H,126-OD2-127-H

368,126,OD2,306,HZ2,126-OD2-306-HZ2

369,126,OD2,134,HH11,126-OD2-134-HH11

370,126,OD1,306,HZ3,126-OD1-306-HZ3

371,126,O,455,HE,126-O-455-HE

372,126,OD2,306,HZ1,126-OD2-306-HZ1

373,126,OD1,306,HZ1,126-OD1-306-HZ1

374,126,O,455,HH22,126-O-455-HH22

375,126,O,455,HH12,126-O-455-HH12

376,126,OD1,306,HZ2,126-OD1-306-HZ2

377,126,OD2,131,H,126-OD2-131-H

378,126,O,127,HG,126-O-127-HG

379,127,OG,104,HZ1,127-OG-104-HZ1

380,127,OG,455,HE,127-OG-455-HE

381,127,OG,104,HZ2,127-OG-104-HZ2

382,127,OG,104,HZ3,127-OG-104-HZ3

383,127,O,104,HZ3,127-O-104-HZ3

384,127,O,104,HZ2,127-O-104-HZ2

385,127,O,104,HZ1,127-O-104-HZ1

386,127,OG,455,HH21,127-OG-455-HH21

387,128,O,131,H,128-O-131-H

388,128,O,132,H,128-O-132-H

389,129,O,297,HD22,129-O-297-HD22

390,129,O,133,H,129-O-133-H

391,129,O,297,HD21,129-O-297-HD21

392,129,O,299,HD22,129-O-299-HD22

393,130,O,134,H,130-O-134-H

394,130,O,133,H,130-O-133-H

395,131,O,134,H,131-O-134-H

396,131,O,135,H,131-O-135-H

397,132,O,135,H,132-O-135-H

398,132,O,136,H,132-O-136-H

399,133,O,136,HE,133-O-136-HE

400,133,O,136,HH21,133-O-136-HH21

401,133,O,136,H,133-O-136-H

402,133,O,136,HH11,133-O-136-HH11

403,133,O,137,H,133-O-137-H

404,134,O,138,H,134-O-138-H

405,135,O,139,H,135-O-139-H

406,135,O,138,H,135-O-138-H

407,135,O,139,HE21,135-O-139-HE21

408,136,O,140,H,136-O-140-H

409,136,O,140,HD22,136-O-140-HD22

410,137,O,141,H,137-O-141-H

411,138,O,141,H,138-O-141-H

412,138,O,142,H,138-O-142-H

413,139,OE1,143,HZ1,139-OE1-143-HZ1

414,139,OE1,143,HZ3,139-OE1-143-HZ3

415,139,O,139,HE21,139-O-139-HE21

416,139,OE1,136,HH11,139-OE1-136-HH11

417,139,NE2,135,HH21,139-NE2-135-HH21

418,139,OE1,135,HE,139-OE1-135-HE

419,139,OE1,135,HH21,139-OE1-135-HH21

420,139,OE1,143,HZ2,139-OE1-143-HZ2

421,139,O,143,H,139-O-143-H

422,139,OE1,140,HD22,139-OE1-140-HD22

423,139,O,143,HZ2,139-O-143-HZ2

424,139,OE1,136,HH12,139-OE1-136-HH12

425,140,OD1,139,HE21,140-OD1-139-HE21

426,140,OD1,136,HE,140-OD1-136-HE

427,140,OD1,143,HZ3,140-OD1-143-HZ3

428,140,OD1,136,HH21,140-OD1-136-HH21

429,140,OD1,288,HE2,140-OD1-288-HE2

430,140,OD1,136,HH11,140-OD1-136-HH11

431,140,O,144,HG,140-O-144-HG

432,140,O,144,H,140-O-144-H

433,140,OD1,143,HZ2,140-OD1-143-HZ2

434,140,OD1,143,HZ1,140-OD1-143-HZ1

435,140,O,140,HD22,140-O-140-HD22

436,141,O,144,HG,141-O-144-HG

437,141,O,145,H,141-O-145-H

438,141,O,144,H,141-O-144-H

439,142,O,146,HG,142-O-146-HG

440,142,O,146,H,142-O-146-H

441,142,O,147,H,142-O-147-H

442,142,O,145,H,142-O-145-H

443,143,O,148,H,143-O-148-H

444,143,O,146,H,143-O-146-H

445,143,O,147,H,143-O-147-H

446,144,O,144,HG,144-O-144-HG

447,145,O,162,HE2,145-O-162-HE2

448,145,O,146,HG,145-O-146-HG

449,146,O,148,H,146-O-148-H

450,146,OG,147,H,146-OG-147-H

451,146,O,159,H,146-O-159-H

452,146,O,158,H,146-O-158-H

453,148,O,158,H,148-O-158-H

454,149,OG,150,H,149-OG-150-H

455,149,OG,156,H,149-OG-156-H

456,149,O,158,HH,149-O-158-HH

457,150,OD1,158,HH,150-OD1-158-HH

458,150,OD2,156,HG,150-OD2-156-HG

459,150,OD2,153,HG,150-OD2-153-HG

460,150,OD1,153,H,150-OD1-153-H

461,150,OD1,156,HG,150-OD1-156-HG

462,150,OD1,153,HG,150-OD1-153-HG

463,150,OD2,154,H,150-OD2-154-H

464,150,O,149,HG,150-O-149-HG

465,150,OD2,153,H,150-OD2-153-H

466,150,OD2,152,H,150-OD2-152-H

467,150,OD2,158,HH,150-OD2-158-HH

468,150,O,154,HG,150-O-154-HG

469,150,OD1,152,H,150-OD1-152-H

470,150,O,152,H,150-O-152-H

471,150,O,154,H,150-O-154-H

472,150,O,153,H,150-O-153-H

473,150,OD1,150,H,150-OD1-150-H

474,150,O,153,HG,150-O-153-HG

475,151,O,154,HG,151-O-154-HG

476,151,O,153,H,151-O-153-H

477,152,O,153,HG,152-O-153-HG

478,152,O,154,H,152-O-154-H

479,153,OG,154,H,153-OG-154-H

480,153,OG,155,HG1,153-OG-155-HG1

481,153,OG,155,H,153-OG-155-H

482,153,O,155,H,153-O-155-H

483,153,O,155,HG1,153-O-155-HG1

484,153,O,156,H,153-O-156-H

485,153,OG,358,HH12,153-OG-358-HH12

486,153,OG,156,H,153-OG-156-H

487,153,O,149,HG,153-O-149-HG

488,153,OG,156,HG,153-OG-156-HG

489,153,O,156,HG,153-O-156-HG

490,154,OG,155,H,154-OG-155-H

491,154,OG,155,HG1,154-OG-155-HG1

492,154,OG,358,HH12,154-OG-358-HH12

493,154,O,155,HG1,154-O-155-HG1

494,154,O,154,HG,154-O-154-HG

495,154,O,149,HG,154-O-149-HG

496,154,O,156,H,154-O-156-H

497,155,O,360,HG,155-O-360-HG

498,155,O,157,H,155-O-157-H

499,155,OG1,358,HH12,155-OG1-358-HH12

500,155,O,360,H,155-O-360-H

501,155,O,155,HG1,155-O-155-HG1

502,155,OG1,360,HG,155-OG1-360-HG

503,155,OG1,153,HG,155-OG1-153-HG

504,155,OG1,358,HH11,155-OG1-358-HH11

505,155,O,149,HG,155-O-149-HG

506,156,O,153,HG,156-O-153-HG

507,156,OG,155,HG1,156-OG-155-HG1

508,156,OG,358,HH12,156-OG-358-HH12

509,156,OG,356,HH22,156-OG-356-HH22

510,156,OG,153,HG,156-OG-153-HG

511,156,O,150,H,156-O-150-H

512,156,OG,356,HH21,156-OG-356-HH21

513,156,OG,150,H,156-OG-150-H

514,156,O,149,HG,156-O-149-HG

515,156,OG,157,H,156-OG-157-H

516,157,O,160,H,157-O-160-H

517,157,O,156,HG,157-O-156-HG

518,157,O,161,H,157-O-161-H

519,158,O,162,H,158-O-162-H

520,158,O,161,H,158-O-161-H

521,159,O,162,H,159-O-162-H

522,159,O,163,H,159-O-163-H

523,160,O,164,H,160-O-164-H

524,160,OE1,358,HH11,160-OE1-358-HH11

525,160,OE2,156,HG,160-OE2-156-HG

526,160,O,164,HG,160-O-164-HG

527,160,OE1,356,HH12,160-OE1-356-HH12

528,160,OE2,164,HG,160-OE2-164-HG

529,160,OE1,356,HH22,160-OE1-356-HH22

530,160,OE1,359,H,160-OE1-359-H

531,160,OE1,157,H,160-OE1-157-H

532,160,OE1,156,HG,160-OE1-156-HG

533,160,OE2,359,H,160-OE2-359-H

534,160,OE2,356,HH21,160-OE2-356-HH21

535,160,OE2,356,HH22,160-OE2-356-HH22

536,160,OE1,356,HH21,160-OE1-356-HH21

537,160,OE2,157,H,160-OE2-157-H

538,160,OE2,358,HH11,160-OE2-358-HH11

539,160,OE2,356,HH12,160-OE2-356-HH12

540,160,OE2,356,HE,160-OE2-356-HE

541,160,OE2,356,HH11,160-OE2-356-HH11

542,160,OE1,356,HE,160-OE1-356-HE

543,161,O,165,HZ3,161-O-165-HZ3

544,161,OE1,165,HZ1,161-OE1-165-HZ1

545,161,O,165,HZ2,161-O-165-HZ2

546,161,OE2,165,HZ3,161-OE2-165-HZ3

547,161,OE1,165,HZ2,161-OE1-165-HZ2

548,161,OE2,165,HZ2,161-OE2-165-HZ2

549,161,OE2,156,HG,161-OE2-156-HG

550,161,OE2,356,HE,161-OE2-356-HE

551,161,OE1,356,HH22,161-OE1-356-HH22

552,161,OE1,356,HE,161-OE1-356-HE

553,161,OE2,356,HH21,161-OE2-356-HH21

554,161,OE1,358,HH12,161-OE1-358-HH12

555,161,OE1,356,HH21,161-OE1-356-HH21

556,161,OE2,153,HG,161-OE2-153-HG

557,161,OE1,153,HG,161-OE1-153-HG

558,161,OE2,356,HH12,161-OE2-356-HH12

559,161,OE2,358,HH22,161-OE2-358-HH22

560,161,OE2,358,HH12,161-OE2-358-HH12

561,161,OE2,356,HH22,161-OE2-356-HH22

562,161,OE1,356,HH12,161-OE1-356-HH12

563,161,O,165,HZ1,161-O-165-HZ1

564,161,OE1,358,HH22,161-OE1-358-HH22

565,161,OE1,156,HG,161-OE1-156-HG

566,161,OE2,165,HZ1,161-OE2-165-HZ1

567,161,O,164,H,161-O-164-H

568,161,O,165,H,161-O-165-H

569,161,OE1,356,HH11,161-OE1-356-HH11

570,161,OE1,165,HZ3,161-OE1-165-HZ3

571,162,ND1,165,HZ2,162-ND1-165-HZ2

572,162,ND1,165,HZ3,162-ND1-165-HZ3

573,162,O,165,H,162-O-165-H

574,162,O,166,H,162-O-166-H

575,162,ND1,165,HZ1,162-ND1-165-HZ1

576,163,O,167,H,163-O-167-H

577,163,O,166,H,163-O-166-H

578,164,OG,356,HH21,164-OG-356-HH21

579,164,O,168,H,164-O-168-H

580,164,OG,356,HE,164-OG-356-HE

581,164,O,167,H,164-O-167-H

582,164,OG,356,HH11,164-OG-356-HH11

583,165,O,168,H,165-O-168-H

584,165,O,169,H,165-O-169-H

585,166,OE2,165,HZ1,166-OE2-165-HZ1

586,166,OE2,165,HZ2,166-OE2-165-HZ2

587,166,OE2,206,HH21,166-OE2-206-HH21

588,166,OE1,209,HE2,166-OE1-209-HE2

589,166,OE1,206,HE,166-OE1-206-HE

590,166,O,169,H,166-O-169-H

591,166,O,170,H,166-O-170-H

592,166,OE1,193,HG,166-OE1-193-HG

593,166,O,193,HG,166-O-193-HG

594,166,OE2,206,HE,166-OE2-206-HE

595,166,OE1,206,HH21,166-OE1-206-HH21

596,166,OE1,196,HD22,166-OE1-196-HD22

597,166,OE2,165,HZ3,166-OE2-165-HZ3

598,167,O,170,H,167-O-170-H

599,167,O,171,H,167-O-171-H

600,168,O,172,H,168-O-172-H

601,168,OE1,477,HH12,168-OE1-477-HH12

602,168,OE1,172,HG,168-OE1-172-HG

603,168,OE1,356,HH21,168-OE1-356-HH21

604,168,O,171,H,168-O-171-H

605,168,OE1,165,HZ3,168-OE1-165-HZ3

606,168,OE1,477,HH22,168-OE1-477-HH22

607,168,OE1,477,HH11,168-OE1-477-HH11

608,168,OE2,477,HH22,168-OE2-477-HH22

609,168,OE2,477,HH11,168-OE2-477-HH11

610,168,OE2,356,HH11,168-OE2-356-HH11

611,168,OE2,356,HE,168-OE2-356-HE

612,168,OE2,356,HH21,168-OE2-356-HH21

613,168,O,172,HG,168-O-172-HG

614,168,OE2,477,HH12,168-OE2-477-HH12

615,169,O,172,HG,169-O-172-HG

616,169,O,173,H,169-O-173-H

617,169,O,172,H,169-O-172-H

618,169,O,173,HG1,169-O-173-HG1

619,170,O,173,H,170-O-173-H

620,170,O,174,H,170-O-174-H

621,171,O,175,H,171-O-175-H

622,171,O,174,H,171-O-174-H

623,171,O,175,HE21,171-O-175-HE21

624,172,O,176,H,172-O-176-H

625,172,O,175,HE21,172-O-175-HE21

626,173,O,176,H,173-O-176-H

627,173,OG1,209,HE2,173-OG1-209-HE2

628,173,O,177,H,173-O-177-H

629,174,O,178,H,174-O-178-H

630,174,O,177,H,174-O-177-H

631,175,O,179,H,175-O-179-H

632,175,O,175,HE21,175-O-175-HE21

633,175,O,178,H,175-O-178-H

634,176,OE1,209,HE2,176-OE1-209-HE2

635,176,O,179,H,176-O-179-H

636,176,OE1,175,HE21,176-OE1-175-HE21

637,176,O,180,H,176-O-180-H

638,176,OE2,209,HE2,176-OE2-209-HE2

639,176,OE2,175,HE21,176-OE2-175-HE21

640,177,O,182,H,177-O-182-H

641,177,O,180,H,177-O-180-H

642,178,O,180,H,178-O-180-H

643,181,O,183,H,181-O-183-H

644,182,O,507,HE21,182-O-507-HE21

645,182,O,507,HE22,182-O-507-HE22

646,183,ND1,507,HE21,183-ND1-507-HE21

647,183,ND1,183,H,183-ND1-183-H

648,184,O,505,H,184-O-505-H

649,184,O,506,H,184-O-506-H

650,185,O,188,H,185-O-188-H

651,185,OD1,188,H,185-OD1-188-H

652,185,OD1,188,HH11,185-OD1-188-HH11

653,185,OD1,187,H,185-OD1-187-H

654,186,O,190,H,186-O-190-H

655,186,O,327,HG,186-O-327-HG

656,186,O,189,H,186-O-189-H

657,187,OH,222,HD22,187-OH-222-HD22

658,187,OH,499,HZ1,187-OH-499-HZ1

659,187,O,189,H,187-O-189-H

660,187,O,191,H,187-O-191-H

661,187,OH,499,HZ3,187-OH-499-HZ3

662,187,O,190,H,187-O-190-H

663,187,OH,499,HZ2,187-OH-499-HZ2

664,188,O,192,H,188-O-192-H

665,188,O,219,HD21,188-O-219-HD21

666,189,O,192,H,189-O-192-H

667,189,O,209,HE2,189-O-209-HE2

668,189,O,193,H,189-O-193-H

669,189,OH,185,H,189-OH-185-H

670,189,OH,188,HH11,189-OH-188-HH11

671,189,OH,188,HE,189-OH-188-HE

672,189,O,193,HG,189-O-193-HG

673,189,OH,188,HH21,189-OH-188-HH21

674,190,O,193,H,190-O-193-H

675,190,O,193,HG,190-O-193-HG

676,190,O,194,H,190-O-194-H

677,191,O,195,H,191-O-195-H

678,191,O,195,HG1,191-O-195-HG1

679,192,O,195,H,192-O-195-H

680,192,O,196,HD22,192-O-196-HD22

681,192,O,196,H,192-O-196-H

682,193,OG,209,HE2,193-OG-209-HE2

683,193,O,196,H,193-O-196-H

684,193,O,197,H,193-O-197-H

685,194,O,198,H,194-O-198-H

686,194,O,197,H,194-O-197-H

687,195,O,199,H,195-O-199-H

688,196,O,200,H,196-O-200-H

689,196,OD1,207,H,196-OD1-207-H

690,197,O,201,H,197-O-201-H

691,197,O,200,H,197-O-200-H

692,198,O,202,H,198-O-202-H

693,198,O,201,H,198-O-201-H

694,199,O,204,H,199-O-204-H

695,199,O,203,H,199-O-203-H

696,199,O,205,H,199-O-205-H

697,200,O,204,H,200-O-204-H

698,201,O,281,H,201-O-281-H

699,202,O,281,H,202-O-281-H

700,202,O,282,HG1,202-O-282-HG1

701,202,O,282,H,202-O-282-H

702,203,O,205,H,203-O-205-H

703,203,O,205,HE,203-O-205-HE

704,203,O,205,HH11,203-O-205-HH11

705,204,O,205,HH11,204-O-205-HH11

706,204,O,206,H,204-O-206-H

707,205,O,207,HH,205-O-207-HH

708,206,O,205,HH21,206-O-205-HH21

709,206,O,205,HH12,206-O-205-HH12

710,207,OH,205,HE,207-OH-205-HE

711,207,OH,205,HH11,207-OH-205-HH11

712,207,O,196,HD21,207-O-196-HD21

713,208,OD2,210,HD22,208-OD2-210-HD22

714,208,OD2,211,HE2,208-OD2-211-HE2

715,208,O,211,H,208-O-211-H

716,208,OD2,206,HH22,208-OD2-206-HH22

717,208,OD1,206,HH12,208-OD1-206-HH12

718,208,OD1,210,HD21,208-OD1-210-HD21

719,208,OD2,209,H,208-OD2-209-H

720,208,OD2,206,HH12,208-OD2-206-HH12

721,208,O,210,H,208-O-210-H

722,208,OD1,209,H,208-OD1-209-H

723,208,OD2,208,H,208-OD2-208-H

724,208,OD1,210,HD22,208-OD1-210-HD22

725,208,OD1,210,H,208-OD1-210-H

726,208,OD1,206,HH22,208-OD1-206-HH22

727,208,OD2,210,H,208-OD2-210-H

728,208,OD1,206,HH11,208-OD1-206-HH11

729,208,OD2,210,HD21,208-OD2-210-HD21

730,208,OD1,211,HE2,208-OD1-211-HE2

731,209,ND1,209,H,209-ND1-209-H

732,209,O,188,HH21,209-O-188-HH21

733,209,ND1,173,HG1,209-ND1-173-HG1

734,209,O,188,HE,209-O-188-HE

735,209,ND1,210,HD22,209-ND1-210-HD22

736,209,ND1,193,HG,209-ND1-193-HG

737,209,ND1,210,H,209-ND1-210-H

738,209,O,210,HD22,209-O-210-HD22

739,209,ND1,188,HE,209-ND1-188-HE

740,209,ND1,188,HH21,209-ND1-188-HH21

741,210,OD1,210,H,210-OD1-210-H

742,210,O,212,HE21,210-O-212-HE21

743,210,O,210,HD22,210-O-210-HD22

744,211,ND1,214,H,211-ND1-214-H

745,211,ND1,212,H,211-ND1-212-H

746,211,O,214,H,211-O-214-H

747,211,O,215,H,211-O-215-H

748,211,ND1,208,H,211-ND1-208-H

749,211,ND1,207,HH,211-ND1-207-HH

750,211,ND1,213,H,211-ND1-213-H

751,212,O,212,HE21,212-O-212-HE21

752,212,OE1,212,H,212-OE1-212-H

753,212,OE1,219,HD21,212-OE1-219-HD21

754,212,O,216,HG,212-O-216-HG

755,212,O,216,H,212-O-216-H

756,213,O,257,HZ1,213-O-257-HZ1

757,213,OE1,264,HZ1,213-OE1-264-HZ1

758,213,O,217,H,213-O-217-H

759,213,OE2,207,HH,213-OE2-207-HH

760,213,OE2,212,HE21,213-OE2-212-HE21

761,213,OE1,212,HE21,213-OE1-212-HE21

762,213,OE2,264,HZ3,213-OE2-264-HZ3

763,213,O,257,HZ2,213-O-257-HZ2

764,213,OE1,264,HZ3,213-OE1-264-HZ3

765,213,O,216,HG,213-O-216-HG

766,213,OE2,264,HZ2,213-OE2-264-HZ2

767,213,OE1,257,HZ1,213-OE1-257-HZ1

768,213,OE1,213,H,213-OE1-213-H

769,213,O,216,H,213-O-216-H

770,213,OE1,264,HZ2,213-OE1-264-HZ2

771,213,OE1,257,HZ2,213-OE1-257-HZ2

772,213,OE1,207,HH,213-OE1-207-HH

773,213,OE1,212,HE22,213-OE1-212-HE22

774,213,OE2,257,HZ1,213-OE2-257-HZ1

775,213,OE2,264,HZ1,213-OE2-264-HZ1

776,213,OE2,213,H,213-OE2-213-H

777,213,OE2,212,HE22,213-OE2-212-HE22

778,213,OE1,257,HZ3,213-OE1-257-HZ3

779,213,OE2,257,HZ3,213-OE2-257-HZ3

780,213,OE2,211,HE2,213-OE2-211-HE2

781,213,OE1,260,HG,213-OE1-260-HG

782,213,O,257,HZ3,213-O-257-HZ3

783,213,OE2,257,HZ2,213-OE2-257-HZ2

784,213,OE2,260,HG,213-OE2-260-HG

785,214,O,217,H,214-O-217-H

786,214,O,219,HD22,214-O-219-HD22

787,214,O,218,H,214-O-218-H

788,215,O,219,HD22,215-O-219-HD22

789,215,O,220,H,215-O-220-H

790,215,O,188,HH12,215-O-188-HH12

791,215,O,218,H,215-O-218-H

792,215,O,219,H,215-O-219-H

793,216,OG,257,HZ3,216-OG-257-HZ3

794,216,OG,212,HE21,216-OG-212-HE21

795,216,OG,257,HZ2,216-OG-257-HZ2

796,216,O,257,HZ2,216-O-257-HZ2

797,216,O,220,H,216-O-220-H

798,216,O,219,H,216-O-219-H

799,216,O,257,HZ1,216-O-257-HZ1

800,216,O,257,HZ3,216-O-257-HZ3

801,216,O,216,HG,216-O-216-HG

802,216,OG,257,HZ1,216-OG-257-HZ1

803,217,O,222,HD21,217-O-222-HD21

804,217,O,222,HD22,217-O-222-HD22

805,217,O,220,H,217-O-220-H

806,218,O,220,H,218-O-220-H

807,218,O,222,HD21,218-O-222-HD21

808,218,O,219,HD22,218-O-219-HD22

809,218,O,187,HH,218-O-187-HH

810,218,O,222,HD22,218-O-222-HD22

811,219,OD1,188,HH12,219-OD1-188-HH12

812,219,O,222,HD21,219-O-222-HD21

813,219,OD1,188,HH22,219-OD1-188-HH22

814,219,OD1,188,HH11,219-OD1-188-HH11

815,219,O,222,H,219-O-222-H

816,219,O,219,HD22,219-O-219-HD22

817,219,OD1,188,HH21,219-OD1-188-HH21

818,219,O,222,HD22,219-O-222-HD22

819,220,O,222,HD21,220-O-222-HD21

820,220,O,257,HZ3,220-O-257-HZ3

821,220,O,222,H,220-O-222-H

822,221,O,224,H,221-O-224-H

823,221,OD1,257,HZ1,221-OD1-257-HZ1

824,221,OD1,257,HZ3,221-OD1-257-HZ3

825,221,OD1,257,HZ2,221-OD1-257-HZ2

826,221,O,223,H,221-O-223-H

827,222,OD1,187,HH,222-OD1-187-HH

828,222,OD1,224,H,222-OD1-224-H

829,222,OD1,222,H,222-OD1-222-H

830,222,O,224,H,222-O-224-H

831,222,OD1,220,H,222-OD1-220-H

832,222,OD1,188,HH22,222-OD1-188-HH22

833,222,O,223,HD22,222-O-223-HD22

834,222,O,222,HD21,222-O-222-HD21

835,222,OD1,499,HZ3,222-OD1-499-HZ3

836,222,OD1,223,H,222-OD1-223-H

837,222,OD1,499,HZ1,222-OD1-499-HZ1

838,222,OD1,188,HH21,222-OD1-188-HH21

839,223,O,227,H,223-O-227-H

840,223,O,226,H,223-O-226-H

841,223,OD1,222,HD21,223-OD1-222-HD21

842,223,O,223,HD22,223-O-223-HD22

843,223,O,247,HG,223-O-247-HG

844,223,OD1,225,H,223-OD1-225-H

845,223,OD1,223,H,223-OD1-223-H

846,223,OD1,247,HG,223-OD1-247-HG

847,224,O,227,H,224-O-227-H

848,224,O,228,H,224-O-228-H

849,224,O,247,HG,224-O-247-HG

850,225,O,227,H,225-O-227-H

851,225,O,229,H,225-O-229-H

852,225,O,496,H,225-O-496-H

853,225,O,228,H,225-O-228-H

854,226,OE1,232,HD21,226-OE1-232-HD21

855,226,O,232,HD22,226-O-232-HD22

856,226,OE2,241,HH21,226-OE2-241-HH21

857,226,OE2,223,HD22,226-OE2-223-HD22

858,226,OE1,57,HZ2,226-OE1-57-HZ2

859,226,O,229,H,226-O-229-H

860,226,OE2,245,HD21,226-OE2-245-HD21

861,226,OE2,232,HD21,226-OE2-232-HD21

862,226,OE1,247,HG,226-OE1-247-HG

863,226,OE2,223,HD21,226-OE2-223-HD21

864,226,OE2,57,HZ2,226-OE2-57-HZ2

865,226,OE2,226,H,226-OE2-226-H

866,226,OE1,226,H,226-OE1-226-H

867,226,OE1,223,HD21,226-OE1-223-HD21

868,226,OE1,57,HZ1,226-OE1-57-HZ1

869,226,OE1,223,HD22,226-OE1-223-HD22

870,226,OE1,245,HD21,226-OE1-245-HD21

871,226,O,241,HH21,226-O-241-HH21

872,226,OE1,57,HZ3,226-OE1-57-HZ3

873,226,OE2,247,HG,226-OE2-247-HG

874,226,OE2,57,HZ3,226-OE2-57-HZ3

875,226,OE2,57,HZ1,226-OE2-57-HZ1

876,226,O,232,HD21,226-O-232-HD21

877,227,O,232,HD21,227-O-232-HD21

878,227,O,111,HG1,227-O-111-HG1

879,227,O,232,HD22,227-O-232-HD22

880,227,O,229,H,227-O-229-H

881,227,O,241,HH21,227-O-241-HH21

882,228,O,232,HD22,228-O-232-HD22

883,228,O,496,H,228-O-496-H

884,228,O,230,HG,228-O-230-HG

885,229,O,232,HD21,229-O-232-HD21

886,229,O,494,HH,229-O-494-HH

887,229,O,232,H,229-O-232-H

888,229,O,232,HD22,229-O-232-HD22

889,229,O,231,H,229-O-231-H

890,230,O,494,HH,230-O-494-HH

891,230,O,230,HG,230-O-230-HG

892,230,O,232,H,230-O-232-H

893,230,OG,494,HH,230-OG-494-HH

894,232,O,236,H,232-O-236-H

895,232,OD1,234,H,232-OD1-234-H

896,232,O,235,H,232-O-235-H

897,232,OD1,235,H,232-OD1-235-H

898,232,ND2,235,H,232-ND2-235-H

899,233,O,236,H,233-O-236-H

900,233,O,237,H,233-O-237-H

901,233,O,235,H,233-O-235-H

902,234,O,241,HH11,234-O-241-HH11

903,234,O,241,HE,234-O-241-HE

904,234,O,241,HH21,234-O-241-HH21

905,234,O,241,HH12,234-O-241-HH12

906,235,OD2,241,HH11,235-OD2-241-HH11

907,235,O,241,HH11,235-O-241-HH11

908,235,OD2,241,HH21,235-OD2-241-HH21

909,235,OD2,241,HH12,235-OD2-241-HH12

910,235,OD1,241,HH11,235-OD1-241-HH11

911,235,OD1,241,HH12,235-OD1-241-HH12

912,235,OD1,111,H,235-OD1-111-H

913,235,OD1,241,HE,235-OD1-241-HE

914,235,OD1,241,HH21,235-OD1-241-HH21

915,235,O,241,HH12,235-O-241-HH12

916,235,OD2,241,HE,235-OD2-241-HE

917,235,O,241,HH21,235-O-241-HH21

918,235,OD2,232,HD22,235-OD2-232-HD22

919,235,OD1,232,HD21,235-OD1-232-HD21

920,235,OD2,232,HD21,235-OD2-232-HD21

921,235,OD1,110,H,235-OD1-110-H

922,235,OD2,110,H,235-OD2-110-H

923,235,OD2,111,HG1,235-OD2-111-HG1

924,235,OD2,241,HH22,235-OD2-241-HH22

925,235,OD1,232,HD22,235-OD1-232-HD22

926,235,OD2,111,H,235-OD2-111-H

927,235,O,241,HE,235-O-241-HE

928,235,OD1,241,HH22,235-OD1-241-HH22

929,235,OD1,111,HG1,235-OD1-111-HG1

930,236,O,241,HH21,236-O-241-HH21

931,236,O,241,HH11,236-O-241-HH11

932,236,O,241,HE,236-O-241-HE

933,236,O,81,HG1,236-O-81-HG1

934,237,O,241,H,237-O-241-H

935,237,O,240,H,237-O-240-H

936,237,O,239,H,237-O-239-H

937,238,O,241,H,238-O-241-H

938,238,O,242,H,238-O-242-H

939,239,O,242,H,239-O-242-H

940,239,O,243,H,239-O-243-H

941,240,O,245,HD22,240-O-245-HD22

942,240,O,243,H,240-O-243-H

943,241,O,241,HH11,241-O-241-HH11

944,242,OH,241,HH21,242-OH-241-HH21

945,243,O,245,H,243-O-245-H

946,244,O,249,HD21,244-O-249-HD21

947,245,OD1,247,H,245-OD1-247-H

948,245,O,248,H,245-O-248-H

949,245,OD1,247,HG,245-OD1-247-HG

950,245,O,249,H,245-O-249-H

951,245,OD1,248,H,245-OD1-248-H

952,245,O,249,HD22,245-O-249-HD22

953,246,O,249,H,246-O-249-H

954,246,O,249,HD22,246-O-249-HD22

955,246,O,223,HD21,246-O-223-HD21

956,246,O,250,H,246-O-250-H
957,247,OG,245,HD21,247-OG-245-HD21
958,247,O,250,H,247-O-250-H
959,247,OG,248,H,247-OG-248-H
960,247,OG,223,HD21,247-OG-223-HD21
961,247,O,223,HD21,247-O-223-HD21
962,247,OG,223,HD22,247-OG-223-HD22
963,247,O,251,H,247-O-251-H
964,248,O,252,H,248-O-252-H
965,248,O,251,H,248-O-251-H
966,249,OD1,245,H,249-OD1-245-H
967,249,O,249,HD22,249-O-249-HD22
968,249,OD1,252,HZ1,249-OD1-252-HZ1
969,249,O,253,H,249-O-253-H
970,249,O,252,H,249-O-252-H
971,249,OD1,252,HZ3,249-OD1-252-HZ3
972,249,OD1,252,HZ2,249-OD1-252-HZ2
973,250,O,254,H,250-O-254-H
974,250,O,221,HD22,250-O-221-HD22
975,250,O,253,H,250-O-253-H
976,251,O,255,H,251-O-255-H
977,251,O,255,HD22,251-O-255-HD22
978,252,O,256,H,252-O-256-H
979,252,O,255,H,252-O-255-H
980,252,O,254,H,252-O-254-H
981,253,OD1,252,HZ1,253-OD1-252-HZ1
982,253,OD1,252,HZ3,253-OD1-252-HZ3
983,253,O,256,H,253-O-256-H
984,253,O,257,HZ1,253-O-257-HZ1
985,253,O,257,HZ2,253-O-257-HZ2
986,253,O,257,HZ3,253-O-257-HZ3
987,253,OD2,249,HD21,253-OD2-249-HD21
988,253,OD2,221,HD22,253-OD2-221-HD22
989,253,O,257,H,253-O-257-H
990,253,OD2,252,HZ2,253-OD2-252-HZ2
991,253,OD2,257,HZ3,253-OD2-257-HZ3
992,253,OD2,221,HD21,253-OD2-221-HD21
993,253,OD1,252,HZ2,253-OD1-252-HZ2
994,253,OD1,221,HD21,253-OD1-221-HD21
995,253,OD2,252,HZ1,253-OD2-252-HZ1
996,253,OD2,257,HZ2,253-OD2-257-HZ2
997,253,OD2,252,HZ3,253-OD2-252-HZ3
998,253,OD1,221,HD22,253-OD1-221-HD22
999,253,OD1,257,HZ3,253-OD1-257-HZ3
1000,253,OD1,257,HZ2,253-OD1-257-HZ2
1001,253,OD2,257,HZ1,253-OD2-257-HZ1
1002,253,OD1,257,HZ1,253-OD1-257-HZ1
1003,254,O,258,H,254-O-258-H
1004,254,O,257,H,254-O-257-H
1005,255,O,259,H,255-O-259-H
1006,255,OD1,116,H,255-OD1-116-H
1007,256,OE2,257,HZ2,256-OE2-257-HZ2
1008,256,OE1,257,HZ1,256-OE1-257-HZ1
1009,256,OE2,260,HG,256-OE2-260-HG
1010,256,O,260,H,256-O-260-H
1011,256,O,260,HG,256-O-260-HG
1012,256,OE1,257,HZ2,256-OE1-257-HZ2
1013,256,OE2,257,HZ3,256-OE2-257-HZ3
1014,256,OE1,252,HZ2,256-OE1-252-HZ2
1015,256,OE1,260,HG,256-OE1-260-HG
1016,256,OE2,252,HZ2,256-OE2-252-HZ2
1017,256,OE1,252,HZ1,256-OE1-252-HZ1
1018,256,OE2,252,HZ3,256-OE2-252-HZ3
1019,256,OE2,257,HZ1,256-OE2-257-HZ1
1020,256,OE2,252,HZ1,256-OE2-252-HZ1
1021,256,OE1,257,HZ3,256-OE1-257-HZ3
1022,256,OE1,252,HZ3,256-OE1-252-HZ3
1023,257,O,261,H,257-O-261-H
1024,257,O,260,HG,257-O-260-HG
1025,258,O,261,H,258-O-261-H
1026,258,O,262,H,258-O-262-H
1027,259,O,263,H,259-O-263-H
1028,259,O,262,H,259-O-262-H
1029,259,OH,263,HE21,259-OH-263-HE21
1030,259,OH,263,HE22,259-OH-263-HE22
1031,259,O,263,HE21,259-O-263-HE21
1032,260,O,263,HE21,260-O-263-HE21
1033,260,OG,264,HZ3,260-OG-264-HZ3
1034,260,O,264,HZ3,260-O-264-HZ3
1035,260,O,264,H,260-O-264-H

1036,260,OG,264,HZ1,260-OG-264-HZ1

1037,260,O,264,HZ2,260-O-264-HZ2

1038,260,OG,264,HZ2,260-OG-264-HZ2

1039,260,O,264,HZ1,260-O-264-HZ1

1040,261,O,265,H,261-O-265-H

1041,261,O,264,H,261-O-264-H

1042,262,O,265,H,262-O-265-H

1043,262,O,266,H,262-O-266-H

1044,263,O,267,HZ2,263-O-267-HZ2

1045,263,OE1,259,HH,263-OE1-259-HH

1046,263,O,267,H,263-O-267-H

1047,263,O,267,HZ1,263-O-267-HZ1

1048,263,O,267,HZ3,263-O-267-HZ3

1049,263,OE1,267,HZ1,263-OE1-267-HZ1

1050,263,O,266,H,263-O-266-H

1051,263,O,263,HE21,263-O-263-HE21

1052,263,OE1,267,HZ2,263-OE1-267-HZ2

1053,263,OE1,267,HZ3,263-OE1-267-HZ3

1054,264,O,268,H,264-O-268-H

1055,264,O,267,HZ1,264-O-267-HZ1

1056,265,O,205,HH22,265-O-205-HH22

1057,265,O,269,H,265-O-269-H

1058,265,O,268,H,265-O-268-H

1059,265,O,205,HE,265-O-205-HE

1060,266,O,270,H,266-O-270-H

1061,266,O,269,H,266-O-269-H

1062,267,O,271,HZ2,267-O-271-HZ2

1063,267,O,271,H,267-O-271-H

1064,267,O,270,H,267-O-270-H

1065,267,O,271,HZ3,267-O-271-HZ3

1066,267,O,271,HZ1,267-O-271-HZ1

1067,268,OE1,205,HE,268-OE1-205-HE

1068,268,OE1,264,HZ1,268-OE1-264-HZ1

1069,268,OE2,271,HZ1,268-OE2-271-HZ1

1070,268,OE2,205,HH22,268-OE2-205-HH22

1071,268,OE2,205,HH12,268-OE2-205-HH12

1072,268,OE2,205,HH21,268-OE2-205-HH21

1073,268,OE2,267,HZ2,268-OE2-267-HZ2

1074,268,OE2,267,HZ3,268-OE2-267-HZ3

1075,268,OE1,267,HZ3,268-OE1-267-HZ3

1076,268,OE1,205,HH21,268-OE1-205-HH21

1077,268,O,272,HG1,268-O-272-HG1

1078,268,OE2,267,HZ1,268-OE2-267-HZ1

1079,268,OE1,264,HZ3,268-OE1-264-HZ3

1080,268,OE2,271,HZ2,268-OE2-271-HZ2

1081,268,OE1,271,HZ1,268-OE1-271-HZ1

1082,268,OE1,271,HZ2,268-OE1-271-HZ2

1083,268,O,272,H,268-O-272-H

1084,268,OE1,271,HZ3,268-OE1-271-HZ3

1085,268,OE1,267,HZ1,268-OE1-267-HZ1

1086,268,OE1,264,HZ2,268-OE1-264-HZ2

1087,268,OE1,267,HZ2,268-OE1-267-HZ2

1088,268,OE2,271,HZ3,268-OE2-271-HZ3

1089,268,OE2,264,HZ2,268-OE2-264-HZ2

1090,268,OE1,205,HH12,268-OE1-205-HH12

1091,268,O,271,H,268-O-271-H

1092,268,O,271,HZ3,268-O-271-HZ3

1093,268,OE2,205,HE,268-OE2-205-HE

1094,268,OE1,205,HH22,268-OE1-205-HH22

1095,269,ND1,205,HH21,269-ND1-205-HH21

1096,269,O,273,H,269-O-273-H

1097,269,O,279,HH12,269-O-279-HH12

1098,269,O,272,HG1,269-O-272-HG1

1099,269,ND1,205,HH22,269-ND1-205-HH22

1100,269,O,279,HH22,269-O-279-HH22

1101,269,O,272,H,269-O-272-H

1102,269,ND1,205,HH12,269-ND1-205-HH12

1103,270,OH,267,HZ1,270-OH-267-HZ1

1104,270,O,273,H,270-O-273-H

1105,270,OH,290,HE22,270-OH-290-HE22

1106,270,OH,290,HE21,270-OH-290-HE21

1107,271,O,272,HG1,271-O-272-HG1

1108,271,O,273,H,271-O-273-H

1109,272,O,279,HH22,272-O-279-HH22

1110,272,OG1,279,HH12,272-OG1-279-HH12

1111,272,OG1,205,HH22,272-OG1-205-HH22

1112,272,OG1,205,HH21,272-OG1-205-HH21

1113,272,O,279,HH12,272-O-279-HH12

1114,272,OG1,279,HH22,272-OG1-279-HH22

1115,272,OG1,205,HH12,272-OG1-205-HH12

1116,273,O,275,H,273-O-275-H

1117,273,O,275,HZ3,273-O-275-HZ3

1118,274,OE2,272,HG1,274-OE2-272-HG1

1119,274,OE1,275,HZ3,274-OE1-275-HZ3

1120,274,OE1,277,HE2,274-OE1-277-HE2

1121,274,OE2,276,H,274-OE2-276-H

1122,274,OE1,275,HZ2,274-OE1-275-HZ2

1123,274,O,276,H,274-O-276-H

1124,274,OE1,275,H,274-OE1-275-H

1125,274,OE1,276,H,274-OE1-276-H

1126,274,OE2,279,HH12,274-OE2-279-HH12

1127,274,OE2,275,HZ1,274-OE2-275-HZ1

1128,274,OE2,275,H,274-OE2-275-H

1129,274,OE1,275,HZ1,274-OE1-275-HZ1

1130,274,OE2,279,HH11,274-OE2-279-HH11

1131,274,O,277,H,274-O-277-H

1132,274,OE2,273,H,274-OE2-273-H

1133,275,O,277,H,275-O-277-H

1134,276,O,278,H,276-O-278-H

1135,277,ND1,278,H,277-ND1-278-H

1136,277,O,279,HE,277-O-279-HE

1137,277,ND1,279,HE,277-ND1-279-HE

1138,277,ND1,279,HH11,277-ND1-279-HH11

1139,280,OD1,269,HE2,280-OD1-269-HE2

1140,280,OD1,282,HG1,280-OD1-282-HG1

1141,280,OD2,269,HE2,280-OD2-269-HE2

1142,280,O,283,H,280-O-283-H

1143,280,O,284,H,280-O-284-H

1144,280,OD1,282,H,280-OD1-282-H

1145,280,OD1,283,H,280-OD1-283-H

1146,280,OD2,205,HH12,280-OD2-205-HH12

1147,280,O,284,HG,280-O-284-HG

1148,281,O,284,H,281-O-284-H

1149,281,O,285,H,281-O-285-H

1150,281,O,284,HG,281-O-284-HG

1151,282,O,286,H,282-O-286-H

1152,282,O,285,H,282-O-285-H

1153,283,OD1,279,HE,283-OD1-279-HE

1154,283,OD2,279,HE,283-OD2-279-HE

1155,283,OD1,279,HH21,283-OD1-279-HH21

1156,283,O,286,H,283-O-286-H

1157,283,OD2,279,H,283-OD2-279-H

1158,283,OD1,279,H,283-OD1-279-H

1159,283,OD2,269,HE2,283-OD2-269-HE2

1160,283,OD2,279,HH21,283-OD2-279-HH21

1161,283,O,287,H,283-O-287-H

1162,284,O,287,H,284-O-287-H

1163,284,O,284,HG,284-O-284-HG

1164,284,O,288,H,284-O-288-H

1165,285,O,288,H,285-O-288-H

1166,285,O,289,H,285-O-289-H

1167,286,O,290,HE21,286-O-290-HE21

1168,286,O,290,H,286-O-290-H

1169,286,O,290,HE22,286-O-290-HE22

1170,286,O,289,H,286-O-289-H

1171,287,OE1,275,HZ2,287-OE1-275-HZ2

1172,287,OE1,290,HE22,287-OE1-290-HE22

1173,287,OE2,290,HE21,287-OE2-290-HE21

1174,287,OE1,275,HZ1,287-OE1-275-HZ1

1175,287,OE1,290,HE21,287-OE1-290-HE21

1176,287,OE1,276,H,287-OE1-276-H

1177,287,O,290,H,287-O-290-H

1178,287,OE2,275,HZ3,287-OE2-275-HZ3

1179,287,O,290,HE21,287-O-290-HE21

1180,287,O,291,H,287-O-291-H

1181,287,OE2,275,HZ2,287-OE2-275-HZ2

1182,287,OE1,275,HZ3,287-OE1-275-HZ3

1183,287,OE2,276,H,287-OE2-276-H

1184,287,OE2,275,HZ1,287-OE2-275-HZ1

1185,288,O,292,H,288-O-292-H

1186,288,O,291,H,288-O-291-H

1187,289,O,293,HE21,289-O-293-HE21

1188,289,O,293,H,289-O-293-H

1189,289,O,292,HZ1,289-O-292-HZ1

1190,289,O,292,HZ2,289-O-292-HZ2

1191,289,O,293,HE22,289-O-293-HE22

1192,289,O,292,HZ3,289-O-292-HZ3

1193,290,O,293,H,290-O-293-H

1194,290,OE1,293,HE21,290-OE1-293-HE21

1195,290,OE1,275,HZ1,290-OE1-275-HZ1

1196,290,OE1,293,HE22,290-OE1-293-HE22

1197,290,O,294,H,290-O-294-H

1198,290,O,292,H,290-O-292-H

1199,290,O,290,HE21,290-O-290-HE21

1200,290,OE1,275,HZ2,290-OE1-275-HZ2

1201,290,OE1,275,HZ3,290-OE1-275-HZ3

1202,290,O,293,HE21,290-O-293-HE21

1203,290,OE1,270,HH,290-OE1-270-HH

1204,290,O,293,HE22,290-O-293-HE22

1205,291,OE1,290,HE21,291-OE1-290-HE21

1206,291,OE2,290,HE21,291-OE2-290-HE21

1207,291,O,294,H,291-O-294-H

1208,291,OE1,293,HE21,291-OE1-293-HE21

1209,291,OE1,290,HE22,291-OE1-290-HE22

1210,291,OE2,275,HZ2,291-OE2-275-HZ2

1211,291,OE2,290,HE22,291-OE2-290-HE22

1212,291,OE1,275,HZ2,291-OE1-275-HZ2

1213,291,O,295,H,291-O-295-H

1214,291,OE1,275,HZ1,291-OE1-275-HZ1

1215,291,OE2,293,HE22,291-OE2-293-HE22

1216,291,OE2,275,HZ3,291-OE2-275-HZ3

1217,291,OE2,293,HE21,291-OE2-293-HE21

1218,291,OE1,293,HE22,291-OE1-293-HE22

1219,291,OE2,275,HZ1,291-OE2-275-HZ1

1220,291,OE1,275,HZ3,291-OE1-275-HZ3

1221,292,O,295,H,292-O-295-H

1222,293,O,293,HE21,293-O-293-HE21

1223,293,OE1,292,HZ1,293-OE1-292-HZ1

1224,293,OE1,290,HE21,293-OE1-290-HE21

1225,293,OE1,292,HZ2,293-OE1-292-HZ2

1226,293,OE1,290,HE22,293-OE1-290-HE22

1227,293,OE1,293,H,293-OE1-293-H

1228,293,O,295,H,293-O-295-H

1229,293,OE1,300,H,293-OE1-300-H

1230,294,O,296,H,294-O-296-H

1231,295,OD2,297,HD21,295-OD2-297-HD21

1232,295,OD2,136,HH12,295-OD2-136-HH12

1233,295,OD2,136,HH22,295-OD2-136-HH22

1234,295,O,297,H,295-O-297-H

1235,295,OD1,136,HH21,295-OD1-136-HH21

1236,295,OD1,136,HH11,295-OD1-136-HH11

1237,295,OD1,288,HE2,295-OD1-288-HE2

1238,295,OD2,136,HH11,295-OD2-136-HH11

1239,295,OD1,136,HE,295-OD1-136-HE

1240,295,OD2,298,H,295-OD2-298-H

1241,295,OD1,136,HH22,295-OD1-136-HH22

1242,295,O,298,H,295-O-298-H

1243,295,OD1,297,HD21,295-OD1-297-HD21

1244,295,O,299,HD22,295-O-299-HD22

1245,295,OD1,136,HH12,295-OD1-136-HH12

1246,295,OD2,296,H,295-OD2-296-H

1247,295,OD1,297,H,295-OD1-297-H

1248,295,OD1,298,H,295-OD1-298-H

1249,295,OD1,296,H,295-OD1-296-H

1250,295,OD2,136,HH21,295-OD2-136-HH21

1251,295,OD2,297,H,295-OD2-297-H

1252,295,OD2,288,HE2,295-OD2-288-HE2

1253,296,OE1,297,HD21,296-OE1-297-HD21

1254,296,O,298,H,296-O-298-H

1255,296,OE2,136,HH22,296-OE2-136-HH22

1256,296,OE1,136,HH22,296-OE1-136-HH22

1257,296,OE1,136,HH12,296-OE1-136-HH12

1258,296,OE2,136,HH12,296-OE2-136-HH12

1259,296,O,299,HD21,296-O-299-HD21

1260,296,OE1,296,H,296-OE1-296-H

1261,296,OE1,297,HD22,296-OE1-297-HD22

1262,296,OE1,136,HH11,296-OE1-136-HH11

1263,296,OE2,297,HD22,296-OE2-297-HD22

1264,296,OE2,296,H,296-OE2-296-H

1265,296,OE2,136,HH21,296-OE2-136-HH21

1266,296,OE1,136,HH21,296-OE1-136-HH21

1267,296,OE2,297,HD21,296-OE2-297-HD21

1268,296,O,297,HD21,296-O-297-HD21

1269,296,OE1,136,HE,296-OE1-136-HE

1270,297,O,299,HD22,297-O-299-HD22

1271,297,O,297,HD21,297-O-297-HD21

1272,297,OD1,136,HH21,297-OD1-136-HH21

1273,297,OD1,136,HH22,297-OD1-136-HH22

1274,297,N,297,HD21,297-N-297-HD21

1275,297,OD1,136,HH11,297-OD1-136-HH11

1276,297,O,136,HH21,297-O-136-HH21

1277,297,OD1,136,HH12,297-OD1-136-HH12

1278,297,OD1,136,HE,297-OD1-136-HE

1279,297,OD1,140,HD21,297-OD1-140-HD21

1280,297,O,299,H,297-O-299-H

1281,297,ND2,136,HH12,297-ND2-136-HH12

1282,299,OD1,301,HE21,299-OD1-301-HE21

1283,299,O,292,HZ2,299-O-292-HZ2

1284,299,OD1,301,H,299-OD1-301-H

1285,299,OD1,302,H,299-OD1-302-H

1286,299,O,302,H,299-O-302-H

1287,299,O,292,HZ1,299-O-292-HZ1

1288,299,OD1,300,H,299-OD1-300-H

1289,299,O,292,HZ3,299-O-292-HZ3

1290,299,ND2,301,H,299-ND2-301-H

1291,300,O,302,H,300-O-302-H

1292,300,O,292,HZ2,300-O-292-HZ2

1293,300,O,292,HZ3,300-O-292-HZ3

1294,300,O,292,HZ1,300-O-292-HZ1

1295,300,O,301,HE21,300-O-301-HE21

1296,301,OE1,299,HD21,301-OE1-299-HD21

1297,301,O,301,HE21,301-O-301-HE21

1298,301,OE1,301,H,301-OE1-301-H

1299,301,OE1,299,HD22,301-OE1-299-HD22

1300,301,OE1,302,H,301-OE1-302-H

1301,301,O,306,HZ2,301-O-306-HZ2

1302,301,OE1,292,HZ3,301-OE1-292-HZ3

1303,301,O,306,HZ1,301-O-306-HZ1

1304,301,O,306,HZ3,301-O-306-HZ3

1305,301,O,303,H,301-O-303-H

1306,302,O,292,HZ2,302-O-292-HZ2

1307,302,O,292,HZ3,302-O-292-HZ3

1308,302,O,292,HZ1,302-O-292-HZ1

1309,303,O,306,H,303-O-306-H

1310,303,OG,306,HZ2,303-OG-306-HZ2

1311,303,O,303,HG,303-O-303-HG

1312,303,O,307,H,303-O-307-H

1313,303,OG,306,H,303-OG-306-H

1314,303,OG,305,H,303-OG-305-H

1315,303,O,292,HZ3,303-O-292-HZ3

1316,303,OG,306,HZ3,303-OG-306-HZ3

1317,303,OG,306,HZ1,303-OG-306-HZ1

1318,304,OD1,293,HE22,304-OD1-293-HE22

1319,304,OD2,292,HZ2,304-OD2-292-HZ2

1320,304,OD1,293,HE21,304-OD1-293-HE21

1321,304,OD2,292,HZ3,304-OD2-292-HZ3

1322,304,OD2,293,HE21,304-OD2-293-HE21

1323,304,O,307,H,304-O-307-H

1324,304,OD2,304,H,304-OD2-304-H

1325,304,OD1,292,HZ1,304-OD1-292-HZ1

1326,304,OD2,292,HZ1,304-OD2-292-HZ1

1327,304,OD2,305,H,304-OD2-305-H

1328,304,OD1,304,H,304-OD1-304-H

1329,304,OD1,305,H,304-OD1-305-H

1330,304,O,308,H,304-O-308-H

1331,304,OD1,292,HZ3,304-OD1-292-HZ3

1332,304,OD2,293,HE22,304-OD2-293-HE22

1333,304,OD1,292,HZ2,304-OD1-292-HZ2

1334,305,OE1,263,HE22,305-OE1-263-HE22

1335,305,OE2,309,HD22,305-OE2-309-HD22

1336,305,OE1,117,HD21,305-OE1-117-HD21

1337,305,OE1,119,HE21,305-OE1-119-HE21

1338,305,O,309,HD21,305-O-309-HD21

1339,305,O,309,H,305-O-309-H

1340,305,O,308,H,305-O-308-H

1341,305,OE1,305,H,305-OE1-305-H

1342,305,O,309,HD22,305-O-309-HD22

1343,305,OE2,306,HZ2,305-OE2-306-HZ2

1344,305,O,119,HE22,305-O-119-HE22

1345,305,OE1,306,HZ2,305-OE1-306-HZ2

1346,305,OE2,306,H,305-OE2-306-H

1347,305,OE2,119,HE21,305-OE2-119-HE21

1348,305,OE2,119,HE22,305-OE2-119-HE22

1349,305,OE2,309,HD21,305-OE2-309-HD21

1350,305,OE2,263,HE22,305-OE2-263-HE22

1351,305,OE1,309,HD22,305-OE1-309-HD22

1352,305,OE1,303,HG,305-OE1-303-HG

1353,305,OE2,306,HZ3,305-OE2-306-HZ3

1354,305,OE1,117,HD22,305-OE1-117-HD22

1355,305,OE2,117,HD22,305-OE2-117-HD22

1356,305,OE1,306,H,305-OE1-306-H

1357,305,OE2,306,HZ1,305-OE2-306-HZ1

1358,305,OE2,303,HG,305-OE2-303-HG

1359,305,OE1,306,HZ1,305-OE1-306-HZ1

1360,305,OE1,309,HD21,305-OE1-309-HD21

1361,305,OE2,259,HH,305-OE2-259-HH

1362,305,OE1,119,HE22,305-OE1-119-HE22

1363,305,OE2,305,H,305-OE2-305-H

1364,305,OE2,117,HD21,305-OE2-117-HD21

1365,305,OE1,306,HZ3,305-OE1-306-HZ3

1366,305,OE1,259,HH,305-OE1-259-HH

1367,306,O,134,HE,306-O-134-HE

1368,306,O,134,HH21,306-O-134-HH21

1369,307,O,310,H,307-O-310-H

1370,308,O,310,H,308-O-310-H

1371,308,O,311,H,308-O-311-H

1372,309,OD1,117,HD22,309-OD1-117-HD22

1373,309,OD1,119,HE21,309-OD1-119-HE21

1374,309,O,312,H,309-O-312-H

1375,309,OD1,263,HE21,309-OD1-263-HE21

1376,309,OD1,134,HH21,309-OD1-134-HH21

1377,309,ND2,117,HD22,309-ND2-117-HD22

1378,309,O,313,H,309-O-313-H

1379,309,OD1,134,HH22,309-OD1-134-HH22

1380,310,O,314,H,310-O-314-H

1381,310,O,313,H,310-O-313-H

1382,311,O,314,H,311-O-314-H

1383,311,O,315,H,311-O-315-H

1384,312,O,316,H,312-O-316-H

1385,313,OD2,121,H,313-OD2-121-H

1386,313,O,516,H10,313-O-516-H10

1387,313,OD2,122,HG,313-OD2-122-HG

1388,313,OD1,116,HG,313-OD1-116-HG

1389,313,OD2,516,H10,313-OD2-516-H10

1390,313,OD2,116,HG,313-OD2-116-HG

1391,313,OD1,121,H,313-OD1-121-H

1392,313,OD1,516,H10,313-OD1-516-H10

1393,313,OD1,120,HG,313-OD1-120-HG

1394,313,OD2,122,H,313-OD2-122-H

1395,313,OD1,122,HG,313-OD1-122-HG

1396,313,OD1,122,H,313-OD1-122-H

1397,313,O,317,H,313-O-317-H

1398,313,OD2,120,HG,313-OD2-120-HG

1399,313,O,316,H,313-O-316-H

1400,314,O,317,H,314-O-317-H

1401,314,O,318,H,314-O-318-H

1402,315,O,319,H,315-O-319-H

1403,316,O,320,H,316-O-320-H

1404,317,O,320,H,317-O-320-H

1405,317,O,321,HG1,317-O-321-HG1

1406,317,O,321,H,317-O-321-H

1407,318,O,323,HG1,318-O-323-HG1

1408,318,O,322,H,318-O-322-H

1409,318,O,321,H,318-O-321-H

1410,318,O,321,HG1,318-O-321-HG1

1411,319,O,323,H,319-O-323-H

1412,319,O,323,HG1,319-O-323-HG1

1413,320,OD2,187,HH,320-OD2-187-HH

1414,320,OD1,499,HZ3,320-OD1-499-HZ3

1415,320,O,324,HG1,320-O-324-HG1

1416,320,OD1,499,HZ1,320-OD1-499-HZ1

1417,320,OD2,222,HD22,320-OD2-222-HD22

1418,320,OD1,187,HH,320-OD1-187-HH

1419,320,OD1,497,HG1,320-OD1-497-HG1

1420,320,O,324,H,320-O-324-H

1421,320,OD2,499,HZ3,320-OD2-499-HZ3

1422,320,OD2,499,HZ2,320-OD2-499-HZ2

1423,320,OD2,324,HG1,320-OD2-324-HG1

1424,320,OD1,222,HD22,320-OD1-222-HD22

1425,320,OD2,499,HZ1,320-OD2-499-HZ1

1426,320,OD1,499,HZ2,320-OD1-499-HZ2

1427,320,OD1,324,HG1,320-OD1-324-HG1

1428,320,OD2,497,HG1,320-OD2-497-HG1

1429,321,O,325,H,321-O-325-H

1430,321,O,324,H,321-O-324-H

1431,321,OG1,322,H,321-OG1-322-H

1432,322,O,326,H,322-O-326-H

1433,323,O,327,H,323-O-327-H

1434,323,O,327,HG,323-O-327-HG

1435,324,OG1,499,HZ2,324-OG1-499-HZ2

1436,324,OG1,499,HZ1,324-OG1-499-HZ1

1437,324,O,327,HG,324-O-327-HG

1438,324,OG1,497,HG1,324-OG1-497-HG1

1439,324,O,328,H,324-O-328-H

1440,324,OG1,187,HH,324-OG1-187-HH

1441,324,OG1,499,HZ3,324-OG1-499-HZ3

1442,324,O,327,H,324-O-327-H

1443,325,O,329,H,325-O-329-H

1444,325,O,329,HG,325-O-329-HG

1445,326,O,330,H,326-O-330-H

1446,326,O,329,HG,326-O-329-HG

1447,326,O,329,H,326-O-329-H

1448,327,O,331,H,327-O-331-H

1449,327,O,327,HG,327-O-327-HG

1450,327,O,330,H,327-O-330-H

1451,328,O,332,H,328-O-332-H

1452,328,O,331,H,328-O-331-H

1453,329,O,333,H,329-O-333-H

1454,330,O,333,H,330-O-333-H

1455,330,O,334,H,330-O-334-H

1456,331,O,335,H,331-O-335-H

1457,331,O,334,H,331-O-334-H

1458,332,O,336,H,332-O-336-H

1459,332,O,336,HD22,332-O-336-HD22

1460,333,O,336,H,333-O-336-H

1461,333,O,340,HE21,333-O-340-HE21

1462,335,O,336,HD22,335-O-336-HD22

1463,335,O,487,HZ1,335-O-487-HZ1

1464,336,O,339,H,336-O-339-H

1465,336,O,340,H,336-O-340-H

1466,337,O,341,HH11,337-O-341-HH11

1467,337,O,341,HE,337-O-341-HE

1468,337,O,341,H,337-O-341-H

1469,337,O,340,H,337-O-340-H

1470,338,O,341,H,338-O-341-H

1471,338,O,342,H,338-O-342-H

1472,338,O,338,HH11,338-O-338-HH11

1473,339,O,342,H,339-O-342-H

1474,339,O,343,H,339-O-343-H

1475,340,OE1,344,HE22,340-OE1-344-HE22

1476,340,OE1,344,HE21,340-OE1-344-HE21

1477,340,OE1,480,H,340-OE1-480-H

1478,340,O,344,H,340-O-344-H

1479,341,O,341,HH11,341-O-341-HH11

1480,341,O,341,HE,341-O-341-HE

1481,341,O,345,H,341-O-345-H

1482,342,O,345,H,342-O-345-H

1483,342,O,346,H,342-O-346-H

1484,342,O,342,HZ1,342-O-342-HZ1

1485,343,O,347,H,343-O-347-H

1486,343,O,346,H,343-O-346-H

1487,344,OE1,341,HH21,344-OE1-341-HH21

1488,344,O,348,H,344-O-348-H

1489,344,OE1,341,HE,344-OE1-341-HE

1490,344,O,347,H,344-O-347-H

1491,344,OE1,341,HH11,344-OE1-341-HH11

1492,345,OE2,341,HE,345-OE2-341-HE

1493,345,OE1,341,HH21,345-OE1-341-HH21

1494,345,OE2,341,HH21,345-OE2-341-HH21

1495,345,OE1,341,HE,345-OE1-341-HE

1496,345,O,349,HG1,345-O-349-HG1

1497,345,OE1,342,HZ1,345-OE1-342-HZ1

1498,345,OE1,342,HZ3,345-OE1-342-HZ3

1499,345,OE2,342,HZ2,345-OE2-342-HZ2

1500,345,OE2,342,HZ3,345-OE2-342-HZ3

1501,345,OE1,338,HH22,345-OE1-338-HH22

1502,345,OE2,338,HH12,345-OE2-338-HH12

1503,345,OE2,341,HH12,345-OE2-341-HH12

1504,345,OE1,341,HH22,345-OE1-341-HH22

1505,345,OE1,338,HH12,345-OE1-338-HH12

1506,345,OE2,341,HH22,345-OE2-341-HH22

1507,345,OE1,341,HH12,345-OE1-341-HH12

1508,345,OE1,342,HZ2,345-OE1-342-HZ2

1509,345,OE2,338,HH22,345-OE2-338-HH22

1510,345,OE2,342,HZ1,345-OE2-342-HZ1

1511,345,O,349,H,345-O-349-H

1512,345,OE1,341,HH11,345-OE1-341-HH11

1513,346,OE1,342,HZ1,346-OE1-342-HZ1

1514,346,OE2,342,HZ2,346-OE2-342-HZ2

1515,346,OE2,367,H,346-OE2-367-H

1516,346,OE1,349,HG1,346-OE1-349-HG1

1517,346,OE1,342,HZ2,346-OE1-342-HZ2

1518,346,OE1,367,H,346-OE1-367-H

1519,346,OE2,342,HZ1,346-OE2-342-HZ1

1520,346,OE2,342,HZ3,346-OE2-342-HZ3

1521,346,O,349,H,346-O-349-H

1522,346,OE1,342,HZ3,346-OE1-342-HZ3

1523,346,O,350,H,346-O-350-H

1524,347,O,352,H,347-O-352-H

1525,347,O,351,H,347-O-351-H

1526,347,O,350,H,347-O-350-H

1527,348,OD2,353,HH21,348-OD2-353-HH21

1528,348,OD1,511,HH12,348-OD1-511-HH12

1529,348,OD1,511,HH22,348-OD1-511-HH22

1530,348,OD2,511,HH22,348-OD2-511-HH22

1531,348,OD2,511,HH12,348-OD2-511-HH12

1532,348,OD2,353,HE,348-OD2-353-HE

1533,348,OD1,353,HH21,348-OD1-353-HH21

1534,348,OD1,353,HE,348-OD1-353-HE

1535,350,O,355,HH21,350-O-355-HH21

1536,351,O,354,HG,351-O-354-HG

1537,352,O,476,HE21,352-O-476-HE21

1538,353,O,476,HE22,353-O-476-HE22

1539,354,OG,355,H,354-OG-355-H

1540,355,O,476,HE22,355-O-476-HE22

1541,356,O,355,HH11,356-O-355-HH11

1542,357,O,356,HH11,357-O-356-HH11

1543,358,O,361,H,358-O-361-H

1544,359,O,362,H,359-O-362-H

1545,359,O,362,HH11,359-O-362-HH11

1546,360,OG,155,HG1,360-OG-155-HG1

1547,360,OG,358,HH11,360-OG-358-HH11

1548,360,O,363,H,360-O-363-H

1549,360,OG,358,HE,360-OG-358-HE

1550,360,O,363,HG,360-O-363-HG

1551,361,OD1,358,HE,361-OD1-358-HE

1552,361,OD1,358,HH21,361-OD1-358-HH21

1553,361,OD2,355,HH12,361-OD2-355-HH12

1554,361,OD2,358,HE,361-OD2-358-HE

1555,361,O,364,H,361-O-364-H

1556,361,OD1,355,HH12,361-OD1-355-HH12

1557,361,OD1,355,HH22,361-OD1-355-HH22

1558,361,OD2,355,HH22,361-OD2-355-HH22

1559,361,OD1,364,HE2,361-OD1-364-HE2

1560,361,O,365,H,361-O-365-H

1561,361,OD2,358,H,361-OD2-358-H

1562,361,OD2,358,HH21,361-OD2-358-HH21

1563,361,OD1,360,HG,361-OD1-360-HG

1564,362,O,365,H,362-O-365-H

1565,362,NH1,465,HE1,362-NH1-465-HE1

1566,362,NH2,464,HH21,362-NH2-464-HH21

1567,364,ND1,363,HG,364-ND1-363-HG

1568,365,O,369,H,365-O-369-H

1569,365,O,368,H,365-O-368-H

1570,366,O,369,H,366-O-369-H

1571,366,O,370,H,366-O-370-H

1572,367,OH,342,HZ1,367-OH-342-HZ1

1573,367,O,371,H,367-O-371-H

1574,368,O,372,H,368-O-372-H

1575,369,OE1,362,HH22,369-OE1-362-HH22

1576,369,OE2,362,HH12,369-OE2-362-HH12

1577,369,OE1,362,HE,369-OE1-362-HE

1578,369,OE1,464,HH21,369-OE1-464-HH21

1579,369,OE2,362,HH22,369-OE2-362-HH22

1580,369,O,373,H,369-O-373-H

1581,369,O,372,H,369-O-372-H

1582,369,OE2,362,HH21,369-OE2-362-HH21

1583,369,OE1,362,HH21,369-OE1-362-HH21

1584,369,OE1,464,HE,369-OE1-464-HE

1585,369,OE1,362,HH12,369-OE1-362-HH12

1586,369,OE2,465,HE1,369-OE2-465-HE1

1587,369,OE2,464,HH21,369-OE2-464-HH21

1588,369,OE2,362,HE,369-OE2-362-HE

1589,369,OE1,465,HE1,369-OE1-465-HE1

1590,369,OE2,464,HE,369-OE2-464-HE

1591,370,O,373,H,370-O-373-H

1592,370,O,374,H,370-O-374-H

1593,371,O,375,HG1,371-O-375-HG1

1594,371,O,375,H,371-O-375-H

1595,372,O,376,H,372-O-376-H

1596,373,O,377,H,373-O-377-H

1597,374,OE2,431,HH12,374-OE2-431-HH12

1598,374,OE1,431,HH22,374-OE1-431-HH22

1599,374,OE2,431,HH11,374-OE2-431-HH11

1600,374,OE2,377,HE,374-OE2-377-HE

1601,374,OE2,431,HH22,374-OE2-431-HH22

1602,374,OE1,428,H,374-OE1-428-H

1603,374,OE2,377,HH21,374-OE2-377-HH21

1604,374,OE1,431,HH11,374-OE1-431-HH11

1605,374,OE1,377,HH21,374-OE1-377-HH21

1606,374,OE2,428,H,374-OE2-428-H

1607,374,OE1,377,HE,374-OE1-377-HE

1608,374,OE1,431,HH12,374-OE1-431-HH12

1609,374,O,378,H,374-O-378-H

1610,375,O,379,HG,375-O-379-HG

1611,375,O,379,H,375-O-379-H

1612,375,OG1,329,HG,375-OG1-329-HG

1613,376,O,380,H,376-O-380-H

1614,377,O,416,HE2,377-O-416-HE2

1615,377,O,380,HG,377-O-380-HG

1616,378,ND1,377,HH22,378-ND1-377-HH22

1617,378,O,416,HE2,378-O-416-HE2

1618,378,ND1,332,HH,378-ND1-332-HH

1619,379,OG,381,H,379-OG-381-H

1620,380,OG,415,HD22,380-OG-415-HD22

1621,380,O,380,HG,380-O-380-HG

1622,380,O,382,H,380-O-382-H

1623,381,O,497,HG1,381-O-497-HG1

1624,381,O,498,H,381-O-498-H

1625,382,O,411,HE21,382-O-411-HE21

1626,383,O,412,H,383-O-412-H

1627,383,O,411,H,383-O-411-H

1628,385,OG1,409,H,385-OG1-409-H

1629,385,O,230,HG,385-O-230-HG

1630,386,O,106,HH12,386-O-106-HH12

1631,386,O,106,HH11,386-O-106-HH11

1632,386,O,385,HG1,386-O-385-HG1

1633,387,O,106,HH11,387-O-106-HH11

1634,388,NE2,385,HG1,388-NE2-385-HG1

1635,388,O,389,HG,388-O-389-HG

1636,388,O,407,H,388-O-407-H

1637,389,O,105,H,389-O-105-H

1638,390,OG1,404,H,390-OG1-404-H

1639,390,OG1,405,H,390-OG1-405-H

1640,390,O,404,H,390-O-404-H

1641,391,OG1,104,HZ1,391-OG1-104-HZ1

1642,391,OG1,104,HZ2,391-OG1-104-HZ2

1643,391,O,403,HZ3,391-O-403-HZ3

1644,391,OG1,105,H,391-OG1-105-H

1645,391,O,403,HZ2,391-O-403-HZ2

1646,391,OG1,104,HZ3,391-OG1-104-HZ3

1647,391,O,403,HZ1,391-O-403-HZ1

1648,393,OD1,403,HZ3,393-OD1-403-HZ3

1649,393,OD1,403,HZ1,393-OD1-403-HZ1

1650,393,OD1,400,HH,393-OD1-400-HH

1651,393,OD2,403,H,393-OD2-403-H

1652,393,OD1,393,H,393-OD1-393-H

1653,393,OD1,392,HH21,393-OD1-392-HH21

1654,393,OD2,403,HZ3,393-OD2-403-HZ3

1655,393,OD1,403,HZ2,393-OD1-403-HZ2

1656,393,OD2,400,HH,393-OD2-400-HH

1657,393,OD2,403,HZ1,393-OD2-403-HZ1

1658,393,OD2,393,H,393-OD2-393-H

1659,393,OD1,403,H,393-OD1-403-H

1660,393,OD2,403,HZ2,393-OD2-403-HZ2

1661,394,OG1,395,H,394-OG1-395-H

1662,394,OG1,99,HE22,394-OG1-99-HE22

1663,394,OG1,99,HE21,394-OG1-99-HE21

1664,394,O,395,HG,394-O-395-HG

1665,394,O,401,H,394-O-401-H

1666,395,O,99,HE22,395-O-99-HE22

1667,396,O,399,H,396-O-399-H

1668,397,O,399,H,397-O-399-H

1669,397,O,38,HZ3,397-O-38-HZ3

1670,397,O,38,HZ2,397-O-38-HZ2

1671,397,O,38,HZ1,397-O-38-HZ1

1672,398,O,38,HZ3,398-O-38-HZ3

1673,398,O,38,HZ2,398-O-38-HZ2

1674,398,O,38,H,398-O-38-H

1675,398,O,38,HZ1,398-O-38-HZ1

1676,399,O,396,H,399-O-396-H

1677,399,O,395,HG,399-O-395-HG

1678,400,O,39,HD22,400-O-39-HD22

1679,401,O,394,H,401-O-394-H

1680,402,O,405,H,402-O-405-H

1681,402,O,390,HG1,402-O-390-HG1

1682,403,O,405,H,403-O-405-H

1683,403,O,405,HE,403-O-405-HE

1684,403,O,405,HH11,403-O-405-HH11

1685,404,O,405,HH11,404-O-405-HH11

1686,404,O,389,HG,404-O-389-HG

1687,405,O,390,H,405-O-390-H

1688,405,O,389,HG,405-O-389-HG

1689,406,O,84,H,406-O-84-H

1690,407,O,388,H,407-O-388-H

1691,408,O,86,H,408-O-86-H

1692,409,O,385,H,409-O-385-H

1693,409,O,411,H,409-O-411-H

1694,410,O,413,H,410-O-413-H

1695,410,OD1,413,H,410-OD1-413-H

1696,410,OD1,412,H,410-OD1-412-H

1697,410,OD1,411,H,410-OD1-411-H

1698,410,O,87,HG,410-O-87-HG

1699,411,O,415,H,411-O-415-H

1700,411,O,414,H,411-O-414-H

1701,411,O,415,HD22,411-O-415-HD22

1702,411,OE1,451,H,411-OE1-451-H

1703,412,O,415,H,412-O-415-H

1704,412,O,416,H,412-O-416-H

1705,413,NE2,68,HE22,413-NE2-68-HE22

1706,413,NE2,87,HG,413-NE2-87-HG

1707,413,OE1,67,HG,413-OE1-67-HG

1708,413,OE1,68,HE21,413-OE1-68-HE21

1709,413,O,416,H,413-O-416-H

1710,413,NE2,68,HE21,413-NE2-68-HE21

1711,413,OE1,68,HE22,413-OE1-68-HE22

1712,413,OE1,87,HG,413-OE1-87-HG

1713,413,O,417,H,413-O-417-H

1714,413,O,413,HE21,413-O-413-HE21

1715,413,OE1,410,HD22,413-OE1-410-HD22

1716,414,O,417,H,414-O-417-H

1717,414,O,421,HE1,414-O-421-HE1

1718,415,O,377,HH11,415-O-377-HH11

1719,415,OD1,448,H,415-OD1-448-H

1720,416,O,418,HE21,416-O-418-HE21

1721,416,ND1,377,HH12,416-ND1-377-HH12

1722,417,O,420,H,417-O-420-H

1723,417,OD2,413,HE21,417-OD2-413-HE21

1724,417,OD1,413,HE21,417-OD1-413-HE21

1725,417,OD2,419,HZ2,417-OD2-419-HZ2

1726,417,OD1,68,HE21,417-OD1-68-HE21

1727,417,OD1,419,H,417-OD1-419-H

1728,417,OD2,68,HE22,417-OD2-68-HE22

1729,417,OD1,419,HZ3,417-OD1-419-HZ3

1730,417,OD1,419,HZ2,417-OD1-419-HZ2

1731,417,OD2,419,H,417-OD2-419-H

1732,417,OD2,418,HE21,417-OD2-418-HE21

1733,417,OD2,419,HZ3,417-OD2-419-HZ3

1734,417,OD1,420,H,417-OD1-420-H

1735,417,OD1,419,HZ1,417-OD1-419-HZ1

1736,417,OD2,418,H,417-OD2-418-H

1737,417,O,421,H,417-O-421-H

1738,417,OD2,420,H,417-OD2-420-H

1739,417,OD1,413,HE22,417-OD1-413-HE22

1740,417,OD1,68,HE22,417-OD1-68-HE22

1741,417,OD2,419,HZ1,417-OD2-419-HZ1

1742,417,OD2,413,HE22,417-OD2-413-HE22

1743,418,OE1,419,HZ3,418-OE1-419-HZ3

1744,418,OE1,419,HZ2,418-OE1-419-HZ2

1745,418,OE1,418,H,418-OE1-418-H

1746,418,O,418,HE21,418-O-418-HE21

1747,418,OE1,419,HZ1,418-OE1-419-HZ1

1748,418,OE1,68,HE22,418-OE1-68-HE22

1749,418,O,421,H,418-O-421-H

1750,420,O,446,HZ2,420-O-446-HZ2

1751,420,O,446,HZ1,420-O-446-HZ1

1752,420,O,446,HZ3,420-O-446-HZ3

1753,422,O,431,HE,422-O-431-HE

1754,422,O,431,HH21,422-O-431-HH21

1755,422,O,431,HH22,422-O-431-HH22

1756,423,O,431,HH22,423-O-431-HH22

1757,423,OD1,425,HG,423-OD1-425-HG

1758,423,OD1,418,HE22,423-OD1-418-HE22

1759,423,O,431,HH21,423-O-431-HH21

1760,423,O,423,HD22,423-O-423-HD22

1761,423,O,426,H,423-O-426-H

1762,423,OD1,431,HH21,423-OD1-431-HH21

1763,424,O,377,HH22,424-O-377-HH22

1764,424,O,426,H,424-O-426-H

1765,424,O,377,HH12,424-O-377-HH12

1766,425,O,378,HE2,425-O-378-HE2

1767,425,O,425,HG,425-O-425-HG

1768,426,O,431,HH22,426-O-431-HH22

1769,426,OE2,425,HG,426-OE2-425-HG

1770,426,OE1,427,H,426-OE1-427-H

1771,426,OE1,425,HG,426-OE1-425-HG

1772,426,OE1,423,HD22,426-OE1-423-HD22

1773,426,OE2,423,HD22,426-OE2-423-HD22

1774,426,OE2,427,H,426-OE2-427-H

1775,426,OE1,423,HD21,426-OE1-423-HD21

1776,426,OE2,423,HD21,426-OE2-423-HD21

1777,426,O,431,HH12,426-O-431-HH12

1778,427,O,338,HH21,427-O-338-HH21

1779,428,O,431,H,428-O-431-H

1780,428,O,431,HH11,428-O-431-HH11

1781,429,O,432,H,429-O-432-H

1782,429,O,433,H,429-O-433-H

1783,430,OE2,338,HH22,430-OE2-338-HH22

1784,430,OE2,430,H,430-OE2-430-H

1785,430,OE2,338,HH12,430-OE2-338-HH12

1786,430,OE1,338,HH12,430-OE1-338-HH12

1787,430,OE1,338,HH21,430-OE1-338-HH21

1788,430,OE2,342,HZ2,430-OE2-342-HZ2

1789,430,OE1,338,HH22,430-OE1-338-HH22

1790,430,OE2,342,HZ3,430-OE2-342-HZ3

1791,430,OE1,367,HH,430-OE1-367-HH

1792,430,OE1,342,HZ3,430-OE1-342-HZ3

1793,430,OE1,430,H,430-OE1-430-H

1794,430,O,433,H,430-O-433-H

1795,430,OE2,367,HH,430-OE2-367-HH

1796,430,OE2,342,HZ1,430-OE2-342-HZ1

1797,430,OE1,342,HZ1,430-OE1-342-HZ1

1798,430,OE1,342,HZ2,430-OE1-342-HZ2

1799,431,O,433,H,431-O-433-H

1800,432,O,440,H,432-O-440-H

1801,434,OG1,438,H,434-OG1-438-H

1802,434,OG1,437,H,434-OG1-437-H

1803,434,OG1,436,H,434-OG1-436-H

1804,434,O,437,H,434-O-437-H

1805,435,O,437,H,435-O-437-H

1806,436,OD2,438,H,436-OD2-438-H

1807,436,OD2,436,H,436-OD2-436-H

1808,436,OD1,434,HG1,436-OD1-434-HG1

1809,436,OD1,436,H,436-OD1-436-H

1810,436,O,438,H,436-O-438-H

1811,436,OD1,438,H,436-OD1-438-H

1812,436,OD2,434,HG1,436-OD2-434-HG1

1813,438,O,434,HG1,438-O-434-HG1

1814,438,O,434,H,438-O-434-H

1815,439,O,441,HZ1,439-O-441-HZ1

1816,439,O,441,H,439-O-441-H

1817,439,O,441,HZ3,439-O-441-HZ3

1818,439,O,441,HZ2,439-O-441-HZ2

1819,440,OD1,442,H,440-OD1-442-H

1820,440,O,443,H,440-O-443-H

1821,440,OD2,442,H,440-OD2-442-H

1822,440,O,442,H,440-O-442-H

1823,440,O,444,H,440-O-444-H

1824,440,O,444,HG,440-O-444-HG

1825,440,OD2,443,H,440-OD2-443-H

1826,440,OD1,443,H,440-OD1-443-H

1827,441,O,445,H,441-O-445-H

1828,441,O,441,HZ3,441-O-441-HZ3

1829,441,O,444,HG,441-O-444-HG

1830,442,O,446,HZ1,442-O-446-HZ1

1831,442,O,446,HZ3,442-O-446-HZ3

1832,442,O,446,HZ2,442-O-446-HZ2

1833,442,O,446,H,442-O-446-H

1834,442,O,445,H,442-O-445-H

1835,443,O,446,H,443-O-446-H

1836,443,O,447,H,443-O-447-H

1837,444,OG,464,HH22,444-OG-464-HH22

1838,444,O,464,HH22,444-O-464-HH22

1839,444,OG,464,HH12,444-OG-464-HH12

1840,444,O,447,H,444-O-447-H

1841,444,O,444,HG,444-O-444-HG

1842,445,OE2,444,HG,445-OE2-444-HG

1843,445,OE2,93,HH21,445-OE2-93-HH21

1844,445,OE1,446,HZ1,445-OE1-446-HZ1

1845,445,OE1,464,HH22,445-OE1-464-HH22

1846,445,OE1,444,HG,445-OE1-444-HG

1847,445,OE1,93,HH21,445-OE1-93-HH21

1848,445,OE1,441,HZ2,445-OE1-441-HZ2

1849,445,OE2,464,HH22,445-OE2-464-HH22

1850,445,OE1,441,HZ3,445-OE1-441-HZ3

1851,445,OE2,446,HZ2,445-OE2-446-HZ2

1852,445,OE2,464,HH12,445-OE2-464-HH12

1853,445,OE1,456,HZ2,445-OE1-456-HZ2

1854,445,OE1,456,HZ3,445-OE1-456-HZ3

1855,445,OE2,446,HZ3,445-OE2-446-HZ3

1856,445,OE2,456,HZ2,445-OE2-456-HZ2

1857,445,OE1,456,HZ1,445-OE1-456-HZ1

1858,445,OE2,456,HZ3,445-OE2-456-HZ3

1859,445,OE1,93,HH12,445-OE1-93-HH12

1860,445,OE1,93,HH22,445-OE1-93-HH22

1861,445,OE1,464,HH12,445-OE1-464-HH12

1862,445,OE2,441,HZ1,445-OE2-441-HZ1

1863,445,OE2,441,HZ3,445-OE2-441-HZ3

1864,445,OE2,93,HH12,445-OE2-93-HH12

1865,445,OE2,93,HH22,445-OE2-93-HH22

1866,445,OE2,446,HZ1,445-OE2-446-HZ1

1867,445,OE2,441,HZ2,445-OE2-441-HZ2

1868,445,OE2,456,HZ1,445-OE2-456-HZ1

1869,445,O,93,HH22,445-O-93-HH22

1870,445,O,93,HH21,445-O-93-HH21

1871,446,O,448,H,446-O-448-H

1872,447,O,449,H,447-O-449-H

1873,448,O,415,HD21,448-O-415-HD21

1874,449,O,411,HE22,449-O-411-HE22

1875,450,O,457,H,450-O-457-H

1876,451,O,456,HZ1,451-O-456-HZ1

1877,451,O,456,HZ3,451-O-456-HZ3

1878,451,O,456,HZ2,451-O-456-HZ2

1879,452,O,455,H,452-O-455-H

1880,452,O,456,H,452-O-456-H

1881,453,O,456,H,453-O-456-H

1882,453,O,455,H,453-O-455-H

1883,454,O,135,HH21,454-O-135-HH21

1884,454,O,456,H,454-O-456-H

1885,454,O,135,HH12,454-O-135-HH12

1886,455,O,452,H,455-O-452-H

1887,455,O,135,HH12,455-O-135-HH12

1888,456,O,135,HH22,456-O-135-HH22

1889,456,O,135,HH12,456-O-135-HH12

1890,457,O,135,HH22,457-O-135-HH22

1891,457,O,456,HZ2,457-O-456-HZ2

1892,457,O,460,H,457-O-460-H

1893,458,O,460,H,458-O-460-H

1894,458,O,139,HE21,458-O-139-HE21

1895,458,O,139,HE22,458-O-139-HE22

1896,459,O,463,H,459-O-463-H

1897,459,O,462,H,459-O-462-H

1898,459,O,461,H,459-O-461-H

1899,460,OE2,464,HH21,460-OE2-464-HH21

1900,460,OE1,461,HG1,460-OE1-461-HG1

1901,460,OE2,461,HG1,460-OE2-461-HG1

1902,460,OE2,456,HZ3,460-OE2-456-HZ3

1903,460,OE2,456,HZ2,460-OE2-456-HZ2

1904,460,OE1,461,H,460-OE1-461-H

1905,460,O,464,HE,460-O-464-HE

1906,460,OE2,456,HZ1,460-OE2-456-HZ1

1907,460,OE1,464,HH21,460-OE1-464-HH21

1908,460,OE2,464,HH12,460-OE2-464-HH12

1909,460,O,464,H,460-O-464-H

1910,460,O,464,HH11,460-O-464-HH11

1911,460,OE1,464,HH22,460-OE1-464-HH22

1912,460,OE1,456,HZ3,460-OE1-456-HZ3

1913,460,OE2,461,H,460-OE2-461-H

1914,460,OE2,464,HH22,460-OE2-464-HH22

1915,460,O,464,HH21,460-O-464-HH21

1916,460,OE1,143,HZ3,460-OE1-143-HZ3

1917,460,O,464,HH12,460-O-464-HH12

1918,460,OE1,456,HZ2,460-OE1-456-HZ2

1919,460,OE1,464,HH12,460-OE1-464-HH12

1920,460,OE1,456,HZ1,460-OE1-456-HZ1

1921,460,OE2,143,HZ2,460-OE2-143-HZ2

1922,460,OE1,464,HH11,460-OE1-464-HH11

1923,461,O,464,H,461-O-464-H

1924,461,O,464,HE,461-O-464-HE

1925,461,O,465,H,461-O-465-H

1926,461,OG1,143,HZ1,461-OG1-143-HZ1

1927,461,OG1,464,HH21,461-OG1-464-HH21

1928,461,OG1,143,HZ3,461-OG1-143-HZ3

1929,462,O,464,H,462-O-464-H

1930,462,O,465,H,462-O-465-H

1931,462,O,466,H,462-O-466-H

1932,463,O,467,H,463-O-467-H

1933,464,O,468,H,464-O-468-H

1934,464,NH2,465,HE1,464-NH2-465-HE1

1935,465,O,468,H,465-O-468-H

1936,465,O,469,H,465-O-469-H

1937,466,O,469,H,466-O-469-H

1938,466,O,470,H,466-O-470-H

1939,467,O,471,H,467-O-471-H

1940,468,O,472,H,468-O-472-H

1941,469,O,473,H,469-O-473-H

1942,470,O,473,H,470-O-473-H

1943,470,O,474,H,470-O-474-H

1944,471,O,475,H,471-O-475-H

1945,471,O,474,H,471-O-474-H

1946,472,O,476,H,472-O-476-H

1947,473,O,477,H,473-O-477-H

1948,473,O,477,HH11,473-O-477-HH11

1949,473,O,477,HE,473-O-477-HE

1950,473,O,477,HH21,473-O-477-HH21

1951,473,O,476,H,473-O-476-H

1952,474,O,477,H,474-O-477-H

1953,474,O,478,H,474-O-478-H

1954,475,O,344,HE21,475-O-344-HE21

1955,475,O,511,HE,475-O-511-HE

1956,475,O,511,HH21,475-O-511-HH21

1957,476,O,511,HE,476-O-511-HE

1958,476,OE1,477,HH22,476-OE1-477-HH22

1959,476,O,353,HH21,476-O-353-HH21

1960,476,O,511,HH21,476-O-511-HH21

1961,476,OE1,477,HH21,476-OE1-477-HH21

1962,476,O,353,HE,476-O-353-HE

1963,476,OE1,477,HE,476-OE1-477-HE

1964,476,OE1,477,HH12,476-OE1-477-HH12

1965,477,O,511,H,477-O-511-H

1966,478,O,344,HE21,478-O-344-HE21

1967,478,O,344,HE22,478-O-344-HE22

1968,479,OE2,512,HG,479-OE2-512-HG

1969,479,OE1,341,HH11,479-OE1-341-HH11

1970,479,OE1,512,HG,479-OE1-512-HG

1971,479,OE1,512,H,479-OE1-512-H

1972,479,OE1,341,HE,479-OE1-341-HE

1973,479,OE2,509,HE21,479-OE2-509-HE21

1974,479,OE1,509,HE21,479-OE1-509-HE21

1975,479,OE2,341,HH11,479-OE2-341-HH11

1976,479,O,509,H,479-O-509-H

1977,479,OE2,512,H,479-OE2-512-H

1978,479,OE1,341,HH21,479-OE1-341-HH21

1979,479,OE2,341,HH21,479-OE2-341-HH21

1980,479,OE2,344,HE22,479-OE2-344-HE22

1981,479,OE2,341,HH12,479-OE2-341-HH12

1982,479,OE1,341,HH22,479-OE1-341-HH22

1983,479,OE2,341,HH22,479-OE2-341-HH22

1984,479,OE1,341,HH12,479-OE1-341-HH12

1985,480,O,340,HE22,480-O-340-HE22

1986,481,O,507,H,481-O-507-H

1987,481,O,507,HE21,481-O-507-HE21

1988,481,OG,509,HE22,481-OG-509-HE22

1989,481,OG,507,HE21,481-OG-507-HE21

1990,481,OG,482,H,481-OG-482-H

1991,482,O,487,HZ3,482-O-487-HZ3

1992,482,O,481,HG,482-O-481-HG

1993,482,O,507,HE21,482-O-507-HE21

1994,483,O,486,H,483-O-486-H

1995,484,O,487,HZ3,484-O-487-HZ3

1996,484,O,487,HZ2,484-O-487-HZ2
1997,484,O,487,HZ1,484-O-487-HZ1
1998,484,O,486,H,484-O-486-H
1999,485,O,487,HZ3,485-O-487-HZ3
2000,485,O,487,H,485-O-487-H
2001,485,O,487,HZ1,485-O-487-HZ1
2002,485,O,487,HZ2,485-O-487-HZ2
2003,486,O,488,H,486-O-488-H
2004,486,O,505,HE2,486-O-505-HE2
2005,487,O,505,HE2,487-O-505-HE2
2006,487,O,489,H,487-O-489-H
2007,488,O,487,HZ1,488-O-487-HZ1
2008,488,O,490,H,488-O-490-H
2009,488,O,487,HZ3,488-O-487-HZ3
2010,488,O,487,HZ2,488-O-487-HZ2
2011,489,OD1,491,HG1,489-OD1-491-HG1
2012,489,O,491,H,489-O-491-H
2013,489,OD1,491,H,489-OD1-491-H
2014,489,OD2,491,HG1,489-OD2-491-HG1
2015,489,OD2,491,H,489-OD2-491-H
2016,489,OD1,490,H,489-OD1-490-H
2017,489,OD1,487,HZ1,489-OD1-487-HZ1
2018,489,O,500,HD1,489-O-500-HD1
2019,489,OD2,490,H,489-OD2-490-H
2020,489,OD1,487,HZ2,489-OD1-487-HZ2
2021,489,O,501,H,489-O-501-H
2022,490,O,501,H,490-O-501-H
2023,491,O,501,H,491-O-501-H
2024,492,O,58,HD21,492-O-58-HD21
2025,493,O,499,H,493-O-499-H
2026,494,OH,230,HG,494-OH-230-HG
2027,494,OH,230,H,494-OH-230-H
2028,494,OH,231,H,494-OH-231-H
2029,496,O,497,HG1,496-O-497-HG1
2030,497,OG1,499,HZ3,497-OG1-499-HZ3
2031,497,O,499,HZ2,497-O-499-HZ2
2032,497,O,499,HZ1,497-O-499-HZ1
2033,497,O,499,HZ3,497-O-499-HZ3
2034,497,OG1,499,HZ1,497-OG1-499-HZ1
2035,497,OG1,499,HZ2,497-OG1-499-HZ2
2036,498,O,328,HE1,498-O-328-HE1
2037,499,O,493,H,499-O-493-H
2038,500,NE2,327,HG,500-NE2-327-HG
2039,500,O,500,HD1,500-O-500-HD1
2040,500,O,502,H,500-O-502-H
2041,501,O,500,HD1,501-O-500-HD1
2042,501,O,185,HD21,501-O-185-HD21
2043,501,O,503,H,501-O-503-H
2044,502,O,185,HD22,502-O-185-HD22
2045,502,O,505,HE2,502-O-505-HE2
2046,502,O,504,H,502-O-504-H
2047,503,O,185,HD22,503-O-185-HD22
2048,503,O,505,H,503-O-505-H
2049,504,OE2,188,HE,504-OE2-188-HE
2050,504,OE2,188,HH22,504-OE2-188-HH22
2051,504,OE1,185,HD22,504-OE1-185-HD22
2052,504,OE1,185,H,504-OE1-185-H
2053,504,OE1,188,HH21,504-OE1-188-HH21
2054,504,O,506,H,504-O-506-H
2055,504,OE2,185,H,504-OE2-185-H
2056,504,OE1,504,H,504-OE1-504-H
2057,504,OE1,188,HH12,504-OE1-188-HH12
2058,504,OE1,188,HH11,504-OE1-188-HH11
2059,504,OE2,188,HH11,504-OE2-188-HH11
2060,504,OE1,188,HE,504-OE1-188-HE
2061,504,OE2,185,HD22,504-OE2-185-HD22
2062,504,OE2,188,HH12,504-OE2-188-HH12
2063,504,OE1,505,HE2,504-OE1-505-HE2
2064,504,OE1,188,HH22,504-OE1-188-HH22
2065,504,OE2,504,H,504-OE2-504-H
2066,504,OE2,188,HH21,504-OE2-188-HH21
2067,505,ND1,504,H,505-ND1-504-H
2068,505,ND1,505,H,505-ND1-505-H
2069,505,ND1,489,H,505-ND1-489-H
2070,506,O,184,H,506-O-184-H
2071,507,OE1,509,HE21,507-OE1-509-HE21
2072,507,OE1,509,HE22,507-OE1-509-HE22
2073,507,O,481,H,507-O-481-H
2074,507,OE1,481,HG,507-OE1-481-HG
2075,507,OE1,183,HE2,507-OE1-183-HE2

2076,507,O,509,HE22,507-O-509-HE22

2077,508,O,509,HE21,508-O-509-HE21

2078,509,OE1,481,HG,509-OE1-481-HG

2079,509,OE1,510,H,509-OE1-510-H

2080,509,O,479,H,509-O-479-H

2081,509,OE1,507,HE21,509-OE1-507-HE21

2082,509,OE1,175,HE22,509-OE1-175-HE22

2083,510,O,509,HE21,510-O-509-HE21

2084,511,O,353,HH12,511-O-353-HH12

2085,512,O,512,HG,512-O-512-HG

2086,512,O,353,HH22,512-O-353-HH22

2087,512,OXT,353,HH12,512-OXT-353-HH12

2088,512,OXT,353,HH22,512-OXT-353-HH22

2089,512,OXT,353,HH11,512-OXT-353-HH11

2090,512,OXT,512,HG,512-OXT-512-HG

2091,512,O,353,HH12,512-O-353-HH12

2092,513,O,516,H17,513-O-516-H17

2093,515,O1A,106,HH22,515-O1A-106-HH22

2094,515,O2D,106,HH21,515-O2D-106-HH21

2095,515,O2A,452,H,515-O2A-452-H

2096,515,O1D,455,HH11,515-O1D-455-HH11

2097,515,O1D,131,HE1,515-O1D-131-HE1

2098,515,O2D,455,HH11,515-O2D-455-HH11

2099,515,O2D,131,HE1,515-O2D-131-HE1

2100,515,O2A,106,HH12,515-O2A-106-HH12

2101,515,O2D,135,HH11,515-O2D-135-HH11

2102,515,O1D,106,HH21,515-O1D-106-HH21

2103,515,O1D,135,HH11,515-O1D-135-HH11

2104,515,O2A,106,HH22,515-O2A-106-HH22

2105,515,O1A,386,H,515-O1A-386-H

2106,515,O1A,106,HH12,515-O1A-106-HH12

2107,516,O15,497,HG1,516-O15-497-HG1

Model II

RES_DONOR,ATOM_DONOR,RES_ACCEPTOR,ATOM_ACCEPTOR,
TAG

1,37,O,39,HD22,37-O-39-HD22

2,37,O,39,H,37-O-39-H

3,38,O,400,H,38-O-400-H

4,39,O,38,HZ3,39-O-38-HZ3

5,39,OD1,38,HZ2,39-OD1-38-HZ2

6,39,OD1,39,H,39-OD1-39-H

7,39,O,39,HD22,39-O-39-HD22

8,39,OD1,405,HH21,39-OD1-405-HH21

9,39,OD1,75,HE22,39-OD1-75-HE22

10,40,O,75,HE21,40-O-75-HE21

11,40,O,39,HD22,40-O-39-HD22

12,41,O,75,H,41-O-75-H

13,43,O,51,HE2,43-O-51-HE2

14,44,O,51,HE2,44-O-51-HE2

15,45,O,77,HH22,45-O-77-HH22

16,45,O,77,HE,45-O-77-HE

17,45,O,77,HH12,45-O-77-HH12

18,45,O,77,HH11,45-O-77-HH11

19,45,O,77,HH21,45-O-77-HH21

20,46,O,49,H,46-O-49-H

21,46,O,77,HH22,46-O-77-HH22

22,46,O,48,H,46-O-48-H

23,46,O,50,H,46-O-50-H

24,46,O,77,HH12,46-O-77-HH12

25,47,O,77,HH11,47-O-77-HH11

26,47,O,77,HE,47-O-77-HE

27,47,O,77,HH12,47-O-77-HH12

28,47,O,77,HH22,47-O-77-HH22

29,47,O,77,HH21,47-O-77-HH21

30,47,O,80,HG,47-O-80-HG

31,49,O,52,H,49-O-52-H

32,49,O,79,H,49-O-79-H

33,50,O,52,H,50-O-52-H

34,50,O,46,H,50-O-46-H

35,51,ND1,51,H,51-ND1-51-H

36,51,O,55,H,51-O-55-H

37,51,O,54,H,51-O-54-H

38,51,ND1,77,H,51-ND1-77-H

39,51,O,54,HG1,51-O-54-HG1

40,52,O,56,H,52-O-56-H

41,52,O,55,H,52-O-55-H

42,53,O,56,H,53-O-56-H

43,54,O,65,HH22,54-O-65-HH22

44,54,OG1,46,HE1,54-OG1-46-HE1

45,54,O,56,H,54-O-56-H

46,55,O,57,H,55-O-57-H

47,55,O,58,H,55-O-58-H

48,57,O,57,HZ1,57-O-57-HZ1

49,57,O,57,HZ2,57-O-57-HZ2

50,57,O,58,HD22,57-O-58-HD22

51,58,O,61,H,58-O-61-H

52,58,OD1,57,HZ2,58-OD1-57-HZ2

53,58,O,62,H,58-O-62-H

54,59,O,63,H,59-O-63-H

55,60,ND1,60,H,60-ND1-60-H

56,60,O,410,HD21,60-O-410-HD21

57,60,O,64,HG,60-O-64-HG

58,60,O,64,H,60-O-64-H

59,60,ND1,61,H,60-ND1-61-H

60,61,O,64,H,61-O-64-H

61,61,O,65,H,61-O-65-H

62,61,O,65,HH11,61-O-65-HH11

63,62,O,66,H,62-O-66-H

64,62,O,65,H,62-O-65-H

65,63,O,66,H,63-O-66-H

66,63,O,413,HE22,63-O-413-HE22

67,63,O,67,HG,63-O-67-HG

68,63,O,67,H,63-O-67-H

69,64,OG,410,HD21,64-OG-410-HD21

70,64,O,67,HG,64-O-67-HG

71,64,O,413,HE22,64-O-413-HE22

72,64,O,68,H,64-O-68-H

73,64,O,68,HE21,64-O-68-HE21

74,64,O,67,H,64-O-67-H

75,64,OG,65,HH21,64-OG-65-HH21

76,65,O,69,HE21,65-O-69-HE21

77,65,O,69,H,65-O-69-H

78,65,O,69,HE22,65-O-69-HE22

79,65,O,65,HH11,65-O-65-HH11

80,65,O,68,H,65-O-68-H

81,66,O,69,HE21,66-O-69-HE21

82,66,O,70,H,66-O-70-H

83,66,O,69,H,66-O-69-H

84,67,OG,68,HE21,67-OG-68-HE21

85,67,OG,413,HE22,67-OG-413-HE22

86,67,O,70,H,67-O-70-H

87,67,O,69,H,67-O-69-H

88,67,O,71,H,67-O-71-H

89,67,OG,410,HD22,67-OG-410-HD22

90,67,OG,87,HG,67-OG-87-HG

91,67,OG,413,HE21,67-OG-413-HE21

92,68,OE1,418,HE22,68-OE1-418-HE22

93,68,OE1,413,HE22,68-OE1-413-HE22

94,68,OE1,65,HH11,68-OE1-65-HH11

95,68,OE1,419,HZ3,68-OE1-419-HZ3

96,68,NE2,413,HE22,68-NE2-413-HE22

97,68,OE1,65,HE,68-OE1-65-HE

98,68,OE1,65,HH21,68-OE1-65-HH21

99,68,OE1,419,HZ1,68-OE1-419-HZ1

100,68,OE1,419,HZ2,68-OE1-419-HZ2

101,68,OE1,413,HE21,68-OE1-413-HE21

102,69,OE1,65,HH22,69-OE1-65-HH22

103,69,OE1,70,HH,69-OE1-70-HH

104,69,OE1,65,HH12,69-OE1-65-HH12

105,69,O,69,HE21,69-O-69-HE21

106,69,OE1,65,HH21,69-OE1-65-HH21

107,70,OH,69,HE21,70-OH-69-HE21

108,71,O,67,HG,71-O-67-HG

109,71,O,87,HG,71-O-87-HG

110,72,OD2,72,H,72-OD2-72-H

111,72,OD1,397,HZ1,72-OD1-397-HZ1

112,72,OD2,397,HZ2,72-OD2-397-HZ2

113,72,OD1,397,HZ2,72-OD1-397-HZ2

114,72,O,87,H,72-O-87-H

115,72,OD1,397,HZ3,72-OD1-397-HZ3

116,72,OD1,72,H,72-OD1-72-H

117,72,O,88,H,72-O-88-H

118,72,OD2,397,HZ3,72-OD2-397-HZ3

119,72,OD2,91,HG1,72-OD2-91-HG1

120,72,OD2,397,HZ1,72-OD2-397-HZ1

121,72,OD1,91,HG1,72-OD1-91-HG1

122,74,O,85,H,74-O-85-H

123,75,NE2,39,HD22,75-NE2-39-HD22

124,75,OE1,405,HE,75-OE1-405-HE

125,75,OE1,405,HH21,75-OE1-405-HH21

126,75,OE1,39,HD22,75-OE1-39-HD22

127,75,OE1,405,HH11,75-OE1-405-HH11

128,75,OE1,39,HD21,75-OE1-39-HD21

129,75,OE1,77,HE,75-OE1-77-HE

130,76,O,75,HE21,76-O-75-HE21

131,76,O,83,H,76-O-83-H

132,77,O,51,H,77-O-51-H

133,78,O,81,H,78-O-81-H

134,78,O,81,HG1,78-O-81-HG1

135,79,O,77,HH21,79-O-77-HH21

136,79,O,77,HE,79-O-77-HE

137,79,O,77,HH11,79-O-77-HH11

138,80,O,77,HH12,80-O-77-HH12

139,80,O,77,HH11,80-O-77-HH11

140,80,O,77,HE,80-O-77-HE

141,80,O,80,HG,80-O-80-HG

142,80,O,77,HH21,80-O-77-HH21

143,80,OG,81,HG1,80-OG-81-HG1

144,81,O,81,HG1,81-O-81-HG1

145,81,OG1,405,HH12,81-OG1-405-HH12

146,81,O,78,H,81-O-78-H

147,82,O,405,HE,82-O-405-HE

148,82,O,405,HH11,82-O-405-HH11

149,83,O,75,HE21,83-O-75-HE21

150,83,O,76,H,83-O-76-H

151,84,O,408,H,84-O-408-H

152,85,O,74,H,85-O-74-H

153,85,O,87,HG,85-O-87-HG

154,86,O,87,HG,86-O-87-HG

155,86,O,410,H,86-O-410-H

156,87,OG,410,HD22,87-OG-410-HD22

157,87,O,67,HG,87-O-67-HG

158,87,O,87,HG,87-O-87-HG

159,87,OG,67,HG,87-OG-67-HG

160,87,O,413,HE22,87-O-413-HE22

161,87,O,413,HE21,87-O-413-HE21

162,88,O,91,H,88-O-91-H

163,88,O,92,H,88-O-92-H

164,89,O,92,H,89-O-92-H

165,89,O,93,H,89-O-93-H

166,90,OD2,93,HH22,90-OD2-93-HH22

167,90,O,94,H,90-O-94-H

168,90,OD2,93,HH11,90-OD2-93-HH11

169,90,O,94,HE21,90-O-94-HE21

170,90,OD1,94,HE21,90-OD1-94-HE21

171,90,OD2,98,HH12,90-OD2-98-HH12

172,90,OD1,93,HH11,90-OD1-93-HH11

173,90,OD1,397,HZ2,90-OD1-397-HZ2

174,90,OD1,91,HG1,90-OD1-91-HG1

175,90,OD1,91,H,90-OD1-91-H

176,90,OD2,397,HZ2,90-OD2-397-HZ2

177,90,OD1,397,HZ3,90-OD1-397-HZ3

178,90,OD2,91,HG1,90-OD2-91-HG1

179,90,OD2,91,H,90-OD2-91-H

180,90,OD1,98,HH22,90-OD1-98-HH22

181,90,OD2,94,HE21,90-OD2-94-HE21

182,90,OD2,397,HZ3,90-OD2-397-HZ3

183,90,OD2,98,HH22,90-OD2-98-HH22

184,90,OD1,397,HZ1,90-OD1-397-HZ1

185,90,OD1,93,HH21,90-OD1-93-HH21

186,90,OD2,93,HH21,90-OD2-93-HH21

187,90,OD2,93,HE,90-OD2-93-HE

188,90,O,98,HH22,90-O-98-HH22

189,90,OD2,93,HH12,90-OD2-93-HH12

190,90,OD1,98,HH12,90-OD1-98-HH12

191,90,OD1,93,HE,90-OD1-93-HE

192,90,OD1,90,H,90-OD1-90-H

193,90,OD2,397,HZ1,90-OD2-397-HZ1

194,90,OD1,93,HH12,90-OD1-93-HH12

195,90,O,98,HH12,90-O-98-HH12

196,90,OD1,93,HH22,90-OD1-93-HH22

197,91,OG1,397,HZ1,91-OG1-397-HZ1

198,91,OG1,397,HZ2,91-OG1-397-HZ2

199,91,OG1,397,HZ3,91-OG1-397-HZ3

200,91,O,95,H,91-O-95-H

201,92,O,95,H,92-O-95-H

202,92,O,96,H,92-O-96-H

203,93,O,98,H,93-O-98-H

204,93,O,97,H,93-O-97-H

205,94,OE1,98,HH11,94-OE1-98-HH11

206,94,OE1,397,HZ1,94-OE1-397-HZ1

207,94,OE1,397,HZ2,94-OE1-397-HZ2

208,94,OE1,397,HZ3,94-OE1-397-HZ3

209,94,OE1,397,H,94-OE1-397-H

210,94,O,98,H,94-O-98-H

211,94,OE1,99,HE21,94-OE1-99-HE21

212,94,OE1,98,HE,94-OE1-98-HE

213,94,OE1,98,HH21,94-OE1-98-HH21

214,94,O,99,HE21,94-O-99-HE21

215,94,O,99,H,94-O-99-H

216,95,O,100,H,95-O-100-H

217,95,O,99,H,95-O-99-H

218,96,O,454,H,96-O-454-H

219,97,O,454,HZ3,97-O-454-HZ3

220,97,O,454,HZ2,97-O-454-HZ2

221,97,O,454,HZ1,97-O-454-HZ1

222,97,O,100,H,97-O-100-H

223,98,O,99,HE21,98-O-99-HE21

224,98,O,98,HE,98-O-98-HE

225,98,O,100,H,98-O-100-H

226,98,O,98,HH11,98-O-98-HH11

227,99,OE1,94,HE21,99-OE1-94-HE21

228,99,OE1,98,HH21,99-OE1-98-HH21

229,99,OE1,98,HH11,99-OE1-98-HH11

230,99,O,103,H,99-O-103-H

231,99,O,102,H,99-O-102-H

232,99,O,101,H,99-O-101-H

233,99,OE1,98,HE,99-OE1-98-HE

234,100,O,104,H,100-O-104-H

235,100,O,103,H,100-O-103-H

236,101,O,392,HH12,101-O-392-HH12

237,101,O,104,HZ1,101-O-104-HZ1

238,101,OD1,454,HZ3,101-OD1-454-HZ3

239,101,OD1,454,HZ1,101-OD1-454-HZ1

240,101,OD1,104,HZ2,101-OD1-104-HZ2

241,101,OD1,104,HZ3,101-OD1-104-HZ3

242,101,OD1,454,HZ2,101-OD1-454-HZ2

243,101,OD2,454,HZ3,101-OD2-454-HZ3

244,101,OD2,454,HZ2,101-OD2-454-HZ2

245,101,O,392,HH11,101-O-392-HH11

246,101,OD2,454,HZ1,101-OD2-454-HZ1

247,101,O,104,HZ3,101-O-104-HZ3

248,101,OD2,392,HH12,101-OD2-392-HH12

249,101,OD2,392,HH22,101-OD2-392-HH22

250,101,OD2,104,HZ1,101-OD2-104-HZ1

251,101,OD2,104,HZ2,101-OD2-104-HZ2

252,101,OD1,392,HH12,101-OD1-392-HH12

253,101,OD1,392,HH22,101-OD1-392-HH22

254,101,OD2,104,HZ3,101-OD2-104-HZ3

255,101,O,104,HZ2,101-O-104-HZ2

256,101,OD2,101,H,101-OD2-101-H

257,101,O,391,HG1,101-O-391-HG1

258,101,OD1,104,HZ1,101-OD1-104-HZ1

259,102,O,391,HG1,102-O-391-HG1

260,102,OD1,392,HH11,102-OD1-392-HH11

261,102,OD2,392,HE,102-OD2-392-HE

262,102,O,392,H,102-O-392-H

263,102,O,391,H,102-O-391-H

264,102,OD1,99,HE21,102-OD1-99-HE21

265,102,OD2,392,HH21,102-OD2-392-HH21

266,102,OD2,394,HG1,102-OD2-394-HG1

267,102,O,104,H,102-O-104-H

268,102,OD1,392,HH21,102-OD1-392-HH21

269,102,OD1,392,HE,102-OD1-392-HE

270,102,OD2,99,HE21,102-OD2-99-HE21

271,102,OD2,392,HH11,102-OD2-392-HH11

272,102,OD1,394,HG1,102-OD1-394-HG1

273,103,O,105,H,103-O-105-H

274,103,O,388,HD1,103-O-388-HD1

275,104,O,455,HE,104-O-455-HE

276,104,O,455,HH21,104-O-455-HH21

277,104,O,127,HG,104-O-127-HG

278,104,O,455,HH22,104-O-455-HH22

279,105,O,389,H,105-O-389-H

280,107,O,109,H,107-O-109-H

281,108,OD1,108,H,108-OD1-108-H

282,108,OD2,108,H,108-OD2-108-H

283,109,O,113,HG1,109-O-113-HG1

284,109,O,113,H,109-O-113-H

285,109,O,112,H,109-O-112-H

286,110,OH,252,HZ1,110-OH-252-HZ1

287,110,OH,252,HZ3,110-OH-252-HZ3

288,110,O,113,H,110-O-113-H

289,110,O,113,HG1,110-O-113-HG1

290,110,O,114,H,110-O-114-H

291,110,OH,252,HZ2,110-OH-252-HZ2

292,111,O,115,H,111-O-115-H

293,111,OG1,112,H,111-OG1-112-H

294,111,OG1,232,HD22,111-OG1-232-HD22

295,111,O,114,H,111-O-114-H

296,112,O,114,H,112-O-114-H

297,112,O,115,H,112-O-115-H

298,113,O,118,H,113-O-118-H

299,114,O,255,HD21,114-O-255-HD21

300,114,O,117,H,114-O-117-H

301,114,O,118,H,114-O-118-H

302,115,O,118,H,115-O-118-H

303,115,O,119,H,115-O-119-H

304,115,O,120,H,115-O-120-H

305,116,O,120,H,116-O-120-H

306,116,O,119,H,116-O-119-H

307,116,OG,309,HD21,116-OG-309-HD21

308,116,OG,516,H10,116-OG-516-H10

309,116,O,309,HD21,116-O-309-HD21

310,116,O,119,HE21,116-O-119-HE21

311,116,O,309,HD22,116-O-309-HD22

312,117,O,119,HE21,117-O-119-HE21

313,117,O,119,H,117-O-119-H

314,117,OD1,119,HE21,117-OD1-119-HE21

315,117,OD1,306,HZ1,117-OD1-306-HZ1

316,117,OD1,255,HD21,117-OD1-255-HD21

317,117,OD1,309,HD22,117-OD1-309-HD22

318,117,O,117,HD22,117-O-117-HD22

319,117,O,306,HZ3,117-O-306-HZ3

320,117,OD1,306,HZ2,117-OD1-306-HZ2

321,117,O,306,HZ1,117-O-306-HZ1

322,117,OD1,306,HZ3,117-OD1-306-HZ3

323,117,O,306,HZ2,117-O-306-HZ2

324,118,O,119,HE21,118-O-119-HE21

325,118,O,124,HG,118-O-124-HG

326,119,OE1,306,HZ3,119-OE1-306-HZ3

327,119,OE1,134,HH12,119-OE1-134-HH12

328,119,OE1,306,HZ1,119-OE1-306-HZ1

329,119,OE1,306,HZ2,119-OE1-306-HZ2

330,119,NE2,306,HZ2,119-NE2-306-HZ2

331,119,OE1,259,HH,119-OE1-259-HH

332,119,O,134,HH22,119-O-134-HH22

333,119,O,309,HD22,119-O-309-HD22

334,119,O,309,HD21,119-O-309-HD21

335,119,O,134,HH12,119-O-134-HH12

336,119,NE2,309,HD22,119-NE2-309-HD22

337,119,OE1,309,HD21,119-OE1-309-HD21

338,119,OE1,309,HD22,119-OE1-309-HD22

339,119,OE1,124,HG,119-OE1-124-HG

340,119,OE1,117,HD22,119-OE1-117-HD22

341,120,OG,116,HG,120-OG-116-HG

342,120,O,134,HH22,120-O-134-HH22

343,120,O,134,HH12,120-O-134-HH12

344,120,OG,122,H,120-OG-122-H

345,120,O,124,HG,120-O-124-HG

346,120,O,124,H,120-O-124-H

347,120,O,123,H,120-O-123-H

348,120,OG,123,H,120-OG-123-H

349,121,O,131,HE1,121-O-131-HE1

350,121,O,106,HH21,121-O-106-HH21

351,121,N,134,HH12,121-N-134-HH12

352,121,O,124,H,121-O-124-H

353,121,O,124,HG,121-O-124-HG

354,121,O,106,HE,121-O-106-HE

355,121,O,455,HH12,121-O-455-HH12

356,122,OG,106,HH21,122-OG-106-HH21

357,122,O,106,HH21,122-O-106-HH21

358,122,O,106,HE,122-O-106-HE

359,124,O,126,H,124-O-126-H

360,124,OG,126,H,124-OG-126-H

361,125,O,106,H,125-O-106-H

362,125,O,127,HG,125-O-127-HG

363,126,OD2,306,HZ2,126-OD2-306-HZ2

364,126,OD2,131,H,126-OD2-131-H

365,126,O,124,HG,126-O-124-HG

366,126,OD1,306,HZ2,126-OD1-306-HZ2

367,126,OD1,131,H,126-OD1-131-H

368,126,OD1,128,H,126-OD1-128-H

369,126,OD2,128,H,126-OD2-128-H

370,126,O,127,HG,126-O-127-HG

371,126,OD1,126,H,126-OD1-126-H

372,126,OD1,127,H,126-OD1-127-H

373,126,O,455,HH22,126-O-455-HH22

374,126,OD1,119,HE22,126-OD1-119-HE22

375,126,OD1,306,HZ3,126-OD1-306-HZ3

376,126,OD1,134,HH11,126-OD1-134-HH11

377,126,OD2,134,HH11,126-OD2-134-HH11

378,126,OD1,124,HG,126-OD1-124-HG

379,126,OD2,127,H,126-OD2-127-H

380,127,OG,104,HZ3,127-OG-104-HZ3

381,127,OG,104,HZ1,127-OG-104-HZ1

382,127,O,104,HZ2,127-O-104-HZ2

383,127,O,104,HZ3,127-O-104-HZ3

384,127,O,104,HZ1,127-O-104-HZ1

385,127,OG,104,HZ2,127-OG-104-HZ2

386,127,OG,455,HH21,127-OG-455-HH21

387,128,O,132,H,128-O-132-H

388,128,O,131,H,128-O-131-H

389,129,O,133,H,129-O-133-H

390,129,O,297,HD22,129-O-297-HD22

391,130,O,133,H,130-O-133-H

392,130,O,134,H,130-O-134-H

393,130,O,299,HD21,130-O-299-HD21

394,130,O,297,HD22,130-O-297-HD22

395,131,O,134,H,131-O-134-H

396,131,O,135,H,131-O-135-H

397,132,O,136,H,132-O-136-H

398,133,O,137,H,133-O-137-H

399,133,O,136,H,133-O-136-H

400,133,O,297,HD22,133-O-297-HD22

401,134,O,138,H,134-O-138-H

402,135,O,139,H,135-O-139-H

403,135,O,138,H,135-O-138-H

404,135,O,139,HE21,135-O-139-HE21

405,136,O,139,H,136-O-139-H

406,136,O,140,HD22,136-O-140-HD22

407,136,O,140,H,136-O-140-H

408,137,O,141,H,137-O-141-H

409,138,O,142,H,138-O-142-H

410,139,OE1,143,HZ1,139-OE1-143-HZ1

411,139,OE1,143,HZ2,139-OE1-143-HZ2

412,139,OE1,135,HE,139-OE1-135-HE

413,139,OE1,136,HH11,139-OE1-136-HH11

414,139,OE1,143,HZ3,139-OE1-143-HZ3

415,139,O,143,HZ1,139-O-143-HZ1

416,139,O,143,H,139-O-143-H

417,139,O,143,HZ2,139-O-143-HZ2

418,139,OE1,136,HH22,139-OE1-136-HH22

419,139,O,139,HE21,139-O-139-HE21

420,139,O,143,HZ3,139-O-143-HZ3

421,139,OE1,135,HH12,139-OE1-135-HH12

422,139,OE1,135,HH11,139-OE1-135-HH11

423,139,OE1,135,HH21,139-OE1-135-HH21

424,140,OD1,143,HZ2,140-OD1-143-HZ2

425,140,OD1,143,HZ1,140-OD1-143-HZ1

426,140,OD1,136,HH12,140-OD1-136-HH12

427,140,OD1,143,HZ3,140-OD1-143-HZ3

428,140,OD1,139,HE21,140-OD1-139-HE21

429,140,O,143,H,140-O-143-H

430,140,OD1,136,HE,140-OD1-136-HE

431,140,OD1,139,HE22,140-OD1-139-HE22

432,140,OD1,136,HH21,140-OD1-136-HH21

433,140,O,144,H,140-O-144-H

434,140,O,144,HG,140-O-144-HG

435,141,O,144,HG,141-O-144-HG

436,141,O,145,H,141-O-145-H

437,141,O,144,H,141-O-144-H

438,142,O,146,HG,142-O-146-HG

439,142,O,146,H,142-O-146-H

440,142,O,147,H,142-O-147-H

441,142,O,145,H,142-O-145-H

442,143,O,146,H,143-O-146-H

443,143,O,148,H,143-O-148-H

444,143,O,147,H,143-O-147-H

445,144,O,144,HG,144-O-144-HG

446,144,O,158,HH,144-O-158-HH

447,145,O,162,HE2,145-O-162-HE2

448,145,O,146,HG,145-O-146-HG

449,146,OG,147,H,146-OG-147-H

450,146,O,159,H,146-O-159-H

451,146,O,146,HG,146-O-146-HG

452,148,O,158,H,148-O-158-H

453,149,O,149,HG,149-O-149-HG

454,149,OG,154,HG,149-OG-154-HG

455,150,OD2,153,HG,150-OD2-153-HG

456,150,O,153,H,150-O-153-H

457,150,OD1,152,H,150-OD1-152-H

458,150,OD1,158,HH,150-OD1-158-HH

459,150,O,154,HG,150-O-154-HG

460,150,OD1,153,HG,150-OD1-153-HG

461,150,O,152,H,150-O-152-H

462,150,OD1,153,H,150-OD1-153-H

463,150,OD2,153,H,150-OD2-153-H

464,150,OD2,152,H,150-OD2-152-H

465,150,O,149,HG,150-O-149-HG

466,151,O,153,H,151-O-153-H

467,152,O,154,H,152-O-154-H

468,153,OG,156,H,153-OG-156-H

469,153,O,156,H,153-O-156-H

470,153,OG,154,H,153-OG-154-H

471,153,OG,155,H,153-OG-155-H

472,153,O,153,HG,153-O-153-HG

473,153,O,155,H,153-O-155-H

474,153,OG,156,HG,153-OG-156-HG

475,154,O,154,HG,154-O-154-HG

476,154,O,149,HG,154-O-149-HG

477,154,OG,155,HG1,154-OG-155-HG1

478,154,OG,155,H,154-OG-155-H

479,154,O,155,HG1,154-O-155-HG1

480,155,O,358,HH22,155-O-358-HH22

481,155,OG1,358,HH12,155-OG1-358-HH12

482,155,O,358,HH21,155-O-358-HH21

483,155,O,149,HG,155-O-149-HG

484,155,OG1,156,HG,155-OG1-156-HG

485,155,OG1,360,HG,155-OG1-360-HG

486,155,OG1,154,HG,155-OG1-154-HG

487,155,OG1,153,HG,155-OG1-153-HG

488,155,O,155,HG1,155-O-155-HG1

489,155,OG1,358,HH22,155-OG1-358-HH22

490,156,O,153,HG,156-O-153-HG

491,156,OG,358,HH12,156-OG-358-HH12

492,156,OG,358,HH22,156-OG-358-HH22

493,156,O,149,HG,156-O-149-HG

494,156,OG,358,HH21,156-OG-358-HH21

495,156,OG,356,HH12,156-OG-356-HH12

496,156,OG,155,HG1,156-OG-155-HG1

497,156,OG,153,HG,156-OG-153-HG

498,156,OG,356,HH22,156-OG-356-HH22

499,156,O,150,H,156-O-150-H

500,156,OG,356,HH21,156-OG-356-HH21

501,156,OG,157,H,156-OG-157-H

502,157,O,161,H,157-O-161-H

503,157,O,160,H,157-O-160-H

504,157,O,156,HG,157-O-156-HG

505,158,O,161,H,158-O-161-H

506,158,O,162,H,158-O-162-H

507,159,O,163,H,159-O-163-H

508,159,O,162,H,159-O-162-H

509,160,OE1,356,HE,160-OE1-356-HE

510,160,OE1,359,H,160-OE1-359-H

511,160,OE2,356,HH22,160-OE2-356-HH22

512,160,O,164,H,160-O-164-H

513,160,OE2,358,HE,160-OE2-358-HE

514,160,OE2,358,HH11,160-OE2-358-HH11

515,160,O,164,HG,160-O-164-HG

516,160,OE2,157,H,160-OE2-157-H

517,160,OE1,156,HG,160-OE1-156-HG

518,160,OE2,356,HE,160-OE2-356-HE

519,160,OE2,358,HH21,160-OE2-358-HH21

520,160,OE1,356,HH12,160-OE1-356-HH12

521,160,OE1,356,HH21,160-OE1-356-HH21

522,160,OE2,156,HG,160-OE2-156-HG

523,160,OE2,359,H,160-OE2-359-H

524,160,OE2,356,HH12,160-OE2-356-HH12

525,160,OE1,356,HH22,160-OE1-356-HH22

526,160,OE1,358,HH11,160-OE1-358-HH11

527,160,OE1,157,H,160-OE1-157-H

528,160,OE2,356,HH21,160-OE2-356-HH21

529,160,OE1,358,HH21,160-OE1-358-HH21

530,160,OE1,358,HE,160-OE1-358-HE

531,161,OE2,165,HZ3,161-OE2-165-HZ3

532,161,OE2,165,HZ2,161-OE2-165-HZ2

533,161,OE1,153,HG,161-OE1-153-HG

534,161,OE1,165,HZ1,161-OE1-165-HZ1

535,161,O,165,HZ2,161-O-165-HZ2

536,161,OE1,356,HH22,161-OE1-356-HH22

537,161,OE2,156,HG,161-OE2-156-HG

538,161,OE2,356,HH12,161-OE2-356-HH12

539,161,OE1,356,HH12,161-OE1-356-HH12

540,161,OE2,356,HH21,161-OE2-356-HH21

541,161,O,165,HZ3,161-O-165-HZ3

542,161,O,165,H,161-O-165-H

543,161,OE1,165,HZ2,161-OE1-165-HZ2

544,161,OE1,356,HH21,161-OE1-356-HH21

545,161,OE2,153,HG,161-OE2-153-HG

546,161,OE1,358,HH12,161-OE1-358-HH12

547,161,OE2,358,HH12,161-OE2-358-HH12

548,161,OE1,165,HZ3,161-OE1-165-HZ3

549,161,OE1,156,HG,161-OE1-156-HG

550,161,OE1,358,HH22,161-OE1-358-HH22

551,161,O,165,HZ1,161-O-165-HZ1

552,161,OE2,356,HH22,161-OE2-356-HH22

553,162,O,166,H,162-O-166-H

554,162,ND1,165,HZ2,162-ND1-165-HZ2

555,162,ND1,165,HZ1,162-ND1-165-HZ1

556,162,ND1,165,HZ3,162-ND1-165-HZ3

557,162,O,165,H,162-O-165-H

558,163,O,167,H,163-O-167-H

559,163,O,166,H,163-O-166-H

560,164,OG,356,HE,164-OG-356-HE

561,164,O,168,H,164-O-168-H

562,164,OG,356,HH21,164-OG-356-HH21

563,164,OG,356,HH11,164-OG-356-HH11

564,165,O,169,H,165-O-169-H

565,165,O,168,H,165-O-168-H

566,166,O,193,HG,166-O-193-HG

567,166,OE2,206,HH21,166-OE2-206-HH21

568,166,OE2,165,HZ2,166-OE2-165-HZ2

569,166,O,169,H,166-O-169-H

570,166,OE2,206,HE,166-OE2-206-HE

571,166,OE1,206,HH21,166-OE1-206-HH21

572,166,O,170,H,166-O-170-H

573,166,OE2,165,HZ1,166-OE2-165-HZ1

574,166,OE1,196,HD22,166-OE1-196-HD22

575,166,OE1,206,HE,166-OE1-206-HE

576,166,OE2,165,HZ3,166-OE2-165-HZ3

577,167,O,171,H,167-O-171-H

578,168,OE1,477,HH22,168-OE1-477-HH22

579,168,OE2,477,HH12,168-OE2-477-HH12

580,168,OE2,356,HH21,168-OE2-356-HH21

581,168,O,172,HG,168-O-172-HG

582,168,OE2,477,HH21,168-OE2-477-HH21

583,168,OE1,477,HE,168-OE1-477-HE

584,168,OE1,477,HH21,168-OE1-477-HH21

585,168,OE2,356,HE,168-OE2-356-HE

586,168,OE2,477,HE,168-OE2-477-HE

587,168,O,171,H,168-O-171-H

588,168,OE1,477,HH11,168-OE1-477-HH11

589,168,OE2,356,HH11,168-OE2-356-HH11

590,168,OE1,356,HE,168-OE1-356-HE

591,168,OE1,356,HH21,168-OE1-356-HH21

592,168,OE2,477,HH22,168-OE2-477-HH22

593,168,OE1,477,HH12,168-OE1-477-HH12

594,168,OE1,356,HH11,168-OE1-356-HH11

595,168,O,172,H,168-O-172-H

596,168,OE2,477,HH11,168-OE2-477-HH11

597,169,O,173,HG1,169-O-173-HG1

598,169,O,172,HG,169-O-172-HG

599,169,O,173,H,169-O-173-H

600,169,O,172,H,169-O-172-H

601,170,O,174,H,170-O-174-H

602,170,O,173,H,170-O-173-H

603,171,O,173,H,171-O-173-H

604,171,O,175,H,171-O-175-H

605,171,O,175,HE21,171-O-175-HE21

606,172,O,175,H,172-O-175-H

607,172,O,176,H,172-O-176-H

608,172,O,175,HE21,172-O-175-HE21

609,173,OG1,209,HE2,173-OG1-209-HE2

610,173,O,176,H,173-O-176-H

611,173,O,177,H,173-O-177-H

612,174,O,177,H,174-O-177-H

613,174,O,178,H,174-O-178-H

614,175,OE1,477,HH11,175-OE1-477-HH11

615,175,OE1,510,H,175-OE1-510-H

616,175,O,175,HE21,175-O-175-HE21

617,175,O,179,H,175-O-179-H

618,175,O,178,H,175-O-178-H

619,176,O,179,H,176-O-179-H

620,176,OE1,175,HE22,176-OE1-175-HE22

621,176,OE1,175,HE21,176-OE1-175-HE21

622,176,O,180,H,176-O-180-H

623,176,OE2,175,HE22,176-OE2-175-HE22

624,176,OE2,172,HG,176-OE2-172-HG

625,177,O,180,H,177-O-180-H

626,177,O,182,H,177-O-182-H

627,178,O,180,H,178-O-180-H

628,180,O,182,H,180-O-182-H

629,181,O,183,H,181-O-183-H

630,182,O,507,HE21,182-O-507-HE21

631,182,O,508,H,182-O-508-H

632,183,ND1,507,HE21,183-ND1-507-HE21

633,183,ND1,183,H,183-ND1-183-H

634,184,O,505,H,184-O-505-H

635,184,O,506,H,184-O-506-H

636,185,OD1,187,H,185-OD1-187-H

637,185,OD1,188,H,185-OD1-188-H

638,185,O,187,H,185-O-187-H

639,185,O,189,H,185-O-189-H

640,186,O,189,H,186-O-189-H

641,186,O,190,H,186-O-190-H

642,186,O,327,HG,186-O-327-HG

643,187,OH,499,HZ2,187-OH-499-HZ2

644,187,O,191,H,187-O-191-H

645,187,OH,499,HZ1,187-OH-499-HZ1

646,187,O,190,H,187-O-190-H

647,187,OH,219,HD22,187-OH-219-HD22

648,187,OH,499,HZ3,187-OH-499-HZ3

649,187,OH,324,HG1,187-OH-324-HG1

650,188,O,219,HD21,188-O-219-HD21

651,189,O,192,H,189-O-192-H

652,189,OH,188,HH21,189-OH-188-HH21

653,189,O,193,H,189-O-193-H

654,189,OH,188,HE,189-OH-188-HE

655,189,O,209,HE2,189-O-209-HE2

656,189,OH,188,HH11,189-OH-188-HH11

657,189,O,193,HG,189-O-193-HG

658,190,O,194,H,190-O-194-H

659,190,O,193,HG,190-O-193-HG

660,190,O,193,H,190-O-193-H

661,191,O,195,H,191-O-195-H

662,191,O,195,HG1,191-O-195-HG1

663,192,O,196,H,192-O-196-H

664,192,O,196,HD22,192-O-196-HD22

665,193,O,197,H,193-O-197-H

666,193,O,196,H,193-O-196-H

667,193,OG,209,HE2,193-OG-209-HE2

668,194,O,197,H,194-O-197-H

669,194,O,198,H,194-O-198-H

670,195,O,199,H,195-O-199-H

671,196,O,200,H,196-O-200-H

672,196,OD1,207,H,196-OD1-207-H

673,197,O,201,H,197-O-201-H

674,197,O,200,H,197-O-200-H

675,198,O,202,H,198-O-202-H

676,199,O,205,H,199-O-205-H

677,199,O,204,H,199-O-204-H

678,199,O,203,H,199-O-203-H

679,200,O,204,H,200-O-204-H

680,201,O,281,H,201-O-281-H

681,202,O,282,HG1,202-O-282-HG1

682,202,O,282,H,202-O-282-H

683,202,O,281,H,202-O-281-H

684,203,O,205,HE,203-O-205-HE

685,203,O,205,HH11,203-O-205-HH11

686,203,O,205,HH21,203-O-205-HH21

687,203,O,205,H,203-O-205-H

688,204,O,205,HE,204-O-205-HE

689,204,O,205,HH11,204-O-205-HH11

690,204,O,205,HH21,204-O-205-HH21

691,206,O,205,HH11,206-O-205-HH11

692,206,O,205,HH12,206-O-205-HH12

693,207,O,196,HD21,207-O-196-HD21

694,208,OD2,209,H,208-OD2-209-H

695,208,O,211,H,208-O-211-H

696,208,OD1,206,HH22,208-OD1-206-HH22

697,208,OD1,210,HD21,208-OD1-210-HD21

698,208,OD1,210,H,208-OD1-210-H

699,208,OD2,210,HD21,208-OD2-210-HD21

700,208,OD1,209,H,208-OD1-209-H

701,208,OD2,206,HH22,208-OD2-206-HH22

702,208,OD1,210,HD22,208-OD1-210-HD22

703,208,OD2,210,H,208-OD2-210-H

704,208,OD2,206,HH11,208-OD2-206-HH11

705,208,OD2,206,HH12,208-OD2-206-HH12

706,208,OD2,210,HD22,208-OD2-210-HD22

707,208,OD2,208,H,208-OD2-208-H

708,208,OD1,208,H,208-OD1-208-H

709,208,O,210,H,208-O-210-H

710,208,OD1,206,HH12,208-OD1-206-HH12

711,209,ND1,173,HG1,209-ND1-173-HG1

712,209,O,212,HE21,209-O-212-HE21

713,209,O,211,H,209-O-211-H

714,209,O,210,HD22,209-O-210-HD22

715,209,ND1,209,H,209-ND1-209-H

716,209,O,212,HE22,209-O-212-HE22

717,210,O,210,HD22,210-O-210-HD22

718,210,O,212,HE21,210-O-212-HE21

719,210,OD1,210,H,210-OD1-210-H

720,210,OD1,212,HE21,210-OD1-212-HE21

721,210,O,212,H,210-O-212-H

722,211,ND1,213,H,211-ND1-213-H

723,211,O,215,H,211-O-215-H

724,211,ND1,214,H,211-ND1-214-H

725,211,ND1,208,H,211-ND1-208-H

726,211,O,214,H,211-O-214-H

727,212,O,216,HG,212-O-216-HG

728,212,O,212,HE21,212-O-212-HE21

729,212,OE1,212,H,212-OE1-212-H

730,212,O,216,H,212-O-216-H

731,212,O,215,H,212-O-215-H

732,212,OE1,216,HG,212-OE1-216-HG

733,213,OE1,212,HE22,213-OE1-212-HE22

734,213,O,257,HZ1,213-O-257-HZ1

735,213,O,257,HZ2,213-O-257-HZ2

736,213,OE1,257,HZ2,213-OE1-257-HZ2

737,213,OE1,213,H,213-OE1-213-H

738,213,OE2,264,HZ1,213-OE2-264-HZ1

739,213,OE1,216,HG,213-OE1-216-HG

740,213,OE2,257,HZ1,213-OE2-257-HZ1

741,213,OE2,264,HZ3,213-OE2-264-HZ3

742,213,OE2,264,HZ2,213-OE2-264-HZ2

743,213,OE1,264,HZ3,213-OE1-264-HZ3

744,213,OE1,264,HZ1,213-OE1-264-HZ1

745,213,O,216,H,213-O-216-H

746,213,OE1,211,HE2,213-OE1-211-HE2

747,213,OE1,257,HZ3,213-OE1-257-HZ3

748,213,OE2,211,HE2,213-OE2-211-HE2

749,213,OE1,264,HZ2,213-OE1-264-HZ2

750,213,OE1,257,HZ1,213-OE1-257-HZ1

751,213,OE2,260,HG,213-OE2-260-HG

752,213,O,217,H,213-O-217-H

753,213,OE2,213,H,213-OE2-213-H

754,213,OE1,212,HE21,213-OE1-212-HE21

755,213,OE2,257,HZ2,213-OE2-257-HZ2

756,213,OE2,207,HH,213-OE2-207-HH

757,213,O,216,HG,213-O-216-HG

758,213,OE2,257,HZ3,213-OE2-257-HZ3

759,214,O,218,H,214-O-218-H

760,214,O,217,H,214-O-217-H

761,215,O,219,H,215-O-219-H

762,215,O,218,H,215-O-218-H

763,215,O,220,H,215-O-220-H

764,215,O,217,H,215-O-217-H

765,216,OG,221,HD22,216-OG-221-HD22

766,216,O,220,H,216-O-220-H

767,216,OG,257,HZ3,216-OG-257-HZ3

768,216,O,222,HD22,216-O-222-HD22

769,216,OG,257,HZ2,216-OG-257-HZ2

770,216,O,257,HZ3,216-O-257-HZ3

771,216,OG,257,HZ1,216-OG-257-HZ1

772,216,OG,212,HE21,216-OG-212-HE21

773,216,O,221,HD22,216-O-221-HD22

774,216,O,257,HZ1,216-O-257-HZ1

775,216,O,216,HG,216-O-216-HG

776,216,O,219,H,216-O-219-H

777,216,O,257,HZ2,216-O-257-HZ2

778,217,O,222,HD21,217-O-222-HD21

779,217,O,222,HD22,217-O-222-HD22

780,218,O,219,HD22,218-O-219-HD22

781,218,O,222,HD22,218-O-222-HD22

782,218,O,222,HD21,218-O-222-HD21

783,219,O,222,HD22,219-O-222-HD22

784,219,O,222,H,219-O-222-H

785,219,OD1,188,HH11,219-OD1-188-HH11

786,219,O,219,HD22,219-O-219-HD22

787,219,O,221,H,219-O-221-H

788,219,O,222,HD21,219-O-222-HD21

789,219,OD1,188,HE,219-OD1-188-HE

790,219,OD1,188,HH21,219-OD1-188-HH21

791,220,O,257,HZ3,220-O-257-HZ3

792,220,O,222,H,220-O-222-H

793,220,O,221,HD22,220-O-221-HD22

794,220,O,257,HZ1,220-O-257-HZ1

795,220,O,222,HD21,220-O-222-HD21

796,221,O,223,H,221-O-223-H

797,221,O,221,HD22,221-O-221-HD22

798,221,O,223,HD22,221-O-223-HD22

799,221,O,257,HZ3,221-O-257-HZ3

800,221,O,257,HZ1,221-O-257-HZ1

801,221,OD1,223,HD22,221-OD1-223-HD22

802,221,OD1,216,HG,221-OD1-216-HG

803,221,O,257,HZ2,221-O-257-HZ2

804,222,OD1,220,H,222-OD1-220-H

805,222,O,222,HD21,222-O-222-HD21

806,222,OD1,225,H,222-OD1-225-H

807,222,OD1,223,H,222-OD1-223-H

808,222,OD1,224,H,222-OD1-224-H

809,222,O,224,H,222-O-224-H

810,223,O,227,H,223-O-227-H

811,223,O,226,H,223-O-226-H

812,223,OD1,247,HG,223-OD1-247-HG

813,223,O,223,HD22,223-O-223-HD22

814,223,OD1,223,H,223-OD1-223-H

815,224,O,227,H,224-O-227-H

816,224,O,228,H,224-O-228-H

817,225,O,496,H,225-O-496-H

818,226,O,247,HG,226-O-247-HG

819,226,OE1,57,HZ2,226-OE1-57-HZ2

820,226,OE1,57,HZ3,226-OE1-57-HZ3

821,226,OE2,57,HZ3,226-OE2-57-HZ3

822,226,OE1,247,HG,226-OE1-247-HG

823,226,OE1,223,HD22,226-OE1-223-HD22

824,226,OE2,57,HZ2,226-OE2-57-HZ2

825,226,OE1,226,H,226-OE1-226-H

826,226,OE2,222,HD22,226-OE2-222-HD22

827,226,OE2,57,HZ1,226-OE2-57-HZ1

828,226,OE2,247,HG,226-OE2-247-HG

829,226,OE1,57,HZ1,226-OE1-57-HZ1

830,226,O,232,HD21,226-O-232-HD21

831,226,OE2,223,HD22,226-OE2-223-HD22

832,227,O,232,HD22,227-O-232-HD22

833,227,O,232,HD21,227-O-232-HD21

834,228,O,496,H,228-O-496-H

835,229,O,231,H,229-O-231-H

836,229,O,232,HD22,229-O-232-HD22

837,229,O,232,H,229-O-232-H

838,230,O,494,HH,230-O-494-HH

839,230,O,230,HG,230-O-230-HG

840,230,OG,494,HH,230-OG-494-HH

841,231,O,229,H,231-O-229-H

842,232,O,235,H,232-O-235-H

843,232,OD1,235,H,232-OD1-235-H

844,232,OD1,234,H,232-OD1-234-H

845,232,OD1,494,HH,232-OD1-494-HH

846,232,O,236,H,232-O-236-H

847,233,O,237,H,233-O-237-H

848,233,O,236,H,233-O-236-H

849,234,O,241,HH11,234-O-241-HH11

850,234,O,237,H,234-O-237-H

851,235,OD2,241,HH11,235-OD2-241-HH11

852,235,OD1,110,H,235-OD1-110-H

853,235,OD1,235,H,235-OD1-235-H

854,235,OD2,110,H,235-OD2-110-H

855,235,OD1,111,H,235-OD1-111-H

856,235,O,241,HH12,235-O-241-HH12

857,235,OD2,232,HD22,235-OD2-232-HD22

858,235,OD1,111,HG1,235-OD1-111-HG1

859,235,O,241,HH11,235-O-241-HH11

860,235,OD1,232,HD22,235-OD1-232-HD22

861,235,OD2,235,H,235-OD2-235-H

862,235,OD2,111,H,235-OD2-111-H

863,235,OD1,232,H,235-OD1-232-H

864,235,OD2,111,HG1,235-OD2-111-HG1

865,236,O,241,HH12,236-O-241-HH12

866,236,O,80,H,236-O-80-H

867,237,O,240,H,237-O-240-H

868,238,O,242,H,238-O-242-H

869,238,O,241,H,238-O-241-H

870,239,O,242,H,239-O-242-H

871,239,O,243,H,239-O-243-H

872,239,O,241,H,239-O-241-H

873,240,O,243,H,240-O-243-H

874,240,O,245,HD22,240-O-245-HD22

875,240,O,245,H,240-O-245-H

876,243,O,245,HD22,243-O-245-HD22

877,243,O,245,H,243-O-245-H

878,244,O,249,HD21,244-O-249-HD21

879,244,O,249,HD22,244-O-249-HD22

880,245,OD1,247,H,245-OD1-247-H

881,245,O,249,H,245-O-249-H

882,245,ND2,245,H,245-ND2-245-H

883,245,OD1,247,HG,245-OD1-247-HG

884,245,O,249,HD22,245-O-249-HD22

885,245,O,248,H,245-O-248-H

886,245,OD1,248,H,245-OD1-248-H

887,246,O,249,HD22,246-O-249-HD22

888,246,O,250,H,246-O-250-H

889,246,O,249,H,246-O-249-H

890,247,OG,245,HD21,247-OG-245-HD21

891,247,O,223,HD21,247-O-223-HD21

892,247,OG,223,HD21,247-OG-223-HD21

893,247,OG,223,HD22,247-OG-223-HD22

894,247,O,251,H,247-O-251-H

895,247,O,250,H,247-O-250-H

896,248,O,251,H,248-O-251-H

897,248,O,252,H,248-O-252-H

898,248,O,110,HH,248-O-110-HH

899,249,O,252,HZ3,249-O-252-HZ3

900,249,OD1,252,HZ2,249-OD1-252-HZ2

901,249,O,253,H,249-O-253-H

902,249,O,252,H,249-O-252-H

903,249,OD1,252,HZ3,249-OD1-252-HZ3

904,249,OD1,245,H,249-OD1-245-H

905,249,O,249,HD22,249-O-249-HD22

906,249,OD1,252,HZ1,249-OD1-252-HZ1

907,249,O,252,HZ1,249-O-252-HZ1

908,250,O,253,H,250-O-253-H

909,250,O,223,HD21,250-O-223-HD21

910,250,O,221,HD21,250-O-221-HD21

911,250,O,254,H,250-O-254-H

912,251,O,255,H,251-O-255-H

913,251,O,255,HD22,251-O-255-HD22

914,252,O,256,H,252-O-256-H

915,252,O,255,H,252-O-255-H

916,253,OD1,257,HZ3,253-OD1-257-HZ3

917,253,OD1,257,HZ1,253-OD1-257-HZ1

918,253,OD2,252,HZ3,253-OD2-252-HZ3

919,253,OD2,257,HZ3,253-OD2-257-HZ3

920,253,OD1,252,HZ2,253-OD1-252-HZ2

921,253,OD2,257,HZ2,253-OD2-257-HZ2

922,253,OD1,252,HZ1,253-OD1-252-HZ1

923,253,OD1,252,HZ3,253-OD1-252-HZ3

924,253,O,257,HZ2,253-O-257-HZ2

925,253,OD2,257,HZ1,253-OD2-257-HZ1

926,253,O,256,H,253-O-256-H

927,253,OD2,252,HZ2,253-OD2-252-HZ2

928,253,OD1,257,HZ2,253-OD1-257-HZ2

929,253,O,257,H,253-O-257-H

930,253,O,257,HZ3,253-O-257-HZ3

931,253,OD2,252,HZ1,253-OD2-252-HZ1

932,254,O,258,H,254-O-258-H

933,254,O,257,H,254-O-257-H

934,255,OD1,116,H,255-OD1-116-H

935,255,OD1,117,H,255-OD1-117-H

936,255,O,259,H,255-O-259-H

937,256,OE2,252,HZ2,256-OE2-252-HZ2

938,256,OE1,252,HZ2,256-OE1-252-HZ2

939,256,O,260,HG,256-O-260-HG

940,256,OE1,117,HD21,256-OE1-117-HD21

941,256,OE1,252,HZ3,256-OE1-252-HZ3

942,256,OE2,255,HD22,256-OE2-255-HD22

943,256,OE1,252,HZ1,256-OE1-252-HZ1

944,256,OE2,252,HZ3,256-OE2-252-HZ3

945,256,OE1,255,HD22,256-OE1-255-HD22

946,256,OE2,117,HD21,256-OE2-117-HD21

947,256,O,260,H,256-O-260-H

948,256,OE2,260,HG,256-OE2-260-HG

949,256,OE2,252,HZ1,256-OE2-252-HZ1

950,256,O,259,H,256-O-259-H

951,257,O,261,H,257-O-261-H

952,257,O,260,HG,257-O-260-HG

953,258,O,261,H,258-O-261-H

954,258,O,262,H,258-O-262-H

955,259,OH,309,HD22,259-OH-309-HD22

956,259,O,263,H,259-O-263-H

957,259,O,262,H,259-O-262-H

958,259,OH,117,HD22,259-OH-117-HD22

959,259,OH,263,HE21,259-OH-263-HE21

960,259,OH,119,HE21,259-OH-119-HE21

961,259,OH,119,HE22,259-OH-119-HE22

962,259,OH,309,HD21,259-OH-309-HD21

963,259,O,263,HE21,259-O-263-HE21

964,260,O,264,HZ1,260-O-264-HZ1

965,260,OG,264,HZ2,260-OG-264-HZ2

966,260,O,264,HZ2,260-O-264-HZ2

967,260,OG,264,HZ1,260-OG-264-HZ1

968,260,O,264,H,260-O-264-H

969,261,O,265,H,261-O-265-H

970,261,O,264,H,261-O-264-H

971,262,O,266,H,262-O-266-H

972,262,O,265,H,262-O-265-H

973,263,OE1,259,HH,263-OE1-259-HH

974,263,OE1,267,HZ1,263-OE1-267-HZ1

975,263,O,267,H,263-O-267-H

976,263,O,267,HZ1,263-O-267-HZ1

977,263,O,266,H,263-O-266-H

978,263,O,263,HE21,263-O-263-HE21

979,263,O,267,HZ2,263-O-267-HZ2

980,263,OE1,267,HZ3,263-OE1-267-HZ3

981,263,O,267,HZ3,263-O-267-HZ3

982,263,OE1,267,HZ2,263-OE1-267-HZ2

983,264,O,268,H,264-O-268-H

984,264,O,267,H,264-O-267-H

985,265,O,205,HH22,265-O-205-HH22

986,265,O,269,H,265-O-269-H

987,265,O,268,H,265-O-268-H

988,266,O,269,H,266-O-269-H

989,266,O,270,H,266-O-270-H

990,267,O,271,HZ1,267-O-271-HZ1

991,267,O,271,HZ3,267-O-271-HZ3

992,267,O,270,H,267-O-270-H

993,267,O,271,H,267-O-271-H

994,267,O,271,HZ2,267-O-271-HZ2

995,268,OE1,205,HH22,268-OE1-205-HH22

996,268,O,272,H,268-O-272-H

997,268,OE1,267,HZ2,268-OE1-267-HZ2

998,268,OE1,267,HZ1,268-OE1-267-HZ1

999,268,OE2,205,HH12,268-OE2-205-HH12

1000,268,OE1,205,HH12,268-OE1-205-HH12

1001,268,OE2,205,HH22,268-OE2-205-HH22

1002,268,OE1,267,HZ3,268-OE1-267-HZ3

1003,268,OE2,267,HZ1,268-OE2-267-HZ1

1004,268,OE2,271,HZ1,268-OE2-271-HZ1

1005,268,OE2,267,HZ3,268-OE2-267-HZ3

1006,268,OE1,264,HZ3,268-OE1-264-HZ3

1007,268,OE2,271,HZ2,268-OE2-271-HZ2

1008,268,OE1,205,HH21,268-OE1-205-HH21

1009,268,OE1,207,HH,268-OE1-207-HH

1010,268,OE1,271,HZ3,268-OE1-271-HZ3

1011,268,OE2,205,HH11,268-OE2-205-HH11

1012,268,OE1,271,HZ1,268-OE1-271-HZ1

1013,268,OE1,205,HH11,268-OE1-205-HH11

1014,268,OE1,271,HZ2,268-OE1-271-HZ2

1015,268,OE2,264,HZ2,268-OE2-264-HZ2

1016,268,OE2,267,HZ2,268-OE2-267-HZ2

1017,268,OE2,205,HH21,268-OE2-205-HH21

1018,268,OE1,205,HE,268-OE1-205-HE

1019,268,O,205,HH22,268-O-205-HH22

1020,268,OE2,207,HH,268-OE2-207-HH

1021,268,OE2,264,HZ3,268-OE2-264-HZ3

1022,268,OE2,271,HZ3,268-OE2-271-HZ3

1023,268,OE1,264,HZ1,268-OE1-264-HZ1

1024,268,O,271,H,268-O-271-H

1025,268,OE1,264,HZ2,268-OE1-264-HZ2

1026,268,OE2,264,HZ1,268-OE2-264-HZ1

1027,268,O,205,HH21,268-O-205-HH21

1028,268,O,272,HG1,268-O-272-HG1

1029,269,O,272,H,269-O-272-H

1030,269,O,279,HH22,269-O-279-HH22

1031,269,O,273,H,269-O-273-H

1032,269,O,279,HH12,269-O-279-HH12

1033,269,ND1,205,HH21,269-ND1-205-HH21

1034,269,O,272,HG1,269-O-272-HG1

1035,269,ND1,205,HH12,269-ND1-205-HH12

1036,270,O,273,H,270-O-273-H

1037,270,OH,290,HE21,270-OH-290-HE21

1038,270,OH,271,HZ2,270-OH-271-HZ2

1039,270,OH,290,HE22,270-OH-290-HE22

1040,271,O,273,H,271-O-273-H

1041,272,O,274,H,272-O-274-H

1042,272,OG1,205,HH22,272-OG1-205-HH22

1043,272,O,279,HH22,272-O-279-HH22

1044,272,OG1,205,HH21,272-OG1-205-HH21

1045,272,O,272,HG1,272-O-272-HG1

1046,272,OG1,279,HH22,272-OG1-279-HH22

1047,272,OG1,205,HH12,272-OG1-205-HH12

1048,272,OG1,279,HH12,272-OG1-279-HH12

1049,272,O,279,HH21,272-O-279-HH21

1050,272,O,279,HH12,272-O-279-HH12

1051,274,OE1,275,HZ2,274-OE1-275-HZ2

1052,274,O,276,H,274-O-276-H

1053,274,OE2,275,HZ2,274-OE2-275-HZ2

1054,274,OE2,276,H,274-OE2-276-H

1055,274,OE1,275,HZ3,274-OE1-275-HZ3

1056,274,O,277,H,274-O-277-H

1057,274,OE1,276,H,274-OE1-276-H

1058,274,OE2,277,HE2,274-OE2-277-HE2

1059,274,OE2,275,H,274-OE2-275-H

1060,274,OE1,279,HH22,274-OE1-279-HH22

1061,274,OE2,275,HZ1,274-OE2-275-HZ1

1062,274,OE1,277,HE2,274-OE1-277-HE2

1063,274,OE1,275,H,274-OE1-275-H

1064,274,OE2,275,HZ3,274-OE2-275-HZ3

1065,275,O,277,H,275-O-277-H

1066,276,O,278,H,276-O-278-H

1067,277,O,279,HH21,277-O-279-HH21

1068,277,ND1,279,HH21,277-ND1-279-HH21

1069,277,O,279,HE,277-O-279-HE

1070,277,ND1,278,H,277-ND1-278-H

1071,277,ND1,279,HH11,277-ND1-279-HH11

1072,278,O,279,HE,278-O-279-HE

1073,280,OD2,282,HG1,280-OD2-282-HG1

1074,280,O,284,H,280-O-284-H

1075,280,OD1,269,HE2,280-OD1-269-HE2

1076,280,OD1,283,H,280-OD1-283-H

1077,280,OD1,282,HG1,280-OD1-282-HG1

1078,280,OD1,282,H,280-OD1-282-H

1079,280,O,283,H,280-O-283-H

1080,280,OD2,269,HE2,280-OD2-269-HE2

1081,280,O,284,HG,280-O-284-HG

1082,281,O,284,HG,281-O-284-HG

1083,281,O,284,H,281-O-284-H

1084,281,O,285,H,281-O-285-H

1085,282,O,285,H,282-O-285-H

1086,282,O,286,H,282-O-286-H

1087,283,OD2,279,HH21,283-OD2-279-HH21

1088,283,OD1,278,H,283-OD1-278-H

1089,283,OD1,279,H,283-OD1-279-H

1090,283,OD1,279,HH21,283-OD1-279-HH21

1091,283,OD2,278,H,283-OD2-278-H

1092,283,OD2,279,HE,283-OD2-279-HE

1093,283,OD1,279,HE,283-OD1-279-HE

1094,283,O,287,H,283-O-287-H

1095,283,OD2,279,H,283-OD2-279-H

1096,283,OD1,269,HE2,283-OD1-269-HE2

1097,284,O,288,H,284-O-288-H

1098,285,O,289,H,285-O-289-H

1099,285,O,288,H,285-O-288-H

1100,286,O,289,H,286-O-289-H

1101,286,O,290,HE21,286-O-290-HE21

1102,286,O,290,H,286-O-290-H

1103,286,O,290,HE22,286-O-290-HE22

1104,287,OE1,275,HZ2,287-OE1-275-HZ2

1105,287,OE2,275,HZ3,287-OE2-275-HZ3

1106,287,OE2,290,HE21,287-OE2-290-HE21

1107,287,OE1,290,HE21,287-OE1-290-HE21

1108,287,OE1,275,HZ3,287-OE1-275-HZ3

1109,287,OE2,275,HZ2,287-OE2-275-HZ2

1110,287,OE2,290,HE22,287-OE2-290-HE22

1111,287,O,291,H,287-O-291-H

1112,287,O,290,H,287-O-290-H

1113,287,O,290,HE21,287-O-290-HE21

1114,287,OE2,276,H,287-OE2-276-H

1115,287,OE1,290,HE22,287-OE1-290-HE22

1116,287,OE1,275,HZ1,287-OE1-275-HZ1

1117,287,OE1,276,H,287-OE1-276-H

1118,287,OE2,275,HZ1,287-OE2-275-HZ1

1119,288,O,291,H,288-O-291-H

1120,288,O,292,H,288-O-292-H

1121,289,O,293,HE21,289-O-293-HE21

1122,289,O,292,H,289-O-292-H

1123,289,O,293,H,289-O-293-H

1124,289,O,292,HZ1,289-O-292-HZ1

1125,289,O,292,HZ3,289-O-292-HZ3

1126,289,O,292,HZ2,289-O-292-HZ2

1127,289,O,293,HE22,289-O-293-HE22

1128,290,O,275,HZ3,290-O-275-HZ3

1129,290,O,293,HE22,290-O-293-HE22

1130,290,O,290,HE21,290-O-290-HE21

1131,290,O,294,H,290-O-294-H

1132,290,OE1,293,HE22,290-OE1-293-HE22

1133,290,OE1,275,HZ1,290-OE1-275-HZ1

1134,290,OE1,275,HZ3,290-OE1-275-HZ3

1135,290,OE1,275,HZ2,290-OE1-275-HZ2

1136,290,OE1,270,HH,290-OE1-270-HH

1137,290,O,293,HE21,290-O-293-HE21

1138,290,O,293,H,290-O-293-H

1139,290,O,292,H,290-O-292-H

1140,291,O,295,H,291-O-295-H

1141,291,OE1,293,HE22,291-OE1-293-HE22

1142,291,OE1,288,HE2,291-OE1-288-HE2

1143,291,OE1,290,HE21,291-OE1-290-HE21

1144,291,OE1,290,HE22,291-OE1-290-HE22

1145,291,OE2,290,HE21,291-OE2-290-HE21

1146,291,OE2,293,HE21,291-OE2-293-HE21

1147,291,OE1,275,HZ1,291-OE1-275-HZ1

1148,291,OE1,275,HZ3,291-OE1-275-HZ3

1149,291,O,294,H,291-O-294-H

1150,291,OE2,275,HZ3,291-OE2-275-HZ3

1151,291,OE2,293,HE22,291-OE2-293-HE22

1152,291,OE1,275,HZ2,291-OE1-275-HZ2

1153,291,OE2,275,HZ1,291-OE2-275-HZ1

1154,291,O,293,H,291-O-293-H

1155,291,OE2,275,HZ2,291-OE2-275-HZ2

1156,292,O,297,HD22,292-O-297-HD22

1157,292,O,295,H,292-O-295-H

1158,292,O,299,H,292-O-299-H

1159,293,O,293,HE21,293-O-293-HE21

1160,293,OE1,292,HZ2,293-OE1-292-HZ2

1161,293,OE1,292,HZ1,293-OE1-292-HZ1

1162,293,OE1,292,HZ3,293-OE1-292-HZ3

1163,293,OE1,293,H,293-OE1-293-H

1164,293,OE1,290,HE21,293-OE1-290-HE21

1165,293,O,295,H,293-O-295-H

1166,293,NE2,293,H,293-NE2-293-H

1167,294,O,288,HE2,294-O-288-HE2

1168,294,O,296,H,294-O-296-H

1169,295,OD2,136,HH21,295-OD2-136-HH21

1170,295,OD1,136,HH21,295-OD1-136-HH21

1171,295,OD2,297,HD21,295-OD2-297-HD21

1172,295,OD2,134,HH22,295-OD2-134-HH22

1173,295,OD1,299,H,295-OD1-299-H

1174,295,OD2,297,HD22,295-OD2-297-HD22

1175,295,OD1,297,H,295-OD1-297-H

1176,295,O,298,H,295-O-298-H

1177,295,OD2,297,H,295-OD2-297-H

1178,295,OD1,288,HE2,295-OD1-288-HE2

1179,295,OD1,299,HD22,295-OD1-299-HD22

1180,295,OD1,134,HH12,295-OD1-134-HH12

1181,295,OD1,296,H,295-OD1-296-H

1182,295,OD1,298,H,295-OD1-298-H

1183,295,OD2,134,HH12,295-OD2-134-HH12

1184,295,OD1,136,HH22,295-OD1-136-HH22

1185,295,OD1,134,HH22,295-OD1-134-HH22

1186,295,OD2,296,H,295-OD2-296-H

1187,295,OD2,299,HD22,295-OD2-299-HD22

1188,295,OD2,288,HE2,295-OD2-288-HE2

1189,295,OD2,134,HH21,295-OD2-134-HH21

1190,295,OD2,299,H,295-OD2-299-H

1191,295,OD1,297,HD21,295-OD1-297-HD21

1192,295,OD2,298,H,295-OD2-298-H

1193,296,OE1,136,HH21,296-OE1-136-HH21

1194,296,OE1,136,HE,296-OE1-136-HE

1195,296,OE2,297,HD21,296-OE2-297-HD21

1196,296,OE2,136,HH12,296-OE2-136-HH12

1197,296,OE1,136,HH11,296-OE1-136-HH11

1198,296,OE1,136,HH22,296-OE1-136-HH22

1199,296,OE2,136,HE,296-OE2-136-HE

1200,296,OE2,136,HH11,296-OE2-136-HH11

1201,296,OE2,297,HD22,296-OE2-297-HD22

1202,296,OE1,136,HH12,296-OE1-136-HH12

1203,296,OE2,296,H,296-OE2-296-H

1204,296,OE2,136,HH22,296-OE2-136-HH22

1205,296,O,136,HH22,296-O-136-HH22

1206,296,OE2,134,HE,296-OE2-134-HE

1207,296,O,299,HD21,296-O-299-HD21

1208,296,OE1,297,HD22,296-OE1-297-HD22

1209,296,O,136,HH12,296-O-136-HH12

1210,296,OE2,134,HH21,296-OE2-134-HH21

1211,296,OE2,297,H,296-OE2-297-H

1212,296,O,298,H,296-O-298-H

1213,296,OE1,296,H,296-OE1-296-H

1214,296,OE2,136,HH21,296-OE2-136-HH21

1215,297,OD1,136,HE,297-OD1-136-HE

1216,297,OD1,299,H,297-OD1-299-H

1217,297,OD1,134,HH21,297-OD1-134-HH21

1218,297,OD1,140,HD22,297-OD1-140-HD22

1219,297,O,136,HH22,297-O-136-HH22

1220,297,OD1,299,HD22,297-OD1-299-HD22

1221,297,OD1,140,HD21,297-OD1-140-HD21

1222,297,OD1,136,HH11,297-OD1-136-HH11

1223,297,O,299,HD22,297-O-299-HD22

1224,297,OD1,136,HH21,297-OD1-136-HH21

1225,297,O,297,HD21,297-O-297-HD21

1226,297,OD1,299,HD21,297-OD1-299-HD21

1227,297,OD1,136,HH22,297-OD1-136-HH22

1228,297,OD1,136,HH12,297-OD1-136-HH12

1229,297,OD1,134,HH11,297-OD1-134-HH11

1230,297,O,299,H,297-O-299-H

1231,297,O,136,HH21,297-O-136-HH21

1232,298,O,301,HE22,298-O-301-HE22

1233,298,O,300,H,298-O-300-H

1234,299,OD1,300,H,299-OD1-300-H

1235,299,OD1,134,HH21,299-OD1-134-HH21

1236,299,O,302,H,299-O-302-H

1237,299,OD1,302,H,299-OD1-302-H

1238,299,O,299,HD22,299-O-299-HD22

1239,299,O,292,HZ1,299-O-292-HZ1

1240,299,OD1,301,HE21,299-OD1-301-HE21

1241,299,O,292,HZ3,299-O-292-HZ3

1242,299,OD1,297,HD21,299-OD1-297-HD21

1243,299,ND2,301,HE22,299-ND2-301-HE22

1244,299,OD1,301,HE22,299-OD1-301-HE22

1245,299,O,301,H,299-O-301-H

1246,299,O,292,HZ2,299-O-292-HZ2

1247,299,OD1,301,H,299-OD1-301-H

1248,300,O,302,H,300-O-302-H

1249,300,O,292,HZ2,300-O-292-HZ2

1250,300,O,292,HZ1,300-O-292-HZ1

1251,300,O,292,HZ3,300-O-292-HZ3

1252,300,O,301,HE21,300-O-301-HE21

1253,301,O,306,HZ2,301-O-306-HZ2

1254,301,OE1,300,H,301-OE1-300-H

1255,301,OE1,306,HZ3,301-OE1-306-HZ3

1256,301,OE1,299,HD22,301-OE1-299-HD22

1257,301,O,306,HZ1,301-O-306-HZ1

1258,301,OE1,306,HZ2,301-OE1-306-HZ2

1259,301,O,306,HZ3,301-O-306-HZ3

1260,301,OE1,301,H,301-OE1-301-H

1261,301,O,303,H,301-O-303-H

1262,301,OE1,299,HD21,301-OE1-299-HD21

1263,301,O,299,HD21,301-O-299-HD21

1264,302,O,292,HZ1,302-O-292-HZ1

1265,302,O,292,HZ3,302-O-292-HZ3

1266,302,O,292,HZ2,302-O-292-HZ2

1267,303,OG,306,HZ1,303-OG-306-HZ1

1268,303,O,306,H,303-O-306-H

1269,303,OG,305,H,303-OG-305-H

1270,303,O,307,H,303-O-307-H

1271,303,OG,306,HZ2,303-OG-306-HZ2

1272,303,OG,306,H,303-OG-306-H

1273,303,OG,306,HZ3,303-OG-306-HZ3

1274,304,OD2,292,HZ2,304-OD2-292-HZ2

1275,304,OD1,304,H,304-OD1-304-H

1276,304,O,308,H,304-O-308-H

1277,304,OD1,292,HZ3,304-OD1-292-HZ3

1278,304,OD2,304,H,304-OD2-304-H

1279,304,OD2,305,H,304-OD2-305-H

1280,304,OD1,293,HE21,304-OD1-293-HE21

1281,304,O,307,H,304-O-307-H

1282,304,OD1,305,H,304-OD1-305-H

1283,304,OD1,270,HH,304-OD1-270-HH

1284,304,OD2,292,HZ3,304-OD2-292-HZ3

1285,304,OD2,292,HZ1,304-OD2-292-HZ1

1286,304,OD1,292,HZ1,304-OD1-292-HZ1

1287,304,OD2,270,HH,304-OD2-270-HH

1288,304,OD1,292,HZ2,304-OD1-292-HZ2

1289,305,OE1,309,HD21,305-OE1-309-HD21

1290,305,OE1,306,H,305-OE1-306-H

1291,305,OE1,309,HD22,305-OE1-309-HD22

1292,305,OE1,306,HZ2,305-OE1-306-HZ2

1293,305,OE2,117,HD21,305-OE2-117-HD21

1294,305,OE2,119,HE22,305-OE2-119-HE22

1295,305,OE2,303,HG,305-OE2-303-HG

1296,305,OE2,305,H,305-OE2-305-H

1297,305,OE2,309,HD21,305-OE2-309-HD21

1298,305,OE2,259,HH,305-OE2-259-HH

1299,305,OE1,119,HE21,305-OE1-119-HE21

1300,305,OE2,306,HZ3,305-OE2-306-HZ3

1301,305,OE1,119,HE22,305-OE1-119-HE22

1302,305,OE1,303,HG,305-OE1-303-HG

1303,305,OE1,259,HH,305-OE1-259-HH

1304,305,OE2,263,HE21,305-OE2-263-HE21

1305,305,OE1,306,HZ1,305-OE1-306-HZ1

1306,305,OE1,305,H,305-OE1-305-H

1307,305,OE1,263,HE21,305-OE1-263-HE21

1308,305,O,308,H,305-O-308-H

1309,305,OE1,267,HZ2,305-OE1-267-HZ2

1310,305,O,119,HE21,305-O-119-HE21

1311,305,OE1,117,HD21,305-OE1-117-HD21

1312,305,O,117,HD22,305-O-117-HD22

1313,305,OE2,309,HD22,305-OE2-309-HD22

1314,305,OE2,306,H,305-OE2-306-H

1315,305,O,119,HE22,305-O-119-HE22

1316,305,OE1,117,HD22,305-OE1-117-HD22

1317,305,OE2,306,HZ1,305-OE2-306-HZ1

1318,305,OE1,306,HZ3,305-OE1-306-HZ3

1319,305,OE2,263,HE22,305-OE2-263-HE22

1320,305,O,309,H,305-O-309-H

1321,305,OE1,263,HE22,305-OE1-263-HE22

1322,305,O,309,HD22,305-O-309-HD22

1323,305,OE2,117,HD22,305-OE2-117-HD22

1324,305,OE2,306,HZ2,305-OE2-306-HZ2

1325,306,O,134,HE,306-O-134-HE

1326,306,O,134,HH21,306-O-134-HH21

1327,306,O,309,H,306-O-309-H

1328,306,O,134,HH22,306-O-134-HH22

1329,306,O,134,HH12,306-O-134-HH12

1330,306,O,119,HE22,306-O-119-HE22

1331,307,O,310,H,307-O-310-H

1332,308,O,310,H,308-O-310-H

1333,308,O,311,H,308-O-311-H

1334,309,OD1,263,HE21,309-OD1-263-HE21

1335,309,OD1,263,HE22,309-OD1-263-HE22

1336,309,OD1,117,HD22,309-OD1-117-HD22

1337,309,OD1,134,HH22,309-OD1-134-HH22

1338,309,OD1,134,HH21,309-OD1-134-HH21

1339,309,ND2,119,HE22,309-ND2-119-HE22

1340,309,O,312,H,309-O-312-H

1341,309,OD1,119,HE22,309-OD1-119-HE22

1342,309,OD1,259,HH,309-OD1-259-HH

1343,309,O,313,H,309-O-313-H

1344,309,OD1,121,H,309-OD1-121-H

1345,309,OD1,134,HE,309-OD1-134-HE

1346,309,O,309,HD22,309-O-309-HD22

1347,309,OD1,134,HH12,309-OD1-134-HH12

1348,310,O,314,H,310-O-314-H

1349,310,N,134,HH21,310-N-134-HH21

1350,310,O,313,H,310-O-313-H

1351,311,O,315,H,311-O-315-H

1352,311,O,314,H,311-O-314-H

1353,312,O,516,H10,312-O-516-H10

1354,312,O,316,H,312-O-316-H

1355,313,OD1,134,HH12,313-OD1-134-HH12

1356,313,OD2,122,HG,313-OD2-122-HG

1357,313,OD1,516,H10,313-OD1-516-H10

1358,313,OD1,122,H,313-OD1-122-H

1359,313,OD1,309,HD22,313-OD1-309-HD22

1360,313,OD2,122,H,313-OD2-122-H

1361,313,OD1,134,HH22,313-OD1-134-HH22

1362,313,O,317,H,313-O-317-H

1363,313,OD2,116,HG,313-OD2-116-HG

1364,313,OD1,122,HG,313-OD1-122-HG

1365,313,OD2,121,H,313-OD2-121-H

1366,313,OD2,134,HH22,313-OD2-134-HH22

1367,313,OD2,134,HH12,313-OD2-134-HH12

1368,313,OD1,120,HG,313-OD1-120-HG

1369,313,OD1,116,HG,313-OD1-116-HG

1370,313,OD2,516,H10,313-OD2-516-H10

1371,313,OD1,309,HD21,313-OD1-309-HD21

1372,313,O,316,H,313-O-316-H

1373,313,OD2,120,HG,313-OD2-120-HG

1374,313,OD1,121,H,313-OD1-121-H

1375,314,O,317,H,314-O-317-H

1376,314,O,318,H,314-O-318-H

1377,315,O,318,H,315-O-318-H

1378,315,O,319,H,315-O-319-H

1379,316,O,516,H17,316-O-516-H17

1380,317,O,321,H,317-O-321-H

1381,317,O,320,H,317-O-320-H

1382,317,O,321,HG1,317-O-321-HG1

1383,318,O,321,HG1,318-O-321-HG1

1384,318,O,321,H,318-O-321-H

1385,318,O,322,H,318-O-322-H

1386,319,O,323,H,319-O-323-H

1387,319,O,323,HG1,319-O-323-HG1

1388,320,OD1,499,HZ3,320-OD1-499-HZ3

1389,320,O,324,HG1,320-O-324-HG1

1390,320,OD1,499,HZ2,320-OD1-499-HZ2

1391,320,OD2,499,HZ2,320-OD2-499-HZ2

1392,320,OD1,499,HZ1,320-OD1-499-HZ1

1393,320,O,323,H,320-O-323-H

1394,320,OD1,187,HH,320-OD1-187-HH

1395,320,OD2,499,HZ1,320-OD2-499-HZ1

1396,320,OD2,187,HH,320-OD2-187-HH

1397,320,OD1,497,HG1,320-OD1-497-HG1

1398,320,OD2,499,HZ3,320-OD2-499-HZ3

1399,320,OD2,497,HG1,320-OD2-497-HG1

1400,320,O,324,H,320-O-324-H

1401,320,OD2,219,HD21,320-OD2-219-HD21

1402,321,OG1,322,H,321-OG1-322-H

1403,321,O,325,H,321-O-325-H

1404,321,O,324,H,321-O-324-H

1405,322,O,326,H,322-O-326-H

1406,322,O,325,H,322-O-325-H

1407,323,O,326,H,323-O-326-H

1408,323,O,327,HG,323-O-327-HG

1409,323,O,327,H,323-O-327-H

1410,324,OG1,499,HZ2,324-OG1-499-HZ2

1411,324,O,327,HG,324-O-327-HG

1412,324,OG1,499,HZ1,324-OG1-499-HZ1

1413,324,OG1,497,HG1,324-OG1-497-HG1

1414,324,OG1,499,HZ3,324-OG1-499-HZ3

1415,324,O,328,H,324-O-328-H

1416,325,O,329,H,325-O-329-H

1417,325,O,329,HG,325-O-329-HG

1418,326,O,330,H,326-O-330-H

1419,326,O,329,HG,326-O-329-HG

1420,326,O,329,H,326-O-329-H

1421,327,O,331,H,327-O-331-H

1422,328,O,332,H,328-O-332-H

1423,328,O,331,H,328-O-331-H

1424,329,O,333,H,329-O-333-H

1425,330,O,334,H,330-O-334-H

1426,330,O,333,H,330-O-333-H

1427,331,O,334,H,331-O-334-H

1428,331,O,335,H,331-O-335-H

1429,332,O,335,H,332-O-335-H

1430,332,O,336,HD22,332-O-336-HD22

1431,332,O,336,H,332-O-336-H

1432,333,O,340,HE21,333-O-340-HE21

1433,333,O,336,H,333-O-336-H

1434,335,O,336,HD22,335-O-336-HD22

1435,336,OD1,339,H,336-OD1-339-H

1436,336,OD1,338,HE,336-OD1-338-HE

1437,336,O,340,H,336-O-340-H

1438,336,OD1,338,H,336-OD1-338-H

1439,336,O,339,H,336-O-339-H

1440,337,O,340,H,337-O-340-H

1441,337,O,341,H,337-O-341-H

1442,338,O,342,H,338-O-342-H

1443,339,O,343,H,339-O-343-H

1444,340,OE1,480,H,340-OE1-480-H

1445,340,OE1,344,HE22,340-OE1-344-HE22

1446,340,OE1,344,HE21,340-OE1-344-HE21

1447,340,O,344,H,340-O-344-H

1448,340,O,343,H,340-O-343-H

1449,341,O,344,H,341-O-344-H

1450,341,O,341,HH11,341-O-341-HH11

1451,341,O,345,H,341-O-345-H

1452,342,O,346,H,342-O-346-H

1453,342,O,342,HZ3,342-O-342-HZ3

1454,342,O,342,HZ2,342-O-342-HZ2

1455,342,O,345,H,342-O-345-H

1456,343,O,346,H,343-O-346-H

1457,343,O,347,H,343-O-347-H

1458,344,O,511,HH22,344-O-511-HH22

1459,344,OE1,341,HH21,344-OE1-341-HH21

1460,344,O,348,H,344-O-348-H

1461,344,OE1,341,HH11,344-OE1-341-HH11

1462,344,OE1,341,HE,344-OE1-341-HE

1463,344,O,511,HH12,344-O-511-HH12

1464,344,OE1,341,HH12,344-OE1-341-HH12

1465,345,OE2,341,HE,345-OE2-341-HE

1466,345,OE2,341,HH22,345-OE2-341-HH22

1467,345,O,349,H,345-O-349-H

1468,345,OE1,341,HH22,345-OE1-341-HH22

1469,345,OE1,342,HZ3,345-OE1-342-HZ3

1470,345,OE2,341,HH12,345-OE2-341-HH12

1471,345,OE2,342,HZ2,345-OE2-342-HZ2

1472,345,OE1,342,HZ1,345-OE1-342-HZ1

1473,345,OE1,342,HZ2,345-OE1-342-HZ2

1474,345,OE2,342,HZ1,345-OE2-342-HZ1

1475,345,OE2,341,HH21,345-OE2-341-HH21

1476,345,O,349,HG1,345-O-349-HG1

1477,345,OE1,341,HH21,345-OE1-341-HH21

1478,345,OE2,342,HZ3,345-OE2-342-HZ3

1479,345,OE1,341,HE,345-OE1-341-HE

1480,345,OE1,341,HH12,345-OE1-341-HH12

1481,346,O,350,H,346-O-350-H

1482,346,O,349,H,346-O-349-H

1483,346,OE2,342,HZ1,346-OE2-342-HZ1

1484,346,OE2,342,HZ3,346-OE2-342-HZ3

1485,346,OE1,342,HZ3,346-OE1-342-HZ3

1486,346,OE1,342,HZ1,346-OE1-342-HZ1

1487,346,OE2,342,HZ2,346-OE2-342-HZ2

1488,346,OE1,342,HZ2,346-OE1-342-HZ2

1489,346,OE1,367,H,346-OE1-367-H

1490,347,O,351,H,347-O-351-H

1491,347,O,352,H,347-O-352-H

1492,347,O,350,H,347-O-350-H

1493,348,OD1,353,HE,348-OD1-353-HE

1494,348,OD2,353,HH21,348-OD2-353-HH21

1495,348,OD2,511,HH12,348-OD2-511-HH12

1496,348,OD1,353,HH21,348-OD1-353-HH21

1497,348,OD2,353,HE,348-OD2-353-HE

1498,348,OD2,511,HH22,348-OD2-511-HH22

1499,348,OD1,511,HH12,348-OD1-511-HH12

1500,348,OD1,476,HE22,348-OD1-476-HE22

1501,348,OD1,511,HH22,348-OD1-511-HH22

1502,350,O,355,HH21,350-O-355-HH21

1503,351,O,354,HG,351-O-354-HG

1504,352,O,476,HE21,352-O-476-HE21

1505,352,O,476,HE22,352-O-476-HE22

1506,353,O,477,HH22,353-O-477-HH22

1507,353,O,477,HH12,353-O-477-HH12

1508,354,O,354,HG,354-O-354-HG

1509,355,O,476,HE21,355-O-476-HE21

1510,355,O,476,HE22,355-O-476-HE22

1511,356,O,355,HH11,356-O-355-HH11

1512,357,O,356,HH11,357-O-356-HH11

1513,358,O,361,H,358-O-361-H

1514,359,O,465,HE1,359-O-465-HE1

1515,359,O,362,HH11,359-O-362-HH11

1516,359,O,362,H,359-O-362-H

1517,360,O,363,H,360-O-363-H

1518,360,OG,358,HE,360-OG-358-HE

1519,360,O,363,HG,360-O-363-HG

1520,361,O,364,H,361-O-364-H

1521,361,OD1,358,HE,361-OD1-358-HE

1522,361,OD2,358,H,361-OD2-358-H

1523,361,OD1,355,HH22,361-OD1-355-HH22

1524,361,O,365,H,361-O-365-H

1525,361,OD1,360,HG,361-OD1-360-HG

1526,361,OD1,358,HH21,361-OD1-358-HH21

1527,361,OD1,355,HH12,361-OD1-355-HH12

1528,361,OD2,355,HH22,361-OD2-355-HH22

1529,361,OD1,364,HE2,361-OD1-364-HE2

1530,361,OD2,358,HE,361-OD2-358-HE

1531,361,OD2,355,HH12,361-OD2-355-HH12

1532,362,NH1,465,HE1,362-NH1-465-HE1

1533,362,O,365,H,362-O-365-H

1534,364,NE2,363,HG,364-NE2-363-HG

1535,364,ND1,363,HG,364-ND1-363-HG

1536,365,O,368,H,365-O-368-H

1537,365,O,369,H,365-O-369-H

1538,366,O,370,H,366-O-370-H

1539,366,O,369,H,366-O-369-H

1540,367,O,371,H,367-O-371-H

1541,367,O,370,H,367-O-370-H

1542,368,O,372,H,368-O-372-H

1543,368,O,371,H,368-O-371-H

1544,369,OE1,362,HE,369-OE1-362-HE

1545,369,OE1,464,HH21,369-OE1-464-HH21

1546,369,OE2,362,HH12,369-OE2-362-HH12

1547,369,O,372,H,369-O-372-H

1548,369,OE1,464,HE,369-OE1-464-HE

1549,369,OE1,362,HH21,369-OE1-362-HH21

1550,369,OE2,362,HE,369-OE2-362-HE

1551,369,OE2,464,HE,369-OE2-464-HE

1552,369,OE2,464,HH21,369-OE2-464-HH21

1553,369,OE2,362,HH22,369-OE2-362-HH22

1554,369,O,373,H,369-O-373-H

1555,369,OE2,362,HH21,369-OE2-362-HH21

1556,369,OE1,362,HH12,369-OE1-362-HH12

1557,370,O,373,H,370-O-373-H

1558,370,O,374,H,370-O-374-H

1559,371,O,375,H,371-O-375-H

1560,371,O,375,HG1,371-O-375-HG1

1561,372,O,376,H,372-O-376-H

1562,373,O,377,H,373-O-377-H

1563,374,OE1,431,HH22,374-OE1-431-HH22

1564,374,OE2,377,HH21,374-OE2-377-HH21

1565,374,O,378,H,374-O-378-H

1566,374,OE2,431,HH22,374-OE2-431-HH22
1567,374,OE2,431,HH11,374-OE2-431-HH11
1568,374,OE2,377,HE,374-OE2-377-HE
1569,374,OE2,431,HH12,374-OE2-431-HH12
1570,374,OE2,428,H,374-OE2-428-H
1571,374,OE1,431,HH11,374-OE1-431-HH11
1572,374,OE1,428,H,374-OE1-428-H
1573,374,OE1,377,HE,374-OE1-377-HE
1574,374,OE1,431,HH12,374-OE1-431-HH12
1575,374,OE1,377,HH21,374-OE1-377-HH21
1576,375,O,379,H,375-O-379-H
1577,375,O,379,HG,375-O-379-HG
1578,375,OG1,329,HG,375-OG1-329-HG
1579,376,O,380,H,376-O-380-H
1580,377,O,380,HG,377-O-380-HG
1581,377,O,416,HE2,377-O-416-HE2
1582,377,O,380,H,377-O-380-H
1583,378,ND1,332,HH,378-ND1-332-HH
1584,378,O,416,HE2,378-O-416-HE2
1585,379,OG,381,H,379-OG-381-H
1586,379,O,412,HE1,379-O-412-HE1
1587,380,OG,415,HD22,380-OG-415-HD22
1588,380,O,382,H,380-O-382-H
1589,380,O,380,HG,380-O-380-HG
1590,380,OG,416,HE2,380-OG-416-HE2
1591,381,O,498,H,381-O-498-H
1592,382,O,411,HE21,382-O-411-HE21
1593,383,O,412,H,383-O-412-H
1594,383,O,411,H,383-O-411-H
1595,384,O,230,HG,384-O-230-HG
1596,385,O,230,HG,385-O-230-HG
1597,386,O,385,HG1,386-O-385-HG1
1598,386,O,106,HH11,386-O-106-HH11
1599,386,O,106,HH12,386-O-106-HH12
1600,387,O,106,HH11,387-O-106-HH11
1601,388,O,407,H,388-O-407-H
1602,388,NE2,385,HG1,388-NE2-385-HG1
1603,388,O,389,HG,388-O-389-HG
1604,389,O,105,H,389-O-105-H
1605,389,O,391,H,389-O-391-H
1606,389,O,391,HG1,389-O-391-HG1
1607,390,OG1,404,H,390-OG1-404-H
1608,390,O,389,HG,390-O-389-HG
1609,390,O,404,H,390-O-404-H
1610,390,OG1,405,H,390-OG1-405-H
1611,391,OG1,104,HZ1,391-OG1-104-HZ1
1612,391,O,403,HZ3,391-O-403-HZ3
1613,391,OG1,104,HZ2,391-OG1-104-HZ2
1614,391,OG1,104,H,391-OG1-104-H
1615,391,O,403,HZ2,391-O-403-HZ2
1616,391,OG1,104,HZ3,391-OG1-104-HZ3
1617,391,OG1,105,H,391-OG1-105-H
1618,391,O,403,HZ1,391-O-403-HZ1
1619,393,OD2,393,H,393-OD2-393-H
1620,393,OD1,400,HH,393-OD1-400-HH
1621,393,OD1,392,HH21,393-OD1-392-HH21
1622,393,OD2,403,HZ3,393-OD2-403-HZ3
1623,393,OD2,400,HH,393-OD2-400-HH
1624,393,OD2,403,H,393-OD2-403-H
1625,393,OD1,403,HZ1,393-OD1-403-HZ1
1626,393,OD1,403,HZ3,393-OD1-403-HZ3
1627,393,O,392,HE,393-O-392-HE
1628,393,O,392,HH21,393-O-392-HH21
1629,393,OD1,403,H,393-OD1-403-H
1630,393,OD2,403,HZ1,393-OD2-403-HZ1
1631,393,OD1,393,H,393-OD1-393-H
1632,393,OD1,403,HZ2,393-OD1-403-HZ2
1633,393,OD2,392,HH21,393-OD2-392-HH21
1634,393,OD2,403,HZ2,393-OD2-403-HZ2
1635,394,OG1,99,HE22,394-OG1-99-HE22
1636,394,O,395,HG,394-O-395-HG
1637,394,OG1,395,H,394-OG1-395-H
1638,394,OG1,99,HE21,394-OG1-99-HE21
1639,394,O,401,H,394-O-401-H
1640,395,O,99,HE21,395-O-99-HE21
1641,395,O,99,HE22,395-O-99-HE22
1642,396,O,398,H,396-O-398-H
1643,396,O,399,H,396-O-399-H
1644,397,O,38,HZ1,397-O-38-HZ1
1645,397,O,38,HZ2,397-O-38-HZ2

1646,397,O,38,HZ3,397-O-38-HZ3

1647,397,O,399,H,397-O-399-H

1648,398,O,38,H,398-O-38-H

1649,398,O,38,HZ3,398-O-38-HZ3

1650,398,O,38,HZ1,398-O-38-HZ1

1651,398,O,38,HZ2,398-O-38-HZ2

1652,399,O,396,H,399-O-396-H

1653,399,O,395,HG,399-O-395-HG

1654,400,O,39,HD22,400-O-39-HD22

1655,401,O,394,H,401-O-394-H

1656,402,O,405,H,402-O-405-H

1657,402,O,390,HG1,402-O-390-HG1

1658,403,O,405,HH11,403-O-405-HH11

1659,404,O,389,HG,404-O-389-HG

1660,404,O,405,HH11,404-O-405-HH11

1661,405,O,389,HG,405-O-389-HG

1662,405,O,390,H,405-O-390-H

1663,405,O,390,HG1,405-O-390-HG1

1664,406,O,84,H,406-O-84-H

1665,407,O,388,H,407-O-388-H

1666,408,O,86,H,408-O-86-H

1667,409,O,411,H,409-O-411-H

1668,409,O,385,H,409-O-385-H

1669,410,OD1,413,H,410-OD1-413-H

1670,410,OD1,412,H,410-OD1-412-H

1671,410,OD1,411,H,410-OD1-411-H

1672,410,O,413,H,410-O-413-H

1673,411,OE1,451,H,411-OE1-451-H

1674,411,O,415,HD22,411-O-415-HD22

1675,411,O,414,H,411-O-414-H

1676,411,O,415,H,411-O-415-H

1677,412,O,415,H,412-O-415-H

1678,412,O,416,H,412-O-416-H

1679,413,OE1,87,HG,413-OE1-87-HG

1680,413,O,417,H,413-O-417-H

1681,413,OE1,88,H,413-OE1-88-H

1682,413,NE2,68,HE22,413-NE2-68-HE22

1683,413,OE1,64,HG,413-OE1-64-HG

1684,413,OE1,68,HE21,413-OE1-68-HE21

1685,413,OE1,67,HG,413-OE1-67-HG

1686,413,OE1,410,HD22,413-OE1-410-HD22

1687,413,O,413,HE21,413-O-413-HE21

1688,413,NE2,68,HE21,413-NE2-68-HE21

1689,413,OE1,68,HE22,413-OE1-68-HE22

1690,413,O,416,H,413-O-416-H

1691,414,O,421,HE1,414-O-421-HE1

1692,414,O,417,H,414-O-417-H

1693,415,OD1,448,H,415-OD1-448-H

1694,415,O,377,HH11,415-O-377-HH11

1695,416,O,418,HE21,416-O-418-HE21

1696,416,ND1,377,HH12,416-ND1-377-HH12

1697,417,OD1,68,HE22,417-OD1-68-HE22

1698,417,OD1,418,HE22,417-OD1-418-HE22

1699,417,OD2,413,HE21,417-OD2-413-HE21

1700,417,OD1,413,HE22,417-OD1-413-HE22

1701,417,OD1,419,HZ1,417-OD1-419-HZ1

1702,417,OD1,413,HE21,417-OD1-413-HE21

1703,417,OD2,413,HE22,417-OD2-413-HE22

1704,417,OD2,68,HE22,417-OD2-68-HE22

1705,417,O,420,H,417-O-420-H

1706,417,OD1,420,H,417-OD1-420-H

1707,417,OD1,419,HZ2,417-OD1-419-HZ2

1708,417,OD2,419,HZ2,417-OD2-419-HZ2

1709,417,OD2,420,H,417-OD2-420-H

1710,417,OD1,419,H,417-OD1-419-H

1711,417,OD1,419,HZ3,417-OD1-419-HZ3

1712,417,OD2,419,HZ1,417-OD2-419-HZ1

1713,417,O,421,H,417-O-421-H

1714,417,OD2,419,H,417-OD2-419-H

1715,417,OD2,419,HZ3,417-OD2-419-HZ3

1716,418,OE1,418,H,418-OE1-418-H

1717,418,OE1,65,HH12,418-OE1-65-HH12

1718,418,OE1,419,HZ2,418-OE1-419-HZ2

1719,418,OE1,65,HH22,418-OE1-65-HH22

1720,418,OE1,413,HE22,418-OE1-413-HE22

1721,418,OE1,68,HE22,418-OE1-68-HE22

1722,418,OE1,423,H,418-OE1-423-H

1723,418,O,422,H,418-O-422-H

1724,418,OE1,68,HE21,418-OE1-68-HE21

1725,418,O,418,HE21,418-O-418-HE21

1726,420,O,446,HZ2,420-O-446-HZ2

1727,420,O,446,HZ1,420-O-446-HZ1

1728,420,O,446,HZ3,420-O-446-HZ3

1729,421,O,431,HE,421-O-431-HE

1730,421,O,431,HH21,421-O-431-HH21

1731,422,O,431,HH21,422-O-431-HH21

1732,422,O,431,HE,422-O-431-HE

1733,423,O,431,HH21,423-O-431-HH21

1734,423,O,426,H,423-O-426-H

1735,423,OD1,418,HE22,423-OD1-418-HE22

1736,423,OD1,426,H,423-OD1-426-H

1737,423,O,423,HD22,423-O-423-HD22

1738,423,O,431,HH22,423-O-431-HH22

1739,424,O,377,HH12,424-O-377-HH12

1740,424,O,377,HH22,424-O-377-HH22

1741,425,O,425,HG,425-O-425-HG

1742,425,O,378,HE2,425-O-378-HE2

1743,426,O,431,HH22,426-O-431-HH22

1744,426,OE2,423,HD21,426-OE2-423-HD21

1745,426,OE1,423,HD22,426-OE1-423-HD22

1746,426,OE1,425,HG,426-OE1-425-HG

1747,426,OE1,427,H,426-OE1-427-H

1748,426,OE2,425,HG,426-OE2-425-HG

1749,426,O,431,HH12,426-O-431-HH12

1750,426,OE2,423,HD22,426-OE2-423-HD22

1751,426,O,431,HH21,426-O-431-HH21

1752,426,OE1,423,HD21,426-OE1-423-HD21

1753,426,OE2,427,H,426-OE2-427-H

1754,427,O,338,HH21,427-O-338-HH21

1755,428,O,431,H,428-O-431-H

1756,428,O,431,HH11,428-O-431-HH11

1757,429,O,432,H,429-O-432-H

1758,429,O,433,H,429-O-433-H

1759,430,OE2,342,HZ1,430-OE2-342-HZ1

1760,430,OE2,430,H,430-OE2-430-H

1761,430,OE1,338,HH12,430-OE1-338-HH12

1762,430,OE1,342,HZ1,430-OE1-342-HZ1

1763,430,OE1,342,HZ2,430-OE1-342-HZ2

1764,430,OE1,342,HZ3,430-OE1-342-HZ3

1765,430,OE2,367,HH,430-OE2-367-HH

1766,430,OE1,367,HH,430-OE1-367-HH

1767,430,OE2,342,HZ2,430-OE2-342-HZ2

1768,430,OE2,342,HZ3,430-OE2-342-HZ3

1769,430,OE1,430,H,430-OE1-430-H

1770,430,OE2,338,HH22,430-OE2-338-HH22

1771,430,OE1,338,HH22,430-OE1-338-HH22

1772,430,O,433,H,430-O-433-H

1773,430,OE2,338,HH12,430-OE2-338-HH12

1774,431,O,433,H,431-O-433-H

1775,432,O,440,H,432-O-440-H

1776,434,O,437,H,434-O-437-H

1777,434,OG1,438,H,434-OG1-438-H

1778,434,OG1,436,H,434-OG1-436-H

1779,434,OG1,437,H,434-OG1-437-H

1780,435,O,437,H,435-O-437-H

1781,436,OD2,436,H,436-OD2-436-H

1782,436,OD1,434,HG1,436-OD1-434-HG1

1783,436,OD1,438,H,436-OD1-438-H

1784,436,OD1,436,H,436-OD1-436-H

1785,436,O,438,H,436-O-438-H

1786,436,OD2,434,HG1,436-OD2-434-HG1

1787,436,OD2,438,H,436-OD2-438-H

1788,438,O,434,HG1,438-O-434-HG1

1789,438,O,434,H,438-O-434-H

1790,439,O,441,HZ3,439-O-441-HZ3

1791,439,O,441,HZ2,439-O-441-HZ2

1792,439,O,441,HZ1,439-O-441-HZ1

1793,439,O,441,H,439-O-441-H

1794,440,O,444,H,440-O-444-H

1795,440,O,444,HG,440-O-444-HG

1796,440,OD1,443,H,440-OD1-443-H

1797,440,O,443,H,440-O-443-H

1798,440,OD2,443,H,440-OD2-443-H

1799,440,OD1,442,H,440-OD1-442-H

1800,440,OD2,442,H,440-OD2-442-H

1801,440,OD1,444,HG,440-OD1-444-HG

1802,440,O,442,H,440-O-442-H

1803,440,OD1,444,H,440-OD1-444-H

1804,441,O,445,H,441-O-445-H

1805,441,O,444,HG,441-O-444-HG

1806,442,O,446,H,442-O-446-H

1807,442,O,446,HZ2,442-O-446-HZ2

1808,442,O,446,HZ3,442-O-446-HZ3

1809,442,O,445,H,442-O-445-H

1810,442,O,446,HZ1,442-O-446-HZ1

1811,443,O,447,H,443-O-447-H

1812,443,O,446,H,443-O-446-H

1813,444,OG,445,H,444-OG-445-H

1814,444,O,447,H,444-O-447-H

1815,445,OE2,446,HZ2,445-OE2-446-HZ2

1816,445,OE2,93,HH12,445-OE2-93-HH12

1817,445,OE2,93,HH21,445-OE2-93-HH21

1818,445,OE1,93,HH21,445-OE1-93-HH21

1819,445,OE1,464,HH22,445-OE1-464-HH22

1820,445,OE2,464,HH12,445-OE2-464-HH12

1821,445,OE2,93,HH22,445-OE2-93-HH22

1822,445,OE2,446,HZ3,445-OE2-446-HZ3

1823,445,OE1,464,HH12,445-OE1-464-HH12

1824,445,OE2,464,HH22,445-OE2-464-HH22

1825,445,OE1,93,HE,445-OE1-93-HE

1826,445,OE1,446,HZ3,445-OE1-446-HZ3

1827,445,OE1,444,HG,445-OE1-444-HG

1828,445,OE2,446,HZ1,445-OE2-446-HZ1

1829,445,OE2,444,HG,445-OE2-444-HG

1830,445,OE1,446,HZ2,445-OE1-446-HZ2

1831,445,OE1,446,HZ1,445-OE1-446-HZ1

1832,445,OE1,441,HZ1,445-OE1-441-HZ1

1833,445,O,93,HH22,445-O-93-HH22

1834,445,OE2,456,HZ2,445-OE2-456-HZ2

1835,445,OE1,93,HH22,445-OE1-93-HH22

1836,445,O,93,HH21,445-O-93-HH21

1837,445,OE1,93,HH12,445-OE1-93-HH12

1838,446,O,448,H,446-O-448-H

1839,448,O,415,HD21,448-O-415-HD21

1840,449,O,411,HE22,449-O-411-HE22

1841,450,O,457,H,450-O-457-H

1842,451,O,456,HZ1,451-O-456-HZ1

1843,451,O,456,HZ3,451-O-456-HZ3

1844,452,O,456,H,452-O-456-H

1845,452,O,455,H,452-O-455-H

1846,453,O,456,HZ1,453-O-456-HZ1

1847,453,O,456,HZ2,453-O-456-HZ2

1848,453,O,456,H,453-O-456-H

1849,453,O,455,H,453-O-455-H

1850,453,O,456,HZ3,453-O-456-HZ3

1851,454,O,456,H,454-O-456-H

1852,454,O,135,HH12,454-O-135-HH12

1853,454,O,135,HH22,454-O-135-HH22

1854,454,O,135,HH21,454-O-135-HH21

1855,455,O,452,H,455-O-452-H

1856,455,O,135,HH12,455-O-135-HH12

1857,456,O,135,HH12,456-O-135-HH12

1858,456,O,135,HH22,456-O-135-HH22

1859,456,O,135,HH21,456-O-135-HH21

1860,457,O,456,HZ2,457-O-456-HZ2

1861,457,O,456,HZ1,457-O-456-HZ1

1862,457,O,460,H,457-O-460-H

1863,457,O,456,HZ3,457-O-456-HZ3

1864,458,O,461,H,458-O-461-H

1865,458,O,461,HG1,458-O-461-HG1

1866,458,O,139,HE21,458-O-139-HE21

1867,458,O,139,HE22,458-O-139-HE22

1868,459,O,463,H,459-O-463-H

1869,459,O,462,H,459-O-462-H

1870,460,OE2,456,HZ3,460-OE2-456-HZ3

1871,460,OE2,464,HH21,460-OE2-464-HH21

1872,460,O,464,HE,460-O-464-HE

1873,460,OE1,461,HG1,460-OE1-461-HG1

1874,460,O,464,HH11,460-O-464-HH11

1875,460,OE2,464,HH12,460-OE2-464-HH12

1876,460,OE2,461,HG1,460-OE2-461-HG1

1877,460,OE1,464,HH11,460-OE1-464-HH11

1878,460,OE1,456,HZ2,460-OE1-456-HZ2

1879,460,OE1,456,HZ3,460-OE1-456-HZ3

1880,460,OE2,464,HH22,460-OE2-464-HH22

1881,460,OE2,456,HZ2,460-OE2-456-HZ2

1882,460,OE1,143,HZ1,460-OE1-143-HZ1

1883,460,OE1,143,HZ2,460-OE1-143-HZ2

1884,460,OE1,464,HH22,460-OE1-464-HH22

1885,460,OE1,461,H,460-OE1-461-H

1886,460,OE2,456,HZ1,460-OE2-456-HZ1

1887,460,OE1,464,HH12,460-OE1-464-HH12

1888,460,OE1,456,HZ1,460-OE1-456-HZ1

1889,460,O,464,H,460-O-464-H

1890,460,OE2,461,H,460-OE2-461-H

1891,460,OE2,143,HZ1,460-OE2-143-HZ1

1892,460,OE2,464,HH11,460-OE2-464-HH11

1893,461,O,465,H,461-O-465-H

1894,461,O,464,H,461-O-464-H

1895,462,O,466,H,462-O-466-H

1896,462,O,464,H,462-O-464-H

1897,462,O,465,H,462-O-465-H

1898,463,O,467,H,463-O-467-H

1899,464,O,362,HH21,464-O-362-HH21

1900,464,O,362,HH12,464-O-362-HH12

1901,464,O,362,HH22,464-O-362-HH22

1902,464,O,468,H,464-O-468-H

1903,465,O,468,H,465-O-468-H

1904,465,O,469,H,465-O-469-H

1905,466,O,470,H,466-O-470-H

1906,466,O,469,H,466-O-469-H

1907,467,O,471,H,467-O-471-H

1908,468,O,472,H,468-O-472-H

1909,468,O,471,H,468-O-471-H

1910,469,O,473,H,469-O-473-H

1911,470,O,474,H,470-O-474-H

1912,471,O,475,H,471-O-475-H

1913,472,O,476,H,472-O-476-H

1914,473,O,477,H,473-O-477-H

1915,473,O,476,H,473-O-476-H

1916,473,O,477,HH21,473-O-477-HH21

1917,473,O,477,HE,473-O-477-HE

1918,474,O,477,H,474-O-477-H

1919,474,O,478,H,474-O-478-H

1920,475,O,511,HH11,475-O-511-HH11

1921,475,O,344,HE21,475-O-344-HE21

1922,475,O,511,HE,475-O-511-HE

1923,475,O,511,HH21,475-O-511-HH21

1924,476,O,353,HE,476-O-353-HE

1925,476,O,511,HE,476-O-511-HE

1926,476,OE1,477,HH21,476-OE1-477-HH21

1927,476,O,511,HH21,476-O-511-HH21

1928,477,O,511,H,477-O-511-H

1929,478,O,344,HE22,478-O-344-HE22

1930,478,O,344,HE21,478-O-344-HE21

1931,479,OE1,512,HG,479-OE1-512-HG

1932,479,OE1,512,H,479-OE1-512-H

1933,479,OE1,341,HH22,479-OE1-341-HH22

1934,479,O,509,H,479-O-509-H

1935,479,OE2,512,HG,479-OE2-512-HG

1936,479,OE2,341,HH12,479-OE2-341-HH12

1937,479,OE1,344,HE22,479-OE1-344-HE22

1938,479,OE1,341,HH12,479-OE1-341-HH12

1939,479,OE2,341,HH22,479-OE2-341-HH22

1940,479,OE1,509,HE21,479-OE1-509-HE21

1941,479,OE2,341,HH11,479-OE2-341-HH11

1942,479,O,509,HE21,479-O-509-HE21

1943,479,OE2,509,HE21,479-OE2-509-HE21

1944,479,OE2,341,HE,479-OE2-341-HE

1945,479,OE1,341,HH11,479-OE1-341-HH11

1946,479,OE1,341,HE,479-OE1-341-HE

1947,479,OE2,341,HH21,479-OE2-341-HH21

1948,479,OE1,341,HH21,479-OE1-341-HH21

1949,479,OE2,512,H,479-OE2-512-H

1950,479,OE2,344,HE22,479-OE2-344-HE22

1951,480,O,340,HE22,480-O-340-HE22

1952,480,O,481,HG,480-O-481-HG

1953,481,O,507,H,481-O-507-H

1954,481,OG,507,HE21,481-OG-507-HE21

1955,481,OG,509,HE22,481-OG-509-HE22

1956,481,OG,482,H,481-OG-482-H

1957,482,O,481,HG,482-O-481-HG

1958,483,O,485,H,483-O-485-H

1959,483,O,486,H,483-O-486-H

1960,484,O,487,HZ1,484-O-487-HZ1

1961,484,O,486,H,484-O-486-H

1962,484,O,487,HZ3,484-O-487-HZ3

1963,485,O,487,HZ2,485-O-487-HZ2

1964,485,O,487,HZ1,485-O-487-HZ1

1965,485,O,487,HZ3,485-O-487-HZ3

1966,486,O,505,HE2,486-O-505-HE2

1967,486,O,488,H,486-O-488-H

1968,487,O,489,H,487-O-489-H

1969,487,O,505,HE2,487-O-505-HE2

1970,488,O,487,HZ3,488-O-487-HZ3

1971,488,O,487,HZ1,488-O-487-HZ1

1972,488,O,487,HZ2,488-O-487-HZ2

1973,489,OD2,487,HZ2,489-OD2-487-HZ2

1974,489,OD2,490,H,489-OD2-490-H

1975,489,OD1,487,HZ1,489-OD1-487-HZ1

1976,489,OD2,487,HZ1,489-OD2-487-HZ1

1977,489,OD1,487,HZ3,489-OD1-487-HZ3

1978,489,OD1,490,H,489-OD1-490-H

1979,489,O,501,H,489-O-501-H

1980,489,O,491,H,489-O-491-H

1981,489,OD2,491,H,489-OD2-491-H

1982,489,OD1,487,HZ2,489-OD1-487-HZ2

1983,489,O,500,HD1,489-O-500-HD1

1984,489,OD2,491,HG1,489-OD2-491-HG1

1985,489,OD1,491,HG1,489-OD1-491-HG1

1986,489,OD1,491,H,489-OD1-491-H

1987,490,O,501,H,490-O-501-H

1988,491,O,501,H,491-O-501-H

1989,492,O,58,HD21,492-O-58-HD21

1990,493,O,499,H,493-O-499-H

1991,494,OH,230,HG,494-OH-230-HG

1992,494,O,57,HZ2,494-O-57-HZ2

1993,494,OH,230,H,494-OH-230-H

1994,494,O,57,HZ3,494-O-57-HZ3

1995,497,OG1,499,HZ1,497-OG1-499-HZ1

1996,497,OG1,499,HZ2,497-OG1-499-HZ2

1997,497,O,499,HZ3,497-O-499-HZ3

1998,497,O,499,HZ1,497-O-499-HZ1

1999,497,OG1,499,HZ3,497-OG1-499-HZ3

2000,497,O,495,H,497-O-495-H

2001,497,O,499,HZ2,497-O-499-HZ2

2002,497,O,497,HG1,497-O-497-HG1

2003,498,O,328,HE1,498-O-328-HE1

2004,499,O,493,H,499-O-493-H

2005,500,ND1,501,H,500-ND1-501-H

2006,500,NE2,327,HG,500-NE2-327-HG

2007,500,O,502,H,500-O-502-H

2008,500,O,500,HD1,500-O-500-HD1

2009,501,O,503,H,501-O-503-H

2010,501,O,500,HD1,501-O-500-HD1

2011,502,O,487,HZ1,502-O-487-HZ1

2012,502,O,487,HZ3,502-O-487-HZ3

2013,503,O,505,H,503-O-505-H

2014,503,O,185,HD22,503-O-185-HD22

2015,504,OE1,185,HD22,504-OE1-185-HD22

2016,504,OE1,188,HE,504-OE1-188-HE

2017,504,OE2,185,H,504-OE2-185-H

2018,504,OE1,188,HH21,504-OE1-188-HH21

2019,504,OE2,504,H,504-OE2-504-H

2020,504,OE1,504,H,504-OE1-504-H

2021,504,OE2,188,HH21,504-OE2-188-HH21

2022,504,OE2,188,HH11,504-OE2-188-HH11

2023,504,OE2,188,HE,504-OE2-188-HE

2024,504,OE1,188,HH11,504-OE1-188-HH11

2025,504,OE2,503,H,504-OE2-503-H

2026,504,OE1,183,HE2,504-OE1-183-HE2

2027,504,OE2,188,HH12,504-OE2-188-HH12

2028,504,OE2,185,HD22,504-OE2-185-HD22

2029,504,OE2,188,HH22,504-OE2-188-HH22

2030,504,OE1,188,HH12,504-OE1-188-HH12

2031,504,OE1,185,H,504-OE1-185-H

2032,504,OE1,188,HH22,504-OE1-188-HH22

2033,505,ND1,504,H,505-ND1-504-H

2034,505,O,507,HE21,505-O-507-HE21

2035,506,O,184,H,506-O-184-H

2036,507,NE2,183,HE2,507-NE2-183-HE2

2037,507,OE1,509,HE22,507-OE1-509-HE22

2038,507,O,481,H,507-O-481-H

2039,507,OE1,481,HG,507-OE1-481-HG

2040,507,OE1,183,HE2,507-OE1-183-HE2

2041,507,OE1,509,HE21,507-OE1-509-HE21

2042,508,O,509,HE21,508-O-509-HE21

2043,509,OE1,507,HE22,509-OE1-507-HE22

2044,509,OE1,481,HG,509-OE1-481-HG

2045,509,O,479,H,509-O-479-H

2046,509,OE1,507,HE21,509-OE1-507-HE21

2047,509,OE1,510,H,509-OE1-510-H

2048,509,O,511,H,509-O-511-H

2049,510,O,509,HE21,510-O-509-HE21

2050,510,O,512,HG,510-O-512-HG

2051,511,O,353,HH12,511-O-353-HH12

2052,511,O,353,HH22,511-O-353-HH22

2053,511,O,353,HH11,511-O-353-HH11

2054,511,O,512,HG,511-O-512-HG

2055,512,O,512,HG,512-O-512-HG

2056,512,O,353,HH11,512-O-353-HH11

2057,512,O,353,HH12,512-O-353-HH12

2058,512,O,511,HH11,512-O-511-HH11

2059,512,OXT,512,HG,512-OXT-512-HG

2060,512,OXT,353,HH22,512-OXT-353-HH22

2061,512,OXT,353,HH11,512-OXT-353-HH11

2062,512,OXT,353,HH12,512-OXT-353-HH12

2063,512,O,353,HH22,512-O-353-HH22

2064,512,O,341,HH12,512-O-341-HH12

2065,515,O1A,106,HH22,515-O1A-106-HH22

2066,515,O1D,135,HE,515-O1D-135-HE

2067,515,O2A,386,H,515-O2A-386-H

2068,515,O2D,455,HH11,515-O2D-455-HH11

2069,515,O1A,386,H,515-O1A-386-H

2070,515,O2D,106,HH21,515-O2D-106-HH21

2071,515,O2A,106,HH22,515-O2A-106-HH22

2072,515,O2A,106,HH12,515-O2A-106-HH12

2073,515,O1D,455,HH11,515-O1D-455-HH11

2074,515,O2A,455,HH11,515-O2A-455-HH11

2075,515,O2D,135,HH21,515-O2D-135-HH21

2076,515,O1D,455,HH12,515-O1D-455-HH12

2077,515,O1A,106,HH12,515-O1A-106-HH12

2078,515,O2D,135,HH11,515-O2D-135-HH11

2079,515,O1D,131,HE1,515-O1D-131-HE1

2080,515,O1D,106,HH21,515-O1D-106-HH21

2081,515,O2D,135,HE,515-O2D-135-HE

2082,515,O1D,135,HH21,515-O1D-135-HH21

2083,515,O1D,135,HH11,515-O1D-135-HH11

2084,516,O15,222,HD22,516-O15-222-HD22

2085,516,O18,122,HG,516-O18-122-HG

2086,516,O15,255,HD22,516-O15-255-HD22

Model III

RES_DONOR,ATOM_DONOR,RES_ACCEPTOR,ATOM_ACCEPTOR,
TAG

1,37,O,39,H,37-O-39-H

2,37,O,39,HD22,37-O-39-HD22

3,38,O,400,H,38-O-400-H

4,39,O,38,HZ1,39-O-38-HZ1

5,39,O,39,HD22,39-O-39-HD22

6,39,O,38,HZ2,39-O-38-HZ2

7,39,OD1,39,H,39-OD1-39-H

8,39,ND2,75,HE22,39-ND2-75-HE22

9,39,N,39,HD22,39-N-39-HD22

10,39,OD1,405,HH21,39-OD1-405-HH21

11,39,OD1,405,HH22,39-OD1-405-HH22

12,39,OD1,75,HE22,39-OD1-75-HE22

13,40,O,75,HE21,40-O-75-HE21

14,40,O,39,HD22,40-O-39-HD22

15,41,O,75,H,41-O-75-H

16,43,O,51,HE2,43-O-51-HE2

17,44,O,51,HE2,44-O-51-HE2

18,45,O,77,HH12,45-O-77-HH12

19,45,O,77,HH21,45-O-77-HH21

20,45,O,77,HH11,45-O-77-HH11

21,45,O,77,HE,45-O-77-HE

22,45,O,77,HH22,45-O-77-HH22

23,46,O,77,HE,46-O-77-HE

24,46,O,50,H,46-O-50-H

25,46,O,48,H,46-O-48-H

26,46,O,77,HH21,46-O-77-HH21

27,46,O,77,HH22,46-O-77-HH22

28,46,O,49,H,46-O-49-H

29,46,O,77,HH12,46-O-77-HH12

30,46,O,77,HH11,46-O-77-HH11

31,47,O,80,HG,47-O-80-HG

32,47,O,77,HH21,47-O-77-HH21

33,47,O,77,HH11,47-O-77-HH11

34,47,O,77,HH12,47-O-77-HH12

35,47,O,77,HE,47-O-77-HE

36,47,O,77,HH22,47-O-77-HH22

37,48,O,77,HH21,48-O-77-HH21

38,48,O,80,HG,48-O-80-HG

39,48,O,77,HH12,48-O-77-HH12

40,49,O,79,H,49-O-79-H

41,49,O,52,H,49-O-52-H

42,50,O,52,H,50-O-52-H

43,50,O,46,H,50-O-46-H

44,51,O,55,H,51-O-55-H

45,51,O,54,H,51-O-54-H

46,51,O,54,HG1,51-O-54-HG1

47,51,O,53,H,51-O-53-H

48,51,ND1,51,H,51-ND1-51-H

49,51,ND1,77,H,51-ND1-77-H

50,52,O,55,H,52-O-55-H

51,52,O,56,H,52-O-56-H

52,53,O,56,H,53-O-56-H

53,54,OG1,46,HE1,54-OG1-46-HE1

54,54,O,54,HG1,54-O-54-HG1

55,54,O,56,H,54-O-56-H

56,54,O,65,HH22,54-O-65-HH22

57,55,O,58,H,55-O-58-H

58,55,O,58,HD22,55-O-58-HD22

59,57,O,57,HZ2,57-O-57-HZ2

60,57,O,57,HZ3,57-O-57-HZ3

61,57,O,57,HZ1,57-O-57-HZ1

62,57,O,58,HD22,57-O-58-HD22

63,58,O,58,HD22,58-O-58-HD22

64,58,O,61,H,58-O-61-H

65,58,OD1,57,HZ2,58-OD1-57-HZ2

66,58,OD1,65,HH22,58-OD1-65-HH22

67,58,O,62,H,58-O-62-H

68,59,O,63,H,59-O-63-H

69,60,O,410,HD21,60-O-410-HD21

70,60,ND1,60,H,60-ND1-60-H

71,60,O,64,H,60-O-64-H

72,60,O,64,HG,60-O-64-HG

73,61,O,64,HG,61-O-64-HG

74,61,O,64,H,61-O-64-H

75,61,O,65,H,61-O-65-H

76,62,O,66,H,62-O-66-H

77,62,O,65,H,62-O-65-H

78,63,O,410,HD21,63-O-410-HD21

79,63,O,67,H,63-O-67-H

80,63,O,410,HD22,63-O-410-HD22

81,63,O,67,HG,63-O-67-HG

82,64,OG,68,HE21,64-OG-68-HE21

83,64,O,67,HG,64-O-67-HG

84,64,OG,410,HD21,64-OG-410-HD21

85,64,O,67,H,64-O-67-H

86,64,OG,68,HE22,64-OG-68-HE22

87,64,O,68,HE21,64-O-68-HE21

88,64,O,413,HE22,64-O-413-HE22

89,64,OG,65,HH21,64-OG-65-HH21

90,64,O,68,H,64-O-68-H

91,64,OG,413,HE22,64-OG-413-HE22

92,65,O,69,H,65-O-69-H

93,65,O,69,HE21,65-O-69-HE21

94,65,O,68,H,65-O-68-H

95,65,NH1,68,HE22,65-NH1-68-HE22

96,65,O,65,HH11,65-O-65-HH11

97,65,O,65,HE,65-O-65-HE

98,65,O,68,HE21,65-O-68-HE21

99,65,O,69,HE22,65-O-69-HE22

100,66,O,69,H,66-O-69-H

101,66,O,70,H,66-O-70-H

102,67,OG,413,HE21,67-OG-413-HE21

103,67,O,70,H,67-O-70-H

104,67,OG,87,HG,67-OG-87-HG

105,67,O,71,H,67-O-71-H

106,67,OG,413,HE22,67-OG-413-HE22

107,67,O,69,H,67-O-69-H

108,67,OG,410,HD22,67-OG-410-HD22

109,67,OG,68,HE21,67-OG-68-HE21

110,68,O,69,HE21,68-O-69-HE21

111,68,O,68,HE21,68-O-68-HE21

112,68,OE1,65,HH22,68-OE1-65-HH22

113,68,OE1,65,HH21,68-OE1-65-HH21

114,68,OE1,413,HE22,68-OE1-413-HE22

115,68,OE1,418,HE22,68-OE1-418-HE22

116,68,OE1,69,HE21,68-OE1-69-HE21

117,68,OE1,419,HZ2,68-OE1-419-HZ2

118,68,NE2,413,HE22,68-NE2-413-HE22

119,68,OE1,419,HZ1,68-OE1-419-HZ1

120,68,OE1,65,HH11,68-OE1-65-HH11

121,68,OE1,65,HE,68-OE1-65-HE

122,68,OE1,419,HZ3,68-OE1-419-HZ3

123,68,OE1,65,HH12,68-OE1-65-HH12

124,69,OE1,65,HH22,69-OE1-65-HH22

125,69,OE1,70,HH,69-OE1-70-HH

126,69,OE1,65,HH21,69-OE1-65-HH21

127,69,OE1,68,HE21,69-OE1-68-HE21

128,69,O,69,HE21,69-O-69-HE21

129,69,OE1,65,HH12,69-OE1-65-HH12

130,70,OH,69,HE21,70-OH-69-HE21

131,71,O,67,HG,71-O-67-HG

132,71,O,87,HG,71-O-87-HG

133,72,OD1,397,HZ3,72-OD1-397-HZ3

134,72,OD1,72,H,72-OD1-72-H

135,72,OD2,397,HZ3,72-OD2-397-HZ3

136,72,OD2,397,HZ1,72-OD2-397-HZ1

137,72,OD2,397,HZ2,72-OD2-397-HZ2

138,72,OD2,72,H,72-OD2-72-H

139,72,O,87,H,72-O-87-H

140,72,O,88,H,72-O-88-H

141,72,OD1,91,HG1,72-OD1-91-HG1

142,72,OD1,397,HZ1,72-OD1-397-HZ1

143,72,OD1,397,HZ2,72-OD1-397-HZ2

144,72,O,87,HG,72-O-87-HG

145,72,OD2,91,HG1,72-OD2-91-HG1

146,74,O,85,H,74-O-85-H

147,75,OE1,405,HH21,75-OE1-405-HH21

148,75,O,75,HE21,75-O-75-HE21

149,75,NE2,39,HD21,75-NE2-39-HD21

150,75,OE1,39,HD22,75-OE1-39-HD22

151,75,OE1,405,HE,75-OE1-405-HE

152,75,OE1,39,HD21,75-OE1-39-HD21

153,76,O,75,HE21,76-O-75-HE21

154,76,O,83,H,76-O-83-H

155,77,O,51,H,77-O-51-H

156,78,O,81,H,78-O-81-H

157,78,O,81,HG1,78-O-81-HG1

158,78,O,80,H,78-O-80-H

159,79,O,77,HH11,79-O-77-HH11

160,79,O,81,H,79-O-81-H

161,79,O,77,HH21,79-O-77-HH21

162,79,O,77,HE,79-O-77-HE

163,80,OG,81,HG1,80-OG-81-HG1

164,80,O,80,HG,80-O-80-HG

165,80,O,77,HH11,80-O-77-HH11

166,80,O,77,HE,80-O-77-HE

167,80,O,77,HH21,80-O-77-HH21

168,80,O,77,HH12,80-O-77-HH12

169,80,OG,77,HH21,80-OG-77-HH21

170,81,O,78,H,81-O-78-H

171,82,O,405,HH11,82-O-405-HH11

172,83,O,76,H,83-O-76-H

173,83,O,75,HE21,83-O-75-HE21

174,84,O,408,H,84-O-408-H

175,85,O,74,H,85-O-74-H

176,85,O,87,HG,85-O-87-HG

177,86,O,410,H,86-O-410-H

178,86,O,87,HG,86-O-87-HG

179,87,OG,410,HD22,87-OG-410-HD22

180,87,O,87,HG,87-O-87-HG

181,87,OG,67,HG,87-OG-67-HG

182,87,O,413,HE21,87-O-413-HE21

183,88,O,92,H,88-O-92-H

184,88,O,91,H,88-O-91-H

185,89,O,92,H,89-O-92-H

186,89,O,93,HH11,89-O-93-HH11

187,89,O,93,HE,89-O-93-HE

188,89,O,93,H,89-O-93-H

189,90,OD1,91,HG1,90-OD1-91-HG1

190,90,OD2,93,HE,90-OD2-93-HE

191,90,OD2,93,HH12,90-OD2-93-HH12

192,90,OD2,93,HH21,90-OD2-93-HH21

193,90,OD2,93,HH22,90-OD2-93-HH22

194,90,OD1,93,HH12,90-OD1-93-HH12

195,90,OD2,397,HZ1,90-OD2-397-HZ1

196,90,OD1,93,HH22,90-OD1-93-HH22

197,90,OD2,93,HH11,90-OD2-93-HH11
198,90,OD2,98,HH22,90-OD2-98-HH22
199,90,OD1,93,HH11,90-OD1-93-HH11
200,90,O,94,HE21,90-O-94-HE21
201,90,OD1,93,HH21,90-OD1-93-HH21
202,90,OD2,397,HZ3,90-OD2-397-HZ3
203,90,OD2,90,H,90-OD2-90-H
204,90,OD1,91,H,90-OD1-91-H
205,90,OD1,397,HZ3,90-OD1-397-HZ3
206,90,OD2,98,HH12,90-OD2-98-HH12
207,90,OD1,94,HE22,90-OD1-94-HE22
208,90,OD1,397,HZ1,90-OD1-397-HZ1
209,90,OD1,90,H,90-OD1-90-H
210,90,OD1,98,HH22,90-OD1-98-HH22
211,90,OD1,93,HE,90-OD1-93-HE
212,90,OD1,98,HH12,90-OD1-98-HH12
213,90,O,98,HH22,90-O-98-HH22
214,90,OD2,91,HG1,90-OD2-91-HG1
215,90,O,94,H,90-O-94-H
216,90,OD2,397,HZ2,90-OD2-397-HZ2
217,90,OD1,397,HZ2,90-OD1-397-HZ2
218,90,OD2,91,H,90-OD2-91-H
219,90,OD2,94,HE21,90-OD2-94-HE21
220,90,OD1,94,HE21,90-OD1-94-HE21
221,91,O,94,H,91-O-94-H
222,91,OG1,397,HZ1,91-OG1-397-HZ1
223,91,OG1,397,HZ3,91-OG1-397-HZ3
224,91,OG1,397,HZ2,91-OG1-397-HZ2
225,91,O,95,H,91-O-95-H
226,92,O,95,H,92-O-95-H
227,92,O,96,H,92-O-96-H
228,93,O,98,H,93-O-98-H
229,93,O,97,H,93-O-97-H
230,94,OE1,98,HH21,94-OE1-98-HH21
231,94,OE1,91,HG1,94-OE1-91-HG1
232,94,O,99,H,94-O-99-H
233,94,OE1,397,H,94-OE1-397-H
234,94,OE1,397,HZ2,94-OE1-397-HZ2
235,94,OE1,397,HZ3,94-OE1-397-HZ3
236,94,NE2,397,H,94-NE2-397-H
237,94,OE1,397,HZ1,94-OE1-397-HZ1
238,94,OE1,98,HH11,94-OE1-98-HH11
239,94,OE1,98,HE,94-OE1-98-HE
240,94,O,99,HE21,94-O-99-HE21
241,95,O,100,H,95-O-100-H
242,95,O,99,H,95-O-99-H
243,96,O,454,H,96-O-454-H
244,96,O,100,H,96-O-100-H
245,97,O,100,H,97-O-100-H
246,98,O,100,H,98-O-100-H
247,98,O,98,HH11,98-O-98-HH11
248,99,OE1,98,HH21,99-OE1-98-HH21
249,99,O,101,H,99-O-101-H
250,99,OE1,94,HE21,99-OE1-94-HE21
251,99,OE1,98,HH11,99-OE1-98-HH11
252,99,O,103,H,99-O-103-H
253,99,OE1,98,HE,99-OE1-98-HE
254,99,O,102,H,99-O-102-H
255,100,O,454,HZ3,100-O-454-HZ3
256,100,O,454,HZ2,100-O-454-HZ2
257,100,O,103,H,100-O-103-H
258,100,O,104,H,100-O-104-H
259,101,OD1,104,HZ3,101-OD1-104-HZ3
260,101,OD1,454,HZ1,101-OD1-454-HZ1
261,101,OD1,392,HH22,101-OD1-392-HH22
262,101,O,104,HZ3,101-O-104-HZ3
263,101,OD2,104,HZ2,101-OD2-104-HZ2
264,101,OD2,392,HH22,101-OD2-392-HH22
265,101,OD2,392,HH12,101-OD2-392-HH12
266,101,OD1,454,HZ2,101-OD1-454-HZ2
267,101,OD2,454,HZ3,101-OD2-454-HZ3
268,101,OD1,454,HZ3,101-OD1-454-HZ3
269,101,OD1,104,HZ1,101-OD1-104-HZ1
270,101,OD2,454,HZ2,101-OD2-454-HZ2
271,101,OD2,104,HZ1,101-OD2-104-HZ1
272,101,O,104,H,101-O-104-H
273,101,OD1,392,HH12,101-OD1-392-HH12
274,101,O,391,HG1,101-O-391-HG1
275,101,OD2,454,HZ1,101-OD2-454-HZ1
276,101,OD1,104,HZ2,101-OD1-104-HZ2

277,101,OD2,104,HZ3,101-OD2-104-HZ3

278,101,O,104,HZ2,101-O-104-HZ2

279,101,O,104,HZ1,101-O-104-HZ1

280,101,O,392,HH12,101-O-392-HH12

281,102,OD1,392,HE,102-OD1-392-HE

282,102,OD2,392,HE,102-OD2-392-HE

283,102,O,104,H,102-O-104-H

284,102,OD2,392,HH11,102-OD2-392-HH11

285,102,OD1,392,HH11,102-OD1-392-HH11

286,102,OD2,394,HG1,102-OD2-394-HG1

287,102,OD1,392,HH21,102-OD1-392-HH21

288,102,OD2,99,HE21,102-OD2-99-HE21

289,102,OD1,99,HE21,102-OD1-99-HE21

290,102,O,391,HG1,102-O-391-HG1

291,102,O,391,H,102-O-391-H

292,102,OD2,392,HH21,102-OD2-392-HH21

293,103,O,388,HD1,103-O-388-HD1

294,103,O,105,H,103-O-105-H

295,103,O,389,H,103-O-389-H

296,104,O,455,HE,104-O-455-HE

297,104,O,455,HH21,104-O-455-HH21

298,104,O,127,HG,104-O-127-HG

299,105,O,455,HH21,105-O-455-HH21

300,105,O,388,HD1,105-O-388-HD1

301,105,O,389,H,105-O-389-H

302,106,NH1,388,HD1,106-NH1-388-HD1

303,107,O,109,H,107-O-109-H

304,108,OD2,108,H,108-OD2-108-H

305,108,OD1,241,HH12,108-OD1-241-HH12

306,108,OD1,108,H,108-OD1-108-H

307,108,O,241,HH12,108-O-241-HH12

308,108,OD2,241,HH22,108-OD2-241-HH22

309,108,OD1,241,HH22,108-OD1-241-HH22

310,108,OD2,241,HH12,108-OD2-241-HH12

311,108,O,241,HH22,108-O-241-HH22

312,109,O,113,HG1,109-O-113-HG1

313,109,O,113,H,109-O-113-H

314,109,O,112,H,109-O-112-H

315,110,O,114,H,110-O-114-H

316,110,O,113,HG1,110-O-113-HG1

317,110,OH,252,HZ2,110-OH-252-HZ2

318,110,OH,252,HZ3,110-OH-252-HZ3

319,110,OH,252,HZ1,110-OH-252-HZ1

320,110,O,113,H,110-O-113-H

321,111,O,115,H,111-O-115-H

322,111,O,114,H,111-O-114-H

323,112,O,115,H,112-O-115-H

324,113,O,115,H,113-O-115-H

325,113,O,118,H,113-O-118-H

326,114,O,117,H,114-O-117-H

327,114,O,118,H,114-O-118-H

328,114,O,255,HD21,114-O-255-HD21

329,114,O,259,HH,114-O-259-HH

330,114,O,255,HD22,114-O-255-HD22

331,115,O,118,H,115-O-118-H

332,116,O,119,H,116-O-119-H

333,116,O,309,HD22,116-O-309-HD22

334,116,OG,309,HD22,116-OG-309-HD22

335,116,OG,259,HH,116-OG-259-HH

336,116,OG,309,HD21,116-OG-309-HD21

337,116,O,116,HG,116-O-116-HG

338,116,O,309,HD21,116-O-309-HD21

339,116,O,119,HE21,116-O-119-HE21

340,117,O,119,HE21,117-O-119-HE21

341,117,OD1,259,HH,117-OD1-259-HH

342,117,OD1,306,HZ1,117-OD1-306-HZ1

343,117,OD1,306,HZ2,117-OD1-306-HZ2

344,117,O,306,HZ1,117-O-306-HZ1

345,117,N,117,HD22,117-N-117-HD22

346,117,OD1,306,HZ3,117-OD1-306-HZ3

347,117,OD1,309,HD22,117-OD1-309-HD22

348,117,ND2,306,HZ1,117-ND2-306-HZ1

349,117,O,306,HZ2,117-O-306-HZ2

350,117,O,306,HZ3,117-O-306-HZ3

351,117,O,119,HE22,117-O-119-HE22

352,117,O,117,HD22,117-O-117-HD22

353,117,O,119,H,117-O-119-H

354,117,OD1,119,HE21,117-OD1-119-HE21

355,118,O,124,HG,118-O-124-HG

356,118,O,119,HE21,118-O-119-HE21

357,118,O,120,H,118-O-120-H

358,119,O,119,HE21,119-O-119-HE21

359,119,OE1,306,HZ1,119-OE1-306-HZ1

360,119,O,134,HH12,119-O-134-HH12

361,119,O,134,HH22,119-O-134-HH22

362,119,OE1,306,HZ3,119-OE1-306-HZ3

363,119,O,309,HD21,119-O-309-HD21

364,119,OE1,117,HD22,119-OE1-117-HD22

365,119,OE1,306,HZ2,119-OE1-306-HZ2

366,119,OE1,309,HD22,119-OE1-309-HD22

367,119,O,134,HH21,119-O-134-HH21

368,119,OE1,309,HD21,119-OE1-309-HD21

369,119,OE1,124,HG,119-OE1-124-HG

370,119,OE1,134,HH22,119-OE1-134-HH22

371,119,OE1,301,HE22,119-OE1-301-HE22

372,119,OE1,134,HH12,119-OE1-134-HH12

373,120,OG,123,H,120-OG-123-H

374,120,OG,122,H,120-OG-122-H

375,120,O,124,HG,120-O-124-HG

376,120,O,131,HE1,120-O-131-HE1

377,120,O,122,H,120-O-122-H

378,120,O,124,H,120-O-124-H

379,120,O,123,H,120-O-123-H

380,121,O,455,HH12,121-O-455-HH12

381,121,N,134,HH12,121-N-134-HH12

382,121,O,131,HE1,121-O-131-HE1

383,122,O,106,HH21,122-O-106-HH21

384,122,O,106,HE,122-O-106-HE

385,122,OG,106,HH21,122-OG-106-HH21

386,122,O,455,HH12,122-O-455-HH12

387,122,O,455,HH11,122-O-455-HH11

388,124,O,126,H,124-O-126-H

389,124,O,455,HH12,124-O-455-HH12

390,124,O,455,HH22,124-O-455-HH22

391,124,OG,119,HE22,124-OG-119-HE22

392,124,OG,126,H,124-OG-126-H

393,125,O,127,HG,125-O-127-HG

394,125,O,127,H,125-O-127-H

395,126,O,124,HG,126-O-124-HG

396,126,O,455,HH22,126-O-455-HH22

397,126,OD1,124,HG,126-OD1-124-HG

398,126,OD2,119,HE22,126-OD2-119-HE22

399,126,O,127,HG,126-O-127-HG

400,126,OD1,301,HE22,126-OD1-301-HE22

401,126,OD2,134,HH11,126-OD2-134-HH11

402,126,OD2,119,HE21,126-OD2-119-HE21

403,126,OD2,128,H,126-OD2-128-H

404,126,OD1,134,HH11,126-OD1-134-HH11

405,126,OD1,119,HE22,126-OD1-119-HE22

406,126,OD2,127,H,126-OD2-127-H

407,126,OD1,128,H,126-OD1-128-H

408,126,OD2,131,H,126-OD2-131-H

409,126,O,128,H,126-O-128-H

410,126,OD1,119,HE21,126-OD1-119-HE21

411,126,O,455,HH21,126-O-455-HH21

412,126,OD2,134,HH12,126-OD2-134-HH12

413,126,OD2,301,HE21,126-OD2-301-HE21

414,126,OD1,134,HH12,126-OD1-134-HH12

415,126,OD1,127,H,126-OD1-127-H

416,126,OD2,306,HZ3,126-OD2-306-HZ3

417,126,OD1,131,H,126-OD1-131-H

418,127,O,104,HZ2,127-O-104-HZ2

419,127,OG,454,HZ3,127-OG-454-HZ3

420,127,OG,454,HZ2,127-OG-454-HZ2

421,127,OG,455,HE,127-OG-455-HE

422,127,O,127,HG,127-O-127-HG

423,127,O,104,HZ1,127-O-104-HZ1

424,127,O,454,HZ2,127-O-454-HZ2

425,127,O,454,HZ1,127-O-454-HZ1

426,127,O,104,HZ3,127-O-104-HZ3

427,127,OG,455,HH21,127-OG-455-HH21

428,127,OG,454,HZ1,127-OG-454-HZ1

429,127,OG,104,HZ1,127-OG-104-HZ1

430,127,O,454,HZ3,127-O-454-HZ3

431,128,O,131,H,128-O-131-H

432,128,O,132,H,128-O-132-H

433,129,O,133,H,129-O-133-H

434,129,O,299,HD21,129-O-299-HD21

435,130,O,134,H,130-O-134-H

436,130,O,133,H,130-O-133-H

437,131,O,134,H,131-O-134-H

438,131,O,135,H,131-O-135-H

439,132,O,136,H,132-O-136-H

440,133,O,136,H,133-O-136-H

441,133,O,137,H,133-O-137-H

442,134,O,138,H,134-O-138-H

443,134,NH2,309,HD21,134-NH2-309-HD21

444,135,O,138,H,135-O-138-H

445,135,O,139,HE21,135-O-139-HE21

446,135,O,139,H,135-O-139-H

447,136,O,140,H,136-O-140-H

448,136,NH2,299,HD21,136-NH2-299-HD21

449,136,O,140,HD22,136-O-140-HD22

450,136,O,139,H,136-O-139-H

451,137,O,140,H,137-O-140-H

452,137,O,141,H,137-O-141-H

453,137,O,140,HD22,137-O-140-HD22

454,138,O,141,H,138-O-141-H

455,138,O,142,H,138-O-142-H

456,139,OE1,136,HH11,139-OE1-136-HH11

457,139,O,139,HE21,139-O-139-HE21

458,139,O,143,H,139-O-143-H

459,139,OE1,135,HH22,139-OE1-135-HH22

460,139,OE1,143,HZ2,139-OE1-143-HZ2

461,139,OE1,461,HG1,139-OE1-461-HG1

462,139,OE1,135,HH21,139-OE1-135-HH21

463,139,OE1,143,HZ3,139-OE1-143-HZ3

464,139,OE1,143,HZ1,139-OE1-143-HZ1

465,139,OE1,135,HH12,139-OE1-135-HH12

466,139,OE1,135,HE,139-OE1-135-HE

467,140,OD1,139,HE21,140-OD1-139-HE21

468,140,O,144,H,140-O-144-H

469,140,OD1,136,HH11,140-OD1-136-HH11

470,140,OD1,136,HE,140-OD1-136-HE

471,140,OD1,136,HH21,140-OD1-136-HH21

472,140,OD1,143,HZ3,140-OD1-143-HZ3

473,140,OD1,288,HE2,140-OD1-288-HE2

474,140,OD1,143,HZ1,140-OD1-143-HZ1

475,140,OD1,139,HE22,140-OD1-139-HE22

476,140,O,144,HG,140-O-144-HG

477,140,OD1,143,HZ2,140-OD1-143-HZ2

478,141,O,144,HG,141-O-144-HG

479,141,O,144,H,141-O-144-H

480,141,O,145,H,141-O-145-H

481,142,O,145,H,142-O-145-H

482,142,O,147,H,142-O-147-H

483,142,O,146,H,142-O-146-H

484,142,O,146,HG,142-O-146-HG

485,143,O,146,H,143-O-146-H

486,143,O,147,H,143-O-147-H

487,143,O,148,H,143-O-148-H

488,144,O,144,HG,144-O-144-HG

489,144,O,158,HH,144-O-158-HH

490,144,O,148,H,144-O-148-H

491,144,O,146,H,144-O-146-H

492,145,O,146,HG,145-O-146-HG

493,145,O,162,HE2,145-O-162-HE2

494,146,OG,147,H,146-OG-147-H

495,146,O,148,H,146-O-148-H

496,146,O,158,H,146-O-158-H

497,146,O,159,H,146-O-159-H

498,148,O,158,H,148-O-158-H

499,149,OG,154,HG,149-OG-154-HG

500,149,O,149,HG,149-O-149-HG

501,149,OG,150,H,149-OG-150-H

502,149,O,158,HH,149-O-158-HH

503,150,OD2,153,HG,150-OD2-153-HG

504,150,O,153,HG,150-O-153-HG

505,150,OD2,152,H,150-OD2-152-H

506,150,OD1,152,H,150-OD1-152-H

507,150,OD1,153,HG,150-OD1-153-HG

508,150,OD2,165,HZ1,150-OD2-165-HZ1

509,150,OD1,158,HH,150-OD1-158-HH

510,150,O,154,HG,150-O-154-HG

511,150,OD2,153,H,150-OD2-153-H

512,150,N,149,HG,150-N-149-HG

513,150,OD2,158,HH,150-OD2-158-HH

514,150,O,149,HG,150-O-149-HG

515,150,OD1,153,H,150-OD1-153-H

516,150,O,153,H,150-O-153-H

517,151,O,153,H,151-O-153-H

518,152,O,154,H,152-O-154-H

519,152,O,153,HG,152-O-153-HG

520,153,OG,154,H,153-OG-154-H

521,153,O,155,H,153-O-155-H

522,153,OG,156,H,153-OG-156-H

523,153,O,156,HG,153-O-156-HG

524,153,OG,156,HG,153-OG-156-HG

525,153,O,156,H,153-O-156-H

526,153,OG,155,HG1,153-OG-155-HG1

527,153,OG,155,H,153-OG-155-H

528,153,O,154,HG,153-O-154-HG

529,154,OG,155,HG1,154-OG-155-HG1

530,154,O,155,HG1,154-O-155-HG1

531,154,O,154,HG,154-O-154-HG

532,154,OG,155,H,154-OG-155-H

533,154,O,149,HG,154-O-149-HG

534,155,O,149,HG,155-O-149-HG

535,155,OG1,358,HH11,155-OG1-358-HH11

536,155,O,155,HG1,155-O-155-HG1

537,155,OG1,358,HH22,155-OG1-358-HH22

538,155,O,358,HH21,155-O-358-HH21

539,155,O,358,HE,155-O-358-HE

540,155,OG1,358,HH12,155-OG1-358-HH12

541,155,O,360,HG,155-O-360-HG

542,155,O,358,HH22,155-O-358-HH22

543,155,OG1,360,HG,155-OG1-360-HG

544,155,OG1,156,HG,155-OG1-156-HG

545,155,OG1,358,HH21,155-OG1-358-HH21

546,155,O,358,HH12,155-O-358-HH12

547,155,OG1,153,HG,155-OG1-153-HG

548,155,O,157,H,155-O-157-H

549,156,O,149,HG,156-O-149-HG

550,156,OG,356,HH21,156-OG-356-HH21

551,156,O,153,HG,156-O-153-HG

552,156,OG,155,HG1,156-OG-155-HG1

553,156,OG,358,HH21,156-OG-358-HH21

554,156,OG,356,HH22,156-OG-356-HH22

555,156,OG,358,HH22,156-OG-358-HH22

556,156,OG,358,HH11,156-OG-358-HH11

557,156,OG,153,HG,156-OG-153-HG

558,156,O,150,H,156-O-150-H

559,157,O,160,H,157-O-160-H

560,157,O,156,HG,157-O-156-HG

561,157,O,161,H,157-O-161-H

562,158,O,161,H,158-O-161-H

563,158,O,162,H,158-O-162-H

564,159,O,162,H,159-O-162-H

565,159,O,163,H,159-O-163-H

566,160,OE1,359,H,160-OE1-359-H

567,160,OE1,358,HE,160-OE1-358-HE

568,160,OE2,356,HH22,160-OE2-356-HH22

569,160,O,164,HG,160-O-164-HG

570,160,OE2,356,HH12,160-OE2-356-HH12

571,160,OE1,358,HH21,160-OE1-358-HH21

572,160,O,164,H,160-O-164-H

573,160,OE2,359,H,160-OE2-359-H

574,160,OE2,356,HH21,160-OE2-356-HH21

575,160,OE2,358,HH21,160-OE2-358-HH21

576,160,OE2,156,HG,160-OE2-156-HG

577,160,OE1,356,HH21,160-OE1-356-HH21

578,160,OE1,356,HH22,160-OE1-356-HH22

579,160,OE2,358,HE,160-OE2-358-HE

580,160,OE2,157,H,160-OE2-157-H

581,160,OE1,356,HH12,160-OE1-356-HH12

582,160,OE2,356,HE,160-OE2-356-HE

583,160,OE1,157,H,160-OE1-157-H

584,160,OE1,358,HH11,160-OE1-358-HH11

585,160,OE1,156,HG,160-OE1-156-HG

586,161,OE2,156,HG,161-OE2-156-HG

587,161,OE1,165,HZ1,161-OE1-165-HZ1

588,161,OE2,153,HG,161-OE2-153-HG

589,161,O,165,HZ2,161-O-165-HZ2

590,161,OE2,356,HH21,161-OE2-356-HH21

591,161,OE1,356,HH21,161-OE1-356-HH21

592,161,OE1,356,HE,161-OE1-356-HE

593,161,OE1,153,HG,161-OE1-153-HG

594,161,O,165,HZ1,161-O-165-HZ1

595,161,O,165,H,161-O-165-H

596,161,OE1,165,HZ2,161-OE1-165-HZ2

597,161,OE2,356,HH22,161-OE2-356-HH22
598,161,OE2,356,HE,161-OE2-356-HE
599,161,OE2,165,HZ1,161-OE2-165-HZ1
600,161,OE2,165,HZ2,161-OE2-165-HZ2
601,161,OE1,156,HG,161-OE1-156-HG
602,161,OE1,356,HH22,161-OE1-356-HH22
603,161,OE2,165,HZ3,161-OE2-165-HZ3
604,161,OE2,358,HH22,161-OE2-358-HH22
605,161,OE1,358,HH12,161-OE1-358-HH12
606,161,O,165,HZ3,161-O-165-HZ3
607,161,OE1,165,HZ3,161-OE1-165-HZ3
608,161,OE1,356,HH12,161-OE1-356-HH12
609,161,OE2,356,HH12,161-OE2-356-HH12
610,162,ND1,165,HZ2,162-ND1-165-HZ2
611,162,O,166,H,162-O-166-H
612,162,ND1,165,HZ1,162-ND1-165-HZ1
613,162,ND1,165,HZ3,162-ND1-165-HZ3
614,162,O,165,H,162-O-165-H
615,163,O,167,H,163-O-167-H
616,163,O,166,H,163-O-166-H
617,164,OG,356,HE,164-OG-356-HE
618,164,OG,356,HH21,164-OG-356-HH21
619,164,OG,356,HH11,164-OG-356-HH11
620,164,O,168,H,164-O-168-H
621,165,O,168,H,165-O-168-H
622,165,O,169,H,165-O-169-H
623,166,O,193,HG,166-O-193-HG
624,166,OE2,206,HH21,166-OE2-206-HH21
625,166,OE1,196,HD22,166-OE1-196-HD22
626,166,OE1,206,HH21,166-OE1-206-HH21
627,166,OE1,206,HE,166-OE1-206-HE
628,166,O,170,H,166-O-170-H
629,166,O,169,H,166-O-169-H
630,166,OE2,165,HZ1,166-OE2-165-HZ1
631,166,OE2,206,HE,166-OE2-206-HE
632,167,O,170,H,167-O-170-H
633,167,O,171,H,167-O-171-H
634,168,OE2,356,HH21,168-OE2-356-HH21
635,168,OE1,477,HE,168-OE1-477-HE
636,168,OE1,356,HE,168-OE1-356-HE
637,168,OE1,477,HH12,168-OE1-477-HH12
638,168,OE1,477,HH21,168-OE1-477-HH21
639,168,OE1,172,HG,168-OE1-172-HG
640,168,OE2,477,HH21,168-OE2-477-HH21
641,168,OE1,477,HH22,168-OE1-477-HH22
642,168,OE2,165,HZ2,168-OE2-165-HZ2
643,168,OE2,165,HZ3,168-OE2-165-HZ3
644,168,OE2,477,HH12,168-OE2-477-HH12
645,168,OE2,356,HE,168-OE2-356-HE
646,168,O,172,HG,168-O-172-HG
647,168,OE2,477,HE,168-OE2-477-HE
648,168,OE1,356,HH21,168-OE1-356-HH21
649,168,O,172,H,168-O-172-H
650,168,OE2,356,HH11,168-OE2-356-HH11
651,168,OE1,477,HH11,168-OE1-477-HH11
652,168,O,171,H,168-O-171-H
653,168,OE2,477,HH22,168-OE2-477-HH22
654,169,O,172,H,169-O-172-H
655,169,O,173,HG1,169-O-173-HG1
656,169,O,173,H,169-O-173-H
657,169,O,172,HG,169-O-172-HG
658,170,O,174,H,170-O-174-H
659,170,O,173,H,170-O-173-H
660,171,O,174,H,171-O-174-H
661,171,O,175,H,171-O-175-H
662,171,O,175,HE21,171-O-175-HE21
663,172,O,175,HE21,172-O-175-HE21
664,172,O,176,H,172-O-176-H
665,173,O,176,H,173-O-176-H
666,173,OG1,209,HE2,173-OG1-209-HE2
667,173,O,177,H,173-O-177-H
668,174,O,178,H,174-O-178-H
669,174,O,177,H,174-O-177-H
670,175,O,175,HE21,175-O-175-HE21
671,175,O,178,H,175-O-178-H
672,175,O,179,H,175-O-179-H
673,176,O,179,H,176-O-179-H
674,176,O,180,H,176-O-180-H
675,177,O,180,H,177-O-180-H
676,177,O,183,H,177-O-183-H

677,177,O,182,H,177-O-182-H
678,178,O,180,H,178-O-180-H
679,181,O,183,H,181-O-183-H
680,182,O,507,HE21,182-O-507-HE21
681,183,ND1,183,H,183-ND1-183-H
682,183,ND1,507,HE21,183-ND1-507-HE21
683,184,O,506,H,184-O-506-H
684,185,OD1,188,H,185-OD1-188-H
685,185,OD1,187,H,185-OD1-187-H
686,186,O,190,H,186-O-190-H
687,186,O,189,H,186-O-189-H
688,186,O,327,HG,186-O-327-HG
689,187,OH,499,HZ3,187-OH-499-HZ3
690,187,OH,324,HG1,187-OH-324-HG1
691,187,OH,499,HZ2,187-OH-499-HZ2
692,187,O,191,H,187-O-191-H
693,187,OH,219,HD22,187-OH-219-HD22
694,187,O,190,H,187-O-190-H
695,187,OH,499,HZ1,187-OH-499-HZ1
696,188,O,188,HH11,188-O-188-HH11
697,188,O,219,HD21,188-O-219-HD21
698,188,NH2,185,HD22,188-NH2-185-HD22
699,189,O,209,HE2,189-O-209-HE2
700,189,OH,188,HH11,189-OH-188-HH11
701,189,O,193,HG,189-O-193-HG
702,189,O,192,H,189-O-192-H
703,189,O,193,H,189-O-193-H
704,190,O,193,H,190-O-193-H
705,190,O,193,HG,190-O-193-HG
706,190,O,194,H,190-O-194-H
707,191,O,195,HG1,191-O-195-HG1
708,191,O,195,H,191-O-195-H
709,192,O,196,H,192-O-196-H
710,193,O,197,H,193-O-197-H
711,193,O,196,H,193-O-196-H
712,193,OG,209,HE2,193-OG-209-HE2
713,194,O,197,H,194-O-197-H
714,194,O,198,H,194-O-198-H
715,195,O,199,H,195-O-199-H
716,196,O,200,H,196-O-200-H
717,196,OD1,207,H,196-OD1-207-H
718,196,ND2,207,H,196-ND2-207-H
719,197,O,201,H,197-O-201-H
720,197,O,200,H,197-O-200-H
721,198,O,202,H,198-O-202-H
722,199,O,202,H,199-O-202-H
723,199,O,203,H,199-O-203-H
724,199,O,204,H,199-O-204-H
725,199,O,205,H,199-O-205-H
726,200,O,204,H,200-O-204-H
727,201,O,281,H,201-O-281-H
728,202,O,281,H,202-O-281-H
729,202,O,282,H,202-O-282-H
730,202,O,282,HG1,202-O-282-HG1
731,203,O,205,HE,203-O-205-HE
732,203,O,205,HH11,203-O-205-HH11
733,203,O,205,H,203-O-205-H
734,203,O,205,HH21,203-O-205-HH21
735,204,O,205,HE,204-O-205-HE
736,205,NH2,269,HE2,205-NH2-269-HE2
737,206,O,205,HH21,206-O-205-HH21
738,207,OH,205,HH11,207-OH-205-HH11
739,207,O,196,HD21,207-O-196-HD21
740,208,OD2,206,HH22,208-OD2-206-HH22
741,208,O,210,H,208-O-210-H
742,208,O,211,H,208-O-211-H
743,208,OD2,206,HH12,208-OD2-206-HH12
744,208,OD2,209,H,208-OD2-209-H
745,208,OD1,210,HD22,208-OD1-210-HD22
746,208,OD2,210,HD22,208-OD2-210-HD22
747,208,OD1,209,H,208-OD1-209-H
748,208,OD2,210,HD21,208-OD2-210-HD21
749,208,OD2,206,HH11,208-OD2-206-HH11
750,208,OD2,208,H,208-OD2-208-H
751,208,OD2,211,H,208-OD2-211-H
752,208,OD1,206,HH22,208-OD1-206-HH22
753,208,OD1,210,HD21,208-OD1-210-HD21
754,208,OD2,210,H,208-OD2-210-H
755,208,OD1,210,H,208-OD1-210-H
756,208,OD1,208,H,208-OD1-208-H

757,208,OD1,206,HH12,208-OD1-206-HH12

758,209,O,188,HH11,209-O-188-HH11

759,209,ND1,173,HG1,209-ND1-173-HG1

760,209,ND1,188,HH12,209-ND1-188-HH12

761,209,O,212,HE22,209-O-212-HE22

762,209,O,188,HH12,209-O-188-HH12

763,209,O,188,HH22,209-O-188-HH22

764,209,ND1,209,H,209-ND1-209-H

765,210,O,212,H,210-O-212-H

766,210,O,210,HD22,210-O-210-HD22

767,210,OD1,188,HH22,210-OD1-188-HH22

768,210,O,212,HE21,210-O-212-HE21

769,210,OD1,188,HH12,210-OD1-188-HH12

770,211,O,214,H,211-O-214-H

771,211,ND1,212,H,211-ND1-212-H

772,211,ND1,214,H,211-ND1-214-H

773,211,ND1,213,H,211-ND1-213-H

774,211,O,215,H,211-O-215-H

775,212,OE1,212,H,212-OE1-212-H

776,212,O,216,HG,212-O-216-HG

777,212,O,212,HE21,212-O-212-HE21

778,212,OE1,216,HG,212-OE1-216-HG

779,212,O,216,H,212-O-216-H

780,212,OE1,213,H,212-OE1-213-H

781,213,OE2,257,HZ3,213-OE2-257-HZ3

782,213,O,217,H,213-O-217-H

783,213,O,257,HZ1,213-O-257-HZ1

784,213,OE2,257,HZ2,213-OE2-257-HZ2

785,213,OE1,257,HZ3,213-OE1-257-HZ3

786,213,OE1,216,HG,213-OE1-216-HG

787,213,OE2,264,HZ3,213-OE2-264-HZ3

788,213,OE2,213,H,213-OE2-213-H

789,213,OE1,257,HZ1,213-OE1-257-HZ1

790,213,OE1,264,HZ1,213-OE1-264-HZ1

791,213,OE1,213,H,213-OE1-213-H

792,213,O,216,H,213-O-216-H

793,213,OE2,264,HZ1,213-OE2-264-HZ1

794,213,OE2,211,HE2,213-OE2-211-HE2

795,213,OE2,257,HZ1,213-OE2-257-HZ1

796,213,OE2,264,HZ2,213-OE2-264-HZ2

797,213,OE2,212,HE21,213-OE2-212-HE21

798,213,OE1,257,HZ2,213-OE1-257-HZ2

799,213,OE2,212,HE22,213-OE2-212-HE22

800,213,OE1,207,HH,213-OE1-207-HH

801,213,OE1,212,HE21,213-OE1-212-HE21

802,213,OE1,264,HZ3,213-OE1-264-HZ3

803,213,O,216,HG,213-O-216-HG

804,213,OE1,264,HZ2,213-OE1-264-HZ2

805,213,OE2,216,HG,213-OE2-216-HG

806,214,O,217,H,214-O-217-H

807,214,O,218,H,214-O-218-H

808,215,O,218,H,215-O-218-H

809,215,O,220,H,215-O-220-H

810,215,O,219,H,215-O-219-H

811,215,O,219,HD22,215-O-219-HD22

812,216,OG,212,HE21,216-OG-212-HE21

813,216,O,257,HZ3,216-O-257-HZ3

814,216,O,221,H,216-O-221-H

815,216,OG,257,HZ2,216-OG-257-HZ2

816,216,O,257,HZ1,216-O-257-HZ1

817,216,O,220,H,216-O-220-H

818,216,OG,257,HZ1,216-OG-257-HZ1

819,216,OG,257,HZ3,216-OG-257-HZ3

820,216,O,222,H,216-O-222-H

821,216,OG,221,HD22,216-OG-221-HD22

822,216,O,221,HD22,216-O-221-HD22

823,216,O,257,HZ2,216-O-257-HZ2

824,216,O,219,H,216-O-219-H

825,217,O,222,HD21,217-O-222-HD21

826,218,O,222,HD21,218-O-222-HD21

827,219,OD1,188,HH21,219-OD1-188-HH21

828,219,OD1,188,HE,219-OD1-188-HE

829,219,O,219,HD22,219-O-219-HD22

830,219,O,222,H,219-O-222-H

831,220,O,221,HD22,220-O-221-HD22

832,220,O,222,H,220-O-222-H

833,221,OD1,257,HZ1,221-OD1-257-HZ1

834,221,O,223,H,221-O-223-H

835,221,O,221,HD22,221-O-221-HD22

836,221,O,223,HD22,221-O-223-HD22

837,221,OD1,216,HG,221-OD1-216-HG

838,221,OD1,257,HZ3,221-OD1-257-HZ3

839,221,OD1,257,HZ2,221-OD1-257-HZ2

840,222,OD1,224,H,222-OD1-224-H

841,223,O,223,HD22,223-O-223-HD22

842,223,O,226,H,223-O-226-H

843,223,O,227,H,223-O-227-H

844,224,O,227,H,224-O-227-H

845,224,O,226,H,224-O-226-H

846,224,O,228,H,224-O-228-H

847,225,O,229,H,225-O-229-H

848,225,O,497,H,225-O-497-H

849,225,O,496,H,225-O-496-H

850,226,OE2,57,HZ1,226-OE2-57-HZ1

851,226,OE1,57,HZ3,226-OE1-57-HZ3

852,226,OE1,247,HG,226-OE1-247-HG

853,226,OE1,232,HD21,226-OE1-232-HD21

854,226,OE2,223,HD22,226-OE2-223-HD22

855,226,OE2,247,HG,226-OE2-247-HG

856,226,OE2,57,HZ2,226-OE2-57-HZ2

857,226,OE2,226,H,226-OE2-226-H

858,226,O,232,HD21,226-O-232-HD21

859,226,OE1,57,HZ1,226-OE1-57-HZ1

860,226,OE2,57,HZ3,226-OE2-57-HZ3

861,226,O,229,H,226-O-229-H

862,226,OE1,223,HD22,226-OE1-223-HD22

863,226,O,232,HD22,226-O-232-HD22

864,226,OE1,226,H,226-OE1-226-H

865,226,OE1,57,HZ2,226-OE1-57-HZ2

866,227,O,232,HD22,227-O-232-HD22

867,227,O,229,H,227-O-229-H

868,228,O,230,H,228-O-230-H

869,228,O,232,HD21,228-O-232-HD21

870,228,O,496,H,228-O-496-H

871,229,O,494,HH,229-O-494-HH

872,229,O,232,HD22,229-O-232-HD22

873,229,O,232,H,229-O-232-H

874,229,N,232,HD21,229-N-232-HD21

875,230,O,494,HH,230-O-494-HH

876,230,OG,231,H,230-OG-231-H

877,230,OG,494,HH,230-OG-494-HH

878,232,OD1,234,H,232-OD1-234-H

879,232,OD1,494,HH,232-OD1-494-HH

880,232,OD1,235,H,232-OD1-235-H

881,232,O,235,H,232-O-235-H

882,232,O,234,H,232-O-234-H

883,232,O,236,H,232-O-236-H

884,233,O,235,H,233-O-235-H

885,233,O,236,H,233-O-236-H

886,233,O,237,H,233-O-237-H

887,234,O,241,HH11,234-O-241-HH11

888,234,O,237,H,234-O-237-H

889,234,O,241,H,234-O-241-H

890,235,OD1,111,H,235-OD1-111-H

891,235,OD2,111,HG1,235-OD2-111-HG1

892,235,OD2,232,HD22,235-OD2-232-HD22

893,235,OD1,110,H,235-OD1-110-H

894,235,OD1,111,HG1,235-OD1-111-HG1

895,235,OD1,241,HH11,235-OD1-241-HH11

896,235,OD1,232,HD22,235-OD1-232-HD22

897,235,O,241,HH11,235-O-241-HH11

898,235,OD1,241,HH12,235-OD1-241-HH12

899,235,OD2,111,H,235-OD2-111-H

900,235,O,241,HH12,235-O-241-HH12

901,235,OD2,110,H,235-OD2-110-H

902,235,OD1,241,HH22,235-OD1-241-HH22

903,235,OD2,241,HH11,235-OD2-241-HH11

904,236,O,80,H,236-O-80-H

905,236,O,81,HG1,236-O-81-HG1

906,237,O,240,H,237-O-240-H

907,237,O,241,H,237-O-241-H

908,238,O,241,H,238-O-241-H

909,238,O,242,H,238-O-242-H

910,238,O,241,HE,238-O-241-HE

911,239,O,243,H,239-O-243-H

912,239,O,241,H,239-O-241-H

913,239,O,242,H,239-O-242-H

914,240,O,245,HD21,240-O-245-HD21

915,240,O,242,H,240-O-242-H

916,240,O,245,HD22,240-O-245-HD22

917,240,O,243,H,240-O-243-H

918,241,NH1,110,H,241-NH1-110-H

919,242,OH,241,HH21,242-OH-241-HH21

920,242,OH,241,HE,242-OH-241-HE

921,243,O,245,H,243-O-245-H

922,243,O,245,HD22,243-O-245-HD22

923,244,O,249,HD21,244-O-249-HD21

924,245,OD1,248,H,245-OD1-248-H

925,245,OD1,247,H,245-OD1-247-H

926,245,O,248,H,245-O-248-H

927,245,O,249,H,245-O-249-H

928,245,O,249,HD22,245-O-249-HD22

929,245,OD1,247,HG,245-OD1-247-HG

930,245,ND2,248,H,245-ND2-248-H

931,246,O,249,H,246-O-249-H

932,246,O,250,H,246-O-250-H

933,247,O,250,H,247-O-250-H

934,247,O,223,HD21,247-O-223-HD21

935,247,OG,245,HD21,247-OG-245-HD21

936,247,O,251,H,247-O-251-H

937,247,OG,223,HD22,247-OG-223-HD22

938,248,O,251,H,248-O-251-H

939,248,O,252,H,248-O-252-H

940,249,OD1,252,HZ3,249-OD1-252-HZ3

941,249,OD1,252,HZ2,249-OD1-252-HZ2

942,249,OD1,252,HZ1,249-OD1-252-HZ1

943,249,O,253,H,249-O-253-H

944,249,O,252,H,249-O-252-H

945,249,OD1,245,H,249-OD1-245-H

946,249,O,249,HD22,249-O-249-HD22

947,250,O,253,H,250-O-253-H

948,250,O,223,HD21,250-O-223-HD21

949,250,O,254,H,250-O-254-H

950,251,O,254,H,251-O-254-H

951,251,O,255,HD22,251-O-255-HD22

952,251,N,223,HD21,251-N-223-HD21

953,251,O,255,H,251-O-255-H

954,252,O,256,H,252-O-256-H

955,252,O,255,H,252-O-255-H

956,253,OD2,249,HD21,253-OD2-249-HD21

957,253,OD1,252,HZ1,253-OD1-252-HZ1

958,253,OD2,221,HD21,253-OD2-221-HD21

959,253,OD2,252,HZ1,253-OD2-252-HZ1

960,253,OD1,252,HZ2,253-OD1-252-HZ2

961,253,OD2,257,HZ3,253-OD2-257-HZ3

962,253,OD2,252,HZ3,253-OD2-252-HZ3

963,253,OD1,257,HZ1,253-OD1-257-HZ1

964,253,OD2,257,HZ1,253-OD2-257-HZ1

965,253,OD1,257,HZ3,253-OD1-257-HZ3

966,253,OD2,257,HZ2,253-OD2-257-HZ2

967,253,OD1,252,HZ3,253-OD1-252-HZ3

968,253,O,257,H,253-O-257-H

969,253,OD1,257,HZ2,253-OD1-257-HZ2

970,253,OD2,252,HZ2,253-OD2-252-HZ2

971,253,O,256,H,253-O-256-H

972,253,O,257,HZ2,253-O-257-HZ2

973,253,O,257,HZ3,253-O-257-HZ3

974,254,O,257,H,254-O-257-H

975,254,O,258,H,254-O-258-H

976,255,OD1,116,H,255-OD1-116-H

977,255,N,255,HD22,255-N-255-HD22

978,255,O,259,H,255-O-259-H

979,255,O,258,H,255-O-258-H

980,256,OE1,257,HZ2,256-OE1-257-HZ2

981,256,OE1,252,HZ3,256-OE1-252-HZ3

982,256,OE1,257,HZ1,256-OE1-257-HZ1

983,256,OE1,117,HD21,256-OE1-117-HD21

984,256,O,259,H,256-O-259-H

985,256,OE1,257,HZ3,256-OE1-257-HZ3

986,256,O,260,H,256-O-260-H

987,256,O,260,HG,256-O-260-HG

988,256,OE2,257,HZ3,256-OE2-257-HZ3

989,256,OE2,257,HZ2,256-OE2-257-HZ2

990,256,OE2,252,HZ1,256-OE2-252-HZ1

991,256,OE2,260,HG,256-OE2-260-HG

992,256,OE2,257,HZ1,256-OE2-257-HZ1

993,256,OE1,260,HG,256-OE1-260-HG

994,256,OE2,259,HH,256-OE2-259-HH

995,256,OE2,252,HZ2,256-OE2-252-HZ2

996,256,OE2,252,HZ3,256-OE2-252-HZ3

997,256,OE1,252,HZ1,256-OE1-252-HZ1
998,256,OE1,252,HZ2,256-OE1-252-HZ2
999,256,OE2,117,HD21,256-OE2-117-HD21
1000,257,O,260,H,257-O-260-H
1001,257,O,260,HG,257-O-260-HG
1002,257,O,261,H,257-O-261-H
1003,258,O,262,H,258-O-262-H
1004,258,O,261,H,258-O-261-H
1005,259,OH,119,HE22,259-OH-119-HE22
1006,259,OH,117,H,259-OH-117-H
1007,259,OH,117,HD22,259-OH-117-HD22
1008,259,OH,309,HD21,259-OH-309-HD21
1009,259,OH,117,HD21,259-OH-117-HD21
1010,259,O,263,H,259-O-263-H
1011,259,OH,116,HG,259-OH-116-HG
1012,259,O,262,H,259-O-262-H
1013,259,OH,309,HD22,259-OH-309-HD22
1014,259,OH,263,HE21,259-OH-263-HE21
1015,259,O,263,HE21,259-O-263-HE21
1016,260,O,264,HZ3,260-O-264-HZ3
1017,260,OG,264,HZ3,260-OG-264-HZ3
1018,260,O,264,H,260-O-264-H
1019,260,O,264,HZ2,260-O-264-HZ2
1020,260,OG,264,HZ2,260-OG-264-HZ2
1021,260,OG,264,HZ1,260-OG-264-HZ1
1022,261,O,264,H,261-O-264-H
1023,261,O,265,H,261-O-265-H
1024,262,O,265,H,262-O-265-H
1025,262,O,267,H,262-O-267-H
1026,262,O,266,H,262-O-266-H
1027,263,OE1,267,HZ2,263-OE1-267-HZ2
1028,263,NE2,259,HH,263-NE2-259-HH
1029,263,OE1,267,HZ3,263-OE1-267-HZ3
1030,263,OE1,267,HZ1,263-OE1-267-HZ1
1031,263,O,267,HZ1,263-O-267-HZ1
1032,263,O,266,H,263-O-266-H
1033,263,O,267,H,263-O-267-H
1034,263,O,263,HE21,263-O-263-HE21
1035,263,OE1,264,HZ3,263-OE1-264-HZ3
1036,263,OE1,259,HH,263-OE1-259-HH
1037,263,O,268,H,263-O-268-H
1038,263,OE1,267,H,263-OE1-267-H
1039,263,OE1,264,HZ1,263-OE1-264-HZ1
1040,264,O,267,H,264-O-267-H
1041,264,O,205,HH12,264-O-205-HH12
1042,264,O,268,H,264-O-268-H
1043,264,O,205,HH22,264-O-205-HH22
1044,265,O,205,HH21,265-O-205-HH21
1045,265,O,205,HH22,265-O-205-HH22
1046,265,O,268,H,265-O-268-H
1047,265,O,269,H,265-O-269-H
1048,265,O,205,HH12,265-O-205-HH12
1049,266,O,270,H,266-O-270-H
1050,266,O,269,H,266-O-269-H
1051,267,O,271,HZ2,267-O-271-HZ2
1052,267,O,270,H,267-O-270-H
1053,267,O,271,HZ1,267-O-271-HZ1
1054,267,O,269,H,267-O-269-H
1055,267,O,271,H,267-O-271-H
1056,267,O,271,HZ3,267-O-271-HZ3
1057,268,OE1,264,HZ1,268-OE1-264-HZ1
1058,268,OE1,267,HZ3,268-OE1-267-HZ3
1059,268,OE2,264,HZ3,268-OE2-264-HZ3
1060,268,OE1,267,HZ2,268-OE1-267-HZ2
1061,268,OE2,264,HZ2,268-OE2-264-HZ2
1062,268,OE1,264,HZ3,268-OE1-264-HZ3
1063,268,OE2,264,HZ1,268-OE2-264-HZ1
1064,268,OE2,271,HZ3,268-OE2-271-HZ3
1065,268,OE1,271,HZ3,268-OE1-271-HZ3
1066,268,OE2,267,HZ2,268-OE2-267-HZ2
1067,268,OE1,271,HZ1,268-OE1-271-HZ1
1068,268,OE1,263,HE21,268-OE1-263-HE21
1069,268,OE1,205,HH12,268-OE1-205-HH12
1070,268,OE1,267,HZ1,268-OE1-267-HZ1
1071,268,OE2,263,HE21,268-OE2-263-HE21
1072,268,OE1,263,HE22,268-OE1-263-HE22
1073,268,OE2,271,HZ2,268-OE2-271-HZ2
1074,268,O,271,HZ3,268-O-271-HZ3
1075,268,OE1,264,HZ2,268-OE1-264-HZ2
1076,268,OE1,271,HZ2,268-OE1-271-HZ2

1077,268,O,271,HZ2,268-O-271-HZ2

1078,268,O,272,H,268-O-272-H

1079,268,O,271,HZ1,268-O-271-HZ1

1080,268,OE2,205,HH11,268-OE2-205-HH11

1081,268,OE2,205,HH22,268-OE2-205-HH22

1082,268,OE2,267,HZ1,268-OE2-267-HZ1

1083,268,O,271,H,268-O-271-H

1084,268,OE2,267,HZ3,268-OE2-267-HZ3

1085,268,OE1,205,HH22,268-OE1-205-HH22

1086,268,OE2,205,HH12,268-OE2-205-HH12

1087,268,OE1,269,HE2,268-OE1-269-HE2

1088,268,OE1,205,HH11,268-OE1-205-HH11

1089,268,O,272,HG1,268-O-272-HG1

1090,268,OE2,271,HZ1,268-OE2-271-HZ1

1091,268,OE2,205,HE,268-OE2-205-HE

1092,269,O,273,H,269-O-273-H

1093,269,O,279,HH22,269-O-279-HH22

1094,269,O,279,HH12,269-O-279-HH12

1095,269,O,272,H,269-O-272-H

1096,269,ND1,205,HH12,269-ND1-205-HH12

1097,269,O,272,HG1,269-O-272-HG1

1098,269,ND1,205,HH21,269-ND1-205-HH21

1099,269,ND1,205,HH22,269-ND1-205-HH22

1100,270,O,272,H,270-O-272-H

1101,270,O,273,H,270-O-273-H

1102,270,OH,290,HE21,270-OH-290-HE21

1103,270,OH,293,HE22,270-OH-293-HE22

1104,271,O,273,H,271-O-273-H

1105,271,O,271,HZ1,271-O-271-HZ1

1106,272,OG1,279,HH12,272-OG1-279-HH12

1107,272,O,279,HH22,272-O-279-HH22

1108,272,OG1,279,HH22,272-OG1-279-HH22

1109,272,O,279,HH12,272-O-279-HH12

1110,274,OE2,275,H,274-OE2-275-H

1111,274,O,277,H,274-O-277-H

1112,274,OE1,275,H,274-OE1-275-H

1113,274,OE2,276,H,274-OE2-276-H

1114,274,OE1,277,H,274-OE1-277-H

1115,274,O,278,H,274-O-278-H

1116,274,OE1,276,H,274-OE1-276-H

1117,274,O,276,H,274-O-276-H

1118,275,O,277,H,275-O-277-H

1119,276,O,278,H,276-O-278-H

1120,277,ND1,278,H,277-ND1-278-H

1121,280,OD1,282,HG1,280-OD1-282-HG1

1122,280,OD2,282,HG1,280-OD2-282-HG1

1123,280,O,284,HG,280-O-284-HG

1124,280,OD1,205,HH21,280-OD1-205-HH21

1125,280,OD2,283,H,280-OD2-283-H

1126,280,OD2,282,H,280-OD2-282-H

1127,280,OD1,283,H,280-OD1-283-H

1128,280,OD1,282,H,280-OD1-282-H

1129,280,O,284,H,280-O-284-H

1130,280,OD2,269,HE2,280-OD2-269-HE2

1131,280,OD1,269,HE2,280-OD1-269-HE2

1132,280,O,283,H,280-O-283-H

1133,281,O,284,H,281-O-284-H

1134,281,O,284,HG,281-O-284-HG

1135,281,O,285,H,281-O-285-H

1136,282,O,286,H,282-O-286-H

1137,282,O,285,H,282-O-285-H

1138,283,O,286,H,283-O-286-H

1139,283,OD2,279,HH21,283-OD2-279-HH21

1140,283,OD1,279,HH21,283-OD1-279-HH21

1141,283,OD2,279,H,283-OD2-279-H

1142,283,O,287,H,283-O-287-H

1143,283,OD1,279,HE,283-OD1-279-HE

1144,283,OD1,269,HE2,283-OD1-269-HE2

1145,283,OD2,279,HE,283-OD2-279-HE

1146,284,O,284,HG,284-O-284-HG

1147,284,O,287,H,284-O-287-H

1148,284,O,288,H,284-O-288-H

1149,285,O,288,H,285-O-288-H

1150,285,O,289,H,285-O-289-H

1151,286,O,289,H,286-O-289-H

1152,286,O,290,H,286-O-290-H

1153,286,O,290,HE21,286-O-290-HE21

1154,286,O,290,HE22,286-O-290-HE22

1155,287,OE1,275,HZ2,287-OE1-275-HZ2

1156,287,OE2,284,HG,287-OE2-284-HG

1157,287,O,291,H,287-O-291-H

1158,287,OE2,275,HZ1,287-OE2-275-HZ1

1159,287,OE2,290,HE21,287-OE2-290-HE21

1160,287,OE2,275,HZ2,287-OE2-275-HZ2

1161,287,OE1,275,HZ3,287-OE1-275-HZ3

1162,287,OE2,275,HZ3,287-OE2-275-HZ3

1163,287,O,290,H,287-O-290-H

1164,287,OE1,275,HZ1,287-OE1-275-HZ1

1165,287,O,275,HZ3,287-O-275-HZ3

1166,288,O,292,HZ1,288-O-292-HZ1

1167,288,O,292,HZ3,288-O-292-HZ3

1168,288,O,292,HZ2,288-O-292-HZ2

1169,288,O,292,H,288-O-292-H

1170,288,O,291,H,288-O-291-H

1171,289,O,292,HZ2,289-O-292-HZ2

1172,289,O,293,H,289-O-293-H

1173,289,O,292,HZ3,289-O-292-HZ3

1174,289,O,292,HZ1,289-O-292-HZ1

1175,289,O,293,HE21,289-O-293-HE21

1176,289,O,292,H,289-O-292-H

1177,290,O,293,HE21,290-O-293-HE21

1178,290,O,292,H,290-O-292-H

1179,290,OE1,275,HZ1,290-OE1-275-HZ1

1180,290,NE2,275,HZ2,290-NE2-275-HZ2

1181,290,OE1,293,HE21,290-OE1-293-HE21

1182,290,O,293,HE22,290-O-293-HE22

1183,290,OE1,293,HE22,290-OE1-293-HE22

1184,290,OE1,275,HZ3,290-OE1-275-HZ3

1185,290,O,290,HE21,290-O-290-HE21

1186,290,O,294,H,290-O-294-H

1187,290,OE1,270,HH,290-OE1-270-HH

1188,290,O,293,H,290-O-293-H

1189,290,NE2,275,HZ1,290-NE2-275-HZ1

1190,290,OE1,275,HZ2,290-OE1-275-HZ2

1191,291,O,295,H,291-O-295-H

1192,291,OE2,290,HE21,291-OE2-290-HE21

1193,291,OE2,290,HE22,291-OE2-290-HE22

1194,291,O,294,H,291-O-294-H

1195,291,OE1,275,HZ1,291-OE1-275-HZ1

1196,291,OE2,275,HZ2,291-OE2-275-HZ2

1197,291,OE2,275,HZ1,291-OE2-275-HZ1

1198,291,OE1,290,HE22,291-OE1-290-HE22

1199,291,OE1,293,HE22,291-OE1-293-HE22

1200,291,OE2,293,HE22,291-OE2-293-HE22

1201,291,OE2,275,HZ3,291-OE2-275-HZ3

1202,291,O,293,H,291-O-293-H

1203,291,OE1,290,HE21,291-OE1-290-HE21

1204,291,OE1,275,HZ3,291-OE1-275-HZ3

1205,291,OE1,275,HZ2,291-OE1-275-HZ2

1206,292,O,294,H,292-O-294-H

1207,292,O,299,HD22,292-O-299-HD22

1208,292,O,295,H,292-O-295-H

1209,292,O,297,HD22,292-O-297-HD22

1210,293,OE1,290,HE22,293-OE1-290-HE22

1211,293,OE1,293,H,293-OE1-293-H

1212,293,OE1,290,HE21,293-OE1-290-HE21

1213,293,O,293,HE21,293-O-293-HE21

1214,293,O,295,H,293-O-295-H

1215,295,OD2,297,H,295-OD2-297-H

1216,295,OD1,299,H,295-OD1-299-H

1217,295,OD2,288,HE2,295-OD2-288-HE2

1218,295,OD1,298,H,295-OD1-298-H

1219,295,OD2,297,HD21,295-OD2-297-HD21

1220,295,OD2,136,HE,295-OD2-136-HE

1221,295,OD1,136,HE,295-OD1-136-HE

1222,295,O,298,H,295-O-298-H

1223,295,OD2,136,HH21,295-OD2-136-HH21

1224,295,OD2,296,H,295-OD2-296-H

1225,295,O,297,H,295-O-297-H

1226,295,OD1,136,HH21,295-OD1-136-HH21

1227,295,OD2,299,HD21,295-OD2-299-HD21

1228,295,OD1,299,HD22,295-OD1-299-HD22

1229,295,OD1,297,HD21,295-OD1-297-HD21

1230,295,O,297,HD21,295-O-297-HD21

1231,295,OD1,297,H,295-OD1-297-H

1232,295,OD1,288,HE2,295-OD1-288-HE2

1233,295,OD1,299,HD21,295-OD1-299-HD21

1234,295,OD1,296,H,295-OD1-296-H

1235,296,OE2,297,HD22,296-OE2-297-HD22

1236,296,OE2,140,HD21,296-OE2-140-HD21

1237,296,O,297,HD21,296-O-297-HD21
1238,296,OE1,140,HD21,296-OE1-140-HD21
1239,296,OE2,297,HD21,296-OE2-297-HD21
1240,296,O,298,H,296-O-298-H
1241,296,OE1,136,HH12,296-OE1-136-HH12
1242,296,OE2,136,HH22,296-OE2-136-HH22
1243,296,OE2,136,HH12,296-OE2-136-HH12
1244,296,OE1,296,H,296-OE1-296-H
1245,296,OE1,136,HH11,296-OE1-136-HH11
1246,296,OE2,296,H,296-OE2-296-H
1247,296,OE1,136,HH22,296-OE1-136-HH22
1248,296,OE1,297,HD21,296-OE1-297-HD21
1249,297,OD1,136,HH12,297-OD1-136-HH12
1250,297,O,297,HD21,297-O-297-HD21
1251,297,OD1,301,HE22,297-OD1-301-HE22
1252,297,OD1,299,H,297-OD1-299-H
1253,297,O,299,HD22,297-O-299-HD22
1254,297,ND2,136,HH22,297-ND2-136-HH22
1255,297,OD1,299,HD22,297-OD1-299-HD22
1256,297,OD1,292,HZ1,297-OD1-292-HZ1
1257,297,OD1,136,HH22,297-OD1-136-HH22
1258,297,O,299,H,297-O-299-H
1259,297,OD1,136,HH21,297-OD1-136-HH21
1260,297,ND2,297,H,297-ND2-297-H
1261,297,OD1,299,HD21,297-OD1-299-HD21
1262,297,OD1,298,H,297-OD1-298-H
1263,297,ND2,299,H,297-ND2-299-H
1264,297,OD1,292,HZ3,297-OD1-292-HZ3
1265,298,O,292,HZ2,298-O-292-HZ2
1266,298,O,292,HZ1,298-O-292-HZ1
1267,298,O,292,HZ3,298-O-292-HZ3
1268,298,O,300,H,298-O-300-H
1269,299,O,292,HZ1,299-O-292-HZ1
1270,299,O,302,H,299-O-302-H
1271,299,O,292,HZ2,299-O-292-HZ2
1272,299,OD1,297,HD21,299-OD1-297-HD21
1273,299,OD1,299,H,299-OD1-299-H
1274,299,O,292,HZ3,299-O-292-HZ3
1275,299,OD1,288,HE2,299-OD1-288-HE2
1276,299,OD1,302,H,299-OD1-302-H
1277,299,O,297,HD22,299-O-297-HD22
1278,299,OD1,136,HH22,299-OD1-136-HH22
1279,299,OD1,301,H,299-OD1-301-H
1280,299,OD1,301,HE21,299-OD1-301-HE21
1281,299,OD1,297,HD22,299-OD1-297-HD22
1282,300,O,303,H,300-O-303-H
1283,300,O,306,HZ1,300-O-306-HZ1
1284,300,O,301,HE21,300-O-301-HE21
1285,300,O,302,H,300-O-302-H
1286,300,O,292,HZ2,300-O-292-HZ2
1287,300,O,306,HZ3,300-O-306-HZ3
1288,300,O,292,HZ1,300-O-292-HZ1
1289,300,O,306,HZ2,300-O-306-HZ2
1290,300,O,303,HG,300-O-303-HG
1291,300,O,292,HZ3,300-O-292-HZ3
1292,301,O,306,HZ2,301-O-306-HZ2
1293,301,OE1,299,HD22,301-OE1-299-HD22
1294,301,OE1,306,HZ3,301-OE1-306-HZ3
1295,301,OE1,119,HE22,301-OE1-119-HE22
1296,301,O,306,HZ3,301-O-306-HZ3
1297,301,OE1,306,HZ2,301-OE1-306-HZ2
1298,301,OE1,301,H,301-OE1-301-H
1299,301,O,301,HE21,301-O-301-HE21
1300,301,OE1,297,HD22,301-OE1-297-HD22
1301,301,OE1,299,HD21,301-OE1-299-HD21
1302,301,O,306,HZ1,301-O-306-HZ1
1303,301,O,303,H,301-O-303-H
1304,301,O,303,HG,301-O-303-HG
1305,301,OE1,300,H,301-OE1-300-H
1306,302,O,292,HZ1,302-O-292-HZ1
1307,302,O,292,HZ2,302-O-292-HZ2
1308,302,O,292,HZ3,302-O-292-HZ3
1309,302,O,288,HE2,302-O-288-HE2
1310,303,O,288,HE2,303-O-288-HE2
1311,303,O,306,H,303-O-306-H
1312,303,OG,306,HZ2,303-OG-306-HZ2
1313,303,OG,306,HZ3,303-OG-306-HZ3
1314,303,OG,306,HZ1,303-OG-306-HZ1
1315,303,O,307,H,303-O-307-H
1316,303,OG,306,H,303-OG-306-H

1317,303,OG,305,H,303-OG-305-H

1318,303,OG,292,HZ2,303-OG-292-HZ2

1319,304,OD2,293,HE22,304-OD2-293-HE22

1320,304,OD1,270,HH,304-OD1-270-HH

1321,304,O,307,H,304-O-307-H

1322,304,OD2,290,HE22,304-OD2-290-HE22

1323,304,OD2,292,HZ2,304-OD2-292-HZ2

1324,304,OD1,293,HE22,304-OD1-293-HE22

1325,304,OD1,305,H,304-OD1-305-H

1326,304,OD1,293,HE21,304-OD1-293-HE21

1327,304,OD1,304,H,304-OD1-304-H

1328,304,OD2,305,H,304-OD2-305-H

1329,304,OD2,292,HZ3,304-OD2-292-HZ3

1330,304,OD1,292,HZ3,304-OD1-292-HZ3

1331,304,OD2,292,HZ1,304-OD2-292-HZ1

1332,304,OD1,292,HZ1,304-OD1-292-HZ1

1333,304,OD2,304,H,304-OD2-304-H

1334,304,OD2,270,HH,304-OD2-270-HH

1335,304,O,308,H,304-O-308-H

1336,304,OD2,288,HE2,304-OD2-288-HE2

1337,304,OD1,292,HZ2,304-OD1-292-HZ2

1338,305,OE2,259,HH,305-OE2-259-HH

1339,305,OE1,267,HZ3,305-OE1-267-HZ3

1340,305,OE2,267,HZ1,305-OE2-267-HZ1

1341,305,OE2,306,HZ1,305-OE2-306-HZ1

1342,305,OE2,309,HD21,305-OE2-309-HD21

1343,305,OE1,263,HE22,305-OE1-263-HE22

1344,305,OE1,306,HZ1,305-OE1-306-HZ1

1345,305,OE2,267,HZ2,305-OE2-267-HZ2

1346,305,OE2,119,HE21,305-OE2-119-HE21

1347,305,OE1,306,HZ2,305-OE1-306-HZ2

1348,305,OE2,306,HZ2,305-OE2-306-HZ2

1349,305,OE1,303,HG,305-OE1-303-HG

1350,305,OE2,263,HE22,305-OE2-263-HE22

1351,305,OE1,306,H,305-OE1-306-H

1352,305,OE2,117,HD21,305-OE2-117-HD21

1353,305,O,309,HD21,305-O-309-HD21

1354,305,O,309,HD22,305-O-309-HD22

1355,305,OE2,267,HZ3,305-OE2-267-HZ3

1356,305,OE1,309,HD21,305-OE1-309-HD21

1357,305,OE1,267,HZ1,305-OE1-267-HZ1

1358,305,OE1,267,HZ2,305-OE1-267-HZ2

1359,305,O,309,H,305-O-309-H

1360,305,OE2,306,HZ3,305-OE2-306-HZ3

1361,305,OE1,306,HZ3,305-OE1-306-HZ3

1362,305,OE2,305,H,305-OE2-305-H

1363,305,O,307,H,305-O-307-H

1364,305,O,117,HD22,305-O-117-HD22

1365,305,OE1,259,HH,305-OE1-259-HH

1366,305,OE1,119,HE22,305-OE1-119-HE22

1367,305,OE2,119,HE22,305-OE2-119-HE22

1368,305,OE1,117,HD21,305-OE1-117-HD21

1369,305,OE2,263,HE21,305-OE2-263-HE21

1370,305,OE1,117,HD22,305-OE1-117-HD22

1371,305,OE1,119,HE21,305-OE1-119-HE21

1372,305,OE2,117,HD22,305-OE2-117-HD22

1373,305,OE1,305,H,305-OE1-305-H

1374,305,OE2,303,HG,305-OE2-303-HG

1375,306,O,309,H,306-O-309-H

1376,306,O,134,HH21,306-O-134-HH21

1377,306,O,119,HE21,306-O-119-HE21

1378,306,O,134,HE,306-O-134-HE

1379,307,O,310,H,307-O-310-H

1380,308,O,311,H,308-O-311-H

1381,309,OD1,134,HE,309-OD1-134-HE

1382,309,O,313,H,309-O-313-H

1383,309,OD1,263,HE22,309-OD1-263-HE22

1384,309,O,312,H,309-O-312-H

1385,309,OD1,134,HH21,309-OD1-134-HH21

1386,309,OD1,134,HH22,309-OD1-134-HH22

1387,309,ND2,134,HH21,309-ND2-134-HH21

1388,309,OD1,306,HZ1,309-OD1-306-HZ1

1389,309,OD1,119,HE22,309-OD1-119-HE22

1390,309,O,309,HD22,309-O-309-HD22

1391,309,OD1,116,HG,309-OD1-116-HG

1392,309,OD1,119,HE21,309-OD1-119-HE21

1393,309,OD1,117,HD21,309-OD1-117-HD21

1394,309,OD1,259,HH,309-OD1-259-HH

1395,309,OD1,263,HE21,309-OD1-263-HE21

1396,310,O,313,H,310-O-313-H

1397,310,O,314,H,310-O-314-H

1398,311,O,315,H,311-O-315-H

1399,311,O,314,H,311-O-314-H

1400,312,O,316,H,312-O-316-H

1401,312,O,516,H17,312-O-516-H17

1402,313,O,317,H,313-O-317-H

1403,313,OD1,134,HH22,313-OD1-134-HH22

1404,313,OD1,134,HH21,313-OD1-134-HH21

1405,313,O,316,H,313-O-316-H

1406,313,OD2,134,HH12,313-OD2-134-HH12

1407,313,OD1,134,HH12,313-OD1-134-HH12

1408,313,OD1,116,HG,313-OD1-116-HG

1409,313,OD1,309,HD22,313-OD1-309-HD22

1410,313,OD1,120,HG,313-OD1-120-HG

1411,313,OD1,121,H,313-OD1-121-H

1412,313,OD2,120,HG,313-OD2-120-HG

1413,313,OD2,309,HD21,313-OD2-309-HD21

1414,313,OD2,116,HG,313-OD2-116-HG

1415,313,OD1,122,H,313-OD1-122-H

1416,313,OD2,309,HD22,313-OD2-309-HD22

1417,313,OD1,309,HD21,313-OD1-309-HD21

1418,313,OD2,122,H,313-OD2-122-H

1419,313,OD2,121,H,313-OD2-121-H

1420,313,OD2,134,HE,313-OD2-134-HE

1421,313,OD1,134,HE,313-OD1-134-HE

1422,313,OD2,134,HH22,313-OD2-134-HH22

1423,313,OD2,134,HH21,313-OD2-134-HH21

1424,314,O,318,H,314-O-318-H

1425,314,O,317,H,314-O-317-H

1426,315,O,319,H,315-O-319-H

1427,315,O,318,H,315-O-318-H

1428,315,O,317,H,315-O-317-H

1429,316,O,516,H17,316-O-516-H17

1430,317,O,321,HG1,317-O-321-HG1

1431,317,O,319,H,317-O-319-H

1432,318,O,321,H,318-O-321-H

1433,318,O,322,H,318-O-322-H

1434,318,O,321,HG1,318-O-321-HG1

1435,319,O,323,H,319-O-323-H

1436,319,O,323,HG1,319-O-323-HG1

1437,320,O,324,H,320-O-324-H

1438,320,OD1,187,HH,320-OD1-187-HH

1439,320,OD2,499,HZ3,320-OD2-499-HZ3

1440,320,OD2,499,HZ2,320-OD2-499-HZ2

1441,320,O,324,HG1,320-O-324-HG1

1442,320,OD1,499,HZ3,320-OD1-499-HZ3

1443,320,OD2,499,HZ1,320-OD2-499-HZ1

1444,320,OD1,499,HZ2,320-OD1-499-HZ2

1445,320,OD2,187,HH,320-OD2-187-HH

1446,320,OD1,499,HZ1,320-OD1-499-HZ1

1447,320,OD2,516,H10,320-OD2-516-H10

1448,320,OD1,222,HD22,320-OD1-222-HD22

1449,320,OD1,497,HG1,320-OD1-497-HG1

1450,320,OD2,497,HG1,320-OD2-497-HG1

1451,321,O,325,H,321-O-325-H

1452,321,O,324,H,321-O-324-H

1453,322,O,325,H,322-O-325-H

1454,322,O,326,H,322-O-326-H

1455,323,O,326,H,323-O-326-H

1456,323,O,327,HG,323-O-327-HG

1457,323,O,327,H,323-O-327-H

1458,324,O,328,H,324-O-328-H

1459,324,OG1,499,HZ2,324-OG1-499-HZ2

1460,324,OG1,499,HZ3,324-OG1-499-HZ3

1461,324,O,327,HG,324-O-327-HG

1462,324,O,327,H,324-O-327-H

1463,324,OG1,499,HZ1,324-OG1-499-HZ1

1464,325,O,329,HG,325-O-329-HG

1465,325,O,329,H,325-O-329-H

1466,326,O,330,H,326-O-330-H

1467,326,O,329,HG,326-O-329-HG

1468,326,O,329,H,326-O-329-H

1469,327,O,327,HG,327-O-327-HG

1470,327,O,331,H,327-O-331-H

1471,328,O,331,H,328-O-331-H

1472,328,O,332,H,328-O-332-H

1473,329,O,333,H,329-O-333-H

1474,330,O,334,H,330-O-334-H

1475,330,O,333,H,330-O-333-H

1476,331,O,334,H,331-O-334-H

1477,331,O,335,H,331-O-335-H

1478,332,O,336,HD22,332-O-336-HD22

1479,332,O,335,H,332-O-335-H

1480,332,O,336,H,332-O-336-H

1481,333,O,336,H,333-O-336-H

1482,333,O,340,HE21,333-O-340-HE21

1483,335,O,336,HD22,335-O-336-HD22

1484,336,OD1,339,H,336-OD1-339-H

1485,336,OD1,338,HE,336-OD1-338-HE

1486,336,O,339,H,336-O-339-H

1487,336,OD1,338,H,336-OD1-338-H

1488,336,O,340,H,336-O-340-H

1489,337,O,341,H,337-O-341-H

1490,337,O,340,H,337-O-340-H

1491,338,O,338,HH11,338-O-338-HH11

1492,338,O,342,H,338-O-342-H

1493,339,O,343,H,339-O-343-H

1494,339,O,342,H,339-O-342-H

1495,340,O,344,H,340-O-344-H

1496,340,OE1,344,HE22,340-OE1-344-HE22

1497,340,OE1,480,H,340-OE1-480-H

1498,340,O,343,H,340-O-343-H

1499,341,O,345,H,341-O-345-H

1500,341,O,341,HH11,341-O-341-HH11

1501,342,O,345,H,342-O-345-H

1502,342,O,346,H,342-O-346-H

1503,342,O,342,HZ1,342-O-342-HZ1

1504,343,O,346,H,343-O-346-H

1505,343,O,347,H,343-O-347-H

1506,344,O,511,HH12,344-O-511-HH12

1507,344,OE1,341,HH11,344-OE1-341-HH11

1508,344,OE1,341,HE,344-OE1-341-HE

1509,344,O,348,H,344-O-348-H

1510,344,O,511,HH22,344-O-511-HH22

1511,344,OE1,341,HH21,344-OE1-341-HH21

1512,345,OE2,338,HH12,345-OE2-338-HH12

1513,345,OE1,338,HH22,345-OE1-338-HH22

1514,345,OE2,338,HH22,345-OE2-338-HH22

1515,345,OE1,338,HH12,345-OE1-338-HH12

1516,345,O,349,HG1,345-O-349-HG1

1517,345,OE1,342,HZ1,345-OE1-342-HZ1

1518,345,OE2,341,HH11,345-OE2-341-HH11

1519,345,OE2,341,HE,345-OE2-341-HE

1520,345,O,349,H,345-O-349-H

1521,345,OE2,342,HZ3,345-OE2-342-HZ3

1522,345,OE2,342,HZ1,345-OE2-342-HZ1

1523,345,OE2,342,HZ2,345-OE2-342-HZ2

1524,345,OE1,341,HH12,345-OE1-341-HH12

1525,345,OE2,341,HH22,345-OE2-341-HH22

1526,345,OE1,341,HH22,345-OE1-341-HH22

1527,345,OE2,341,HH12,345-OE2-341-HH12

1528,345,OE1,342,HZ3,345-OE1-342-HZ3

1529,345,OE2,341,HH21,345-OE2-341-HH21

1530,345,OE1,342,HZ2,345-OE1-342-HZ2

1531,346,OE2,342,HZ3,346-OE2-342-HZ3

1532,346,OE1,342,HZ3,346-OE1-342-HZ3

1533,346,OE2,342,HZ1,346-OE2-342-HZ1

1534,346,O,350,H,346-O-350-H

1535,346,OE1,367,H,346-OE1-367-H

1536,346,OE1,368,H,346-OE1-368-H

1537,346,O,349,H,346-O-349-H

1538,346,OE1,342,HZ1,346-OE1-342-HZ1

1539,346,OE1,342,HZ2,346-OE1-342-HZ2

1540,346,OE2,367,H,346-OE2-367-H

1541,346,OE2,342,HZ2,346-OE2-342-HZ2

1542,347,O,350,H,347-O-350-H

1543,347,O,352,H,347-O-352-H

1544,347,O,351,H,347-O-351-H

1545,348,OD1,353,HE,348-OD1-353-HE

1546,348,OD2,511,HH22,348-OD2-511-HH22

1547,348,OD1,511,HH12,348-OD1-511-HH12

1548,348,OD2,511,HH12,348-OD2-511-HH12

1549,348,OD1,353,HH21,348-OD1-353-HH21

1550,348,OD1,511,HH22,348-OD1-511-HH22

1551,348,OD2,353,HE,348-OD2-353-HE

1552,348,O,352,H,348-O-352-H

1553,348,OD2,353,HH21,348-OD2-353-HH21

1554,350,O,355,HH21,350-O-355-HH21

1555,350,O,355,HH22,350-O-355-HH22

1556,351,O,355,HH11,351-O-355-HH11

1557,351,O,355,HH21,351-O-355-HH21

1558,351,O,354,HG,351-O-354-HG

1559,351,O,355,HE,351-O-355-HE

1560,352,O,355,H,352-O-355-H

1561,352,O,476,HE21,352-O-476-HE21

1562,352,O,354,H,352-O-354-H

1563,353,O,477,HH21,353-O-477-HH21

1564,353,O,477,HH22,353-O-477-HH22

1565,353,O,476,HE21,353-O-476-HE21

1566,353,O,477,HH12,353-O-477-HH12

1567,354,OG,355,H,354-OG-355-H

1568,355,O,476,HE22,355-O-476-HE22

1569,355,O,476,HE21,355-O-476-HE21

1570,356,O,355,HE,356-O-355-HE

1571,356,O,355,HH11,356-O-355-HH11

1572,356,O,355,HH21,356-O-355-HH21

1573,357,O,356,HH11,357-O-356-HH11

1574,358,NH2,360,HG,358-NH2-360-HG

1575,358,O,361,H,358-O-361-H

1576,359,O,362,HH11,359-O-362-HH11

1577,359,O,362,H,359-O-362-H

1578,360,OG,358,HE,360-OG-358-HE

1579,360,O,363,HG,360-O-363-HG

1580,360,O,360,HG,360-O-360-HG

1581,360,O,363,H,360-O-363-H

1582,361,OD2,358,HH21,361-OD2-358-HH21

1583,361,OD1,360,HG,361-OD1-360-HG

1584,361,OD1,364,HE2,361-OD1-364-HE2

1585,361,OD2,355,HE,361-OD2-355-HE

1586,361,OD1,355,HH12,361-OD1-355-HH12

1587,361,OD1,358,HH21,361-OD1-358-HH21

1588,361,OD2,355,HH22,361-OD2-355-HH22

1589,361,O,364,H,361-O-364-H

1590,361,OD2,358,HE,361-OD2-358-HE

1591,361,O,365,H,361-O-365-H

1592,361,OD2,358,H,361-OD2-358-H

1593,361,OD1,355,HH22,361-OD1-355-HH22

1594,361,OD2,355,HH12,361-OD2-355-HH12

1595,361,OD1,358,HE,361-OD1-358-HE

1596,361,OD2,355,HH21,361-OD2-355-HH21

1597,361,OD1,358,HH11,361-OD1-358-HH11

1598,362,O,365,H,362-O-365-H

1599,362,NH1,465,HE1,362-NH1-465-HE1

1600,364,ND1,363,HG,364-ND1-363-HG

1601,364,ND1,364,H,364-ND1-364-H

1602,365,O,369,H,365-O-369-H

1603,365,O,368,H,365-O-368-H

1604,366,O,369,H,366-O-369-H

1605,366,O,370,H,366-O-370-H

1606,367,O,371,H,367-O-371-H

1607,367,OH,342,HZ3,367-OH-342-HZ3

1608,368,O,372,H,368-O-372-H

1609,369,OE1,362,HH21,369-OE1-362-HH21

1610,369,OE2,362,HE,369-OE2-362-HE

1611,369,OE1,464,HH21,369-OE1-464-HH21

1612,369,OE2,464,HH21,369-OE2-464-HH21

1613,369,OE2,362,HH21,369-OE2-362-HH21

1614,369,OE2,441,HZ2,369-OE2-441-HZ2

1615,369,OE1,362,HH22,369-OE1-362-HH22

1616,369,OE1,362,HH12,369-OE1-362-HH12

1617,369,OE2,362,HH22,369-OE2-362-HH22

1618,369,OE1,464,HE,369-OE1-464-HE

1619,369,O,372,H,369-O-372-H

1620,369,OE2,464,HE,369-OE2-464-HE

1621,369,OE1,362,HE,369-OE1-362-HE

1622,369,O,373,H,369-O-373-H

1623,370,O,374,H,370-O-374-H

1624,371,O,374,H,371-O-374-H

1625,371,O,375,H,371-O-375-H

1626,371,O,375,HG1,371-O-375-HG1

1627,372,O,376,H,372-O-376-H

1628,373,O,377,H,373-O-377-H

1629,374,OE1,377,HE,374-OE1-377-HE

1630,374,OE2,431,HH22,374-OE2-431-HH22

1631,374,OE2,431,HH12,374-OE2-431-HH12

1632,374,OE2,377,HE,374-OE2-377-HE

1633,374,OE2,377,HH21,374-OE2-377-HH21

1634,374,O,378,H,374-O-378-H

1635,374,OE1,431,HH11,374-OE1-431-HH11

1636,374,OE1,431,HH12,374-OE1-431-HH12

1637,374,OE1,431,HH22,374-OE1-431-HH22

1638,374,OE1,377,HH21,374-OE1-377-HH21

1639,374,OE2,431,HH11,374-OE2-431-HH11

1640,374,OE2,428,H,374-OE2-428-H

1641,375,O,379,HG,375-O-379-HG

1642,375,O,379,H,375-O-379-H

1643,375,OG1,329,HG,375-OG1-329-HG

1644,376,O,380,H,376-O-380-H

1645,377,O,416,HE2,377-O-416-HE2

1646,377,O,380,HG,377-O-380-HG

1647,378,ND1,332,HH,378-ND1-332-HH

1648,378,O,416,HE2,378-O-416-HE2

1649,379,OG,381,H,379-OG-381-H

1650,379,O,412,HE1,379-O-412-HE1

1651,380,O,382,H,380-O-382-H

1652,380,OG,415,HD22,380-OG-415-HD22

1653,380,OG,416,HE2,380-OG-416-HE2

1654,381,O,498,H,381-O-498-H

1655,382,O,411,HE21,382-O-411-HE21

1656,383,O,411,H,383-O-411-H

1657,383,O,412,H,383-O-412-H

1658,384,O,411,HE21,384-O-411-HE21

1659,385,O,385,HG1,385-O-385-HG1

1660,385,O,230,HG,385-O-230-HG

1661,385,OG1,409,H,385-OG1-409-H

1662,386,O,388,HD1,386-O-388-HD1

1663,386,O,106,HH12,386-O-106-HH12

1664,386,O,385,HG1,386-O-385-HG1

1665,387,O,106,HH11,387-O-106-HH11

1666,388,O,389,HG,388-O-389-HG

1667,388,O,407,H,388-O-407-H

1668,388,NE2,385,HG1,388-NE2-385-HG1

1669,389,O,105,H,389-O-105-H

1670,390,OG1,405,H,390-OG1-405-H

1671,390,OG1,404,H,390-OG1-404-H

1672,390,O,404,H,390-O-404-H

1673,390,O,403,HZ3,390-O-403-HZ3

1674,391,O,403,HZ3,391-O-403-HZ3

1675,391,OG1,104,HZ1,391-OG1-104-HZ1

1676,391,OG1,104,HZ3,391-OG1-104-HZ3

1677,391,O,403,HZ2,391-O-403-HZ2

1678,391,OG1,392,HE,391-OG1-392-HE

1679,391,O,403,HZ1,391-O-403-HZ1

1680,391,OG1,104,HZ2,391-OG1-104-HZ2

1681,391,OG1,104,H,391-OG1-104-H

1682,391,OG1,105,H,391-OG1-105-H

1683,393,OD1,393,H,393-OD1-393-H

1684,393,OD1,403,HZ1,393-OD1-403-HZ1

1685,393,OD2,403,H,393-OD2-403-H

1686,393,OD1,403,HZ3,393-OD1-403-HZ3

1687,393,OD2,400,HH,393-OD2-400-HH

1688,393,OD1,403,H,393-OD1-403-H

1689,393,OD1,403,HZ2,393-OD1-403-HZ2

1690,393,OD2,403,HZ1,393-OD2-403-HZ1

1691,393,OD2,393,H,393-OD2-393-H

1692,393,OD1,400,HH,393-OD1-400-HH

1693,393,OD2,403,HZ3,393-OD2-403-HZ3

1694,393,OD2,403,HZ2,393-OD2-403-HZ2

1695,394,OG1,99,HE21,394-OG1-99-HE21

1696,394,OG1,395,H,394-OG1-395-H

1697,394,O,401,H,394-O-401-H

1698,394,O,395,HG,394-O-395-HG

1699,395,O,94,HE21,395-O-94-HE21

1700,395,O,99,HE21,395-O-99-HE21

1701,395,O,99,HE22,395-O-99-HE22

1702,396,O,399,H,396-O-399-H

1703,397,O,38,HZ3,397-O-38-HZ3

1704,397,O,399,H,397-O-399-H

1705,397,O,38,HZ1,397-O-38-HZ1

1706,398,O,38,HZ1,398-O-38-HZ1

1707,398,O,38,H,398-O-38-H

1708,398,O,38,HZ2,398-O-38-HZ2

1709,398,O,38,HZ3,398-O-38-HZ3

1710,399,O,396,H,399-O-396-H

1711,399,O,395,HG,399-O-395-HG

1712,400,O,39,HD22,400-O-39-HD22

1713,401,O,394,H,401-O-394-H

1714,402,O,390,HG1,402-O-390-HG1

1715,402,O,405,H,402-O-405-H

1716,403,O,405,HH11,403-O-405-HH11

1717,404,O,405,HH11,404-O-405-HH11

1718,405,O,390,H,405-O-390-H

1719,405,O,389,HG,405-O-389-HG

1720,405,O,390,HG1,405-O-390-HG1

1721,405,NH2,75,HE22,405-NH2-75-HE22

1722,406,O,84,H,406-O-84-H

1723,407,O,388,H,407-O-388-H

1724,408,O,86,H,408-O-86-H

1725,409,O,411,H,409-O-411-H

1726,409,O,385,H,409-O-385-H

1727,410,OD1,412,H,410-OD1-412-H

1728,410,OD1,413,H,410-OD1-413-H

1729,410,O,413,H,410-O-413-H

1730,410,O,414,H,410-O-414-H

1731,411,O,415,HD22,411-O-415-HD22

1732,411,OE1,451,H,411-OE1-451-H

1733,411,O,415,H,411-O-415-H

1734,411,O,414,H,411-O-414-H

1735,412,O,415,H,412-O-415-H

1736,412,O,416,H,412-O-416-H

1737,413,OE1,68,HE21,413-OE1-68-HE21

1738,413,OE1,410,HD22,413-OE1-410-HD22

1739,413,NE2,68,HE22,413-NE2-68-HE22

1740,413,OE1,64,HG,413-OE1-64-HG

1741,413,O,417,H,413-O-417-H

1742,413,O,413,HE21,413-O-413-HE21

1743,413,O,416,H,413-O-416-H

1744,413,NE2,87,HG,413-NE2-87-HG

1745,413,OE1,68,HE22,413-OE1-68-HE22

1746,413,OE1,87,HG,413-OE1-87-HG

1747,413,OE1,67,HG,413-OE1-67-HG

1748,414,O,421,HE1,414-O-421-HE1

1749,414,O,417,H,414-O-417-H

1750,415,OD1,448,H,415-OD1-448-H

1751,415,O,377,HH11,415-O-377-HH11

1752,416,ND1,377,HH12,416-ND1-377-HH12

1753,416,O,418,HE21,416-O-418-HE21

1754,417,OD2,419,H,417-OD2-419-H

1755,417,O,419,H,417-O-419-H

1756,417,OD1,419,HZ3,417-OD1-419-HZ3

1757,417,OD2,419,HZ1,417-OD2-419-HZ1

1758,417,OD1,418,HE21,417-OD1-418-HE21

1759,417,OD2,419,HZ2,417-OD2-419-HZ2

1760,417,OD2,420,H,417-OD2-420-H

1761,417,OD1,419,H,417-OD1-419-H

1762,417,OD2,419,HZ3,417-OD2-419-HZ3

1763,417,OD1,419,HZ2,417-OD1-419-HZ2

1764,417,O,420,H,417-O-420-H

1765,417,OD2,68,HE22,417-OD2-68-HE22

1766,417,O,421,H,417-O-421-H

1767,417,OD2,413,HE22,417-OD2-413-HE22

1768,417,OD1,419,HZ1,417-OD1-419-HZ1

1769,417,OD1,68,HE22,417-OD1-68-HE22

1770,417,OD1,420,H,417-OD1-420-H

1771,417,OD1,413,HE21,417-OD1-413-HE21

1772,417,OD2,413,HE21,417-OD2-413-HE21

1773,418,OE1,68,HE22,418-OE1-68-HE22

1774,418,OE1,418,H,418-OE1-418-H

1775,418,OE1,68,HE21,418-OE1-68-HE21

1776,418,OE1,423,H,418-OE1-423-H

1777,418,NE2,418,H,418-NE2-418-H

1778,418,O,418,HE21,418-O-418-HE21

1779,420,O,446,HZ1,420-O-446-HZ1

1780,420,O,446,HZ2,420-O-446-HZ2

1781,420,O,446,HZ3,420-O-446-HZ3

1782,422,O,431,HH21,422-O-431-HH21

1783,422,O,431,HE,422-O-431-HE

1784,423,OD1,425,HG,423-OD1-425-HG

1785,423,O,426,H,423-O-426-H

1786,423,OD1,425,H,423-OD1-425-H

1787,423,O,431,HH21,423-O-431-HH21

1788,423,O,423,HD22,423-O-423-HD22

1789,423,O,431,HH22,423-O-431-HH22

1790,423,N,423,HD22,423-N-423-HD22

1791,423,OD1,426,H,423-OD1-426-H

1792,424,O,377,HH22,424-O-377-HH22

1793,424,O,377,HH12,424-O-377-HH12

1794,425,O,425,HG,425-O-425-HG

1795,425,O,378,HE2,425-O-378-HE2

1796,426,OE2,423,HD22,426-OE2-423-HD22

1797,426,OE1,423,HD22,426-OE1-423-HD22

1798,426,O,431,HH22,426-O-431-HH22

1799,426,O,431,HH12,426-O-431-HH12

1800,426,OE1,427,H,426-OE1-427-H

1801,426,OE1,425,HG,426-OE1-425-HG

1802,426,OE2,425,HG,426-OE2-425-HG

1803,426,OE2,427,H,426-OE2-427-H

1804,426,O,428,H,426-O-428-H

1805,427,O,338,HH21,427-O-338-HH21

1806,428,O,431,H,428-O-431-H

1807,429,O,432,H,429-O-432-H

1808,429,O,433,H,429-O-433-H

1809,430,OE1,342,HZ3,430-OE1-342-HZ3

1810,430,OE1,338,HH22,430-OE1-338-HH22

1811,430,OE2,342,HZ3,430-OE2-342-HZ3

1812,430,OE2,338,HH12,430-OE2-338-HH12

1813,430,OE2,430,H,430-OE2-430-H

1814,430,OE1,367,HH,430-OE1-367-HH

1815,430,OE2,342,HZ1,430-OE2-342-HZ1

1816,430,OE2,342,HZ2,430-OE2-342-HZ2

1817,430,OE1,342,HZ2,430-OE1-342-HZ2

1818,430,OE2,367,HH,430-OE2-367-HH

1819,430,O,433,H,430-O-433-H

1820,430,OE1,430,H,430-OE1-430-H

1821,430,OE1,338,HH12,430-OE1-338-HH12

1822,430,OE2,338,HH22,430-OE2-338-HH22

1823,430,OE1,342,HZ1,430-OE1-342-HZ1

1824,432,O,440,H,432-O-440-H

1825,434,O,437,H,434-O-437-H

1826,434,OG1,437,H,434-OG1-437-H

1827,434,OG1,438,H,434-OG1-438-H

1828,434,OG1,436,H,434-OG1-436-H

1829,435,O,437,H,435-O-437-H

1830,436,O,438,H,436-O-438-H

1831,436,OD2,434,HG1,436-OD2-434-HG1

1832,436,OD1,436,H,436-OD1-436-H

1833,436,OD1,438,H,436-OD1-438-H

1834,436,OD2,438,H,436-OD2-438-H

1835,436,OD2,436,H,436-OD2-436-H

1836,436,OD1,434,HG1,436-OD1-434-HG1

1837,438,O,434,HG1,438-O-434-HG1

1838,438,O,434,H,438-O-434-H

1839,439,O,441,HZ1,439-O-441-HZ1

1840,439,O,441,HZ2,439-O-441-HZ2

1841,439,O,441,H,439-O-441-H

1842,439,O,441,HZ3,439-O-441-HZ3

1843,440,OD2,443,H,440-OD2-443-H

1844,440,O,444,HG,440-O-444-HG

1845,440,OD1,442,H,440-OD1-442-H

1846,440,OD1,443,H,440-OD1-443-H

1847,440,O,443,H,440-O-443-H

1848,440,OD2,441,H,440-OD2-441-H

1849,440,O,444,H,440-O-444-H

1850,440,O,442,H,440-O-442-H

1851,440,OD2,442,H,440-OD2-442-H

1852,441,O,444,HG,441-O-444-HG

1853,441,O,445,H,441-O-445-H

1854,442,O,446,HZ3,442-O-446-HZ3

1855,442,O,446,H,442-O-446-H

1856,442,O,445,H,442-O-445-H

1857,442,O,446,HZ2,442-O-446-HZ2

1858,442,O,446,HZ1,442-O-446-HZ1

1859,443,O,447,H,443-O-447-H

1860,443,O,446,H,443-O-446-H

1861,444,OG,464,HH22,444-OG-464-HH22

1862,444,O,464,HH22,444-O-464-HH22

1863,444,O,447,H,444-O-447-H

1864,444,OG,445,H,444-OG-445-H

1865,445,OE1,464,HH12,445-OE1-464-HH12

1866,445,OE2,441,HZ3,445-OE2-441-HZ3

1867,445,OE1,446,HZ3,445-OE1-446-HZ3

1868,445,OE2,93,HH21,445-OE2-93-HH21

1869,445,OE1,444,HG,445-OE1-444-HG

1870,445,OE1,456,HZ3,445-OE1-456-HZ3

1871,445,OE2,93,HH22,445-OE2-93-HH22

1872,445,OE1,441,HZ3,445-OE1-441-HZ3

1873,445,OE1,93,HH12,445-OE1-93-HH12

1874,445,OE1,464,HH22,445-OE1-464-HH22

1875,445,OE2,464,HH12,445-OE2-464-HH12

1876,445,OE1,441,HZ2,445-OE1-441-HZ2

1877,445,O,93,HH21,445-O-93-HH21

1878,445,OE1,93,HH22,445-OE1-93-HH22

1879,445,OE1,441,HZ1,445-OE1-441-HZ1

1880,445,OE2,464,HH22,445-OE2-464-HH22

1881,445,OE2,441,HZ2,445-OE2-441-HZ2

1882,445,O,447,H,445-O-447-H

1883,445,OE1,93,HH21,445-OE1-93-HH21

1884,445,OE2,444,HG,445-OE2-444-HG

1885,445,OE2,441,HZ1,445-OE2-441-HZ1

1886,445,OE2,93,HH12,445-OE2-93-HH12

1887,445,O,93,HH22,445-O-93-HH22

1888,446,O,448,H,446-O-448-H

1889,448,O,415,HD21,448-O-415-HD21

1890,449,O,411,HE22,449-O-411-HE22

1891,450,O,457,H,450-O-457-H

1892,451,O,456,HZ1,451-O-456-HZ1

1893,451,O,456,HZ3,451-O-456-HZ3

1894,451,O,456,HZ2,451-O-456-HZ2

1895,452,O,456,H,452-O-456-H

1896,452,O,455,H,452-O-455-H

1897,453,O,455,H,453-O-455-H

1898,453,O,456,HZ2,453-O-456-HZ2

1899,453,O,456,H,453-O-456-H

1900,454,O,135,HH12,454-O-135-HH12

1901,454,O,456,H,454-O-456-H

1902,454,O,135,HH22,454-O-135-HH22

1903,455,NH1,131,HE1,455-NH1-131-HE1

1904,455,O,135,HH12,455-O-135-HH12

1905,455,O,452,H,455-O-452-H

1906,456,O,135,HH22,456-O-135-HH22

1907,456,O,135,HH12,456-O-135-HH12

1908,457,O,460,H,457-O-460-H

1909,457,O,456,HZ2,457-O-456-HZ2

1910,457,O,456,HZ1,457-O-456-HZ1

1911,457,O,135,HH22,457-O-135-HH22

1912,458,O,461,H,458-O-461-H

1913,458,O,139,HE22,458-O-139-HE22

1914,458,O,461,HG1,458-O-461-HG1

1915,458,O,139,HE21,458-O-139-HE21

1916,458,O,462,H,458-O-462-H

1917,459,O,463,H,459-O-463-H

1918,459,O,462,H,459-O-462-H

1919,459,O,464,H,459-O-464-H

1920,460,OE1,456,HZ3,460-OE1-456-HZ3

1921,460,OE2,461,HG1,460-OE2-461-HG1

1922,460,OE2,456,HZ2,460-OE2-456-HZ2

1923,460,OE2,464,HH22,460-OE2-464-HH22

1924,460,O,464,HH11,460-O-464-HH11

1925,460,O,464,H,460-O-464-H

1926,460,OE1,461,H,460-OE1-461-H

1927,460,OE2,464,HH11,460-OE2-464-HH11

1928,460,OE2,464,HH12,460-OE2-464-HH12

1929,460,OE2,461,H,460-OE2-461-H

1930,460,OE1,464,HH11,460-OE1-464-HH11

1931,460,OE2,139,HE22,460-OE2-139-HE22

1932,460,OE1,456,HZ2,460-OE1-456-HZ2

1933,460,OE1,456,HZ1,460-OE1-456-HZ1

1934,460,OE1,461,HG1,460-OE1-461-HG1

1935,460,OE2,456,HZ3,460-OE2-456-HZ3

1936,460,OE2,456,HZ1,460-OE2-456-HZ1

1937,461,O,464,H,461-O-464-H

1938,461,O,465,H,461-O-465-H

1939,461,O,461,HG1,461-O-461-HG1

1940,461,OG1,139,HE22,461-OG1-139-HE22

1941,461,OG1,139,HE21,461-OG1-139-HE21

1942,462,O,466,H,462-O-466-H

1943,462,O,465,H,462-O-465-H

1944,463,O,467,H,463-O-467-H

1945,464,O,362,HH11,464-O-362-HH11

1946,464,O,468,H,464-O-468-H

1947,464,O,362,HH21,464-O-362-HH21

1948,464,O,362,HE,464-O-362-HE

1949,465,O,468,H,465-O-468-H

1950,465,O,469,H,465-O-469-H

1951,465,NE1,362,HH12,465-NE1-362-HH12

1952,466,O,469,H,466-O-469-H

1953,466,O,470,H,466-O-470-H

1954,467,O,471,H,467-O-471-H

1955,468,O,472,H,468-O-472-H

1956,469,O,473,H,469-O-473-H

1957,469,O,472,H,469-O-472-H

1958,470,O,474,H,470-O-474-H

1959,471,O,475,H,471-O-475-H

1960,472,O,476,H,472-O-476-H

1961,473,O,476,H,473-O-476-H

1962,473,O,477,HE,473-O-477-HE

1963,473,O,477,HH11,473-O-477-HH11

1964,473,O,477,H,473-O-477-H

1965,473,O,477,HH21,473-O-477-HH21

1966,474,O,478,H,474-O-478-H

1967,474,O,477,H,474-O-477-H

1968,475,O,344,HE21,475-O-344-HE21

1969,475,O,511,HH21,475-O-511-HH21

1970,475,O,511,HE,475-O-511-HE

1971,476,O,476,HE21,476-O-476-HE21

1972,476,O,511,HH21,476-O-511-HH21

1973,476,O,511,HE,476-O-511-HE

1974,477,O,511,H,477-O-511-H

1975,478,O,344,HE22,478-O-344-HE22

1976,478,O,344,HE21,478-O-344-HE21

1977,479,OE1,512,H,479-OE1-512-H

1978,479,OE1,341,HH21,479-OE1-341-HH21

1979,479,OE1,512,HG,479-OE1-512-HG

1980,479,OE2,512,H,479-OE2-512-H

1981,479,OE1,341,HE,479-OE1-341-HE

1982,479,OE1,341,HH22,479-OE1-341-HH22

1983,479,OE2,341,HE,479-OE2-341-HE

1984,479,OE2,341,HH12,479-OE2-341-HH12

1985,479,OE1,344,HE22,479-OE1-344-HE22

1986,479,OE1,341,HH12,479-OE1-341-HH12

1987,479,OE2,509,HE21,479-OE2-509-HE21

1988,479,OE2,341,HH22,479-OE2-341-HH22

1989,479,OE2,512,HG,479-OE2-512-HG

1990,479,OE2,341,HH21,479-OE2-341-HH21

1991,479,O,509,H,479-O-509-H

1992,479,O,509,HE21,479-O-509-HE21

1993,479,OE1,509,HE21,479-OE1-509-HE21

1994,480,O,340,HE22,480-O-340-HE22

1995,481,OG,509,HE22,481-OG-509-HE22

1996,481,OG,482,H,481-OG-482-H

1997,481,O,507,H,481-O-507-H

1998,481,OG,507,HE21,481-OG-507-HE21

1999,482,O,485,H,482-O-485-H

2000,483,O,486,H,483-O-486-H

2001,483,O,485,H,483-O-485-H

2002,484,O,486,H,484-O-486-H

2003,485,O,487,HZ2,485-O-487-HZ2

2004,485,O,487,HZ1,485-O-487-HZ1

2005,485,O,487,H,485-O-487-H

2006,485,O,487,HZ3,485-O-487-HZ3

2007,486,O,488,H,486-O-488-H

2008,486,O,505,HE2,486-O-505-HE2

2009,487,O,489,H,487-O-489-H

2010,487,O,505,HE2,487-O-505-HE2

2011,488,O,487,HZ1,488-O-487-HZ1

2012,488,O,487,HZ3,488-O-487-HZ3

2013,489,OD2,491,HG1,489-OD2-491-HG1

2014,489,O,491,H,489-O-491-H

2015,489,OD2,490,H,489-OD2-490-H

2016,489,O,501,H,489-O-501-H

2017,489,OD2,487,HZ1,489-OD2-487-HZ1

2018,489,O,500,HD1,489-O-500-HD1

2019,489,OD1,487,HZ3,489-OD1-487-HZ3

2020,489,OD2,487,HZ3,489-OD2-487-HZ3

2021,489,OD1,491,HG1,489-OD1-491-HG1

2022,489,OD1,487,HZ2,489-OD1-487-HZ2

2023,489,OD1,491,H,489-OD1-491-H

2024,489,OD2,491,H,489-OD2-491-H

2025,490,O,501,H,490-O-501-H

2026,491,O,501,H,491-O-501-H

2027,492,O,57,HZ1,492-O-57-HZ1

2028,492,O,57,HZ3,492-O-57-HZ3

2029,492,O,58,HD21,492-O-58-HD21

2030,492,O,60,HE2,492-O-60-HE2

2031,492,O,57,HZ2,492-O-57-HZ2

2032,493,O,499,H,493-O-499-H

2033,494,OH,230,H,494-OH-230-H

2034,497,O,499,HZ3,497-O-499-HZ3

2035,497,OG1,499,HZ3,497-OG1-499-HZ3

2036,497,O,497,HG1,497-O-497-HG1

2037,497,O,499,HZ2,497-O-499-HZ2

2038,497,OG1,499,HZ2,497-OG1-499-HZ2

2039,497,O,499,HZ1,497-O-499-HZ1

2040,497,OG1,499,HZ1,497-OG1-499-HZ1

2041,497,O,499,H,497-O-499-H

2042,497,OG1,516,H10,497-OG1-516-H10

2043,498,O,328,HE1,498-O-328-HE1

2044,499,O,493,H,499-O-493-H

2045,500,O,500,HD1,500-O-500-HD1

2046,500,O,502,H,500-O-502-H

2047,500,NE2,327,HG,500-NE2-327-HG

2048,501,O,500,HD1,501-O-500-HD1

2049,501,O,503,H,501-O-503-H

2050,502,O,504,H,502-O-504-H

2051,502,O,185,HD21,502-O-185-HD21

2052,502,O,487,HZ3,502-O-487-HZ3

2053,503,O,506,H,503-O-506-H

2054,503,O,185,HD22,503-O-185-HD22

2055,503,O,505,H,503-O-505-H

2056,504,OE2,188,HE,504-OE2-188-HE

2057,504,OE1,504,H,504-OE1-504-H

2058,504,OE1,188,HH12,504-OE1-188-HH12

2059,504,OE2,188,HH22,504-OE2-188-HH22

2060,504,OE2,185,HD22,504-OE2-185-HD22

2061,504,OE2,188,HH21,504-OE2-188-HH21

2062,504,OE1,188,HE,504-OE1-188-HE

2063,504,OE1,183,HE2,504-OE1-183-HE2

2064,504,OE2,504,H,504-OE2-504-H

2065,504,OE1,185,HD22,504-OE1-185-HD22

2066,504,OE2,188,HH12,504-OE2-188-HH12

2067,504,OE1,188,HH22,504-OE1-188-HH22

2068,504,OE1,188,HH21,504-OE1-188-HH21

2069,504,OE1,505,HE2,504-OE1-505-HE2

2070,505,ND1,504,H,505-ND1-504-H

2071,505,ND1,505,H,505-ND1-505-H

2072,506,O,184,H,506-O-184-H

2073,507,OE1,481,HG,507-OE1-481-HG

2074,507,OE1,183,HE2,507-OE1-183-HE2

2075,507,O,481,H,507-O-481-H

2076,507,OE1,509,HE21,507-OE1-509-HE21

2077,508,O,509,HE21,508-O-509-HE21

2078,509,OE1,175,HE22,509-OE1-175-HE22

2079,509,O,511,H,509-O-511-H

2080,509,OE1,481,HG,509-OE1-481-HG

2081,509,O,479,H,509-O-479-H

2082,509,OE1,510,H,509-OE1-510-H

2083,510,O,509,HE21,510-O-509-HE21

2084,511,NH1,353,HH21,511-NH1-353-HH21

2085,511,O,353,HH12,511-O-353-HH12

2086,511,O,512,HG,511-O-512-HG

2087,512,O,512,HG,512-O-512-HG

2088,512,O,353,HH11,512-O-353-HH11

2089,512,OXT,511,HH11,512-OXT-511-HH11

2090,512,OXT,353,HH11,512-OXT-353-HH11

2091,512,O,353,HH12,512-O-353-HH12

2092,512,OXT,512,HG,512-OXT-512-HG

2093,512,OXT,353,HH22,512-OXT-353-HH22

2094,512,OXT,353,HH12,512-OXT-353-HH12

2095,512,O,353,HH22,512-O-353-HH22

2096,512,OG,341,HH22,512-OG-341-HH22

2097,515,O1D,455,HH11,515-O1D-455-HH11

2098,515,O2A,386,H,515-O2A-386-H

2099,515,O2D,135,HH12,515-O2D-135-HH12

2100,515,O2D,106,HH21,515-O2D-106-HH21

2101,515,O2D,135,HH22,515-O2D-135-HH22

2102,515,O1D,106,HE,515-O1D-106-HE

2103,515,O2D,456,H,515-O2D-456-H

2104,515,O1D,131,HE1,515-O1D-131-HE1

2105,515,O1D,122,HG,515-O1D-122-HG

2106,515,O1A,106,HH12,515-O1A-106-HH12

2107,515,O2D,131,HE1,515-O2D-131-HE1

2108,515,O1D,135,HH11,515-O1D-135-HH11

2109,515,O2A,106,HH12,515-O2A-106-HH12

2110,515,O1A,386,H,515-O1A-386-H

2111,515,O2A,106,HH22,515-O2A-106-HH22

2112,515,O2D,455,HH11,515-O2D-455-HH11

2113,515,O2D,122,HG,515-O2D-122-HG

2114,515,O1A,122,HG,515-O1A-122-HG

2115,515,O1D,106,HH21,515-O1D-106-HH21

2116,515,O1A,106,HH22,515-O1A-106-HH22

2117,515,O2D,135,HH11,515-O2D-135-HH11

2118,516,O15,116,H,516-O15-116-H

2119,516,O18,122,HG,516-O18-122-HG

2120,516,O15,222,HD22,516-O15-222-HD22

2121,516,O18,120,HG,516-O18-120-HG

Model IV

RES_DONOR,ATOM_DONOR,RES_ACCEPTOR,ATOM_ACCEPTOR,TAG

1,37,O,39,H,37-O-39-H

2,38,O,39,HD22,38-O-39-HD22

3,38,O,400,H,38-O-400-H

4,39,OD1,75,HE22,39-OD1-75-HE22

5,39,OD1,38,HZ2,39-OD1-38-HZ2

6,39,ND2,39,H,39-ND2-39-H

7,39,ND2,75,HE22,39-ND2-75-HE22

8,39,OD1,405,HH21,39-OD1-405-HH21

9,39,OD1,38,HZ1,39-OD1-38-HZ1

10,39,O,39,HD22,39-O-39-HD22

11,39,OD1,39,H,39-OD1-39-H

12,39,O,38,HZ1,39-O-38-HZ1

13,40,O,39,HD22,40-O-39-HD22

14,40,O,75,HE21,40-O-75-HE21

15,41,O,75,H,41-O-75-H

16,43,O,77,HH22,43-O-77-HH22

17,43,O,51,HE2,43-O-51-HE2

18,44,O,51,HE2,44-O-51-HE2

19,45,O,77,HH12,45-O-77-HH12

20,45,O,77,HE,45-O-77-HE

21,45,O,77,HH22,45-O-77-HH22

22,45,O,77,HH21,45-O-77-HH21

23,45,N,51,HE2,45-N-51-HE2

24,46,O,77,HH21,46-O-77-HH21

25,46,O,77,HH12,46-O-77-HH12

26,46,O,48,H,46-O-48-H

27,46,O,49,H,46-O-49-H

28,46,O,77,HH22,46-O-77-HH22

29,46,O,50,H,46-O-50-H

30,47,O,77,HH11,47-O-77-HH11

31,47,O,80,HG,47-O-80-HG

32,47,O,77,HH12,47-O-77-HH12

33,47,O,77,HE,47-O-77-HE

34,47,O,77,HH21,47-O-77-HH21

35,47,O,77,HH22,47-O-77-HH22

36,48,O,77,HH21,48-O-77-HH21

37,49,O,52,H,49-O-52-H

38,49,O,79,H,49-O-79-H

39,50,O,53,H,50-O-53-H

40,50,O,52,H,50-O-52-H

41,50,O,46,H,50-O-46-H

42,51,ND1,51,H,51-ND1-51-H

43,51,ND1,77,H,51-ND1-77-H

44,51,O,55,H,51-O-55-H

45,51,O,54,HG1,51-O-54-HG1

46,51,O,54,H,51-O-54-H

47,52,O,54,H,52-O-54-H

48,52,O,55,H,52-O-55-H

49,52,O,56,H,52-O-56-H

50,53,O,46,HE1,53-O-46-HE1

51,53,O,56,H,53-O-56-H

52,54,OG1,46,HE1,54-OG1-46-HE1

53,54,O,54,HG1,54-O-54-HG1

54,54,O,65,HH22,54-O-65-HH22

55,54,OG1,65,HH22,54-OG1-65-HH22

56,55,O,58,HD22,55-O-58-HD22

57,55,O,58,H,55-O-58-H

58,57,O,58,HD22,57-O-58-HD22

59,57,O,57,HZ3,57-O-57-HZ3

60,57,O,57,HZ2,57-O-57-HZ2

61,57,O,57,HZ1,57-O-57-HZ1

62,58,O,61,H,58-O-61-H

63,58,O,62,H,58-O-62-H

64,58,OD1,57,HZ1,58-OD1-57-HZ1

65,58,O,58,HD22,58-O-58-HD22

66,59,O,62,H,59-O-62-H

67,59,O,63,H,59-O-63-H

68,60,ND1,494,HH,60-ND1-494-HH

69,60,O,64,HG,60-O-64-HG

70,60,O,410,HD21,60-O-410-HD21

71,60,O,64,H,60-O-64-H

72,60,ND1,60,H,60-ND1-60-H

73,61,O,65,HE,61-O-65-HE

74,61,O,64,H,61-O-64-H

75,61,O,65,H,61-O-65-H

76,62,O,65,H,62-O-65-H

77,62,O,66,H,62-O-66-H

78,63,O,67,HG,63-O-67-HG

79,63,O,67,H,63-O-67-H

80,64,O,413,HE22,64-O-413-HE22

81,64,O,68,HE21,64-O-68-HE21

82,64,O,68,H,64-O-68-H

83,64,O,67,HG,64-O-67-HG

84,64,O,67,H,64-O-67-H

85,64,OG,410,HD21,64-OG-410-HD21

86,65,O,68,HE21,65-O-68-HE21

87,65,O,65,HH11,65-O-65-HH11

88,65,O,69,HE22,65-O-69-HE22

89,65,O,68,H,65-O-68-H

90,65,O,69,H,65-O-69-H

91,65,O,69,HE21,65-O-69-HE21

92,66,O,70,H,66-O-70-H

93,66,O,69,H,66-O-69-H

94,67,OG,87,HG,67-OG-87-HG

95,67,O,69,H,67-O-69-H

96,67,OG,413,HE21,67-OG-413-HE21

97,67,O,71,H,67-O-71-H

98,67,OG,68,HE21,67-OG-68-HE21

99,67,OG,413,HE22,67-OG-413-HE22

100,67,O,70,H,67-O-70-H

101,68,OE1,69,HE21,68-OE1-69-HE21

102,68,OE1,65,HH21,68-OE1-65-HH21

103,68,OE1,419,HZ2,68-OE1-419-HZ2

104,68,OE1,418,HE22,68-OE1-418-HE22

105,68,O,68,HE21,68-O-68-HE21

106,68,OE1,419,HZ3,68-OE1-419-HZ3

107,68,OE1,413,HE22,68-OE1-413-HE22

108,68,OE1,419,HZ1,68-OE1-419-HZ1

109,68,OE1,65,HH11,68-OE1-65-HH11

110,68,NE2,413,HE22,68-NE2-413-HE22

111,68,OE1,65,HE,68-OE1-65-HE

112,69,OE1,70,HH,69-OE1-70-HH

113,69,NE2,70,HH,69-NE2-70-HH

114,69,OE1,65,HH21,69-OE1-65-HH21

115,69,OE1,65,HH12,69-OE1-65-HH12

116,70,OH,69,HE21,70-OH-69-HE21

117,71,O,67,HG,71-O-67-HG

118,72,OD2,72,H,72-OD2-72-H

119,72,OD1,397,HZ3,72-OD1-397-HZ3

120,72,O,88,H,72-O-88-H

121,72,OD1,72,H,72-OD1-72-H

122,72,OD2,91,HG1,72-OD2-91-HG1

123,72,OD1,91,HG1,72-OD1-91-HG1

124,72,OD1,73,H,72-OD1-73-H

125,72,OD1,397,HZ2,72-OD1-397-HZ2

126,72,O,87,HG,72-O-87-HG

127,72,OD2,397,HZ2,72-OD2-397-HZ2

128,72,OD2,397,HZ1,72-OD2-397-HZ1

129,72,OD1,397,HZ1,72-OD1-397-HZ1

130,72,O,87,H,72-O-87-H

131,72,OD2,397,HZ3,72-OD2-397-HZ3

132,74,O,85,H,74-O-85-H

133,75,OE1,405,HH21,75-OE1-405-HH21

134,75,OE1,405,HE,75-OE1-405-HE

135,75,OE1,39,HD21,75-OE1-39-HD21

136,75,OE1,39,HD22,75-OE1-39-HD22

137,75,NE2,39,HD21,75-NE2-39-HD21

138,75,NE2,39,HD22,75-NE2-39-HD22

139,76,O,75,HE21,76-O-75-HE21

140,76,O,83,H,76-O-83-H

141,77,O,51,H,77-O-51-H

142,78,O,81,H,78-O-81-H

143,78,O,81,HG1,78-O-81-HG1

144,79,O,81,H,79-O-81-H

145,79,O,77,HE,79-O-77-HE

146,79,O,77,HH21,79-O-77-HH21

147,79,O,77,HH11,79-O-77-HH11

148,80,O,77,HE,80-O-77-HE

149,80,O,77,HH21,80-O-77-HH21

150,80,OG,77,HH21,80-OG-77-HH21

151,80,O,77,HH11,80-O-77-HH11

152,80,O,77,HH12,80-O-77-HH12

153,80,O,80,HG,80-O-80-HG

154,80,OG,241,HH12,80-OG-241-HH12

155,80,OG,81,HG1,80-OG-81-HG1

156,81,O,78,H,81-O-78-H

157,81,O,81,HG1,81-O-81-HG1

158,82,O,405,HH11,82-O-405-HH11

159,83,O,76,H,83-O-76-H

160,84,O,408,H,84-O-408-H

161,85,O,74,H,85-O-74-H

162,86,O,87,HG,86-O-87-HG

163,86,O,410,H,86-O-410-H

164,87,OG,410,HD22,87-OG-410-HD22

165,87,O,91,HG1,87-O-91-HG1

166,87,O,87,HG,87-O-87-HG

167,87,OG,67,HG,87-OG-67-HG

168,87,O,413,HE22,87-O-413-HE22

169,87,O,413,HE21,87-O-413-HE21

170,88,O,91,H,88-O-91-H

171,88,O,91,HG1,88-O-91-HG1

172,88,O,92,H,88-O-92-H

173,89,O,93,HE,89-O-93-HE

174,89,O,92,H,89-O-92-H

175,89,O,93,H,89-O-93-H

176,90,OD2,93,HE,90-OD2-93-HE

177,90,OD1,98,HH22,90-OD1-98-HH22

178,90,OD1,93,HH11,90-OD1-93-HH11

179,90,OD1,93,HE,90-OD1-93-HE

180,90,O,98,HH12,90-O-98-HH12

181,90,OD2,98,HH22,90-OD2-98-HH22

182,90,O,94,H,90-O-94-H

183,90,OD1,93,HH21,90-OD1-93-HH21

184,90,OD2,397,HZ1,90-OD2-397-HZ1

185,90,OD2,397,HZ3,90-OD2-397-HZ3

186,90,OD1,98,HH12,90-OD1-98-HH12

187,90,OD2,93,HH22,90-OD2-93-HH22

188,90,OD1,93,HH12,90-OD1-93-HH12

189,90,OD1,90,H,90-OD1-90-H

190,90,OD1,397,HZ1,90-OD1-397-HZ1

191,90,OD2,94,HE21,90-OD2-94-HE21

192,90,O,98,HH22,90-O-98-HH22

193,90,OD2,91,HG1,90-OD2-91-HG1

194,90,OD1,397,HZ2,90-OD1-397-HZ2

195,90,OD2,397,HZ2,90-OD2-397-HZ2

196,90,OD2,93,HH11,90-OD2-93-HH11

197,90,OD1,91,HG1,90-OD1-91-HG1

198,90,OD2,93,HH21,90-OD2-93-HH21

199,90,OD1,397,HZ3,90-OD1-397-HZ3

200,90,OD2,98,HH12,90-OD2-98-HH12

201,90,OD1,93,HH22,90-OD1-93-HH22

202,90,OD2,93,HH12,90-OD2-93-HH12

203,91,OG1,397,HZ1,91-OG1-397-HZ1

204,91,O,95,H,91-O-95-H

205,91,OG1,397,HZ2,91-OG1-397-HZ2

206,91,OG1,397,HZ3,91-OG1-397-HZ3

207,92,O,95,H,92-O-95-H

208,92,O,96,H,92-O-96-H

209,93,O,98,H,93-O-98-H

210,93,O,97,H,93-O-97-H

211,94,O,98,H,94-O-98-H

212,94,OE1,397,HZ2,94-OE1-397-HZ2

213,94,O,94,HE21,94-O-94-HE21

214,94,O,99,H,94-O-99-H

215,94,OE1,397,HZ3,94-OE1-397-HZ3

216,94,OE1,98,HH21,94-OE1-98-HH21

217,94,OE1,98,HH11,94-OE1-98-HH11

218,94,OE1,98,HE,94-OE1-98-HE

219,94,OE1,397,HZ1,94-OE1-397-HZ1

220,94,NE2,397,H,94-NE2-397-H

221,94,OE1,397,H,94-OE1-397-H

222,94,O,99,HE21,94-O-99-HE21

223,95,O,99,H,95-O-99-H

224,95,O,100,H,95-O-100-H

225,96,O,454,H,96-O-454-H

226,96,O,100,H,96-O-100-H

227,97,O,454,HZ2,97-O-454-HZ2

228,97,O,100,H,97-O-100-H

229,97,O,454,HZ1,97-O-454-HZ1

230,97,O,454,HZ3,97-O-454-HZ3

231,98,O,98,HH11,98-O-98-HH11

232,98,O,99,HE21,98-O-99-HE21

233,99,O,102,H,99-O-102-H

234,99,OE1,394,HG1,99-OE1-394-HG1

235,99,O,101,H,99-O-101-H

236,99,O,103,H,99-O-103-H

237,99,OE1,94,HE21,99-OE1-94-HE21

238,100,O,454,HZ1,100-O-454-HZ1

239,100,O,454,HZ3,100-O-454-HZ3

240,100,O,103,H,100-O-103-H

241,100,O,104,H,100-O-104-H

242,100,O,454,HZ2,100-O-454-HZ2

243,101,O,104,HZ2,101-O-104-HZ2

244,101,OD1,104,HZ3,101-OD1-104-HZ3

245,101,OD1,104,HZ2,101-OD1-104-HZ2

246,101,O,391,HG1,101-O-391-HG1

247,101,OD1,392,HH22,101-OD1-392-HH22

248,101,OD1,392,HH12,101-OD1-392-HH12

249,101,O,104,H,101-O-104-H

250,101,OD2,104,HZ3,101-OD2-104-HZ3

251,101,OD2,454,HZ2,101-OD2-454-HZ2

252,101,OD2,454,HZ1,101-OD2-454-HZ1

253,101,OD1,454,HZ1,101-OD1-454-HZ1

254,101,O,104,HZ1,101-O-104-HZ1

255,101,OD2,392,HH12,101-OD2-392-HH12

256,101,OD2,104,HZ2,101-OD2-104-HZ2

257,101,OD1,104,HZ1,101-OD1-104-HZ1

258,101,OD1,454,HZ2,101-OD1-454-HZ2

259,101,OD1,454,HZ3,101-OD1-454-HZ3

260,101,OD2,454,HZ3,101-OD2-454-HZ3

261,101,O,392,HH22,101-O-392-HH22

262,101,OD2,104,HZ1,101-OD2-104-HZ1

263,101,O,104,HZ3,101-O-104-HZ3

264,101,OD2,392,HH22,101-OD2-392-HH22

265,102,O,391,HG1,102-O-391-HG1

266,102,OD1,394,HG1,102-OD1-394-HG1

267,102,OD1,392,HH21,102-OD1-392-HH21

268,102,OD1,99,HE21,102-OD1-99-HE21

269,102,O,104,H,102-O-104-H

270,102,OD1,392,HE,102-OD1-392-HE

271,102,OD2,392,HE,102-OD2-392-HE

272,102,OD2,392,HH21,102-OD2-392-HH21

273,102,OD2,394,HG1,102-OD2-394-HG1

274,102,O,391,H,102-O-391-H

275,102,OD1,392,HH11,102-OD1-392-HH11

276,102,OD2,99,HE21,102-OD2-99-HE21

277,102,OD2,392,HH11,102-OD2-392-HH11

278,103,O,105,H,103-O-105-H

279,103,O,389,H,103-O-389-H

280,103,O,388,HD1,103-O-388-HD1

281,104,O,455,HH21,104-O-455-HH21

282,104,O,455,HE,104-O-455-HE

283,104,O,127,HG,104-O-127-HG

284,104,O,127,H,104-O-127-H

285,105,O,389,H,105-O-389-H

286,105,O,388,HD1,105-O-388-HD1

287,107,O,109,H,107-O-109-H

288,108,OD1,108,H,108-OD1-108-H

289,108,OD2,108,H,108-OD2-108-H

290,109,O,113,HG1,109-O-113-HG1

291,109,O,112,H,109-O-112-H

292,109,O,113,H,109-O-113-H

293,110,O,114,H,110-O-114-H

294,110,OH,252,HZ2,110-OH-252-HZ2

295,110,OH,252,HZ1,110-OH-252-HZ1

296,110,O,113,HG1,110-O-113-HG1

297,110,O,113,H,110-O-113-H

298,111,OG1,112,H,111-OG1-112-H

299,111,O,114,H,111-O-114-H

300,111,OG1,232,HD22,111-OG1-232-HD22

301,111,O,115,H,111-O-115-H

302,112,O,115,H,112-O-115-H

303,113,O,118,H,113-O-118-H

304,114,O,117,H,114-O-117-H

305,114,O,118,H,114-O-118-H

306,114,O,255,HD21,114-O-255-HD21

307,115,O,120,H,115-O-120-H

308,115,O,118,H,115-O-118-H

309,115,O,119,H,115-O-119-H

310,116,O,119,HE21,116-O-119-HE21

311,116,OG,309,HD22,116-OG-309-HD22

312,116,O,309,HD22,116-O-309-HD22

313,116,O,259,HH,116-O-259-HH

314,116,O,119,H,116-O-119-H

315,116,OG,259,HH,116-OG-259-HH

316,116,OG,255,HD21,116-OG-255-HD21

317,117,OD1,306,HZ2,117-OD1-306-HZ2

318,117,O,119,H,117-O-119-H

319,117,O,306,HZ3,117-O-306-HZ3

320,117,OD1,263,HE22,117-OD1-263-HE22

321,117,O,306,HZ1,117-O-306-HZ1

322,117,OD1,259,HH,117-OD1-259-HH

323,117,O,119,HE22,117-O-119-HE22

324,117,O,306,HZ2,117-O-306-HZ2

325,117,ND2,306,HZ2,117-ND2-306-HZ2

326,117,O,117,HD22,117-O-117-HD22

327,117,OD1,306,HZ1,117-OD1-306-HZ1

328,117,OD1,306,HZ3,117-OD1-306-HZ3

329,117,O,119,HE21,117-O-119-HE21

330,117,OD1,119,HE21,117-OD1-119-HE21

331,117,OD1,255,HD21,117-OD1-255-HD21

332,117,OD1,309,HD22,117-OD1-309-HD22

333,118,O,119,HE21,118-O-119-HE21

334,118,O,120,H,118-O-120-H

335,118,O,124,HG,118-O-124-HG

336,119,NE2,309,HD21,119-NE2-309-HD21

337,119,OE1,306,HZ1,119-OE1-306-HZ1

338,119,OE1,309,HD21,119-OE1-309-HD21

339,119,OE1,117,HD22,119-OE1-117-HD22

340,119,OE1,259,HH,119-OE1-259-HH

341,119,O,134,HH22,119-O-134-HH22

342,119,OE1,309,HD22,119-OE1-309-HD22

343,119,OE1,117,HD21,119-OE1-117-HD21

344,119,O,309,HD21,119-O-309-HD21

345,119,OE1,306,HZ3,119-OE1-306-HZ3

346,119,OE1,134,HH12,119-OE1-134-HH12

347,119,OE1,306,HZ2,119-OE1-306-HZ2

348,119,O,119,HE21,119-O-119-HE21

349,119,O,134,HH12,119-O-134-HH12

350,119,OE1,134,HH22,119-OE1-134-HH22

351,120,O,134,HH12,120-O-134-HH12

352,120,OG,122,H,120-OG-122-H

353,120,OG,123,H,120-OG-123-H

354,120,O,124,HG,120-O-124-HG

355,120,O,124,H,120-O-124-H

356,120,O,123,H,120-O-123-H

357,120,OG,122,HG,120-OG-122-HG

358,121,O,131,HE1,121-O-131-HE1

359,121,O,455,HH12,121-O-455-HH12

360,121,O,124,H,121-O-124-H

361,121,O,124,HG,121-O-124-HG

362,121,O,455,HH22,121-O-455-HH22

363,122,OG,516,H13,122-OG-516-H13

364,122,O,455,HH12,122-O-455-HH12

365,122,OG,123,H,122-OG-123-H

366,122,O,106,HE,122-O-106-HE

367,122,O,124,H,122-O-124-H

368,124,OG,455,HH22,124-OG-455-HH22

369,124,OG,126,H,124-OG-126-H

370,124,O,455,HH22,124-O-455-HH22

371,124,O,126,H,124-O-126-H

372,124,OG,455,HH12,124-OG-455-HH12

373,125,O,127,H,125-O-127-H

374,125,O,106,H,125-O-106-H

375,125,O,127,HG,125-O-127-HG

376,126,O,127,HG,126-O-127-HG

377,126,OD1,104,HZ3,126-OD1-104-HZ3

378,126,OD1,134,HH12,126-OD1-134-HH12

379,126,OD2,104,HZ1,126-OD2-104-HZ1

380,126,OD2,104,HZ2,126-OD2-104-HZ2

381,126,OD2,134,HH11,126-OD2-134-HH11

382,126,OD2,128,H,126-OD2-128-H

383,126,OD1,306,HZ3,126-OD1-306-HZ3

384,126,OD1,306,HZ1,126-OD1-306-HZ1

385,126,OD1,131,H,126-OD1-131-H

386,126,OD1,128,H,126-OD1-128-H

387,126,O,124,HG,126-O-124-HG

388,126,O,455,HH22,126-O-455-HH22

389,126,OD2,306,HZ2,126-OD2-306-HZ2

390,126,OD2,104,HZ3,126-OD2-104-HZ3

391,126,OD1,306,HZ2,126-OD1-306-HZ2

392,126,OD1,104,HZ2,126-OD1-104-HZ2

393,126,OD2,134,HH22,126-OD2-134-HH22
394,126,OD1,127,H,126-OD1-127-H
395,126,OD2,126,H,126-OD2-126-H
396,126,OD1,119,HE22,126-OD1-119-HE22
397,126,OD2,119,HE22,126-OD2-119-HE22
398,126,O,455,HH21,126-O-455-HH21
399,126,OD2,124,HG,126-OD2-124-HG
400,126,OD1,134,HH22,126-OD1-134-HH22
401,126,OD1,124,HG,126-OD1-124-HG
402,126,OD2,134,HH12,126-OD2-134-HH12
403,126,OD2,127,H,126-OD2-127-H
404,126,OD1,104,HZ1,126-OD1-104-HZ1
405,126,O,128,H,126-O-128-H
406,126,O,131,H,126-O-131-H
407,127,O,104,HZ3,127-O-104-HZ3
408,127,OG,455,HH21,127-OG-455-HH21
409,127,O,127,HG,127-O-127-HG
410,127,OG,455,HE,127-OG-455-HE
411,127,O,104,HZ1,127-O-104-HZ1
412,127,O,454,HZ3,127-O-454-HZ3
413,127,O,104,HZ2,127-O-104-HZ2
414,128,O,131,H,128-O-131-H
415,128,O,132,H,128-O-132-H
416,129,O,133,H,129-O-133-H
417,129,O,297,HD22,129-O-297-HD22
418,130,O,134,H,130-O-134-H
419,130,O,133,H,130-O-133-H
420,131,O,135,H,131-O-135-H
421,132,O,136,H,132-O-136-H
422,133,O,137,H,133-O-137-H
423,133,O,136,H,133-O-136-H
424,134,O,138,H,134-O-138-H
425,134,O,137,H,134-O-137-H
426,135,O,139,H,135-O-139-H
427,135,O,139,HE21,135-O-139-HE21
428,136,O,140,H,136-O-140-H
429,136,O,140,HD22,136-O-140-HD22
430,137,O,141,H,137-O-141-H
431,137,O,140,H,137-O-140-H
432,138,O,141,H,138-O-141-H
433,138,O,142,H,138-O-142-H
434,139,OE1,143,HZ1,139-OE1-143-HZ1
435,139,NE2,143,HZ1,139-NE2-143-HZ1
436,139,O,139,HE21,139-O-139-HE21
437,139,OE1,143,HZ2,139-OE1-143-HZ2
438,139,OE1,140,HD22,139-OE1-140-HD22
439,139,OE1,135,HE,139-OE1-135-HE
440,139,OE1,136,HH21,139-OE1-136-HH21
441,139,OE1,135,HH21,139-OE1-135-HH21
442,139,OE1,461,HG1,139-OE1-461-HG1
443,139,O,143,H,139-O-143-H
444,139,OE1,143,HZ3,139-OE1-143-HZ3
445,140,OD1,143,HZ1,140-OD1-143-HZ1
446,140,OD1,136,HH11,140-OD1-136-HH11
447,140,O,144,H,140-O-144-H
448,140,OD1,136,HH22,140-OD1-136-HH22
449,140,O,144,HG,140-O-144-HG
450,140,OD1,136,HE,140-OD1-136-HE
451,140,OD1,143,HZ2,140-OD1-143-HZ2
452,140,O,143,H,140-O-143-H
453,140,O,143,HZ2,140-O-143-HZ2
454,140,OD1,143,HZ3,140-OD1-143-HZ3
455,140,OD1,139,HE21,140-OD1-139-HE21
456,141,O,145,H,141-O-145-H
457,141,O,144,H,141-O-144-H
458,142,O,145,H,142-O-145-H
459,142,O,146,HG,142-O-146-HG
460,142,O,147,H,142-O-147-H
461,142,O,146,H,142-O-146-H
462,143,O,148,H,143-O-148-H
463,143,O,147,H,143-O-147-H
464,143,O,146,H,143-O-146-H
465,144,O,148,H,144-O-148-H
466,144,OG,143,HZ2,144-OG-143-HZ2
467,144,O,146,H,144-O-146-H
468,145,O,162,HE2,145-O-162-HE2
469,146,O,158,H,146-O-158-H
470,146,O,159,H,146-O-159-H
471,146,O,148,H,146-O-148-H
472,146,OG,147,H,146-OG-147-H

473,147,O,143,HZ1,147-O-143-HZ1

474,148,O,158,H,148-O-158-H

475,149,O,158,HH,149-O-158-HH

476,149,OG,150,H,149-OG-150-H

477,149,O,149,HG,149-O-149-HG

478,150,O,153,H,150-O-153-H

479,150,OD1,155,HG1,150-OD1-155-HG1

480,150,O,153,HG,150-O-153-HG

481,150,O,152,H,150-O-152-H

482,150,O,154,HG,150-O-154-HG

483,150,O,149,HG,150-O-149-HG

484,150,O,154,H,150-O-154-H

485,150,OD1,155,H,150-OD1-155-H

486,150,OD2,153,HG,150-OD2-153-HG

487,150,OD1,153,HG,150-OD1-153-HG

488,150,OD1,152,H,150-OD1-152-H

489,150,OD2,156,HG,150-OD2-156-HG

490,150,OD2,152,H,150-OD2-152-H

491,150,OD2,158,HH,150-OD2-158-HH

492,150,OD1,153,H,150-OD1-153-H

493,150,OD1,277,HE2,150-OD1-277-HE2

494,150,OD2,277,HE2,150-OD2-277-HE2

495,150,OD1,158,HH,150-OD1-158-HH

496,150,OD2,153,H,150-OD2-153-H

497,150,OD1,279,HH11,150-OD1-279-HH11

498,151,O,153,H,151-O-153-H

499,151,O,154,HG,151-O-154-HG

500,152,O,153,HG,152-O-153-HG

501,152,O,154,H,152-O-154-H

502,152,O,154,HG,152-O-154-HG

503,153,O,156,HG,153-O-156-HG

504,153,OG,155,H,153-OG-155-H

505,153,OG,155,HG1,153-OG-155-HG1

506,153,O,156,H,153-O-156-H

507,153,O,154,HG,153-O-154-HG

508,153,O,155,HG1,153-O-155-HG1

509,153,OG,156,H,153-OG-156-H

510,153,OG,154,H,153-OG-154-H

511,153,O,155,H,153-O-155-H

512,153,O,153,HG,153-O-153-HG

513,153,OG,156,HG,153-OG-156-HG

514,154,O,149,HG,154-O-149-HG

515,154,O,154,HG,154-O-154-HG

516,154,O,156,H,154-O-156-H

517,154,O,155,HG1,154-O-155-HG1

518,154,OG,155,HG1,154-OG-155-HG1

519,154,O,358,HH12,154-O-358-HH12

520,154,OG,155,H,154-OG-155-H

521,154,O,358,HH22,154-O-358-HH22

522,155,OG1,156,H,155-OG1-156-H

523,155,O,358,HH22,155-O-358-HH22

524,155,O,149,HG,155-O-149-HG

525,155,O,155,HG1,155-O-155-HG1

526,155,OG1,358,HH21,155-OG1-358-HH21

527,155,O,356,HH21,155-O-356-HH21

528,155,OG1,358,HH22,155-OG1-358-HH22

529,155,O,358,HH12,155-O-358-HH12

530,155,OG1,156,HG,155-OG1-156-HG

531,155,O,358,HH21,155-O-358-HH21

532,155,OG1,358,HH12,155-OG1-358-HH12

533,155,OG1,153,HG,155-OG1-153-HG

534,155,O,358,HE,155-O-358-HE

535,155,O,156,HG,155-O-156-HG

536,156,OG,358,HH21,156-OG-358-HH21

537,156,OG,155,HG1,156-OG-155-HG1

538,156,OG,356,HH21,156-OG-356-HH21

539,156,OG,153,HG,156-OG-153-HG

540,156,O,150,H,156-O-150-H

541,156,O,153,HG,156-O-153-HG

542,156,OG,358,HH22,156-OG-358-HH22

543,156,O,149,HG,156-O-149-HG

544,156,OG,358,HH12,156-OG-358-HH12

545,156,OG,356,HH22,156-OG-356-HH22

546,157,O,160,H,157-O-160-H

547,157,O,156,HG,157-O-156-HG

548,157,O,161,H,157-O-161-H

549,158,O,161,H,158-O-161-H

550,158,OH,277,HE2,158-OH-277-HE2

551,158,O,162,H,158-O-162-H

552,159,O,163,H,159-O-163-H

553,159,O,162,H,159-O-162-H

554,160,OE1,157,H,160-OE1-157-H

555,160,OE2,358,HH21,160-OE2-358-HH21

556,160,O,164,HG,160-O-164-HG

557,160,O,164,H,160-O-164-H

558,160,OE2,156,HG,160-OE2-156-HG

559,160,OE2,356,HE,160-OE2-356-HE

560,160,OE2,358,HE,160-OE2-358-HE

561,160,OE1,156,HG,160-OE1-156-HG

562,160,OE2,358,HH11,160-OE2-358-HH11

563,160,OE2,359,H,160-OE2-359-H

564,160,O,356,HH21,160-O-356-HH21

565,160,OE2,356,HH21,160-OE2-356-HH21

566,160,OE1,356,HH21,160-OE1-356-HH21

567,160,OE2,157,H,160-OE2-157-H

568,160,OE1,356,HE,160-OE1-356-HE

569,160,OE1,358,HH11,160-OE1-358-HH11

570,160,OE1,359,H,160-OE1-359-H

571,160,OE1,358,HH21,160-OE1-358-HH21

572,160,OE1,358,HE,160-OE1-358-HE

573,161,OE1,356,HH12,161-OE1-356-HH12

574,161,OE2,356,HH12,161-OE2-356-HH12

575,161,OE1,165,HZ1,161-OE1-165-HZ1

576,161,OE2,356,HH22,161-OE2-356-HH22

577,161,OE1,153,HG,161-OE1-153-HG

578,161,OE1,156,HG,161-OE1-156-HG

579,161,OE1,356,HH22,161-OE1-356-HH22

580,161,O,165,HZ3,161-O-165-HZ3

581,161,OE2,153,HG,161-OE2-153-HG

582,161,O,165,H,161-O-165-H

583,161,OE2,156,HG,161-OE2-156-HG

584,161,O,165,HZ1,161-O-165-HZ1

585,161,OE1,165,HZ2,161-OE1-165-HZ2

586,161,OE2,165,HZ3,161-OE2-165-HZ3

587,161,OE1,165,HZ3,161-OE1-165-HZ3

588,161,OE2,165,HZ1,161-OE2-165-HZ1

589,161,O,165,HZ2,161-O-165-HZ2

590,162,ND1,165,HZ3,162-ND1-165-HZ3

591,162,O,166,H,162-O-166-H

592,162,ND1,165,HZ2,162-ND1-165-HZ2

593,162,O,165,H,162-O-165-H

594,162,ND1,165,HZ1,162-ND1-165-HZ1

595,163,O,167,H,163-O-167-H

596,163,O,166,H,163-O-166-H

597,164,OG,356,HE,164-OG-356-HE

598,164,OG,356,HH11,164-OG-356-HH11

599,164,O,168,H,164-O-168-H

600,164,OG,356,HH21,164-OG-356-HH21

601,165,O,168,H,165-O-168-H

602,165,O,169,H,165-O-169-H

603,166,OE1,206,HE,166-OE1-206-HE

604,166,OE2,196,HD22,166-OE2-196-HD22

605,166,OE1,209,HE2,166-OE1-209-HE2

606,166,O,170,H,166-O-170-H

607,166,OE1,206,HH21,166-OE1-206-HH21

608,166,OE1,196,HD22,166-OE1-196-HD22

609,166,O,193,HG,166-O-193-HG

610,166,OE2,206,HH21,166-OE2-206-HH21

611,166,O,169,H,166-O-169-H

612,166,OE2,206,HE,166-OE2-206-HE

613,166,OE1,193,HG,166-OE1-193-HG

614,167,O,171,H,167-O-171-H

615,167,O,170,H,167-O-170-H

616,168,OE2,356,HH21,168-OE2-356-HH21

617,168,OE2,477,HH22,168-OE2-477-HH22

618,168,OE1,356,HH21,168-OE1-356-HH21

619,168,OE2,356,HH11,168-OE2-356-HH11

620,168,OE1,477,HH12,168-OE1-477-HH12

621,168,OE2,356,HH12,168-OE2-356-HH12

622,168,O,172,HG,168-O-172-HG

623,168,OE2,356,HE,168-OE2-356-HE

624,168,OE2,477,HH12,168-OE2-477-HH12

625,168,OE1,477,HH22,168-OE1-477-HH22

626,168,O,172,H,168-O-172-H

627,169,O,172,HG,169-O-172-HG

628,169,O,173,HG1,169-O-173-HG1

629,169,O,173,H,169-O-173-H

630,170,O,174,H,170-O-174-H

631,170,O,173,H,170-O-173-H

632,171,O,175,H,171-O-175-H

633,171,O,174,H,171-O-174-H

634,171,O,175,HE21,171-O-175-HE21

635,172,O,176,H,172-O-176-H

636,172,O,175,HE21,172-O-175-HE21

637,173,O,177,H,173-O-177-H

638,173,O,176,H,173-O-176-H

639,174,O,178,H,174-O-178-H

640,174,O,177,H,174-O-177-H

641,175,O,178,H,175-O-178-H

642,175,O,179,H,175-O-179-H

643,175,O,175,HE21,175-O-175-HE21

644,176,OE1,175,HE21,176-OE1-175-HE21

645,176,O,180,H,176-O-180-H

646,176,O,179,H,176-O-179-H

647,177,O,180,H,177-O-180-H

648,177,O,182,H,177-O-182-H

649,178,O,180,H,178-O-180-H

650,181,O,507,HE22,181-O-507-HE22

651,181,O,183,H,181-O-183-H

652,182,O,507,HE21,182-O-507-HE21

653,182,O,508,H,182-O-508-H

654,183,ND1,507,HE21,183-ND1-507-HE21

655,184,O,506,H,184-O-506-H

656,185,O,188,H,185-O-188-H

657,185,OD1,188,H,185-OD1-188-H

658,185,ND2,188,HH12,185-ND2-188-HH12

659,185,OD1,187,H,185-OD1-187-H

660,186,O,190,H,186-O-190-H

661,186,O,189,H,186-O-189-H

662,186,O,327,HG,186-O-327-HG

663,187,OH,222,HD21,187-OH-222-HD21

664,187,O,190,H,187-O-190-H

665,187,OH,188,HH21,187-OH-188-HH21

666,187,OH,499,HZ1,187-OH-499-HZ1

667,187,O,189,H,187-O-189-H

668,187,O,191,H,187-O-191-H

669,187,OH,499,HZ2,187-OH-499-HZ2

670,187,OH,499,HZ3,187-OH-499-HZ3

671,188,O,219,HD21,188-O-219-HD21

672,188,O,192,H,188-O-192-H

673,188,NH2,185,HD22,188-NH2-185-HD22

674,189,OH,188,HE,189-OH-188-HE

675,189,O,193,H,189-O-193-H

676,189,O,193,HG,189-O-193-HG

677,189,OH,188,HH21,189-OH-188-HH21

678,189,O,192,H,189-O-192-H

679,190,O,193,H,190-O-193-H

680,190,O,194,H,190-O-194-H

681,190,O,193,HG,190-O-193-HG

682,191,O,195,HG1,191-O-195-HG1

683,191,O,195,H,191-O-195-H

684,192,O,196,H,192-O-196-H

685,192,O,209,HE2,192-O-209-HE2

686,192,O,196,HD22,192-O-196-HD22

687,193,O,196,H,193-O-196-H

688,193,O,197,H,193-O-197-H

689,193,OG,209,HE2,193-OG-209-HE2

690,194,O,197,H,194-O-197-H

691,194,O,198,H,194-O-198-H

692,195,O,199,H,195-O-199-H

693,196,O,200,H,196-O-200-H

694,196,OD1,207,H,196-OD1-207-H

695,197,O,201,H,197-O-201-H

696,197,O,200,H,197-O-200-H

697,198,O,202,H,198-O-202-H

698,198,O,201,H,198-O-201-H

699,199,O,205,H,199-O-205-H

700,199,O,202,H,199-O-202-H

701,199,O,203,H,199-O-203-H

702,199,O,204,H,199-O-204-H

703,200,O,279,HH21,200-O-279-HH21

704,200,O,204,H,200-O-204-H

705,201,O,281,H,201-O-281-H

706,202,O,282,H,202-O-282-H

707,202,O,282,HG1,202-O-282-HG1

708,203,O,269,HE2,203-O-269-HE2

709,203,O,205,HE,203-O-205-HE

710,203,O,205,HH21,203-O-205-HH21

711,203,O,205,HH11,203-O-205-HH11

712,203,O,205,H,203-O-205-H

713,204,O,279,HE,204-O-279-HE
714,204,O,279,HH21,204-O-279-HH21
715,204,O,279,HH22,204-O-279-HH22
716,204,O,205,HE,204-O-205-HE
717,204,O,279,HH12,204-O-279-HH12
718,204,O,205,HH21,204-O-205-HH21
719,206,O,205,HH21,206-O-205-HH21
720,206,O,205,HE,206-O-205-HE
721,206,O,205,HH11,206-O-205-HH11
722,207,OH,264,HZ2,207-OH-264-HZ2
723,207,OH,205,HH11,207-OH-205-HH11
724,207,O,206,HH22,207-O-206-HH22
725,207,OH,205,HH12,207-OH-205-HH12
726,207,O,196,HD21,207-O-196-HD21
727,207,OH,205,HE,207-OH-205-HE
728,207,OH,205,HH21,207-OH-205-HH21
729,208,OD2,206,HH22,208-OD2-206-HH22
730,208,OD2,206,HH12,208-OD2-206-HH12
731,208,OD1,209,H,208-OD1-209-H
732,208,OD1,210,HD21,208-OD1-210-HD21
733,208,OD1,210,H,208-OD1-210-H
734,208,OD1,206,HH12,208-OD1-206-HH12
735,208,OD2,210,HD21,208-OD2-210-HD21
736,208,OD1,210,HD22,208-OD1-210-HD22
737,208,OD2,209,H,208-OD2-209-H
738,208,O,210,H,208-O-210-H
739,208,OD2,210,HD22,208-OD2-210-HD22
740,208,OD2,208,H,208-OD2-208-H
741,208,O,211,H,208-O-211-H
742,208,OD2,210,H,208-OD2-210-H
743,209,ND1,206,HH22,209-ND1-206-HH22
744,209,O,210,HD22,209-O-210-HD22
745,209,ND1,209,H,209-ND1-209-H
746,209,ND1,196,HD22,209-ND1-196-HD22
747,209,ND1,173,HG1,209-ND1-173-HG1
748,210,O,212,HE21,210-O-212-HE21
749,210,OD1,210,H,210-OD1-210-H
750,210,O,210,HD22,210-O-210-HD22
751,211,O,215,H,211-O-215-H
752,211,ND1,213,H,211-ND1-213-H
753,211,O,214,H,211-O-214-H
754,211,ND1,207,HH,211-ND1-207-HH
755,212,O,216,H,212-O-216-H
756,212,OE1,216,HG,212-OE1-216-HG
757,212,OE1,212,H,212-OE1-212-H
758,212,NE2,212,H,212-NE2-212-H
759,212,O,216,HG,212-O-216-HG
760,212,O,212,HE21,212-O-212-HE21
761,213,OE1,264,HZ3,213-OE1-264-HZ3
762,213,O,216,HG,213-O-216-HG
763,213,OE2,211,HE2,213-OE2-211-HE2
764,213,OE1,212,HE22,213-OE1-212-HE22
765,213,OE1,257,HZ3,213-OE1-257-HZ3
766,213,OE2,216,HG,213-OE2-216-HG
767,213,OE2,207,HH,213-OE2-207-HH
768,213,OE2,264,HZ2,213-OE2-264-HZ2
769,213,OE1,260,HG,213-OE1-260-HG
770,213,O,217,H,213-O-217-H
771,213,OE1,212,HE21,213-OE1-212-HE21
772,213,O,216,H,213-O-216-H
773,213,OE2,257,HZ2,213-OE2-257-HZ2
774,213,OE1,257,HZ1,213-OE1-257-HZ1
775,213,OE2,264,HZ1,213-OE2-264-HZ1
776,213,OE1,264,HZ1,213-OE1-264-HZ1
777,213,O,257,HZ2,213-O-257-HZ2
778,213,OE1,264,HZ2,213-OE1-264-HZ2
779,213,OE2,257,HZ1,213-OE2-257-HZ1
780,213,OE2,257,HZ3,213-OE2-257-HZ3
781,213,OE2,212,HE22,213-OE2-212-HE22
782,213,OE1,207,HH,213-OE1-207-HH
783,213,O,257,HZ3,213-O-257-HZ3
784,213,O,257,HZ1,213-O-257-HZ1
785,213,OE1,257,HZ2,213-OE1-257-HZ2
786,213,OE2,212,HE21,213-OE2-212-HE21
787,213,OE1,211,HE2,213-OE1-211-HE2
788,213,OE2,264,HZ3,213-OE2-264-HZ3
789,214,O,218,H,214-O-218-H
790,214,O,217,H,214-O-217-H
791,215,O,219,H,215-O-219-H
792,215,O,218,H,215-O-218-H

793,215,O,220,H,215-O-220-H

794,215,O,219,HD22,215-O-219-HD22

795,216,OG,257,HZ3,216-OG-257-HZ3

796,216,O,257,HZ2,216-O-257-HZ2

797,216,OG,257,HZ1,216-OG-257-HZ1

798,216,O,257,HZ1,216-O-257-HZ1

799,216,OG,212,HE21,216-OG-212-HE21

800,216,O,220,H,216-O-220-H

801,216,O,219,H,216-O-219-H

802,216,OG,257,HZ2,216-OG-257-HZ2

803,216,O,257,HZ3,216-O-257-HZ3

804,216,O,219,HD22,216-O-219-HD22

805,217,O,221,H,217-O-221-H

806,217,O,220,H,217-O-220-H

807,217,O,222,HD21,217-O-222-HD21

808,217,O,219,H,217-O-219-H

809,218,O,220,H,218-O-220-H

810,218,O,187,HH,218-O-187-HH

811,218,O,221,H,218-O-221-H

812,218,O,188,HH12,218-O-188-HH12

813,218,O,222,HD21,218-O-222-HD21

814,218,O,221,HD22,218-O-221-HD22

815,219,OD1,221,HD21,219-OD1-221-HD21

816,219,O,257,HZ2,219-O-257-HZ2

817,219,OD1,257,HZ3,219-OD1-257-HZ3

818,219,O,223,HD22,219-O-223-HD22

819,219,OD1,188,HH12,219-OD1-188-HH12

820,219,OD1,188,HH22,219-OD1-188-HH22

821,219,O,223,HD21,219-O-223-HD21

822,219,O,221,H,219-O-221-H

823,219,OD1,257,HZ2,219-OD1-257-HZ2

824,219,OD1,188,HH21,219-OD1-188-HH21

825,219,O,221,HD22,219-O-221-HD22

826,219,O,219,HD22,219-O-219-HD22

827,219,OD1,257,HZ1,219-OD1-257-HZ1

828,220,O,223,H,220-O-223-H

829,220,O,257,HZ1,220-O-257-HZ1

830,220,O,223,HD22,220-O-223-HD22

831,220,O,222,H,220-O-222-H

832,221,O,223,HD22,221-O-223-HD22

833,221,O,223,H,221-O-223-H

834,221,O,221,HD22,221-O-221-HD22

835,221,OD1,223,HD21,221-OD1-223-HD21

836,221,OD1,187,HH,221-OD1-187-HH

837,221,OD1,221,H,221-OD1-221-H

838,221,OD1,188,HE,221-OD1-188-HE

839,221,OD1,188,HH21,221-OD1-188-HH21

840,222,OD1,499,HZ1,222-OD1-499-HZ1

841,222,OD1,224,H,222-OD1-224-H

842,222,OD1,187,HH,222-OD1-187-HH

843,222,OD1,499,HZ2,222-OD1-499-HZ2

844,222,OD1,499,HZ3,222-OD1-499-HZ3

845,222,ND2,499,HZ2,222-ND2-499-HZ2

846,222,O,226,H,222-O-226-H

847,222,O,499,HZ1,222-O-499-HZ1

848,222,ND2,499,HZ3,222-ND2-499-HZ3

849,222,O,499,HZ3,222-O-499-HZ3

850,222,O,499,HZ2,222-O-499-HZ2

851,222,OD1,223,H,222-OD1-223-H

852,222,ND2,499,HZ1,222-ND2-499-HZ1

853,222,O,225,H,222-O-225-H

854,223,O,226,H,223-O-226-H

855,223,O,227,H,223-O-227-H

856,223,O,247,HG,223-O-247-HG

857,223,OD1,257,HZ2,223-OD1-257-HZ2

858,223,OD1,221,HD22,223-OD1-221-HD22

859,223,O,223,HD22,223-O-223-HD22

860,223,OD1,223,H,223-OD1-223-H

861,224,O,228,H,224-O-228-H

862,224,O,227,H,224-O-227-H

863,225,O,496,H,225-O-496-H

864,225,O,229,H,225-O-229-H

865,226,OE1,223,HD22,226-OE1-223-HD22

866,226,OE2,226,H,226-OE2-226-H

867,226,OE2,223,HD21,226-OE2-223-HD21

868,226,OE1,57,HZ1,226-OE1-57-HZ1

869,226,O,229,H,226-O-229-H

870,226,O,57,HZ2,226-O-57-HZ2

871,226,OE1,223,HD21,226-OE1-223-HD21

872,226,OE2,223,HD22,226-OE2-223-HD22

873,226,O,232,HD22,226-O-232-HD22

874,226,OE2,57,HZ2,226-OE2-57-HZ2

875,226,OE1,57,HZ3,226-OE1-57-HZ3

876,226,OE2,57,HZ3,226-OE2-57-HZ3

877,226,OE1,226,H,226-OE1-226-H

878,226,OE2,247,HG,226-OE2-247-HG

879,226,OE1,57,HZ2,226-OE1-57-HZ2

880,226,OE1,247,HG,226-OE1-247-HG

881,227,O,229,H,227-O-229-H

882,227,O,232,HD22,227-O-232-HD22

883,228,O,230,H,228-O-230-H

884,228,O,231,H,228-O-231-H

885,229,O,494,HH,229-O-494-HH

886,229,O,231,H,229-O-231-H

887,229,O,232,H,229-O-232-H

888,229,O,230,HG,229-O-230-HG

889,230,O,494,HH,230-O-494-HH

890,230,O,230,HG,230-O-230-HG

891,230,OG,494,HH,230-OG-494-HH

892,230,O,232,H,230-O-232-H

893,232,OD1,235,H,232-OD1-235-H

894,232,O,236,H,232-O-236-H

895,232,OD1,234,H,232-OD1-234-H

896,232,OD1,245,HD21,232-OD1-245-HD21

897,232,O,235,H,232-O-235-H

898,232,OD1,247,HG,232-OD1-247-HG

899,233,O,237,H,233-O-237-H

900,233,O,236,H,233-O-236-H

901,234,O,237,H,234-O-237-H

902,234,O,241,HH11,234-O-241-HH11

903,234,O,241,HH12,234-O-241-HH12

904,235,OD2,232,H,235-OD2-232-H

905,235,OD1,111,H,235-OD1-111-H

906,235,OD2,111,HG1,235-OD2-111-HG1

907,235,O,241,HH12,235-O-241-HH12

908,235,OD2,241,HH11,235-OD2-241-HH11

909,235,OD2,111,H,235-OD2-111-H

910,235,OD1,241,HH22,235-OD1-241-HH22

911,235,OD1,241,HH12,235-OD1-241-HH12

912,235,OD1,111,HG1,235-OD1-111-HG1

913,235,O,241,HH11,235-O-241-HH11

914,235,OD1,232,HD22,235-OD1-232-HD22

915,235,OD1,110,H,235-OD1-110-H

916,235,OD1,241,HH11,235-OD1-241-HH11

917,235,OD2,232,HD22,235-OD2-232-HD22

918,236,O,81,HG1,236-O-81-HG1

919,236,O,80,H,236-O-80-H

920,236,O,80,HG,236-O-80-HG

921,236,O,241,HH12,236-O-241-HH12

922,236,O,241,HH11,236-O-241-HH11

923,237,O,240,H,237-O-240-H

924,237,O,241,H,237-O-241-H

925,238,O,241,H,238-O-241-H

926,238,O,242,H,238-O-242-H

927,239,O,243,H,239-O-243-H

928,239,O,242,H,239-O-242-H

929,240,O,245,HD22,240-O-245-HD22

930,240,O,245,HD21,240-O-245-HD21

931,240,O,243,H,240-O-243-H

932,242,OH,241,HH21,242-OH-241-HH21

933,243,O,245,HD22,243-O-245-HD22

934,243,O,245,H,243-O-245-H

935,244,O,245,HD22,244-O-245-HD22

936,244,O,249,HD21,244-O-249-HD21

937,245,O,248,H,245-O-248-H

938,245,OD1,247,HG,245-OD1-247-HG

939,245,O,249,H,245-O-249-H

940,245,O,249,HD22,245-O-249-HD22

941,245,OD1,247,H,245-OD1-247-H

942,245,OD1,248,H,245-OD1-248-H

943,246,O,250,H,246-O-250-H

944,246,O,249,H,246-O-249-H

945,246,O,249,HD22,246-O-249-HD22

946,247,O,250,H,247-O-250-H

947,247,O,251,H,247-O-251-H

948,247,OG,245,HD21,247-OG-245-HD21

949,247,O,223,HD21,247-O-223-HD21

950,247,OG,223,HD21,247-OG-223-HD21

951,247,OG,245,HD22,247-OG-245-HD22

952,247,OG,223,HD22,247-OG-223-HD22

953,247,OG,232,HD21,247-OG-232-HD21

954,248,O,252,HZ2,248-O-252-HZ2

955,248,O,252,H,248-O-252-H

956,248,O,251,H,248-O-251-H

957,249,O,252,H,249-O-252-H

958,249,O,252,HZ1,249-O-252-HZ1

959,249,OD1,252,HZ2,249-OD1-252-HZ2

960,249,OD1,252,HZ3,249-OD1-252-HZ3

961,249,O,252,HZ3,249-O-252-HZ3

962,249,O,249,HD22,249-O-249-HD22

963,249,OD1,245,H,249-OD1-245-H

964,249,OD1,252,HZ1,249-OD1-252-HZ1

965,249,O,253,H,249-O-253-H

966,249,O,252,HZ2,249-O-252-HZ2

967,250,O,254,H,250-O-254-H

968,250,O,253,H,250-O-253-H

969,250,O,223,HD21,250-O-223-HD21

970,251,O,255,HD22,251-O-255-HD22

971,251,O,254,H,251-O-254-H

972,251,O,255,H,251-O-255-H

973,252,O,255,H,252-O-255-H

974,252,O,256,H,252-O-256-H

975,253,OD1,249,HD21,253-OD1-249-HD21

976,253,OD2,252,HZ2,253-OD2-252-HZ2

977,253,OD2,249,HD21,253-OD2-249-HD21

978,253,OD1,257,HZ1,253-OD1-257-HZ1

979,253,OD1,257,HZ3,253-OD1-257-HZ3

980,253,OD1,252,HZ1,253-OD1-252-HZ1

981,253,OD2,257,HZ2,253-OD2-257-HZ2

982,253,OD2,252,HZ1,253-OD2-252-HZ1

983,253,O,257,H,253-O-257-H

984,253,O,257,HZ3,253-O-257-HZ3

985,253,OD2,257,HZ3,253-OD2-257-HZ3

986,253,O,256,H,253-O-256-H

987,253,OD2,257,HZ1,253-OD2-257-HZ1

988,253,OD1,252,HZ3,253-OD1-252-HZ3

989,253,OD1,252,HZ2,253-OD1-252-HZ2

990,253,OD1,221,HD22,253-OD1-221-HD22

991,253,OD2,252,HZ3,253-OD2-252-HZ3

992,253,OD1,257,HZ2,253-OD1-257-HZ2

993,254,O,257,H,254-O-257-H

994,254,O,258,H,254-O-258-H

995,254,O,256,H,254-O-256-H

996,255,O,255,HD22,255-O-255-HD22

997,255,O,117,HD21,255-O-117-HD21

998,255,OD1,116,H,255-OD1-116-H

999,255,OD1,252,HZ1,255-OD1-252-HZ1

1000,255,OD1,117,H,255-OD1-117-H

1001,255,O,259,H,255-O-259-H

1002,256,OE1,117,HD21,256-OE1-117-HD21

1003,256,OE2,257,HZ3,256-OE2-257-HZ3

1004,256,OE1,252,HZ2,256-OE1-252-HZ2

1005,256,OE1,252,HZ1,256-OE1-252-HZ1

1006,256,O,260,HG,256-O-260-HG

1007,256,OE2,117,HD21,256-OE2-117-HD21

1008,256,OE2,260,HG,256-OE2-260-HG

1009,256,OE1,257,HZ3,256-OE1-257-HZ3

1010,256,OE1,252,HZ3,256-OE1-252-HZ3

1011,256,OE1,260,HG,256-OE1-260-HG

1012,256,O,260,H,256-O-260-H

1013,256,OE2,255,HD22,256-OE2-255-HD22

1014,256,OE2,252,HZ2,256-OE2-252-HZ2

1015,256,OE2,257,HZ1,256-OE2-257-HZ1

1016,256,OE2,252,HZ1,256-OE2-252-HZ1

1017,256,OE1,257,HZ2,256-OE1-257-HZ2

1018,256,OE1,257,HZ1,256-OE1-257-HZ1

1019,256,OE2,252,HZ3,256-OE2-252-HZ3

1020,256,O,259,H,256-O-259-H

1021,257,O,260,HG,257-O-260-HG

1022,257,O,261,H,257-O-261-H

1023,258,O,262,H,258-O-262-H

1024,258,O,261,H,258-O-261-H

1025,259,OH,306,HZ2,259-OH-306-HZ2

1026,259,OH,306,HZ3,259-OH-306-HZ3

1027,259,O,262,H,259-O-262-H

1028,259,OH,119,HE22,259-OH-119-HE22

1029,259,OH,263,HE21,259-OH-263-HE21

1030,259,O,263,HE21,259-O-263-HE21

1031,259,OH,309,HD22,259-OH-309-HD22

1032,259,O,263,H,259-O-263-H

1033,260,OG,264,HZ3,260-OG-264-HZ3

1034,260,O,264,HZ2,260-O-264-HZ2

1035,260,OG,264,HZ1,260-OG-264-HZ1

1036,260,O,264,HZ1,260-O-264-HZ1

1037,260,OG,257,HZ1,260-OG-257-HZ1

1038,260,O,264,H,260-O-264-H

1039,260,O,264,HZ3,260-O-264-HZ3

1040,260,OG,264,HZ2,260-OG-264-HZ2

1041,261,O,264,H,261-O-264-H

1042,261,O,265,H,261-O-265-H

1043,262,O,266,H,262-O-266-H

1044,262,O,265,H,262-O-265-H

1045,263,OE1,267,HZ2,263-OE1-267-HZ2

1046,263,O,267,H,263-O-267-H

1047,263,O,267,HZ2,263-O-267-HZ2

1048,263,OE1,267,HZ3,263-OE1-267-HZ3

1049,263,OE1,267,HZ1,263-OE1-267-HZ1

1050,263,O,267,HZ1,263-O-267-HZ1

1051,263,O,263,HE21,263-O-263-HE21

1052,263,OE1,259,HH,263-OE1-259-HH

1053,263,O,267,HZ3,263-O-267-HZ3

1054,263,OE1,117,HD21,263-OE1-117-HD21

1055,264,O,267,H,264-O-267-H

1056,264,O,267,HZ2,264-O-267-HZ2

1057,264,O,268,H,264-O-268-H

1058,264,O,267,HZ1,264-O-267-HZ1

1059,265,O,269,H,265-O-269-H

1060,265,O,205,HH12,265-O-205-HH12

1061,265,O,268,H,265-O-268-H

1062,265,O,205,HH22,265-O-205-HH22

1063,266,O,269,H,266-O-269-H

1064,266,O,270,H,266-O-270-H

1065,267,O,270,H,267-O-270-H

1066,267,O,271,HZ3,267-O-271-HZ3

1067,267,O,271,H,267-O-271-H

1068,267,O,271,HZ2,267-O-271-HZ2

1069,267,O,271,HZ1,267-O-271-HZ1

1070,268,OE1,264,HZ3,268-OE1-264-HZ3

1071,268,OE1,264,HZ2,268-OE1-264-HZ2

1072,268,OE1,205,HE,268-OE1-205-HE

1073,268,OE2,271,HZ2,268-OE2-271-HZ2

1074,268,OE2,264,HZ2,268-OE2-264-HZ2

1075,268,OE2,264,HZ3,268-OE2-264-HZ3

1076,268,OE1,205,HH21,268-OE1-205-HH21

1077,268,OE2,271,HZ3,268-OE2-271-HZ3

1078,268,OE2,264,HZ1,268-OE2-264-HZ1

1079,268,O,271,H,268-O-271-H

1080,268,OE2,267,HZ3,268-OE2-267-HZ3

1081,268,OE2,267,HZ2,268-OE2-267-HZ2

1082,268,OE2,205,HH22,268-OE2-205-HH22

1083,268,OE1,267,HZ1,268-OE1-267-HZ1

1084,268,OE1,205,HH22,268-OE1-205-HH22

1085,268,O,272,HG1,268-O-272-HG1

1086,268,OE2,205,HH21,268-OE2-205-HH21

1087,268,OE1,271,HZ3,268-OE1-271-HZ3

1088,268,O,272,H,268-O-272-H

1089,268,OE1,271,HZ1,268-OE1-271-HZ1

1090,268,OE1,264,HZ1,268-OE1-264-HZ1

1091,268,OE2,205,HE,268-OE2-205-HE

1092,268,OE1,271,HZ2,268-OE1-271-HZ2

1093,268,OE2,267,HZ1,268-OE2-267-HZ1

1094,268,OE1,267,HZ2,268-OE1-267-HZ2

1095,268,OE2,205,HH12,268-OE2-205-HH12

1096,268,OE1,205,HH12,268-OE1-205-HH12

1097,268,OE2,271,HZ1,268-OE2-271-HZ1

1098,268,OE1,267,HZ3,268-OE1-267-HZ3

1099,269,O,272,H,269-O-272-H

1100,269,O,279,HH22,269-O-279-HH22

1101,269,ND1,272,HG1,269-ND1-272-HG1

1102,269,ND1,205,HH22,269-ND1-205-HH22

1103,269,ND1,205,HH12,269-ND1-205-HH12

1104,269,NE2,275,HZ3,269-NE2-275-HZ3

1105,269,O,271,H,269-O-271-H

1106,269,O,272,HG1,269-O-272-HG1

1107,269,O,275,HZ2,269-O-275-HZ2

1108,269,O,279,HH12,269-O-279-HH12

1109,269,ND1,205,HH21,269-ND1-205-HH21

1110,269,O,273,H,269-O-273-H

1111,269,O,275,HZ3,269-O-275-HZ3

1112,269,O,275,HZ1,269-O-275-HZ1

1113,270,O,275,HZ2,270-O-275-HZ2

1114,270,O,273,H,270-O-273-H

1115,270,O,275,HZ3,270-O-275-HZ3

1116,270,O,275,HZ1,270-O-275-HZ1

1117,270,OH,290,HE21,270-OH-290-HE21

1118,270,O,272,H,270-O-272-H

1119,270,OH,290,HE22,270-OH-290-HE22

1120,271,O,273,H,271-O-273-H

1121,271,O,272,HG1,271-O-272-HG1

1122,272,OG1,279,HH11,272-OG1-279-HH11

1123,272,O,279,HH22,272-O-279-HH22

1124,272,OG1,205,HH21,272-OG1-205-HH21

1125,272,OG1,279,HH12,272-OG1-279-HH12

1126,272,OG1,205,HH22,272-OG1-205-HH22

1127,272,OG1,205,HH12,272-OG1-205-HH12

1128,272,O,275,HZ2,272-O-275-HZ2

1129,272,OG1,279,HH22,272-OG1-279-HH22

1130,272,O,275,HZ3,272-O-275-HZ3

1131,272,O,272,HG1,272-O-272-HG1

1132,272,O,279,HH12,272-O-279-HH12

1133,272,O,275,HZ1,272-O-275-HZ1

1134,273,O,275,H,273-O-275-H

1135,273,O,275,HZ2,273-O-275-HZ2

1136,273,O,275,HZ3,273-O-275-HZ3

1137,274,O,276,H,274-O-276-H

1138,274,OE2,274,H,274-OE2-274-H

1139,274,OE2,273,H,274-OE2-273-H

1140,274,OE1,276,H,274-OE1-276-H

1141,274,OE2,275,H,274-OE2-275-H

1142,274,OE2,277,H,274-OE2-277-H

1143,274,O,279,HH22,274-O-279-HH22

1144,274,OE1,274,H,274-OE1-274-H

1145,274,OE1,275,H,274-OE1-275-H

1146,274,O,275,HZ2,274-O-275-HZ2

1147,274,O,279,HH12,274-O-279-HH12

1148,274,O,272,HG1,274-O-272-HG1

1149,274,O,279,HH11,274-O-279-HH11

1150,274,OE2,279,HH22,274-OE2-279-HH22

1151,274,OE2,276,H,274-OE2-276-H

1152,274,OE1,279,HH22,274-OE1-279-HH22

1153,274,O,277,H,274-O-277-H

1154,274,OE1,279,HH21,274-OE1-279-HH21

1155,274,OE2,279,HH12,274-OE2-279-HH12

1156,274,OE1,273,H,274-OE1-273-H

1157,276,O,275,HZ1,276-O-275-HZ1

1158,276,O,275,HZ3,276-O-275-HZ3

1159,276,O,275,HZ2,276-O-275-HZ2

1160,276,O,278,H,276-O-278-H

1161,277,ND1,278,H,277-ND1-278-H

1162,277,O,279,HH11,277-O-279-HH11

1163,277,ND1,277,H,277-ND1-277-H

1164,277,O,275,HZ2,277-O-275-HZ2

1165,277,ND1,279,HH21,277-ND1-279-HH21

1166,277,O,279,HH21,277-O-279-HH21

1167,277,O,279,HE,277-O-279-HE

1168,278,O,158,HH,278-O-158-HH

1169,279,O,279,HE,279-O-279-HE

1170,280,OD1,282,HG1,280-OD1-282-HG1

1171,280,OD2,205,HH12,280-OD2-205-HH12

1172,280,O,284,HG,280-O-284-HG

1173,280,OD2,269,HE2,280-OD2-269-HE2

1174,280,O,284,H,280-O-284-H

1175,280,OD1,282,H,280-OD1-282-H

1176,280,OD2,279,HE,280-OD2-279-HE

1177,280,OD2,279,HH11,280-OD2-279-HH11

1178,280,OD1,269,HE2,280-OD1-269-HE2

1179,280,OD1,283,H,280-OD1-283-H

1180,280,OD2,279,HH21,280-OD2-279-HH21

1181,281,O,285,H,281-O-285-H

1182,281,O,284,H,281-O-284-H

1183,281,O,284,HG,281-O-284-HG

1184,282,O,286,H,282-O-286-H

1185,282,O,285,H,282-O-285-H

1186,283,OD1,279,HH21,283-OD1-279-HH21

1187,283,OD2,279,HE,283-OD2-279-HE

1188,283,OD2,279,HH21,283-OD2-279-HH21

1189,283,O,286,H,283-O-286-H

1190,283,OD2,279,H,283-OD2-279-H

1191,283,OD2,269,HE2,283-OD2-269-HE2

1192,283,OD1,275,HZ3,283-OD1-275-HZ3

1193,283,O,275,HZ3,283-O-275-HZ3

1194,283,OD1,275,HZ1,283-OD1-275-HZ1

1195,283,O,275,HZ2,283-O-275-HZ2

1196,283,OD2,275,HZ1,283-OD2-275-HZ1

1197,283,OD2,275,HZ3,283-OD2-275-HZ3

1198,283,OD2,275,HZ2,283-OD2-275-HZ2

1199,283,OD1,275,HZ2,283-OD1-275-HZ2

1200,283,O,287,H,283-O-287-H

1201,283,O,275,HZ1,283-O-275-HZ1

1202,283,OD1,279,HE,283-OD1-279-HE

1203,283,OD1,269,HE2,283-OD1-269-HE2

1204,284,O,288,H,284-O-288-H

1205,285,O,288,H,285-O-288-H

1206,285,O,289,H,285-O-289-H

1207,286,O,290,HE22,286-O-290-HE22

1208,286,O,290,H,286-O-290-H

1209,286,O,289,H,286-O-289-H

1210,286,O,290,HE21,286-O-290-HE21

1211,287,OE1,275,HZ2,287-OE1-275-HZ2

1212,287,OE1,275,HZ1,287-OE1-275-HZ1

1213,287,O,290,HE21,287-O-290-HE21

1214,287,OE1,275,HZ3,287-OE1-275-HZ3

1215,287,OE2,290,HE21,287-OE2-290-HE21

1216,287,O,291,H,287-O-291-H

1217,287,OE2,275,HZ2,287-OE2-275-HZ2

1218,287,OE1,290,HE22,287-OE1-290-HE22

1219,287,OE1,290,HE21,287-OE1-290-HE21

1220,287,OE1,284,HG,287-OE1-284-HG

1221,287,OE2,275,HZ1,287-OE2-275-HZ1

1222,287,O,290,H,287-O-290-H

1223,287,OE2,275,HZ3,287-OE2-275-HZ3

1224,287,OE2,290,HE22,287-OE2-290-HE22

1225,288,O,292,H,288-O-292-H

1226,288,O,291,H,288-O-291-H

1227,289,O,292,HZ2,289-O-292-HZ2

1228,289,O,292,H,289-O-292-H

1229,289,O,292,HZ1,289-O-292-HZ1

1230,289,O,293,HE22,289-O-293-HE22

1231,289,O,292,HZ3,289-O-292-HZ3

1232,289,O,293,HE21,289-O-293-HE21

1233,290,O,293,HE21,290-O-293-HE21

1234,290,OE1,275,HZ2,290-OE1-275-HZ2

1235,290,O,292,H,290-O-292-H

1236,290,O,275,HZ2,290-O-275-HZ2

1237,290,O,293,H,290-O-293-H

1238,290,OE1,293,HE22,290-OE1-293-HE22

1239,290,O,293,HE22,290-O-293-HE22

1240,290,OE1,275,HZ3,290-OE1-275-HZ3

1241,290,OE1,270,HH,290-OE1-270-HH

1242,290,OE1,293,HE21,290-OE1-293-HE21

1243,290,O,294,H,290-O-294-H

1244,290,OE1,275,HZ1,290-OE1-275-HZ1

1245,291,OE2,290,HE22,291-OE2-290-HE22

1246,291,OE2,275,HZ2,291-OE2-275-HZ2

1247,291,OE2,275,HZ3,291-OE2-275-HZ3

1248,291,OE1,275,HZ2,291-OE1-275-HZ2

1249,291,OE1,275,HZ3,291-OE1-275-HZ3

1250,291,O,294,H,291-O-294-H

1251,291,O,295,H,291-O-295-H

1252,291,OE1,290,HE21,291-OE1-290-HE21

1253,291,OE1,293,HE22,291-OE1-293-HE22

1254,291,OE1,275,HZ1,291-OE1-275-HZ1

1255,291,O,293,H,291-O-293-H

1256,291,OE1,290,HE22,291-OE1-290-HE22

1257,291,OE2,275,HZ1,291-OE2-275-HZ1

1258,291,OE2,293,HE22,291-OE2-293-HE22

1259,292,O,295,H,292-O-295-H

1260,293,OE1,292,HZ2,293-OE1-292-HZ2

1261,293,OE1,293,H,293-OE1-293-H

1262,293,NE2,293,H,293-NE2-293-H

1263,293,OE1,292,HZ3,293-OE1-292-HZ3

1264,293,O,293,HE21,293-O-293-HE21

1265,293,OE1,292,HZ1,293-OE1-292-HZ1

1266,293,OE1,294,H,293-OE1-294-H

1267,293,OE1,299,H,293-OE1-299-H

1268,293,O,295,H,293-O-295-H

1269,295,OD2,297,HD21,295-OD2-297-HD21

1270,295,OD1,298,H,295-OD1-298-H

1271,295,OD2,297,H,295-OD2-297-H

1272,295,OD2,288,HE2,295-OD2-288-HE2

1273,295,OD1,296,H,295-OD1-296-H

1274,295,OD1,297,H,295-OD1-297-H

1275,295,OD2,136,HH12,295-OD2-136-HH12

1276,295,OD1,297,HD21,295-OD1-297-HD21

1277,295,OD2,298,H,295-OD2-298-H

1278,295,OD2,296,H,295-OD2-296-H

1279,295,OD1,288,HE2,295-OD1-288-HE2

1280,295,O,298,H,295-O-298-H

1281,295,OD1,136,HH22,295-OD1-136-HH22

1282,296,OE1,136,HH12,296-OE1-136-HH12

1283,296,O,298,H,296-O-298-H

1284,296,OE1,136,HH22,296-OE1-136-HH22

1285,296,OE2,136,HH12,296-OE2-136-HH12

1286,296,OE1,297,HD22,296-OE1-297-HD22

1287,296,OE2,136,HH22,296-OE2-136-HH22

1288,296,OE1,297,HD21,296-OE1-297-HD21

1289,296,OE2,297,HD21,296-OE2-297-HD21

1290,296,OE1,296,H,296-OE1-296-H

1291,296,OE2,296,H,296-OE2-296-H

1292,296,OE2,297,HD22,296-OE2-297-HD22

1293,297,OD1,136,HE,297-OD1-136-HE

1294,297,OD1,136,HH22,297-OD1-136-HH22

1295,297,O,134,HH21,297-O-134-HH21

1296,297,O,288,HE2,297-O-288-HE2

1297,297,OD1,136,HH21,297-OD1-136-HH21

1298,297,OD1,136,HH11,297-OD1-136-HH11

1299,297,O,297,HD21,297-O-297-HD21

1300,298,O,299,HD22,298-O-299-HD22

1301,298,O,300,H,298-O-300-H

1302,299,O,293,HE22,299-O-293-HE22

1303,299,OD1,300,H,299-OD1-300-H

1304,299,OD1,301,HE21,299-OD1-301-HE21

1305,299,OD1,301,H,299-OD1-301-H

1306,299,O,302,H,299-O-302-H

1307,299,OD1,301,HE22,299-OD1-301-HE22

1308,299,ND2,301,HE22,299-ND2-301-HE22

1309,299,OD1,302,H,299-OD1-302-H

1310,300,O,293,HE22,300-O-293-HE22

1311,300,O,292,HZ3,300-O-292-HZ3

1312,300,O,292,HZ2,300-O-292-HZ2

1313,300,O,302,H,300-O-302-H

1314,300,O,292,HZ1,300-O-292-HZ1

1315,301,O,306,HZ2,301-O-306-HZ2

1316,301,OE1,301,H,301-OE1-301-H

1317,301,O,306,HZ3,301-O-306-HZ3

1318,301,O,301,HE21,301-O-301-HE21

1319,301,O,303,H,301-O-303-H

1320,301,OE1,299,HD22,301-OE1-299-HD22

1321,301,OE1,299,HD21,301-OE1-299-HD21

1322,301,O,306,HZ1,301-O-306-HZ1

1323,302,O,292,HZ1,302-O-292-HZ1

1324,302,O,292,HZ2,302-O-292-HZ2

1325,302,O,292,HZ3,302-O-292-HZ3

1326,303,OG,306,HZ1,303-OG-306-HZ1

1327,303,O,292,HZ2,303-O-292-HZ2

1328,303,OG,305,H,303-OG-305-H

1329,303,OG,306,HZ2,303-OG-306-HZ2

1330,303,O,307,H,303-O-307-H

1331,303,OG,306,H,303-OG-306-H

1332,303,O,306,H,303-O-306-H

1333,304,OD1,292,HZ3,304-OD1-292-HZ3

1334,304,OD2,292,HZ3,304-OD2-292-HZ3

1335,304,OD1,270,HH,304-OD1-270-HH

1336,304,OD2,305,H,304-OD2-305-H

1337,304,OD2,292,HZ2,304-OD2-292-HZ2

1338,304,OD2,304,H,304-OD2-304-H

1339,304,OD1,292,HZ2,304-OD1-292-HZ2

1340,304,O,308,H,304-O-308-H

1341,304,OD2,292,HZ1,304-OD2-292-HZ1

1342,304,OD1,292,HZ1,304-OD1-292-HZ1

1343,304,OD2,290,HE22,304-OD2-290-HE22

1344,304,OD2,270,HH,304-OD2-270-HH

1345,304,OD1,305,H,304-OD1-305-H

1346,304,O,307,H,304-O-307-H

1347,304,OD1,304,H,304-OD1-304-H

1348,305,O,259,HH,305-O-259-HH

1349,305,OE1,263,HE21,305-OE1-263-HE21

1350,305,OE2,270,HH,305-OE2-270-HH

1351,305,OE2,117,HD22,305-OE2-117-HD22

1352,305,OE2,305,H,305-OE2-305-H

1353,305,OE1,306,HZ1,305-OE1-306-HZ1

1354,305,OE1,259,HH,305-OE1-259-HH

1355,305,OE2,117,HD21,305-OE2-117-HD21

1356,305,OE1,306,HZ3,305-OE1-306-HZ3

1357,305,OE1,270,HH,305-OE1-270-HH

1358,305,OE2,309,HD22,305-OE2-309-HD22

1359,305,OE2,303,HG,305-OE2-303-HG

1360,305,OE1,117,HD21,305-OE1-117-HD21

1361,305,OE1,306,HZ2,305-OE1-306-HZ2

1362,305,OE1,119,HE21,305-OE1-119-HE21

1363,305,O,117,HD22,305-O-117-HD22

1364,305,O,309,HD22,305-O-309-HD22

1365,305,OE2,306,HZ2,305-OE2-306-HZ2

1366,305,OE1,263,HE22,305-OE1-263-HE22

1367,305,OE2,263,HE21,305-OE2-263-HE21

1368,305,OE1,305,H,305-OE1-305-H

1369,305,OE2,263,HE22,305-OE2-263-HE22

1370,305,O,119,HE22,305-O-119-HE22

1371,305,O,309,H,305-O-309-H

1372,305,OE1,119,HE22,305-OE1-119-HE22

1373,305,OE2,306,HZ1,305-OE2-306-HZ1

1374,305,OE1,117,HD22,305-OE1-117-HD22

1375,305,OE2,259,HH,305-OE2-259-HH

1376,305,OE1,303,HG,305-OE1-303-HG

1377,305,OE2,306,HZ3,305-OE2-306-HZ3

1378,306,O,134,HH12,306-O-134-HH12

1379,306,O,119,HE21,306-O-119-HE21

1380,306,O,309,HD21,306-O-309-HD21

1381,306,O,134,HE,306-O-134-HE

1382,306,O,119,HE22,306-O-119-HE22

1383,306,O,134,HH21,306-O-134-HH21

1384,306,O,134,HH22,306-O-134-HH22

1385,307,O,310,H,307-O-310-H

1386,307,O,134,HH21,307-O-134-HH21

1387,308,O,311,H,308-O-311-H

1388,308,O,310,H,308-O-310-H

1389,309,OD1,134,HH22,309-OD1-134-HH22

1390,309,OD1,134,HH21,309-OD1-134-HH21

1391,309,OD1,119,HE21,309-OD1-119-HE21

1392,309,O,313,H,309-O-313-H

1393,309,OD1,134,HH12,309-OD1-134-HH12

1394,309,O,309,HD22,309-O-309-HD22

1395,309,O,312,H,309-O-312-H

1396,309,OD1,117,HD22,309-OD1-117-HD22

1397,310,O,314,H,310-O-314-H

1398,310,O,313,H,310-O-313-H

1399,311,O,314,H,311-O-314-H

1400,311,O,315,H,311-O-315-H

1401,312,O,516,H13,312-O-516-H13

1402,312,O,316,H,312-O-316-H

1403,313,OD1,309,HD22,313-OD1-309-HD22

1404,313,OD2,121,H,313-OD2-121-H

1405,313,OD2,309,HD22,313-OD2-309-HD22

1406,313,OD1,313,H,313-OD1-313-H

1407,313,OD2,120,HG,313-OD2-120-HG

1408,313,OD1,516,H13,313-OD1-516-H13

1409,313,OD1,120,HG,313-OD1-120-HG

1410,313,OD1,116,HG,313-OD1-116-HG

1411,313,OD2,122,HG,313-OD2-122-HG

1412,313,OD1,122,HG,313-OD1-122-HG

1413,313,OD2,309,HD21,313-OD2-309-HD21

1414,313,O,317,H,313-O-317-H

1415,313,OD1,122,H,313-OD1-122-H

1416,313,OD2,116,HG,313-OD2-116-HG

1417,313,OD1,121,H,313-OD1-121-H

1418,313,OD2,122,H,313-OD2-122-H

1419,313,OD2,516,H13,313-OD2-516-H13

1420,314,O,318,H,314-O-318-H

1421,314,O,317,H,314-O-317-H

1422,315,O,317,H,315-O-317-H

1423,315,O,319,H,315-O-319-H

1424,316,O,516,H13,316-O-516-H13

1425,316,O,320,H,316-O-320-H

1426,317,O,322,H,317-O-322-H

1427,317,O,321,H,317-O-321-H

1428,317,O,320,H,317-O-320-H

1429,317,O,321,HG1,317-O-321-HG1

1430,318,O,322,H,318-O-322-H

1431,318,O,323,HG1,318-O-323-HG1

1432,318,O,323,H,318-O-323-H

1433,319,O,323,HG1,319-O-323-HG1

1434,319,O,221,HD22,319-O-221-HD22

1435,319,O,221,HD21,319-O-221-HD21

1436,319,O,222,HD22,319-O-222-HD22

1437,319,O,222,HD21,319-O-222-HD21

1438,320,OD1,222,H,320-OD1-222-H

1439,320,OD1,324,HG1,320-OD1-324-HG1

1440,320,O,324,H,320-O-324-H

1441,320,OD2,499,HZ1,320-OD2-499-HZ1

1442,320,OD2,499,HZ3,320-OD2-499-HZ3

1443,320,OD1,499,HZ1,320-OD1-499-HZ1

1444,320,OD2,187,HH,320-OD2-187-HH

1445,320,OD2,499,HZ2,320-OD2-499-HZ2

1446,320,OD1,221,HD21,320-OD1-221-HD21

1447,320,OD1,222,HD21,320-OD1-222-HD21

1448,320,OD2,222,HD21,320-OD2-222-HD21

1449,320,OD1,497,HG1,320-OD1-497-HG1

1450,320,OD2,221,HD21,320-OD2-221-HD21

1451,320,OD1,222,HD22,320-OD1-222-HD22

1452,320,OD2,324,HG1,320-OD2-324-HG1

1453,320,O,324,HG1,320-O-324-HG1

1454,320,OD1,499,HZ2,320-OD1-499-HZ2

1455,320,OD2,222,HD22,320-OD2-222-HD22

1456,320,OD2,497,HG1,320-OD2-497-HG1

1457,320,OD1,187,HH,320-OD1-187-HH

1458,320,OD1,499,HZ3,320-OD1-499-HZ3

1459,321,O,325,H,321-O-325-H

1460,321,OG1,322,H,321-OG1-322-H

1461,321,O,324,H,321-O-324-H

1462,322,O,325,H,322-O-325-H

1463,322,O,326,H,322-O-326-H

1464,323,O,327,HG,323-O-327-HG

1465,323,O,327,H,323-O-327-H

1466,324,OG1,497,HG1,324-OG1-497-HG1

1467,324,OG1,499,HZ3,324-OG1-499-HZ3

1468,324,O,328,H,324-O-328-H

1469,324,O,327,HG,324-O-327-HG

1470,324,OG1,499,HZ1,324-OG1-499-HZ1

1471,324,O,327,H,324-O-327-H

1472,324,OG1,499,HZ2,324-OG1-499-HZ2

1473,325,O,329,H,325-O-329-H

1474,325,O,329,HG,325-O-329-HG

1475,326,O,329,H,326-O-329-H

1476,326,O,329,HG,326-O-329-HG

1477,326,O,330,H,326-O-330-H

1478,327,O,331,H,327-O-331-H

1479,328,O,332,H,328-O-332-H

1480,328,O,331,H,328-O-331-H

1481,329,O,333,H,329-O-333-H

1482,330,O,333,H,330-O-333-H

1483,330,O,334,H,330-O-334-H

1484,331,O,334,H,331-O-334-H

1485,331,O,335,H,331-O-335-H

1486,332,O,336,H,332-O-336-H

1487,332,O,335,H,332-O-335-H

1488,332,O,336,HD22,332-O-336-HD22

1489,333,O,336,H,333-O-336-H

1490,333,O,340,HE21,333-O-340-HE21

1491,335,O,336,HD22,335-O-336-HD22

1492,336,OD1,338,H,336-OD1-338-H

1493,336,OD1,339,H,336-OD1-339-H

1494,336,O,340,H,336-O-340-H

1495,336,O,339,H,336-O-339-H

1496,337,O,340,H,337-O-340-H

1497,337,O,341,H,337-O-341-H

1498,338,O,342,H,338-O-342-H

1499,339,O,343,H,339-O-343-H

1500,339,O,342,H,339-O-342-H

1501,340,OE1,344,HE21,340-OE1-344-HE21

1502,340,O,343,H,340-O-343-H

1503,340,O,344,H,340-O-344-H

1504,340,OE1,344,HE22,340-OE1-344-HE22

1505,340,OE1,480,H,340-OE1-480-H

1506,341,O,345,H,341-O-345-H

1507,342,O,342,HZ2,342-O-342-HZ2

1508,342,O,346,H,342-O-346-H

1509,342,O,345,H,342-O-345-H

1510,343,O,347,H,343-O-347-H

1511,343,O,346,H,343-O-346-H

1512,344,OE1,341,HH11,344-OE1-341-HH11

1513,344,O,511,HH22,344-O-511-HH22

1514,344,O,348,H,344-O-348-H

1515,344,OE1,341,HE,344-OE1-341-HE

1516,344,O,511,HH12,344-O-511-HH12

1517,344,OE1,341,HH21,344-OE1-341-HH21

1518,345,OE2,341,HH12,345-OE2-341-HH12

1519,345,OE2,342,HZ1,345-OE2-342-HZ1

1520,345,OE1,342,HZ3,345-OE1-342-HZ3

1521,345,OE2,341,HH11,345-OE2-341-HH11

1522,345,OE1,342,HZ1,345-OE1-342-HZ1

1523,345,OE2,341,HE,345-OE2-341-HE

1524,345,OE2,342,HZ2,345-OE2-342-HZ2

1525,345,O,348,H,345-O-348-H

1526,345,OE1,341,HH22,345-OE1-341-HH22

1527,345,OE2,341,HH22,345-OE2-341-HH22

1528,345,OE1,341,HH21,345-OE1-341-HH21

1529,345,OE1,341,HH12,345-OE1-341-HH12

1530,345,OE1,341,HE,345-OE1-341-HE

1531,345,OE2,341,HH21,345-OE2-341-HH21

1532,345,O,349,H,345-O-349-H

1533,345,OE1,341,HH11,345-OE1-341-HH11

1534,345,OE2,342,HZ3,345-OE2-342-HZ3

1535,345,OE1,342,HZ2,345-OE1-342-HZ2

1536,345,O,349,HG1,345-O-349-HG1

1537,346,OE2,367,H,346-OE2-367-H

1538,346,O,350,H,346-O-350-H

1539,346,OE1,349,HG1,346-OE1-349-HG1

1540,346,OE1,342,HZ1,346-OE1-342-HZ1

1541,346,OE1,342,HZ2,346-OE1-342-HZ2

1542,346,OE1,368,H,346-OE1-368-H

1543,346,OE2,342,HZ1,346-OE2-342-HZ1

1544,346,OE2,342,HZ3,346-OE2-342-HZ3

1545,346,OE1,367,H,346-OE1-367-H

1546,346,O,349,H,346-O-349-H

1547,346,OE2,342,HZ2,346-OE2-342-HZ2

1548,346,OE1,342,HZ3,346-OE1-342-HZ3

1549,347,O,351,H,347-O-351-H

1550,347,O,352,H,347-O-352-H

1551,348,OD2,353,HE,348-OD2-353-HE

1552,348,OD1,511,HH22,348-OD1-511-HH22

1553,348,O,352,H,348-O-352-H

1554,348,OD2,511,HH22,348-OD2-511-HH22

1555,348,OD2,353,HH21,348-OD2-353-HH21

1556,348,OD2,511,HH12,348-OD2-511-HH12

1557,348,OD1,353,HE,348-OD1-353-HE

1558,348,OD1,353,HH21,348-OD1-353-HH21

1559,348,OD1,511,HH12,348-OD1-511-HH12

1560,350,O,364,HE2,350-O-364-HE2

1561,350,O,355,HH21,350-O-355-HH21

1562,351,O,354,H,351-O-354-H

1563,351,O,354,HG,351-O-354-HG

1564,352,O,354,H,352-O-354-H

1565,352,O,476,HE21,352-O-476-HE21

1566,353,O,355,H,353-O-355-H

1567,354,O,354,HG,354-O-354-HG

1568,354,OG,355,H,354-OG-355-H

1569,355,O,476,HE22,355-O-476-HE22

1570,356,O,355,HH11,356-O-355-HH11

1571,358,O,361,H,358-O-361-H

1572,359,O,465,HE1,359-O-465-HE1

1573,359,O,362,H,359-O-362-H

1574,360,O,363,H,360-O-363-H

1575,360,OG,358,HE,360-OG-358-HE

1576,360,O,363,HG,360-O-363-HG

1577,360,O,360,HG,360-O-360-HG

1578,361,OD2,355,HH22,361-OD2-355-HH22

1579,361,O,365,H,361-O-365-H

1580,361,OD1,355,HH22,361-OD1-355-HH22

1581,361,OD2,358,HH21,361-OD2-358-HH21

1582,361,OD1,358,HE,361-OD1-358-HE

1583,361,OD1,358,HH11,361-OD1-358-HH11

1584,361,OD1,358,HH21,361-OD1-358-HH21

1585,361,OD2,358,H,361-OD2-358-H

1586,361,OD1,360,HG,361-OD1-360-HG

1587,361,OD2,355,HH12,361-OD2-355-HH12

1588,361,O,364,H,361-O-364-H

1589,361,OD2,358,HE,361-OD2-358-HE

1590,361,OD1,364,HE2,361-OD1-364-HE2

1591,362,O,365,H,362-O-365-H

1592,364,ND1,363,HG,364-ND1-363-HG

1593,365,O,368,H,365-O-368-H

1594,365,O,369,H,365-O-369-H

1595,366,O,370,H,366-O-370-H

1596,366,O,369,H,366-O-369-H

1597,367,O,370,H,367-O-370-H

1598,367,O,371,H,367-O-371-H

1599,368,O,371,H,368-O-371-H

1600,368,O,372,H,368-O-372-H

1601,369,OE1,362,HE,369-OE1-362-HE

1602,369,OE2,465,HE1,369-OE2-465-HE1

1603,369,OE2,464,HH21,369-OE2-464-HH21

1604,369,OE2,362,HH11,369-OE2-362-HH11

1605,369,OE1,464,HE,369-OE1-464-HE

1606,369,O,373,H,369-O-373-H

1607,369,OE1,465,HE1,369-OE1-465-HE1

1608,369,OE2,464,HE,369-OE2-464-HE

1609,369,O,372,H,369-O-372-H

1610,369,OE2,362,HH12,369-OE2-362-HH12

1611,369,OE2,362,HE,369-OE2-362-HE

1612,369,OE2,464,HH22,369-OE2-464-HH22

1613,369,OE1,362,HH21,369-OE1-362-HH21

1614,369,OE1,464,HH21,369-OE1-464-HH21

1615,369,OE2,362,HH21,369-OE2-362-HH21

1616,369,OE1,362,HH11,369-OE1-362-HH11

1617,370,O,374,H,370-O-374-H

1618,371,O,375,H,371-O-375-H

1619,371,O,375,HG1,371-O-375-HG1

1620,372,O,375,H,372-O-375-H

1621,372,O,376,H,372-O-376-H

1622,373,O,377,H,373-O-377-H

1623,374,OE1,431,HH22,374-OE1-431-HH22

1624,374,OE2,431,HH12,374-OE2-431-HH12

1625,374,OE2,431,HH11,374-OE2-431-HH11

1626,374,OE1,428,H,374-OE1-428-H

1627,374,O,378,H,374-O-378-H

1628,374,OE1,431,HH12,374-OE1-431-HH12

1629,374,OE2,377,HH21,374-OE2-377-HH21

1630,374,OE1,431,HH11,374-OE1-431-HH11

1631,374,OE1,377,HH21,374-OE1-377-HH21

1632,374,OE2,377,HE,374-OE2-377-HE

1633,374,OE1,377,HE,374-OE1-377-HE

1634,374,OE2,431,HH22,374-OE2-431-HH22

1635,374,OE2,428,H,374-OE2-428-H

1636,375,O,379,H,375-O-379-H

1637,375,OG1,329,HG,375-OG1-329-HG

1638,375,O,379,HG,375-O-379-HG

1639,376,O,380,H,376-O-380-H

1640,377,O,416,HE2,377-O-416-HE2

1641,377,O,380,HG,377-O-380-HG

1642,378,ND1,332,HH,378-ND1-332-HH

1643,378,O,416,HE2,378-O-416-HE2

1644,379,O,412,HE1,379-O-412-HE1

1645,379,OG,381,H,379-OG-381-H

1646,380,O,380,HG,380-O-380-HG

1647,380,O,382,H,380-O-382-H

1648,380,OG,416,HE2,380-OG-416-HE2

1649,380,OG,415,HD22,380-OG-415-HD22

1650,381,O,498,H,381-O-498-H

1651,381,O,497,HG1,381-O-497-HG1

1652,382,O,411,HE21,382-O-411-HE21

1653,383,O,411,H,383-O-411-H

1654,383,O,412,H,383-O-412-H

1655,385,O,230,HG,385-O-230-HG

1656,385,OG1,409,H,385-OG1-409-H

1657,386,O,385,HG1,386-O-385-HG1

1658,386,O,106,HH12,386-O-106-HH12

1659,386,O,106,HH11,386-O-106-HH11

1660,387,O,106,HH11,387-O-106-HH11

1661,388,O,407,H,388-O-407-H

1662,388,NE2,385,HG1,388-NE2-385-HG1

1663,388,O,389,HG,388-O-389-HG

1664,389,O,105,H,389-O-105-H

1665,389,O,389,HG,389-O-389-HG

1666,389,O,391,H,389-O-391-H

1667,390,OG1,405,H,390-OG1-405-H

1668,390,O,404,H,390-O-404-H

1669,390,OG1,404,H,390-OG1-404-H

1670,391,OG1,104,HZ3,391-OG1-104-HZ3

1671,391,O,403,HZ2,391-O-403-HZ2

1672,391,OG1,105,H,391-OG1-105-H

1673,391,O,403,HZ1,391-O-403-HZ1

1674,391,OG1,104,HZ2,391-OG1-104-HZ2

1675,391,O,403,HZ3,391-O-403-HZ3

1676,391,OG1,104,H,391-OG1-104-H

1677,391,OG1,104,HZ1,391-OG1-104-HZ1

1678,393,O,392,HH11,393-O-392-HH11

1679,393,OD1,400,HH,393-OD1-400-HH

1680,393,OD1,393,H,393-OD1-393-H

1681,393,OD2,393,H,393-OD2-393-H

1682,393,OD1,403,HZ3,393-OD1-403-HZ3

1683,393,OD1,403,H,393-OD1-403-H

1684,393,OD2,403,H,393-OD2-403-H

1685,393,OD2,403,HZ1,393-OD2-403-HZ1

1686,393,OD2,400,HH,393-OD2-400-HH

1687,393,OD1,403,HZ2,393-OD1-403-HZ2

1688,393,OD2,403,HZ2,393-OD2-403-HZ2

1689,393,OD1,403,HZ1,393-OD1-403-HZ1

1690,393,OD2,403,HZ3,393-OD2-403-HZ3

1691,394,O,401,H,394-O-401-H

1692,394,O,395,HG,394-O-395-HG

1693,394,OG1,395,H,394-OG1-395-H

1694,394,OG1,99,HE22,394-OG1-99-HE22

1695,394,OG1,99,HE21,394-OG1-99-HE21

1696,395,O,99,HE22,395-O-99-HE22

1697,395,O,99,HE21,395-O-99-HE21

1698,396,O,398,H,396-O-398-H

1699,396,O,399,H,396-O-399-H

1700,397,O,38,HZ3,397-O-38-HZ3

1701,398,O,38,H,398-O-38-H

1702,398,O,38,HZ3,398-O-38-HZ3

1703,398,O,38,HZ1,398-O-38-HZ1

1704,398,O,38,HZ2,398-O-38-HZ2

1705,399,O,395,HG,399-O-395-HG

1706,399,O,396,H,399-O-396-H

1707,400,O,39,HD22,400-O-39-HD22

1708,401,O,394,H,401-O-394-H

1709,402,O,390,HG1,402-O-390-HG1

1710,403,O,405,HH11,403-O-405-HH11

1711,404,O,405,HH11,404-O-405-HH11

1712,405,O,390,HG1,405-O-390-HG1

1713,405,O,389,HG,405-O-389-HG

1714,405,O,390,H,405-O-390-H

1715,406,O,84,H,406-O-84-H

1716,407,O,388,H,407-O-388-H

1717,408,O,86,H,408-O-86-H

1718,409,O,385,H,409-O-385-H

1719,409,O,411,H,409-O-411-H

1720,410,OD1,413,H,410-OD1-413-H

1721,410,OD1,411,H,410-OD1-411-H

1722,410,O,413,H,410-O-413-H

1723,410,OD1,412,H,410-OD1-412-H

1724,411,OE1,451,H,411-OE1-451-H

1725,411,O,415,H,411-O-415-H

1726,411,O,414,H,411-O-414-H

1727,411,O,415,HD22,411-O-415-HD22

1728,412,O,416,H,412-O-416-H

1729,412,O,414,H,412-O-414-H

1730,412,O,415,H,412-O-415-H

1731,413,OE1,67,HG,413-OE1-67-HG

1732,413,O,413,HE21,413-O-413-HE21

1733,413,NE2,87,HG,413-NE2-87-HG

1734,413,O,416,H,413-O-416-H

1735,413,OE1,68,HE21,413-OE1-68-HE21

1736,413,NE2,68,HE22,413-NE2-68-HE22

1737,413,OE1,87,HG,413-OE1-87-HG

1738,413,OE1,410,HD22,413-OE1-410-HD22

1739,413,NE2,68,HE21,413-NE2-68-HE21

1740,413,O,417,H,413-O-417-H

1741,414,O,421,HE1,414-O-421-HE1

1742,414,O,417,H,414-O-417-H

1743,415,OD1,448,H,415-OD1-448-H

1744,415,O,377,HH11,415-O-377-HH11

1745,416,ND1,377,HH12,416-ND1-377-HH12

1746,417,OD2,419,HZ3,417-OD2-419-HZ3

1747,417,OD1,418,HE21,417-OD1-418-HE21

1748,417,OD2,420,H,417-OD2-420-H

1749,417,OD2,413,HE22,417-OD2-413-HE22

1750,417,OD1,413,HE21,417-OD1-413-HE21

1751,417,OD1,68,HE22,417-OD1-68-HE22

1752,417,OD2,419,HZ1,417-OD2-419-HZ1

1753,417,OD1,419,H,417-OD1-419-H

1754,417,OD1,420,H,417-OD1-420-H

1755,417,O,420,H,417-O-420-H

1756,417,OD2,68,HE22,417-OD2-68-HE22

1757,417,OD1,419,HZ3,417-OD1-419-HZ3

1758,417,OD2,419,HZ2,417-OD2-419-HZ2

1759,417,O,421,H,417-O-421-H

1760,417,OD1,419,HZ2,417-OD1-419-HZ2

1761,417,OD1,419,HZ1,417-OD1-419-HZ1

1762,417,OD2,419,H,417-OD2-419-H

1763,417,OD2,413,HE21,417-OD2-413-HE21

1764,418,OE1,68,HE22,418-OE1-68-HE22

1765,418,OE1,423,H,418-OE1-423-H

1766,418,OE1,419,H,418-OE1-419-H

1767,418,NE2,418,H,418-NE2-418-H

1768,418,OE1,418,H,418-OE1-418-H

1769,418,O,420,H,418-O-420-H

1770,418,O,418,HE21,418-O-418-HE21

1771,420,O,446,HZ1,420-O-446-HZ1

1772,420,O,422,H,420-O-422-H

1773,420,O,446,HZ2,420-O-446-HZ2

1774,420,O,446,HZ3,420-O-446-HZ3

1775,421,O,418,HE21,421-O-418-HE21

1776,422,O,431,HE,422-O-431-HE

1777,422,O,431,HH21,422-O-431-HH21

1778,423,OD1,426,H,423-OD1-426-H

1779,423,O,431,HH22,423-O-431-HH22

1780,423,OD1,418,HE22,423-OD1-418-HE22

1781,423,O,426,H,423-O-426-H

1782,423,O,431,HH21,423-O-431-HH21

1783,423,OD1,425,H,423-OD1-425-H

1784,423,O,423,HD22,423-O-423-HD22

1785,423,OD1,425,HG,423-OD1-425-HG

1786,424,O,377,HH12,424-O-377-HH12

1787,424,O,377,HH22,424-O-377-HH22

1788,425,O,425,HG,425-O-425-HG

1789,425,O,378,HE2,425-O-378-HE2

1790,426,OE2,423,HD22,426-OE2-423-HD22

1791,426,O,431,HH12,426-O-431-HH12

1792,426,O,431,HH21,426-O-431-HH21

1793,426,O,431,HH22,426-O-431-HH22

1794,426,OE1,427,H,426-OE1-427-H

1795,426,OE1,423,HD21,426-OE1-423-HD21

1796,426,OE2,425,HG,426-OE2-425-HG

1797,426,OE2,427,H,426-OE2-427-H

1798,426,OE2,423,HD21,426-OE2-423-HD21

1799,426,OE1,423,HD22,426-OE1-423-HD22

1800,426,OE1,425,HG,426-OE1-425-HG

1801,427,O,338,HH21,427-O-338-HH21

1802,428,O,431,H,428-O-431-H

1803,428,O,431,HH11,428-O-431-HH11

1804,429,O,432,H,429-O-432-H

1805,429,O,433,H,429-O-433-H

1806,430,OE1,430,H,430-OE1-430-H

1807,430,OE1,342,HZ1,430-OE1-342-HZ1

1808,430,OE2,367,HH,430-OE2-367-HH

1809,430,OE2,430,H,430-OE2-430-H

1810,430,OE1,338,HH12,430-OE1-338-HH12

1811,430,OE1,342,HZ3,430-OE1-342-HZ3

1812,430,OE2,338,HH22,430-OE2-338-HH22

1813,430,OE2,342,HZ3,430-OE2-342-HZ3

1814,430,OE1,342,HZ2,430-OE1-342-HZ2

1815,430,O,433,H,430-O-433-H

1816,430,OE2,342,HZ1,430-OE2-342-HZ1

1817,430,OE1,338,HH22,430-OE1-338-HH22

1818,430,OE2,342,HZ2,430-OE2-342-HZ2

1819,430,OE2,338,HH12,430-OE2-338-HH12

1820,430,OE1,367,HH,430-OE1-367-HH

1821,432,O,440,H,432-O-440-H

1822,434,OG1,436,H,434-OG1-436-H

1823,434,OG1,437,H,434-OG1-437-H

1824,434,OG1,438,H,434-OG1-438-H

1825,434,O,437,H,434-O-437-H

1826,436,OD2,434,HG1,436-OD2-434-HG1

1827,436,OD2,438,H,436-OD2-438-H

1828,436,OD1,434,HG1,436-OD1-434-HG1

1829,436,OD1,436,H,436-OD1-436-H

1830,436,OD1,438,H,436-OD1-438-H

1831,436,O,438,H,436-O-438-H

1832,436,OD2,436,H,436-OD2-436-H

1833,438,O,434,HG1,438-O-434-HG1

1834,438,O,434,H,438-O-434-H

1835,439,O,441,HZ3,439-O-441-HZ3

1836,439,O,441,H,439-O-441-H

1837,439,O,441,HZ1,439-O-441-HZ1

1838,439,O,441,HZ2,439-O-441-HZ2

1839,440,OD2,442,H,440-OD2-442-H

1840,440,O,444,HG,440-O-444-HG

1841,440,OD2,441,H,440-OD2-441-H

1842,440,OD1,442,H,440-OD1-442-H

1843,440,O,443,H,440-O-443-H

1844,440,O,444,H,440-O-444-H

1845,440,OD2,443,H,440-OD2-443-H

1846,440,O,442,H,440-O-442-H

1847,440,OD1,443,H,440-OD1-443-H

1848,441,O,444,H,441-O-444-H

1849,441,O,445,H,441-O-445-H

1850,441,O,444,HG,441-O-444-HG

1851,442,O,445,H,442-O-445-H

1852,442,O,446,H,442-O-446-H

1853,442,O,446,HZ3,442-O-446-HZ3

1854,442,O,446,HZ2,442-O-446-HZ2

1855,442,O,446,HZ1,442-O-446-HZ1

1856,443,O,447,H,443-O-447-H

1857,443,O,446,H,443-O-446-H

1858,444,O,464,HH22,444-O-464-HH22

1859,444,O,447,H,444-O-447-H

1860,444,OG,464,HH22,444-OG-464-HH22

1861,445,OE2,446,HZ3,445-OE2-446-HZ3

1862,445,OE1,441,HZ3,445-OE1-441-HZ3

1863,445,OE2,93,HE,445-OE2-93-HE

1864,445,OE1,441,HZ1,445-OE1-441-HZ1

1865,445,OE2,441,HZ2,445-OE2-441-HZ2

1866,445,OE2,444,HG,445-OE2-444-HG

1867,445,OE1,93,HH12,445-OE1-93-HH12

1868,445,OE2,441,HZ1,445-OE2-441-HZ1

1869,445,OE1,441,HZ2,445-OE1-441-HZ2

1870,445,OE2,93,HH22,445-OE2-93-HH22

1871,445,OE1,93,HE,445-OE1-93-HE

1872,445,OE2,464,HH12,445-OE2-464-HH12

1873,445,OE1,464,HH22,445-OE1-464-HH22

1874,445,OE2,441,HZ3,445-OE2-441-HZ3

1875,445,OE1,93,HH22,445-OE1-93-HH22

1876,445,OE2,93,HH12,445-OE2-93-HH12

1877,445,OE1,464,HH12,445-OE1-464-HH12

1878,445,OE2,93,HH21,445-OE2-93-HH21

1879,445,O,93,HH22,445-O-93-HH22

1880,445,OE1,93,HH21,445-OE1-93-HH21

1881,445,OE1,444,HG,445-OE1-444-HG

1882,445,OE2,464,HH22,445-OE2-464-HH22

1883,446,O,448,H,446-O-448-H

1884,448,O,415,HD21,448-O-415-HD21

1885,449,O,451,H,449-O-451-H

1886,449,O,411,HE22,449-O-411-HE22

1887,450,O,457,H,450-O-457-H

1888,450,O,456,HZ3,450-O-456-HZ3

1889,450,O,456,HZ2,450-O-456-HZ2

1890,451,O,456,HZ1,451-O-456-HZ1

1891,451,O,456,HZ3,451-O-456-HZ3

1892,451,O,456,HZ2,451-O-456-HZ2

1893,453,O,456,HZ1,453-O-456-HZ1

1894,453,O,456,HZ3,453-O-456-HZ3

1895,453,O,456,HZ2,453-O-456-HZ2

1896,453,O,456,H,453-O-456-H

1897,455,O,135,HH12,455-O-135-HH12

1898,455,NH1,106,HE,455-NH1-106-HE

1899,456,O,135,HH22,456-O-135-HH22

1900,456,O,135,HH12,456-O-135-HH12

1901,457,O,456,HZ1,457-O-456-HZ1

1902,457,O,456,HZ2,457-O-456-HZ2

1903,457,O,456,HZ3,457-O-456-HZ3

1904,458,O,139,HE22,458-O-139-HE22

1905,458,O,139,HE21,458-O-139-HE21

1906,458,O,461,HG1,458-O-461-HG1

1907,458,O,461,H,458-O-461-H

1908,459,O,462,H,459-O-462-H

1909,459,O,463,H,459-O-463-H

1910,460,OE2,461,HG1,460-OE2-461-HG1

1911,460,O,464,H,460-O-464-H

1912,460,OE2,456,HZ3,460-OE2-456-HZ3

1913,460,OE1,464,HH11,460-OE1-464-HH11

1914,460,OE2,456,HZ1,460-OE2-456-HZ1

1915,460,OE2,464,HH11,460-OE2-464-HH11

1916,460,OE1,461,HG1,460-OE1-461-HG1

1917,460,O,464,HE,460-O-464-HE

1918,460,OE1,464,HE,460-OE1-464-HE

1919,460,OE2,456,HZ2,460-OE2-456-HZ2

1920,460,OE1,456,HZ3,460-OE1-456-HZ3

1921,460,OE1,456,HZ2,460-OE1-456-HZ2

1922,460,OE1,464,HH21,460-OE1-464-HH21

1923,460,OE2,464,HE,460-OE2-464-HE

1924,460,OE2,464,HH21,460-OE2-464-HH21

1925,460,OE1,456,HZ1,460-OE1-456-HZ1

1926,461,O,143,HZ1,461-O-143-HZ1

1927,461,OG1,139,HE22,461-OG1-139-HE22

1928,461,OG1,139,HE21,461-OG1-139-HE21

1929,461,O,461,HG1,461-O-461-HG1

1930,461,OG1,143,HZ1,461-OG1-143-HZ1

1931,461,O,143,HZ2,461-O-143-HZ2

1932,462,O,466,H,462-O-466-H

1933,462,O,465,H,462-O-465-H

1934,463,O,466,H,463-O-466-H

1935,463,O,467,H,463-O-467-H

1936,464,O,362,HH11,464-O-362-HH11

1937,464,O,468,H,464-O-468-H

1938,464,O,362,HH22,464-O-362-HH22

1939,464,O,362,HH12,464-O-362-HH12

1940,465,NE1,362,HH11,465-NE1-362-HH11

1941,465,O,468,H,465-O-468-H

1942,465,O,469,H,465-O-469-H

1943,466,O,469,H,466-O-469-H

1944,466,O,470,H,466-O-470-H

1945,467,O,471,H,467-O-471-H

1946,468,O,471,H,468-O-471-H

1947,468,O,472,H,468-O-472-H

1948,469,O,473,H,469-O-473-H

1949,470,O,474,H,470-O-474-H

1950,471,O,475,H,471-O-475-H

1951,471,O,474,H,471-O-474-H

1952,472,O,475,H,472-O-475-H

1953,472,O,476,H,472-O-476-H

1954,473,O,477,HH11,473-O-477-HH11

1955,473,O,477,HH21,473-O-477-HH21

1956,473,O,476,H,473-O-476-H

1957,473,O,477,HE,473-O-477-HE

1958,474,O,477,H,474-O-477-H

1959,474,O,478,H,474-O-478-H

1960,475,O,511,HH21,475-O-511-HH21

1961,475,O,511,HE,475-O-511-HE

1962,475,O,344,HE21,475-O-344-HE21

1963,476,O,511,HE,476-O-511-HE

1964,476,O,512,HG,476-O-512-HG

1965,476,O,511,HH21,476-O-511-HH21

1966,476,O,353,HE,476-O-353-HE

1967,477,O,511,H,477-O-511-H

1968,478,O,344,HE21,478-O-344-HE21

1969,479,OE1,512,H,479-OE1-512-H

1970,479,OE1,341,HE,479-OE1-341-HE

1971,479,OE1,341,HH12,479-OE1-341-HH12

1972,479,OE2,341,HH22,479-OE2-341-HH22

1973,479,OE2,341,HH12,479-OE2-341-HH12

1974,479,OE1,341,HH22,479-OE1-341-HH22

1975,479,O,509,H,479-O-509-H

1976,479,OE2,512,H,479-OE2-512-H

1977,479,OE1,341,HH11,479-OE1-341-HH11

1978,479,OE1,512,HG,479-OE1-512-HG

1979,479,O,509,HE21,479-O-509-HE21

1980,479,OE2,512,HG,479-OE2-512-HG

1981,479,OE2,509,HE21,479-OE2-509-HE21

1982,479,OE1,344,HE22,479-OE1-344-HE22

1983,479,OE1,509,HE21,479-OE1-509-HE21

1984,479,OE2,341,HE,479-OE2-341-HE

1985,479,OE2,341,HH21,479-OE2-341-HH21

1986,479,OE1,341,HH21,479-OE1-341-HH21

1987,479,OE2,344,HE22,479-OE2-344-HE22

1988,480,O,340,HE22,480-O-340-HE22

1989,481,O,507,H,481-O-507-H

1990,481,OG,507,HE21,481-OG-507-HE21

1991,481,OG,482,H,481-OG-482-H

1992,481,OG,509,HE22,481-OG-509-HE22

1993,482,O,481,HG,482-O-481-HG

1994,483,O,487,HZ1,483-O-487-HZ1

1995,483,O,487,HZ2,483-O-487-HZ2

1996,483,O,485,H,483-O-485-H

1997,483,O,486,H,483-O-486-H

1998,483,O,487,HZ3,483-O-487-HZ3

1999,484,O,486,H,484-O-486-H

2000,485,O,487,HZ3,485-O-487-HZ3

2001,485,O,487,HZ1,485-O-487-HZ1

2002,485,O,487,HZ2,485-O-487-HZ2

2003,486,O,488,H,486-O-488-H

2004,486,O,487,HZ1,486-O-487-HZ1

2005,486,O,505,HE2,486-O-505-HE2

2006,487,O,505,HE2,487-O-505-HE2

2007,487,O,489,H,487-O-489-H

2008,488,O,487,HZ1,488-O-487-HZ1

2009,488,O,487,HZ2,488-O-487-HZ2

2010,488,O,487,HZ3,488-O-487-HZ3

2011,489,OD1,487,HZ3,489-OD1-487-HZ3

2012,489,OD1,491,HG1,489-OD1-491-HG1

2013,489,OD2,491,H,489-OD2-491-H

2014,489,O,491,H,489-O-491-H

2015,489,OD1,487,HZ1,489-OD1-487-HZ1

2016,489,OD2,491,HG1,489-OD2-491-HG1

2017,489,O,491,HG1,489-O-491-HG1

2018,489,OD1,489,H,489-OD1-489-H

2019,489,O,501,H,489-O-501-H

2020,489,OD2,490,H,489-OD2-490-H

2021,489,OD2,487,HZ3,489-OD2-487-HZ3

2022,489,OD1,491,H,489-OD1-491-H

2023,489,OD1,487,HZ2,489-OD1-487-HZ2

2024,489,OD1,490,H,489-OD1-490-H

2025,490,O,501,H,490-O-501-H

2026,490,O,491,HG1,490-O-491-HG1

2027,491,O,501,H,491-O-501-H

2028,492,O,57,HZ2,492-O-57-HZ2

2029,492,O,58,HD21,492-O-58-HD21

2030,493,O,499,H,493-O-499-H

2031,494,OH,230,HG,494-OH-230-HG

2032,494,O,222,HD22,494-O-222-HD22

2033,496,O,497,HG1,496-O-497-HG1

2034,497,OG1,499,HZ3,497-OG1-499-HZ3

2035,497,OG1,324,HG1,497-OG1-324-HG1

2036,497,O,499,HZ1,497-O-499-HZ1

2037,497,O,499,HZ3,497-O-499-HZ3

2038,497,O,499,HZ2,497-O-499-HZ2

2039,497,OG1,499,HZ1,497-OG1-499-HZ1

2040,497,OG1,499,HZ2,497-OG1-499-HZ2

2041,498,O,328,HE1,498-O-328-HE1

2042,499,O,493,H,499-O-493-H

2043,500,NE2,327,HG,500-NE2-327-HG

2044,500,NE2,502,H,500-NE2-502-H

2045,500,NE2,503,H,500-NE2-503-H

2046,500,O,500,HD1,500-O-500-HD1

2047,500,O,502,H,500-O-502-H

2048,501,O,500,HD1,501-O-500-HD1

2049,501,O,503,H,501-O-503-H

2050,502,O,487,HZ2,502-O-487-HZ2

2051,502,O,185,HD22,502-O-185-HD22

2052,502,O,487,HZ1,502-O-487-HZ1

2053,502,O,504,H,502-O-504-H

2054,502,O,185,HD21,502-O-185-HD21

2055,503,O,505,H,503-O-505-H

2056,503,O,185,HD22,503-O-185-HD22

2057,504,OE1,188,HH22,504-OE1-188-HH22

2058,504,OE1,185,HD22,504-OE1-185-HD22

2059,504,OE2,188,HH21,504-OE2-188-HH21

2060,504,OE1,188,HH12,504-OE1-188-HH12

2061,504,OE2,188,HH12,504-OE2-188-HH12

2062,504,OE2,188,HE,504-OE2-188-HE

2063,504,OE1,188,HH21,504-OE1-188-HH21

2064,504,OE1,188,HE,504-OE1-188-HE

2065,504,OE2,185,HD22,504-OE2-185-HD22

2066,504,OE2,188,HH22,504-OE2-188-HH22

2067,505,ND1,505,H,505-ND1-505-H

2068,505,ND1,504,H,505-ND1-504-H

2069,506,O,184,H,506-O-184-H

2070,507,OE1,481,HG,507-OE1-481-HG

2071,507,OE1,183,HE2,507-OE1-183-HE2

2072,507,O,481,H,507-O-481-H

2073,507,OE1,505,HE2,507-OE1-505-HE2

2074,507,OE1,509,HE22,507-OE1-509-HE22

2075,508,O,509,HE21,508-O-509-HE21

2076,509,OE1,481,HG,509-OE1-481-HG

2077,509,O,479,H,509-O-479-H

2078,509,OE1,175,HE22,509-OE1-175-HE22

2079,509,OE1,510,H,509-OE1-510-H

2080,509,OE1,507,HE21,509-OE1-507-HE21

2081,510,O,509,HE21,510-O-509-HE21

2082,511,O,512,HG,511-O-512-HG

2083,511,NH1,353,HH21,511-NH1-353-HH21

2084,511,O,353,HH12,511-O-353-HH12

2085,511,O,353,HH22,511-O-353-HH22

2086,512,OG,353,HH11,512-OG-353-HH11

2087,512,O,353,HH22,512-O-353-HH22

2088,512,O,353,HH12,512-O-353-HH12

2089,512,OXT,353,HH12,512-OXT-353-HH12

2090,512,OXT,353,HH22,512-OXT-353-HH22

2091,512,O,353,HH11,512-O-353-HH11

2092,512,OXT,511,HH11,512-OXT-511-HH11

2093,512,OXT,353,HH11,512-OXT-353-HH11

2094,512,OG,353,HH12,512-OG-353-HH12

2095,512,O,512,HG,512-O-512-HG

2096,512,OXT,512,HG,512-OXT-512-HG

2097,513,O2A,106,HH22,513-O2A-106-HH22

2098,513,O2D,106,HH21,513-O2D-106-HH21

2099,513,O1D,131,HE1,513-O1D-131-HE1

2100,513,O1D,455,HH11,513-O1D-455-HH11

2101,513,O1A,452,H,513-O1A-452-H

2102,513,O2A,106,HH12,513-O2A-106-HH12

2103,513,O1D,135,HH11,513-O1D-135-HH11

2104,513,O2D,106,HE,513-O2D-106-HE

2105,513,O2A,388,HD1,513-O2A-388-HD1

2106,513,O2D,455,HH11,513-O2D-455-HH11

2107,515,O1,516,H9,515-O1-516-H9
